# Electricity-driven asymmetric bromocyclization enabled by chiral phosphate anion phase-transfer catalysis

**DOI:** 10.1038/s41467-023-36000-6

**Published:** 2023-01-23

**Authors:** Xuefeng Tan, Qingli Wang, Jianwei Sun

**Affiliations:** 1 Department of Chemistry, Energy Institute, Institute for Advanced Study, and the Hong Kong Branch of Chinese National Engineering Research Centre for Tissue Restoration & Reconstruction, The Hong Kong University of Science and Technology (HKUST), Clear Water Bay, Kowloon, Hong Kong SAR China; 2 HKUST Shenzhen Research Institute, No. 9 Yuexing 1st Rd, 518057 Shenzhen, China; 3 Shenzhen Bay Laboratory, 518107 Shenzhen, China

**Keywords:** Asymmetric synthesis, Organocatalysis

## Abstract

Electricity-driven asymmetric catalysis is an emerging powerful tool in organic synthesis. However, asymmetric induction so far has mainly relied on forming strong bonds with a chiral catalyst. Asymmetry induced by weak interactions with a chiral catalyst in an electrochemical medium remains challenging due to compatibility issues related to solvent polarity, electrolyte interference, etc. Enabled by a properly designed phase-transfer strategy, here we have achieved two efficient electricity-driven catalytic asymmetric bromocyclization processes induced by weak ion-pairing interaction. The combined use of a phase-transfer catalyst and a chiral phosphate catalyst, together with NaBr as the bromine source, constitutes the key advantages over the conventional chemical oxidation approach. Synergy over multiple events, including anodic oxidation, ion exchange, phase transfer, asymmetric bromination, and inhibition of Br_2_ decomposition by NaHCO_3_, proved critical to the success.

## Abstract (web summary)

Asymmetric electrochemical synthesis via ion-pairing catalysis is challenging due to interference of the electrolyte with the ion-pairing catalyst. Here, the authors describe a biphasic system to enable the asymmetric ion-pairing catalysis under electrochemical conditions.

## Introduction

Organic reactions driven by electricity has emerged as one of the most attractive tools in modern practical organic synthesis owing to their many “green” features, including atom economy, environmental benignity, and mild conditions^1–14^. The direct oxidation and reduction by the electric current with tunable redox potential constitute the most prominent advantage over conventional processes using stoichiometric chemical oxidants and reductants. Meanwhile, asymmetric synthesis, which provides expedient access to the valuable single-handed chiral molecules, holds an important position in modern organic synthesis^15,16^. Therefore, merging electrochemistry with asymmetric synthesis represents a highly desirable and enabling synthetic tool.

In the past few decades, substantial efforts have been devoted to asymmetric electrosynthesis. Impressive progress has been achieved by the strategic introduction of different chiral factors, including chiral electrode surface, chiral solvent, chiral electrolyte, chiral auxiliary, chiral mediator, and chiral catalyst^17–25^. Among these strategies, the use of a chiral catalyst for asymmetric amplification represents the most attractive approach^19–25^. However, it is not straightforward to implement asymmetric catalysis in an electrochemical context. Various compatibility issues need to be addressed in order to achieve both high efficiency and excellent asymmetric induction. Although a number of efficient electrocatalytic asymmetric transformations have been developed in the past decade, the modes of asymmetric induction are rather limited and there remain unsolved questions^19–25^.

**Fig. 1:**
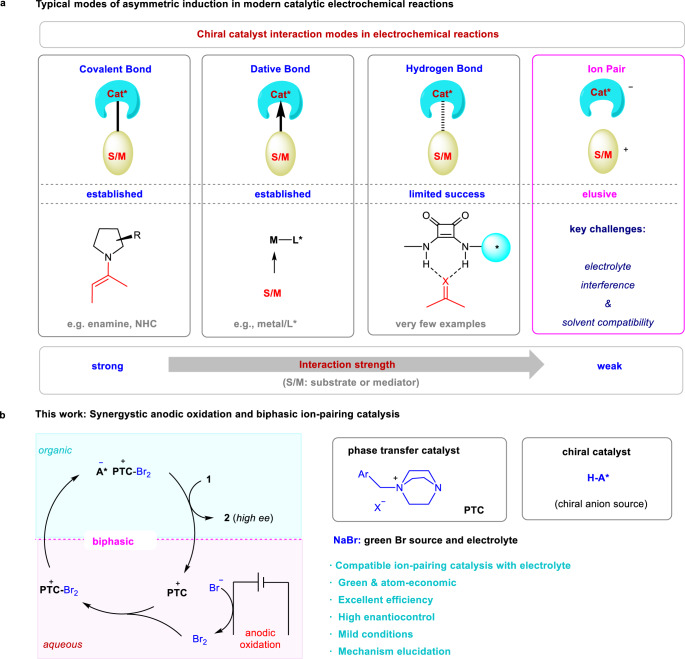
Introduction to asymmetric electricity-driven catalysis and our design strategy by phase-transfer ion-pairing catalysis. **a** Introduction to different strategies in asymmetric electrochemical catalysis. Covalent bond and dative bond with the chiral catalyst have been established, but weak bond interaction has been less successful. Ion-pairing catalysis remains elusive due to interference by the ionic electrolyte. Cat* chiral catalyst, S/M substrate or mediator. **b** The design principle of an electricity-driven asymmetric system using phase-transfer ion-pairing strategy. PTC phase-transfer catalyst, A* chiral phosphate anion.

Based on the interaction strength between the chiral catalyst and substrate (or reagent/mediator), modern catalytic asymmetric induction has primarily relied on four types of interactions, namely, covalent bond, dative bond (e.g., metal–ligand coordination), weak bond (e.g., hydrogen bond), and ion-pair interaction, in order of decreasing strength (Fig. 1a)^15,16^. However, their applicability in electrochemistry varied significantly^19–25^. Among them, covalent bonding with a chiral catalyst proved most successful^26–32^. For example, chiral amine catalysis via an enamine intermediate has been established in many electrochemical processes, in which the covalently linked substrate-catalyst adduct ensures robust asymmetric induction in the presence of many other interactions in a complex electrochemical system. Likewise, transition metal catalysis relying on the relatively strong coordination between a chiral organometallic catalyst and substrate (or mediator) has also enabled a number of highly enantioselective electrocatalytic processes^33–42^. However, weak interactions have met with limited success so far in electrocatalytic asymmetric systems^43–48^. For example, excellent enantioselectivity induced by hydrogen bonding interaction with a small chiral organocatalyst has been achieved only recently^46^. In fact, to the best of our knowledge, chiral ion-pairing interaction, which is weakest among the above four types of interactions, has found almost no success in inducing high enantioselectivity, in spite of its outstanding performance in non-electrochemical contexts^49–51^. The key challenge lies in the compatibility between these two systems. For example, the weak but essential chiral ion-pair interaction can be easily interrupted by the electrolyte, which is also ionic and must be used in a large amount to ensure conductivity. Thus, the key chiral ion-pair intermediate surrounded by massive achiral ions from the electrolyte would lead to low integrity for asymmetric induction. Moreover, asymmetric ion-pairing catalysis is best operated in nonpolar solvents to guarantee contact ion-pair formation, but electrochemical systems typically favor polar solvents.

To address the above challenge, we envisioned the use of a phase-transfer catalysis (PTC) strategy (Fig. 1b)^52^. If the key chiral ion-pair intermediate, once generated, can be spontaneously separated from the electrolyte and function in a different phase, high asymmetric induction could be expected purely from the chiral counter ion. However, the implementation of this concept for electrochemistry entails the choice of a suitable catalytic system. For example, the chiral ion pair prefers to enter the organic phase where asymmetric induction takes place, whereas the electrolyte stays in the aqueous phase to support the redox event. A suitable phase-transfer catalyst may also be needed to impeccably transport the relevant intermediates between phases.

Asymmetric halocyclization is an important family of transformations that have found broad applications in the synthesis natural products and biologically important molecules^53^. Traditional chemical oxidation approaches typically require the use of stoichiometric amounts of an electrophilic halogenation source, which typically result in a large amount of waste. For example, in 2013, Xie et al. achieved such an elegant process with stoichiometric phase-transfer-catalyst-bound bromination regent^54^. In contrast, anodic oxidation of aqueous NaBr could serve as a green electrophilic source (Fig. 1b)^55^. Thus, the **PTC-Br**_**2**_ cation generated in aqueous phase is expected to pair with a large chiral organic anion. Driven by its high lipophilicity, this chiral ion pair enters the organic phase and triggers asymmetric bromination without interference by the electrolyte. After that, the **PTC** re-enters the aqueous phase to transport Br_2_ for the next catalytic cycle. The whole process would use only a catalytic amount of **PTC** and chiral anion, together with a green bromide source.

Here, we describe two efficient asymmetric bromocyclization processes enabled by chiral phosphate anion phase-transfer catalysis and synergistic electrochemical oxidation of an inorganic bromide source.

## Results

### Reaction development

*tert*-Butyloxycarbonyl (Boc)-protected tryptamine **1a** was employed as the model substrate for the evaluation of reaction parameters in the electrocatalytic asymmetric bromocyclization. Various reaction parameters were systematically investigated, including chiral catalyst, solvent and so on (see Supplementary Information for details). These considerable efforts led to the choice of an operationally simple undivided cell equipped with platinum anode and cathode (Table 1). To simplify this system, NaBr was used as both the bromine source and the supporting electrolyte. The DABCO-derived ammonium salt **PTC1** was identified as the best phase-transfer catalyst. Notably, with electricity as the end oxidant, this carrier is only required in a catalytic amount, in sharp contrast to the use of stoichiometric amounts of electrophilic bromine sources in traditional chemical oxidation protocols. A range of chiral phosphoric acids (CPAs) were also compared for asymmetric induction (see Supplementary Tables 2 and 3), which identified a new structure, [H_8_]BINOL-derivative **CPA1** bearing two bulky substituents, as the superior chiral catalyst to provide excellent enantioselectivity (95% e.e.) and quantitative yield in a toluene–water biphasic system under a constant current of 2 mA at room temperature. Notably, almost all the components in this system were crucial for this outcome. Without **CPA1**, the reaction could proceed, but very slowly to the product in racemic form^56,57^, implying that the chiral phosphate was not only for asymmetric induction but also playing a phase-transfer role to pull the **PTC1-Br**_**2**_ cation to the organic phase by increasing its solubility, thus facilitating the process. Without **PTC1**, the reaction proceeded relatively slowly and, more importantly, with only 25% e.e., which was likely a result of the less efficient background reaction directly with Br_2_ as the bromination agent. Of particular note, NaHCO_3_ was essential to enhancing the reactivity. Without it or replacing it with other bases, such as Na_2_CO_3_ or NaOH, little or no conversion was observed. The origin of sensitivity to base and the critical role of NaHCO_3_ will be elaborated in mechanistic discussion (*vide infra*). Moreover, KBr could be used in place of NaBr, but NaCl or NaI did not lead to the corresponding halogenation product. We also evaluated other electrodes, including glassy carbon and nickel form, but they were less efficient. Polar organic solvents, such as EtOAc and DCM, led to dramatic decrease in enantioselectivity (see Supplementary Tables 5). The high solubility of the initially generated **PTC1-Br**_**2**_ cation (with Br^−^/Cl^−^ as counter anion) in these polar organic solvents allowed the background reaction to take place without involving the chiral phosphate anion. Finally, it is also worth noting that the stirring speed had a minor influence to enantioselectivity. Stirring at 1000 r/min resulted in slightly higher enantioselectivity than that at 660 r/min (from 93% e.e. to 95% e.e.). A higher speed may facilitate ion exchange and subsequent phase transfer of the newly generated **PTC1-Br**_**2**_ salt, which was initially deposited on the electrode surface due to poor solubility in the aqueous phase. To the best of our knowledge, the development of efficient electricity-driven asymmetric processes catalyzed by chiral phosphoric acids remains elusive^58,59^.

**Table 1:** Reaction condition optimization


Entry	Deviation from standard condition	Yield, e.e.
1	None	99%, 95% e.e.
2	Without (*R*)-**CPA1**	30%, −
3	Without **PTC1**	66%, 25% e.e.
4	Without NaHCO_3_	27%, 89% e.e.
5	Na_2_CO_3_ instead of NaHCO_3_	17%, −
6	KBr instead of NaBr	>95%, 93% e.e.
7	NaCl or NaI instead of NaBr	no reaction
8	Glassy carbon as anode	30%, 75% e.e.
9	Nickel foam as cathode	45%, 73% e.e.
10	EtOAc instead of toluene	90%, 20% e.e.

Detailed reaction parameters for the optimized conditions and the reaction outcomes upon changing the parameters. Reaction scale: **1a** (0.1 mmol), (*R*)-**CPA1** (5 mol %), **PTC1** (10 mol %), NaHCO_3_ (0.1 M in H_2_O), NaBr (1.0 M in H_2_O), toluene (2 mL), H_2_O (2.5 mL), Pt anode and cathode (10 mm × 10 mm × 0.2 mm), stirring speed: 1000 r/min, 2 mA, rt, 4 h. *Boc tert*-butyloxylcarbonyl. The yields were determined by crude ^1^H NMR analysis, and e.e. values were determined by chiral HPLC.

#### Substrate scope exploration

**Fig. 2:**
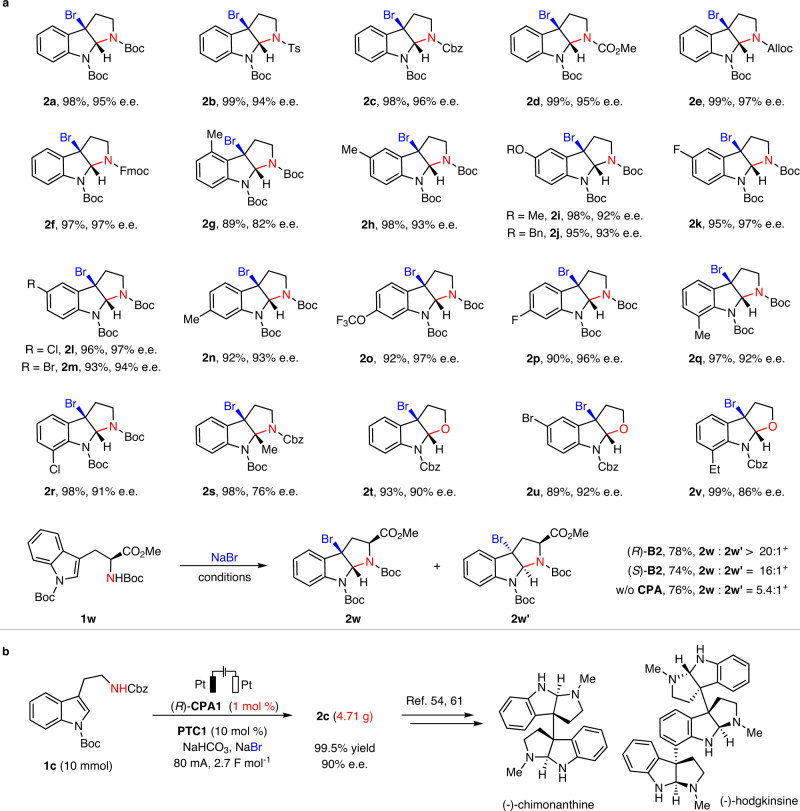
Evaluation of scope and application of the electrocatalytic asymmetric bromocyclization of tryptamine and tryptophol derivatives. **a** Reaction scope regarding the tryptamine and tryptophol derivatives. **1** (0.3 mmol), (*R*)-**CPA1** (5 mol %), **PTC1** (10 mol %), NaHCO_3_ (0.1 M in H_2_O), NaBr (1 M in H_2_O), toluene (6 mL), H_2_O (5 mL), Pt anode and cathode (10 mm × 10 mm × 0.2 mm), stirring speed: 1000 r/min, 4 mA (2.5–3.0 F mol^−1^), rt. Isolated yields were provided, and e.e. values were determined by chiral HPLC. *Ts p*-toluenesulfonyl, *Cbz* benzyloxycarbonyl, *Alloc* allyloxycarbonyl, *Fmoc* 9-fluorenylmethyl, *Bn* benzyl ^*+*^ Electric current at 3 mA for 18 h (6.7 F mol^−1^). **b** A gram-scale protocol for the synthesis of **2c** with reduced catalyst loading and high current and selected natural products that were synthesized from such bromination products.

With the optimized conditions, the substrate scope of this electricity-driven asymmetric bromocyclization was examined (Fig. 2a). First of all, different useful *N*-protective groups were employed for the tryptamine structure, and they were all compatible with the mild conditions, giving rise to excellent yields and enantioselectivities (**2a**-**f**) and permitting application in different contexts, such as late-stage modifications. Then, the substitution effect at different positions (C-4 to C-7 positions) of the indole ring was investigated. Regardless of the electron-donating or electron-withdrawing nature of the substituent, these substituted tryptamines all resulted in excellent outcomes (**2g**-**r**). Of note, methyl substitution at the C-2 position led to moderate enantioselectivity, albeit with excellent yield. However, this case provided vicinal tetrasubstituted stereogenic centers (**2s**), a structural motif challenging to assemble in asymmetric synthesis. Moreover, tryptophol derivatives, which bear an internal alcohol nucleophile, could also undergo bromocyclization with excellent efficiency and enantioselectivity (**2t**-**v**)^60^. It is worth noting that all these cases exhibited high Faraday efficiencies (66–80%), illustrating the design rationality of this system. A tryptophan derivative (*S*)-**1w**, with an existing chirality, was also examined. In the absence of the CPA catalyst but under otherwise almost identical conditions, the reaction afforded a diastereomeric mixture (**2w/2w′** = 5.4:1), suggesting the substrate-directing effect. Interestingly, in the presence of the CPA catalyst, regardless of its absolute configuration, the product diastereoselectivity was significantly enhanced, both favoring the same diastereomer **2w**, indicating that the absolute configuration was mainly controlled by the substrate’s existing chirality. The enhanced diastereoselectivity by the CPA catalyst is likely owing to the improved substrate facial sensitivity during C−Br bond formation when a bulky chiral phosphate anion (vs. the small Br^−^) is bound to the active bromination reagent.

Finally, this efficient protocol was further applied in a multigram synthesis of **2c** to demonstrate its robust practicability (Fig. 2b). With a reduced loading (1 mol %) of the CPA catalyst and a higher current (80 mA), the 10-mmol reaction of **1c** proceeded in quantitative yield with excellent enantioselectivity. Such enantioenriched 3-bromoindoline structures have been widely utilized as intermediates toward the syntheses of various cyclotryptamine alkaloids, including (–)-chimonanthine^54^ and (–)-hodgkinsine^61^.

### Strategy extension

**Fig. 3:**
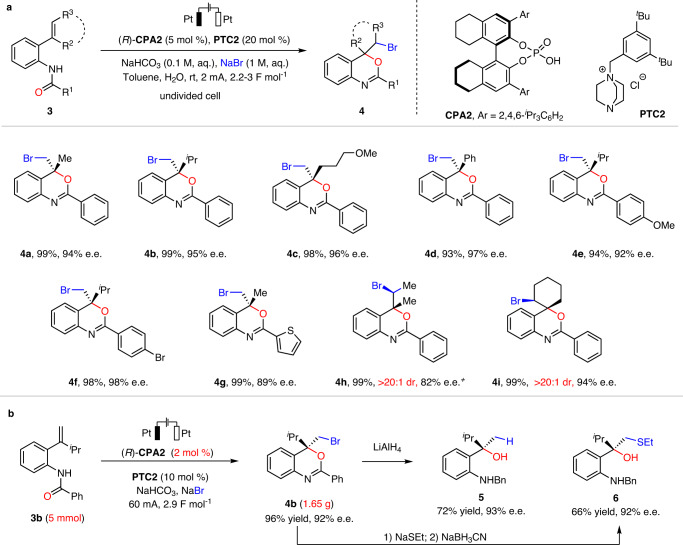
Reaction conditions, scope and application of electricity-driven asymmetric bromocyclization of 2-amidostyrenes. **a** Reaction scale: **3** (0.3 mmol), **(*R*)-CPA2** (5 mol %), **PTC2** (20 mol %), NaHCO_3_ (0.1 M in H_2_O), NaBr (1.0 M in H_2_O), toluene (6 mL), H_2_O (5 mL), Pt anode and cathode (10 mm × 10 mm × 0.2 mm), stirring speed: 1000 r/min, 4 mA (2.5–3.0 F mol^−1^), rt. Isolated yields were provided, and e.e. values were determined by chiral HPLC. ^*+*^10 mol % of **(*R*)-CPA2** was used. **b** Gram-scale protocol with 2 mol % of catalyst and high current for the synthesis of **4b** and further transformations.

The success of the above electrocatalytic asymmetric protocol enabled by chiral anion phase-transfer strategy prompted us to further study its applicability in other asymmetric halogenation processes. For example, intramolecular asymmetric bromocyclization of 2-amidostyrenes **3** represents another useful reaction providing expedient access to chiral *4H*-benzo[*d*][1,3]oxazines, representing potential chiral ligands and synthetic building blocks (Fig. 3a)^62^. Minor modifications from the above catalytic system and the conventional chemical oxidation conditions provided excellent reaction efficiency and enantioselectivity for this process. Notably, the use of **CPA2** as the chiral catalyst and **PTC2** as the phase-transfer catalyst were essential since those previously used catalysts for tryptamine cyclization or chemical oxidation were not able to deliver excellent results in this case (see Supplementary Table 8). Thus, a diverse set of highly enantioenriched chiral *4H*-benzo[*d*][1,3]oxazines bearing different substituents could be synthesized with high efficiency (essentially quantitative yields in all the cases). In addition to terminal olefins, internal and cyclic olefins also reacted with high enantioselectivity and diastereoselectivity (**4h** and **4i**). This robust protocol could also be scaled up for the gram-scale synthesis of **4b** with reduced catalyst loading but without obvious erosion in efficiency or stereoselectivity (Fig. 3b). We also carried out two derivatizations of the *4H*−3,1-benzoxazine product **4b**. Simple reduction by LiAlH_4_ readily furnished chiral tertiary alcohol **5**. Furthermore, thiolation of the bromide unit followed by reduction by NaBH_3_CN resulted in thioether **6**. Combined with the bromocyclization step, these two reactions can be considered as net asymmetric hydration and vicinal thiohydroxylation of olefins, which are still challenging to achieve directly.

### Mechanistic studies

**Fig. 4:**
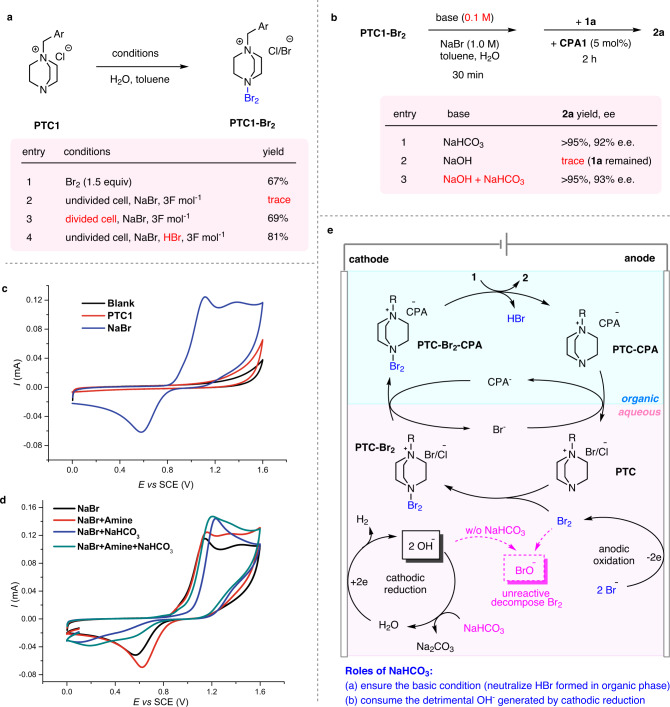
Mechanistic study and proposed mechanism. **a** Comparison of the **PTC1-Br**_**2**_ intermediate formation conditions. Ar = 3,5-bis(trifluoromethyl)phenyl. **b** Effect of NaHCO_3_ and NaOH in the bromination process using preformed **PTC1-Br**_**2**_ as a stoichiometric reagent. When NaOH was used, no conversion of **1a** was observed because it decomposed **PTC1-Br**_**2**_. In contrast, NaHCO_3_ prevented this decomposition and recovered the desired reactivity, which mimicked the standard condition. **c**, **d** Cyclic voltammetry measurements in H_2_O with 0.1 M NaNO_3_ and 5 mM substrate at room temperature with a scan rate of 100 mVs^−1^. **e** Detailed mechanism including the roles of NaHCO_3_.

We next performed a series of mechanistic studies. First of all, mixing **Br**_**2**_ and **PTC1** in water and toluene resulted in the immediate formation of the adduct **PTC1-Br**_**2**_ (Fig. 4a, entry 1). However, oxidation of NaBr by electricity in the presence of stoichiometric **PTC1** in an undivided cell resulted in trace **PTC1-Br**_**2**_ (Fig. 4a, entry 2), which appeared to contradict the proposed intermediacy of **PTC1-Br**_**2**_ in the standard protocol. However, this experiment may not reflect the whole picture of the standard reaction. We reasoned that the OH^–^ generated by water reduction on the cathode could capture Br_2_ generated by anodic oxidation by forming NaBrO, which explains the failure to observe **PTC1-Br**_**2**_. In contrast, the bromination event in the organic phase of the standard protocol indeed generates a half equivalent of HBr and contributes to neutralizing the base generated in the cathode. To support this rationale, we performed the same experiment in a divided cell, in which the OH^–^ generated on the cathode is physically isolated from the anode part. As expected, **PTC1-Br**_**2**_ could be successfully obtained in good yield (Fig. 4a, entry 3). Alternatively, the addition of HBr to an undivided cell could also recover the efficient formation of **PTC1-Br**_**2**_, which was in agreement with the above analysis (Fig. 4a, entry 4). These experiments implied that the OH^−^ generated on the cathode is a hidden factor that needs to be considered in such a system.

Next, we examined the chemical competence of **PTC1-Br**_**2**_ for asymmetric bromination. Mixing **PTC1-Br**_**2**_ with NaHCO_3_ in the same biphasic media (toluene and aqueous NaBr) followed by the addition of **1a** and **CPA1** (5 mol%) successfully produced product **2a** in >95% yield and 92% e.e., consistent with the standard electricity-driven protocol (Fig. 4b, entry 1). In contrast, if NaOH was used as a base, immediate decomposition of **PTC1-Br**_**2**_ was observed (Fig. 4b, entry 2), and further addition of **1a** and **CPA1** resulted in no bromination, which proved the detrimental effect of NaOH. Notably, in the standard protocol, NaOH is indeed generated at the cathode anyway and only half of it is neutralized by HBr (generated from bromination). Therefore, the standard protocol must involve others means to consume the excess NaOH, for which NaHCO_3_ might be responsible. Indeed, when NaHCO_3_ was used in combination with one equivalent of NaOH (relative to **1a**), which mimicked the system of the standard protocol, the decomposition of **PTC1-Br**_**2**_ was inhibited (or retarded) and the bromination reactivity was recovered (Fig. 4b, entry 3). It was believed that NaHCO_3_ quickly captured NaOH (by forming Na_2_CO_3_) like a buffer, which prevented the rapid decomposition of **PTC1-Br**_**2**_. This explained the critical role of NaHCO_3_ in the standard protocol.

Next, we performed cyclic voltammetry studies to disclose the redox behavior on the electrodes. A comparison of the oxidation potential of **PTC1** and NaBr revealed that Br^−^ is easier to oxidize (E_p_ = 1.12 V vs SCE), which ensured the stability of catalyst **PTC1** in the reaction (Fig. 4c). The addition of **PTC1** or NaHCO_3_ into the NaBr solution can stimulate a catalytic current of Br^−^ oxidation (Fig. 4d, red and blue, also see the Supplementary Information for more details). Further comparison indicated that **PTC1** can also stimulate the reduction current. This observation revealed that the reaction of **PTC1** with Br_2_ is reversible or the interaction between **PTC1** and Br_2_ is weak. In contrast, NaHCO_3_ diminishes the reduction current, which might be related to irreversible decomposition of Br_2_ by the base. However, when **PTC1** was added to the mixture of NaBr and NaHCO_3_, the reduction peak was observed again, suggesting that the formation of **PTC1-Br**_**2**_ and the base-induced decomposition of Br_2_ are competing with each other (Fig. 4d, green).

Based on the above experiments, a detailed reaction cycle is proposed (Fig. 4e). The reaction starts by anodic oxidation of Br^−^ to form Br_2_, which is captured by **PTC** to generate **PTC-Br**_**2**_. Subsequent anion exchange with the chiral phosphate anion (CPA^−^) forms a more organic-soluble ion pair **CPA-PTC-Br**_**2**_, which initiates asymmetric bromination in the organic phase. In addition to product formation, a molecule of HBr and ammonium salt **PTC-CPA** are formed. The acid is immediately neutralized by the basic medium, and the latter re-enters the aqueous phase, upon pairing with Br^−^ to regenerate **PTC**. Alternatively, the bromination step can also directly form **PTC** and a molecule of chiral phosphoric acid. Importantly, the successful operation of this catalytic cycle relies on the critical use of NaHCO_3_. Although the exact roles of NaHCO_3_ are not fully understood, we believe that it not only ensures a slightly basic medium for the phosphate anion to operate, but also efficiently inhibits (or retards) Br_2_ decomposition by consuming NaOH generated at the cathode.

We have developed a highly efficient electricity-driven catalytic asymmetric protocol employing weak ion-pairing interaction for asymmetric induction. In contrast to the broad success of asymmetric induction by relatively strong bonds asymmetric catalysis induced by weak interactions under electrochemical conditions has been notoriously challenging due to various compatibility issues. For ion-pairing catalysis, particularly problematic is the interference by electrolyte and polar solvents that are typical for electrochemical systems. In the context of this formidable challenge, we have designed an aqueous/organic phase-transfer strategy to permit useful asymmetric bromocyclization to take place with both excellent efficiency and high enantioselectivity. Compared with the conventional chemical oxidation approaches that require a stoichiometric amount of an electrophilic bromination reagent, our electricity-driven system employs only a small amount of a phase-transfer catalyst, together with NaBr as the green bromine source. Two representative asymmetric bromocyclization processes have been developed to showcase this powerful system. The practical utility of this system has also been illustrated by efficient gram-scale electrosynthesis at low catalyst loading. Mechanistic studies revealed that the catalytic cycle impeccably synergizes multiple events, including anodic oxidation, ion exchange, phase transfer, and asymmetric bromination. Of particular note is the critical roles of NaHCO_3_, which ensures a weakly basic condition and inhibits decomposition of the bromination reagent. This is also a rare demonstration of using chiral phosphoric acids as efficient chiral inducer in electrosynthesis. This system is expected to open a paradigm of electricity-driven asymmetric catalysis via weak ion-pairing interactions.

## Methods

### General procedure for the synthesis of 2 and 4

To an undivided vial (25 mL) equipped with the platinum anode (10 × 15 × 0.2 mm), platinum cathode (10 × 15 × 0.2 mm), and two magnetic stir bars (oval, 6 × 10 mm) were added substrate **1** or **3** (0.3 mmol), **CPA** (5 mol %), **PTC1** (10 mol %), NaHCO_3_ (42 mg, 0.5 mmol), NaBr (515 mg, 5.0 mmol), toluene (6 mL), and H_2_O (5 mL) (*note*: for substrate **1**, catalysts (*R*)-**CPA1** and **PTC1** were used; for substrate **3**, catalysts (*R*)-**CPA2** and **PTC2** were used). This vial was placed on a stir plate with a stirring speed of 1000 r/min. The electrolysis was carried out with a constant current of 4 mA for 4.5–6 h (2.2–3.0 F mol^−1^). The reaction progress was monitored by thin-layer chromatography. Upon completion, the organic layer of the reaction mixture was separated and the aqueous layer was extracted with EtOAc (5 mL × 2). The combined organic layers were dried over anhydrous Na_2_SO_4_, filtered, and concentrated. The residue was purified by silica gel chromatography to yield the desired product **2** or **4**.

## Supplementary information

Supplementary Information

## Data Availability

Source data are provided with this paper. The authors declare that all data supporting the findings of this study are available within the article and Supplementary Information files.
