# Supplementary material for: Electricity-driven asymmetric bromocyclization enabled by chiral phosphate anion phase-transfer catalysis
Source: Nat Commun. 2023 Jan 23;14:357. doi: 10.1038/s41467-023-36000-6 (PMC9870882; doi:10.1038/s41467-023-36000-6)
Supplement: Supplementary file 1 — Supplementary Information [file 41467_2023_36000_MOESM1_ESM.pdf]

Supplementary Information

Electricity-Driven Asymmetric Bromocyclization Enabled by Chiral Phosphate Anion Phase-Transfer Catalysis

Xuefeng Tan[1,2]*, Qingli Wang[1,3], and Jianwei Sun[1,2]*

[1]*Department of Chemistry, Energy Institute, Institute for Advanced Study, and the Hong Kong Branch of Chinese National Engineering Research Centre for Tissue Restoration & Reconstruction, The Hong Kong University of Science and Technology, Clear Water Bay, Kowloon, Hong Kong SAR, China*

[2]*HKUST Shenzhen Research Institute, No. 9 Yuexing 1st Rd, Shenzhen 518057, China*

[3]*Shenzhen Bay Laboratory, Shenzhen 518107, China*

*e-mail: xuefengtan@ust.hk; sunjw@ust.hk.

Table of Contents

1. Supplementary Methods S-3

1.1 General Information S-3

1.2 Electrochemical Setup S-4

2. Supplementary Discussion S-5

2.1 Reaction Condition Optimization S-5

2.2 Synthesis of Catalysts S-14

2.3 Synthesis of Substrates S-17

2.4 Asymmetric Electrochemical Indole Bromocyclization S-25

2.5 Asymmetric Electrochemical 2-Amidostyrene Bromocyclization S-41

2.6 Gram-Scale Reaction and Product Derivatizations S-48

3. Supplementary Notes S-52

3.1 Mechanistic Studies S-52

3.2 Determination of the Product Stereochemistry S-58

4. Supplementary References S-59

NMR Spectra and HPLC Traces

1. Supplementary Methods

1.1 General Information

Flash column chromatography was performed over silica gel (200-300 mesh) purchased from Qindao Puke Co., China. All air or moisture sensitive reactions were conducted in oven-dried glassware under nitrogen atmosphere using anhydrous solvents. Anhydrous dichloromethane, diethyl ether, and tetrahydrofuran were purified by the Innovative® solvent purification system or distilled under common conditions. The ACS grade toluene was purchased from Scharlab and used as received. Electrocatalysis was conducted using BioLogic VMP-3 Multichannel Potentiostat in constant current (CP) mode. Cyclic voltammetry studies were also performed using BioLogic VMP-3 Multichannel Potentiostat under the CV mode. ^{1}H, ^{13}C, ^{19}F and ^{31}P NMR spectra were collected on a Bruker AV 400 MHz NMR spectrometer using residue solvent peaks as an internal standard (^{1}H NMR: $CDCl_3$ at 7.26 ppm; ^{13}C NMR: $CDCl_3$ at 77.0 ppm). Mass spectra were collected on a MALDI Micro MX mass spectrometer, or an API QSTAR XL System. Optical rotations were measured on RUDOLPH A28576-T-LED API automatic polarimeter with $[\alpha]^{D}$ values reported in degrees; concentration (*c*) is in 10 mg/mL. The enantiomeric excess values were determined by chiral HPLC using an Agilent 1200 LC instrument with a Daicel CHIRALCEL OD-H or OJ-H column, or a Daicel CHIRALPAK AD-H, AS-H or IC column.

1.2 Electrochemical Setup

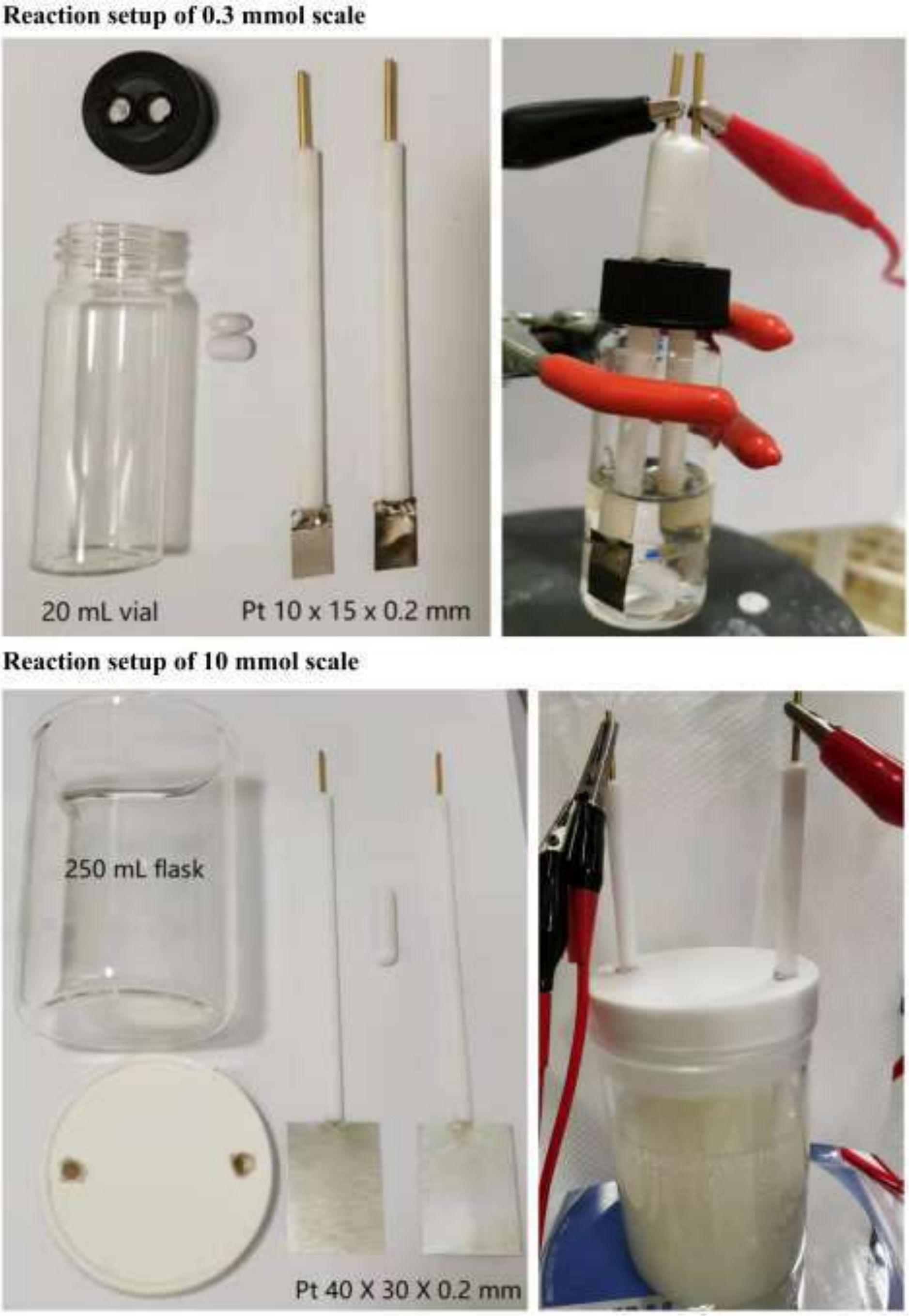

Supplementary Figure 1. Electrochemical setups for small and scale-up reactions.

2. Supplementary Discussion

2.1 Reaction Condition Optimization

Supplementary Table 1. Preliminary studies of the asymmetric electrochemical bromocyclization[a]

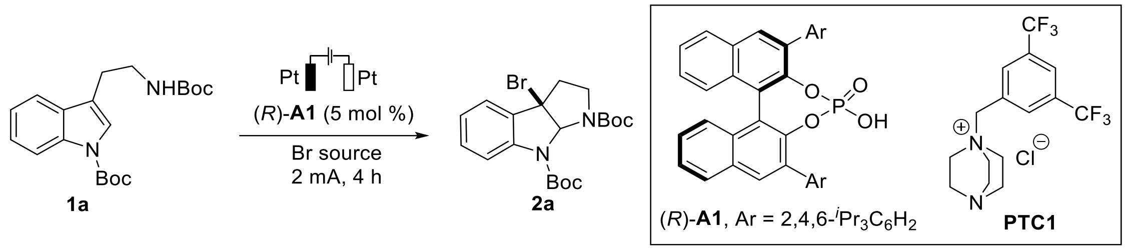

Entry	Solvent	Br source	$NaHCO_3$	Additive	Yield (%)[b]	E.e. (%)[c]
1	DCM/H_2O	KBr	4 equiv	–	75	7
2[d]	DCM/H_2O	KBr	4 equiv	–	76	0
3	DCM/H_2O	NaBr	4 equiv	–	73	8
4	DCM/H_2O	LiBr	4 equiv	–	75	7
5	DCM/H_2O	KBr	–	DABCO	ND	–
6	DCM/H_2O	KBr	–	Et_3N	ND	–
7	DCM/H_2O	KBr	–	pyridine	72	6
9	DCM/H_2O	NaBr	–	**PTC1**	70	24
10	DCM/H_2O	NaBr	4 equiv	**PTC 1**	93	22
11	toluene/H_2O	NaBr	–	**PTC 1**	20	78
12	toluene/H_2O	NaBr	4 equiv	**PTC 1**	91	86

[a]Reaction scale: **1a** (0.1 mmol), (*R*)-**A1** (5 mol %), Br source (1 M in H_2O), additive (4 equiv) except for **PTC1** (1 equiv), solvent using DCM/H_2O or toluene/H_2O (2 mL: 2.5 mL), Pt anode (15 mm × 10 mm × 0.2 mm), Pt cathode (10 mm × 10 mm × 0.2 mm), stirred at 660 r/min, 20-25 °C, 4 mA, 2 h. [b]Determined by crude ^1H NMR. [c]Determined by HPLC. [d]Run without a CPA catalyst.

Supplementary Table 2. Evaluation of the CPA catalysts[a]

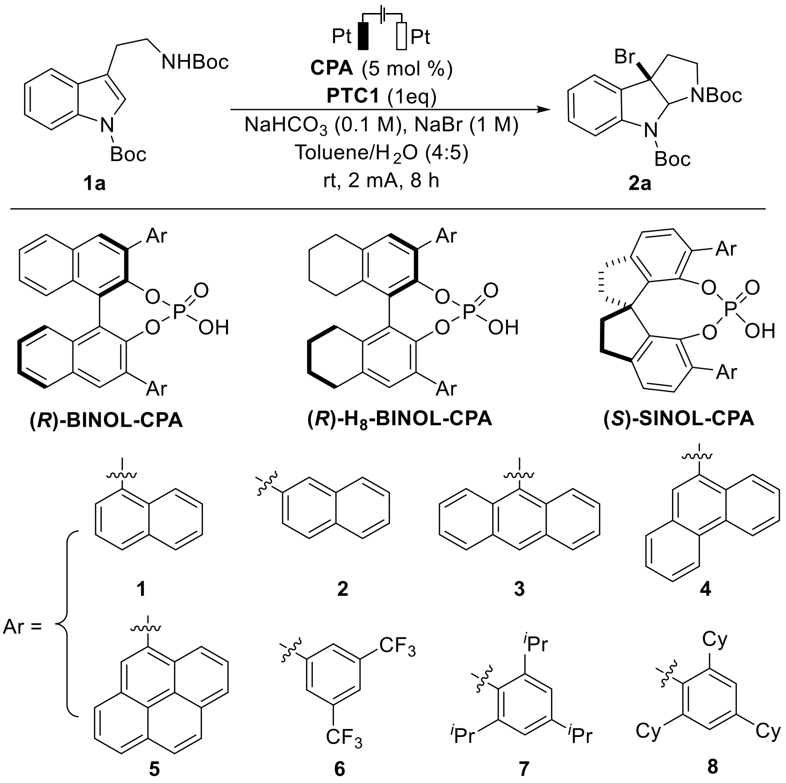

(*R*)-BINOL-CPA			(*R*)- [H8]BINOL-CPA			(*S*)-SPINOL-CPA		
Ar	Yield (%)	E.e. (%)	Ar	Yield (%)	E.e. (%)	Ar	Yield (%)	E.e. (%)
**1**	46	58	**1**	96	57	**1**	81	68
**2**	87	46	**2**	75	55	**2**	80	60
**3**	67	79	**3**	71	79	**3**	60	73
**4**	70	56	**4**	95	77	**4**	79	75
**5**	55	79	**5**	41	79	**5**	56	-69
**6**	38	73	**6**	46	25	**6**	35	8
**7**	96	86	**7**	87	88	**7**	66	-56
**8**	63	55	**8**	61	74	**8**	46	8

[a]Reaction scale: **1a** (0.1 mmol), **CPA** (5 mol %), NaBr (1 M in H_2O), $NaHCO_3$ (0.1 M in H_2O), **PTC1** (1 equiv), toluene/H_2O (2 : 2.5 mL), Pt anode (10 mm × 10 mm × 0.2 mm), Pt cathode (15 mm × 10 mm × 0.2 mm), stirred at 660 r/min, 20-25 °C, 2 mA, 8 h, yields were determined by crude ^1H NMR and e.e. values were determined by HPLC.

Supplementary Table 3. Further modification of the CPA catalysts[a]

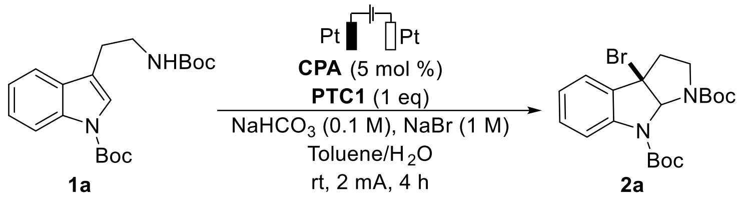

1a → **2a**

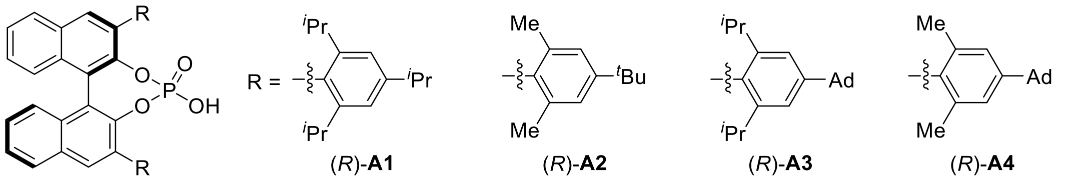

A

(*R*)-**A1**	(*R*)-**A2**	(*R*)-**A3**	(*R*)-**A4**
96% yield, 86% e.e.	71% yield, 87% e.e.	82% yield, 90% e.e.	96% yield, 91% e.e.

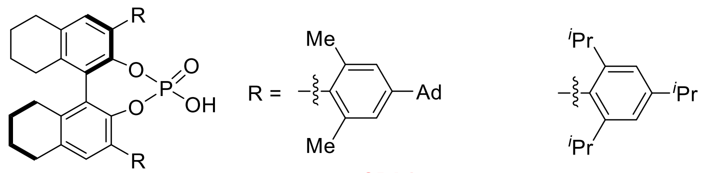

B

(*R*)-**CPA1**	(*R*)-**CPA2**
96% yield, 93% e.e.	87% yield, 88% e.e.

[a]Reaction scale: **1a** (0.1 mmol), **CPA** (5 mol %), **PTC 1** (1 equiv), $NaHCO_3$ (0.1 M in H_2O), NaBr (1 M in H_2O), toluene/H_2O (2 : 2.5 mL), Pt anode (10 mm × 10 mm × 0.2 mm), Pt cathode (15 mm × 10 mm × 0.2 mm), stirred at 660 r/min, 20-25 °C, 2 mA, 4 h, yields were determined by crude ^{1}H NMR and e.e. values were determined by HPLC.

Supplementary Table 4. Effect of the PTC loading[a]

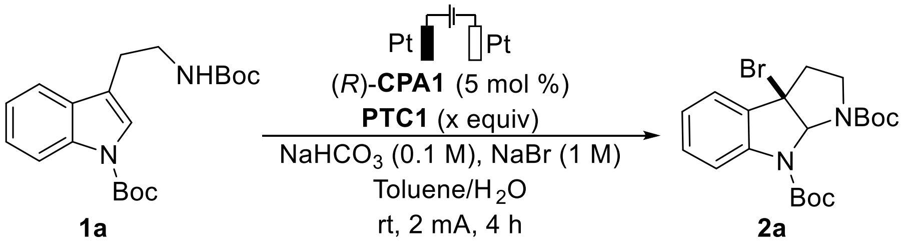

Entry	**PTC1** (x equiv)	Yield (%)	E.e. (%)
1	1	95	93
2	0.5	97	93
3	0.2	97	93
4	0.1	98	93
5	0.05	96	93
6	0	66	25

[a]Reaction scale: **1a** (0.1 mmol), (*R*)-**CPA1** (5 mol %), **PTC1** (0-1 equiv), $NaHCO_3$ (0.1 M in H_2O), NaBr (1 M in H_2O), toluene/H_2O (2 : 2.5 mL), Pt anode (10 mm × 10 mm × 0.2 mm), Pt cathode (15 mm × 10 mm × 0.2 mm), stirring at 660 r/min, 20-25 °C, 2 mA, 4 h, yields were determined by crude ^{1}H NMR and e.e. values were determined by HPLC.

Influence of the stirring speed

During the course of reaction condition optimization, we found the e.e. values were not always reproducible, with a fluctuation range of 1-5%. With various experimentation, we discovered that the stirring speed has slight influence on the result. The higher the stirring speed, the better results (especially ee values) were obtained. This phenomenon is common in phase-transfer catalytic systems, because the stirring speed could influence the mixing of different phases. Finally, we applied a stirring speed of 1000 r/min, instead of the previous speed of 660 r/min, which led to an improved e.e. value of 95%.

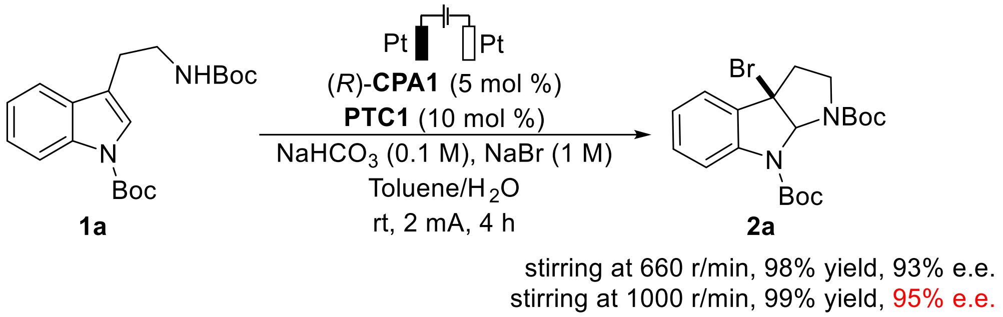

Supplementary Table 5. Optimization of solvent[a]

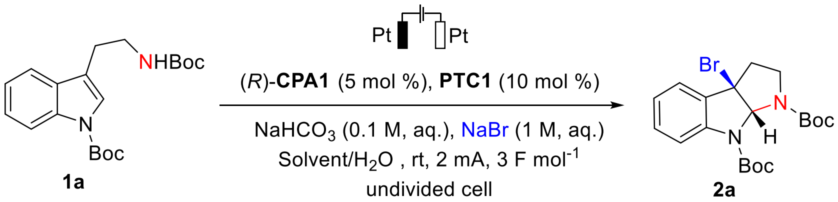

Entry	Solvent	Yield (%)	E.e. (%)
1	hexane	73	14
2	Et_2O	37	84
3	xylene	>95	92
4	DCM	>95	54
5	EtOAc	90	20

[a]Reaction scale: **1a** (0.1 mmol), (*R*)-**CPA1**(5 mol %), **PTC1** (10 mol %), $NaHCO_3$ (0.1 M in H_2O), NaBr (1 M in H_2O), solvent/H_2O (2 : 2.5 mL), Pt anode (10 mm × 10 mm × 0.2 mm), Pt cathode (15 mm × 10 mm × 0.2 mm), stirred at 1000 r/min, 20-25 °C, 2 mA, 4 h, yields were determined by crude ^1H NMR and e.e. values were determined by HPLC.

Supplementary Table 6. Screen of the phase-transfer catalysts[a]

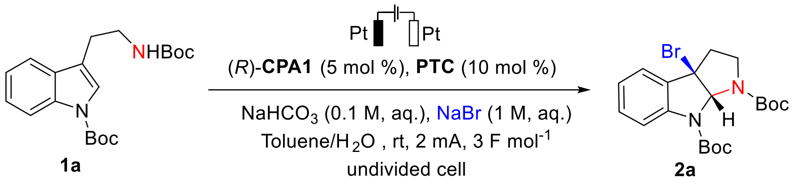

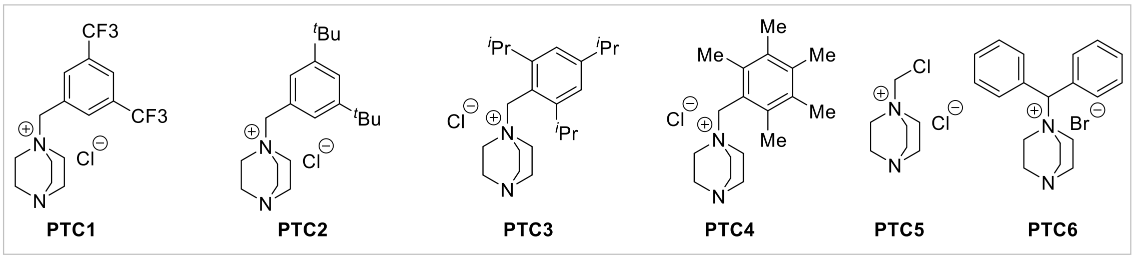

Entry	PTC	Yield (%)	E.e. (%)
1	**PTC1**	>95	95
2	**PTC2**	>95	88
3	**PTC3**	83	80
4	**PTC4**	73	85
5	**PTC5**	95	81
6	**PTC6**	94	75

[a]Reaction scale: **1a** (0.1 mmol), (*R*)-**CPA1**(5 mol %), **PTC** (10 mol %), $NaHCO_3$ (0.1 M in H_2O), NaBr (1 M in H_2O), toluene/H_2O (2 : 2.5 mL), Pt anode (10 mm × 10 mm × 0.2 mm), Pt cathode (15 mm × 10 mm × 0.2 mm), stirred at 1000 r/min, 20-25 °C, 2 mA, 4 h, yields were determined by crude ^1H NMR and e.e. values were determined by HPLC.

Supplementary Table 7. Comprehensive understanding of different reaction parameters based on the standard reaction[a]

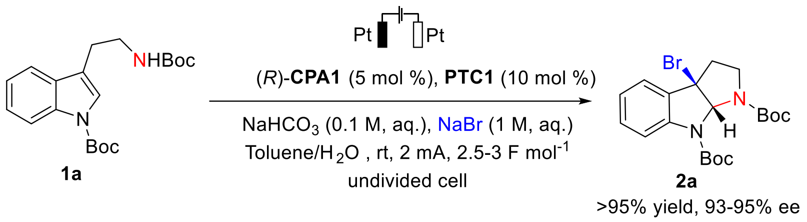

Deviation from the standard conditions	Yield, E.e.
Without (*R*)-**CPA1**	30%, --
Without **PTC1**	66%, 25%
Without $NaHCO_3$	27%, 89%
KBr or LiBr instead of NaBr	>95%, 93% / >95%, 92%
NaCl or NaI instead of NaBr	No reaction
Glassy carbon as anode	30%, 75%
Nickel foam as cathode	45%, 73%
DCM / EtOAc instead of toluene	>95%, 54% / 90%, 20%
1 mA or 4 mA	95%, 93% / 91%, 90%
0 ˚C	95%, 93%

[a]Reaction scale: **1a** (0.1 mmol), (*R*)-**CPA1** (5 mol %), **PTC1** (10 mol %), $NaHCO_3$ (0.1 M in H_2O), NaBr (1 M in H_2O), toluene/H_2O (2 mL : 2.5 mL), Pt anode (10 mm × 10 mm × 0.2 mm), Pt cathode (10 mm × 10 mm × 0.2 mm), stirred at 1000 r/min, 20-25 °C, 2 mA, 4 h, yields were determined by crude ^1H NMR and e.e. values were determined by HPLC.

Supplementary Table 8. Condition optimization for the reaction of 3b[a]

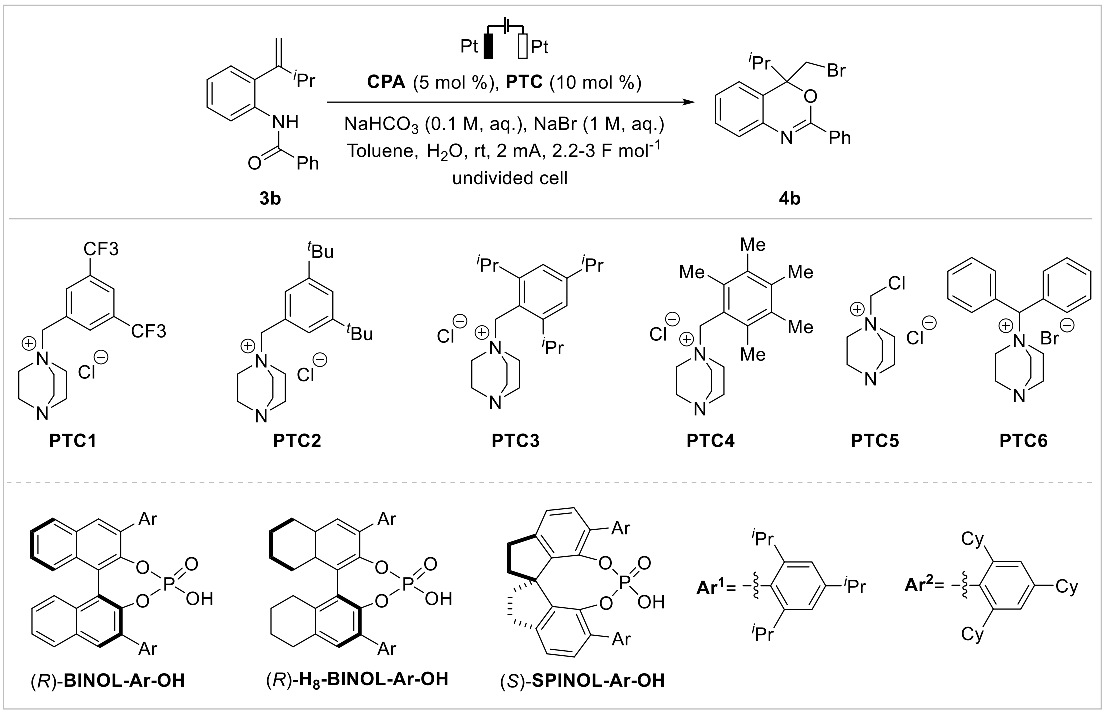

Entry	CPA	PTC	Solvent	Yield (%)	E.e (%)
1	**CPA1**	**PTC1**	toluene	>95	62
2	**CPA1**	**PTC2**	toluene	>95	73
3	**CPA1**	**PTC3**	toluene	>95	37
4	**CPA1**	**PTC4**	toluene	>95	29
5	**CPA1**	**PTC5**	toluene	>95	30
6	**CPA1**	**PTC6**	toluene	>95	65
7	**CPA1**	**PTC2**	*p*-xylene	68	65
8	**CPA1**	**PTC2**	mesitylene	24	64
9	**CPA1**	**PTC2**	$PhCF_3$	>95	44

10	**CPA1**	**PTC2**	hexane	18	72
11	(*R*)-**BINOL-Ar¹-OH**	**PTC2**	toluene	>95	90
12	(*R*)-**BINOL-Ar²-OH**	**PTC2**	toluene	>95	88
13	(*R*)-**H_8-BINOL- Ar¹-OH (CPA2)**	**PTC2**	toluene	>95	93
14[b]	(*R*)-**H_8-BINOL- Ar¹-OH (CPA2)**	**PTC2**	toluene	>95	95
14	(*R*)-**H_8-BINOL- Ar²-OH**	**PTC2**	toluene	>95	90
15	(*S*)-**SPINOL-Ar¹-OH**	**PTC2**	toluene	>95	88
16	(*S*)-**SPINOL-Ar²-OH**	**PTC2**	toluene	>95	32

[a]Reaction scale: **3a** (0.1 mmol), **CPA** (5 mol %), **PTC** (10 mol %), $NaHCO_3$ (0.1 M in H_2O), NaBr (1 M in H_2O), toluene/H_2O (2 mL : 2.5 mL), Pt anode (10 mm × 10 mm × 0.2 mm), Pt cathode (10 mm × 10 mm × 0.2 mm), stirred at 1000 r/min, 20-25 °C, 2 mA, 4 h, yields were determined by crude ^1H NMR and e.e. values were determined by HPLC. [b]Run with 20 mol % of **PTC2**.

2.2 Synthesis of Catalysts

Synthesis of the CPA catalyst (*R*)-CPA1

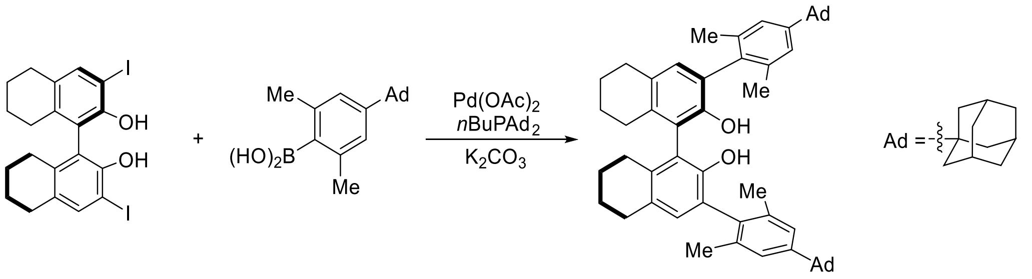

(*R*)-**S1** (*R*)-**S2**

(*R*)-3,3'-Bis(4-((3s)-adamantan-1-yl)-2,6-dimethylphenyl)-5,5',6,6',7,7',8,8'-octahydro-[1,1'-binaphthalene]-2,2'-diol ((*R*)-S2). Under N_2, to a Schlenk flask charged with (*R*)-**S1** (1.09 g, 2 mmol), (4-((3s)-adamantan-1-yl)-2,6-dimethylphenyl)boronic acid (3.41 g, 12 mmol), $Pd(OAc)_2$ (22.4 mg, 0.1 mmol), ${}^{n}BuPAd_2$ (71.8 mg, 0.2 mmol) and K_2CO_3 (2.2 g, 16 mmol) were added 1 2-dimethoxyethane (30 mL) and H_2O (10 mL). The mixture was degassed by the freeze-pump-thaw method to remove the dissolved oxygen in solution. Then the mixture was heated to 95 °C and stirred for 12 h. After cooling to room temperature, the reaction mixture was poured into H_2O (100 mL) and extracted with EtOAc (50 mL × 3). The combined organic phases were washed with brine, dried over anhydrous Na_2SO_4, filtered, and concentrated *in vacuo*. The residue was purified by silica gel column chromatography (eluent: hexanes/DCM = 10:1 →3:1) to afford (*R*)-**S2** as a light yellowish-brown solid (1.46 g, 95% yield).

$[\alpha]_D^{25}$: -48.9 (c = 1.0, $CHCl_3$).

^{1}H NMR (400 MHz, $CDCl_3$) δ 7.17 – 7.04 (m, 4H), 6.84 (s, 2H), 4.49 (s, 2H), 2.83 – 2.67 (m, 4H), 2.44 – 2.25 (m, 4H), 2.18 – 2.01 (m, 18H), 1.95 (s, 12H), 1.86 – 1.66 (m, 20H) ppm.

^{13}C NMR (100 MHz, $CDCl_3$) δ 150.5, 147.9, 136.7, 136.6, 135.9, 133.7, 131.1, 129.6, 124.7, 124.2, 124.0, 120.5, 43.3, 37.0, 35.9, 29.3, 29.1, 27.1, 23.3, 23.2, 21.01, 20.95. ppm.

HRMS (CI-) Calcd for $C_{56}H_{65}O_2$ $[M-H]^-$: 769.4990, found: 769.4998.

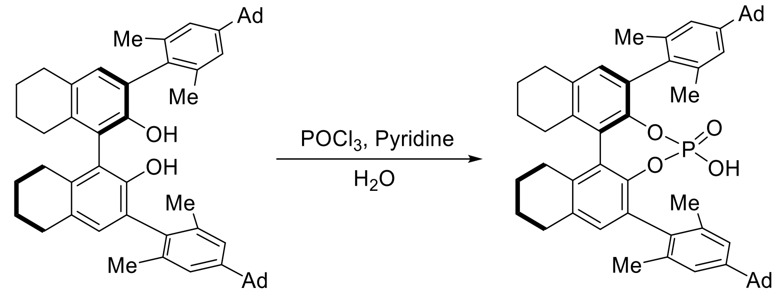

(*R*)-**S2** (*R*)-**CPA1**

(*R*)-2-(4-((3*S*)-Adamantan-1-yl)-2,6-dimethylphenyl)-6-(4-((3*S*)-adamantan-1-yl)-2,6-dimethylphenyl)-4-hydroxy-8,9,10,11,12,13,14,15-octahydrodinaphtho [2,1-d:1',2'-f][1,3,2]dioxaphosphepine 4-oxide ((*R*)-CPA1). At 0 °C under N_2, to a solution of (*R*)-**S2** (1.46 g, 1.9 mmol) in pyridine (20 mL) was added $POCl_3$ (2 mL) dropwise. The mixture was heated to 80 °C and stirred at the same temperature for 5 h. Then, the mixture was cooled to 0 °C followed by slow addition of water (4 mL). Next, the mixture was heated to 70 °C and stirred at the same temperature for 3 h before it was cooled to room temperature and diluted with DCM (50 mL). The mixture was washed with an aqueous HCl solution (6.0 M, 60 mL). The layers were separated, and the aqueous layer was extracted with DCM (50 mL × 3). The combined organic layers were dried over Na_2SO_4 and concentrated. The residue was purified by silica gel column chromatography (eluent: DCM→DCM/EtOAc = 5:1). The obtained product was re-dissolved in DCM (40 mL) and treated with aqueous hydrochloric acid (6.0 M, 30 mL). The mixture was stirred vigorously for 10 min. The organic layer was separated, dried over Na_2SO_4, and evaporated to give the desired chiral phosphoric acid (*R*)-**CPA1** as a white solid (1.51 g, 95% yield).

$[\alpha]_D^{25}$: -73.8 (c = 1.0, $CHCl_3$).

^{1}H NMR (400 MHz, $CDCl_3$) δ 8.43 (s, 1H), 6.93 (s, 4H), 6.85 (s, 2H), 2.92 – 2.63 (m, 6H), 2.35 – 2.18 (m, 2H), 2.14 (s, 6H), 2.05 – 1.91 (m, 12H), 1.91 – 1.76 (m, 18H), 1.76 – 1.58 (m, 14H) ppm.

^{13}C NMR (100 MHz, $CDCl_3$) δ 149.9, 143.6, 136.2, 136.0, 135.9, 134.8, 133.3, 131.9,

130.5, 126.7, 124.2, 122.9, 42.9, 37.0, 35.7, 29.2, 29.0, 27.7, 22.8, 22.7, 21.5, 20.8 ppm.

^{31}P NMR (162 MHz, $CDCl_3$) δ 3.29 ppm.

HRMS (CI-) Calcd for $C_{56}H_{64}O_4P$ $[M-H]^-$: 831.4548, found: 831.4538.

Synthesis of the PTC catalysts

PTC1 has been reported in the literature and **PTC2** was prepared according to the literature procedure.[1]

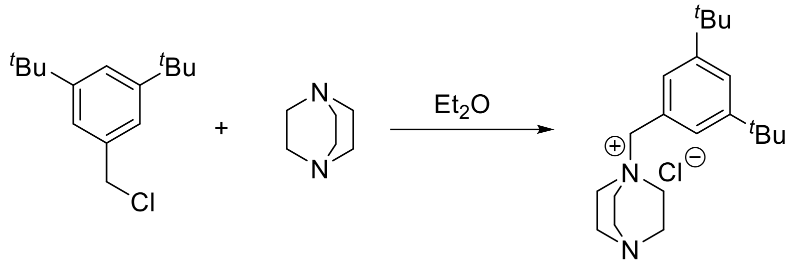

PTC2

1-(3,5-Di-*tert*-butylbenzyl)-1,4-diazabicyclo[2.2.2]octan-1-ium chloride (PTC2). A solution of 1,3-di-*tert*-butyl-5-(chloromethyl)benzene (1.19 g, 5.0 mmol) and 1,4-diazabicyclo[2.2.2]octane (0.56 g, 5.0 mmol) in diethyl ether (20 mL) was heated at 40 °C for 16 h. The resultant white solid was isolated by filtration and washed repeatedly with diethyl ether (1.26 g, 72% yield).

^{1}H NMR (400 MHz, DMSO) δ 7.52 (s, 1H), 7.37 (s, 2H), 4.58 (s, 2H), 3.34 (t, *J* = 7.1 Hz, 6H), 3.03 (t, *J* = 7.3 Hz, 6H), 1.31 (s, 18H) ppm.

^{13}C NMR (100 MHz, DMSO) δ 151.4, 127.9, 127.0, 124.1, 67.4, 52.0, 45.2, 35.0, 31.6 ppm.

HRMS (CI+) Calcd for $C_{21}H_{35}N_2$ $[M-Cl]^-$: 315.2795, found: 315.2796.

2.3 Synthesis of Substrates

Substrates **1a-b**,[2] **1c**,[3] **1d**,[2] **1f**,[3] **1g**,[2] **1h**,[3] **1i**,[2] **1k-m**,[3] **1p-r**,[2] **1t**,[4] **1w**,[4] **3a-b**,[1] **3d**[1] and **3g-i**[1] are known compounds and were prepared according to the literature procedures. The other substrates were prepared and characterized as shown below.

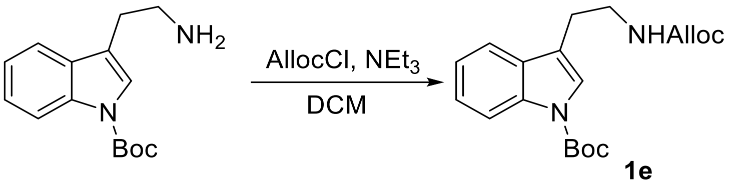

1e

***tert*-Butyl 3-(2-(((allyloxy)carbonyl)amino)ethyl)-1*H*-indole-1-carboxylate (1e).** *tert*-Butyl 3-(2-aminoethyl)-1*H*-indole-1-carboxylate (0.78 g, 3 mmol) and triethylamine (0.83 mL, 6 mmol) were dissolved in DCM (15 mL). At 0 °C, AllocCl (380 mg, 3.15 mmol) was added dropwise. The reaction was warmed to room temperature and stirred for additional 2 h. The reaction mixture was diluted with DCM (30 mL), and washed sequentially with an aqueous HCl solution (20 mL, 1 M), a saturated aqueous $NaHCO_3$ solution and brine. The organic phase was dried and concentrated, and the residue was subjected to silica gel flash chromatography directly to afford **1e** as a colorless viscous oil (950 mg, 92% yield).

^{1}H NMR (400 MHz, $CDCl_3$) δ 8.13 (d, *J* = 5.9 Hz, 1H), 7.53 (d, *J* = 7.7 Hz, 1H), 7.42 (s, 1H), 7.36 – 7.27 (m, 1H), 7.27 – 7.19 (m, 1H), 5.90 (ddt, *J* = 16.2, 10.9, 5.6 Hz, 1H), 5.28 (d, *J* = 17.2 Hz, 1H), 5.19 (d, *J* = 10.4 Hz, 1H), 5.02 (s, 1H), 4.56 (d, *J* = 5.4 Hz, 2H), 3.51 (dd, *J* = 13.1, 6.6 Hz, 2H), 2.90 (t, *J* = 6.9 Hz, 2H), 1.66 (s, 9H) ppm.

^{13}C NMR (100 MHz, $CDCl_3$) δ 156.3, 149.7, 135.6, 133.0, 130.4, 124.5, 123.2, 122.6, 118.9, 117.6, 117.6, 115.4, 83.6, 65.5, 40.6, 28.2, 25.6 ppm.

HRMS (CI+) Calcd for $C_{19}H_{24}N_2NaO_4$ $[M+Na]^+$: 367.1628, found: 367.1631.

Preparation of 1j, 1n and 1o.

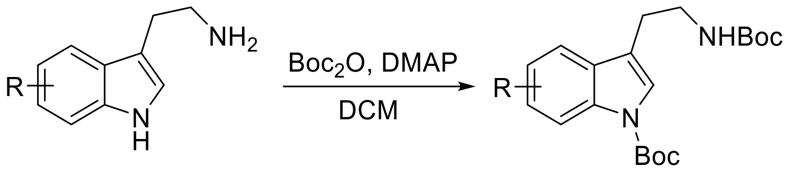

S3 **1j, 1n** and **1o**

At 0 °C, to a solution of the commercially available tryptamine derivative **S3** (3 mmol) and DMAP (36.6 mg, 0.3 mmol) in DCM (15 mL) was added Boc_2O (1.31 g, 6.0 mmol) dropwise. The reaction was warmed to room temperature and stirred for 12 h. The solvent was removed under reduced pressure, and the residue was subjected to silica gel flash chromatography to afford the corresponding product (**1j**, **1n** and **1o**).

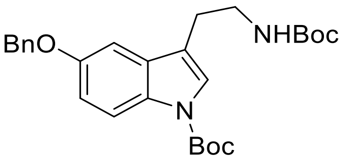

1j

***tert*-Butyl 5-(benzyloxy)-3-(2-((*tert*-butoxycarbonyl)amino)ethyl)-1*H*-indole-1-carboxylate (1j)** was prepared as a viscous oil (0.91 g, 65% yield).

^{1}H NMR (400 MHz, $CDCl_3$) δ 7.95 (s, 1H), 7.49 – 7.42 (m, 2H), 7.42 – 7.33 (m, 3H), 7.30 (t, *J* = 7.2 Hz, 1H), 7.08 (s, 1H), 7.02 – 6.96 (m, 1H), 5.09 (s, 2H), 4.74 (s, 1H), 3.42 (d, *J* = 6.1 Hz, 2H), 2.83 (t, *J* = 6.5 Hz, 2H), 1.64 (s, 9H), 1.43 (s, 9H) ppm.

^{13}C NMR (100 MHz, $CDCl_3$) δ 156.0, 155.0, 149.7, 137.3, 131.3, 130.5, 128.6, 127.9, 127.6, 123.9, 117.7, 116.1, 113.7, 103.5, 83.4, 79.2, 70.7, 40.1, 28.5, 28.3, 25.7 ppm.

HRMS (CI+) Calcd for $C_{27}H_{34}N_2NaO_5$ $[M+Na]^+$: 489.2360, found: 489.2367.

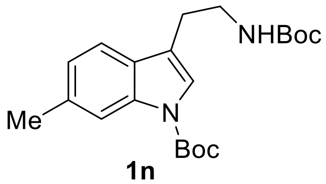

1n

***tert*-Butyl 3-(2-((*tert*-butoxycarbonyl)amino)ethyl)-6-methyl-1*H*-indole-1-**

carboxylate (1n) was prepared as a viscous oil (0.85 g, 76% yield).

^{1}H NMR (400 MHz, $CDCl_3$) δ 8.00 (s, 1H), 7.40 (d, *J* = 8.0 Hz, 1H), 7.32 (s, 1H), 7.06 (d, *J* = 8.0 Hz, 1H), 4.69 (s, 1H), 3.56 – 3.29 (m, 2H), 2.86 (t, *J* = 6.8 Hz, 2H), 2.48 (s, 3H), 1.66 (s, 9H), 1.44 (s, 9H) ppm.

^{13}C NMR (100 MHz, $CDCl_3$) δ 155.9, 149.8, 136.1, 134.5, 128.2, 123.9, 122.5, 118.6, 117.7, 115.6, 83.3, 79.2, 40.2, 28.4, 28.2, 25.7, 22.0 ppm.

HRMS (CI+) Calcd for $C_{21}H_{30}N_2NaO_4$ $[M+Na]^+$: 397.2098, found: 397.2099.

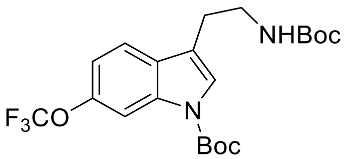

1o

***tert*-Butyl 3-(2-((*tert*-butoxycarbonyl)amino)ethyl)-6-(trifluoromethoxy)-1*H*-indole-1-carboxylate (1o)** was prepared as a viscous oil (1.09 g, 82% yield).

^{1}H NMR (400 MHz, $CDCl_3$) δ 8.06 (s, 1H), 7.52 (d, *J* = 8.4 Hz, 1H), 7.46 (s, 1H), 7.12 (d, *J* = 8.4 Hz, 1H), 4.73 (s, 1H), 3.55 – 3.30 (m, 2H), 2.89 (t, *J* = 6.7 Hz, 2H), 1.67 (s, 9H), 1.44 (s, 9H) ppm.

^{13}C NMR (100 MHz, $CDCl_3$) δ 155.9, 149.3, 146.6, 135.4, 129.1, 124.3, 120.7 (q, *J* = 256.5 Hz), 119.6, 117.6, 116.2, 108.8, 84.2, 79.3, 40.3, 28.4, 28.1, 25.5 ppm.

^{19}F NMR (376 MHz, $CDCl_3$) δ -57.92 (s) ppm.

HRMS (CI+) Calcd for $C_{21}H_{27}F_3N_2NaO_5$ $[M+Na]^+$: 467.1764, found: 467.1771.

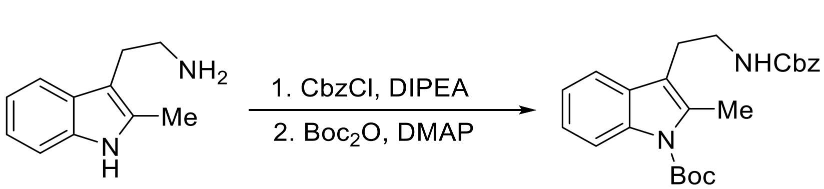

1s

***tert*-Butyl 3-(2-(((benzyloxy)carbonyl)amino)ethyl)-1*H*-indole-1-carboxylate (1s)**. At 0 °C, to a solution of 2-(2-Methyl-1*H*-indol-3-yl)ethan-1-amine (523 mg, 3 mmol) and DIPEA (0.7 mL, 6 mmol) in DCM (15 mL) was added a solution of CbzCl (0.56 g, 3.3 mmol) in DCM (5 mL) dropwise. The reaction was warmed

to room temperature and stirred for 1 h. The reaction mixture was successively washed with HCl (20 mL, 2 M), saturated aqueous $NaHCO_3$ (20 mL) and brine (20 mL). The organic phase was dried over Na_2SO_4 and concentrated. The crude product was used directly for the next step.

At 0 °C, to a solution of the above crude product and DMAP (36.6 mg, 0.3 mmol) in DCM (15 mL) was added Boc_2O (1.31 g, 6.0 mmol) dropwise. The reaction was warmed to room temperature and stirred for 12 h. The solvent was removed under reduced pressure, and the residue was directly subjected to silica gel flash chromatography to afford **1s** as a white solid (1.04 g, 78% yield).

^{1}H NMR (400 MHz, $CDCl_3$) δ 8.09 (d, *J* = 8.0 Hz, 1H), 7.43 (d, *J* = 7.4 Hz, 1H), 7.39 – 7.26 (m, 5H), 7.26 – 7.11 (m, 2H), 5.09 (s, 2H), 4.86 (s, 1H), 3.47 – 3.25 (m, 2H), 2.89 (t, *J* = 6.8 Hz, 2H), 2.47 (s, 3H), 1.67 (s, 9H) ppm.

^{13}C NMR (100 MHz, $CDCl_3$) δ 156. 5, 150.7, 136.6, 135.8, 134.3, 129.7, 128.6, 128.2 (2C), 123.5, 122.6, 117.7, 115.5, 115.0, 83.7, 66.6, 40.9, 28.3, 24.5, 14.0 ppm.

HRMS (CI+) Calcd for $C_{24}H_{28}N_2NaO_4$ $[M+Na]^+$: 431.1941, found: 431.1944.

Preparation of 1u and 1v.

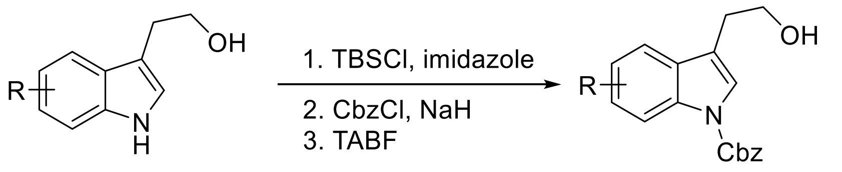

1u and **1v**

At room temperature, to a solution of tryptophol (3 mmol) and imidazole (0.41 g, 6 mmol) in DCM (15 mL) was added a solution of TBSCl (0.54 g, 3.6 mmol) in DCM (5 mL) dropwise. The mixture was stirred for 0.5 h, and then diluted with DCM (20 mL) and washed with H_2O (20 mL) and brine (20 mL). The organic phase was dried over Na_2SO_4 and concentrated.

The crude product was dissolved in DMF (1 mL). At 0 °C, this solution was added dropwise to a mixture of NaH (200 mg, 60 wt%, 5 mmol) and DMF (10

mL). The mixture was stirred for 0.5 h before CbzCl (853 mg, 5 mmol) was added dropwise at 0 ° C. Then reaction mixture was warmed to room temperature and stirred overnight. H_2O (30 mL) was added to quench the reaction, and the mixture was extracted with Et_2O (20 mL × 3). The combined organic layers were dried over Na_2SO_4 and concentrated.

The above residual was dissolved in THF (10 mL), to which a solution of TBAF (4 mL, 1 M in THF, 4 mmol) was added. After stirring for 1 h, the reaction mixture was diluted with EtOAc (30 mL) and then washed with H_2O (20 mL × 2), dried over Na_2SO_4 and concentrated. The crude product was purified by silica gel chromatography (eluent: *n*-hexane/EtOAc = 20:1→5:1).

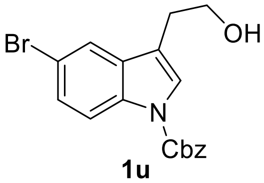

1u

Benzyl 5-bromo-3-(2-hydroxyethyl)-1*H*-indole-1-carboxylate (1u) was obtained as a white solid.

^{1}H NMR (400 MHz, $CDCl_3$) δ 7.98 (s, 1H), 7.66 – 7.59 (m, 1H), 7.51 – 7.30 (m, 7H), 5.37 (s, 2H), 3.85 (t, *J* = 6.1 Hz, 2H), 2.83 (t, *J* = 6.4 Hz, 2H), 1.97 (s, 1H) ppm.

^{13}C NMR (100 MHz, $CDCl_3$) δ 150.5, 134.9, 134.3, 132.4, 128.9, 128.8, 128.6, 127.6, 124.2, 121.9, 117.7, 116.8, 116.4, 68.9, 61.7, 28.2 ppm.

HRMS (CI+) Calcd for $C_{18}H_{16}BrNNaO_3$ $[M+Na]^+$: 396.0206, found: 396.0210.

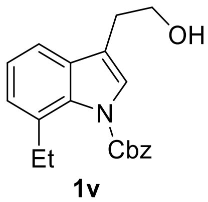

1v

Benzyl 7-ethyl-3-(2-hydroxyethyl)-1*H*-indole-1-carboxylate (1v) was obtained as a viscous oil.

^{1}**H NMR** (400 MHz, $CDCl_3$) δ 7.50 – 7.42 (m, 3H), 7.42 – 7.28 (m, 4H), 7.25 – 7.12 (m, 2H), 5.35 (s, 2H), 3.83 (t, *J* = 6.5 Hz, 2H), 3.11 (t, *J* = 7.4 Hz, 2H), 2.87 (t, *J* = 6.4, 2H), 1.87 (s, 1H), 1.20 (t, *J* = 7.4 Hz, 3H) ppm.

^{13}**C NMR** (100 MHz, $CDCl_3$) δ 150.8, 135.2, 134.5, 132.3, 132.2, 128.75, 128.79, 128.6, 126.8, 125.5, 123.7, 117.9, 116.7, 68.9, 61.8, 28.4, 28.0, 15.3 ppm.

HRMS (CI+) Calcd for $C_{20}H_{21}NNaO_3$ $[M+Na]^+$: 346.1414, found: 346.1420.

Preparation of 3c, 3e and 3f.

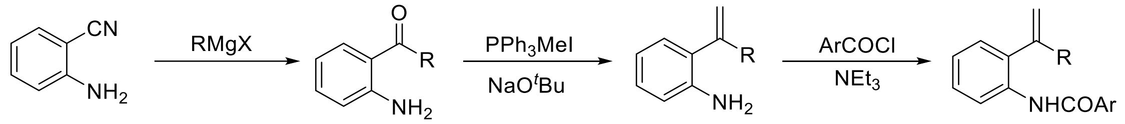

3c, **3e** and **3f**

At 0 °C, to a solution of 2-aminobenzonitrile (0.59 g, 5 mmol) in THF (20 mL) was added a solution of the Grignard reagent RMgX (15 mmol, 3 M in THF) dropwise. The mixture was stirred at room temperature overnight. An aqueous HCl solution (20 mL, 2 M) was cautiously added and the mixture was stirred for 1 h. A saturated aqueous $NaHCO_3$ solution (30 mL) was added to neutralize the mixture, which was then extracted with EtOAc (30 mL × 3). The combined organic layers were dried over Na_2SO_4 and concentrated. The crude ketone was purified by silica gel chromatography (eluent: *n*-hexane/EtOAc = 20:1→5:1).

At 0 °C, to a suspension solution of methyltriphenylphosphonium iodide (4.04 g, 10 mmol) in THF (40 mL) was added NaO^{t}Bu (0.96 g, 10 mmol). After stirring at room temperature for 0.5 h, a solution of the above prepared ketone in THF (5 mL) was added. The mixture was warmed to 50 °C and stirred overnight. After cooling to room temperature, a saturated aqueous NH_4Cl solution (20 mL) and hexane (30 mL) were added successively. The mixture was rigorously stirred for 5 min and then filtered and washed with Et_2O. The organic layer in the filtrate was separated, dried and concentrated. The olefin product was purified by silica gel chromatography (eluent: *n*-hexane/EtOAc = 20:1→10:1).

The obtained olefin and Et_3N (1.4 mL, 10 mmol) were dissolved in DCM (15 mL) and cooled to 0 °C, to which was added a solution of the acyl chloride ArCOCl (5 mmol) in DCM (2 mL) dropwise. After stirring at room temperature for 1 h, the mixture was diluted with DCM (50 mL) and washed sequentially with an aqueous HCl solution (20 mL, 1 M), a saturated aqueous $NaHCO_3$ solution (20 mL), and brine. The organic layer was dried and concentrated. The residue was purified by silica gel chromatography (eluent: *n*-hexane/EtOAc = 20:1→10:1).

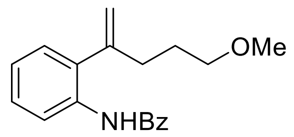

3c

***N*-(2-(4-Methoxybutanoyl)phenyl)benzamide (3c)** was obtained as a viscous oil.

^{1}H NMR (400 MHz, $CDCl_3$) δ 8.58 – 8.44 (m, 2H), 7.87 – 7.77 (m, 2H), 7.59 – 7.42 (m, 3H), 7.37 – 7.28 (m, 1H), 7.19 – 7.06 (m, 2H), 5.48 (s, 1H), 5.14 (s, 1H), 3.35 (t, *J* = 6.1 Hz, 2H), 3.20 (s, 3H), 2.56 – 2.42 (m, 2H), 1.76 – 1.61 (m, 2H) ppm.

^{13}C NMR (100 MHz, $CDCl_3$) δ 165.1, 147.2, 135.1, 134.6, 132.8, 131.8, 128.9, 128.1, 127.9, 127.0, 123. 9, 120.9, 116.3, 72.2, 58.6, 35.1, 27.9 ppm.

HRMS (CI+) Calcd for $C_{19}H_{21}NNaO_2$ $[M+Na]^+$: 318.1465, found: 318.1470.

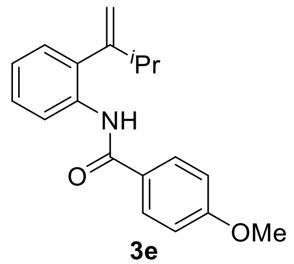

3e

4-Methoxy-*N*-(2-(3-methylbut-1-en-2-yl)phenyl)benzamide (3e) was obtained as a white solid.

^{1}H NMR (400 MHz, $CDCl_3$) δ 8.50 (d, *J* = 8.2 Hz, 1H), 8.32 (s, 1H), 7.87 – 7.68 (m,

2H), 7.40 – 7.27 (m, 1H), 7.16 – 7.04 (m, 2H), 7.04 – 6.90 (m, 2H), 5.49 – 5.39 (m, 1H), 5.09 (s, 1H), 3.85 (s, 3H), 2.69 – 2.50 (m, 1H), 1.09 (d, *J* = 6.8 Hz, 6H) ppm.

^{13}C NMR (100 MHz, $CDCl_3$) δ 164.4, 162.4, 153.9, 135.0, 133.1, 128.7, 128.2, 127.9, 127.3, 123.3, 120.3, 114.1, 113.6, 55.5, 35.5, 21.5 ppm.

HRMS (CI+) Calcd for $C_{19}H_{21}NNaO_2$ [M+Na]$^+$: 318.1465, found: 318.1466.

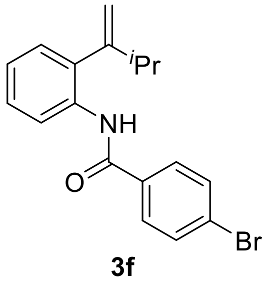

3f

4-Bromo-*N*-(2-(3-methylbut-1-en-2-yl)phenyl)benzamide (3f) was obtained as a white solid.

^{1}H NMR (400 MHz, $CDCl_3$) δ 8.47 (d, *J* = 8.2 Hz, 1H), 8.34 (s, 1H), 7.70 – 7.64 (m, 2H), 7.64 – 7.58 (m, 2H), 7.36 – 7.29 (m, 1H), 7.15 – 7.08 (m, 2H), 5.49 – 5.39 (m, 1H), 2.59 (hept, *J* = 6.7 Hz, 1H), 1.08 (d, *J* = 6.8 Hz, 6H) ppm.

^{13}C NMR (100 MHz, $CDCl_3$) δ 163.9, 153.8, 134.5, 133.9, 133.2, 132.1, 128.4, 128.3, 128.0, 126.6, 123.8, 120.4, 113.8, 35.5, 21.5 ppm.

HRMS (CI+) Calcd for $C_{18}H_{18}BrNNaO$ [M+Na]$^+$: 366.0464, found: 366.0470.

2.4 Asymmetric Electrochemical Indole Bromocyclization

General Procedure A

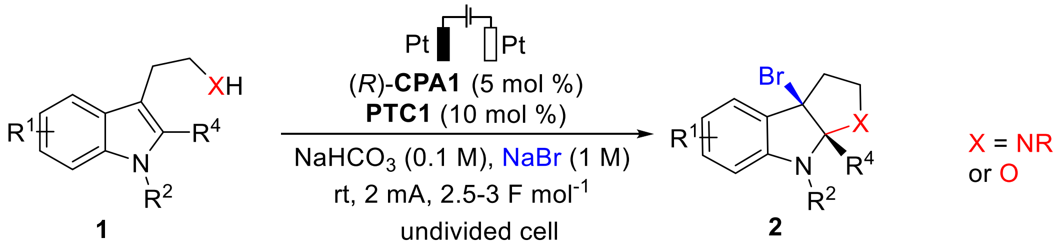

To an undivided vial (25 mL) equipped with platinum anode (10 x 15 x 0.2 mm), platinum cathode (10 x 15 x 0.2 mm), and two magnetic stir bars (oval, 6 x 10 mm) were added the indole substrate **1** (0.3 mmol), (*R*)-**CPA1** (12.5 mg, 0.015 mmol, 5 mol %), **PTC1** (11.3 mg, 0.03 mmol, 10 mol %), $NaHCO_3$ (42 mg, 0.5 mmol), NaBr (515 mg, 5.0 mmol), toluene (6 mL) and H_2O (5 mL). This vial was placed on a stir plate with a stirring speed of 1000 r/min. The electrolysis was carried out with a constant current of 4 mA for 5–6 h (2.5–3.0 F mol^{-1}). The reaction completion was determined by TLC. The organic layer of the reaction mixture was separated and the aqueous layer was extracted with EtOAc (5 mL x 2). The combined organic layers were dried over anhydrous Na_2SO_4, filtered, and concentrated. The residue was purified by silica gel chromatography to yield the desired product **2**.

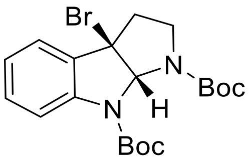

2a

Di-*tert*-butyl (3a*R*,8a*R*)-3a-bromo-2,3,3a,8a-tetrahydropyrrolo[2,3-b]indole-1,8-dicarboxylate (2a) was prepared as a white solid from **1a** (108 mg, 0.3 mmol) according to the General Procedure A (6 h, eluent: *n*-hexane/EtOAc = 20:1→10:1, 129 mg, 98% yield, 95% ee).

$[\alpha]_D^{25}$: -184.6 (c = 1.0, $CHCl_3$). HPLC analysis of the product: Daicel CHIRALPAK® IC column; 1% *i*-PrOH in *n*-hexane; 1 mL/min; retention times: 8.2 min (major), 12.5 min (minor).

^{1}H NMR (400 MHz, $CDCl_3$) δ 7.76 – 7.48 (m, 1H), 7.36 (d, J = 7.6 Hz, 1H), 7.33 – 7.22 (m, 1H), 7.15 – 7.04 (m, 1H), 6.44 (s, 1H), 3.73 (dd, J = 9.7, 7.2 Hz, 1H), 2.90 – 2.63 (m, 3H), 1.59 (s, 9H), 1.49 (s, 9H) ppm.

^{13}C NMR (100 MHz, $CDCl_3$) δ 153.4, 152.2, 142.1, 132.7, 130.3, 124.1, 123.8, 117.4, 83.9, 82.1, 80.8, 62.2, 46.2, 41.2, 28.4, 28.3 ppm.

HRMS (CI+) Calcd for $C_{20}H_{27}BrN_2NaO_4$ $[M+Na]^+$: 461.1046, found: 461.1053.

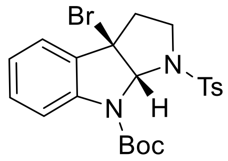

2b

***tert*-Butyl (3a*R*,8a*S*)-3a-bromo-1-tosyl-2,3,3a,8a-tetrahydropyrrolo[2,3-b]indole-8(1*H*)-carboxylate (2b)** was prepared as a white solid from **1b** (124 mg, 0.3 mmol) according to the General Procedure A (5.5 h, eluent: *n*-hexane/EtOAc = 20:1→10:1, 147 mg, 99% yield, 94% ee).

$[\alpha]_D^{25}$: -142.3 (c = 1.0, $CHCl_3$). HPLC analysis of the product: Daicel CHIRALPAK® IC column; 20% *i*-PrOH in *n*-hexane; 1 mL/min; retention times: 13.0 min (minor), 31.9 min (major).

^{1}H NMR (400 MHz, $CDCl_3$) δ 7.74 (d, J = 8.1 Hz, 2H), 7.51 (d, J = 7.1 Hz, 1H), 7.35 – 7.20 (m, 4H), 7.13 – 6.99 (m, 1H), 6.41 (s, 1H), 3.73 (dd, J = 11.1, 7.7 Hz, 1H), 2.93 – 2.80 (m, 1H), 2.73 (dd, J = 12.4, 4.7 Hz, 1H), 2.64 – 2.48 (m, 1H), 2.41 (s, 3H), 1.58 (s, 9H) ppm.

^{13}C NMR (100 MHz, $CDCl_3$) δ 151.8, 143.6, 141.5, 137.0, 131.9, 130.5, 129.6, 127.1, 124.3, 123.8, 116.9, 85.9, 83.0, 62.1, 48.4, 42.2, 28.2, 21.6 ppm.

HRMS (CI+) Calcd for $C_{22}H_{25}BrN_2NaO_4S$ $[M+Na]^+$: 515.0611, found: 515.0617.

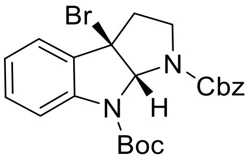

2c

1-Benzyl 8-(*tert*-butyl) (3a*R*,8a*R*)-3a-bromo-2,3,3a,8a-tetrahydropyrrolo[2,3-b]indole-1,8-dicarboxylate (2c) was prepared as a viscous oil from **1c** (118 mg, 0.3 mmol) according to the General Procedure A (5 h, eluent: *n*-hexane/EtOAc = 10:1→5:1, 140 mg, 98% yield, 96% ee).

$[\alpha]_D^{25}$: -183.5 (*c* = 1.0, $CHCl_3$). HPLC analysis of the product: Daicel CHIRALPAK® IC column; 10% *i*-PrOH in *n*-hexane; 1 mL/min; retention times: 8.2 min (major), 11.2 min (minor).

^{1}H NMR (400 MHz, $CDCl_3$) δ 7.63 (s, 1H), 7.43 – 7.26 (m, 7H), 7.14 – 7.04 (m, 1H), 6.46 (s, 1H), 5.26 – 5.11 (m, 2H), 3.78 (dd, *J* = 10.8, 7.7 Hz, 1H), 2.96 – 2.67 (m, 3H), 1.54 (s, 9H) ppm.

^{13}C NMR (100 MHz, $CDCl_3$) δ 154.0, 152.1, 142.0, 136.5, 132.4, 130.5, 128.5, 128.1, 128.0, 124.2, 123.8, 117.5, 84.1, 82.3, 67.3, 62.0, 46.4, 41.3, 28.2 ppm.

HRMS (CI+) Calcd for $C_{23}H_{25}BrN_2NaO_4$ $[M+Na]^+$: 495.0890, found: 495.0892.

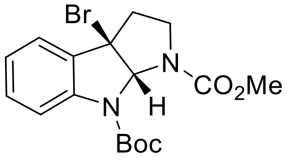

2d

8-(*tert*-Butyl) 1-methyl (3a*R*,8a*R*)-3a-bromo-2,3,3a,8a-tetrahydropyrrolo[2,3-b]indole-1,8-dicarboxylate (2d) was prepared as a viscous oil from **1d** (95.4 mg, 0.3 mmol) according to the General Procedure A (5 h, eluent: *n*-hexane/EtOAc = 10:1→5:1, 122 mg, 99% yield, 95% ee).

$[\alpha]_D^{25}$: -175.6 (*c* = 1.0, $CHCl_3$). HPLC analysis of the product: Daicel CHIRALPAK® IC column; 10% *i*-PrOH in *n*-hexane; 1 mL/min; retention times: 9.2 min (major), 11.9 min (minor).

^{1}H NMR (400 MHz, $CDCl_3$) δ 7.66 (d, *J* = 8.0 Hz, 1H), 7.38 (d, *J* = 7.6 Hz, 1H),

7.35 – 7.26 (m, 1H), 7.16 – 7.08 (m, 1H), 6.40 (s, 1H), 3.85 – 3.70 (m, 4H), 2.98 – 2.67 (m, 3H), 1.61 (s, 9H) ppm.

^{13}C NMR (100 MHz, $CDCl_3$) δ 154.7, 152.12, 142.0, 132. 3, 130.5, 124.2, 123.7, 117.4, 84.0, 82.2, 62.1, 52.8, 46.3, 41.1, 28.3 ppm.

HRMS (CI+) Calcd for $C_{17}H_{21}BrN_2NaO_4$ [M+Na]$^+$: 419.0577, found: 419.0582.

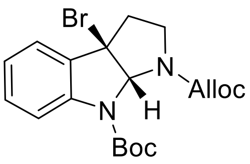

2e

1-Allyl 8-(*tert*-butyl) (3a*R*,8a*R*)-3a-bromo-2,3,3a,8a-tetrahydropyrrolo[2,3-b]indole-1,8-dicarboxylate (2e) was prepared as a viscous oil from **1e** (103 mg, 0.3 mmol) according to the General Procedure A (6 h, eluent: *n*-hexane/EtOAc = 20:1→10:1, 129 mg, 99% yield, 97% ee).

$[\alpha]_D^{25}$: -177.7 (c = 1.0, $CHCl_3$). HPLC analysis of the product: Daicel CHIRALPAK® IC column; 4% *i*-PrOH in *n*-hexane; 1 mL/min; retention times: 10.6 min (major), 15.0 min (minor).

^{1}H NMR (400 MHz, $CDCl_3$) δ 7.63 (d, J = 6.7 Hz, 1H), 7.37 (d, J = 7.6 Hz, 1H), 7.29 (dd, J = 14.6, 7.0 Hz, 1H), 7.20 – 7.01 (m, 1H), 6.42 (s, 1H), 5.95 (ddd, J = 22.6, 10.8, 5.5 Hz, 1H), 5.31 (d, J = 17.1 Hz, 1H), 5.21 (d, J = 10.4 Hz, 1H), 4.64 (d, J = 5.3 Hz, 2H), 3.88 – 3.71 (m, 1H), 3.00 – 2.67 (m, 3H), 1.58 (s, 9H) ppm.

^{13}C NMR (100 MHz, $CDCl_3$) δ 153.9, 152.1, 142.0, 132.8, 132.4, 130.5, 124.2, 123.7, 117.7, 117.5, 84.0, 82.2, 66.3, 62.0, 46.3, 41.0, 28.3 ppm.

HRMS (CI+) Calcd for $C_{19}H_{23}BrN_2NaO_4$ [M+Na]$^+$: 445.0733, found: 445.0738.

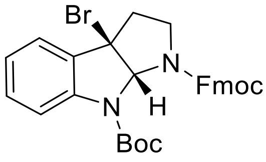

2f

1-((9*H*-Fluoren-9-yl)methyl) 8-(*tert*-butyl) (3a*R*,8a*R*)-3a-bromo-2,3,3a,8a-

tetrahydropyrrolo[2,3-b]indole-1,8-dicarboxylate (2f) was prepared as a white solid from **1f** (145 mg, 0.3 mmol) according to the General Procedure A (6 h, eluent: *n*-hexane/EtOAc = 20:1→10:1, 164 mg, 97% yield, 97% ee).

$[\alpha]_D^{25}$: -146.2 (*c* = 1.0, $CHCl_3$). HPLC analysis of the product: Daicel CHIRALPAK® IC column; 20% *i*-PrOH in *n*-hexane; 1 mL/min; retention times: 7.2 min (major), 12.6 min (minor).

^{1}H NMR (400 MHz, $CDCl_3$) δ 7.74 (d, *J* = 7.5 Hz, 2H), 7.69 (d, *J* = 7.2 Hz, 1H), 7.61 (s, 1H), 7.55 (d, *J* = 7.3 Hz, 1H), 7.44 – 7.25 (m, 6H), 7.16 – 7.06 (m, 1H), 6.50 (s, 1H), 4.59 – 4.19 (m, 3H), 3.72 (s, 1H), 3.04 – 2.58 (m, 3H), 1.52 (s, 9H) ppm.

^{13}C NMR (100 MHz, $CDCl_3$) δ 154.1, 152.2, 144.0 (2C), 142.0, 141.4, 141.3, 132.4, 130.6, 127.8 (2C), 127.2, 127.1, 125.3, 125.1, 124.3, 123.9, 120.1, 120.0, 117.4, 84.3, 82.4, 67.8, 62.1, 47.1, 46.4, 41.4, 28.3 ppm.

HRMS (CI+) Calcd for $C_{30}H_{29}BrN_2NaO_4$ $[M+Na]^+$: 583.1203, found: 583.1211.

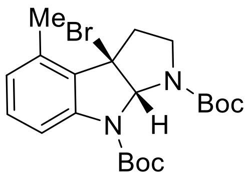

2g

Di-*tert*-butyl (3a*R*,8a*R*)-3a-bromo-4-methyl-2,3,3a,8a-tetrahydropyrrolo[2,3-b]indole-1,8-dicarboxylate (2g) was prepared as a white solid from **1g** (112 mg, 0.3 mmol) according to the General Procedure A (6 h, eluent: *n*-hexane/EtOAc = 20:1→10:1, 121 mg, 89% yield, 82% ee).

$[\alpha]_D^{25}$: -106.6 (*c* = 1.0, $CHCl_3$). HPLC analysis of the product: Daicel CHIRALPAK® IC column; 1% *i*-PrOH in *n*-hexane; 1 mL/min; retention times: 7.4 min (major), 10.3 min (minor).

^{1}H NMR (400 MHz, $CDCl_3$) δ 7.58 – 7.37 (m, 1H), 7.24 – 7.14 (m, 1H), 6.85 (d, *J* = 7.6 Hz, 1H), 6.48 (s, 1H), 3.74 (dd, *J* = 11.1, 7.7 Hz, 1H), 3.01 (dd, *J* = 12.2, 4.6 Hz, 1H), 2.88 – 2.76 (m, 1H), 2.70 – 2.59 (m, 1H), 2.48 (s, 3H), 1.57 (s, 9H), 1.49 (s, 9H) ppm.

^{13}C NMR (100 MHz, $CDCl_3$) δ 153.6, 152.2, 142.7, 135.1, 130.3, 129.1, 126.3, 114.9, 84.5, 82.0, 80.7, 63.6, 46.2, 40.2, 28.4, 28.3, 18.5 ppm.

HRMS (CI+) Calcd for $C_{21}H_{29}BrN_2NaO_4$ $[M+Na]^+$: 475.1203, found: 475.1210.

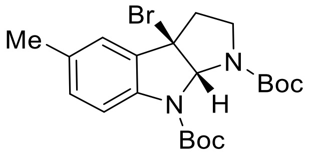

2h

Di-*tert*-butyl (3a*R*,8a*R*)-3a-bromo-5-methyl-2,3,3a,8a-tetrahydropyrrolo[2,3-b]indole-1,8-dicarboxylate (2h) was prepared as a white solid from **1h** (112 mg, 0.3 mmol) according to the General Procedure A (5.5 h, eluent: *n*-hexane/EtOAc = 20:1→10:1, 134 mg, 98% yield, 93% ee).

$[\alpha]_D^{25}$: -154.0 (*c* = 1.0, $CHCl_3$). HPLC analysis of the product: Daicel CHIRALPAK® IC column; 4% *i*-PrOH in *n*-hexane; 1 mL/min; retention times: 6.1 min (major), 8.7 min (minor).

^{1}H NMR (400 MHz, $CDCl_3$) δ 7.45 (s, 1H), 7.16 (s, 1H), 7.09 (d, *J* = 8.3 Hz, 1H), 6.42 (s, 1H), 3.72 (dd, *J* = 10.7, 7.4 Hz, 1H), 2.88 – 2.63 (m, 3H), 2.32 (s, 3H), 1.58 (s, 9H), 1.49 (s, 9H) ppm.

^{13}C NMR (100 MHz, $CDCl_3$) δ 153.4, 152.2, 139.9, 133.8, 132.7, 131.1, 124.0, 117.3, 84.0, 81.9, 80.7, 62.5, 46.1, 41.5, 28.4, 28.3, 21.0 ppm.

HRMS (CI+) Calcd for $C_{21}H_{29}BrN_2NaO_4$ $[M+Na]^+$: 475.1203, found: 475.1209.

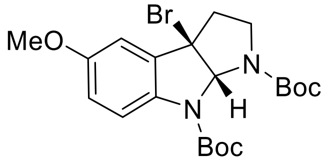

2i

Di-*tert*-butyl (3a*R*,8a*R*)-3a-bromo-5-methoxy-2,3,3a,8a-tetrahydropyrrolo [2,3-b]indole-1,8-dicarboxylate (2i) was prepared as a white solid from **1i** (117 mg, 0.3 mmol) according to the General Procedure A (5 h, eluent: *n*-hexane/EtOAc = 10:1, 138 mg, 98% yield, 92% ee).

$[\alpha]_D^{25}$: -141.1 (*c* = 1.0, $CHCl_3$). HPLC analysis of the product: Daicel CHIRALPAK® IC column; 4% *i*-PrOH in *n*-hexane; 1 mL/min; retention times: 7.6 min (major), 10.7 min (minor).

^{1}H NMR (400 MHz, $CDCl_3$) δ 7.42 (s, 1H), 6.92 – 6.79 (m, 2H), 6.41 (s, 1H), 3.80 (s, 3H), 3.72 (dd, *J* = 10.6, 7.3 Hz, 1H), 2.89 – 2.64 (m, 3H), 1.57 (s, 9H), 1.49 (s, 9H) ppm.

^{13}C NMR (100 MHz, $CDCl_3$) δ 156.7, 153.4, 152.3, 135.7, 133.8, 118.5, 116.3, 108.5, 84.1, 81.8, 80.7, 62.4, 60.4, 55.8, 46.0, 28.4, 28.3 ppm.

HRMS (CI+) Calcd for $C_{21}H_{29}BrN_2NaO_5$ $[M+Na]^+$: 491.1152, found: 491.1159.

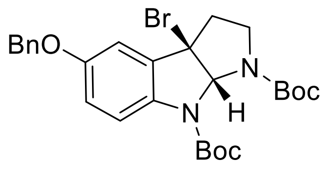

2j

Di-*tert*-butyl (3a*R*,8a*R*)-5-(benzyloxy)-3a-bromo-2,3,3a,8a-tetrahydropyrrolo [2,3-b]indole-1,8-dicarboxylate (2j) was prepared as a white solid from **1j** (140 mg, 0.3 mmol) according to the General Procedure A (6 h, eluent: *n*-hexane/EtOAc = 10:1, 155 mg, 95% yield, 93% ee).

$[\alpha]_D^{25}$: -106.6 (*c* = 1.0, $CHCl_3$). HPLC analysis of the product: Daicel CHIRALPAK® IC column; 4% *i*-PrOH in *n*-hexane; 1 mL/min; retention times: 8.2 min (major), 11.5 min (minor).

^{1}H NMR (400 MHz, $CDCl_3$) δ 7.65 – 7.28 (m, 6H), 7.03 – 6.83 (m, 2H), 6.41 (s, 1H), 5.03 (s, 2H), 3.71 (dd, *J* = 10.5, 6.6 Hz, 1H), 2.88 – 2.78 (m, 1H), 2.78 – 2.60 (m, 2H), 1.57 (s, 9H), 1.49 (s, 9H) ppm.

^{13}C NMR (100 MHz, $CDCl_3$) δ 155.8, 153.4, 152.3, 136.7, 136.0, 133.8, 128.6, 128.1, 127.6, 118.4, 117.1, 109.9, 84.1, 81.9, 80.8, 70.7, 62.3, 46.1, 41.2, 28.4, 28.3 ppm.

HRMS (CI+) Calcd for $C_{27}H_{33}BrN_2NaO_5$ $[M+Na]^+$: 567.1465, found: 567.1472.

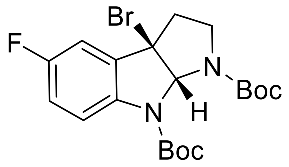

2k

Di-*tert*-butyl (3a*R*,8a*R*)-3a-bromo-5-fluoro-2,3,3a,8a-tetrahydropyrrolo[2,3-b]indole-1,8-dicarboxylate (2k) was prepared as a white solid from **1k** (114 mg, 0.3 mmol) according to the General Procedure A (6 h, eluent: *n*-hexane/EtOAc = 20:1→10:1, 133 mg, 95% yield, 97% ee).

$[\alpha]_D^{25}$: -185.2 (*c* = 1.0, $CHCl_3$). HPLC analysis of the product: Daicel CHIRALPAK® IC column; 1% *i*-PrOH in *n*-hexane; 1 mL/min; retention times: 6.5 min (major), 10.4 min (minor).

^{1}H NMR (400 MHz, $CDCl_3$) δ 7.56 (s, 1H), 7.06 (dd, *J* = 7.7, 2.6 Hz, 1H), 7.03 – 6.94 (m, 1H), 6.44 (s, 1H), 3.84 – 3.69 (m, 1H), 2.92 – 2.79 (m, 1H), 2.79 – 2.63 (m, 2H), 1.58 (s, 9H), 1.49 (s, 9H) ppm.

^{13}C NMR (100 MHz, $CDCl_3$) δ 159.4 (d, $^1J_{C-F}$ = 243.3 Hz), 153.3, 152.07, 138.2, 134.3, 118.6, 117.2 (d, $^2J_{C-F}$ = 23.3 Hz), 110.6 (d, $^2J_{C-F}$ = 24.4 Hz), 84.3, 82.3, 80.9, 61.4, 46.1, 41.4, 28.4, 28.3 ppm.

^{19}F NMR (376 MHz, $CDCl_3$) δ -118.3 ppm.

HRMS (CI+) Calcd for $C_{20}H_{26}BrFN_2NaO_4$ $[M+Na]^+$: 479.0952, found: 479.0958.

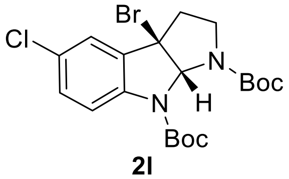

2l

Di-*tert*-butyl (3a*R*,8a*R*)-3a-bromo-5-chloro-2,3,3a,8a-tetrahydropyrrolo[2,3-b]indole-1,8-dicarboxylate (2l) was prepared as a white solid from **1l** (119 mg, 0.3 mmol) according to the General Procedure A (6 h, eluent: *n*-hexane/EtOAc = 20:1→10:1, 136 mg, 96% yield, 97% ee).

$[\alpha]_D^{25}$: -147.4 (*c* = 1.0, $CHCl_3$). HPLC analysis of the product: Daicel CHIRALPAK® IC column; 4% *i*-PrOH in *n*-hexane; 1 mL/min; retention times:

5.0 min (major), 6.5 min (minor).

^{1}H NMR (400 MHz, $CDCl_3$) δ 7.55 (s, 1H), 7.36 – 7.30 (m, 1H), 7.25 (dd, *J* = 8.7, 2.2 Hz, 1H), 6.44 (s, 1H), 3.77 (dd, *J* = 11.0, 6.7 Hz, 1H), 2.93 – 2.63 (m, 3H), 1.58 (s, 9H), 1.49 (s, 9H) ppm.

^{13}C NMR (100 MHz, $CDCl_3$) δ 153.3, 151.9, 140.7, 134.4, 130.4, 128.9, 124.0, 118.4, 84.3, 82.5, 80.9, 61.2, 46.2, 41.6, 28.4, 28.2 ppm.

HRMS (CI+) Calcd for $C_{20}H_{26}BrClN_2NaO_4$ [M+Na]$^+$: 495.0657, found: 495.0665.

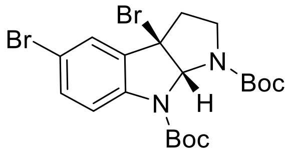

2m

Di-*tert*-butyl (3a*R*,8a*R*)-3a,5-dibromo-2,3,3a,8a-tetrahydropyrrolo[2,3-b]indole-1,8-dicarboxylate (2m) was prepared as a white solid from **1m** (132 mg, 0.3 mmol) according to the General Procedure A (6 h, eluent: *n*-hexane/EtOAc = 20:1→10:1, 145 mg, 93% yield, 94% ee).

$[\alpha]_D^{25}$: -125.5 (*c* = 1.0, $CHCl_3$). HPLC analysis of the product: Daicel CHIRALPAK® IC column; 2% *i*-PrOH in *n*-hexane; 1 mL/min; retention times: 5.7 min (major), 7.9 min (minor).

^{1}H NMR (400 MHz, $CDCl_3$) δ 7.66 – 7.44 (m, 2H), 7.39 (d, *J* = 8.7 Hz, 1H), 6.44 (s, 1H), 3.77 (dd, *J* = 11.0, 6.9 Hz, 1H), 2.92 – 2.63 (m, 3H), 1.59 (s, 9H), 1.49 (s, 9H) ppm.

^{13}C NMR (100 MHz, $CDCl_3$) δ 153.3, 151.8, 141.2, 134.7, 133.3, 126.9, 118.7, 116.2, 84.2, 82.5, 80.9, 61.1, 46.2, 41.8, 28.4, 28.3 ppm.

HRMS (CI+) Calcd for $C_{20}H_{26}Br_2N_2NaO_4$ [M+Na]$^+$: 539.0152, found: 539.0155.

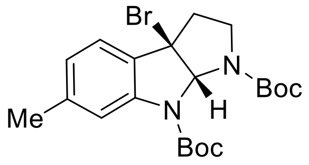

2n

Di-*tert*-butyl (3a*R*,8a*R*)-3a-bromo-6-methyl-2,3,3a,8a-tetrahydropyrrolo[2,3-b]indole-1,8-dicarboxylate (2n) was prepared as a viscous oil from **1n** (112 mg, 0.3 mmol) according to the General Procedure A (6 h, eluent: *n*-hexane/EtOAc = 20:1→10:1, 125 mg, 92% yield, 93% ee).

$[\alpha]_D^{25}$: -190.9 (*c* = 1.0, $CHCl_3$). HPLC analysis of the product: Daicel CHIRALPAK® IC column; 1% *i*-PrOH in *n*-hexane; 1 mL/min; retention times: 7.1 min (major), 10.4 min (minor).

^{1}H NMR (400 MHz, $CDCl_3$) δ 7.48 (s, 1H), 7.25 (d, *J* = 7.8 Hz, 1H), 6.93 (d, *J* = 7.8 Hz, 1H), 6.44 (s, 1H), 3.73 (dd, *J* = 10.3, 7.5 Hz, 1H), 2.89 – 2.65 (m, 3H), 2.36 (s, 3H), 1.60 (s, 9H), 1.50 (s, 9H) ppm.

^{13}C NMR (100 MHz, $CDCl_3$) δ 153.4, 152.3, 142.3, 140. 8, 129.9, 125.0, 123.4, 117.9, 84.2, 82.0, 80.7, 62.6, 46.3, 42.0, 28.4, 28.3, 21.9 ppm.

HRMS (CI+) Calcd for $C_{21}H_{29}BrN_2NaO_4$ $[M+Na]^+$: 475.1203, found: 475.1207.

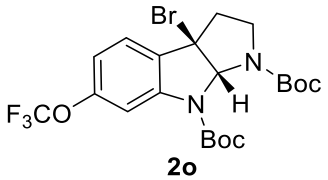

2o

Di-*tert*-butyl (3a*R*,8a*R*)-3a-bromo-6-(trifluoromethoxy)-2,3,3a,8a-tetrahydro pyrrolo[2,3-b]indole-1,8-dicarboxylate (2o) was prepared as a white solid from **1o** (133 mg, 0.3 mmol) according to the General Procedure A (6 h, eluent: *n*-hexane/EtOAc = 20:1→10:1, 144 mg, 92% yield, 97% ee).

$[\alpha]_D^{25}$: -162.5 (*c* = 1.0, $CHCl_3$). HPLC analysis of the product: Daicel CHIRALPAK® IC column; 1% *i*-PrOH in *n*-hexane; 1 mL/min; retention times: 4.7 min (major), 6.8 min (minor).

^{1}H NMR (400 MHz, $CDCl_3$) δ 7.52 (s, 1H), 7.36 (d, *J* = 8.4 Hz, 1H), 6.93 (d, *J* = 8.3

Hz, 1H), 6.48 (s, 1H), 3.78 (dd, J = 10.8, 6.7 Hz, 1H), 2.91 – 2.66 (m, 3H), 1.59 (s, 9H), 1.49 (s, 9H) ppm.

^{13}C NMR (100 MHz, $CDCl_3$) δ 153.3, 151.5, 150.6, 143.3, 131.1, 124.8, 120.4 (q, $^1J_{C-F}$ = 257.8 Hz), 116.4, 110.1, 84.6, 82.8, 81.0, 61.1, 46.2, 41.8, 28.3, 28.1 ppm.

^{19}F NMR (376 MHz, $CDCl_3$) δ -57.7 ppm.

HRMS (CI+) Calcd for $C_{21}H_{26}BrF_3N_2NaO_5$ [M+Na]$^+$: 545.0869, found: 545.0875.

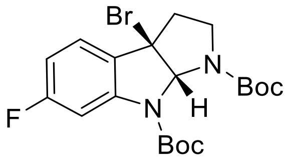

2p

Di-*tert*-butyl (3a*R*,8a*R*)-3a-bromo-6-fluoro-2,3,3a,8a-tetrahydropyrrolo[2,3-b]indole-1,8-dicarboxylate (2p) was prepared as a viscous oil from **1p** (114 mg, 0.3 mmol) according to the General Procedure A (6 h, eluent: *n*-hexane/EtOAc = 20:1→10:1, 124 mg, 90% yield, 96% ee).

$[\alpha]_D^{25}$: -174.9 (c = 1.0, $CHCl_3$). HPLC analysis of the product: Daicel CHIRALPAK® IC column; 2% *i*-PrOH in *n*-hexane; 1 mL/min; retention times: 5.5 min (major), 7.8 min (minor).

^{1}H NMR (400 MHz, $CDCl_3$) δ 7.44 – 7.23 (m, 2H), 6.87 – 6.72 (m, 1H), 6.46 (s, 1H), 3.76 (dd, J = 10.7, 7.2 Hz, 1H), 2.93 – 2.62 (m, 3H), 1.59 (s, 9H), 1.49 (s, 9H) ppm.

^{13}C NMR (100 MHz, $CDCl_3$) δ 164.1 (d, $^1J_{C-F}$ = 246.8 Hz), 153.3, 151.7, 143.6 (d, $^1J_{C-F}$ = 12.8 Hz), 128.3, 125.0 (d, $^3J_{C-F}$ = 10.5 Hz), 111.0 (d, $^2J_{C-F}$ = 23.4 Hz), 105.03 (d, $^2J_{C-F}$ = 21.8 Hz), 84.7, 82.7, 80.9, 61.7, 46.3, 42.1, 28.4, 28.2 ppm.

^{19}F NMR (376 MHz, $CDCl_3$) δ -109.29 – -109.58 (m) ppm.

HRMS (CI+) Calcd for $C_{20}H_{26}BrFN_2NaO_4$ [M+Na]$^+$: 479.0952, found: 479.0958.

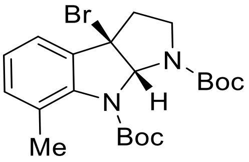

2q

Di-*tert*-butyl (3a*R*,8a*R*)-3a-bromo-7-methyl-2,3,3a,8a-tetrahydropyrrolo[2,3-b]indole-1,8-dicarboxylate (2q) was prepared as a viscous oil from **1q** (112 mg, 0.3 mmol) according to the General Procedure A (6 h, eluent: *n*-hexane/EtOAc = 20:1→10:1, 132 mg, 97% yield, 92% ee).

$[\alpha]_D^{25}$: -125.7 (*c* = 1.0, $CHCl_3$). HPLC analysis of the product: Daicel CHIRALPAK® IC column; 2% *i*-PrOH in *n*-hexane; 1 mL/min; retention times: 11.6 min (major), 12.9 min (minor).

^{1}H NMR (400 MHz, $CDCl_3$) δ 7.24 – 7.17 (m, 1H), 7.17 – 7.07 (m, 2H), 6.24 (s, 1H), 3.59 – 3.47 (m, 1H), 2.87 – 2.62 (m, 3H), 2.30 (s, 3H), 1.53 (s, 9H), 1.50 (s, 9H) ppm.

^{13}C NMR (100 MHz, $CDCl_3$) δ 153.7, 153.6, 141.6, 135.1, 132.4, 131.0, 126.2, 120.3, 86.0, 82.0, 80.6, 62.2, 45.6, 37.9, 28.6, 28.2, 19.3 ppm.

HRMS (CI+) Calcd for $C_{21}H_{29}BrN_2NaO_4$ $[M+Na]^+$: 475.1203, found: 475.1208.

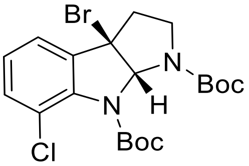

2r

Di-*tert*-butyl (3a*R*,8a*R*)-3a-bromo-7-chloro-2,3,3a,8a-tetrahydropyrrolo[2,3-b]indole-1,8-dicarboxylate (2r) was prepared as a white solid from **1r** (119 mg, 0.3 mmol) according to the General Procedure A (6 h, eluent: *n*-hexane/EtOAc = 20:1→10:1, 140 mg, 98% yield, 91% ee).

$[\alpha]_D^{25}$: -109.4 (*c* = 1.0, $CHCl_3$). HPLC analysis of the product: Daicel CHIRALPAK® IC column; 2% *i*-PrOH in *n*-hexane; 1 mL/min; retention times: 5.6 min (major), 6.3 min (minor).

^{1}H NMR (400 MHz, $CDCl_3$) δ 7.58 (s, 1H), 7.29 – 7.18 (m, 1H), 7.03 (d, *J* = 8.0 Hz,

1H), 6.48 (s, 1H), 3.76 (dd, *J* = 11.3, 7.8 Hz, 1H), 3.41 (dd, *J* = 12.6, 4.5 Hz, 1H), 2.92 – 2.75 (m, 1H), 2.59 (td, *J* = 12.5, 7.9 Hz, 1H), 1.58 (s, 9H), 1.49 (s, 9H).

^{13}C NMR (100 MHz, $CDCl_3$) δ 153.4, 151.8, 144.2, 131.4, 131.0, 128.0, 125.0, 115.7, 84.6, 82.6, 80.8, 61.8, 46.4, 39.1, 28.4, 28.2 ppm.

HRMS (CI+) Calcd for $C_{20}H_{26}BrClN_2NaO_4$ [M+Na]$^+$: 495.0657, found: 495.0663.

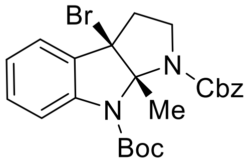

2s

1-Benzyl 8-(*tert*-butyl) (3a*R*,8a*R*)-3a-bromo-8a-methyl-2,3,3a,8a-tetrahydro pyrrolo[2,3-b]indole-1,8-dicarboxylate (2s) was prepared as a viscous oil from **1s** (123 mg, 0.3 mmol) according to the General Procedure A (6 h, eluent: *n*-hexane/EtOAc = 20:1→10:1, 143 mg, 98% yield, 76% ee).

[α]$_D^{25}$: -138.7 (*c* = 1.0, $CHCl_3$). HPLC analysis of the product: Daicel CHIRALPAK® IC column; 5% *i*-PrOH in *n*-hexane; 1 mL/min; retention times: 8.3 min (major), 9.5 min (minor).

^{1}H NMR (400 MHz, $CDCl_3$) δ 7.77 (s, 1H), 7.42 – 7.20 (m, 7H), 7.17 – 6.96 (m, 1H), 5.13 (s, 2H), 3.51 (t, *J* = 9.5 Hz, 1H), 3.03 – 2.93 (m, 1H), 2.87 (dd, *J* = 12.7, 6.5 Hz, 1H), 2.74 – 2.60 (m, 1H), 2.16 (s, 3H), 1.56 (s, 9H) ppm.

^{13}C NMR (100 MHz, $CDCl_3$) δ 153.1, 152.0, 142.2, 136.7, 131.7, 130.3, 128.5, 127.9, 127.7, 123.7, 123.1, 118.3, 88.4, 82.0, 70.2, 66.7, 45.8, 36.0, 28.4, 24.4 ppm.

HRMS (CI+) Calcd for $C_{24}H_{27}BrN_2NaO_4$ [M+Na]$^+$: 509.1046, found: 509.1050.

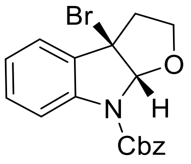

2t

Benzyl (3a*R*,8a*S*)-3a-bromo-2,3,3a,8a-tetrahydro-8H-furo[2,3-b]indole-8-carboxylate (2t) was prepared as a viscous oil from **1t** (88.5 mg, 0.3 mmol)

according to the General Procedure A (6 h, eluent: *n*-hexane/EtOAc = 20:1→10:1, 104 mg, 93% yield, 90% ee).

$[\alpha]_D^{25}$: -107.3 (*c* = 1.0, $CHCl_3$). HPLC analysis of the product: Daicel CHIRALPAK® IC column; 3% *i*-PrOH in *n*-hexane; 1 mL/min; retention times: 13.5 min (minor), 14.5 min (major).

^{1}H NMR (400 MHz, $CDCl_3$) δ 7.88 (s, 1H), 7.55 – 7.18 (m, 7H), 7.17 – 6.97 (m, 1H), 6.29 (s, 1H), 5.49 – 5.19 (m, 2H), 4.00 (t, *J* = 8.1 Hz, 1H), 3.49 (ddd, *J* = 11.2, 9.1, 4.8 Hz, 1H), 2.95 – 2.83 (m, 1H), 2.83 – 2.74 (m, 1H) ppm.

^{13}C NMR (100 MHz, $CDCl_3$) δ 152.4, 141.5, 135.9, 131.7, 130.6, 128.7, 128.4, 128.0, 124.9, 124.2, 115.0, 100.7, 68.0, 67.6, 61.8, 45.0 ppm.

HRMS (CI+) Calcd for $C_{18}H_{16}BrNNaO_3$ $[M+Na]^+$: 396.0206, found: 396.0210.

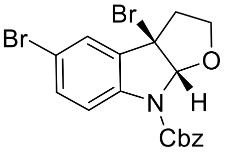

2u

Benzyl (3a*R*,8a*S*)-3a,5-dibromo-2,3,3a,8a-tetrahydro-8*H*-furo[2,3-b]indole-8-carboxylate (2u) was prepared as a viscous oil from **1u** (112 mg, 0.3 mmol) according to the General Procedure A (5.5 h, eluent: *n*-hexane/EtOAc = 20:1→ 10:1, 121 mg, 89% yield, 92% ee).

$[\alpha]_D^{25}$: -34.0 (*c* = 1.0, $CHCl_3$). HPLC analysis of the product: Daicel CHIRALPAK® OD column; 5% *i*-PrOH in *n*-hexane; 1 mL/min; retention times: 8.8 min (major), 9.6 min (minor).

^{1}H NMR (400 MHz, $CDCl_3$) δ 7.90 – 7.64 (m, 1H), 7.52 (d, *J* = 1.1 Hz, 1H), 7.50 – 7.26 (m, 6H), 6.26 (s, 1H), 5.32 (q, *J* = 12.3 Hz, 2H), 4.01 (t, *J* = 8.1 Hz, 1H), 3.50 (ddd, *J* = 11.0, 9.4, 4.7 Hz, 1H), 2.93 – 2.81 (m, 1H), 2.81 – 2.71 (m, 1H) ppm.

^{13}C NMR (100 MHz, $CDCl_3$) δ 152.2, 140.6, 135.6, 133.8, 133.5, 128.7, 128.4, 128.0, 127.9, 116.5, 116.4, 100.9, 68.0, 67.8, 60.5, 44.9 ppm.

HRMS (CI+) Calcd for $C_{18}H_{15}Br_2NNaO_3$ $[M+Na]^+$: 473.9311, found: 473.9315.

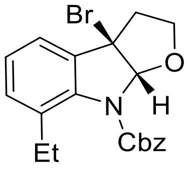

2v

Benzyl (3a*R*,8a*S*)-3a-bromo-7-ethyl-2,3,3a,8a-tetrahydro-8*H*-furo[2,3-b]indole-8-carboxylate (2v) was prepared as a viscous oil from **1v** (97.0 mg, 0.3 mmol) according to the General Procedure A (5.5 h, eluent: *n*-hexane/EtOAc = 20:1→10:1, 119 mg, 99% yield, 86% ee).

$[\alpha]_D^{25}$: -102.6 (c = 1.0, $CHCl_3$). HPLC analysis of the product: Daicel CHIRALPAK® AD column; 15% *i*-PrOH in *n*-hexane; 1 mL/min; retention times: 6.6 min (major), 9.8 min (minor).

^{1}H NMR (400 MHz, $CDCl_3$) δ 7.47 – 7.28 (m, 5H), 7.28 – 7.11 (m, 3H), 6.27 (s, 1H), 5.38 (d, J = 12.3 Hz, 1H), 5.24 (d, J = 12.3 Hz, 1H), 4.00 – 3.87 (m, 1H), 3.47 – 3.31 (m, 1H), 2.89 – 2.79 (m, 1H), 2.79 – 2.66 (m, 3H), 1.17 (t, J = 7.5 Hz, 3H) ppm.

^{13}C NMR (100 MHz, $CDCl_3$) δ 153.5, 139.3, 135.8, 134.7, 134.3, 131.1, 128.7, 128.4, 128.1, 126.3, 121.8, 102.3, 68.2, 68.1, 61.3, 43.6, 26.0, 13.7 ppm.

HRMS (CI+) Calcd for $C_{20}H_{20}BrNNaO_3$ $[M+Na]^+$: 424.0519, found: 424.0521.

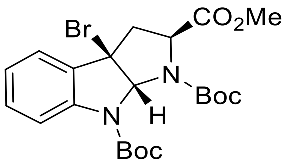

2w

1,8-Di-*tert*-butyl 2-methyl (2*S*,3a*R*,8a*R*)-3a-bromo-2,3,3a,8a-tetrahydro pyrrolo [2,3-b]indole-1,2,8-tricarboxylate (2w) was prepared as a white solid from **1w** (126 mg, 0.3 mmol) according to the General Procedure A, except that an excessive current of 3 mA was applied for 18 h (eluent: *n*-hexane/EtOAc = 10:1→5:1, 116 mg, 78% yield, > 20:1 dr).

$[\alpha]_D^{25}$: -171.6 (c = 1.0, $CHCl_3$).

^{1}H NMR (400 MHz, $CDCl_3$) δ 7.83 – 7.42 (m, 1H), 7.42 – 7.29 (m, 2H), 7.17 – 7.05

(m, 1H), 6.40 (s, 1H), 3.94 – 3.85 (m, 1H), 3.74 (s, 3H), 3.21 (dd, *J* = 12.6, 6.3 Hz, 1H), 2.82 (dd, *J* = 12.4, 10.5 Hz, 1H), 1.59 (s, 9H), 1.40 (s, 9H) ppm.

^{13}C NMR (100 MHz, $CDCl_3$) δ 171.5, 152.2, 144.2, 141.5, 132.8, 130.7, 124.4, 123.3, 119.1, 83.8, 82.3, 81.6, 59.8, 59.5, 52.4, 42.0, 28.3 (2C) ppm.

HRMS (CI+) Calcd for $C_{22}H_{29}BrN_2NaO_6$ $[M+Na]^+$: 519.1101, found: 519.1104.

2.5 Asymmetric Electrochemical 2-Amidostyrene Bromocyclization

General Procedure B

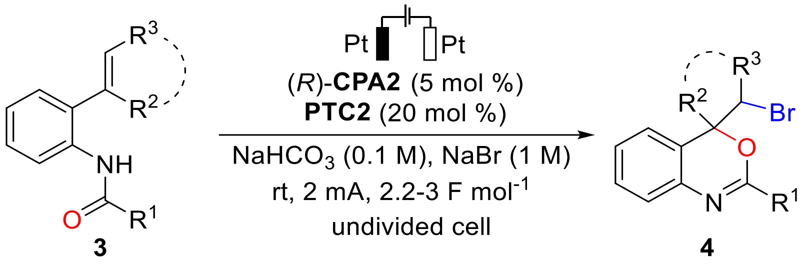

To an undivided vial (25 mL) equipped with platinum anode (10 x 15 x 0.2 mm), platinum cathode (10 x 15 x 0.2 mm), and two magnetic stir bars (oval, 6 x 10 mm) were added the substrate **3** (0.3 mmol), (*R*)-**CPA2** (11.4 mg, 0.015 mmol, 5 mol %), **PTC2** (21.1 mg, 0.06 mmol, 20 mol %), $NaHCO_3$ (42 mg, 0.5 mmol), NaBr (515 mg, 5.0 mmol), toluene (6 mL), and H_2O (5 mL). This vial was placed on a stir plate with a stirring speed of 1000 r/min. The electrolysis was carried out with a constant current of 4 mA for 4.5–6 h (2.2–3.0 F mol^{-1}). The reaction completion was determined by TLC. The organic layer of the reaction mixture was separated and the aqueous layer was extracted with EtOAc (5 mL × 2). The combined organic layers were dried over anhydrous Na_2SO_4, filtered, and concentrated. The residue was purified by silica gel chromatography to yield the desired product **4**.

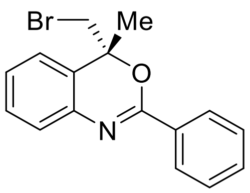

4a

(*R*)-4-(Bromomethyl)-4-methyl-2-phenyl-4*H*-benzo[*d*][1,3]oxazine (4a) was prepared as a white solid from **3a** (71.2 mg, 0.3 mmol) according to the General Procedure B (5.5 h, eluent: *n*-hexane/EtOAc = 20:1→10:1, 94.7 mg, 99% yield, 94% ee).

$[\alpha]_D^{25}$: +15.4 (c = 1.0, $CHCl_3$). HPLC analysis of the product: Daicel CHIRALPAK® OD column; 1% *i*-PrOH in *n*-hexane; 1 mL/min; retention times: 6.2 min (minor), 7.7 min (major).

^{1}H NMR (400 MHz, $CDCl_3$) δ 8.26 – 8.17 (m, 2H), 7.54 – 7.41 (m, 3H), 7.39 – 7.31 (m, 2H), 7.25 – 7.18 (m, 1H), 7.18 – 7.11 (m, 1H), 3.76 (d, J = 11.2 Hz, 1H), 3.53 (d, J = 11.2 Hz, 1H), 1.91 (s, 3H).

^{13}C NMR (100 MHz, $CDCl_3$) δ 156.2, 139.1, 132.3, 131.6, 129.6, 128.33, 128.32, 127.1, 126.8, 125.6, 123.3, 78.1, 39.8, 24.9 ppm.

HRMS (CI+) Calcd for $C_{16}H_{15}BrNO$ $[M+H]^+$: 316.0332, found: 316.0328.

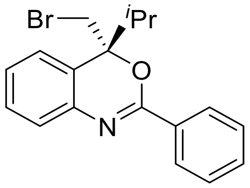

4b

(*R*)-4-(Bromomethyl)-4-isopropyl-2-phenyl-4*H*-benzo[*d*][1,3]oxazine (4b) was prepared as a viscous oil from **3b** (79.6 mg, 0.3 mmol) according to the General Procedure B (6 h, eluent: *n*-hexane/EtOAc = 20:1→10:1, 103 mg, 99% yield, 95% ee).

$[\alpha]_D^{25}$: -4.3 (c = 1.0, $CHCl_3$). HPLC analysis of the product: Daicel CHIRALPAK® OD column; 1% *i*-PrOH in *n*-hexane; 1 mL/min; retention times: 6.2 min (minor), 6.9 min (major).

^{1}H NMR (400 MHz, $CDCl_3$) δ 8.22 – 8.12 (m, 2H), 7.54 – 7.41 (m, 3H), 7.36 – 7.28 (m, 2H), 7.24 – 7.17 (m, 1H), 7.13 – 7.06 (m, 1H), 3.95 (d, J = 10.9 Hz, 1H), 3.79 (d, J = 10.9 Hz, 1H), 2.47 (hept, J = 6.8 Hz, 1H), 1.15 (d, J = 6.8 Hz, 3H), 0.96 (d, J = 6.9 Hz, 3H) ppm.

^{13}C NMR (100 MHz, $CDCl_3$) δ 155.8, 139.7, 132.5, 131.4, 129.1, 128.3, 127.8, 126.5, 125.7, 125.0, 123.8, 84.2, 39.6, 37.1, 18.0, 16.0 ppm.

HRMS (CI+) Calcd for $C_{18}H_{19}BrNO$ $[M+H]^+$: 344.0645, found: 344.0654.

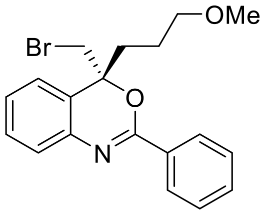

4c

(*R*)-4-(Bromomethyl)-4-(3-methoxypropyl)-2-phenyl-4*H*-benzo[*d*][1,3] oxazine (4c) was prepared as a viscous oil from **3c** (88.6 mg, 0.3 mmol) according to the General Procedure B (6 h, eluent: *n*-hexane/EtOAc = 10:1→5:1, 110 mg, 98% yield, 96% ee).

$[\alpha]_D^{25}$: -2.4 (c = 1.0, $CHCl_3$). HPLC analysis of the product: Daicel CHIRALPAK® OD column; 1% *i*-PrOH in *n*-hexane; 1 mL/min; retention times: 15.6 min (major), 16.6 min (minor).

^{1}H NMR (400 MHz, $CDCl_3$) δ 8.26 – 8.10 (m, 2H), 7.55 – 7.39 (m, 3H), 7.38 – 7.30 (m, 2H), 7.26 – 7.16 (m, 1H), 7.15 – 7.06 (m, 1H), 3.76 (d, J = 11.2 Hz, 1H), 3.62 (d, J = 11.2 Hz, 1H), 3.35 (t, J = 6.1 Hz, 2H), 3.26 (s, 3H), 2.46 – 2.32 (m, 1H), 2.26 – 2.12 (m, 1H), 1.78 – 1.64 (m, 1H), 1.64 – 1.50 (m, 1H) ppm.

^{13}C NMR (100 MHz, $CDCl_3$) δ 156.0, 139.7, 132.3, 131.6, 129.5, 128.3, 128.2, 126.8, 125.8, 124.9, 123.7, 81.1, 72.2, 58.6, 40.4, 34.7, 24.3 ppm.

HRMS (CI+) Calcd for $C_{19}H_{21}BrNO_2$ $[M+H]^+$: 374.0750, found: 374.0755.

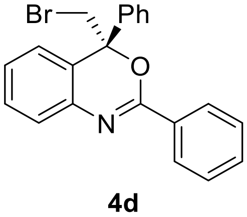

4d

(*R*)-4-(Bromomethyl)-2,4-diphenyl-4*H*-benzo[*d*][1,3]oxazine (4d) was prepared as a viscous oil from **3d** (89.8 mg, 0.3 mmol) according to the General Procedure B (6 h, eluent: *n*-hexane/EtOAc = 20:1→10:1, 105 mg, 93% yield, 97% ee).

$[\alpha]_D^{25}$: -66.7 (c = 1.0, $CHCl_3$). HPLC analysis of the product: Daicel CHIRALPAK® AD column; 1% *i*-PrOH in *n*-hexane; 1 mL/min; retention times:

12.1 min (minor), 12.8 min (maior).

^{1}H NMR (400 MHz, $CDCl_3$) δ 8.28 (d, *J* = 8.2 Hz, 2H), 7.54 – 7.23 (m, 10H), 7.23 – 7.16 (m, 1H), 7.06 (d, *J* = 7.6 Hz, 1H), 4.21 – 3.96 (m, 2H) ppm.

^{13}C NMR (100 MHz, $CDCl_3$) δ 156.1, 140.2, 139.5, 132.2, 131.8, 129.8, 128.7, 128.6, 128.5, 128.3, 126.7, 126.4, 126.1, 125.8, 125.0, 82.3, 37.3 ppm.

HRMS (CI+) Calcd for $C_{21}H_{17}BrNO$ $[M+H]^+$: 378.0488, found: 378.0487.

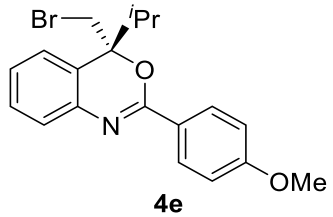

4e

(*R*)-4-(Bromomethyl)-4-isopropyl-2-(4-methoxyphenyl)-4*H*-benzo[*d*][1,3] oxazine (4e) was prepared as a viscous oil from **3e** (80.2 mg, 0.3 mmol) according to the General Procedure B (6 h, eluent: *n*-hexane/EtOAc = 10:1→5:1, 105 mg, 94% yield, 92% ee).

$[\alpha]_D^{25}$: +7.3 (*c* = 1.0, $CHCl_3$). HPLC analysis of the product: Daicel CHIRALPAK® OD column; 3% *i*-PrOH in *n*-hexane; 1 mL/min; retention times: 7.8 min (minor), 10.4 min (major).

^{1}H NMR (400 MHz, $CDCl_3$) δ 8.15 – 8.07 (m, 2H), 7.34 – 7.25 (m, 2H), 7.20 – 7.14 (m, 1H), 7.10 – 7.03 (m, 1H), 6.99 – 6.92 (m, 2H), 3.92 (d, *J* = 10.9 Hz, 1H), 3.84 (s, 3H), 3.77 (d, *J* = 10.9 Hz, 1H), 2.45 (hept, *J* = 6.8 Hz, 1H), 1.11 (d, *J* = 6.8 Hz, 3H), 0.94 (d, *J* = 6.9 Hz, 3H) ppm.

^{13}C NMR (100 MHz, $CDCl_3$) δ 162.3, 155.7, 140.0, 129.6, 129.0, 126.0, 125.4, 124.9, 124.8, 123.9, 113.7, 83.9, 55.5, 39.5, 36.9, 18.0, 16.0 ppm.

HRMS (CI+) Calcd for $C_{19}H_{21}BrNO_2$ $[M+H]^+$: 374.0750, found: 374.0757.

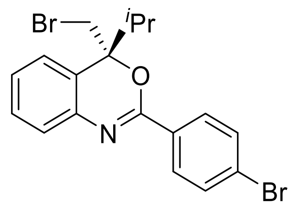

4f

(*R*)-4-(Bromomethyl)-2-(4-bromophenyl)-4-isopropyl-4*H*-benzo[*d*][1,3] oxazine (4f) was prepared as a viscous oil from **3f** (94.9 mg, 0.3 mmol) according to the General Procedure B (6 h, eluent: *n*-hexane/EtOAc = 20:1→10:1, 125 mg, 98% yield, 98% ee).

$[\alpha]_D^{25}$: +2.9 (c = 1.0, $CHCl_3$). HPLC analysis of the product: Daicel CHIRALPAK® OD column; 1% *i*-PrOH in *n*-hexane; 1 mL/min; retention times: 6.0 min (minor), 7.7 min (major).

^{1}H NMR (400 MHz, $CDCl_3$) δ 8.06 – 7.97 (m, 2H), 7.61 – 7.54 (m, 2H), 7.37 – 7.27 (m, 2H), 7.26 – 7.16 (m, 1H), 7.11 – 7.03 (m, 1H), 3.92 (d, J = 11.0 Hz, 1H), 3.76 (d, J = 11.0 Hz, 1H), 2.42 (hept, J = 6.8 Hz, 1H), 1.10 (d, J = 6.8 Hz, 3H), 0.93 (d, J = 6.9 Hz, 3H) ppm.

^{13}C NMR (100 MHz, $CDCl_3$) δ 154.9, 139.5, 131.6, 131.4, 129.3, 129.2, 126.7, 126.1, 125.7, 124.9, 123.8, 84.5, 39.7, 37.3, 18.0, 16.0 ppm.

HRMS (CI+) Calcd for $C_{18}H_{18}Br_2NO$ $[M+H]^+$: 421.9750, found: 421.9746.

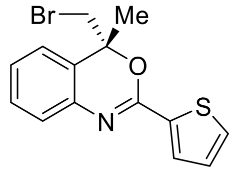

4g

(*R*)-4-(Bromomethyl)-4-methyl-2-(thiophen-2-yl)-4*H*-benzo[*d*][1,3]oxazine (4g) was prepared as a white solid from **3g** (73.0 mg, 0.3 mmol) according to the General Procedure B (5 h, eluent: *n*-hexane/EtOAc = 20:1→10:1, 96 mg, 99% yield, 89% ee).

$[\alpha]_D^{25}$: +33.7 (c = 1.0, $CHCl_3$). HPLC analysis of the product: Daicel CHIRALPAK® OD column; 1% *i*-PrOH in *n*-hexane; 1 mL/min; retention times:

8.3 min (minor), 13.2 min (major).

^{1}H NMR (400 MHz, $CDCl_3$) δ 7.82 (s, 1H), 7.56 – 7.42 (m, 1H), 7.40 – 7.26 (m, 2H), 7.26 – 7.01 (m, 3H), 3.75 (d, *J* = 11.2 Hz, 1H), 3.52 (d, *J* = 11.2 Hz, 1H), 1.90 (s, 3H) ppm.

^{13}C NMR (100 MHz, $CDCl_3$) δ 152.8, 139.0, 136.6, 130.7, 130.69, 129.67, 127.8, 127.0, 126.7, 125.3, 123.4, 78.5, 39.6, 24.8 ppm.

HRMS (CI+) Calcd for $C_{14}H_{13}BrNOS$ $[M+H]^+$: 321.9896, found: 321.9898.

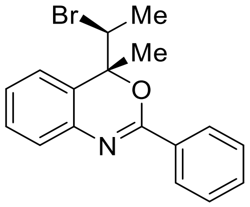

4h

(*R*)-4-((*S*)-1-Bromoethyl)-4-methyl-2-phenyl-4*H*-benzo[*d*][1,3]oxazine (4h) was prepared as a viscous oil from **3h** (75.4 mg, 0.3 mmol) according to the General Procedure B, except that 10 mol % of (*R*)**-CPA2** (22.8 mg) was used (6 h, eluent: *n*-hexane/EtOAc = 20:1→10:1, 98 mg, 99% yield, 82% ee).

$[\alpha]_D^{25}$: +6.1 (*c* = 1.0, $CHCl_3$). HPLC analysis of the product: Daicel CHIRALPAK® OD column; 1% *i*-PrOH in *n*-hexane; 1 mL/min; retention times: 5.5 min (minor), 6.7 min (major).

^{1}H NMR (400 MHz, $CDCl_3$) δ 8.30 – 8.18 (m, 2H), 7.54 – 7.40 (m, 3H), 7.39 – 7.30 (m, 2H), 7.27 – 7.16 (m, 2H), 4.42 (q, *J* = 6.9 Hz, 1H), 1.91 (s, 3H), 1.63 (d, *J* = 6.9 Hz, 3H) ppm.

^{13}C NMR (100 MHz, $CDCl_3$) δ 155.7, 138.6, 131.7, 131.0, 128.8, 127.8, 127.6, 125.9, 125.9, 124.9, 123.9, 80.8, 54.3, 21.9, 20.3 ppm.

HRMS (CI+) Calcd for $C_{17}H_{17}BrNO$ $[M+H]^+$: 330.0488, found: 330.0492.

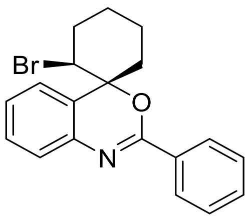

4i

(2'*S*,4*R*)-2'-Bromo-2-phenylspiro[benzo[*d*][1,3]oxazine-4,1'-cyclohexane] (4i) was prepared as a viscous oil from **3i** (89.8 mg, 0.3 mmol) according to the General Procedure B (6 h, eluent: *n*-hexane/EtOAc = 20:1→10:1, 105 mg, 99% yield, 94% ee).

$[\alpha]_D^{25}$: -16.6 (c = 1.0, $CHCl_3$). HPLC analysis of the product: Daicel CHIRALPAK® OD column; 1% *i*-PrOH in *n*-hexane; 1 mL/min; retention times: 5.1 min (major), 5.8 min (minor).

^{1}H NMR (400 MHz, $CDCl_3$) δ 8.28 – 8.14 (m, 2H), 7.54 – 7.40 (m, 3H), 7.39 – 7.24 (m, 3H), 7.24 – 7.14 (m, 1H), 4.51 (s, 1H), 2.83 – 2.70 (m, 1H), 2.51 – 2.37 (m, 1H), 2.19 – 2.01 (m, 2H), 2.01 – 1.87 (m, 2H), 1.82 (d, J = 13.3 Hz, 1H), 1.69 (d, J = 13.4 Hz, 1H) ppm.

^{13}C NMR (100 MHz, $CDCl_3$) δ 155.5, 138.8, 132.5, 131.7, 129.2, 128.5, 127.9, 127.8, 126.4, 126.0, 124.8, 79.2, 53.6, 30.0, 29.6, 20.7, 19.6 ppm.

HRMS (CI+) Calcd for $C_{19}H_{19}BrNO$ $[M+H]^+$: 356.0645, found: 356.0647.

2.6 Gram-Scale Reaction and Product Derivatizations

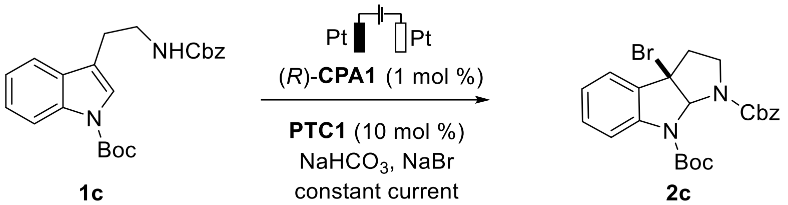

1c
10 mmol, 3.94 g

2c
4.71 g, 99.5% yield, 90% ee
catalyst recovery: 93%

In an undivided flask (cylindrical, 250 mL) equipped with platinum anode (30 × 40 × 0.2 mm), platinum cathode (30 × 40 × 0.2 mm), and a magnetic stir bar (cylindrical, 10 x 30 mm) were added the substrate **1c** (3.94g, 10 mmol), (*R*)-**CPA1** (83.3 mg, 0.1 mmol, 1 mol %), **PTC1** (375 mg, 1.0 mmol, 10 mol %), $NaHCO_3$ (2.1 g, 25 mmol), NaBr (10.3 g, 10 mmol, 1 M), toluene (100 mL), and H_2O (100 mL). The flask was placed on a stir plate with a stirring speed of 800 r/min. The electrolysis was carried out with a constant current of 80 mA for 9 h. The reaction completion was determined by TLC. The organic layer of the reaction mixture was separated and the aqueous layer was extracted with EtOAc (30 mL × 3). The combined organic layers were dried over anhydrous Na_2SO_4, filtered, and concentrated. The residue was purified by silica gel chromatography (eluent: *n*-hexane/EtOAc = 10:1→1:1) to yield **2c** (4.71 g, >99% yield, 90% ee), together with the recovered catalyst (*R*)-**CPA1** (78 mg, 93% yield) after being acidified aqueous HCl (6 M).

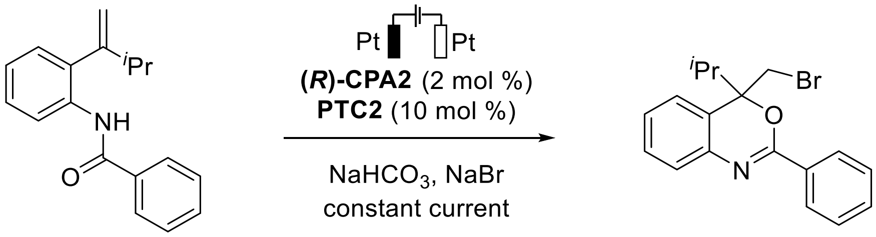

3b
5 mmol, 1.19 g

4b
1.65 g, 96% yield, 92% ee
catalyst recovery: 85%

In an undivided flask (cylindrical, 250 mL) equipped with platinum anode (30

× 40 × 0.2 mm), platinum cathode (30 × 40 × 0.2 mm), and a magnetic stir bar (cylindrical, 10 × 30 mm) were added the substrate **3b** (1.19 g, 5 mmol), (*R*)-**CPA2** (76.1 mg, 0.1 mmol, 2 mol %), **PTC2** (176 mg, 0.5 mmol, 10 mol %), $NaHCO_3$ (1.05 g, 12.5 mmol), NaBr (8.24 g, 80 mmol, 1 M), toluene (60 mL), and H_2O (80 mL). The flask was placed on a stir plate with a stirring speed of 800 r/min. The electrolysis was carried out with a constant current of 60 mA for 6.5 h. The reaction completion was determined by TLC. The organic layer of the reaction mixture was separated and the aqueous layer was extracted with EtOAc (30 mL x 3). The combined organic layers were dried over anhydrous Na_2SO_4, filtered, and concentrated. The residue was purified by silica gel chromatography (eluent: *n*-hexane/EtOAc = 10:1→1:1) to yield **4b** (1.65 g, 96% yield, 92% ee), together with the recovered catalyst (*R*)-**CPA2** (65 mg, 85% yield) after being acidified aqueous HCl (6 M).

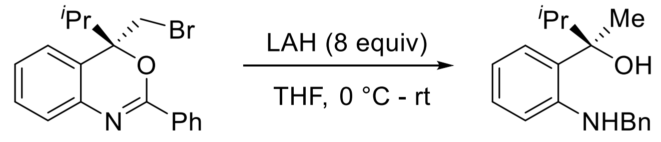

4b 0.5 mmol, 92% ee — **5** 72% yield, 93% ee

(*R*)-2-(2-(Benzylamino)phenyl)-3-methylbutan-2-ol (5). At 0 °C, to a suspension of $LiAlH_4$ (76 mg, 2 mmol) in THF (3 ml) was added a solution of compound **4b** (172 mg, 0.5 mmol) in THF (1 mL) dropwise. The mixture was stirred at room temperature for 1 h. Then, an additional portion of $LiAlH_4$ (76 mg, 2 mmol) was added and stirred for another 2 h. The mixture was cooled to 0 °C and carefully quenched by an aqueous NaOH solution (20 mL, 10 wt %). The mixture was extracted with EtOAc (10 mL × 3). The combined organic layers were dried over anhydrous Na_2SO_4, filtered, and concentrated. The residue was purified by silica gel column chromatography (eluent: *n*-hexane/EtOAc = 20:1→5:1) to yield **5** as a colorless viscous oil (97 mg, 72% yield, 93% ee).

$[\alpha]_D^{25}$: +33.2 (c = 1.0, $CHCl_3$). HPLC analysis of the product: Daicel CHIRALPAK® OD column; 7% *i*-PrOH in *n*-hexane; 1 mL/min; retention times: 9.0 min (minor), 12.8 min (major).

^{1}H NMR (400 MHz, $CDCl_3$) δ 7.39 – 7.27 (m, 4H), 7.27 – 7.18 (m, 1H), 7.11 – 7.00 (m, 2H), 6.67 – 6.56 (m, 2H), 4.43 – 4.26 (m, 2H), 2.66 (hept, *J* = 6.8 Hz, 1H), 1.54 (s, 3H), 1.02 (d, *J* = 6.8 Hz, 3H), 0.73 (d, *J* = 6.9 Hz, 3H) ppm.

^{13}C NMR (100 MHz, $CDCl_3$) δ 147.0, 140.1, 129.4, 128.6, 128.3, 127.4, 127.2, 126.9, 115.9, 112.1, 80.1, 48.1, 33.4, 23.3, 18.8, 16.9 ppm.

HRMS (CI+) Calcd for $C_{18}H_{22}N$ $[M-OH]^+$: 252.1747, found: 252.1747.

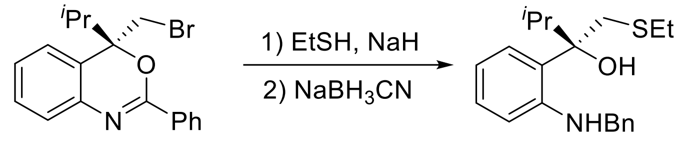

4b
0.5 mmol, 92% ee

6
66% yield, 92% ee

(*R*)-2-(2-(Benzylamino)phenyl)-1-(ethylthio)-3-methylbutan-2-ol (6). Under N_2 at 0 °C, to a suspension of NaH (1.25 mmol, 50 mg, 60% dispersion in mineral oil) in DMF (1.5 mL) was added EtSH (93 mg, 108 μL, 1.5 mmol) dropwise. The mixture was stirred for 10 min followed by addition of a solution of **4b** (163 mg, 0.5 mmol) in DMF (0.5 mL). Then, the reaction mixture was warmed to room temperature and stirred for 2 h before it was quenched with a saturated aqueous NH_4Cl solution (20 mL). The mixture was extracted with Et_2O (10 mL x 3). The combined organic layers were dried over anhydrous Na_2SO_4, and filtered through a short silica gel plug (eluent: *n*-hexane/Et_2O = 1:1). The filtrate was concentrated to afford a viscous oil, which was dissolved in AcOH (2 mL). $NaBH_3CN$ (3 mmol, 188 mg) was added in three portions over 3 h. Then, the mixture was poured into a saturated aqueous $NaHCO_3$ solution (30 mL) and extracted with Et_2O (10 mL × 3). The combined organic layers were dried over anhydrous Na_2SO_4, filtered, and concentrated. The residue was purified by silica gel column chromatography (eluent: *n*-hexane/EtOAc = 10:1

→4:1) to give thioether **5** as a colorless viscous oil (108 mg, 66% yield, 92% ee). $[\alpha]_D^{25}$: +42.0 (*c* = 1.0, $CHCl_3$). HPLC analysis of the product: Daicel CHIRALPAK® OD column; 2% *i*-PrOH in *n*-hexane; 1 mL/min; retention times: 6.7 min (minor), 7.6 min (major).

^{1}H NMR (400 MHz, $CDCl_3$) δ 7.42 – 7.31 (m, 4H), 7.30 – 7.23 (m, 1H), 7.13 – 7.05 (m, 1H), 6.95 (dd, *J* = 7.7, 1.4 Hz, 1H), 6.66 – 6.57 (m, 2H), 4.36 (s, 2H), 3.63 (d, *J* = 13.2 Hz, 1H), 2.76 (d, *J* = 13.2 Hz, 1H), 2.72 – 2.59 (m, 1H), 2.59 – 2.45 (m, 2H), 1.26 (t, *J* = 7.4 Hz, 3H), 1.05 (d, *J* = 6.9 Hz, 3H), 0.83 (d, *J* = 7.0 Hz, 3H) ppm.

^{13}C NMR (100 MHz, $CDCl_3$) δ 148.0, 140.1, 128.6, 128.5, 128.1, 127.3, 126.9, 126.0, 115.6, 112.3, 81.0, 48.1, 39.8, 34.3, 28.4, 18.1, 17.1, 15.3 ppm.

HRMS (CI+) Calcd for $C_{20}H_{26}NS$ $[M+H]^+$: 312.1780, found: 312.1784.

3. Supplementary Notes

3.1 Mechanistic Studies

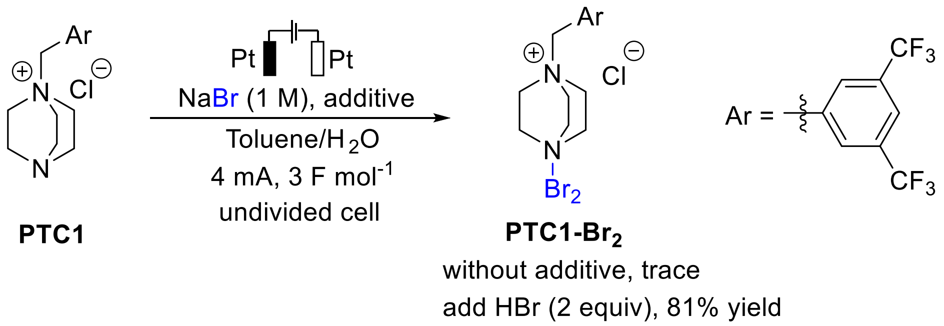

1-(3,5-Bis(trifluoromethyl)benzyl)-1,4-diazabicyclo[2.2.2]octan-1-ium-bromine (PTC1-Br_2). In two separate undivided vials (10 mL) equipped with platinum anode (10 × 10 × 0.2 mm), platinum cathode (10 × 10 × 0.2 mm), and a magnetic stir bar were added **PTC1** (0.1 mmol, 37.5 mg), NaBr (258 mg, 2.5 mmol), toluene (1 mL), and H_2O (2.5 mL). To each of the two vials was added (1) nothing and (2) HBr (16.2 mg, 48 wt % aqueous solution, 23 uL), respectively. The two vials were placed on a stir plate with a stirring speed of 1000 r/min. The electrolysis was carried out with a constant current of 4 mA for 2 h (3.0 F mol^{-1}). The suspended yellow solid was dissolved by adding acetone (1.5 mL) and separated from the aqueous phase. The aqueous phase was extracted with acetone/ethyl acetate (1:1, 2 mL × 3). The combined organic phase was dried, filtered and concentrated to obtain the desired **PTC1-Br_2**. Only a negligible amount of the yellow solid was obtained when no additive was used. When HBr was used as additive, the desired product was obtained as a yellow solid (47 mg, 81% yield, where the counter anion X^- was assumed to be Br^-).

^{1}H NMR (400 MHz, DMSO-d_6) δ 8.44 – 8.26 (m, 3H), 4.91 (s, 2H), 3.61 (t, J = 7.1 Hz, 6H), 3.35 (t, J = 7.1 Hz, 6H) ppm.

^{13}C NMR (100 MHz, DMSO-d_6) δ 134.5, 131.4 (q, $^1J_{C\text{-}F}$ = 33.2 Hz), 130.6, 124.9, 122.2, 64.9, 51.5, 44.5 ppm.

^{19}**F NMR** (376 MHz, DMSO-d_6) δ -61.2 ppm.

HRMS (CI+) Calcd for $C_{15}H_{17}F_6N_2$ $[M-Br_3]^+$: 339.1290, found: 339.1298.

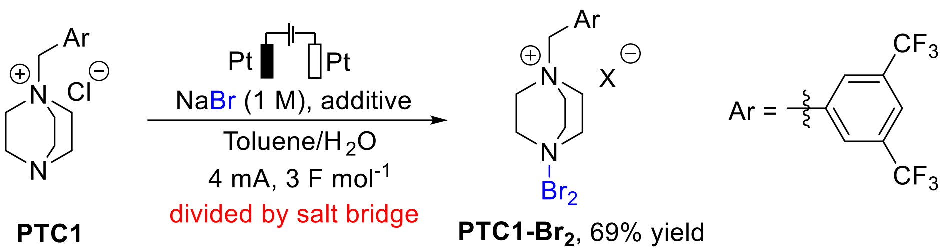

The electrolysis was carried out in a divided cell with a salt bridge. Two separate 10-mL vials were equipped with a platinum (10 × 10 × 0.2 mm) and a magnetic stir bar. To the anodic cell were added **PTC1** (0.1 mmol, 37.5 mg), NaBr (309 mg, 3.0 mmol), toluene (1 mL), and H_2O (3.0 mL). To the cathodic cell were added NaBr (309 mg, 3.0 mmol), toluene (1 mL), and H_2O (3.0 mL). The two cells were connected by a salt bridge filled with an aqueous NaBr solution (2 M). The setup was placed on a stir plate with a stirring speed of 1000 r/min. The electrolysis was carried out with a constant current of 4 mA for 2 h (3.0 F mol^{-1}). The suspended yellow solid was dissolved by adding acetone (1.5 mL). The two phases were separated. The aqueous phase was extracted with acetone/ethyl acetate (1:1, 2 mL × 3). The combined organic phases were dried, filtered and concentrated to afford the desired **PTC1-Br$_2$** (40 mg, 69% yield).

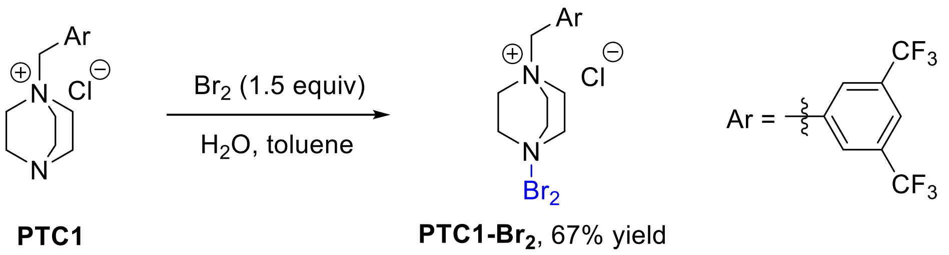

In a 10-mL vial, **PTC1** (37.5 mg, 0.1 mmol) was dissolved in H_2O (2.5 mL) and toluene (1.0 mL). Upon vigorously stirring, bromine (24 mg, 7.8 uL, 0.15 mmol) was added. The mixture was stirred for additional 10 min. The suspended yellow solid was dissolved by adding acetone (1.5 mL). The two phases were

separated. The aqueous phase was extracted with acetone/ethyl acetate (1:1, 2 mL × 3). The combined organic phases were dried, filtered and concentrated to give the desired **PTC1-Br₂** as a yellow solid (36 mg, 67% yield).

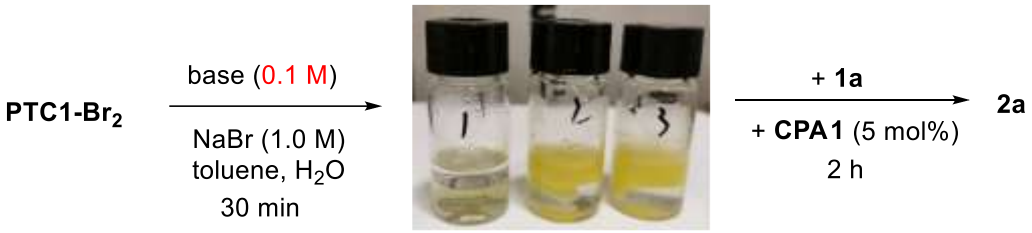

entry	base	**2a** yield, ee
1	NaOH	trace (**1a** remained)
2	$NaHCO_3$	>95%, 92% ee
3	NaOH + $NaHCO_3$	>95%, 93% ee

To three parallel vials (entries 1-3) were added NaOH (18 mg, 0.45 mmol), $NaHCO_3$ (38 mg, 0.45 mmol) and NaOH/$NaCO_3$ (8/38 mg, 0.2/0.45 mmol), respectively. An aqueous NaBr solution (2.5 mL, 1 M) was added to each of the three vials and stirred for 2 min. Then **PTC1-Br$_2$** (53.5 mg, 0.1 mmol) was added to each of the three vials. After stirring for 30 min, **1a** and (*R*)-**CPA1** were added to each of the three vials and the stirring continued for an additional 2 h. The reaction mixture was extracted with ethyl acetate (2 mL × 3). The combined organic phases were dried, filtered and concentrated. The crude product was directly subjected to silica gel flash chromatography to afford **2a** (yields and ee values were indicated in the above table for each case).

Note: After completion of the first step of the reaction, a photo was taken and presented in the above equation. The yellow insoluble solid **PTC-Br$_2$** was fully consumed under the condition of NaOH as a base (entry 1). In contrast, it stayed unchanged in the case of $NaHCO_3$ and NaOH/$NaHCO_3$ (which was regarded as a base of Na_2CO_3/$NaHCO_3$) (entries 2-3). This experiment indicated the strong base NaOH can consume **PTC-Br$_2$** and the process is irreversible.

The addition of $NaHCO_3$ to NaOH leads to formation of Na_2CO_3 and thus avoids rapid decomposition of **PTC-Br$_2$**.

Cyclic Voltammetry

The cyclic voltammetry measurements were carried out using BioLogic VMP-3 Multichannel Potentiostat. For all the experiments, a glassy-carbon (GC) electrode (3 mm-diameter, disc-electrode) was used as the working electrode, a platinum wire was used as the counter electrode and an SCE reference electrode was employed. The measurements were recorded at a scan rate of 100 mVs^{-1}.

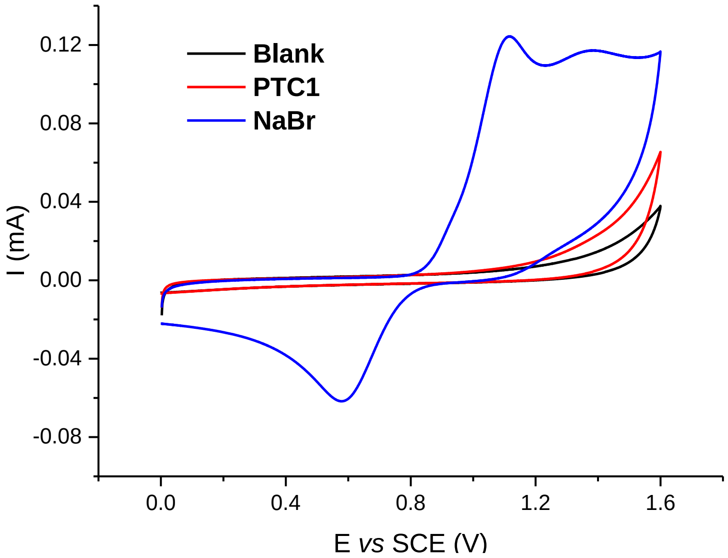

Supplementary Figure 2. The comparison of oxidation potentials of Blank (black), PTC1 (red) and NaBr (blue). Cyclic voltammetry measurements in distilled H_2O with $NaNO_3$ (0.1 M) and the substrate (5 mM) at room temperature with a scan rate of 100 mVs^{-1}.

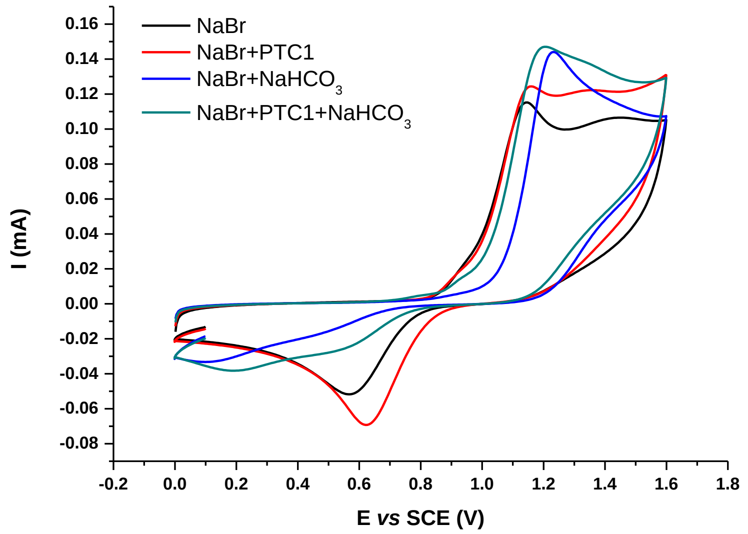

Supplementary Figure 3. Influence of **PTC1** and $NaHCO_3$ on the cyclic voltametric behavior of NaBr. Cyclic voltammetry measurements in distilled H_2O with $NaNO_3$ (0.1 M) and the substrate (5 mM) at room temperature with a scan rate of 100 mVs^{-1}.

From the above cyclic voltammetry measurements, we can see the addition of **PTC1** to the NaBr solution can induce catalytic current (red line *vs* black line in Supplementary Figure 3). However, there is a possibility that the current increment was caused by the oxidation of **PTC1** solution, rather than catalytic current. To further clarify this issue, we did the following cyclic voltammetry studies, measuring the CV curves of blank and NaBr solution in the presence of **PTC1** at different concentrations. Increasing the concentration of **PTC1** led to slight shift of the oxidation peaks of NaBr from 1.11 V to 1.22 V (Supplementary Figure 4). Therefore, we measured the current increment of the **PTC1** solution at these two potentials. The current change at 1.11 V is 0.007 mA (ΔI_1) and 1.22 V is 0.011 mA (ΔI_2), respectively. Both these two currents are smaller than the current increment of the NaBr solution (ΔI_3, 0.043 mA). This experiment further illustrated that **PTC1** can induce catalytic current of NaBr,

but not just simple addition of the current of **PTC1** to that of NaBr.

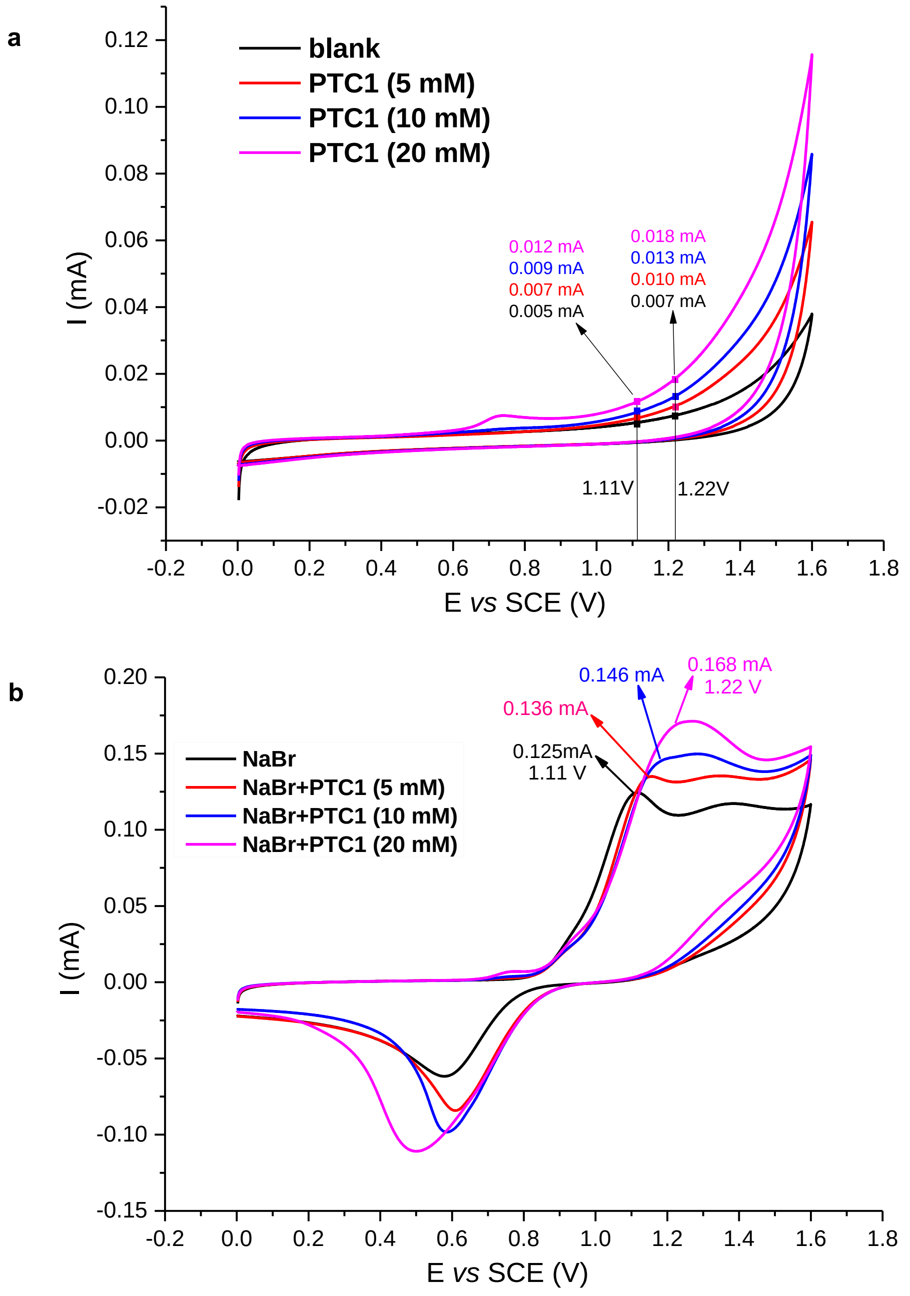

Supplementary Figure 4. **a**, Cyclic voltametric studies of **PTC1** at different concentrations. Cyclic voltammetry measurements in distilled H_2O with $NaNO_3$ (0.1 M) at room temperature with a scan rate of 100 mVs^{-1}. **b**, Cyclic voltametric studies of NaBr with different concentrations of **PTC1**. Cyclic voltammetry measurements in distilled H_2O with $NaNO_3$ (0.1 M) and NaBr (5 mM) at room temperature with a scan rate of 100 mVs^{-1}.

3.2 Determination of the Product Stereochemistry

The absolute configurations of product **2a**-**w** were determined by comparison of the optical rotation values with the literature values.[2,5]

For example:

2a: $[\alpha]_D^{25}$: -184.6 (*c* = 1.0, $CHCl_3$, 95% ee); Literature value: $[\alpha]_D^{25.3}$: -175.5 (*c* = 1.0, $CHCl_3$, 95% ee).[2]

2w: $[\alpha]_D^{25}$: -157.3 (*c* = 1.0, $CHCl_3$); Literature value: $[\alpha]_D^{25}$: -171.6 (*c* = 0.635, $CHCl_3$).[5]

For products **4a**-**4i**, most of them are known compounds.[6] However, the optical rotation values were not given in the literature. Thus, the absolute configurations were determined by comparison of retention times of the chiral HPLC signals under identical conditions. We also compared the catalyst configuration that induced the same products, which is consistent with our assignment.

For example, the following comparison was performed for product (*R*)-**4a**.

HPLC conditions: Daicel CHIRALPAK® OD column; 1% *i*-PrOH in *n*-hexane; 1 mL/min).

Retention times for (*R*)-**4a** in the literature: 7.4 min (minor), 9.4min (major).[6]

Retention times for (*R*)-**4a** from our reaction: 6.2 min (minor), 7.7 min (major).

4. Supplementary References

(1) Wang, Y.-M.; Wu, J.; Hoong, C.; Rauniyar, V.; Toste, F. D. Enantioselective halocyclization using reagents tailored for chiral anion phase-transfer catalysis. *J. Am. Chem. Soc.* **134**, 12928-12931 (2012).

(2) Xie, W.; Jiang, G.; Liu, H.; Hu, J.; Pan, X.; Zhang, H.; Wan, X.; Lai, Y.; Ma, D. Highly enantioselective bromocyclization of tryptamines and its application in the synthesis of (−)-chimonanthine. *Angew. Chem. Int. Ed.* **52**, 12924-12927 (2013).

(3) Liu, K.; Jiang, H.-J.; Li, N.; Li, H.; Wang, J.; Zhang, Z.-Z.; Yu, J. Enantioselective bromocyclization of tryptamines induced by chiral co(iii)-complex-templated brønsted acids under an air atmosphere. *J. Org. Chem.* **83**, 6815-6823 (2018).

(4) Xu, J.; Tong, R. An environmentally friendly protocol for oxidative halocyclization of tryptamine and tryptophol derivatives. *Green Chem.* **19**, 2952-2956 (2017).

(5) Espejo, V. R. & Rainier, J. D. An expeditious synthesis of C(3)−N(1′) heterodimeric indolines. *J. Am. Chem. Soc.* **130**, 12894-12895 (2008).

(6) Phipps, R. J., Hiramatsu, K. & Toste, F. D. Asymmetric fluorination of enamides: access to α-fluoroimines using an anionic chiral phase-transfer catalyst. *J. Am. Chem. Soc.* **134**, 8376-8379 (2012).

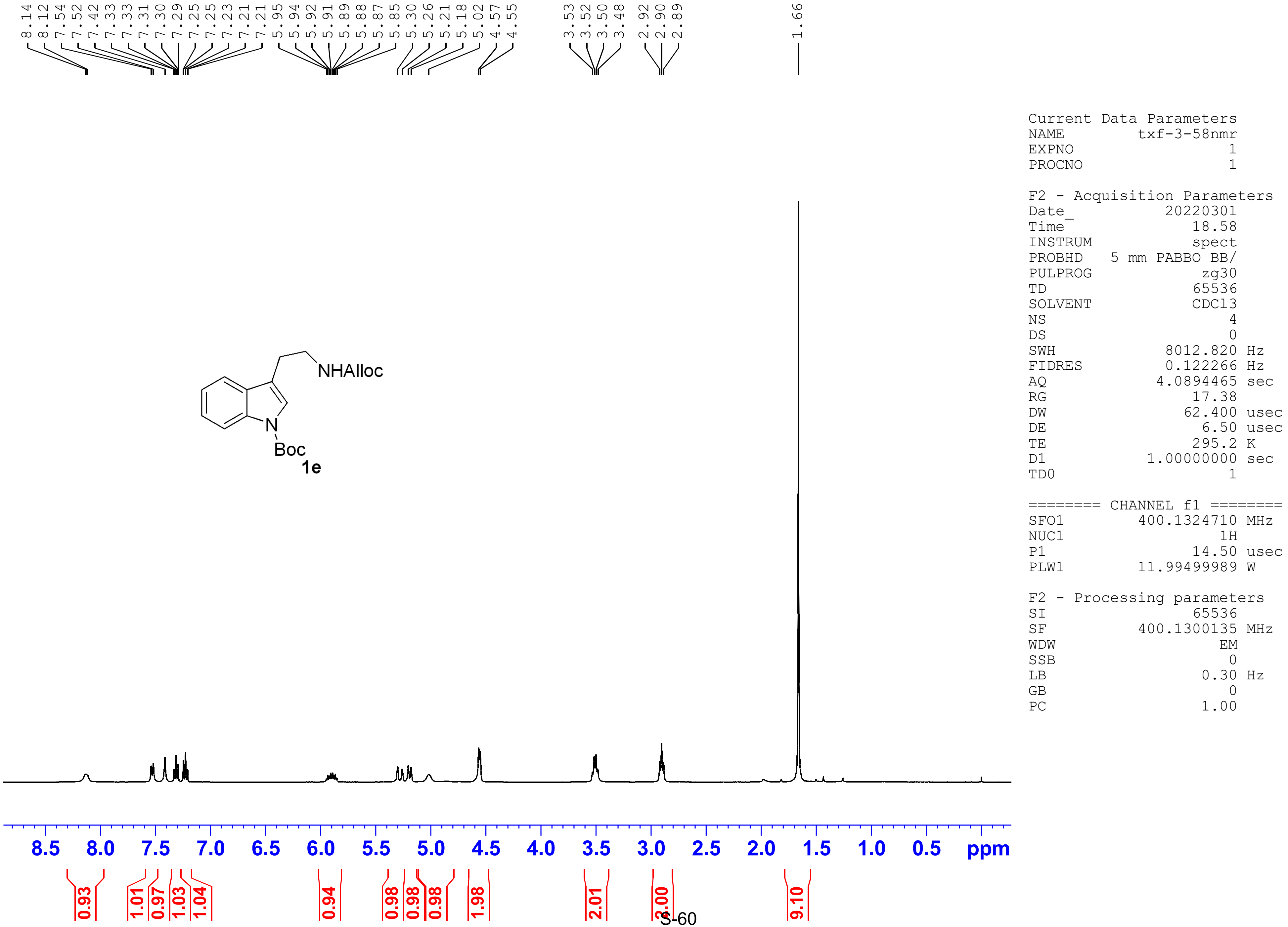

Supplementary Figure 5. ^{1}H NMR spectrum of **1e** (400 MHz, r.t., $CDCl_3$)

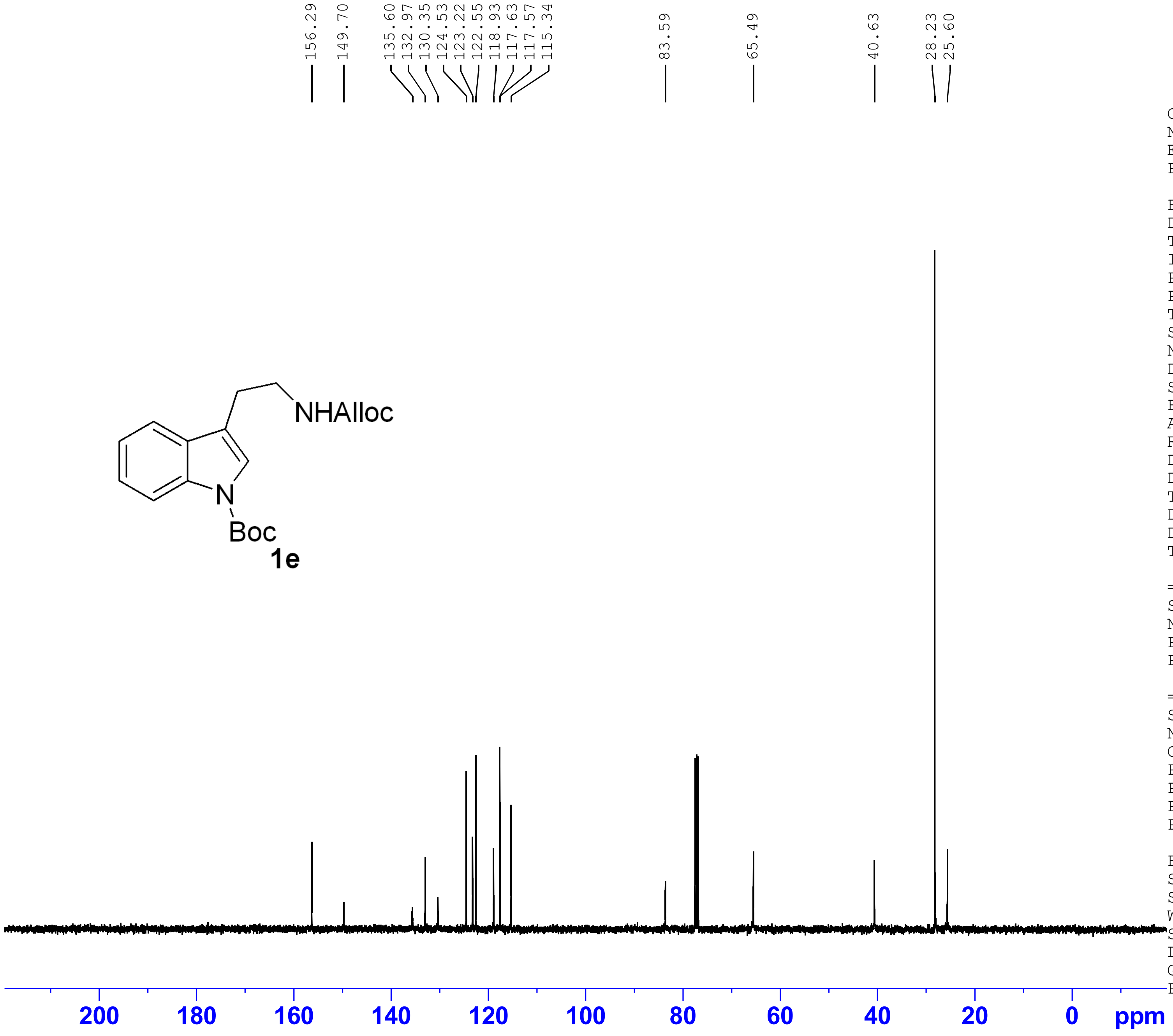

Current Data Parameters
NAME txf-3-58nmr
EXPNO 2
PROCNO 1

F2 - Acquisition Parameters
Date_ 20220301
Time 19.00
INSTRUM spect
PROBHD 5 mm PABBO BB/
PULPROG zgpg30
TD 65536
SOLVENT CDCl3
NS 61
DS 2
SWH 24038.461 Hz
FIDRES 0.366798 Hz
AQ 1.3631488 sec
RG 196.92
DW 20.800 usec
DE 6.50 usec
TE 295.7 K
D1 2.00000000 sec
D11 0.03000000 sec
TD0 1

======== CHANNEL f1 ========
SFO1 100.6228298 MHz
NUC1 13C
P1 9.70 usec
PLW1 46.98899841 W

======== CHANNEL f2 ========
SFO2 400.1316005 MHz
NUC2 1H
CPDPRG[2 waltz16
PCPD2 90.00 usec
PLW2 11.99499989 W
PLW12 0.34213999 W
PLW13 0.27713001 W

F2 - Processing parameters
SI 32768
SF 100.6127690 MHz
WDW EM
SSB 0
LB 1.00 Hz
GB 0
PC 1.40

Supplementary Figure 6. ^{13}C NMR spectrum of **1e** (100 MHz, r.t., $CDCl_3$)

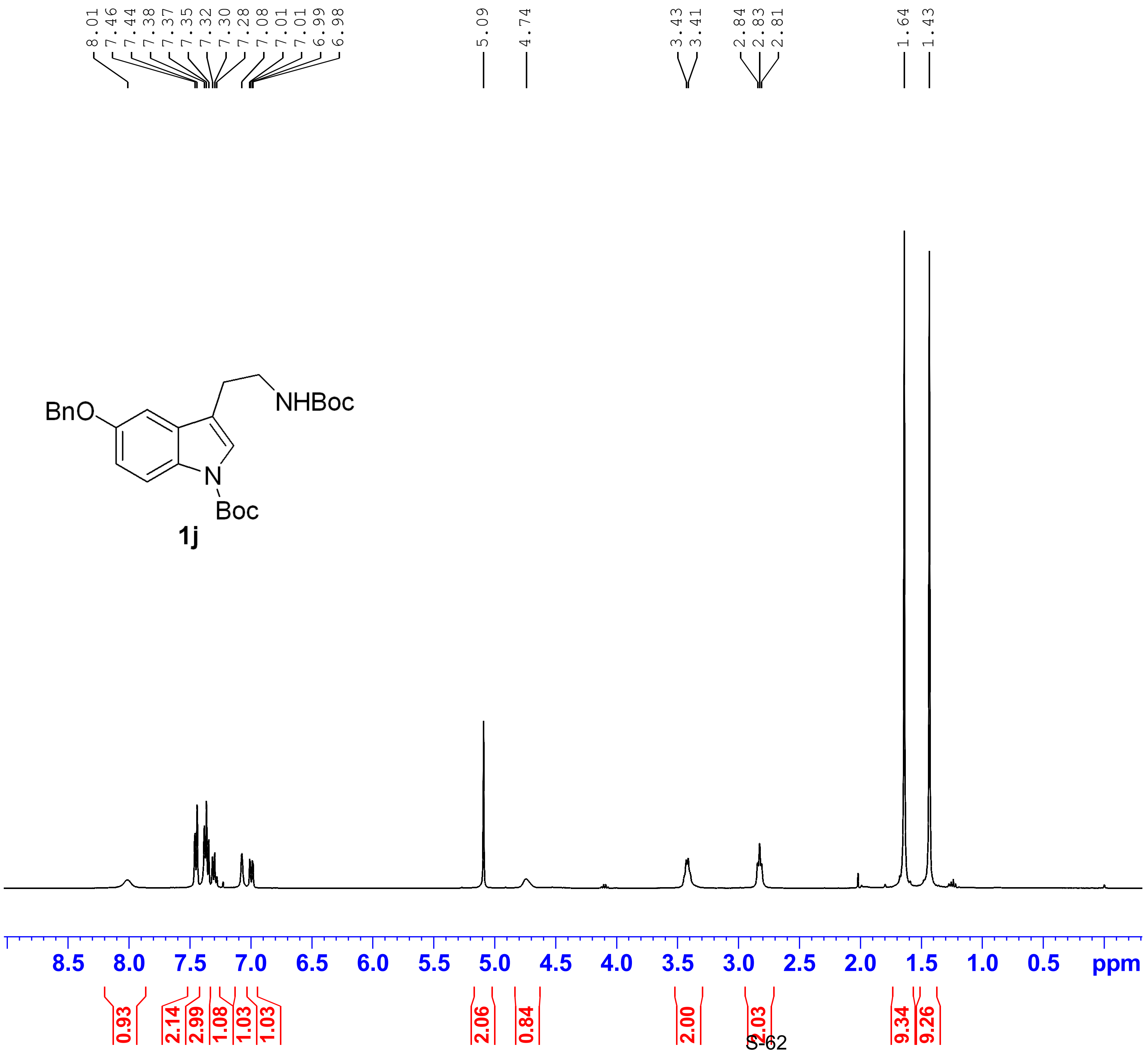

1j

```
Current Data Parameters
NAME          txf-3-110nmr
EXPNO                    1
PROCNO                   1

F2 - Acquisition Parameters
Date_             20220106
Time                 13.18
INSTRUM              spect
PROBHD     5 mm PABBO BB/
PULPROG               zg30
TD                   65536
SOLVENT              CDCl3
NS                       4
DS                       0
SWH            8012.820 Hz
FIDRES         0.122266 Hz
AQ            4.0894465 sec
RG                   17.38
DW              62.400 usec
DE                6.50 usec
TE               295.7 K
D1          1.00000000 sec
TD0                      1

======== CHANNEL f1 ========
SFO1       400.1324710 MHz
NUC1                    1H
P1               14.50 usec
PLW1       11.99499989 W

F2 - Processing parameters
SI                   65536
SF         400.1300220 MHz
WDW                     EM
SSB                      0
LB                 0.30 Hz
GB                       0
PC                    1.00
```


Supplementary Figure 7. ^{1}H NMR spectrum of **1j** (400 MHz, r.t., $CDCl_3$)

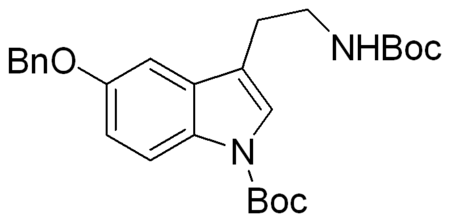

1j

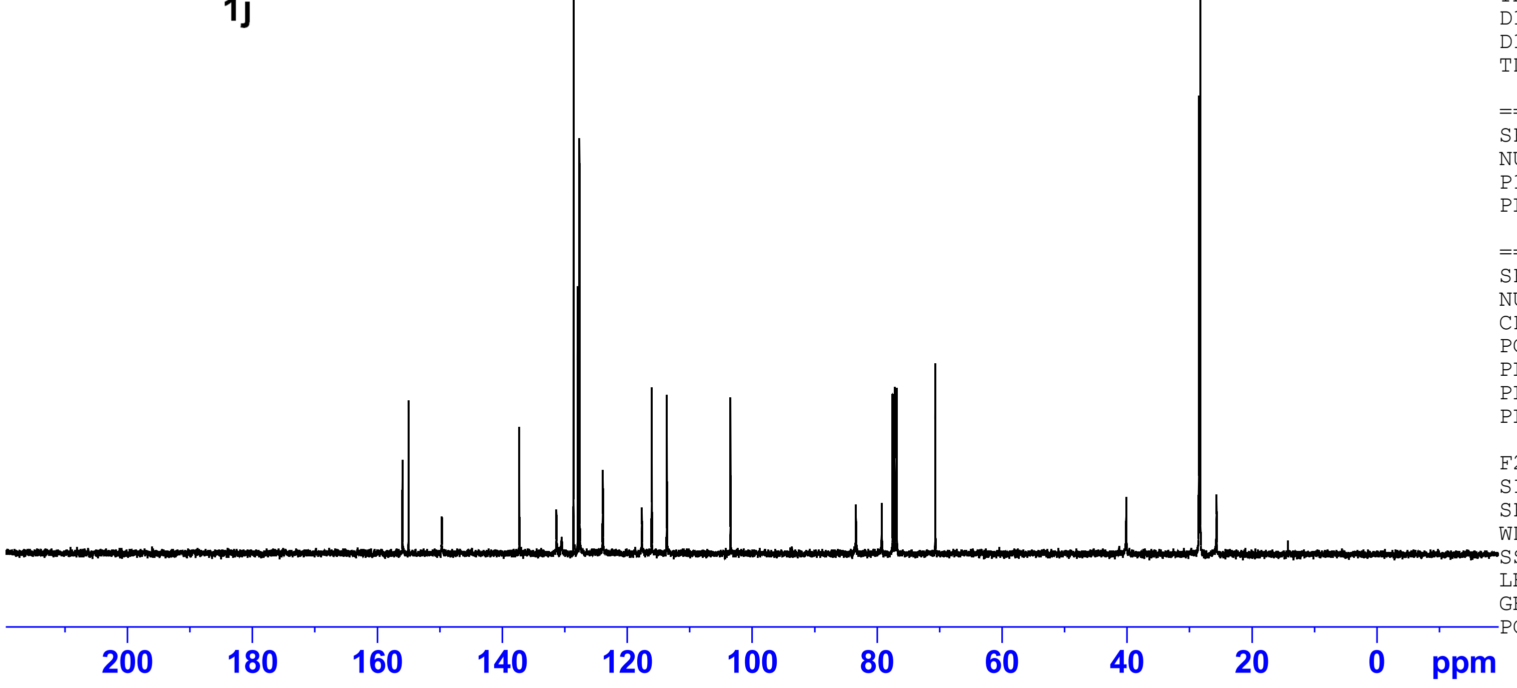

Current Data Parameters
NAME txf-3-110nmr
EXPNO 2
PROCNO 1

F2 - Acquisition Parameters
Date_ 20220106
Time 13.20
INSTRUM spect
PROBHD 5 mm PABBO BB/
PULPROG zgpg30
TD 65536
SOLVENT CDCl3
NS 92
DS 2
SWH 24038.461 Hz
FIDRES 0.366798 Hz
AQ 1.3631488 sec
RG 196.92
DW 20.800 usec
DE 6.50 usec
TE 296.4 K
D1 2.00000000 sec
D11 0.03000000 sec
TD0 1

======== CHANNEL f1 ========
SFO1 100.6228298 MHz
NUC1 13C
P1 9.70 usec
PLW1 46.98899841 W

======== CHANNEL f2 ========
SFO2 400.1316005 MHz
NUC2 1H
CPDPRG[2 waltz16
PCPD2 90.00 usec
PLW2 11.99499989 W
PLW12 0.34213999 W
PLW13 0.27713001 W

F2 - Processing parameters
SI 32768
SF 100.6127690 MHz
WDW EM
SSB 0
LB 1.00 Hz
GB 0
PC 1.40

Supplementary Figure 8. ^{13}C NMR spectrum of **1j** (100 MHz, r.t., $CDCl_3$)

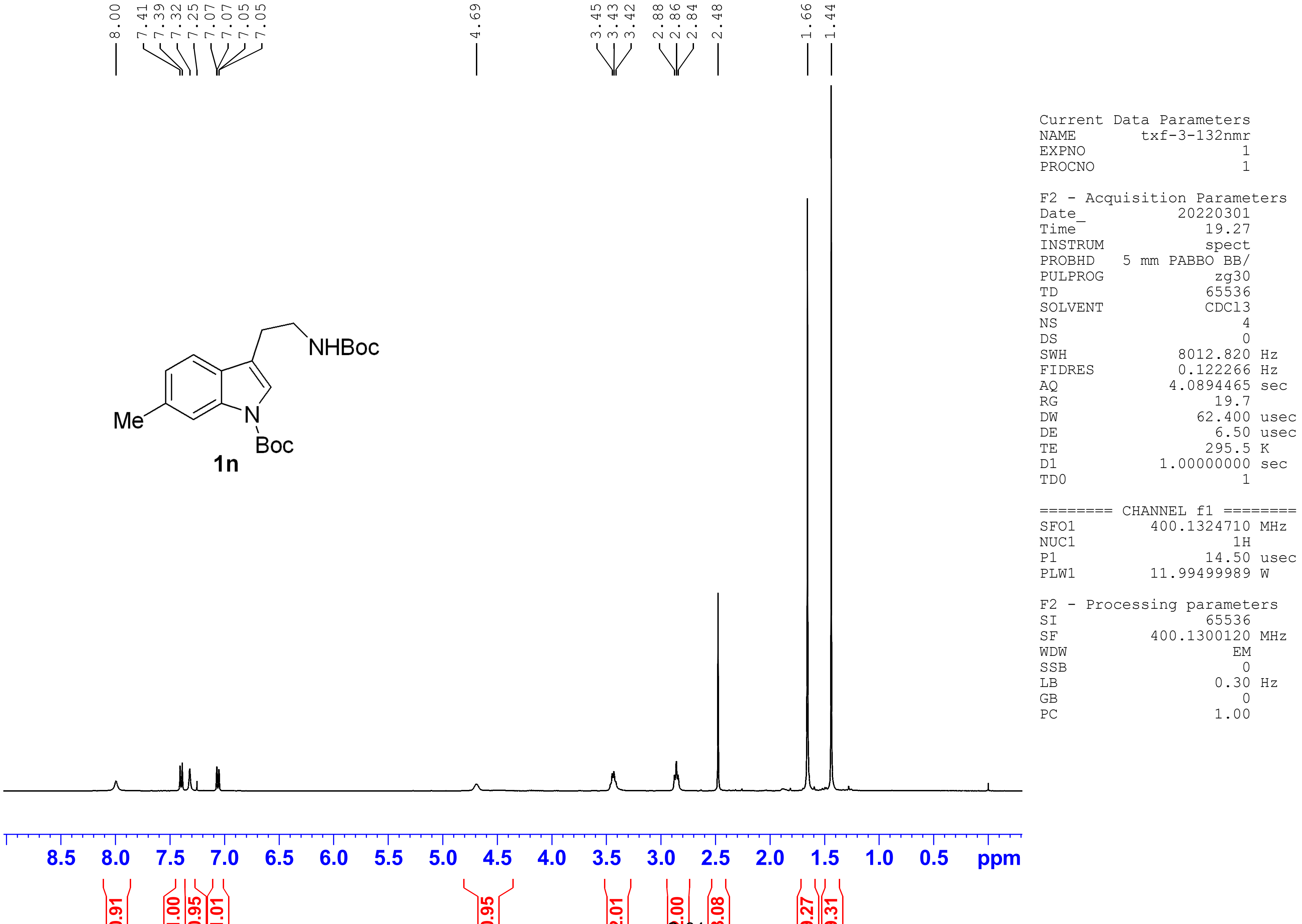

Supplementary Figure 9. ^{1}H NMR spectrum of **1n** (400 MHz, r.t., $CDCl_3$)

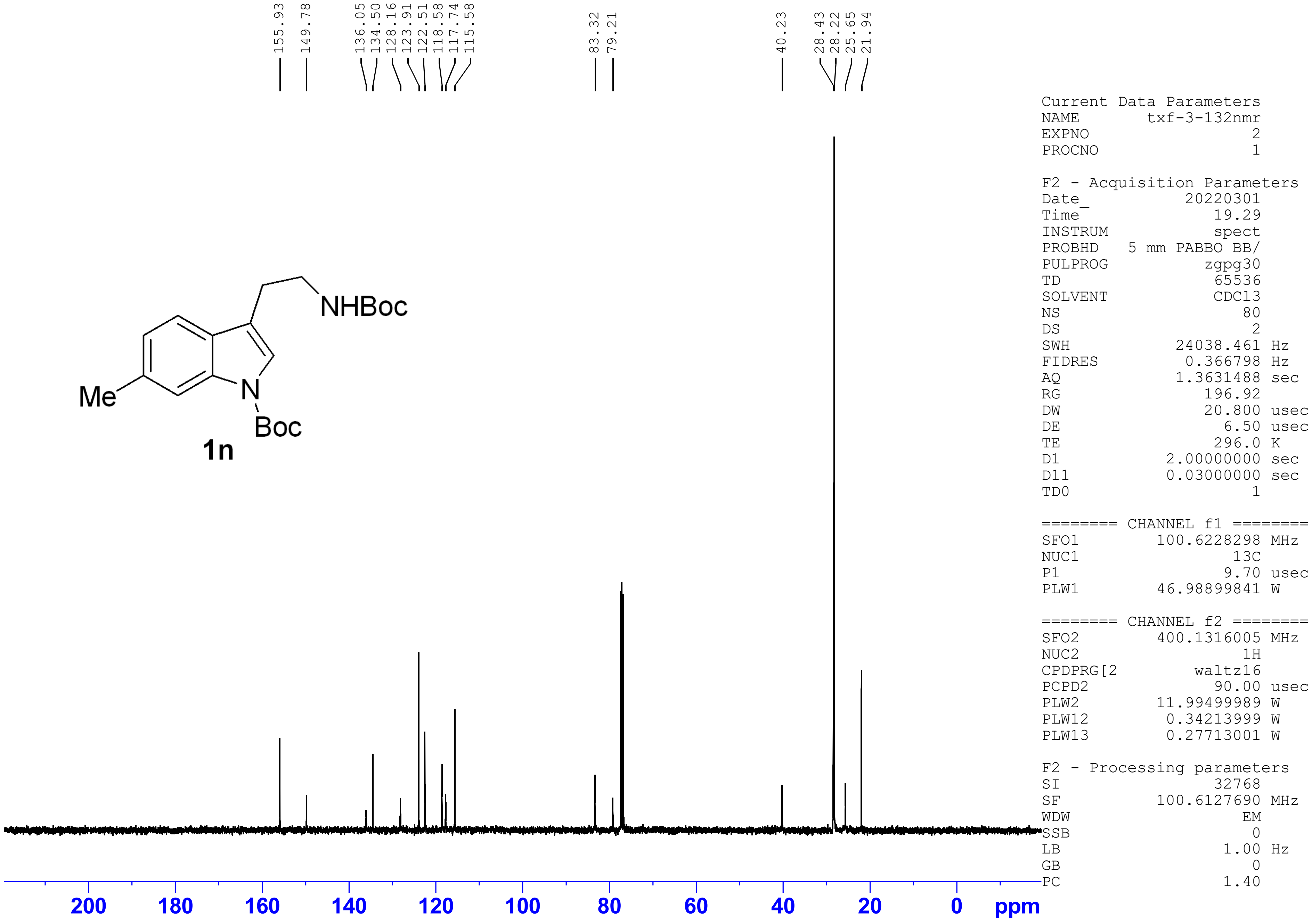

Supplementary Figure 10. ^{13}C NMR spectrum of **1e** (100 MHz, r.t., $CDCl_3$)

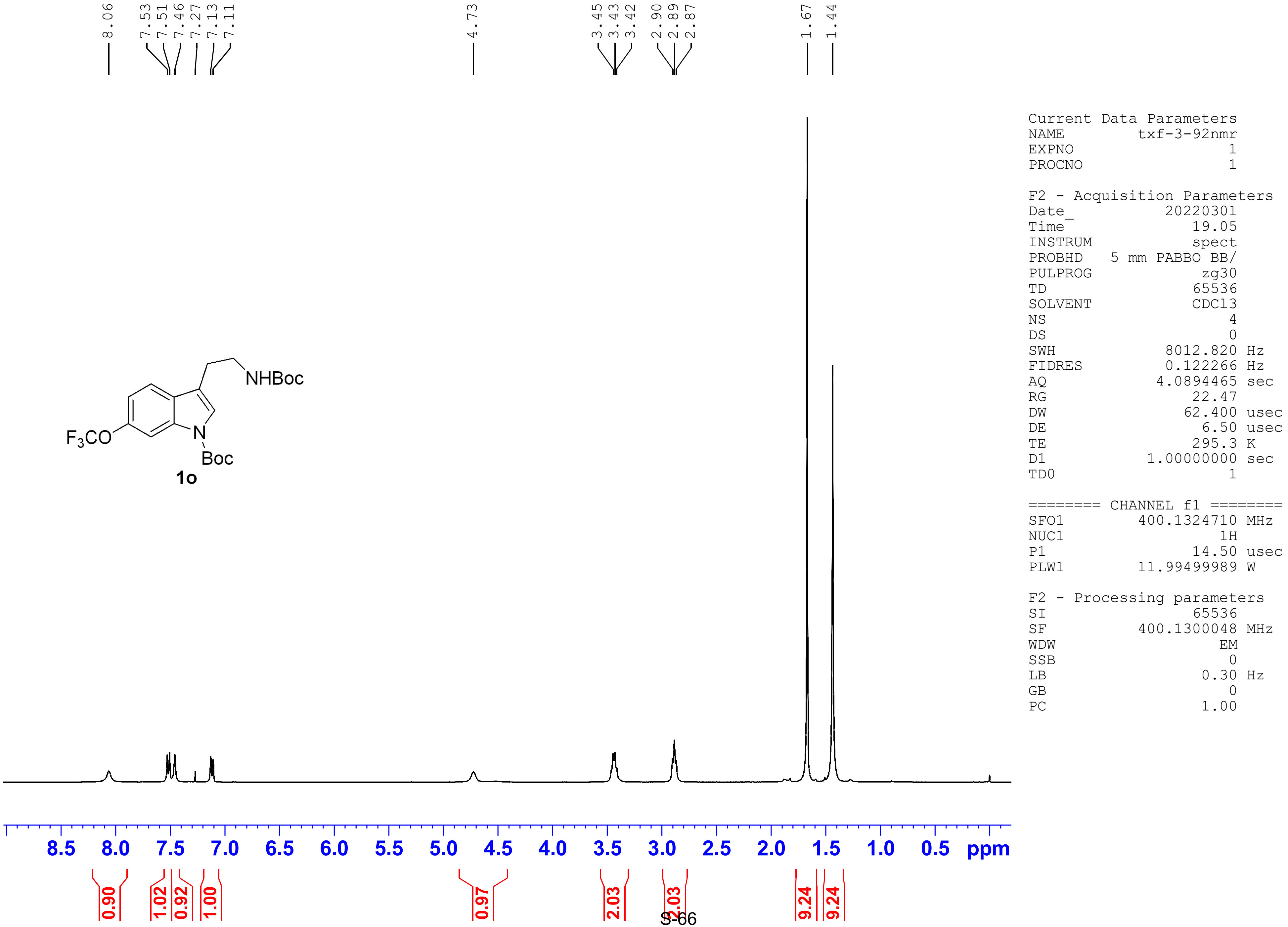

Supplementary Figure 11. ^{1}H NMR spectrum of **1o** (400 MHz, r.t., $CDCl_3$)

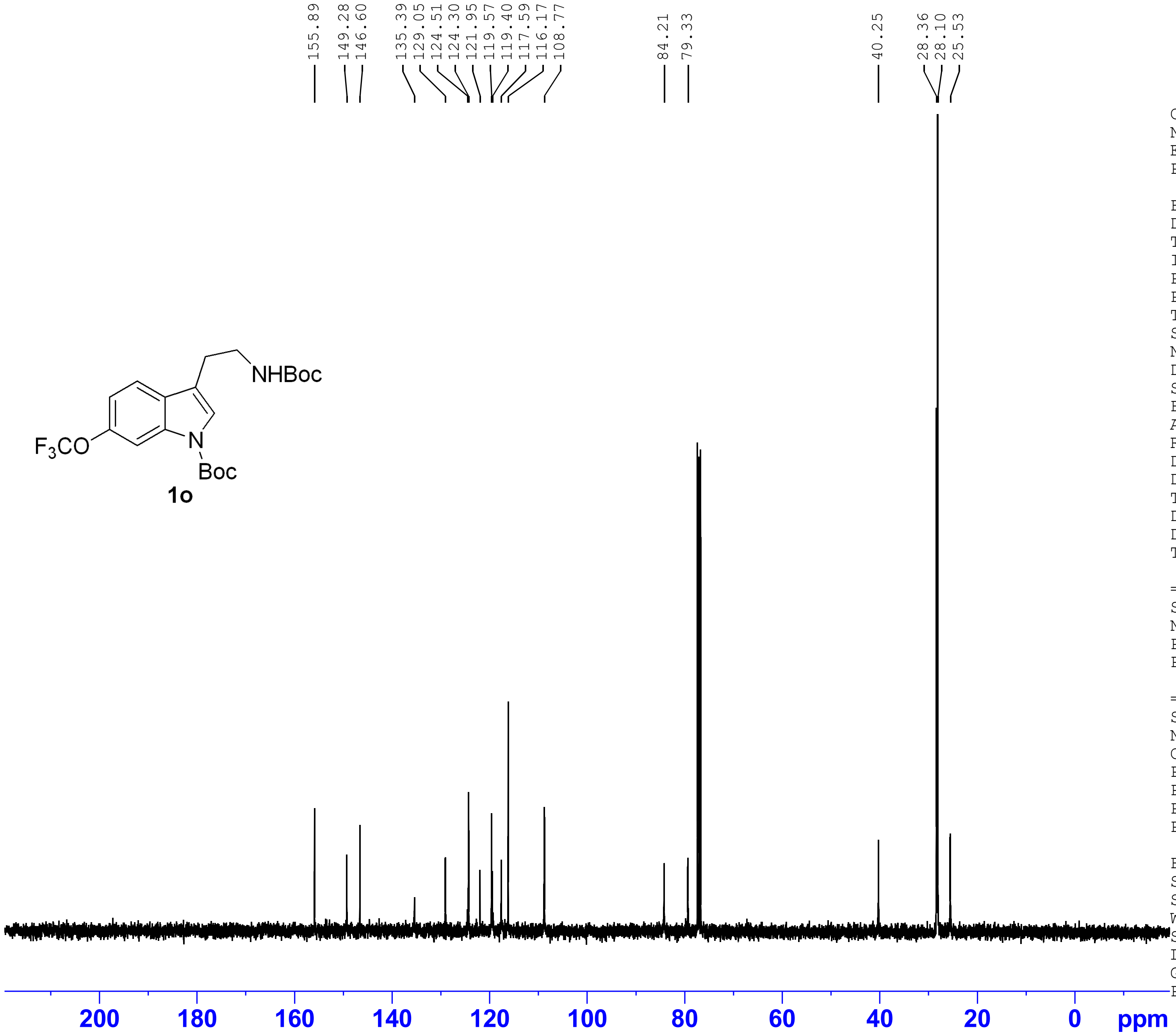

Current Data Parameters
NAME txf-3-92nmr
EXPNO 2
PROCNO 1

F2 - Acquisition Parameters
Date_ 20220301
Time 19.06
INSTRUM spect
PROBHD 5 mm PABBO BB/
PULPROG zgpg30
TD 65536
SOLVENT CDCl3
NS 72
DS 2
SWH 24038.461 Hz
FIDRES 0.366798 Hz
AQ 1.3631488 sec
RG 196.92
DW 20.800 usec
DE 6.50 usec
TE 295.8 K
D1 2.00000000 sec
D11 0.03000000 sec
TD0 1

======== CHANNEL f1 ========
SFO1 100.6228298 MHz
NUC1 13C
P1 9.70 usec
PLW1 46.98899841 W

======== CHANNEL f2 ========
SFO2 400.1316005 MHz
NUC2 1H
CPDPRG[2 waltz16
PCPD2 90.00 usec
PLW2 11.99499989 W
PLW12 0.34213999 W
PLW13 0.27713001 W

F2 - Processing parameters
SI 32768
SF 100.6127690 MHz
WDW EM
SSB 0
LB 1.00 Hz
GB 0
PC 1.40

Supplementary Figure 12. ^{13}C NMR spectrum of **1o** (100 MHz, r.t., $CDCl_3$)

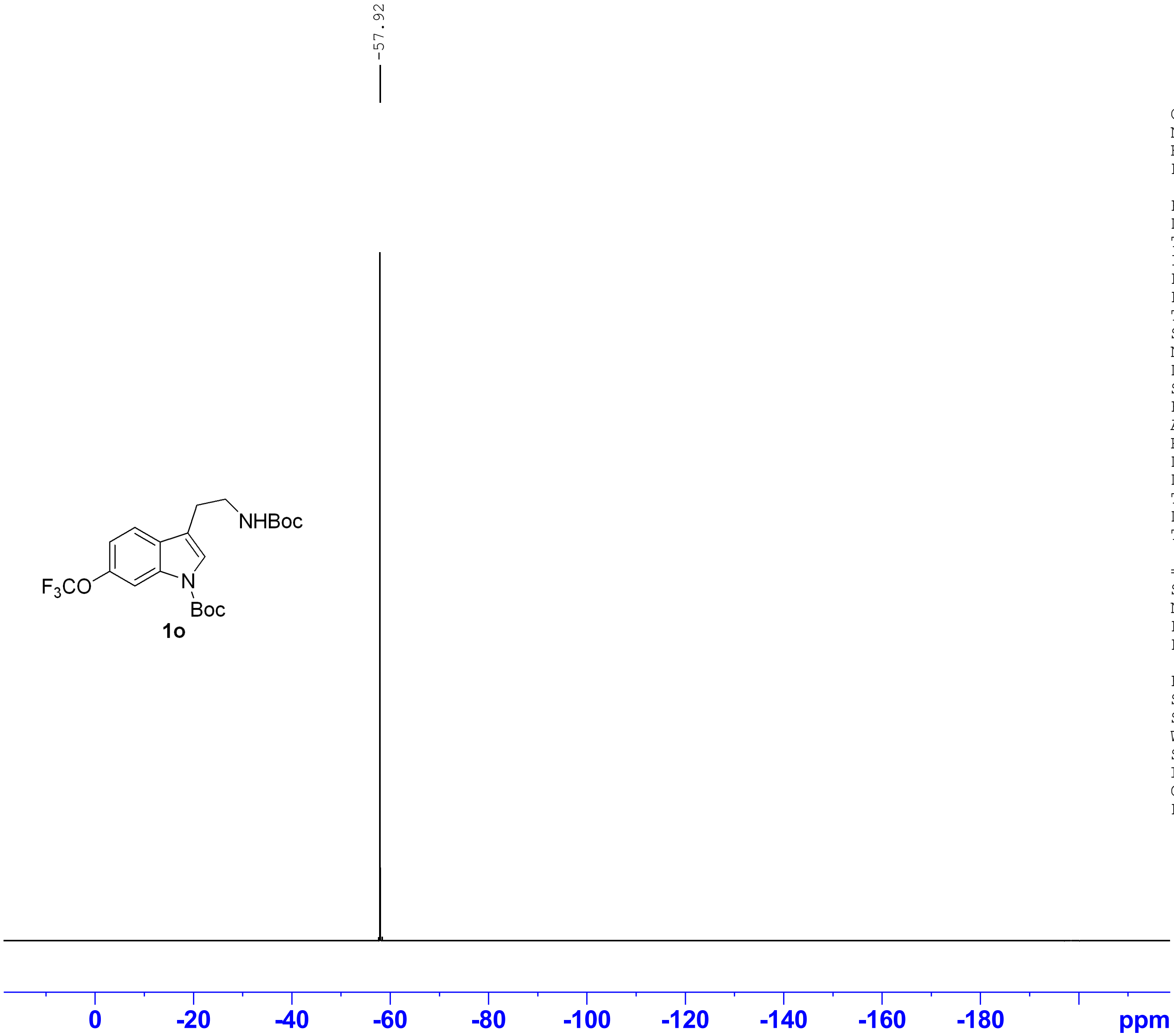

1o

Current Data Parameters

NAME	txf-3-92nmr
EXPNO	3
PROCNO	1

F2 - Acquisition Parameters

Date_	20220301
Time	19.35
INSTRUM	spect
PROBHD	5 mm PABBO BB/
PULPROG	zgflqn
TD	131072
SOLVENT	CDCl3
NS	16
DS	4
SWH	89285.711 Hz
FIDRES	0.681196 Hz
AQ	0.7340032 sec
RG	196.92
DW	5.600 usec
DE	6.50 usec
TE	295.5 K
D1	1.00000000 sec
TD0	1

======== CHANNEL f1 ========

SFO1	376.4607164 MHz
NUC1	19F
P1	14.70 usec
PLW1	15.99600029 W

F2 - Processing parameters

SI	65536
SF	376.4983660 MHz
WDW	EM
SSB	0
LB	0.30 Hz
GB	0
PC	1.00

Supplementary Figure 13. ^{19}F NMR spectrum of 1o (376 MHz, r.t., $CDCl_3$)

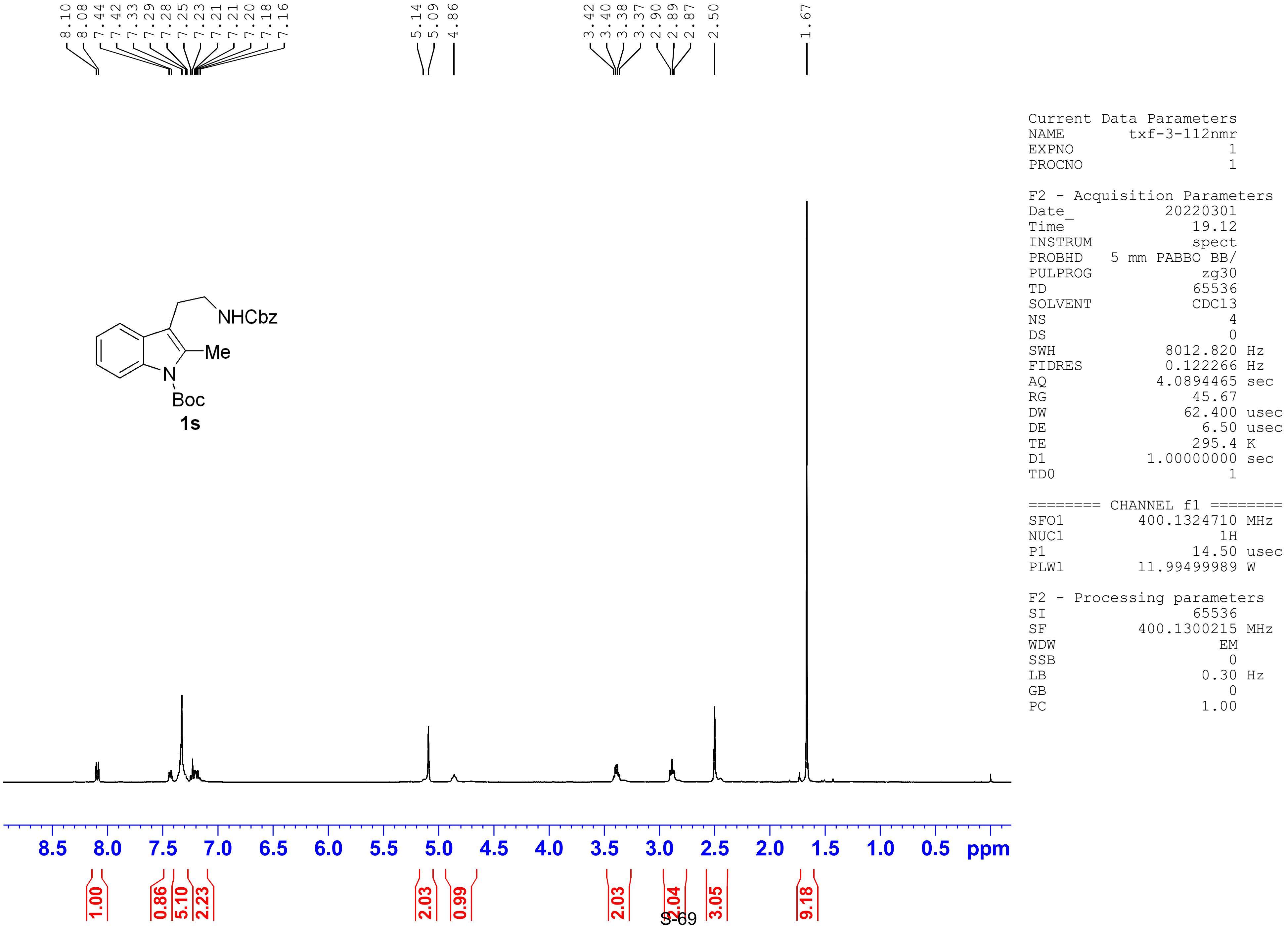

Supplementary Figure 14. ^{1}H NMR spectrum of **1s** (400 MHz, r.t., $CDCl_3$)

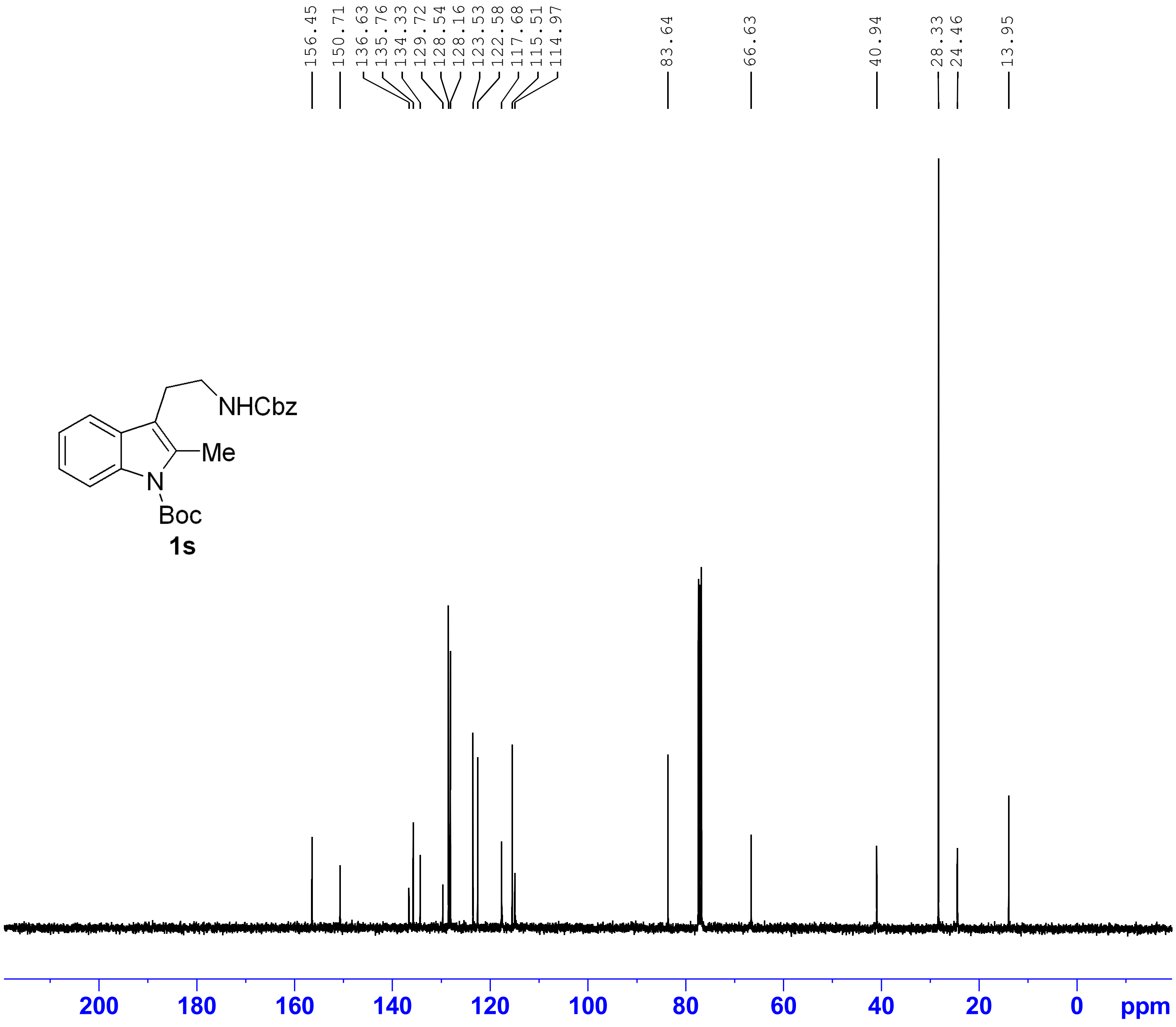

1s

Current Data Parameters
NAME txf-3-112nmr
EXPNO 2
PROCNO 1

F2 - Acquisition Parameters
Date_ 20220301
Time 19.14
INSTRUM spect
PROBHD 5 mm PABBO BB/
PULPROG zgpg30
TD 65536
SOLVENT CDCl3
NS 88
DS 2
SWH 24038.461 Hz
FIDRES 0.366798 Hz
AQ 1.3631488 sec
RG 196.92
DW 20.800 usec
DE 6.50 usec
TE 295.9 K
D1 2.00000000 sec
D11 0.03000000 sec
TD0 1

======== CHANNEL f1 ========
SFO1 100.6228298 MHz
NUC1 13C
P1 9.70 usec
PLW1 46.98899841 W

======== CHANNEL f2 ========
SFO2 400.1316005 MHz
NUC2 1H
CPDPRG[2 waltz16
PCPD2 90.00 usec
PLW2 11.99499989 W
PLW12 0.34213999 W
PLW13 0.27713001 W

F2 - Processing parameters
SI 32768
SF 100.6127690 MHz
WDW EM
SSB 0
LB 1.00 Hz
GB 0
PC 1.40

Supplementary Figure 15. ^{13}C NMR spectrum of **1s** (100 MHz, r.t., CDCl3)

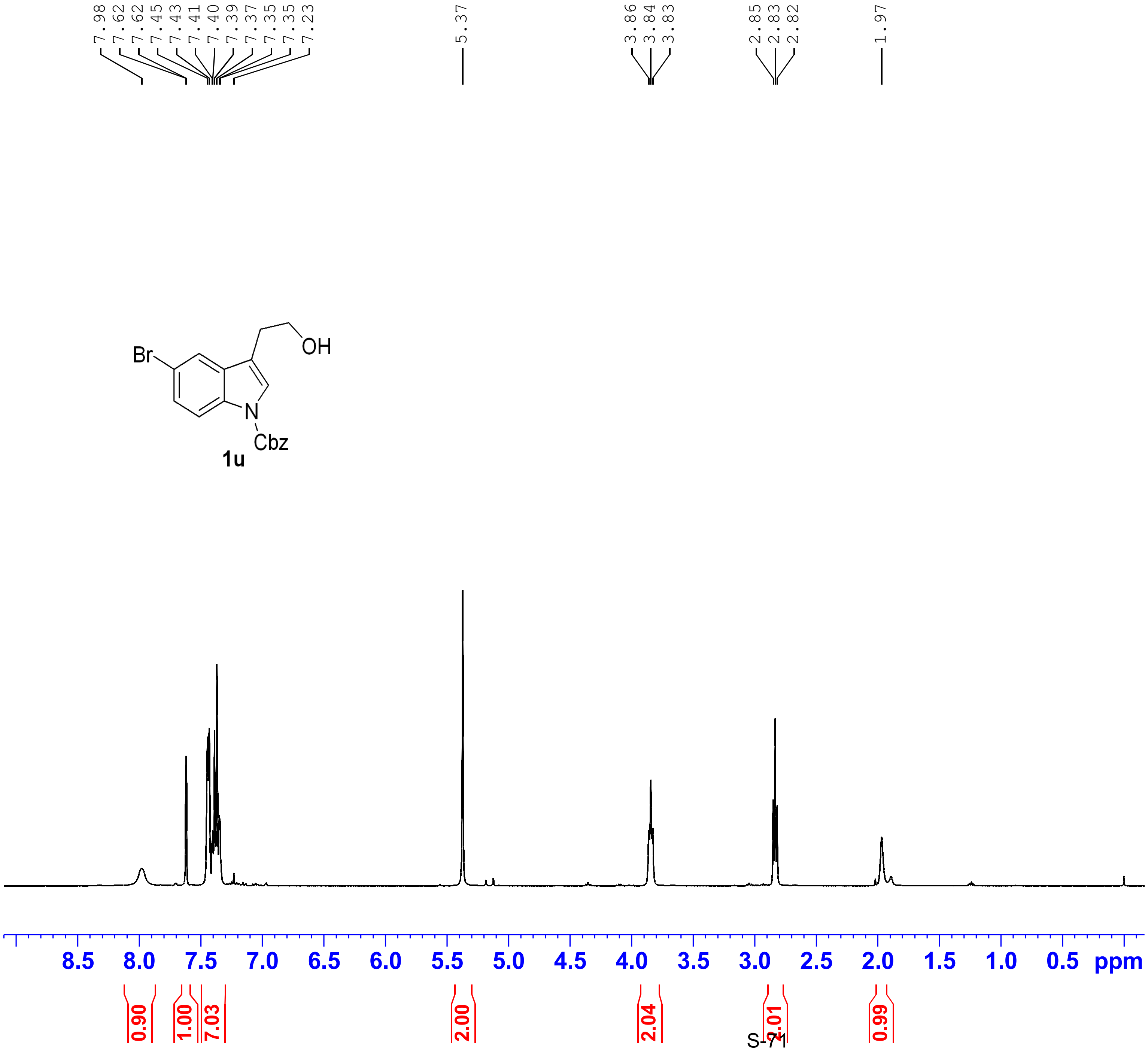


```
Current Data Parameters
NAME        txf-3-116nmr
EXPNO                  1
PROCNO                 1

F2 - Acquisition Parameters
Date_           20220106
Time               20.48
INSTRUM            spect
PROBHD    5 mm PABBO BB/
PULPROG             zg30
TD                 65536
SOLVENT            CDCl3
NS                     4
DS                     0
SWH             8012.820 Hz
FIDRES          0.122266 Hz
AQ             4.0894465 sec
RG                 82.92
DW                62.400 usec
DE                  6.50 usec
TE                 295.4 K
D1            1.00000000 sec
TD0                    1

======== CHANNEL f1 ========
SFO1         400.1324710 MHz
NUC1                  1H
P1                 14.50 usec
PLW1         11.99499989 W

F2 - Processing parameters
SI                 65536
SF           400.1300207 MHz
WDW                   EM
SSB                    0
LB                  0.30 Hz
GB                     0
PC                  1.00
```


Supplementary Figure 16. ^{1}H NMR spectrum of **1u** (400 MHz, r.t., CDCl3)

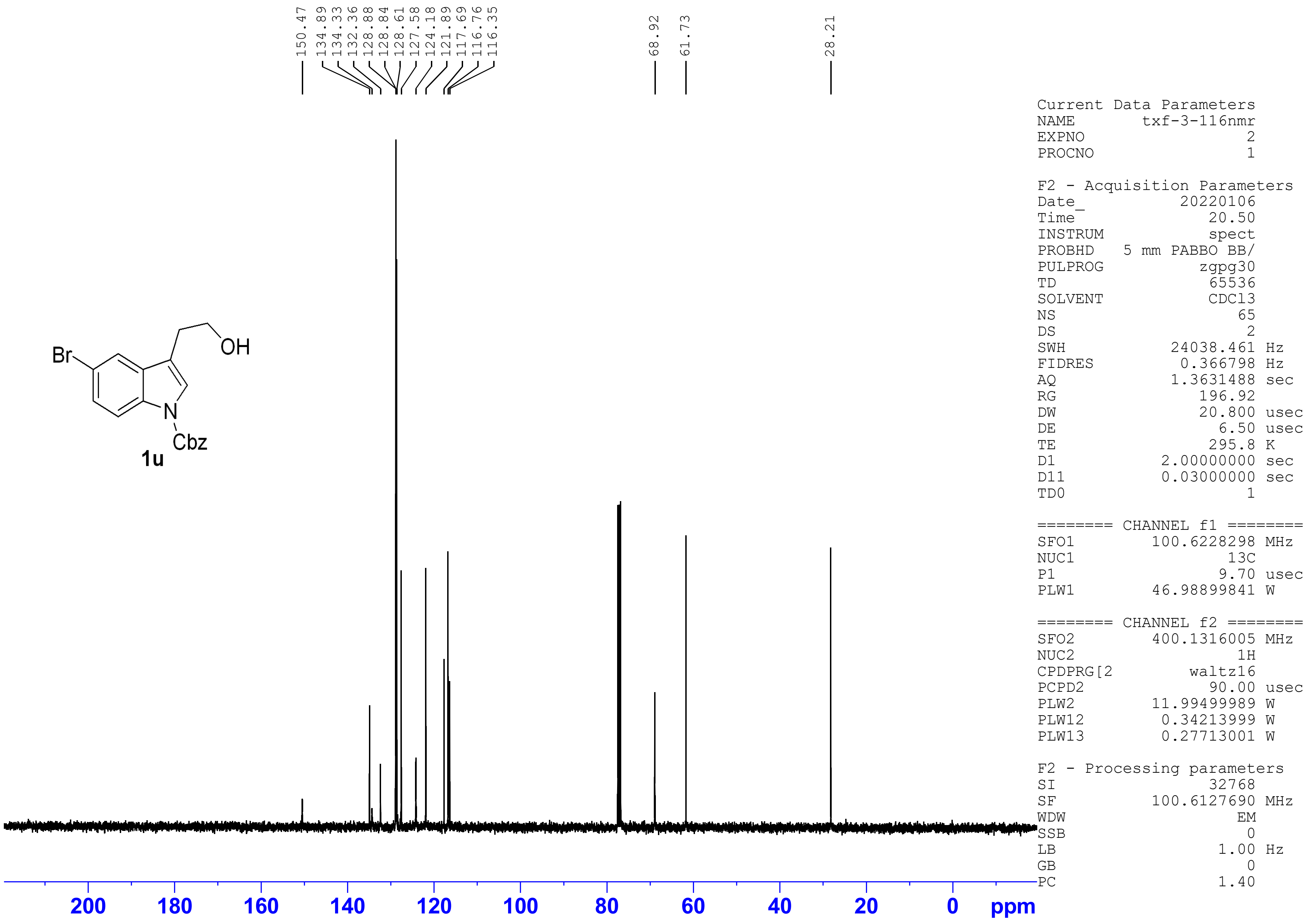

Supplementary Figure 17. ^{13}C NMR spectrum of **1u** (100 MHz, r.t., $CDCl_3$)

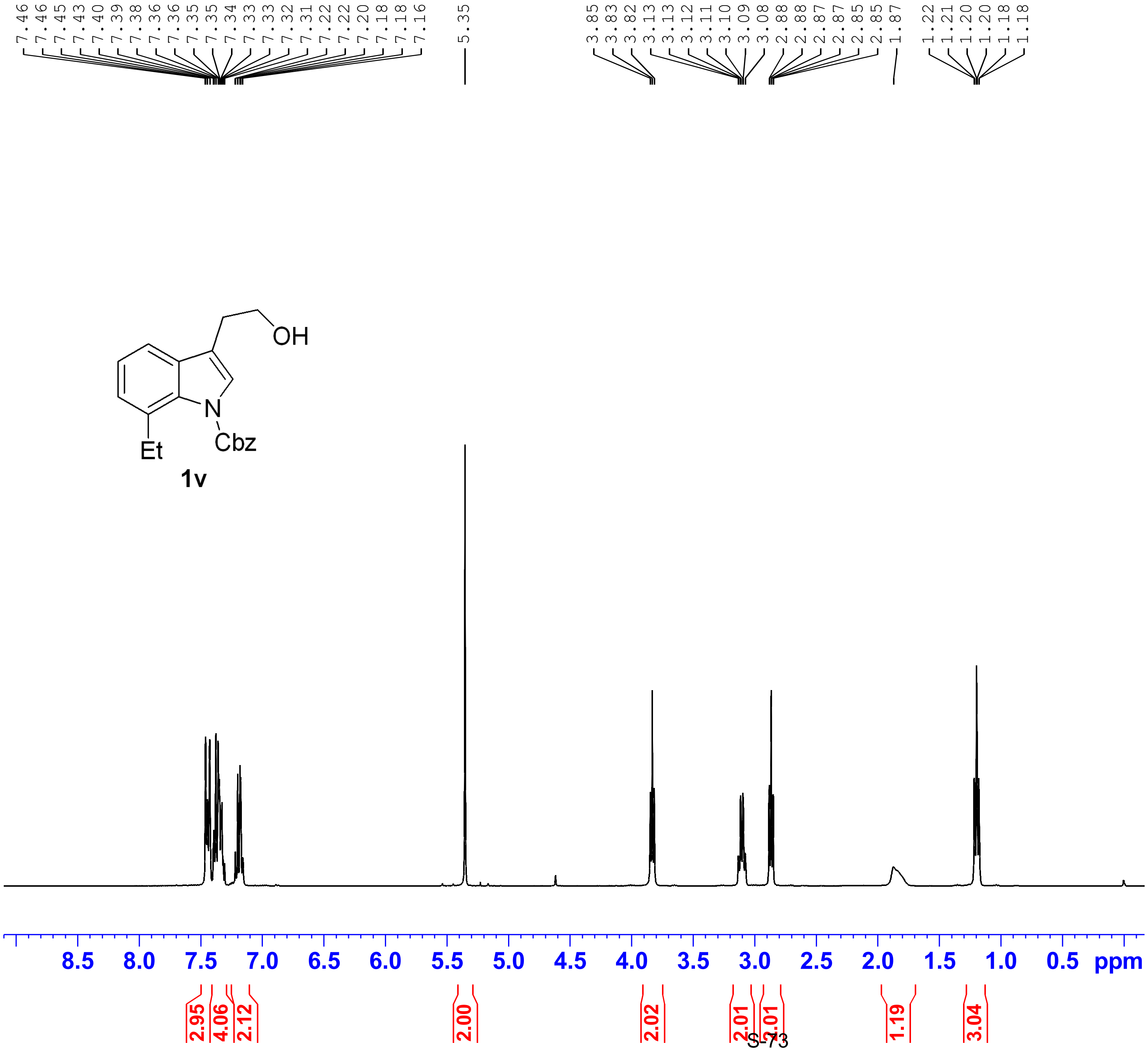


```
Current Data Parameters
NAME          txf-3-117nmr
EXPNO                    1
PROCNO                   1

F2 - Acquisition Parameters
Date_             20220301
Time                 19.20
INSTRUM              spect
PROBHD    5 mm PABBO BB/
PULPROG               zg30
TD                   65536
SOLVENT              CDCl3
NS                       4
DS                       0
SWH            8012.820 Hz
FIDRES         0.122266 Hz
AQ            4.0894465 sec
RG                   22.47
DW              62.400 usec
DE                6.50 usec
TE               295.5 K
D1          1.00000000 sec
TD0                      1

======== CHANNEL f1 ========
SFO1         400.1324710 MHz
NUC1                    1H
P1               14.50 usec
PLW1         11.99499989 W

F2 - Processing parameters
SI                   65536
SF           400.1300335 MHz
WDW                     EM
SSB                      0
LB                0.30 Hz
GB                       0
PC                    1.00
```


Supplementary Figure 18. ^{1}H NMR spectrum of **1v** (400 MHz, r.t., $CDCl_3$)

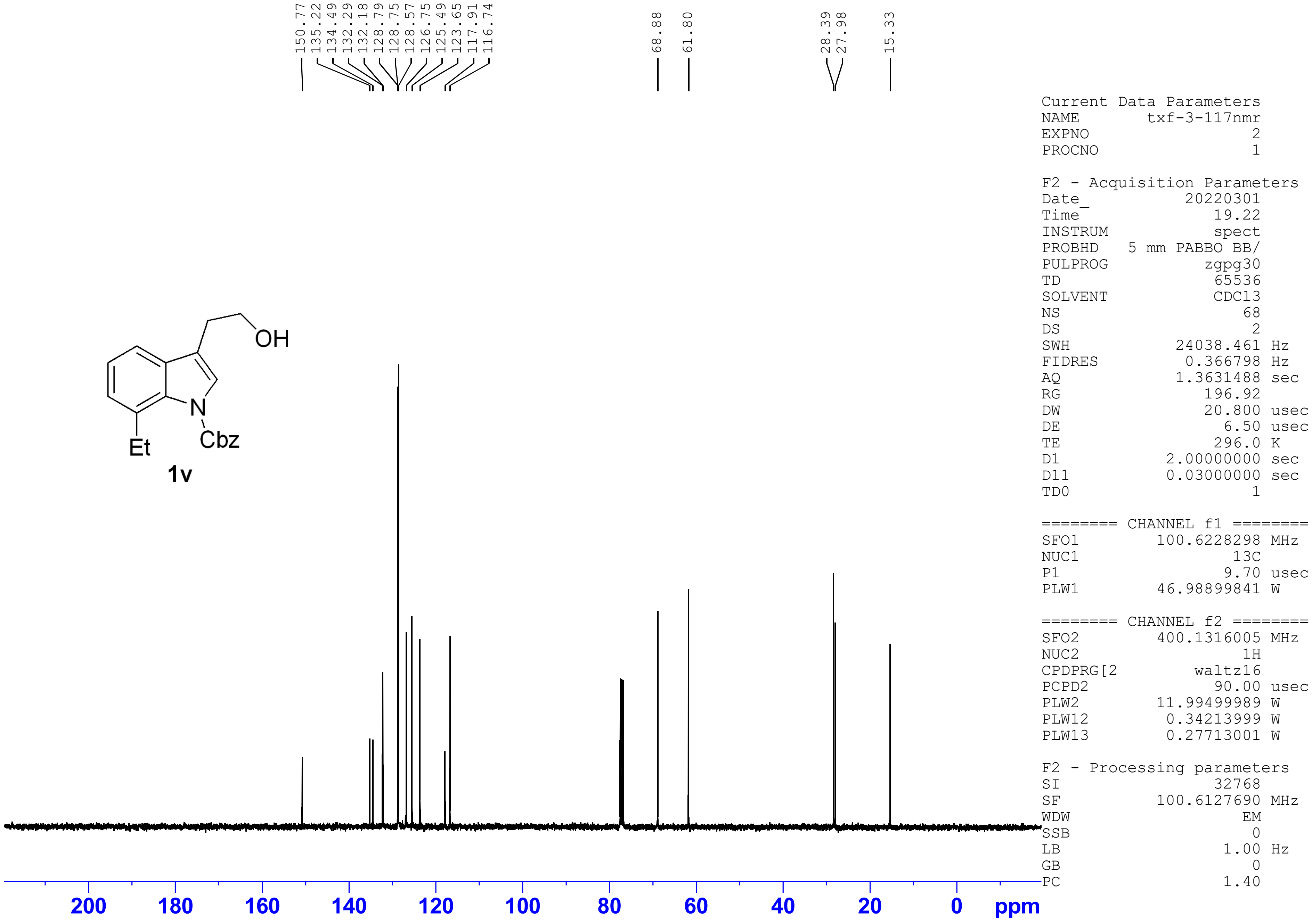

Supplementary Figure 19. ^{13}C NMR spectrum of **1v** (100 MHz, r.t., $CDCl_3$)

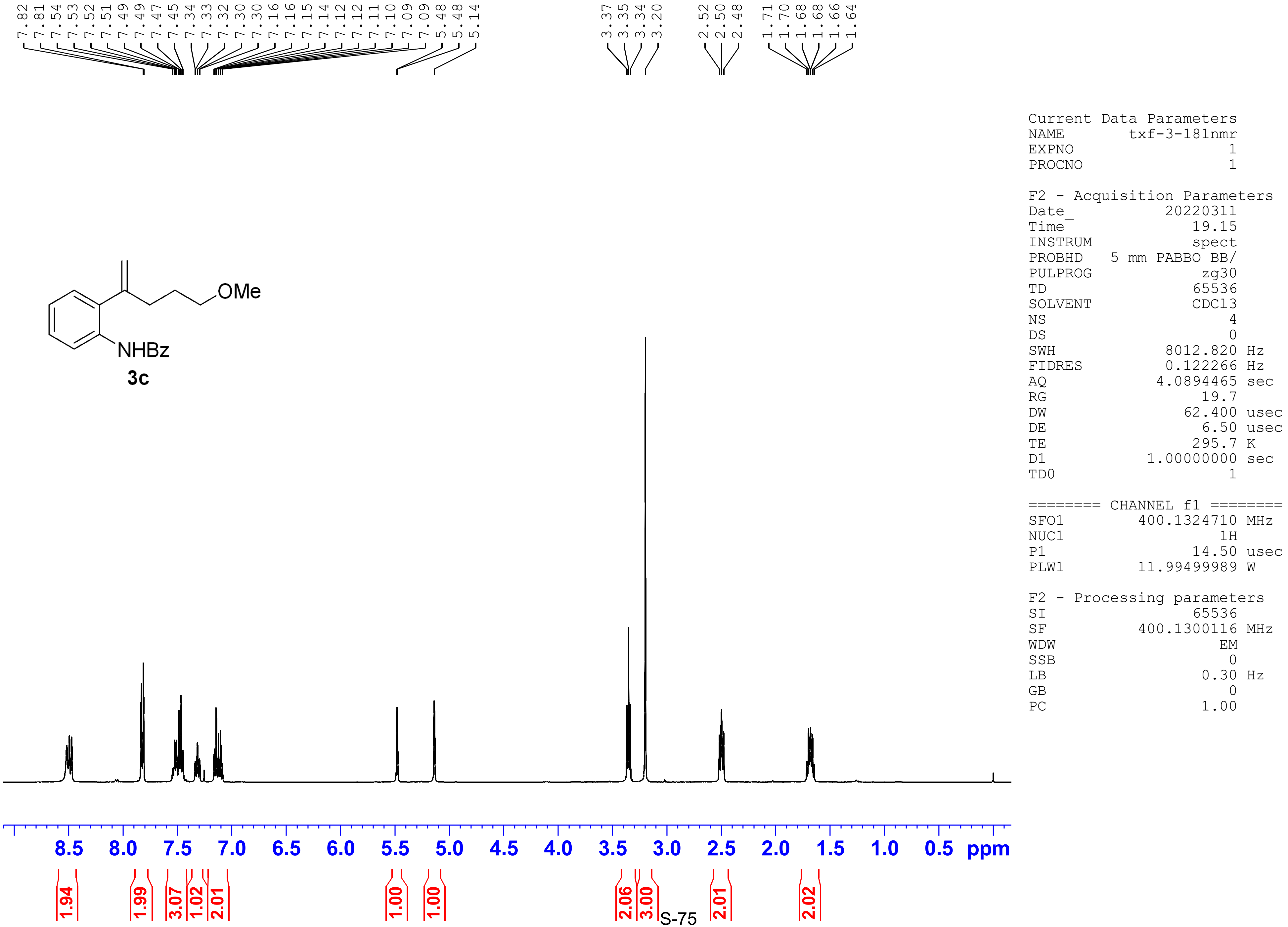

3c

Supplementary Figure 20. ^{1}H NMR spectrum of **3c** (400 MHz, r.t., $CDCl_3$)

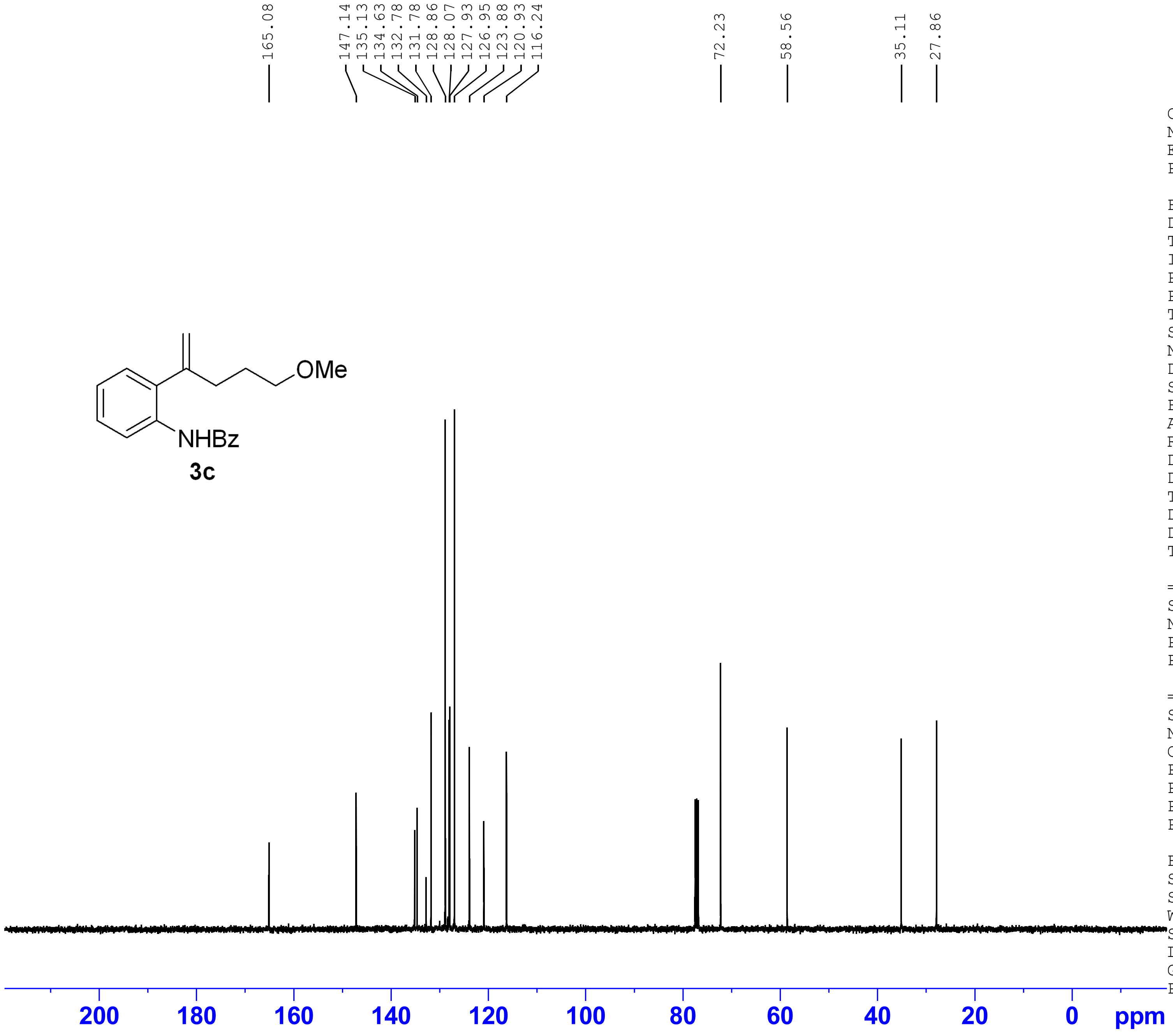

3c

Current Data Parameters
NAME txf-3-181nmr
EXPNO 2
PROCNO 1

F2 - Acquisition Parameters
Date_ 20220311
Time 19.17
INSTRUM spect
PROBHD 5 mm PABBO BB/
PULPROG zgpg30
TD 65536
SOLVENT CDCl3
NS 54
DS 2
SWH 24038.461 Hz
FIDRES 0.366798 Hz
AQ 1.3631488 sec
RG 196.92
DW 20.800 usec
DE 6.50 usec
TE 296.2 K
D1 2.00000000 sec
D11 0.03000000 sec
TD0 1

======== CHANNEL f1 ========
SFO1 100.6228298 MHz
NUC1 13C
P1 9.70 usec
PLW1 46.98899841 W

======== CHANNEL f2 ========
SFO2 400.1316005 MHz
NUC2 1H
CPDPRG[2 waltz16
PCPD2 90.00 usec
PLW2 11.99499989 W
PLW12 0.34213999 W
PLW13 0.27713001 W

F2 - Processing parameters
SI 32768
SF 100.6127690 MHz
WDW EM
SSB 0
LB 1.00 Hz
GB 0
PC 1.40

Supplementary Figure 21. ^{13}C NMR spectrum of **3c** (100 MHz, r.t., $CDCl_3$)

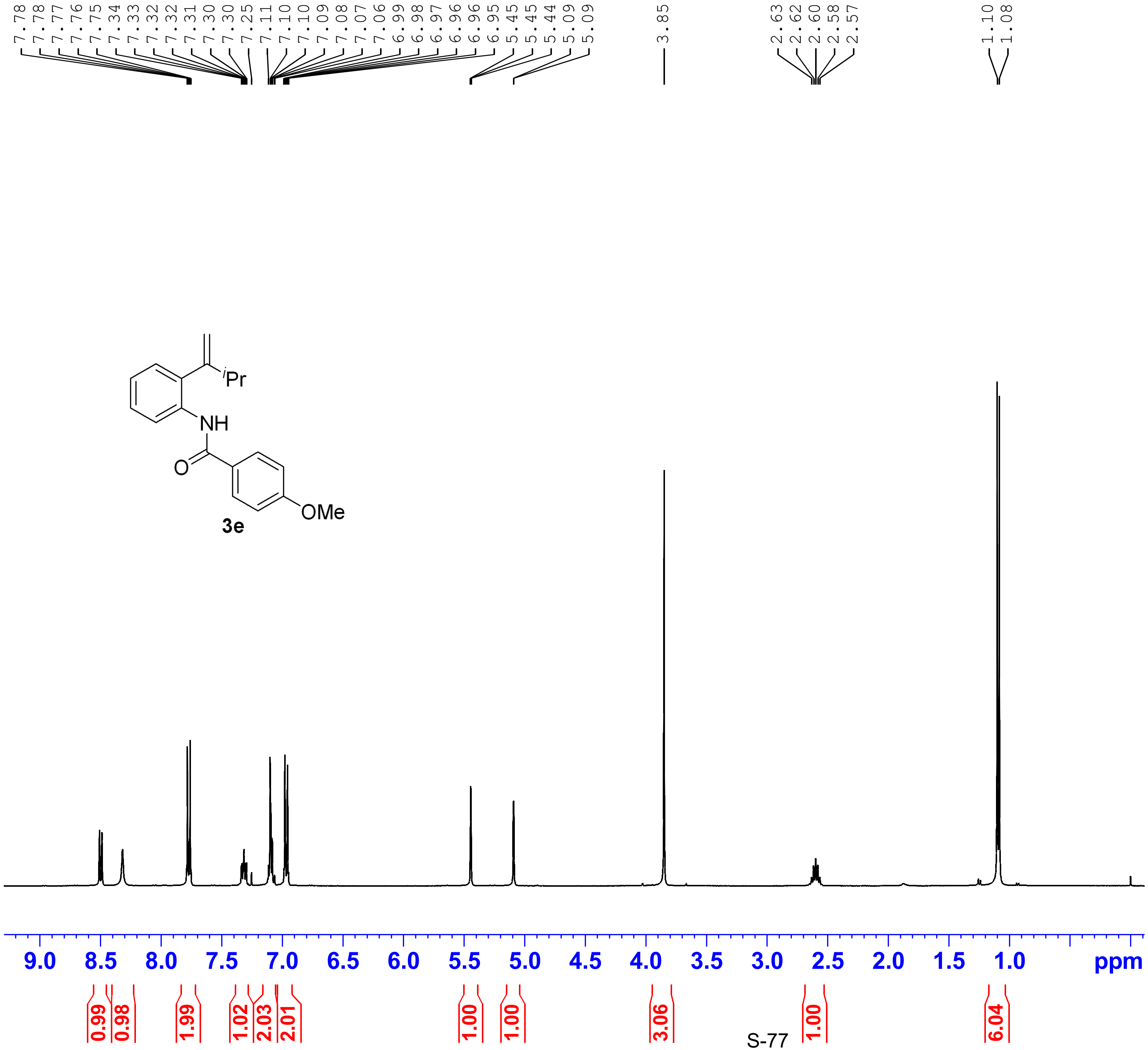

3e

```
Current Data Parameters
NAME        txf-3-182nmr
EXPNO                  1
PROCNO                 1

F2 - Acquisition Parameters
Date_           20220311
Time               19.22
INSTRUM            spect
PROBHD    5 mm PABBO BB/
PULPROG             zg30
TD                 65536
SOLVENT            CDCl3
NS                     4
DS                     0
SWH             8012.820 Hz
FIDRES          0.122266 Hz
AQ             4.0894465 sec
RG                 25.32
DW                62.400 usec
DE                  6.50 usec
TE                 295.7 K
D1            1.00000000 sec
TD0                    1

======== CHANNEL f1 ========
SFO1         400.1324710 MHz
NUC1                  1H
P1                 14.50 usec
PLW1         11.99499989 W

F2 - Processing parameters
SI                 65536
SF           400.1300119 MHz
WDW                   EM
SSB                    0
LB                  0.30 Hz
GB                     0
PC                  1.00
```


Supplementary Figure 22. ^{1}H NMR spectrum of **3e** (400 MHz, r.t., $CDCl_3$)

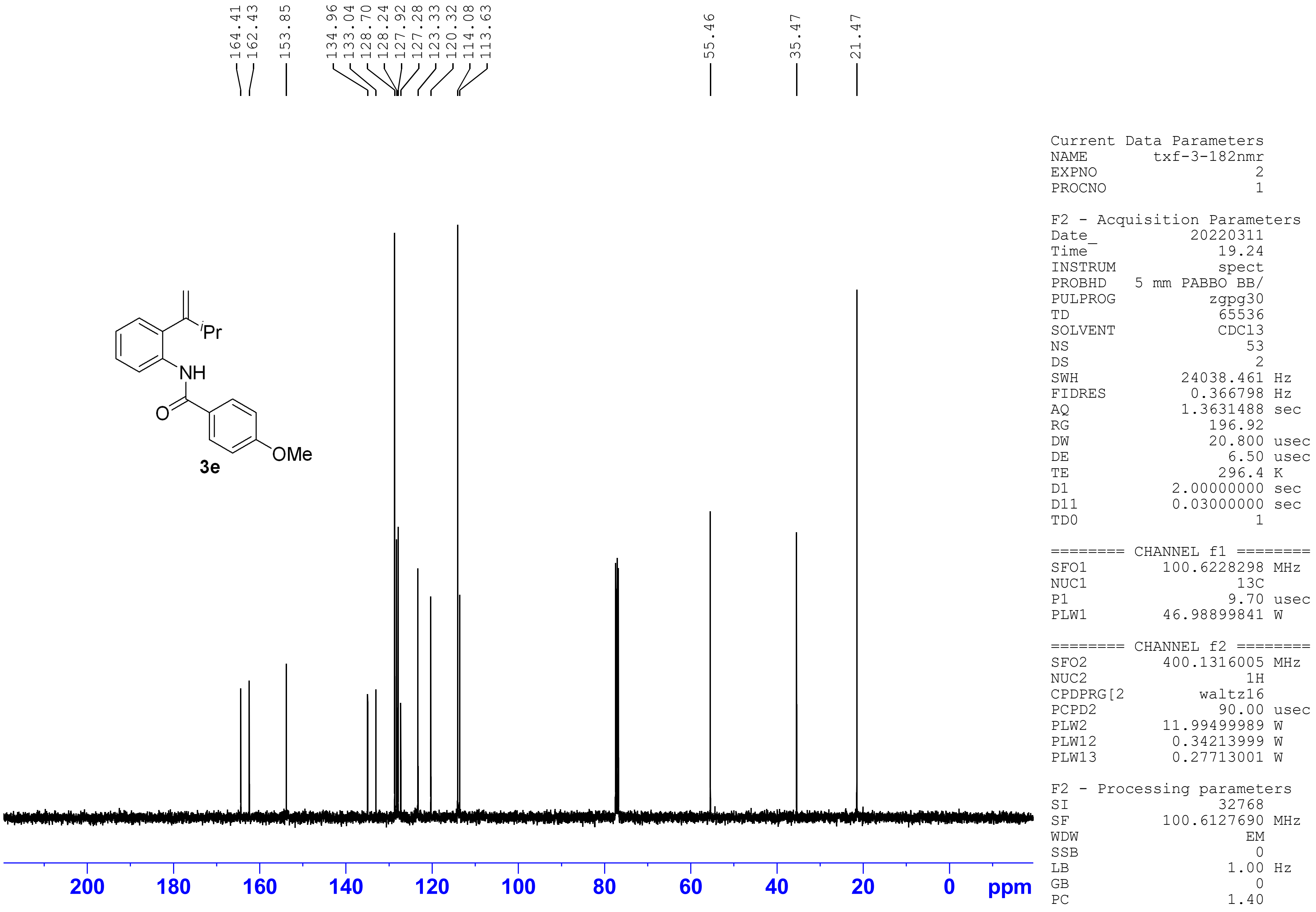

Supplementary Figure 23. ^{13}C NMR spectrum of **3e** (100 MHz, r.t., $CDCl_3$)

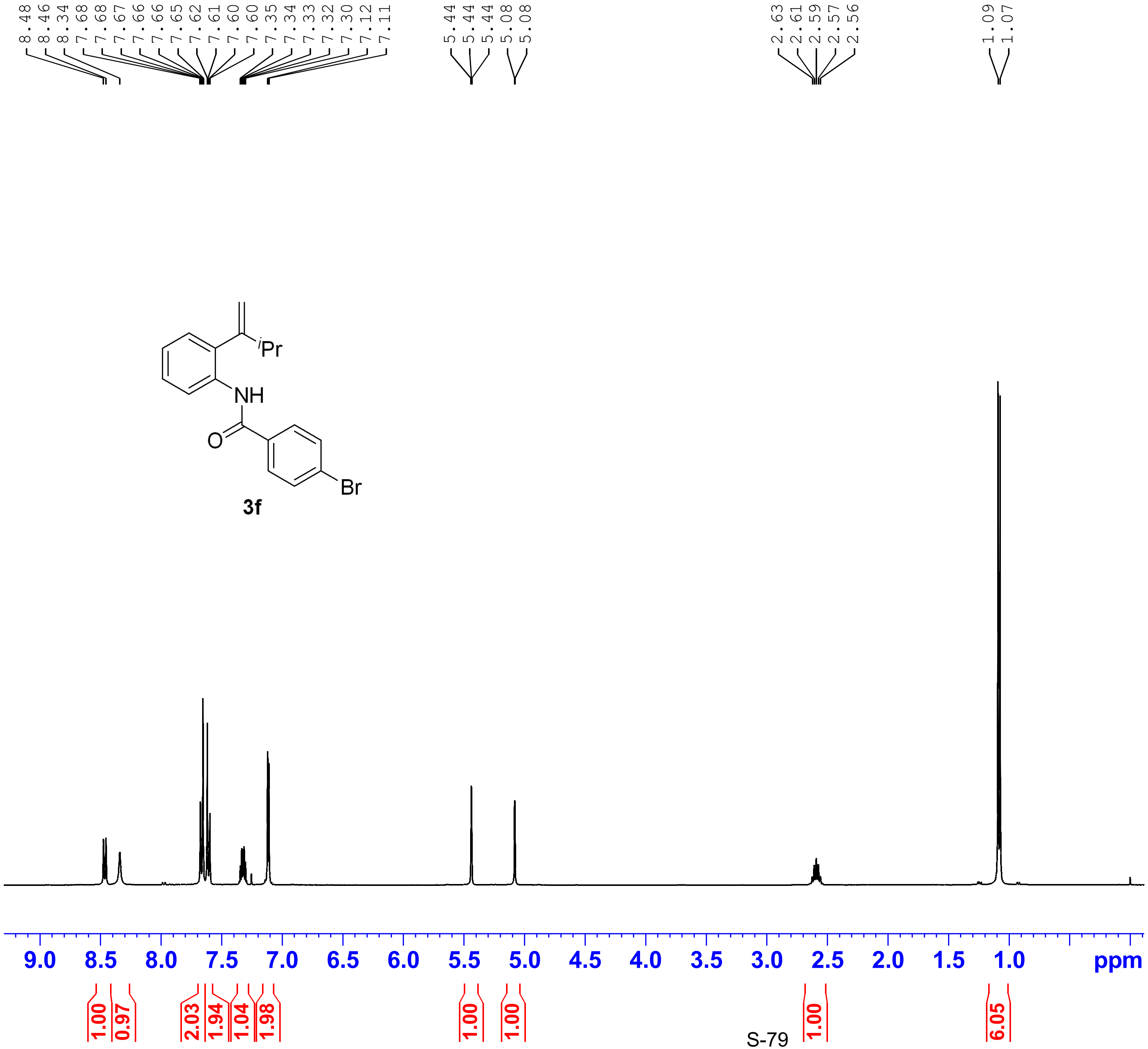


```
Current Data Parameters
NAME        txf-3-183nmr
EXPNO                  1
PROCNO                 1

F2 - Acquisition Parameters
Date_           20220311
Time               19.28
INSTRUM            spect
PROBHD   5 mm PABBO BB/
PULPROG             zg30
TD                 65536
SOLVENT            CDCl3
NS                     4
DS                     0
SWH             8012.820 Hz
FIDRES          0.122266 Hz
AQ             4.0894465 sec
RG                 34.77
DW                62.400 usec
DE                  6.50 usec
TE                 295.7 K
D1            1.00000000 sec
TD0                    1

======== CHANNEL f1 ========
SFO1         400.1324710 MHz
NUC1                  1H
P1                 14.50 usec
PLW1         11.99499989 W

F2 - Processing parameters
SI                 65536
SF           400.1300118 MHz
WDW                   EM
SSB                    0
LB                  0.30 Hz
GB                     0
PC                  1.00
```


Supplementary Figure 24. ^{1}H NMR spectrum of **3f** (400 MHz, r.t., $CDCl_3$)

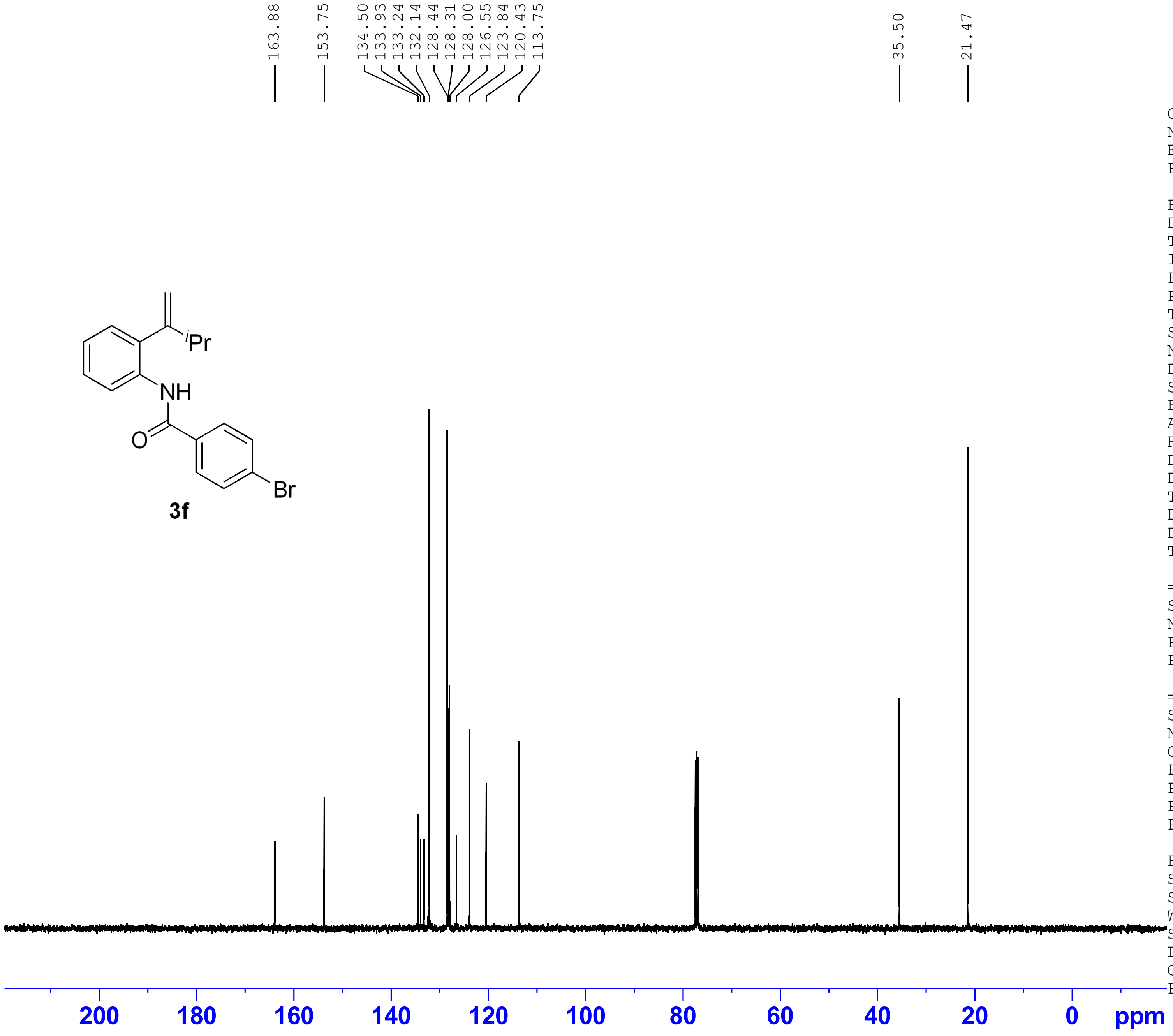


```
Current Data Parameters
NAME         txf-3-183nmr
EXPNO                   2
PROCNO                  1

F2 - Acquisition Parameters
Date_            20220311
Time                19.30
INSTRUM             spect
PROBHD    5 mm PABBO BB/
PULPROG           zgpg30
TD                  65536
SOLVENT             CDCl3
NS                     58
DS                      2
SWH             24038.461 Hz
FIDRES           0.366798 Hz
AQ              1.3631488 sec
RG                 196.92
DW                 20.800 usec
DE                   6.50 usec
TE                  296.2 K
D1             2.00000000 sec
D11            0.03000000 sec
TD0                     1

======== CHANNEL f1 ========
SFO1          100.6228298 MHz
NUC1                  13C
P1                   9.70 usec
PLW1          46.98899841 W

======== CHANNEL f2 ========
SFO2          400.1316005 MHz
NUC2                   1H
CPDPRG[2          waltz16
PCPD2               90.00 usec
PLW2          11.99499989 W
PLW12          0.34213999 W
PLW13          0.27713001 W

F2 - Processing parameters
SI                  32768
SF            100.6127690 MHz
WDW                    EM
SSB                     0
LB                   1.00 Hz
GB                      0
PC                   1.40
```


Supplementary Figure 25. ^{13}C NMR spectrum of **3f** (100 MHz, r.t., $CDCl_3$)

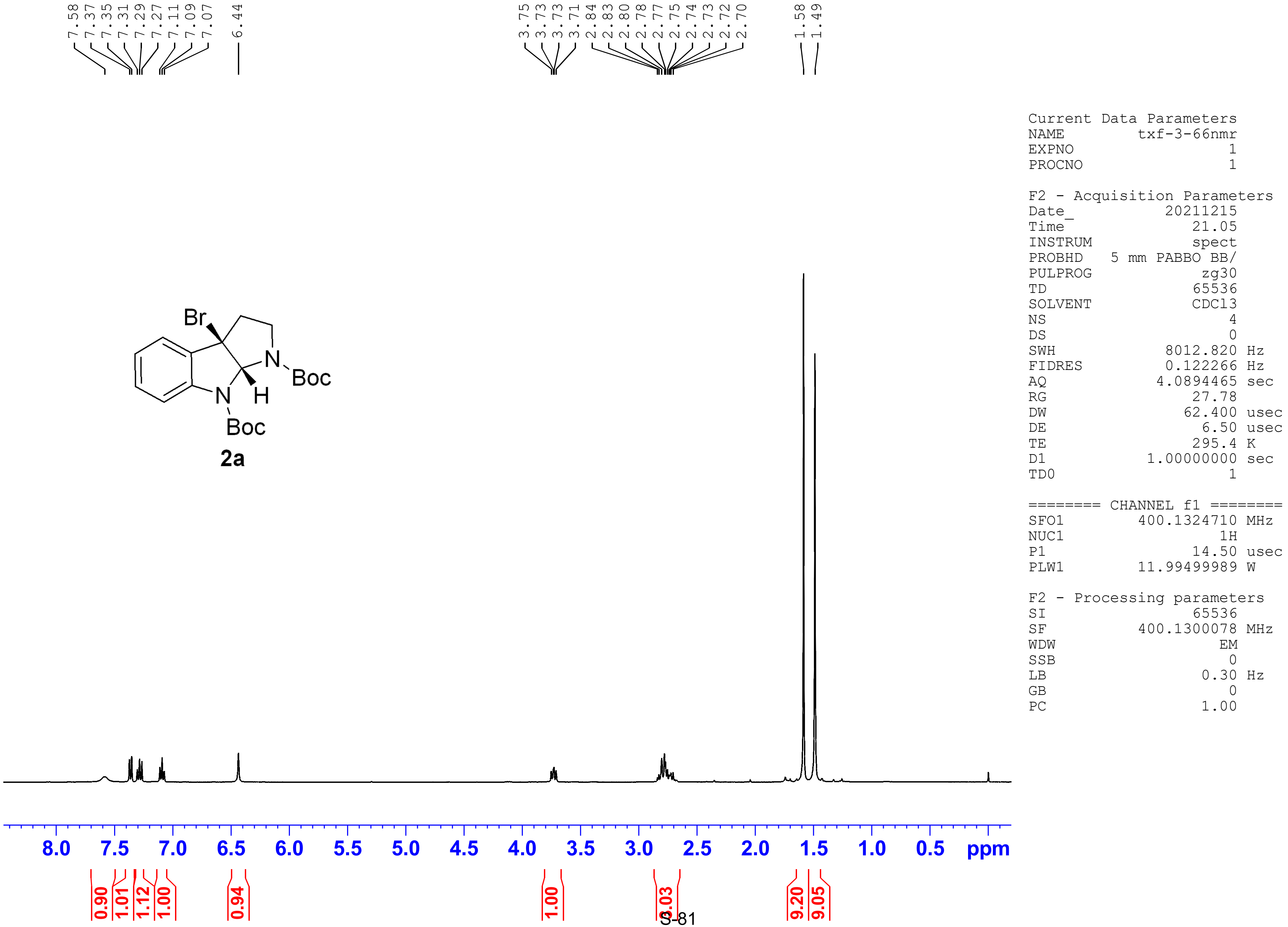

Supplementary Figure 26. ^{1}H NMR spectrum of **2a** (400 MHz, r.t., $CDCl_3$)

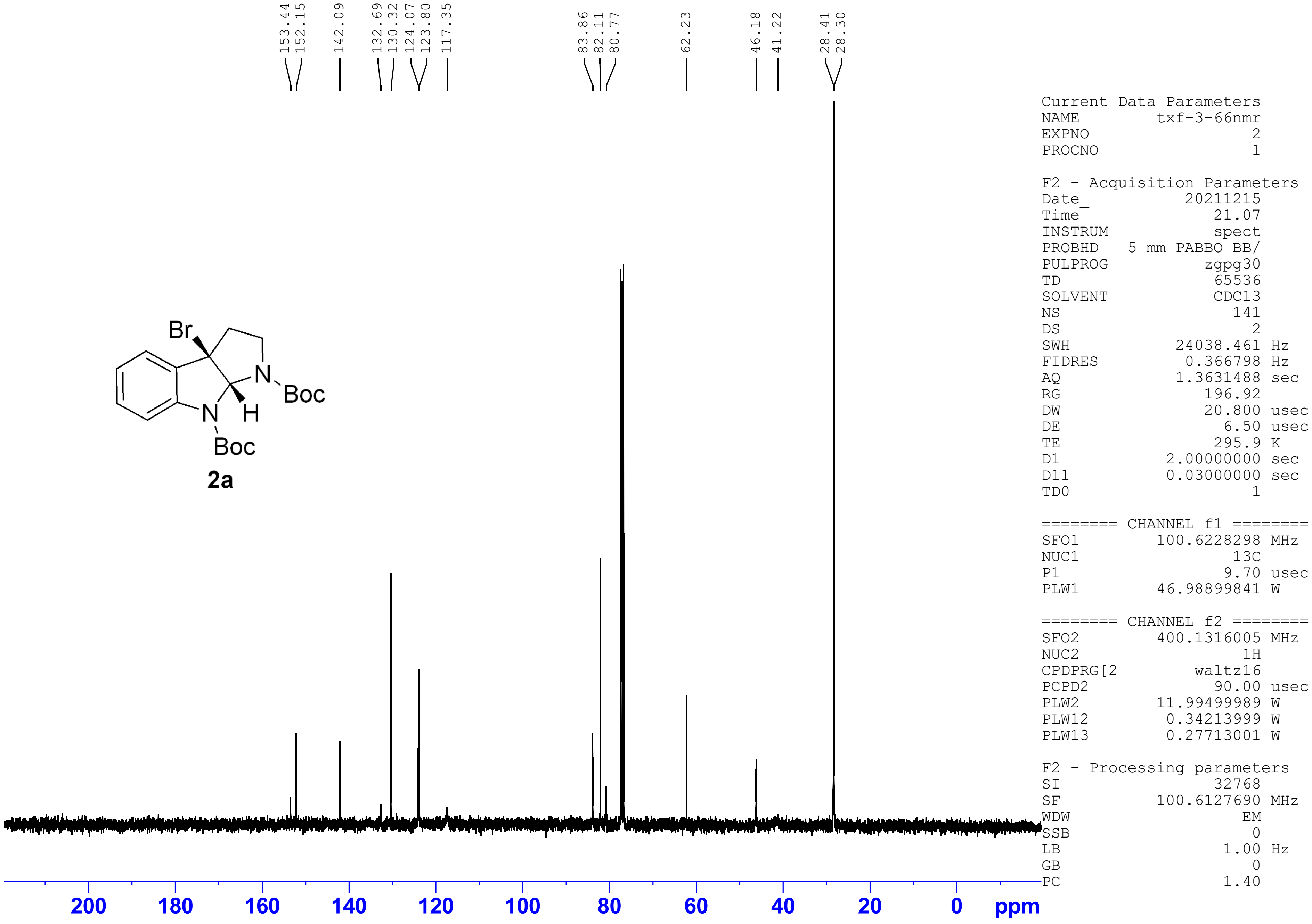

Supplementary Figure 27. ^{13}C NMR spectrum of **2a** (100 MHz, r.t., $CDCl_3$)

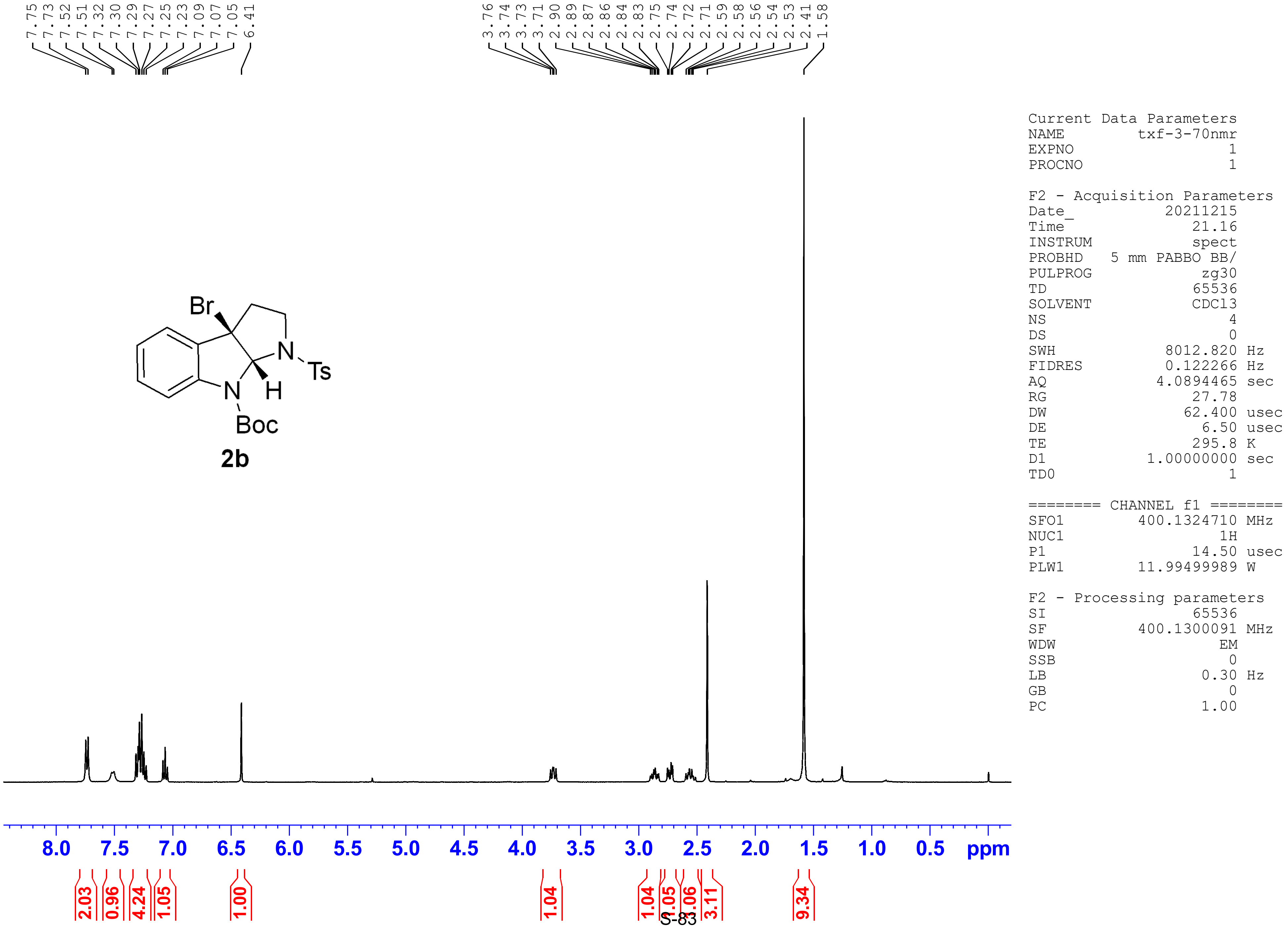

Supplementary Figure 28. ^{1}H NMR spectrum of **2b** (400 MHz, r.t., $CDCl_3$)

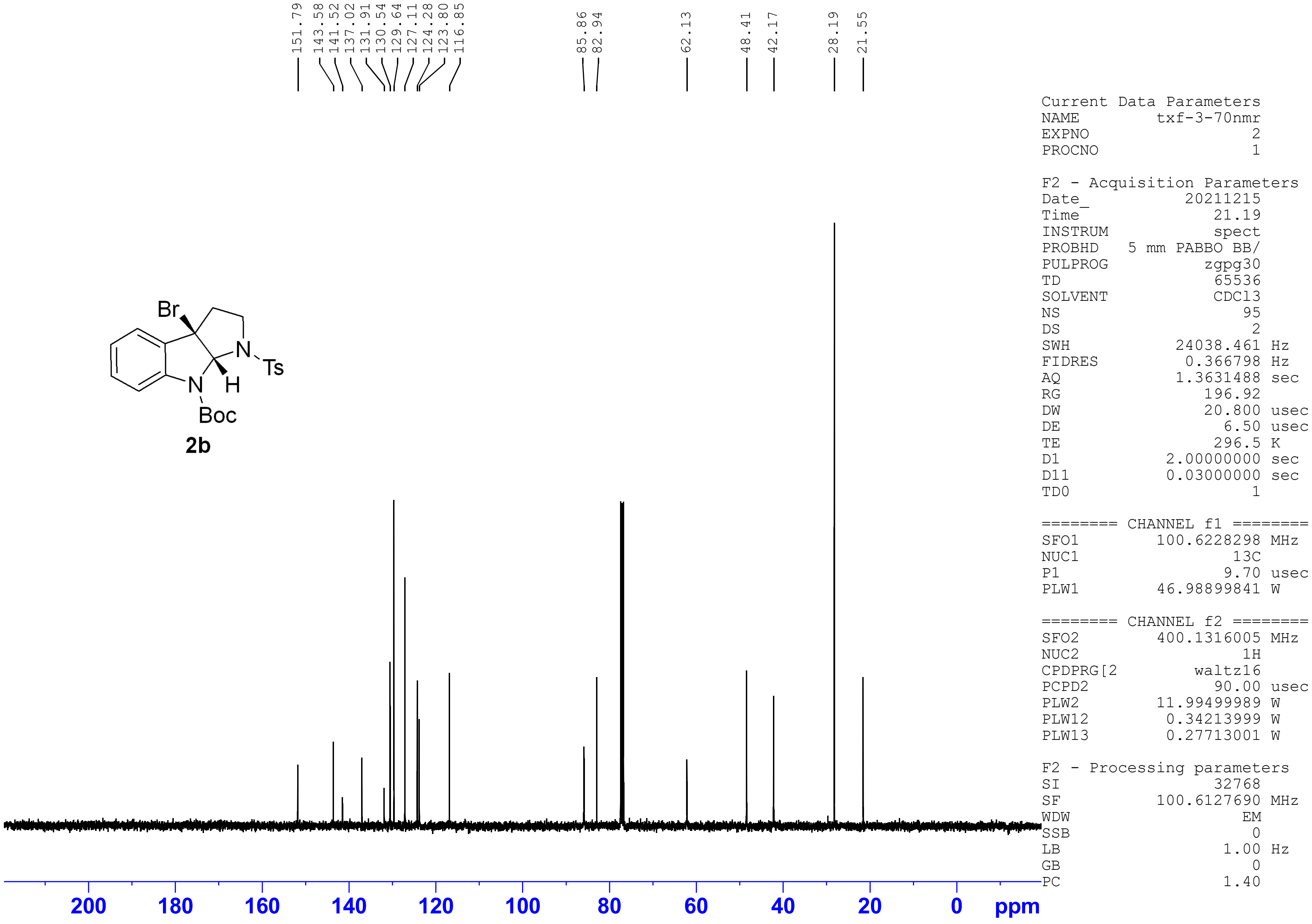

Supplementary Figure 29. ^{13}C NMR spectrum of **2b** (100 MHz, r.t., $CDCl_3$)

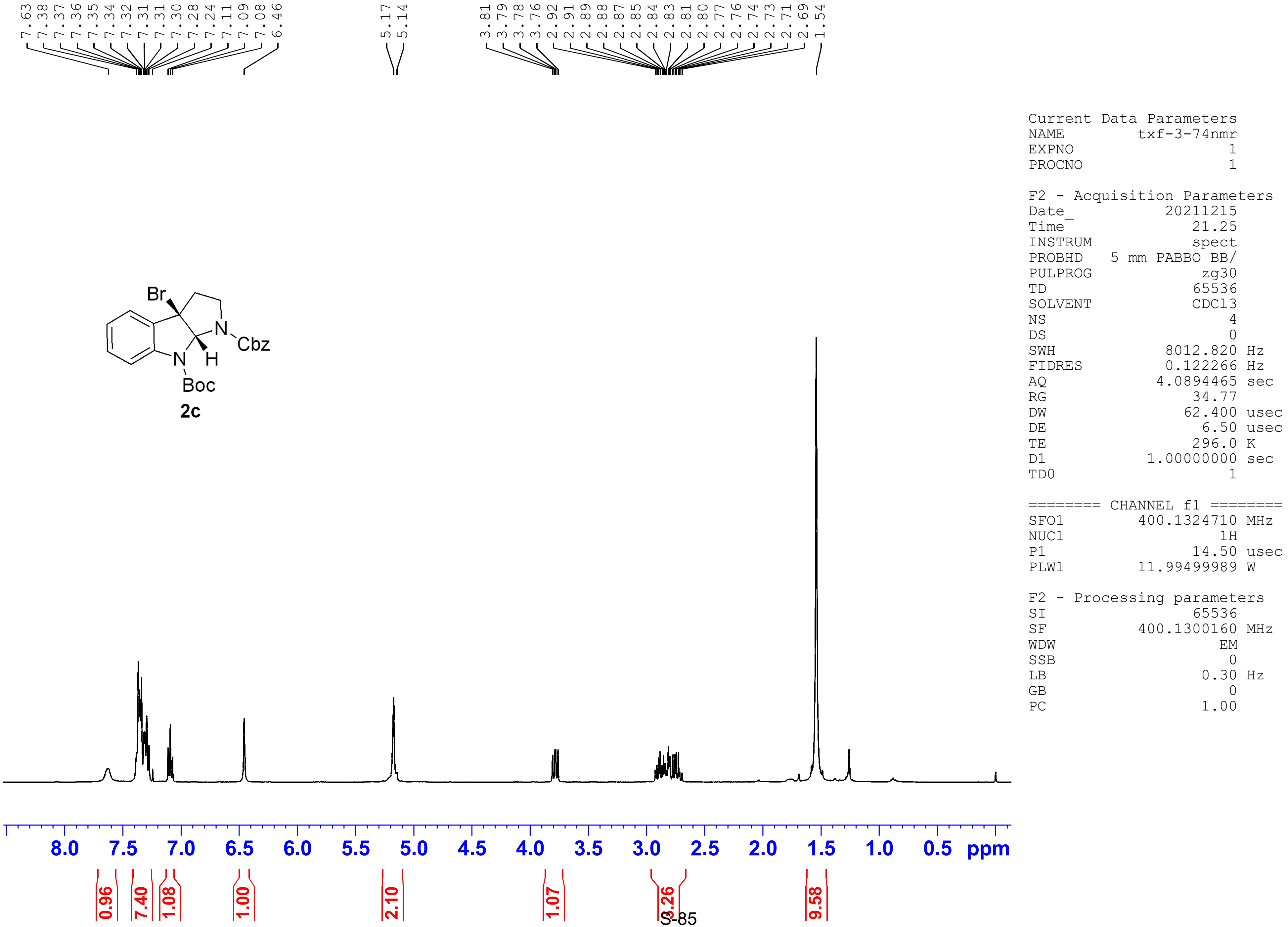

Supplementary Figure 30. ^{1}H NMR spectrum of **2c** (400 MHz, r.t., $CDCl_3$)

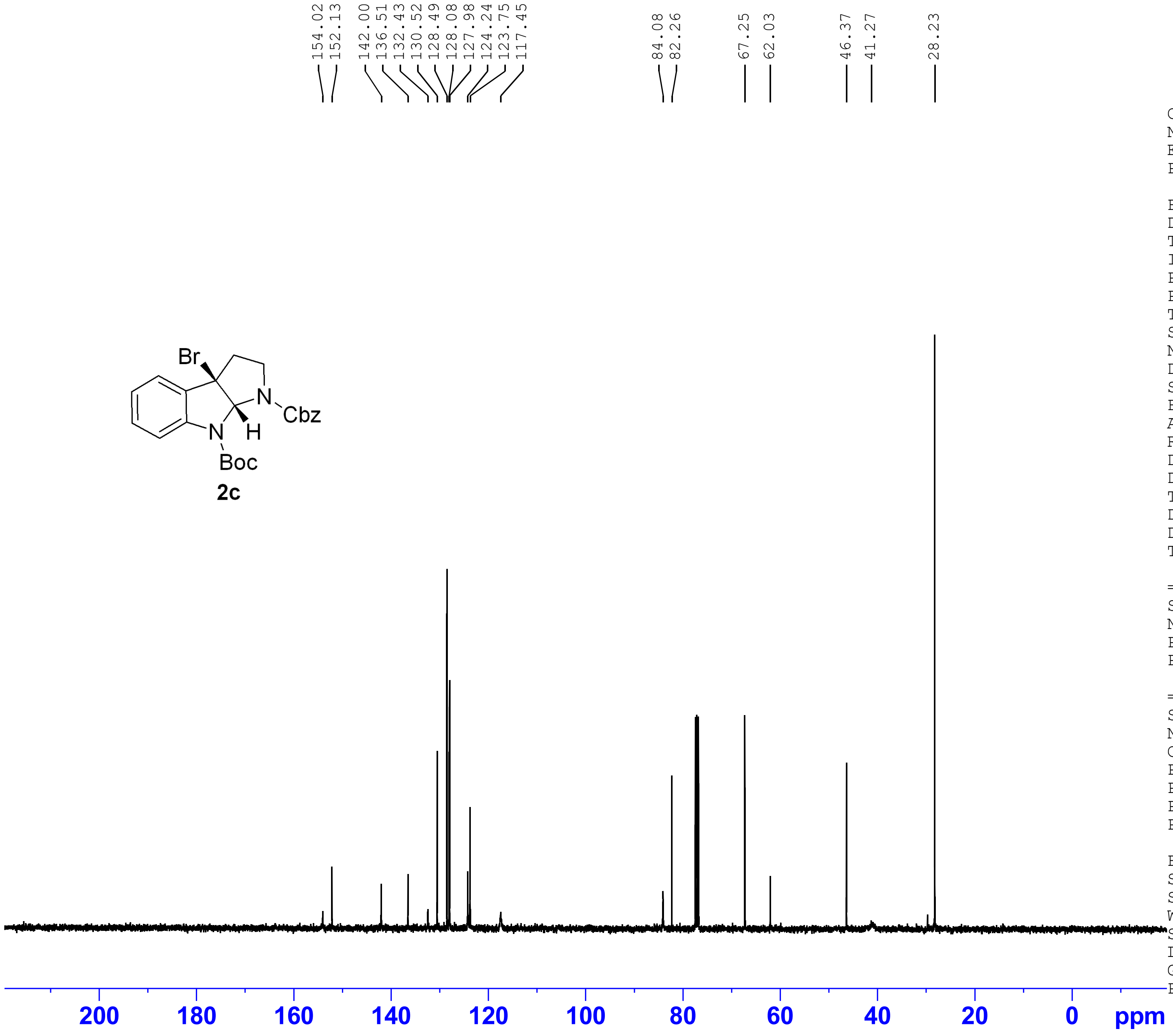

2c


```
Current Data Parameters
NAME          txf-3-74nmr
EXPNO                   2
PROCNO                  1

F2 - Acquisition Parameters
Date_            20211215
Time                21.27
INSTRUM             spect
PROBHD   5 mm PABBO BB/
PULPROG            zgpg30
TD                  65536
SOLVENT             CDCl3
NS                    108
DS                      2
SWH             24038.461 Hz
FIDRES           0.366798 Hz
AQ              1.3631488 sec
RG                 196.92
DW                 20.800 usec
DE                   6.50 usec
TE                  296.6 K
D1             2.00000000 sec
D11            0.03000000 sec
TD0                     1

======== CHANNEL f1 ========
SFO1          100.6228298 MHz
NUC1                  13C
P1                   9.70 usec
PLW1          46.98899841 W

======== CHANNEL f2 ========
SFO2          400.1316005 MHz
NUC2                   1H
CPDPRG[2          waltz16
PCPD2               90.00 usec
PLW2          11.99499989 W
PLW12          0.34213999 W
PLW13          0.27713001 W

F2 - Processing parameters
SI                  32768
SF            100.6127690 MHz
WDW                    EM
SSB                     0
LB                   1.00 Hz
GB                      0
PC                   1.40
```


Supplementary Figure 31. ^{13}C NMR spectrum of **2c** (100 MHz, r.t., $CDCl_3$)

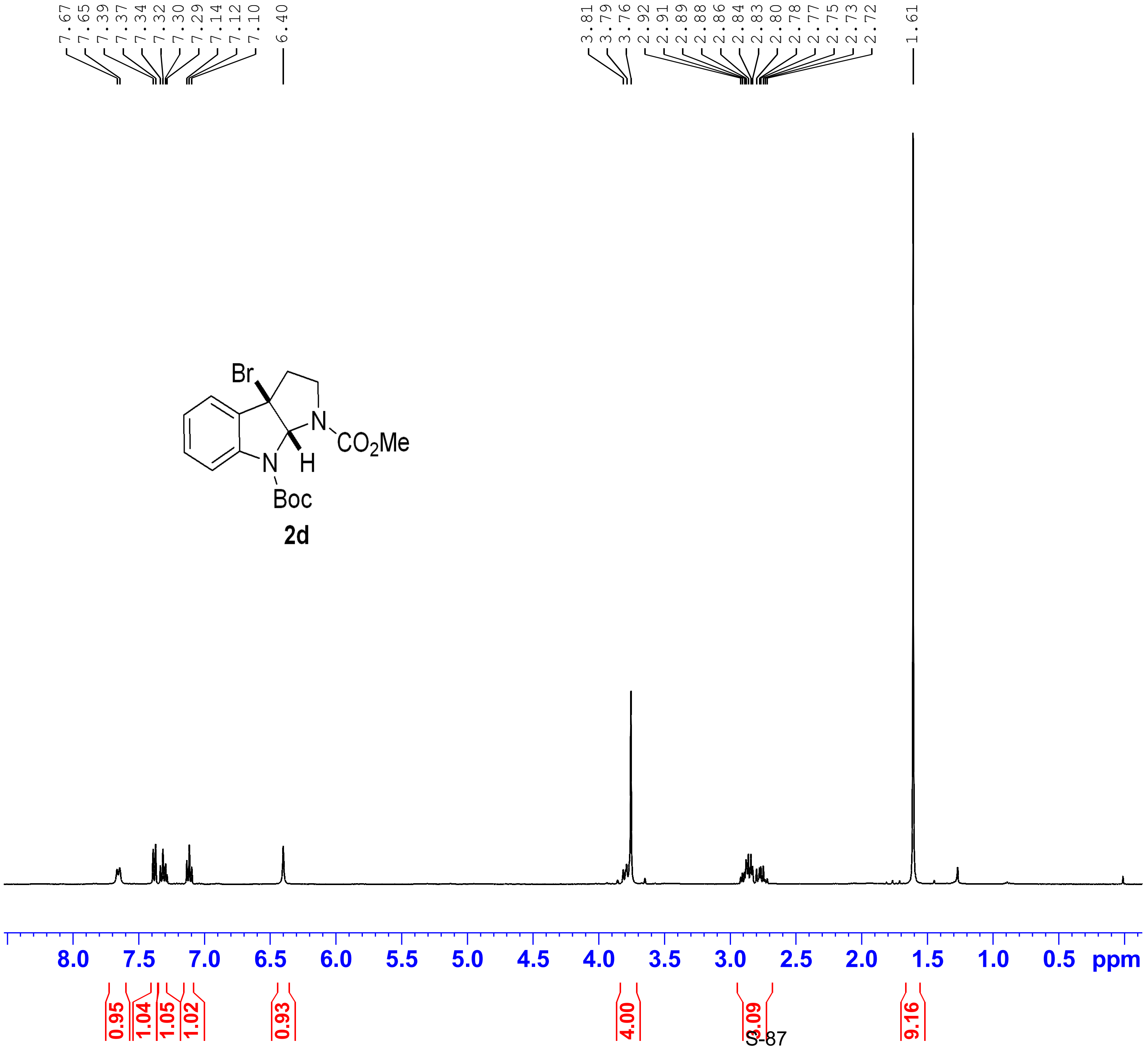


```
Current Data Parameters
NAME          txf-3-75nmr
EXPNO                   1
PROCNO                  1

F2 - Acquisition Parameters
Date_            20211215
Time                21.34
INSTRUM             spect
PROBHD    5 mm PABBO BB/
PULPROG              zg30
TD                  65536
SOLVENT             CDCl3
NS                      4
DS                      0
SWH              8012.820 Hz
FIDRES           0.122266 Hz
AQ              4.0894465 sec
RG                  27.78
DW                 62.400 usec
DE                   6.50 usec
TE                  296.1 K
D1             1.00000000 sec
TD0                     1

======== CHANNEL f1 ========
SFO1          400.1324710 MHz
NUC1                   1H
P1                  14.50 usec
PLW1          11.99499989 W

F2 - Processing parameters
SI                  65536
SF            400.1300000 MHz
WDW                    EM
SSB                     0
LB                   0.30 Hz
GB                      0
PC                   1.00
```


Supplementary Figure 32. ^1H NMR spectrum of **2d** (400 MHz, r.t., $CDCl_3$)

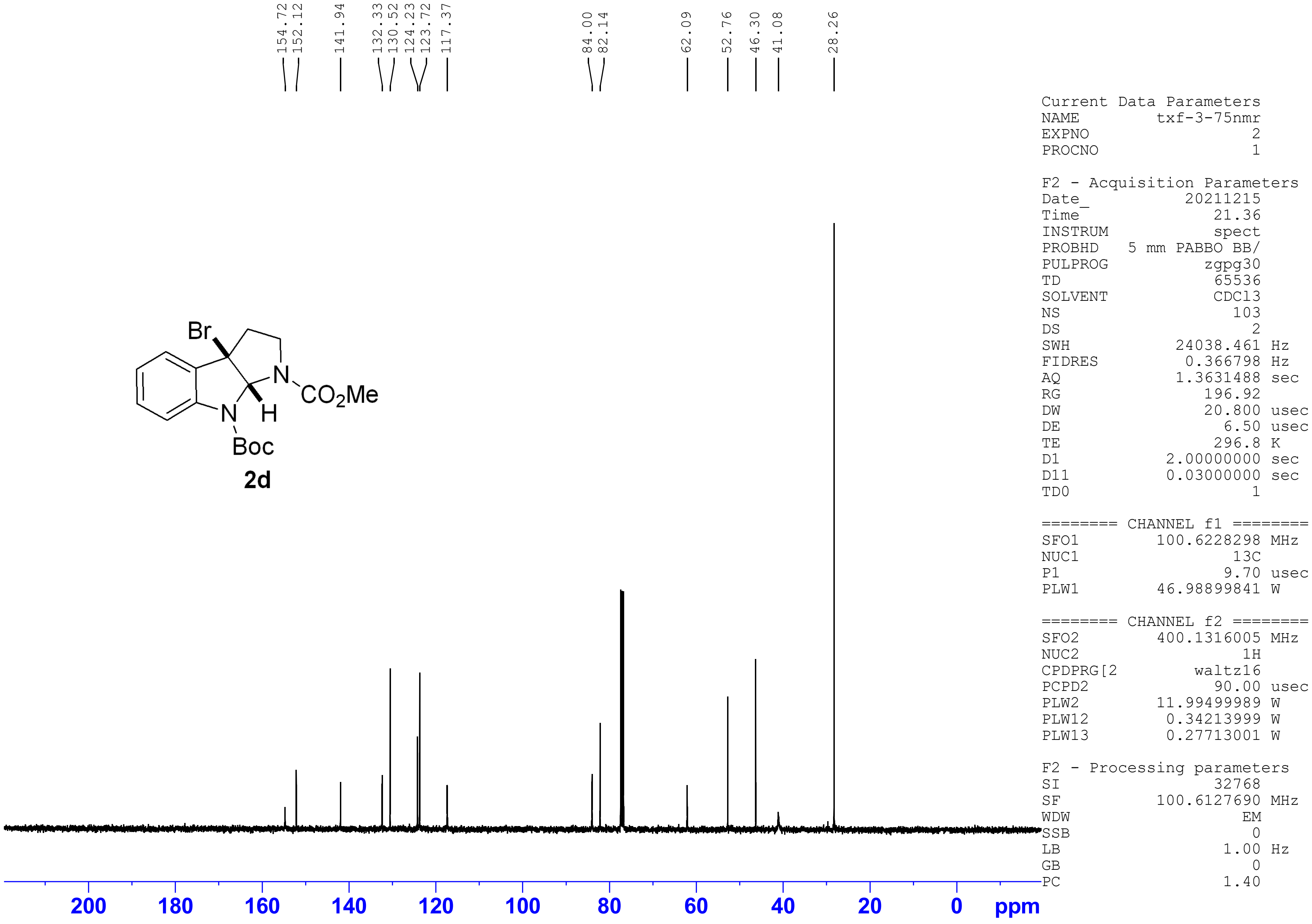

Supplementary Figure 33. ^{13}C NMR spectrum of **2d** (100 MHz, r.t., $CDCl_3$)

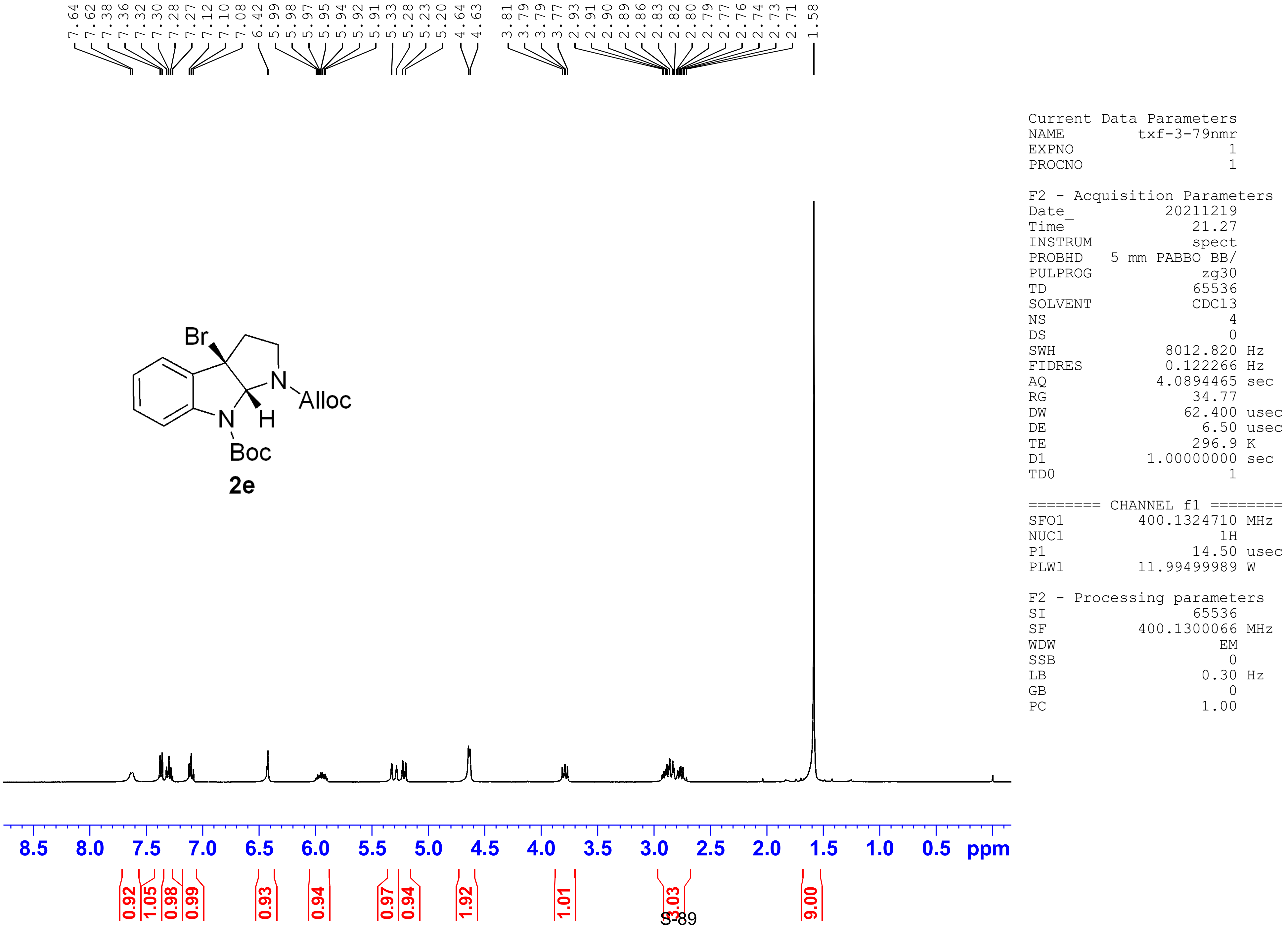

Supplementary Figure 34. ^{1}H NMR spectrum of **2e** (400 MHz, r.t., $CDCl_3$)

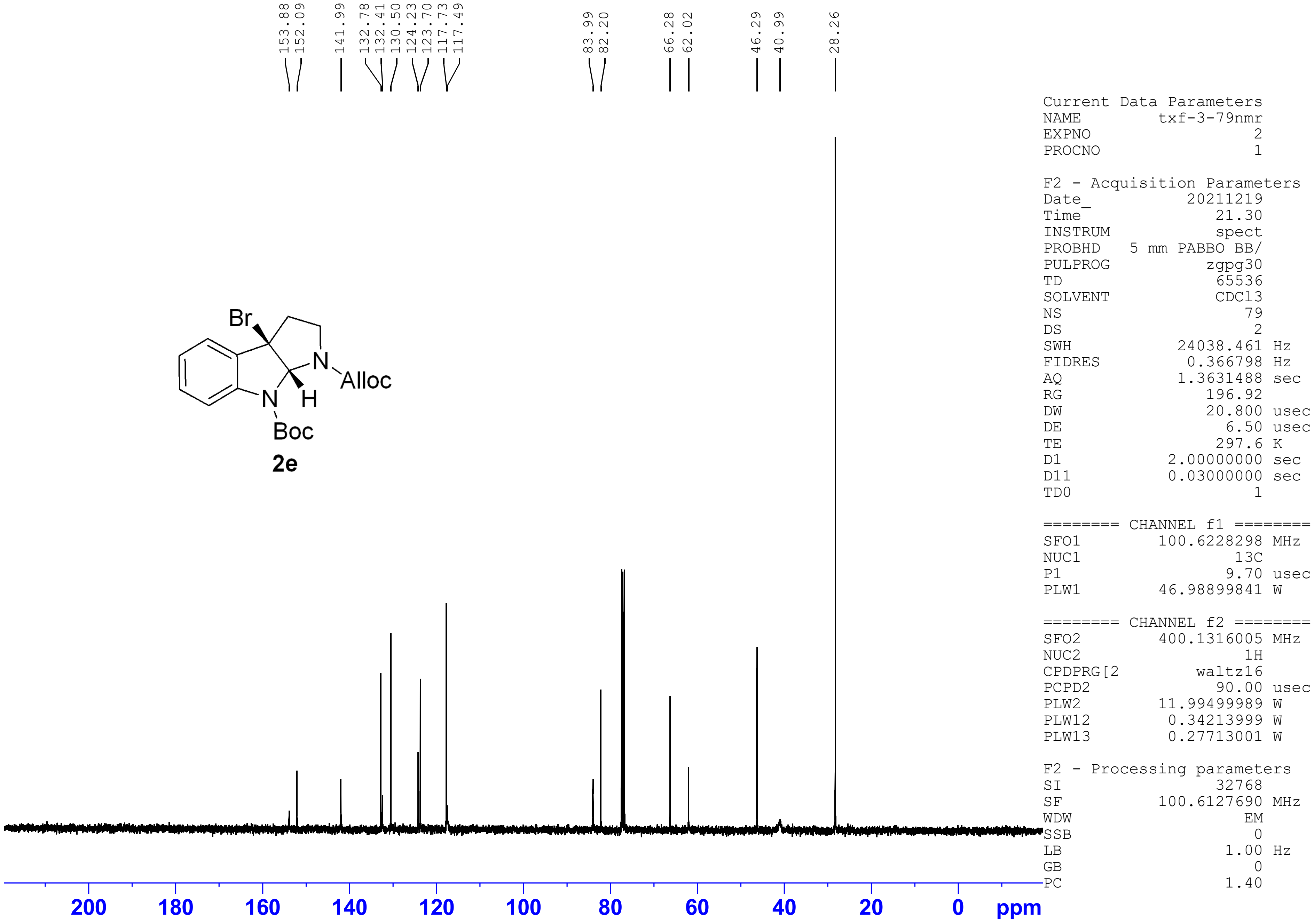

Supplementary Figure 35. ^{13}C NMR spectrum of **2e** (100 MHz, r.t., $CDCl_3$)

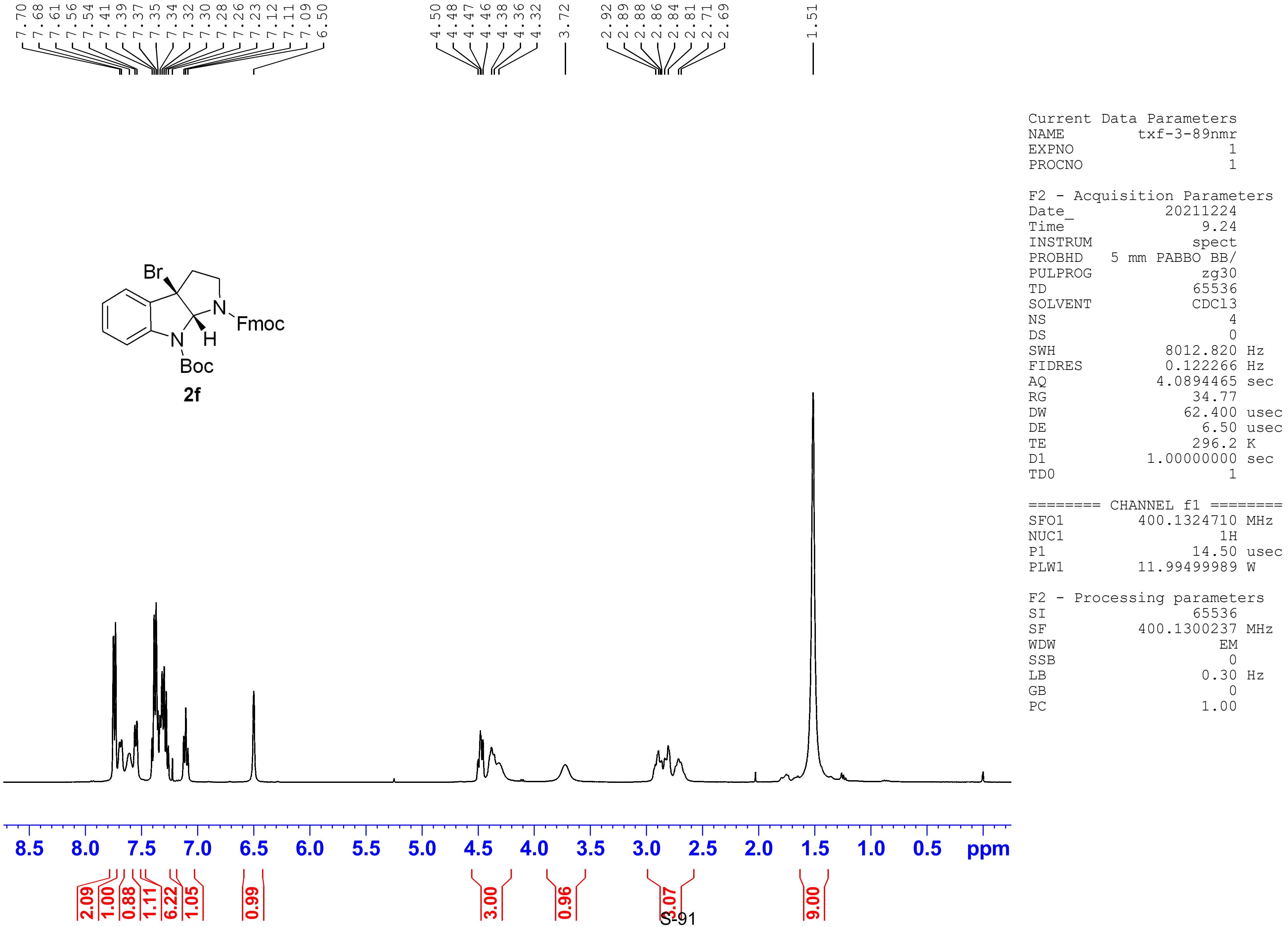

Supplementary Figure 36. ^{1}H NMR spectrum of **2f** (400 MHz, r.t., $CDCl_3$)

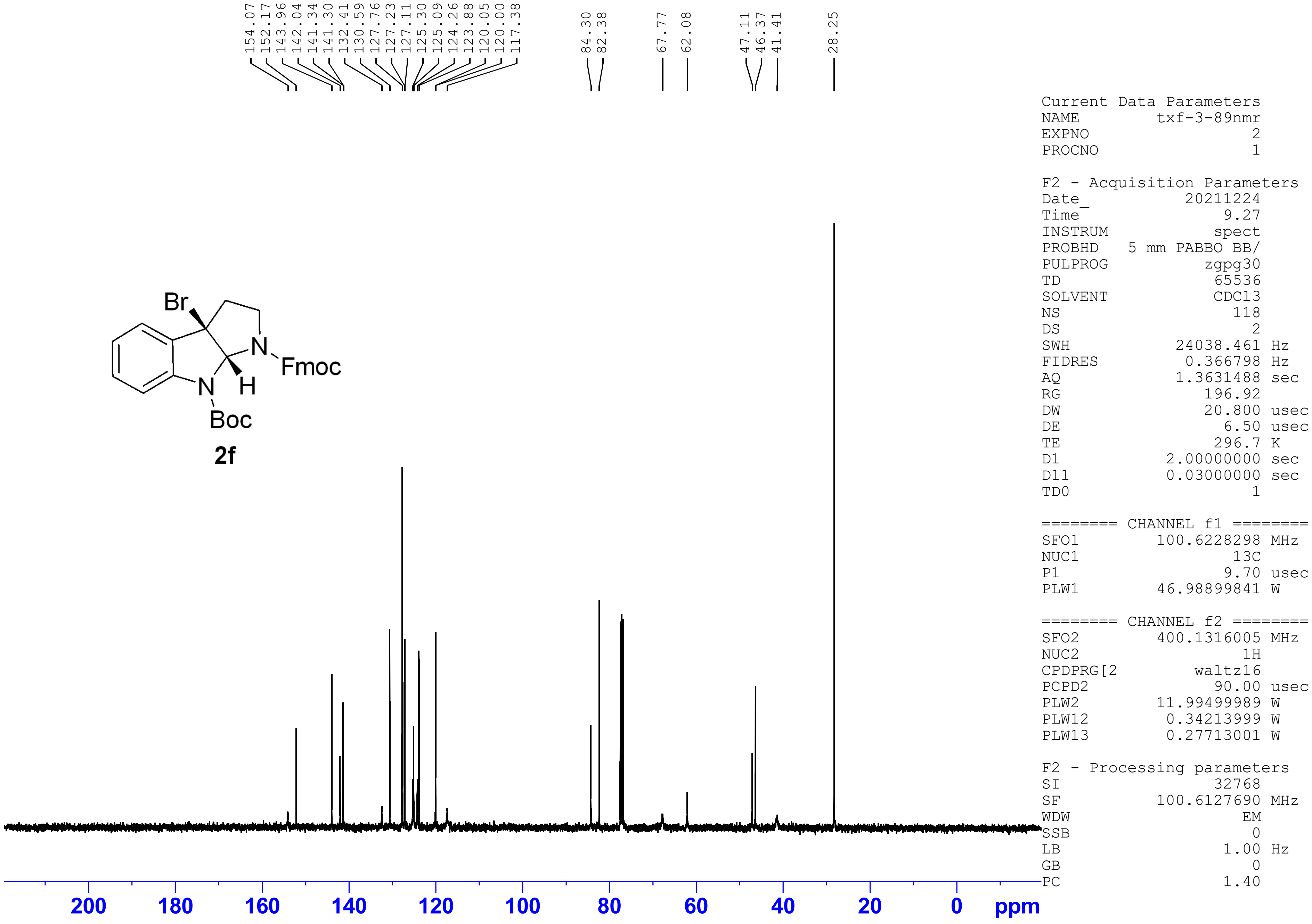

Supplementary Figure 37. ^{13}C NMR spectrum of **2f** (100 MHz, r.t., $CDCl_3$)

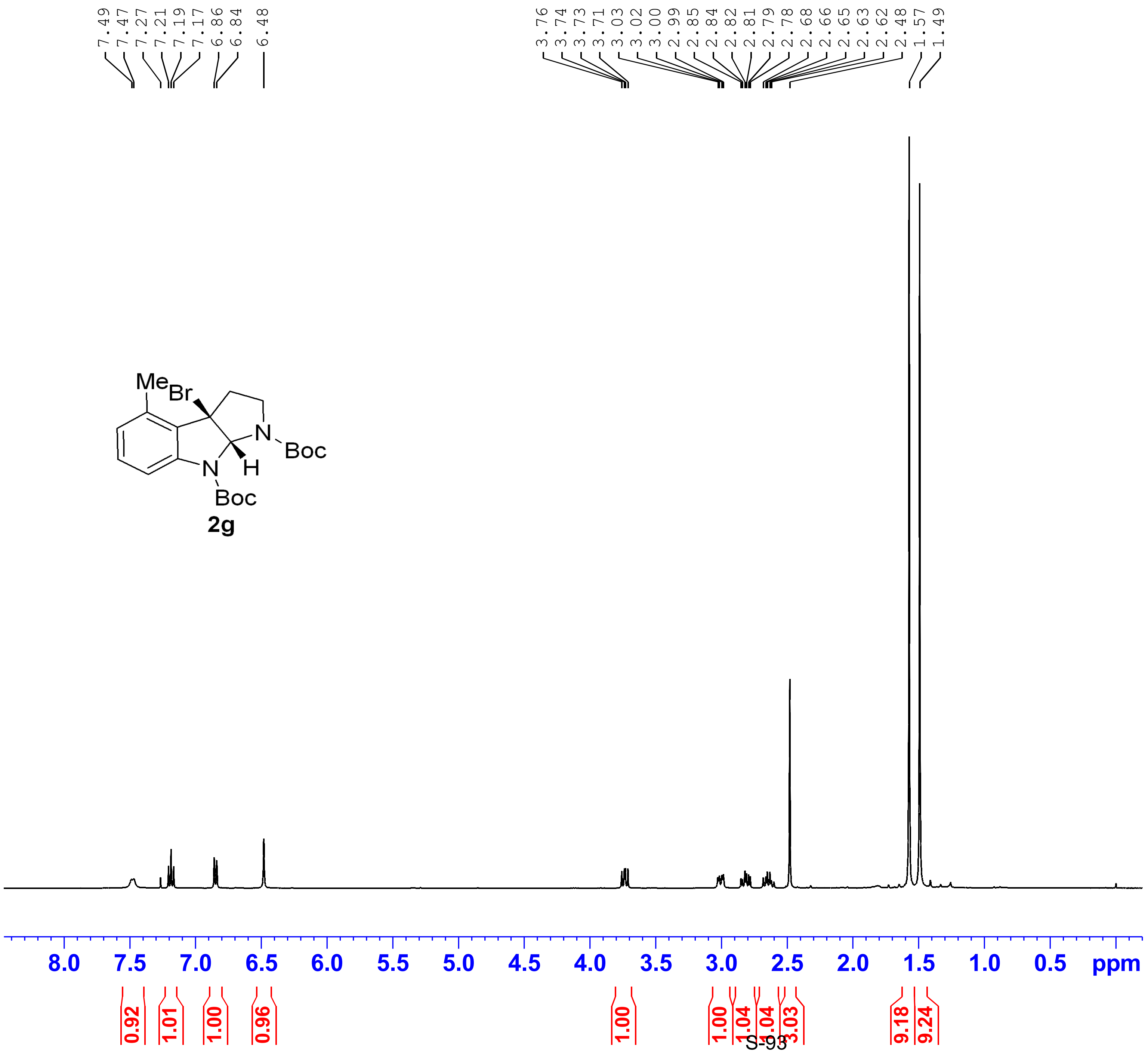

Current Data Parameters
NAME txf-3-133nmr
EXPNO 1
PROCNO 1

F2 - Acquisition Parameters
Date_ 20220122
Time 15.24
INSTRUM spect
PROBHD 5 mm PABBO BB/
PULPROG zg30
TD 65536
SOLVENT CDCl3
NS 4
DS 0
SWH 8012.820 Hz
FIDRES 0.122266 Hz
AQ 4.0894465 sec
RG 39.46
DW 62.400 usec
DE 6.50 usec
TE 295.4 K
D1 1.00000000 sec
TD0 1

======== CHANNEL f1 ========
SFO1 400.1324710 MHz
NUC1 1H
P1 14.50 usec
PLW1 11.99499989 W

F2 - Processing parameters
SI 65536
SF 400.1300071 MHz
WDW EM
SSB 0
LB 0.30 Hz
GB 0
PC 1.00

Supplementary Figure 38. ^{1}H NMR spectrum of **2g** (400 MHz, r.t., $CDCl_3$)

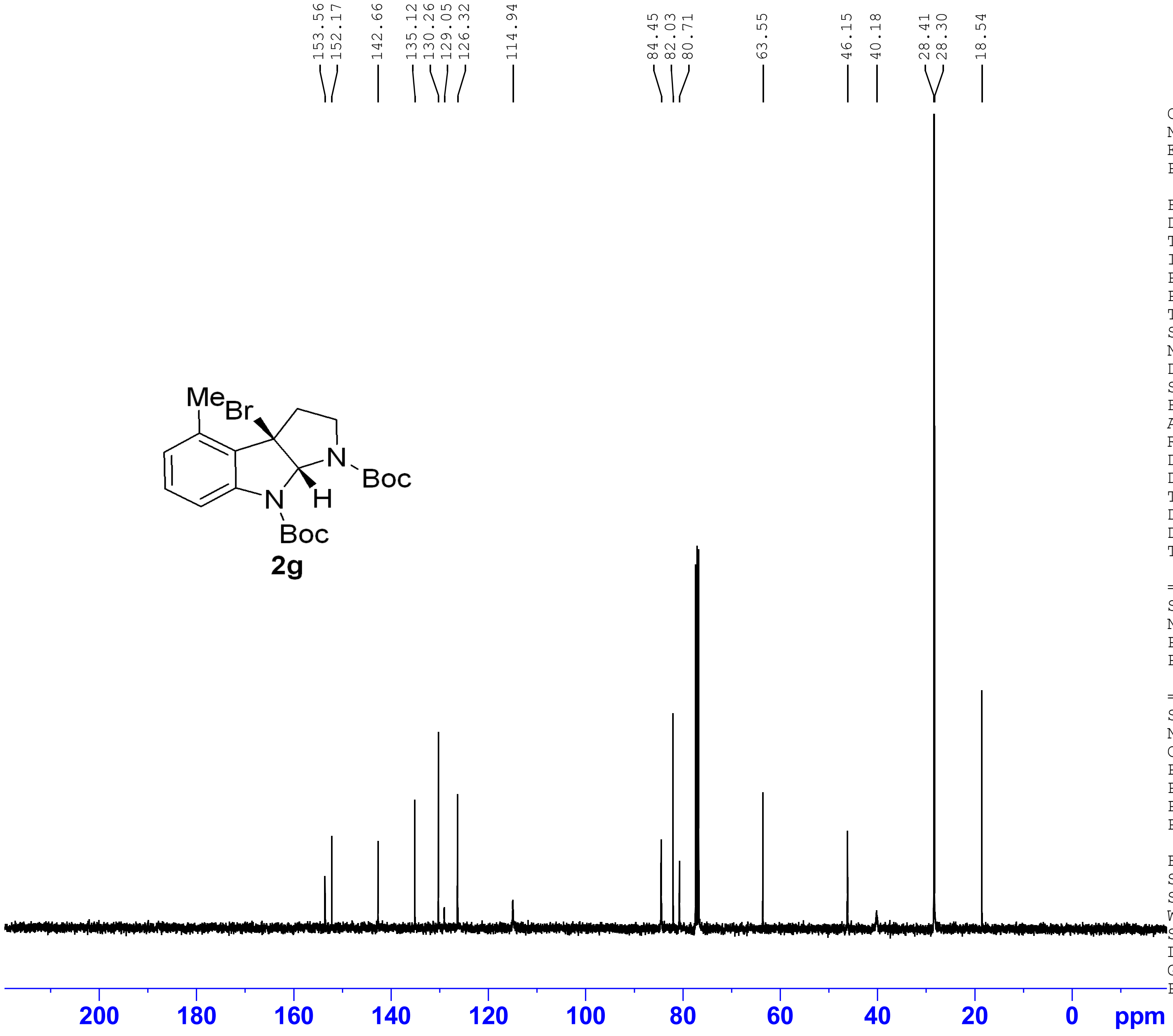


```
Current Data Parameters
NAME        txf-3-133nmr
EXPNO                  2
PROCNO                 1

F2 - Acquisition Parameters
Date_           20220122
Time               15.26
INSTRUM            spect
PROBHD   5 mm PABBO BB/
PULPROG          zgpg30
TD                 65536
SOLVENT            CDCl3
NS                    92
DS                     2
SWH            24038.461 Hz
FIDRES          0.366798 Hz
AQ             1.3631488 sec
RG                196.92
DW                20.800 usec
DE                  6.50 usec
TE                 295.8 K
D1            2.00000000 sec
D11           0.03000000 sec
TD0                    1

======== CHANNEL f1 ========
SFO1         100.6228298 MHz
NUC1                 13C
P1                  9.70 usec
PLW1         46.98899841 W

======== CHANNEL f2 ========
SFO2         400.1316005 MHz
NUC2                  1H
CPDPRG[2         waltz16
PCPD2              90.00 usec
PLW2         11.99499989 W
PLW12         0.34213999 W
PLW13         0.27713001 W

F2 - Processing parameters
SI                 32768
SF           100.6127690 MHz
WDW                   EM
SSB                    0
LB                  1.00 Hz
GB                     0
PC                  1.40
```


Supplementary Figure 39. ^{13}C NMR spectrum of **2g** (100 MHz, r.t., $CDCl_3$)

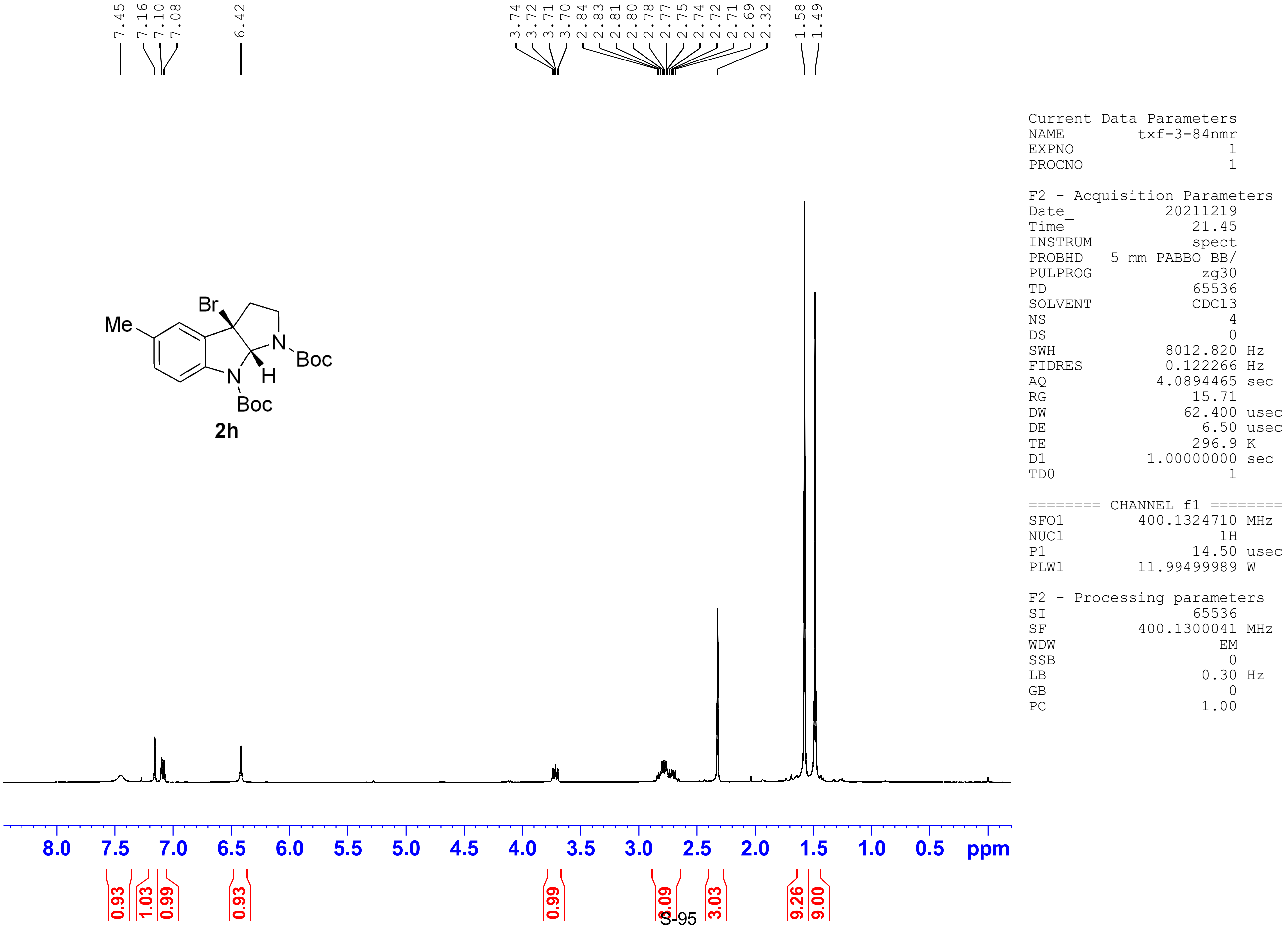

Supplementary Figure 40. ^{1}H NMR spectrum of **2h** (400 MHz, r.t., $CDCl_3$)

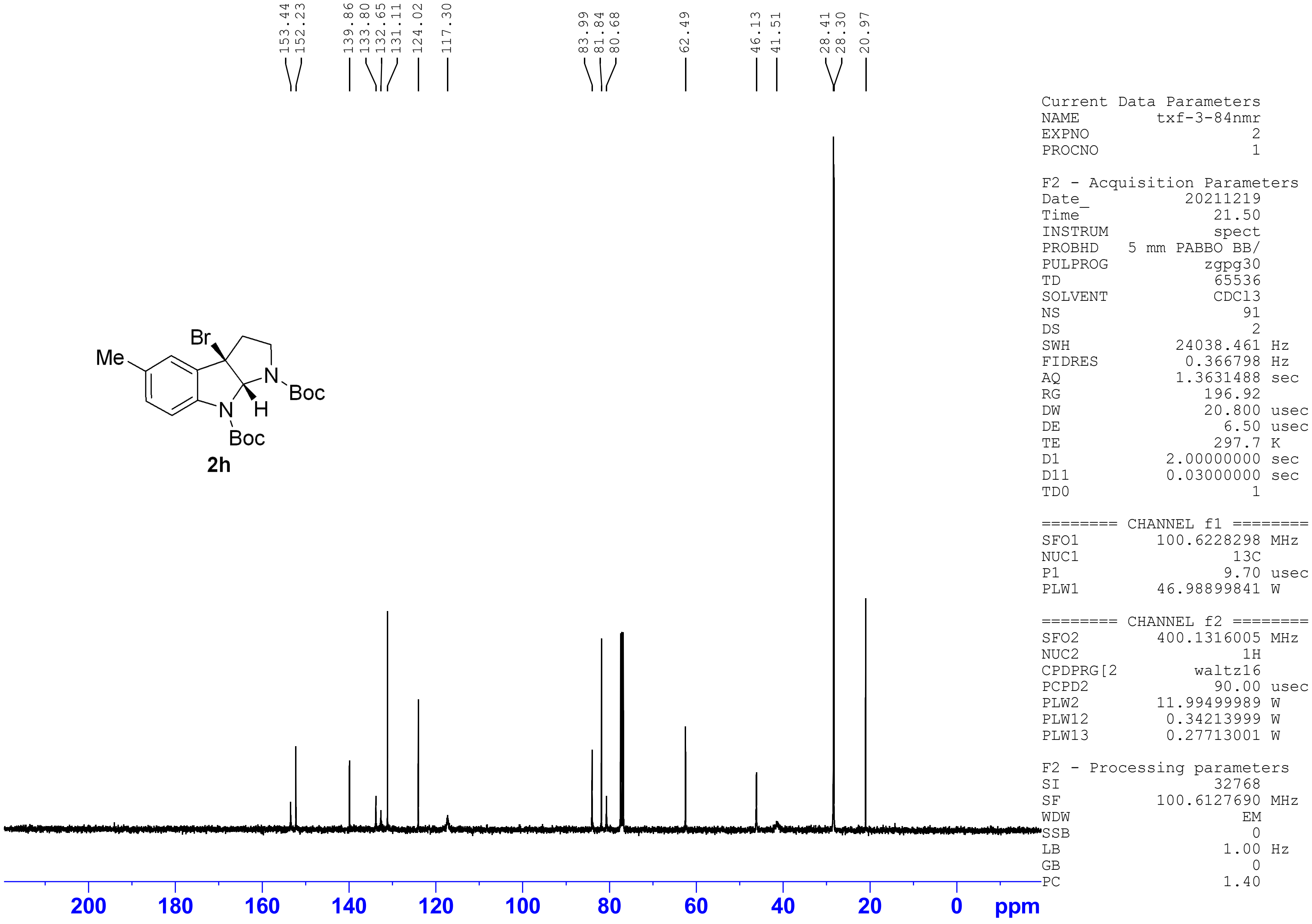

Supplementary Figure 41. ^{13}C NMR spectrum of **2h** (100 MHz, r.t., $CDCl_3$)

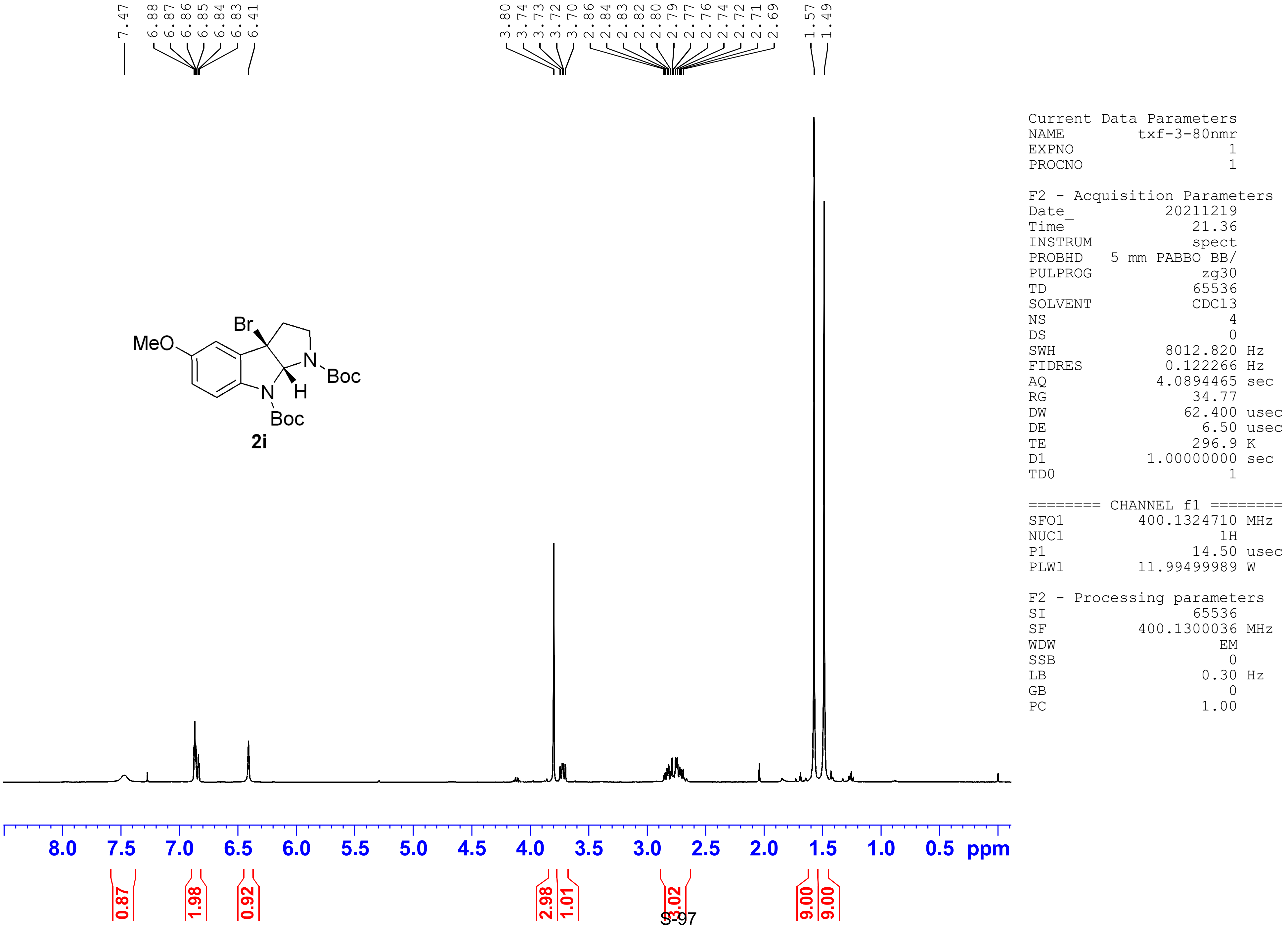

Supplementary Figure 42. ^{1}H NMR spectrum of **2i** (400 MHz, r.t., $CDCl_3$)

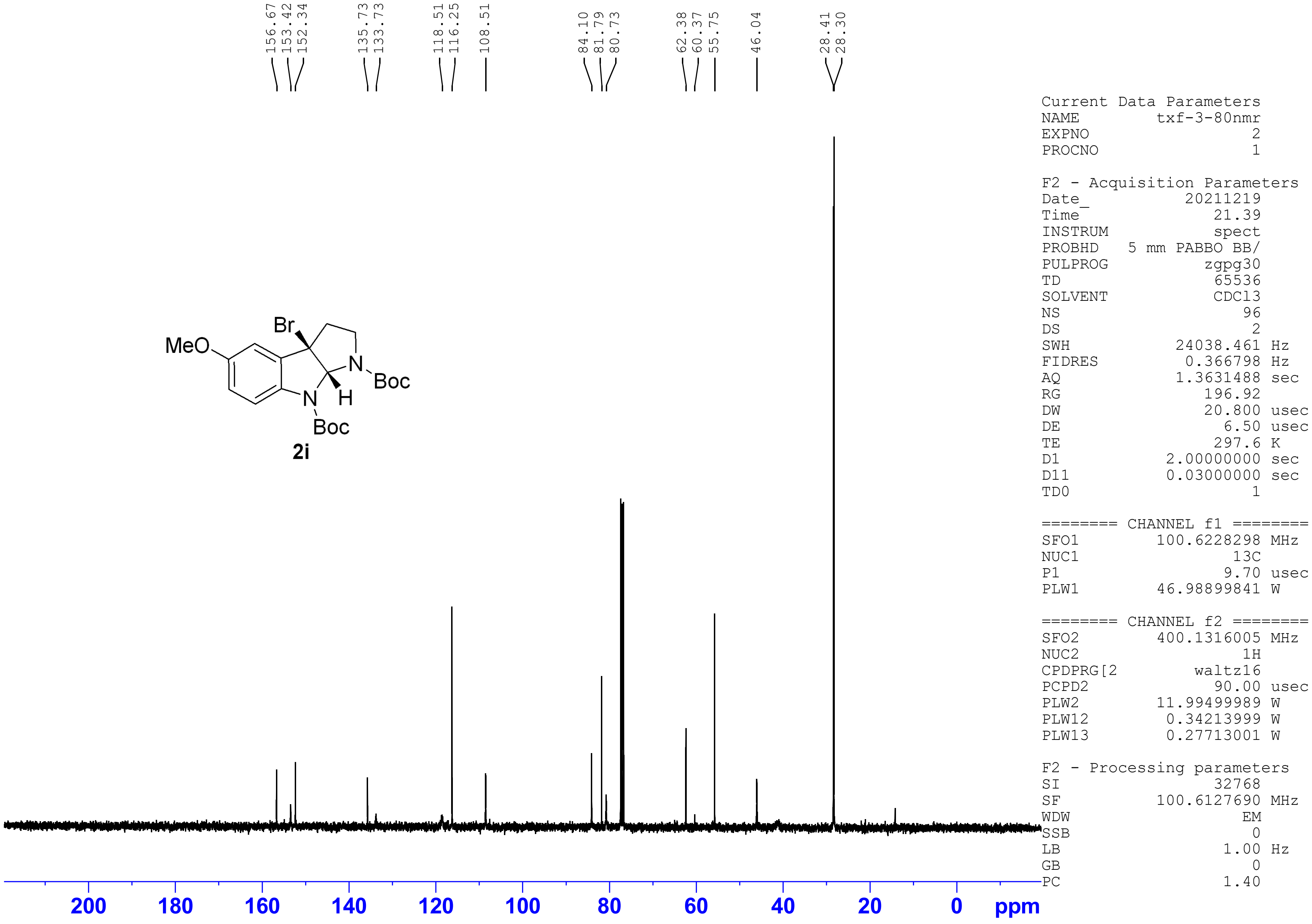

Supplementary Figure 43. ^{13}C NMR spectrum of **2i** (100 MHz, r.t., $CDCl_3$)

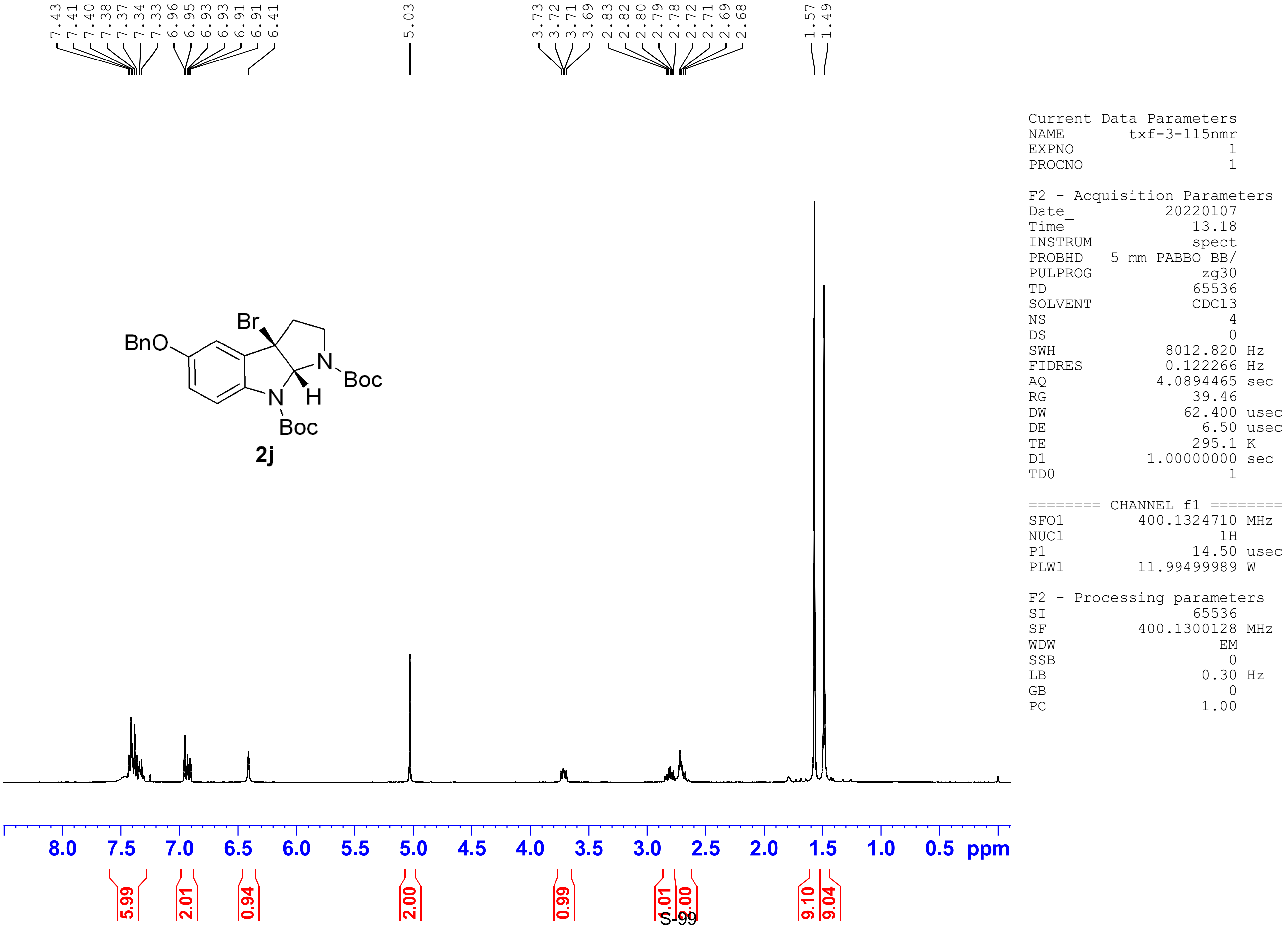

Supplementary Figure 44. ^{1}H NMR spectrum of **2j** (400 MHz, r.t., $CDCl_3$)

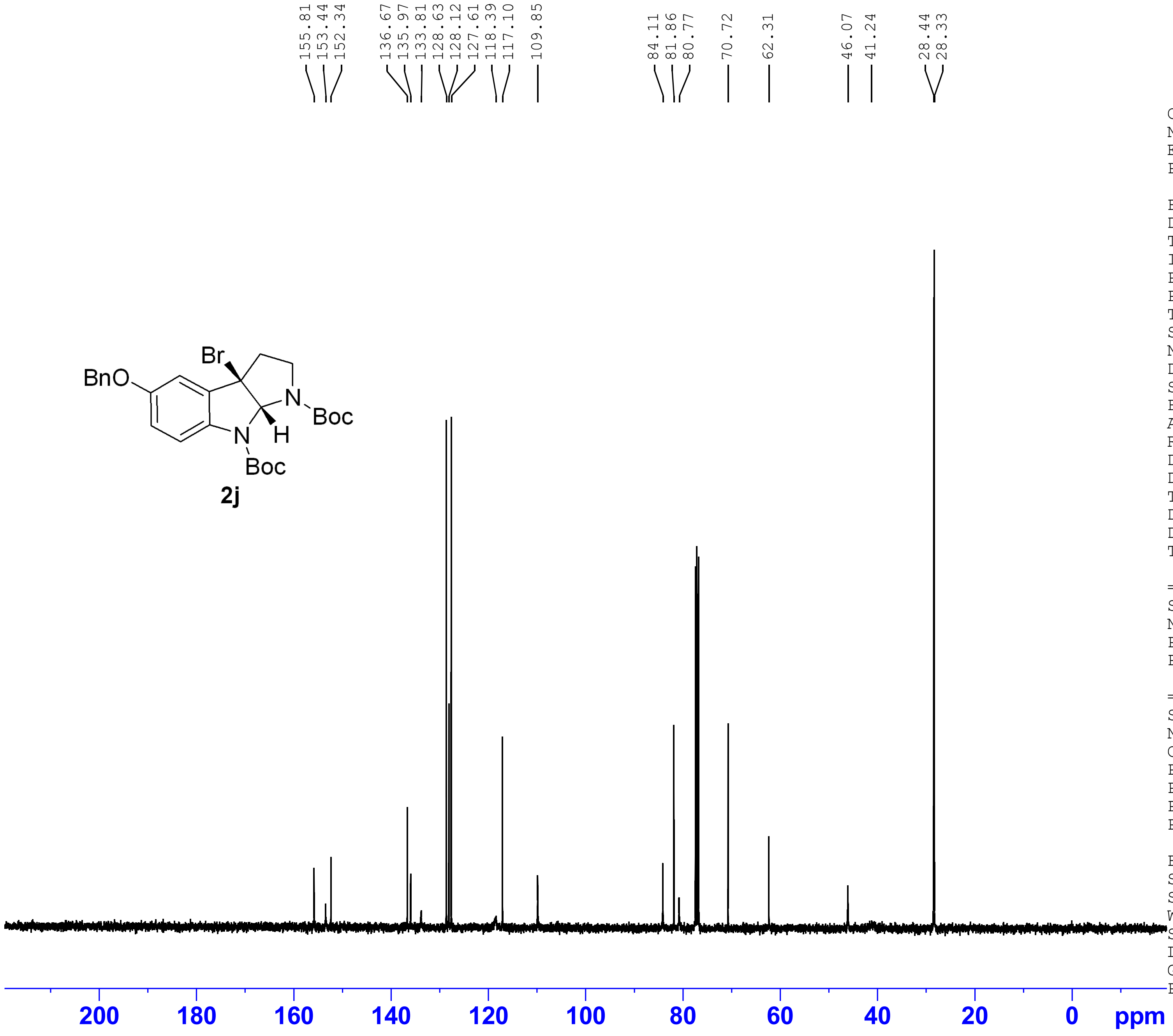


```
Current Data Parameters
NAME       txf-3-115nmr
EXPNO                 2
PROCNO                1

F2 - Acquisition Parameters
Date_          20220107
Time              13.21
INSTRUM           spect
PROBHD   5 mm PABBO BB/
PULPROG         zgpg30
TD               65536
SOLVENT          CDCl3
NS                 110
DS                   2
SWH          24038.461 Hz
FIDRES        0.366798 Hz
AQ           1.3631488 sec
RG              196.92
DW              20.800 usec
DE                6.50 usec
TE               295.5 K
D1          2.00000000 sec
D11         0.03000000 sec
TD0                  1

======== CHANNEL f1 ========
SFO1       100.6228298 MHz
NUC1               13C
P1                9.70 usec
PLW1       46.98899841 W

======== CHANNEL f2 ========
SFO2       400.1316005 MHz
NUC2                1H
CPDPRG[2       waltz16
PCPD2            90.00 usec
PLW2       11.99499989 W
PLW12       0.34213999 W
PLW13       0.27713001 W

F2 - Processing parameters
SI               32768
SF         100.6127690 MHz
WDW                 EM
SSB                  0
LB                1.00 Hz
GB                   0
PC                1.40
```


Supplementary Figure 45. ^{13}C NMR spectrum of **2j** (100 MHz, r.t., $CDCl_3$)

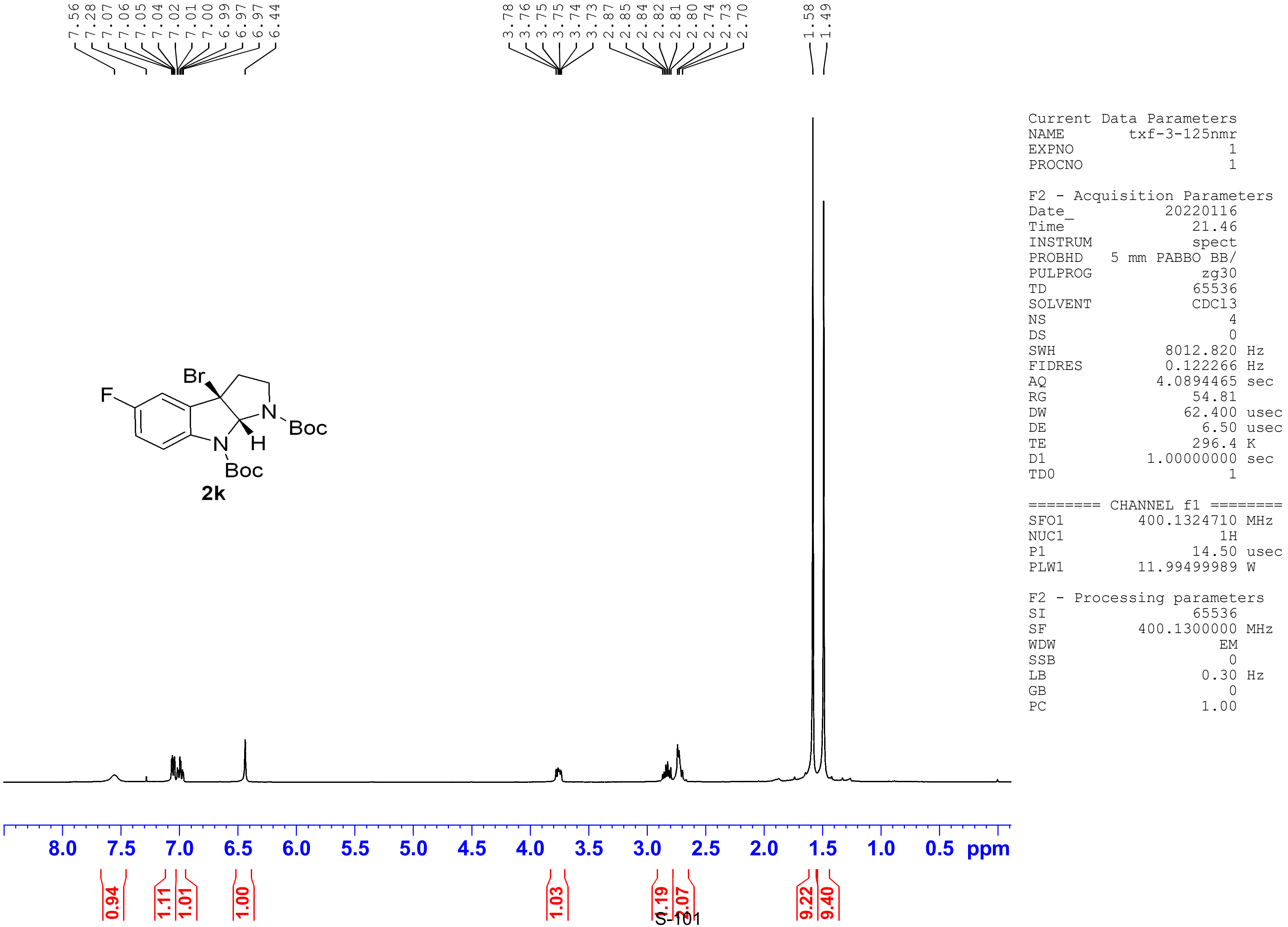

Supplementary Figure 46. ^{1}H NMR spectrum of **2k** (400 MHz, r.t., $CDCl_3$)

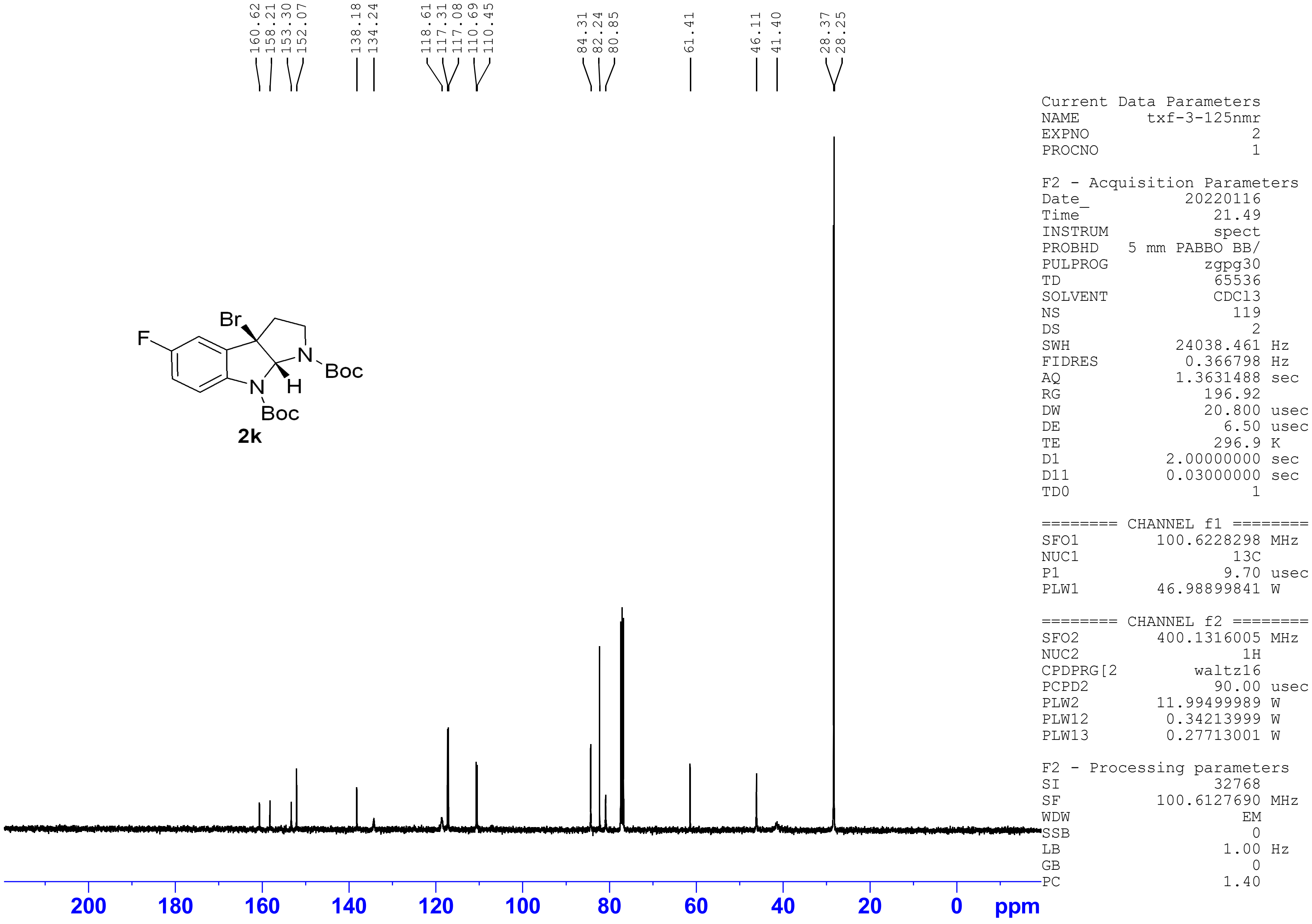

Supplementary Figure 47. ^{13}C NMR spectrum of **2k** (100 MHz, r.t., $CDCl_3$)

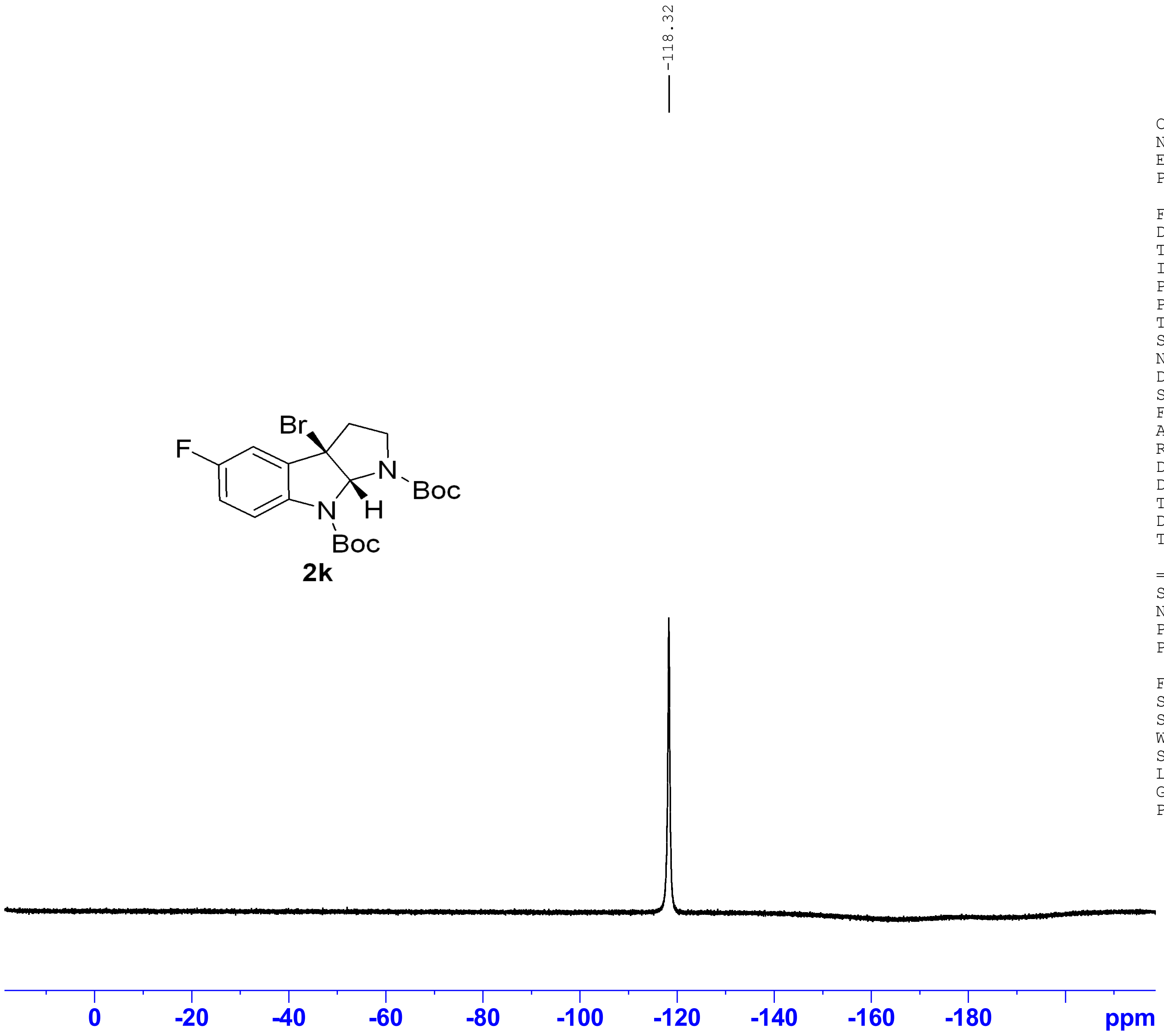


```
Current Data Parameters
NAME        txf-3-125nmr
EXPNO                  3
PROCNO                 1

F2 - Acquisition Parameters
Date_           20220116
Time               21.56
INSTRUM            spect
PROBHD    5 mm PABBO BB/
PULPROG           zgflqn
TD                131072
SOLVENT            CDCl3
NS                    16
DS                     4
SWH            89285.711 Hz
FIDRES          0.681196 Hz
AQ             0.7340032 sec
RG                196.92
DW                 5.600 usec
DE                  6.50 usec
TE                 296.7 K
D1            1.00000000 sec
TD0                    1

======== CHANNEL f1 ========
SFO1         376.4607164 MHz
NUC1                 19F
P1                 14.70 usec
PLW1         15.99600029 W

F2 - Processing parameters
SI                 65536
SF           376.4983660 MHz
WDW                   EM
SSB                    0
LB                  0.30 Hz
GB                     0
PC                  1.00
```


Supplementary Figure 48. ^{19}F NMR spectrum of **2k** (376 MHz, r.t., $CDCl_3$)

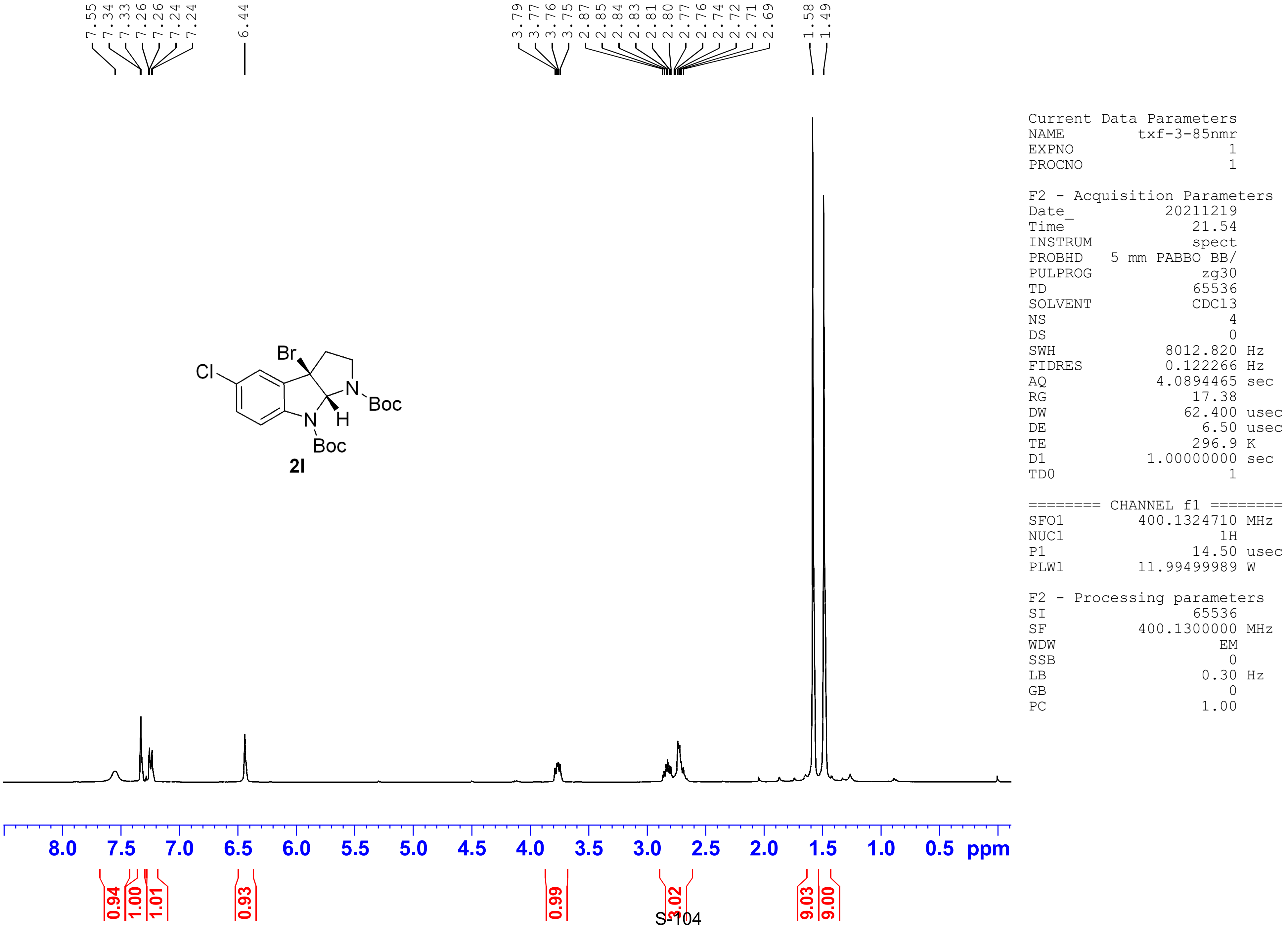

Supplementary Figure 49. ^{1}H NMR spectrum of **2l** (400 MHz, r.t., $CDCl_3$)

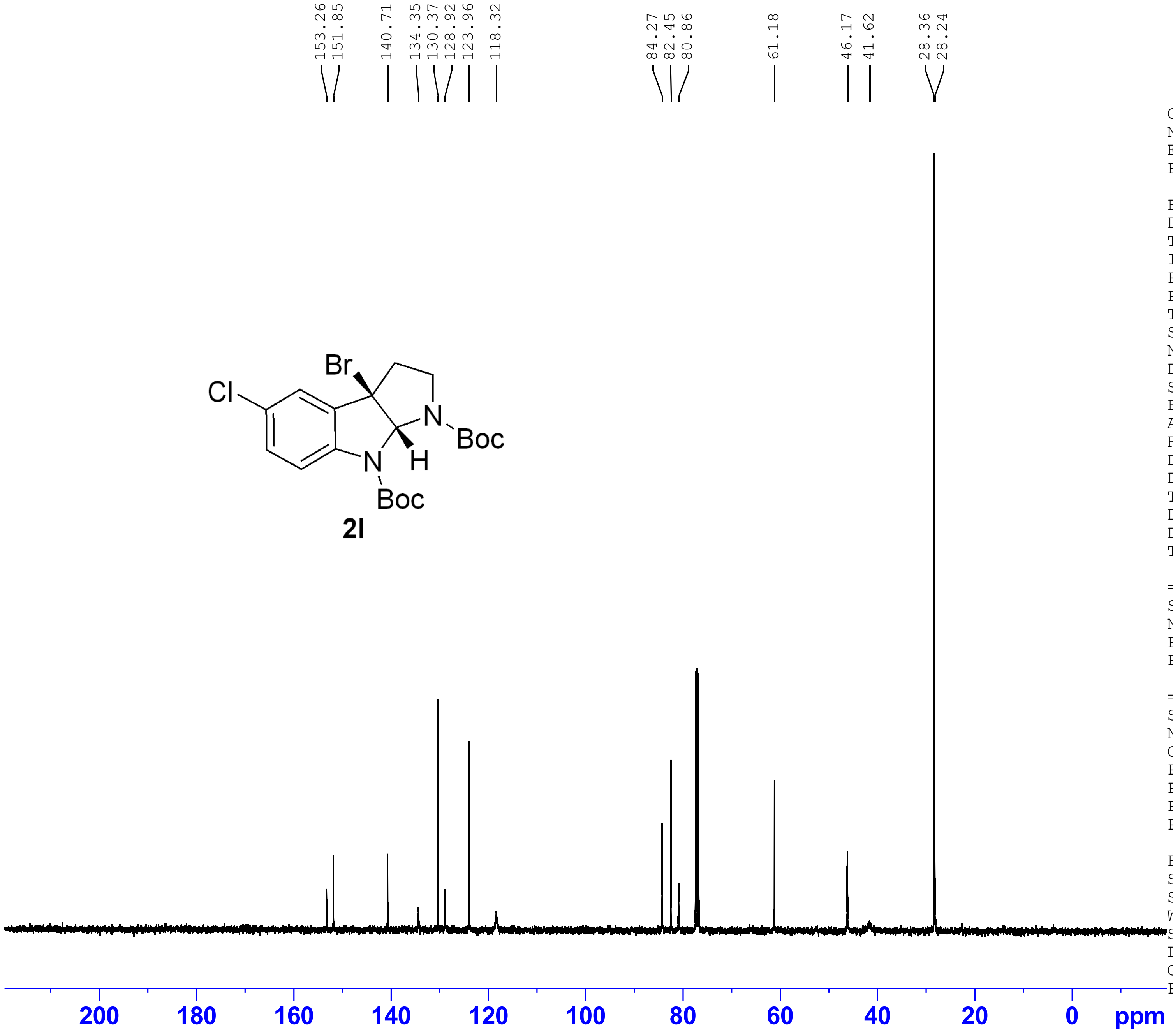


```
Current Data Parameters
NAME          txf-3-85nmr
EXPNO                   2
PROCNO                  1

F2 - Acquisition Parameters
Date_            20211219
Time                21.57
INSTRUM             spect
PROBHD    5 mm PABBO BB/
PULPROG           zgpg30
TD                 65536
SOLVENT            CDCl3
NS                   110
DS                     2
SWH            24038.461 Hz
FIDRES          0.366798 Hz
AQ             1.3631488 sec
RG                196.92
DW                20.800 usec
DE                  6.50 usec
TE                 297.6 K
D1            2.00000000 sec
D11           0.03000000 sec
TD0                    1

======== CHANNEL f1 ========
SFO1         100.6228298 MHz
NUC1                 13C
P1                  9.70 usec
PLW1         46.98899841 W

======== CHANNEL f2 ========
SFO2         400.1316005 MHz
NUC2                  1H
CPDPRG[2         waltz16
PCPD2              90.00 usec
PLW2         11.99499989 W
PLW12         0.34213999 W
PLW13         0.27713001 W

F2 - Processing parameters
SI                 32768
SF           100.6127690 MHz
WDW                   EM
SSB                    0
LB                  1.00 Hz
GB                     0
PC                  1.40
```


Supplementary Figure 50. ^{13}C NMR spectrum of **2l** (100 MHz, r.t., $CDCl_3$)

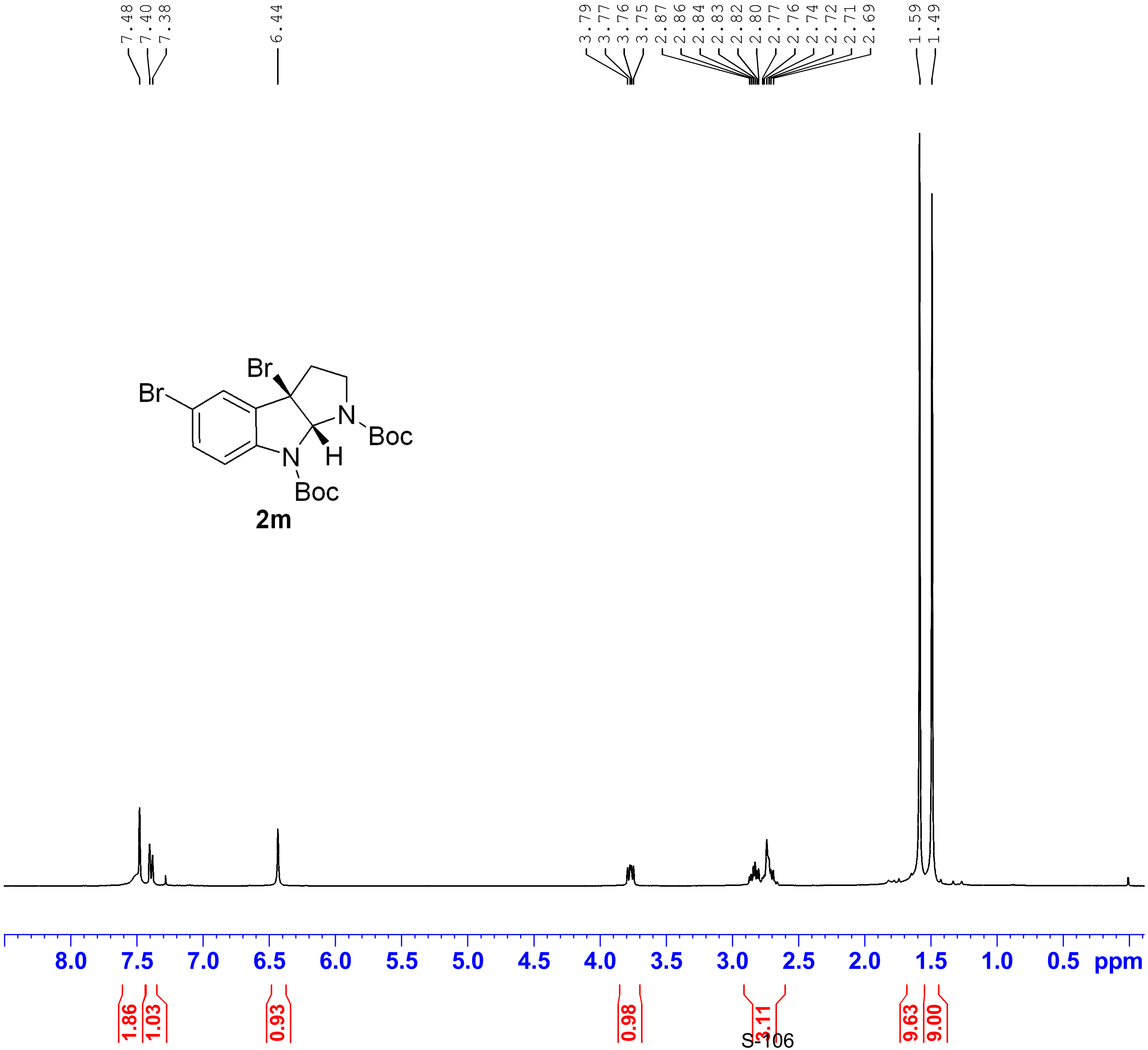

Current Data Parameters
NAME txf-3-88nmr
EXPNO 1
PROCNO 1

F2 - Acquisition Parameters
Date_ 20211224
Time 9.14
INSTRUM spect
PROBHD 5 mm PABBO BB/
PULPROG zg30
TD 65536
SOLVENT CDCl3
NS 4
DS 0
SWH 8012.820 Hz
FIDRES 0.122266 Hz
AQ 4.0894465 sec
RG 49.32
DW 62.400 usec
DE 6.50 usec
TE 296.3 K
D1 1.00000000 sec
TD0 1

======== CHANNEL f1 ========
SFO1 400.1324710 MHz
NUC1 1H
P1 14.50 usec
PLW1 11.99499989 W

F2 - Processing parameters
SI 65536
SF 400.1300000 MHz
WDW EM
SSB 0
LB 0.30 Hz
GB 0
PC 1.00

Supplementary Figure 51. ^{1}H NMR spectrum of **2m** (400 MHz, r.t., $CDCl_3$)

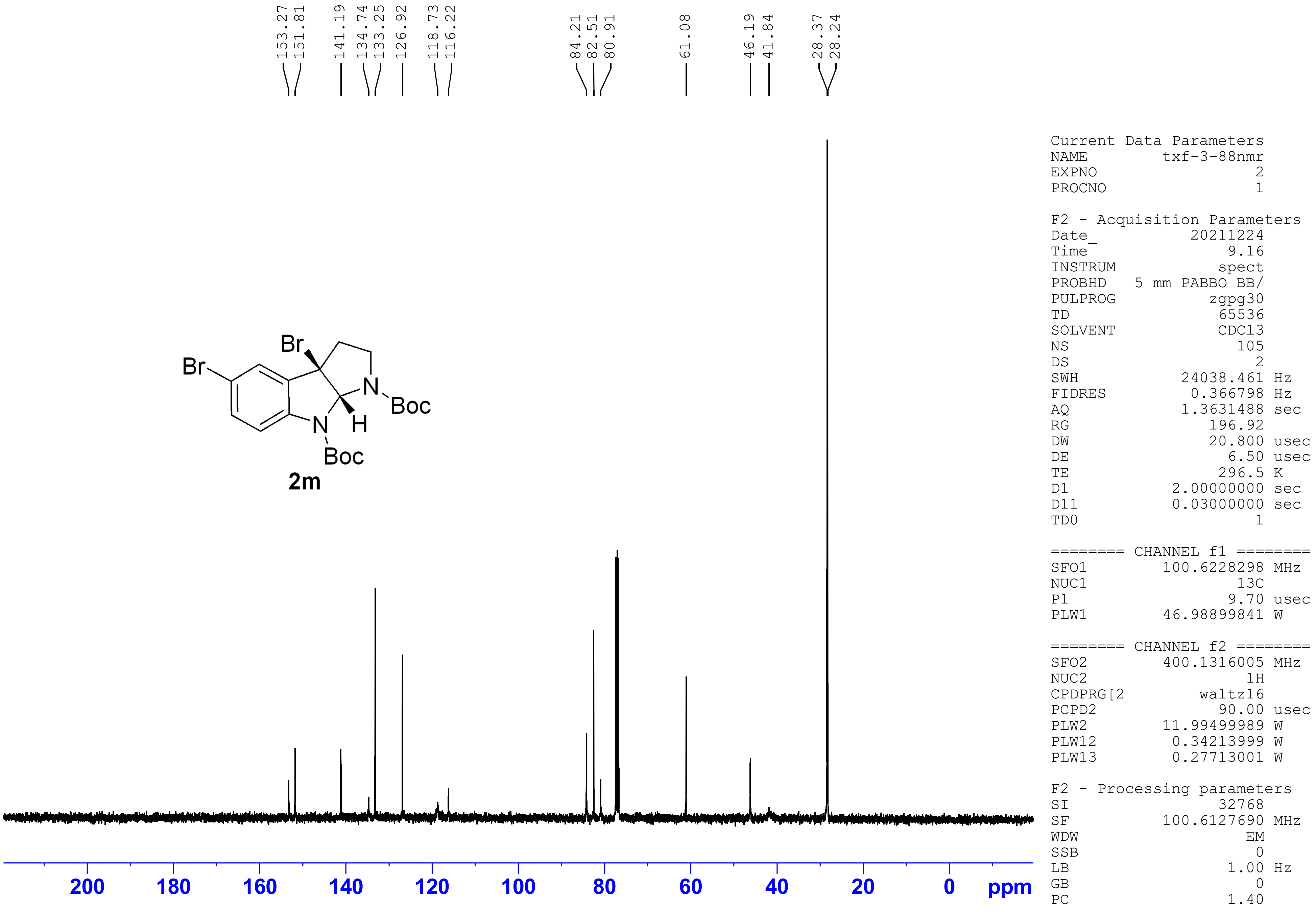

Supplementary Figure 52. ^{13}C NMR spectrum of **2m** (100 MHz, r.t., $CDCl_3$)

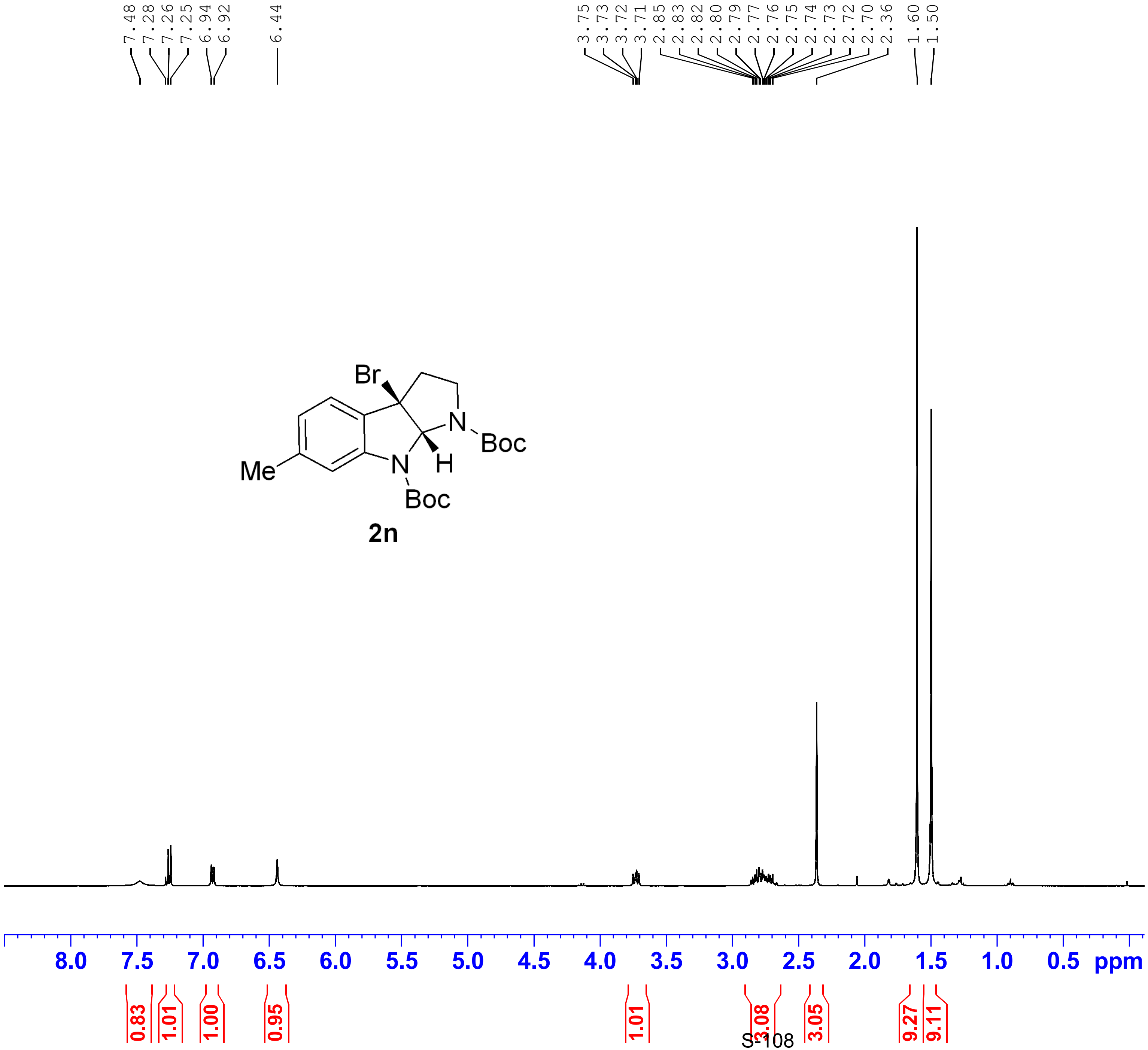


```
Current Data Parameters
NAME        txf-3-134nmr
EXPNO                  1
PROCNO                 1

F2 - Acquisition Parameters
Date_           20220121
Time               22.30
INSTRUM            spect
PROBHD   5 mm PABBO BB/
PULPROG             zg30
TD                 65536
SOLVENT            CDCl3
NS                     4
DS                     0
SWH             8012.820 Hz
FIDRES          0.122266 Hz
AQ             4.0894465 sec
RG                 39.46
DW                62.400 usec
DE                  6.50 usec
TE                 295.4 K
D1            1.00000000 sec
TD0                    1

======== CHANNEL f1 ========
SFO1         400.1324710 MHz
NUC1                  1H
P1                 14.50 usec
PLW1         11.99499989 W

F2 - Processing parameters
SI                 65536
SF           400.1300000 MHz
WDW                   EM
SSB                    0
LB                  0.30 Hz
GB                     0
PC                  1.00
```


Supplementary Figure 53. ^{1}H NMR spectrum of **2n** (400 MHz, r.t., $CDCl_3$)

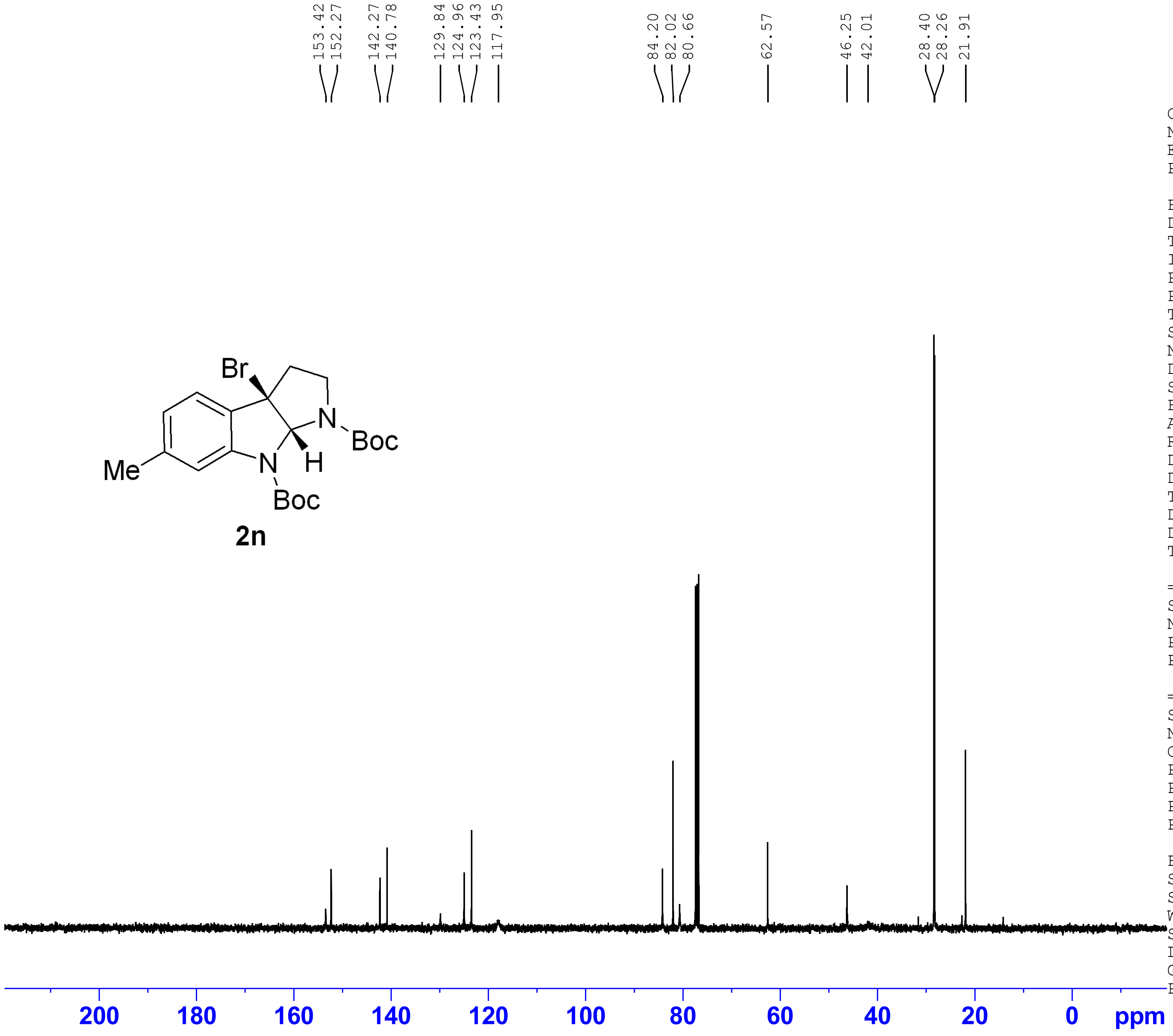

2n

```
Current Data Parameters
NAME        txf-3-134nmr
EXPNO                  2
PROCNO                 1

F2 - Acquisition Parameters
Date_           20220121
Time               22.32
INSTRUM            spect
PROBHD    5 mm PABBO BB/
PULPROG          zgpg30
TD                 65536
SOLVENT            CDCl3
NS                   104
DS                     2
SWH            24038.461 Hz
FIDRES          0.366798 Hz
AQ             1.3631488 sec
RG                196.92
DW                20.800 usec
DE                  6.50 usec
TE                 295.8 K
D1            2.00000000 sec
D11           0.03000000 sec
TD0                    1

======== CHANNEL f1 ========
SFO1         100.6228298 MHz
NUC1                 13C
P1                  9.70 usec
PLW1         46.98899841 W

======== CHANNEL f2 ========
SFO2         400.1316005 MHz
NUC2                  1H
CPDPRG[2         waltz16
PCPD2              90.00 usec
PLW2         11.99499989 W
PLW12         0.34213999 W
PLW13         0.27713001 W

F2 - Processing parameters
SI                 32768
SF           100.6127690 MHz
WDW                   EM
SSB                    0
LB                  1.00 Hz
GB                     0
PC                  1.40
```


Supplementary Figure 54. ^{13}C NMR spectrum of **2n** (100 MHz, r.t., $CDCl_3$)

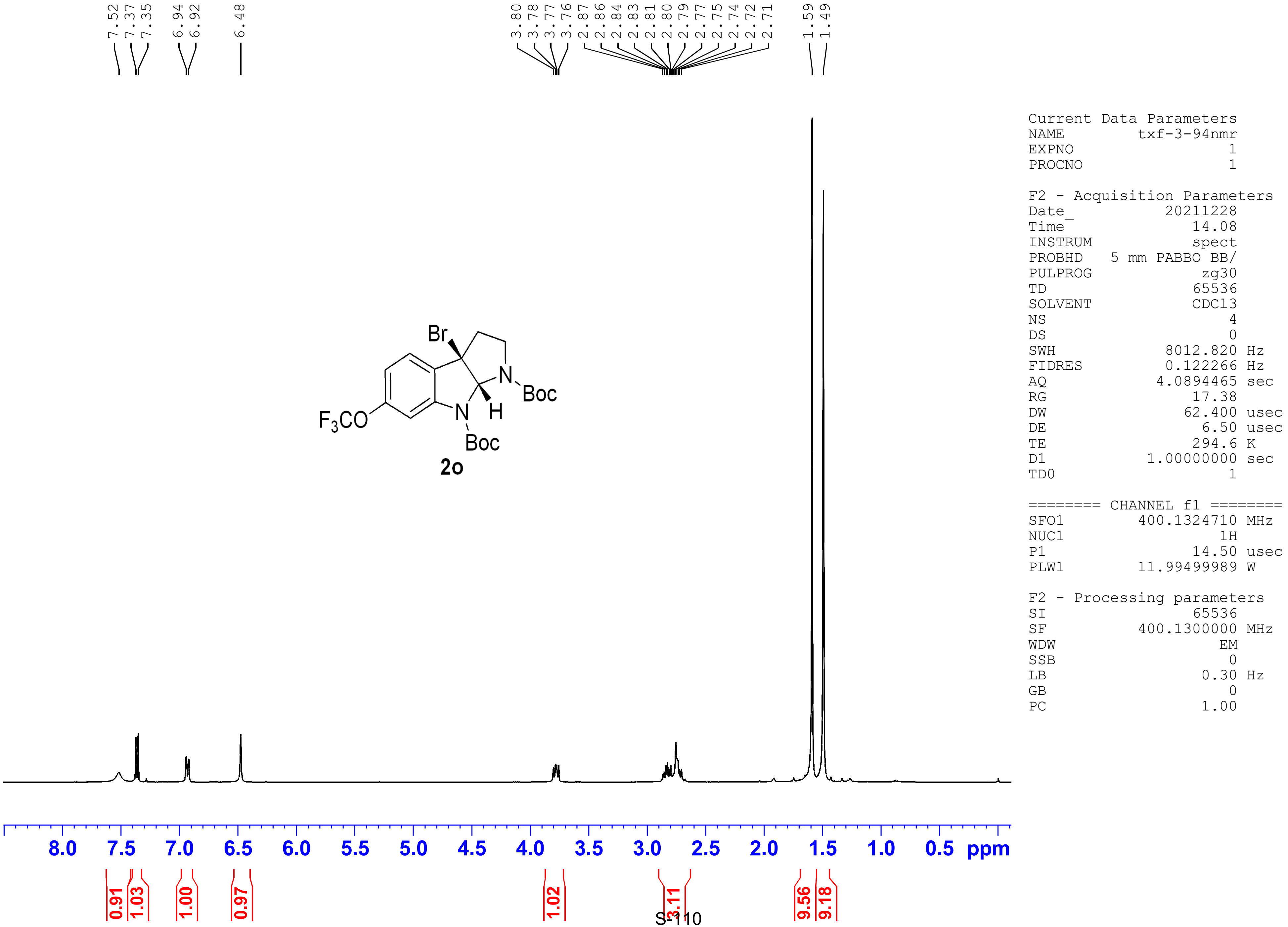

Supplementary Figure 55. ^{1}H NMR spectrum of **2o** (400 MHz, r.t., $CDCl_3$)

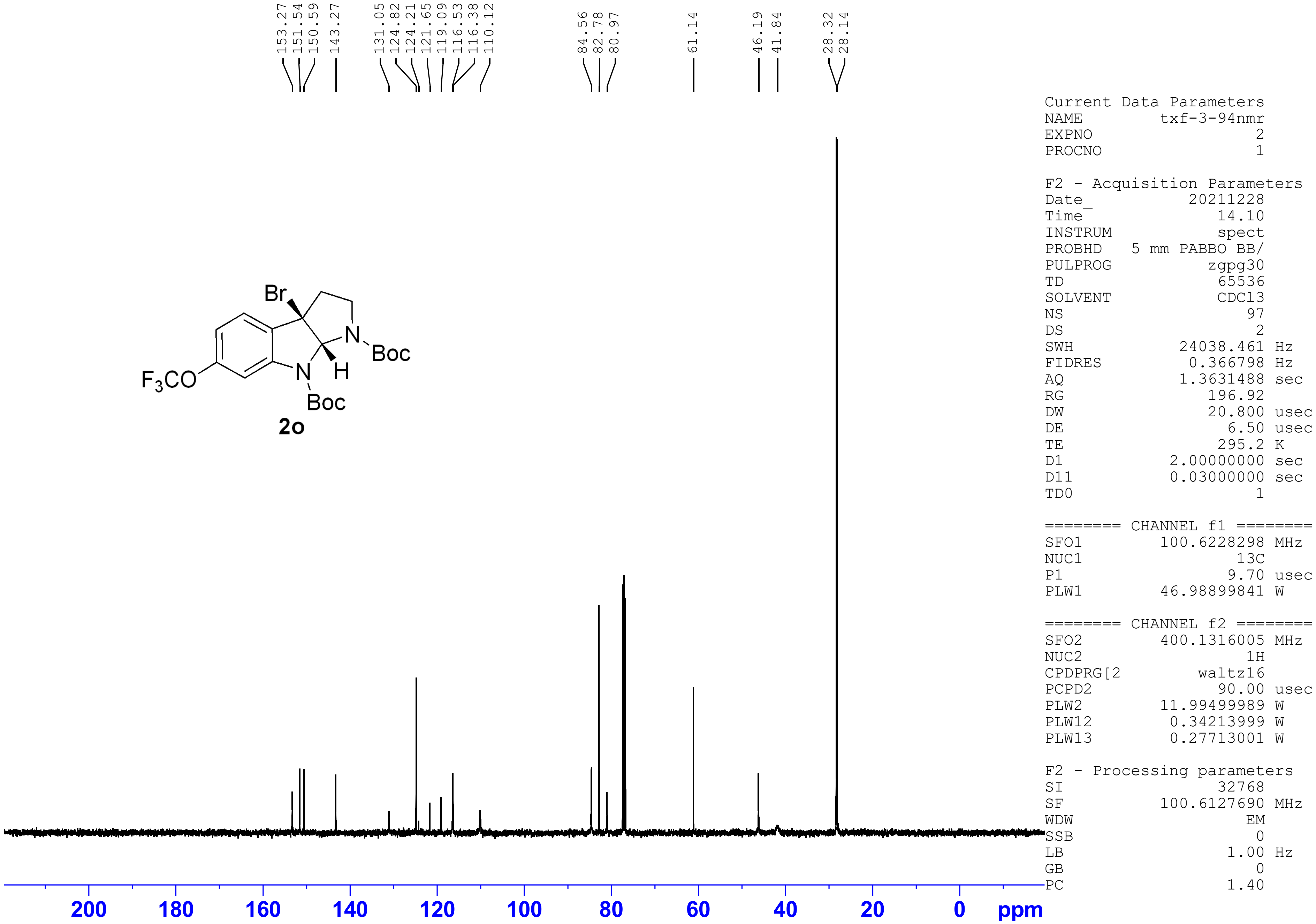

Supplementary Figure 56. ^{13}C NMR spectrum of **2o** (100 MHz, r.t., $CDCl_3$)

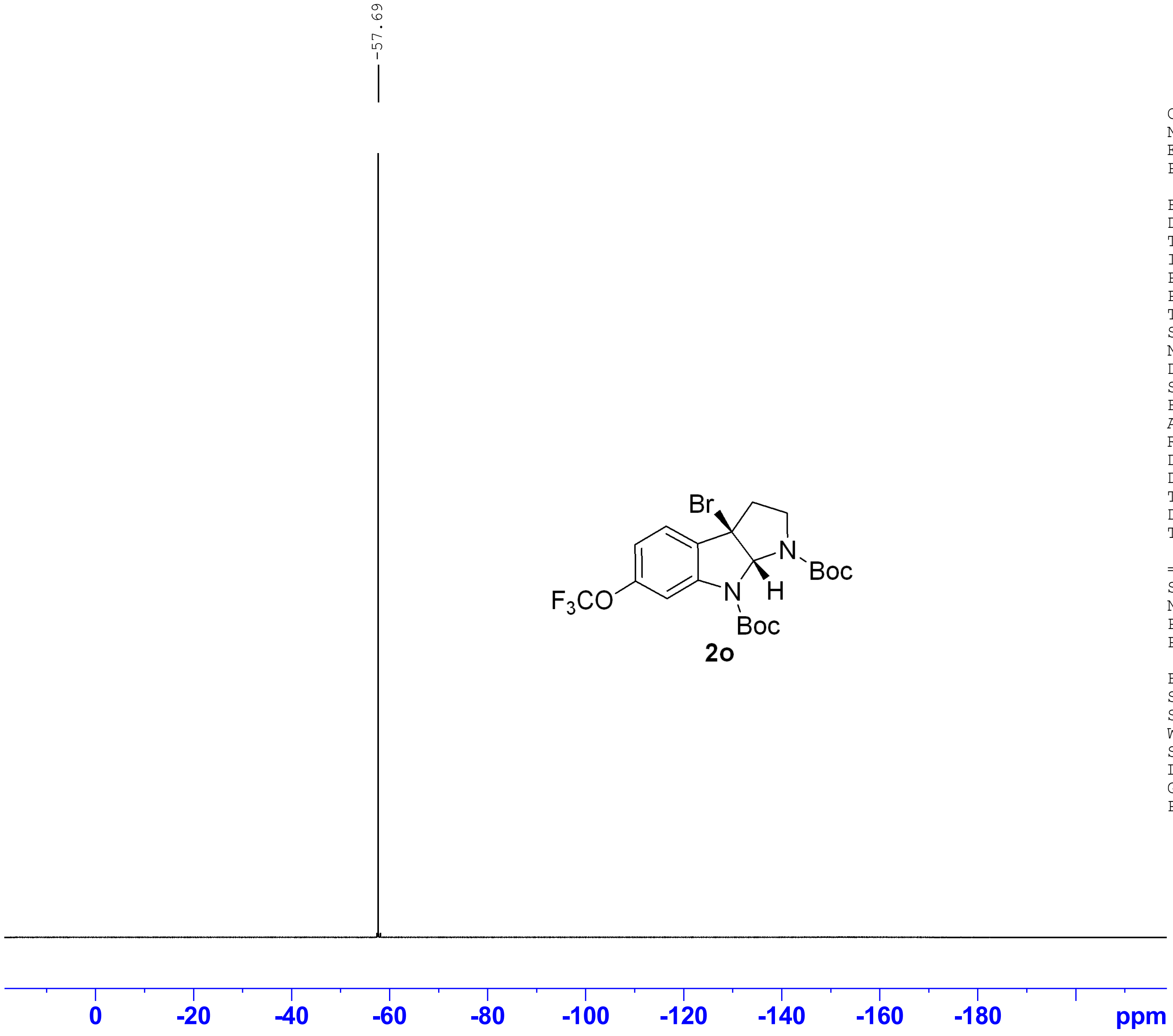


```
Current Data Parameters
NAME          txf-3-94nmr
EXPNO                   3
PROCNO                  1

F2 - Acquisition Parameters
Date_            20211228
Time                14.16
INSTRUM             spect
PROBHD   5 mm PABBO BB/
PULPROG            zgflqn
TD                 131072
SOLVENT             CDCl3
NS                      6
DS                      4
SWH            89285.711 Hz
FIDRES          0.681196 Hz
AQ             0.7340032 sec
RG                 196.92
DW                  5.600 usec
DE                   6.50 usec
TE                  294.9 K
D1             1.00000000 sec
TD0                     1

======== CHANNEL f1 ========
SFO1         376.4607164 MHz
NUC1                  19F
P1                  14.70 usec
PLW1         15.99600029 W

F2 - Processing parameters
SI                  65536
SF           376.4983660 MHz
WDW                    EM
SSB                     0
LB                   0.30 Hz
GB                      0
PC                   1.00
```


Supplementary Figure 57. ^{19}F NMR spectrum of **2o** (376 MHz, r.t., $CDCl_3$)

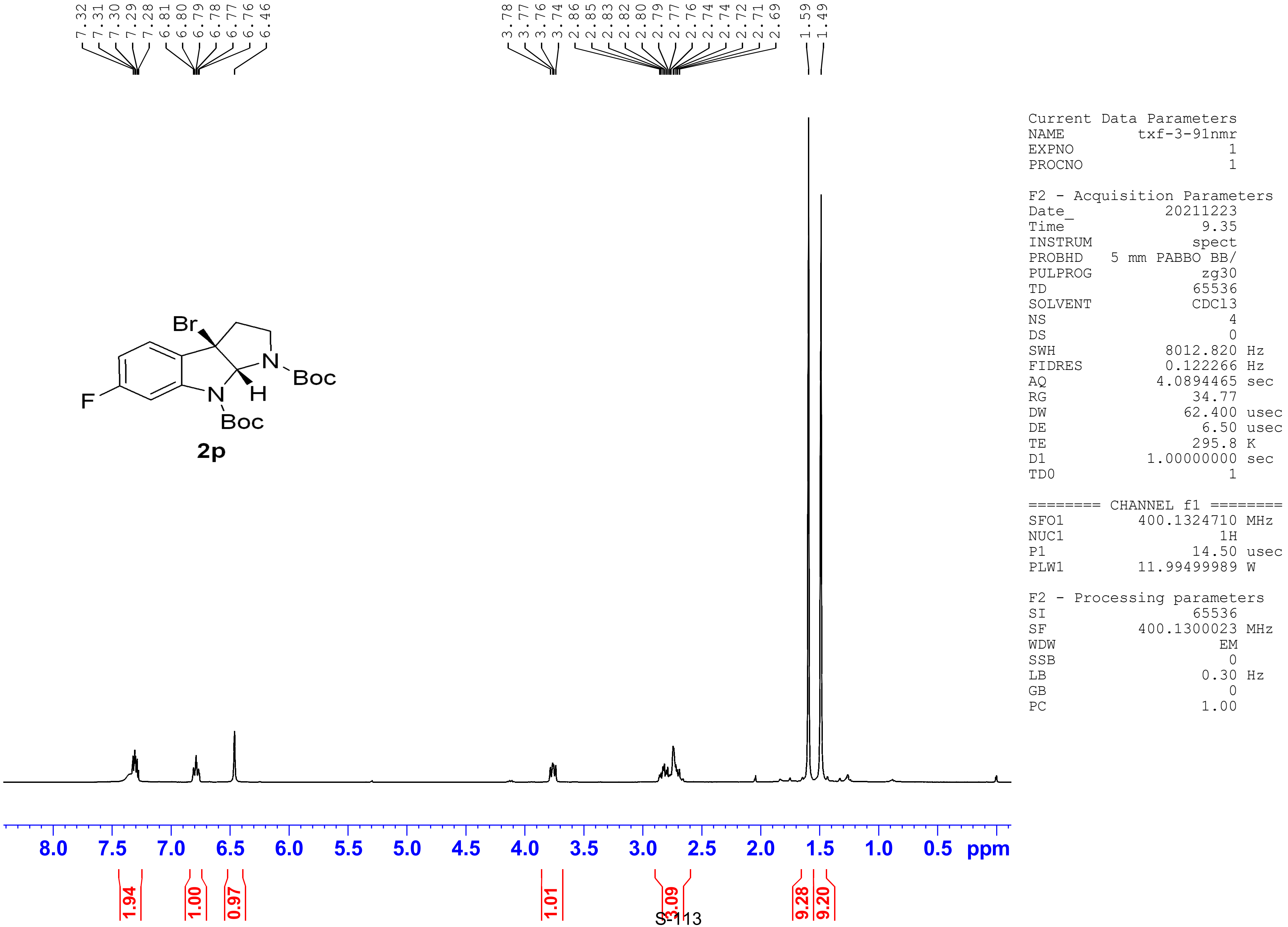

Supplementary Figure 58. ^{1}H NMR spectrum of **2p** (400 MHz, r.t., $CDCl_3$)

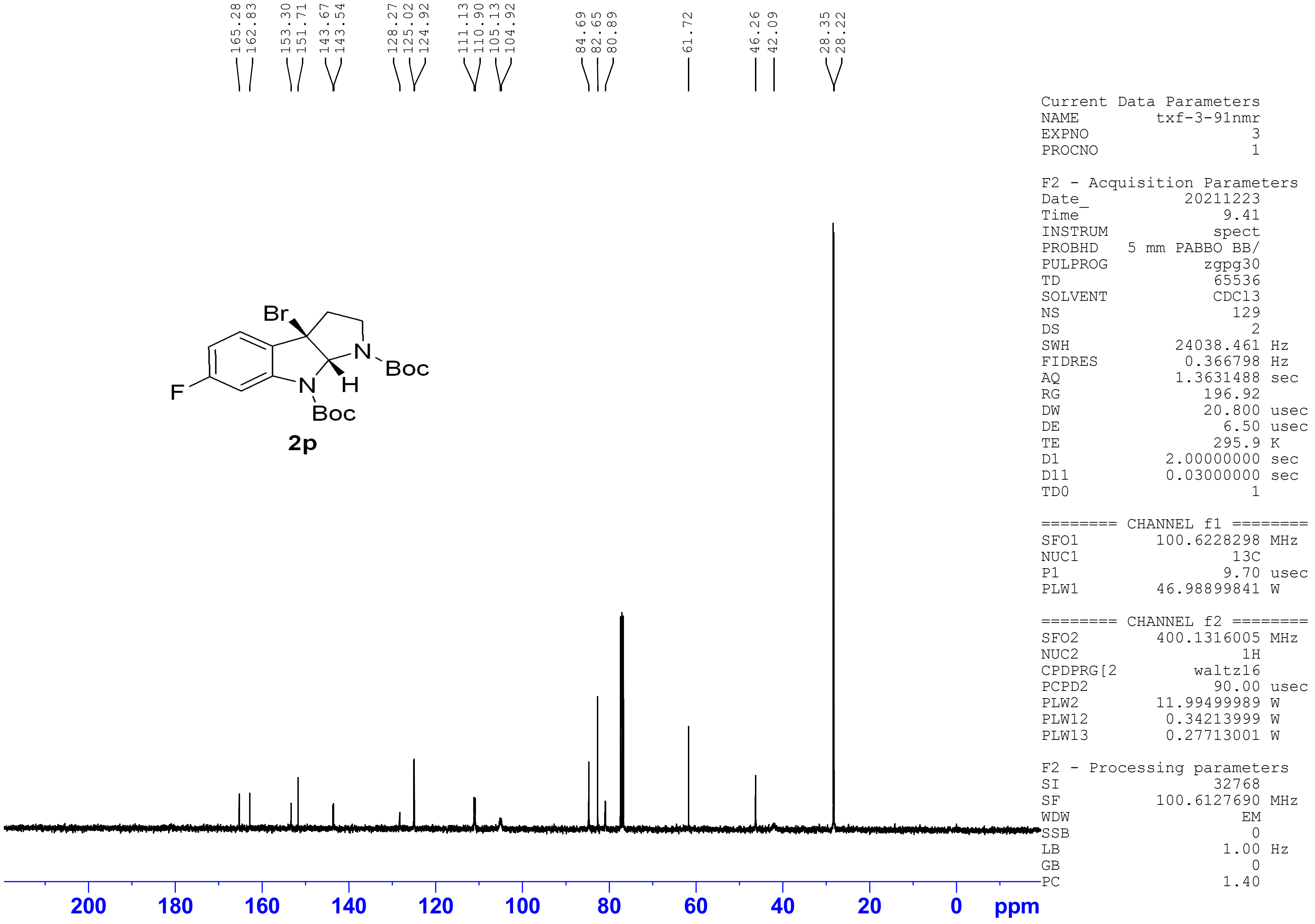

Supplementary Figure 59. ^{13}C NMR spectrum of **2p** (100 MHz, r.t., $CDCl_3$)

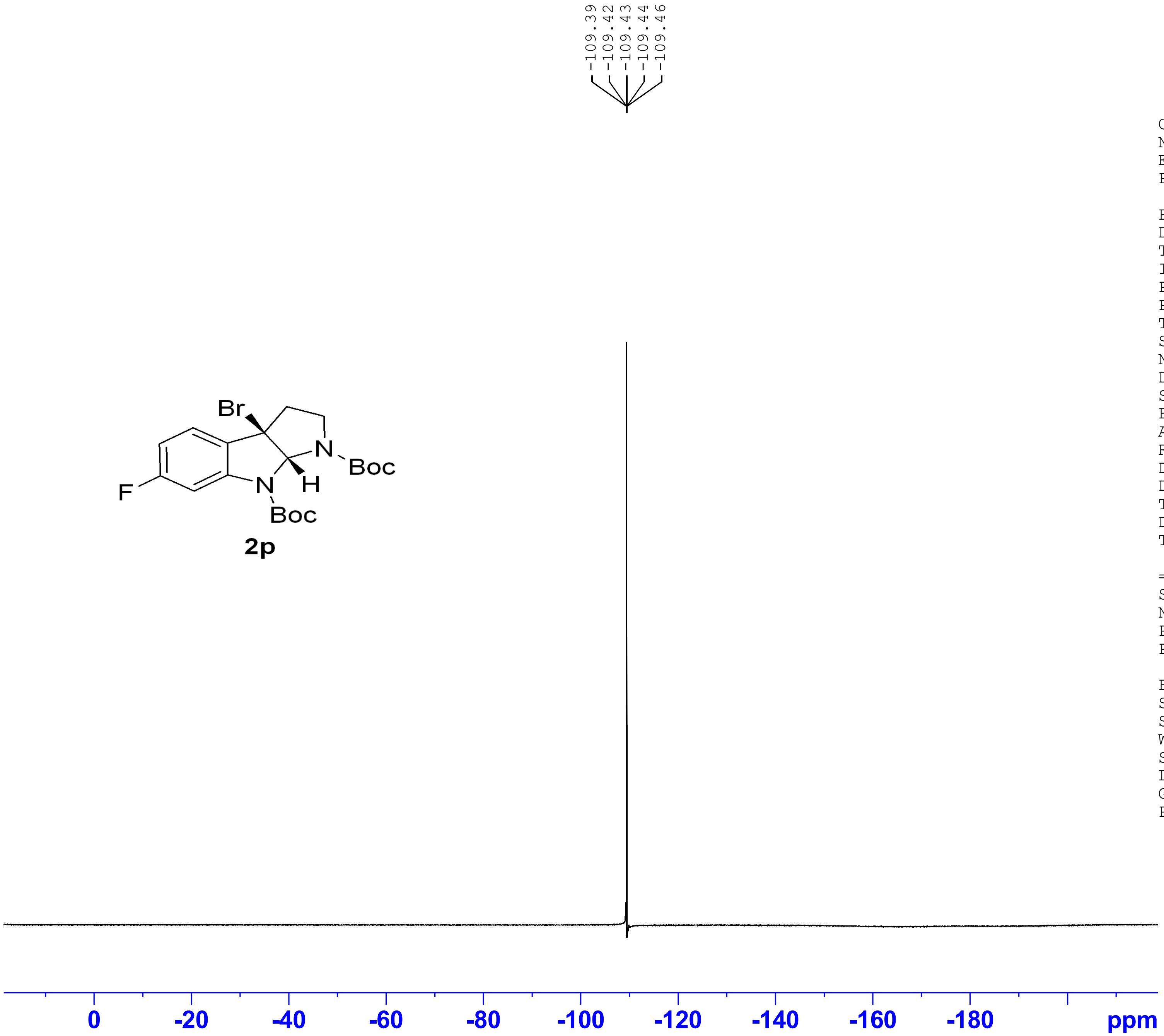


```
Current Data Parameters
NAME         txf-3-91nmr
EXPNO                  2
PROCNO                 1

F2 - Acquisition Parameters
Date_           20211223
Time                9.36
INSTRUM            spect
PROBHD   5 mm PABBO BB/
PULPROG           zgflqn
TD                131072
SOLVENT            CDCl3
NS                    16
DS                     4
SWH           89285.711 Hz
FIDRES         0.681196 Hz
AQ            0.7340032 sec
RG               196.92
DW                5.600 usec
DE                 6.50 usec
TE                295.7 K
D1           1.00000000 sec
TD0                    1

======== CHANNEL f1 ========
SFO1       376.4607164 MHz
NUC1               19F
P1               14.70 usec
PLW1       15.99600029 W

F2 - Processing parameters
SI                 65536
SF          376.4983660 MHz
WDW                   EM
SSB                    0
LB                  0.30 Hz
GB                     0
PC                  1.00
```


Supplementary Figure 60. ^{19}F NMR spectrum of **2p** (376 MHz, r.t., $CDCl_3$)

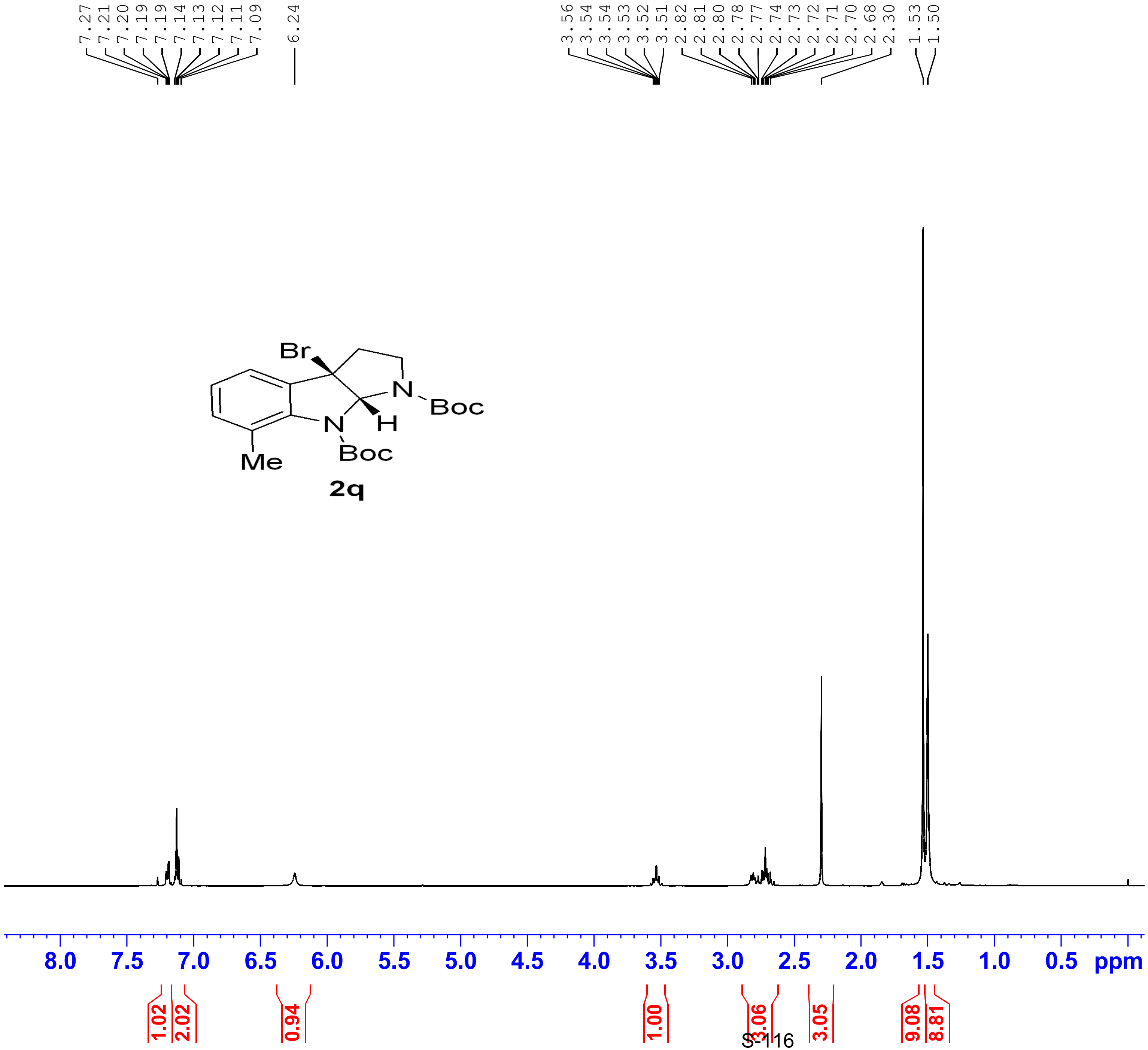


```
Current Data Parameters
NAME        txf-3-100nmr
EXPNO                  1
PROCNO                 1

F2 - Acquisition Parameters
Date_           20220302
Time               21.06
INSTRUM            spect
PROBHD   5 mm PABBO BB/
PULPROG             zg30
TD                 65536
SOLVENT            CDCl3
NS                     4
DS                     0
SWH             8012.820 Hz
FIDRES          0.122266 Hz
AQ             4.0894465 sec
RG                 22.47
DW                62.400 usec
DE                  6.50 usec
TE                 295.0 K
D1            1.00000000 sec
TD0                    1

======== CHANNEL f1 ========
SFO1         400.1324710 MHz
NUC1                  1H
P1                 14.50 usec
PLW1         11.99499989 W

F2 - Processing parameters
SI                 65536
SF           400.1300056 MHz
WDW                   EM
SSB                    0
LB                  0.30 Hz
GB                     0
PC                  1.00
```


Supplementary Figure 61. ^{1}H NMR spectrum of **2q** (400 MHz, r.t., $CDCl_3$)

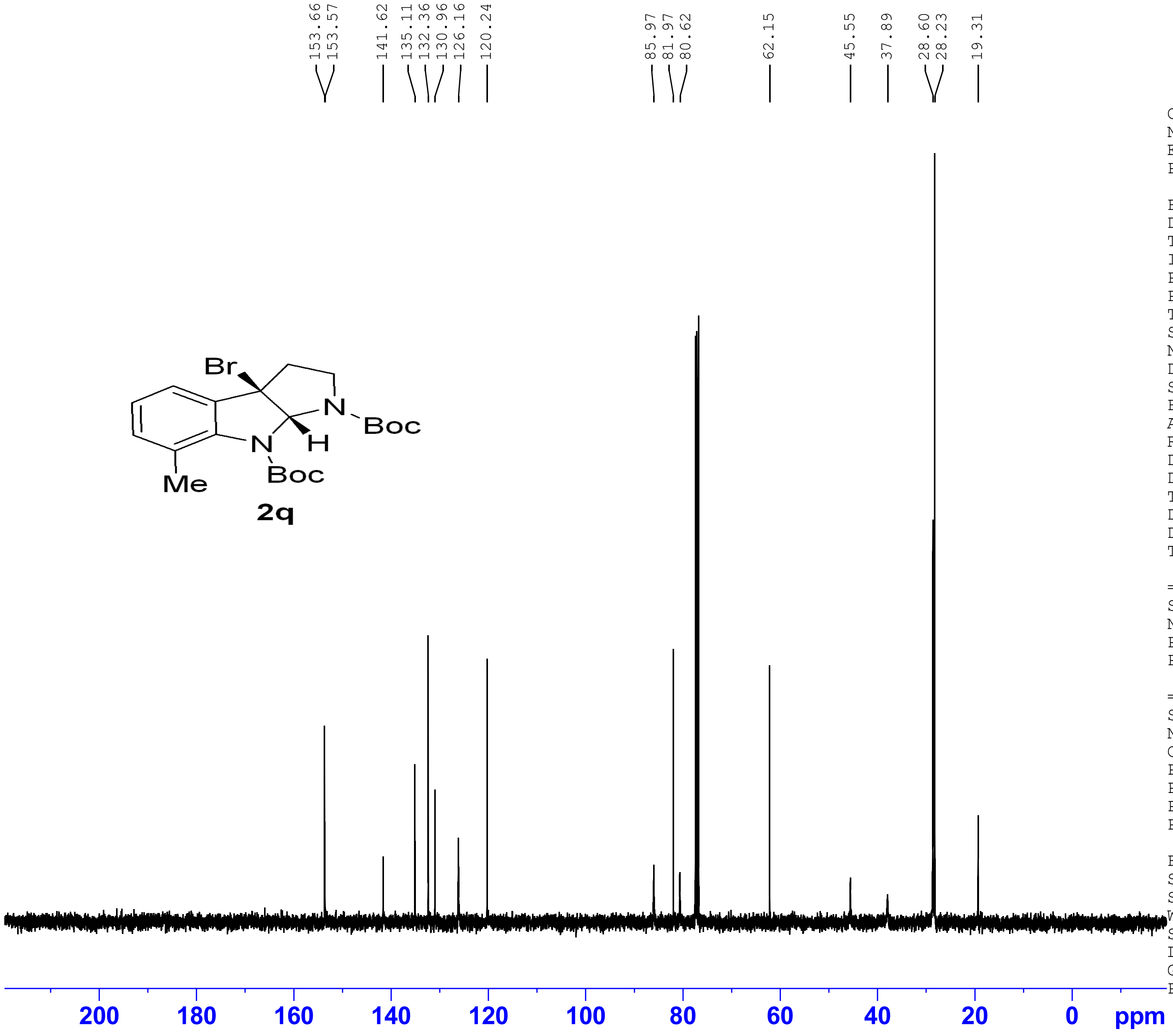

Current Data Parameters
NAME txf-3-100nmr
EXPNO 2
PROCNO 1

F2 - Acquisition Parameters
Date_ 20220302
Time 21.20
INSTRUM spect
PROBHD 5 mm PABBO BB/
PULPROG zgpg30
TD 65536
SOLVENT CDCl3
NS 75
DS 2
SWH 24038.461 Hz
FIDRES 0.366798 Hz
AQ 1.3631488 sec
RG 196.92
DW 20.800 usec
DE 6.50 usec
TE 295.6 K
D1 2.00000000 sec
D11 0.03000000 sec
TD0 1

======== CHANNEL f1 ========
SFO1 100.6228298 MHz
NUC1 13C
P1 9.70 usec
PLW1 46.98899841 W

======== CHANNEL f2 ========
SFO2 400.1316005 MHz
NUC2 1H
CPDPRG[2 waltz16
PCPD2 90.00 usec
PLW2 11.99499989 W
PLW12 0.34213999 W
PLW13 0.27713001 W

F2 - Processing parameters
SI 32768
SF 100.6127690 MHz
WDW EM
SSB 0
LB 1.00 Hz
GB 0
PC 1.40

Supplementary Figure 62. ^{13}C NMR spectrum of **2q** (100 MHz, r.t., $CDCl_3$)

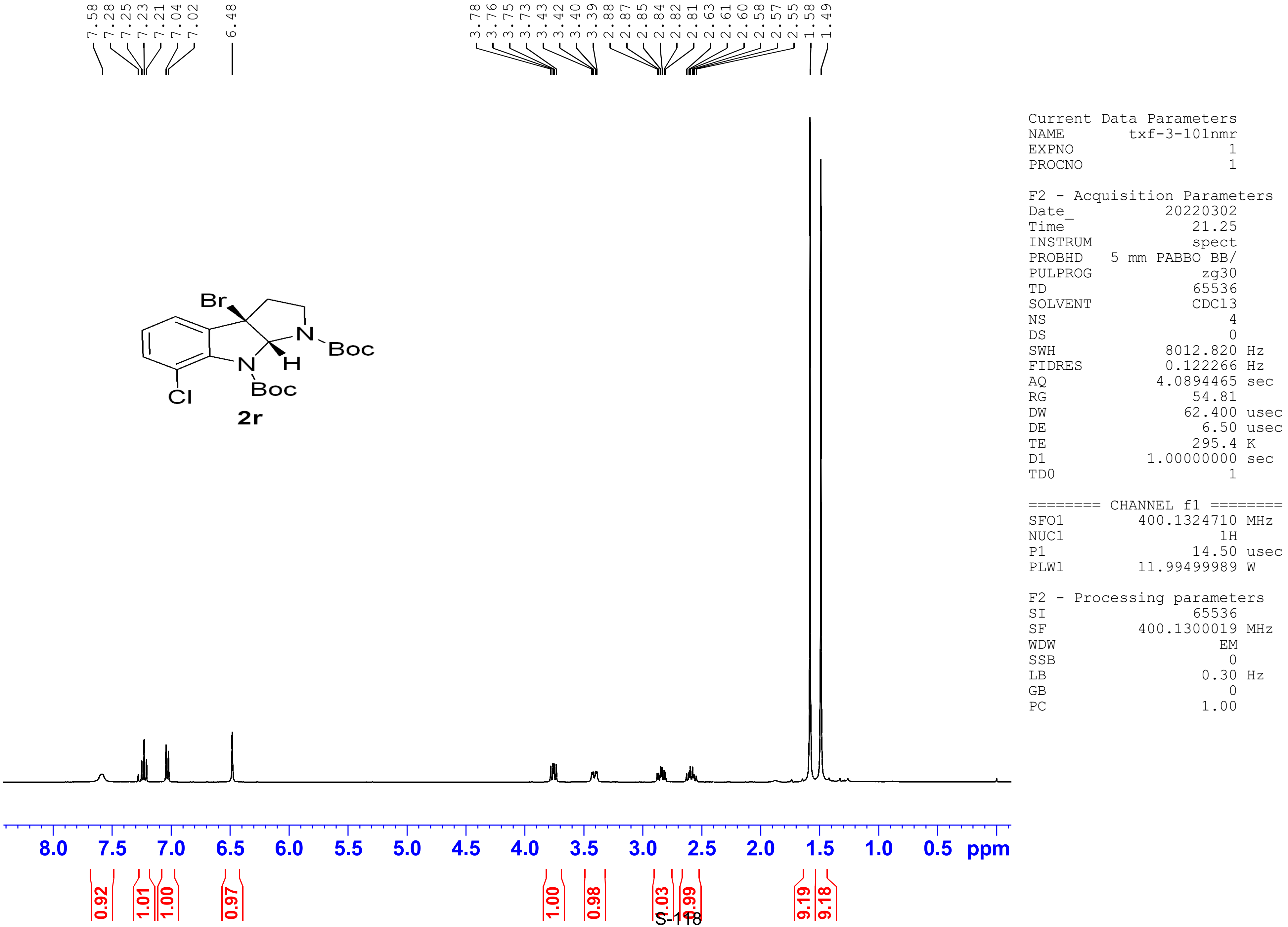

Supplementary Figure 63. ^{1}H NMR spectrum of **2r** (400 MHz, r.t., $CDCl_3$)

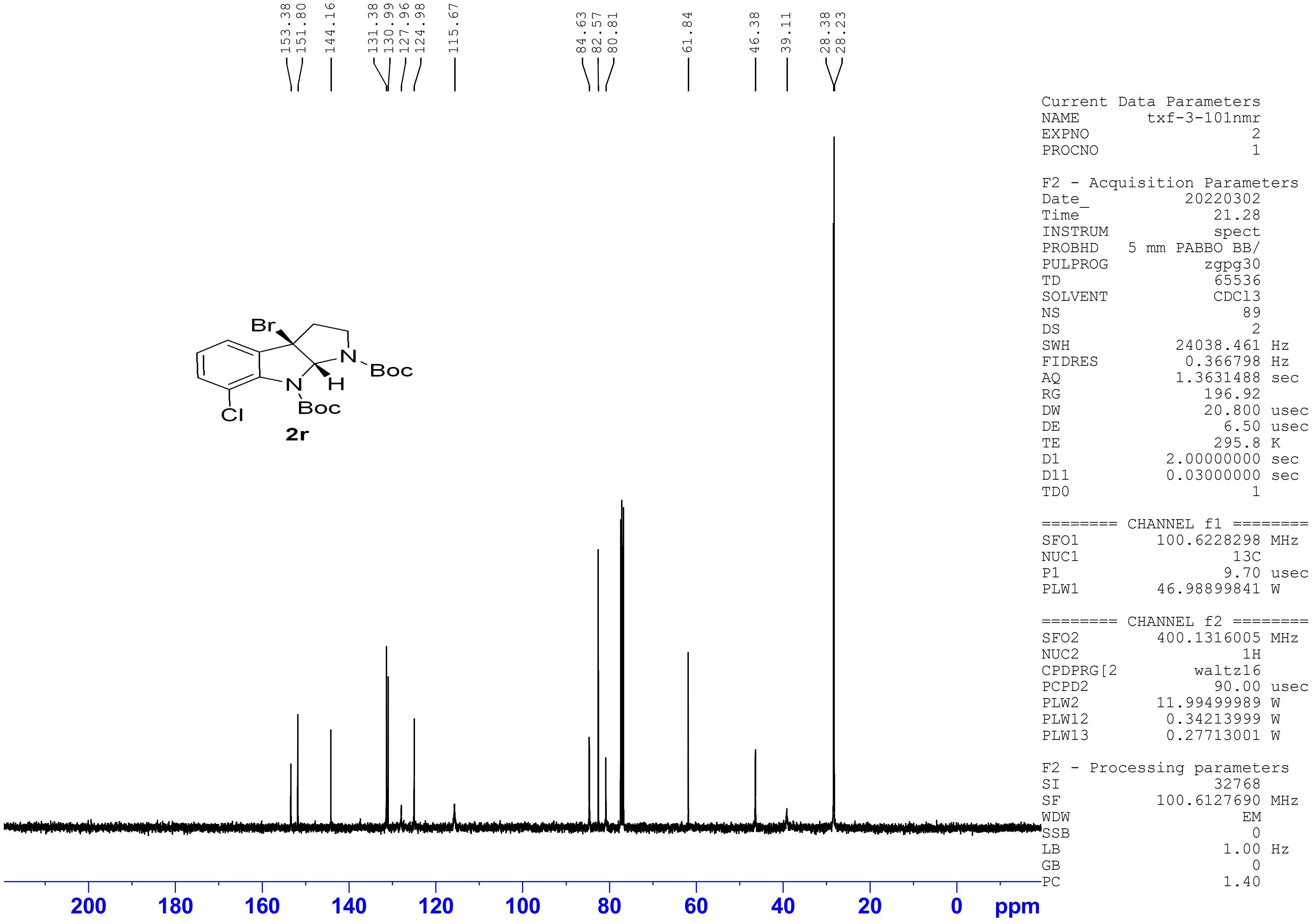

Supplementary Figure 64. ^{13}C NMR spectrum of **2r** (100 MHz, r.t., $CDCl_3$)

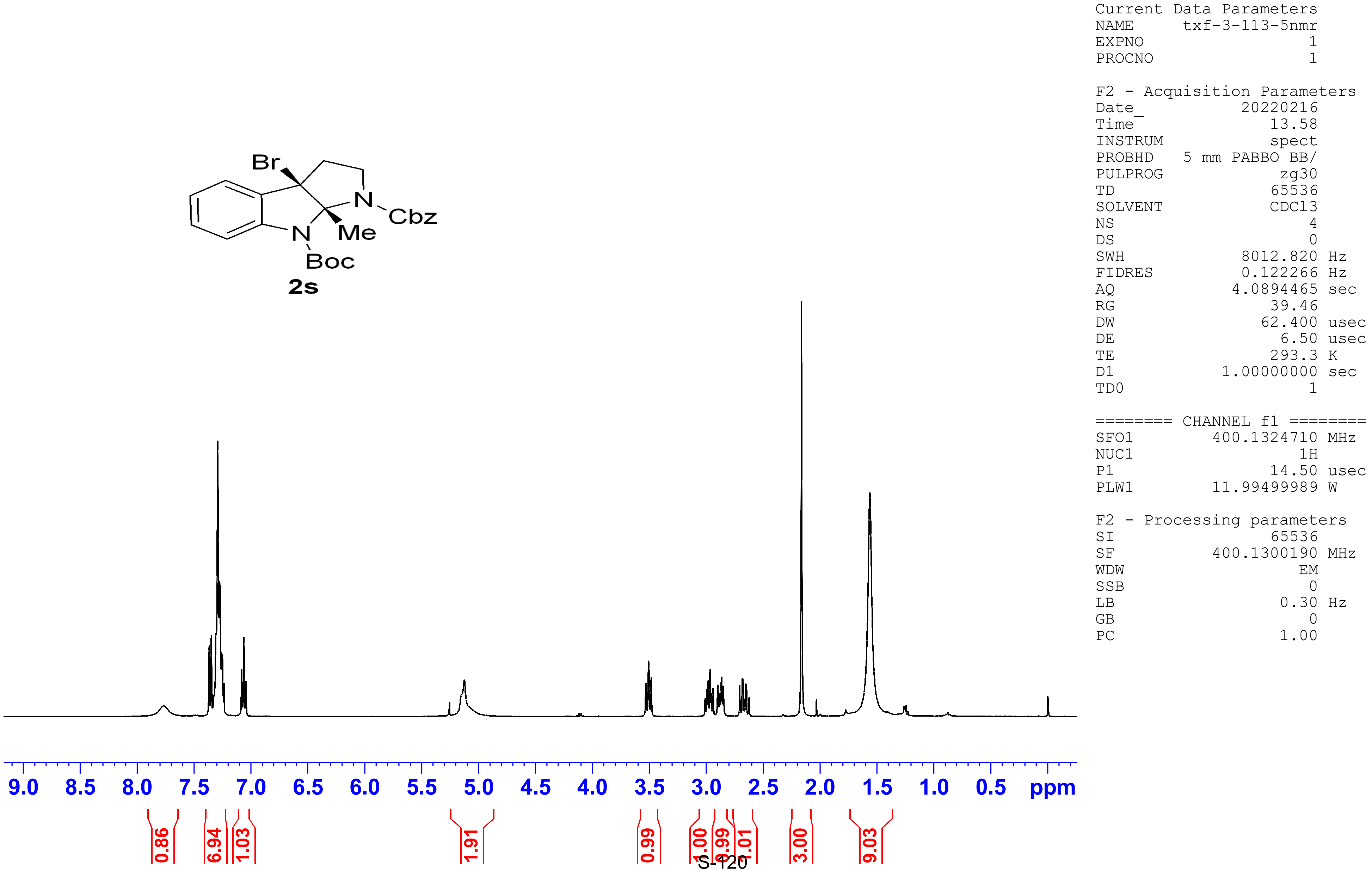

Supplementary Figure 65. ^{1}H NMR spectrum of **2s** (400 MHz, r.t., $CDCl_3$)

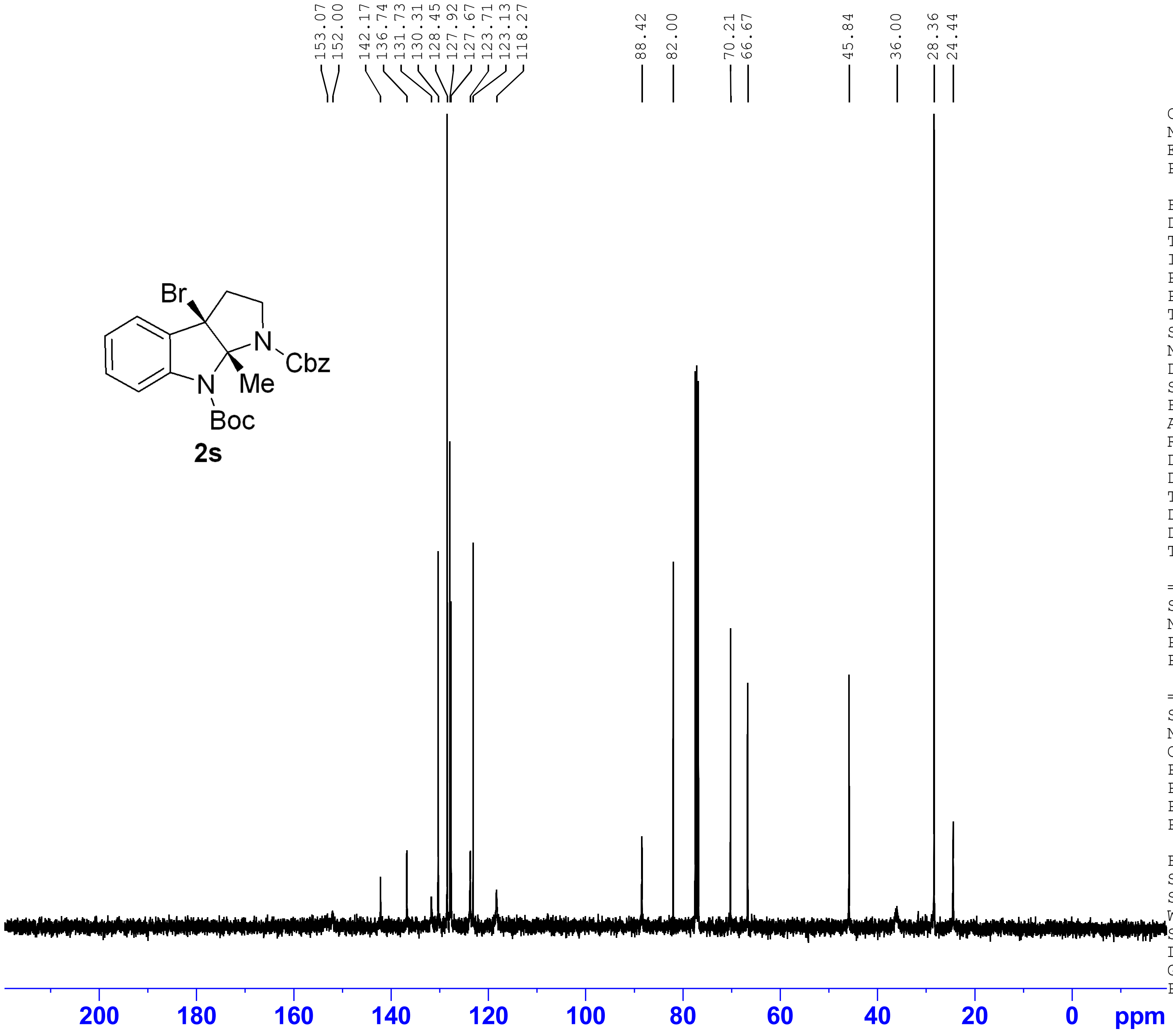


```
Current Data Parameters
NAME      txf-3-113-5nmr
EXPNO                  2
PROCNO                 1

F2 - Acquisition Parameters
Date_           20220216
Time               14.00
INSTRUM            spect
PROBHD   5 mm PABBO BB/
PULPROG          zgpg30
TD                 65536
SOLVENT            CDCl3
NS                   119
DS                     2
SWH            24038.461 Hz
FIDRES          0.366798 Hz
AQ             1.3631488 sec
RG                196.92
DW                20.800 usec
DE                  6.50 usec
TE                 293.7 K
D1            2.00000000 sec
D11           0.03000000 sec
TD0                    1

======== CHANNEL f1 ========
SFO1         100.6228298 MHz
NUC1                 13C
P1                  9.70 usec
PLW1         46.98899841 W

======== CHANNEL f2 ========
SFO2         400.1316005 MHz
NUC2                  1H
CPDPRG[2         waltz16
PCPD2              90.00 usec
PLW2         11.99499989 W
PLW12         0.34213999 W
PLW13         0.27713001 W

F2 - Processing parameters
SI                 32768
SF           100.6127690 MHz
WDW                   EM
SSB                    0
LB                  1.00 Hz
GB                     0
PC                  1.40
```


Supplementary Figure 66. ^{13}C NMR spectrum of **2s** (100 MHz, r.t., $CDCl_3$)

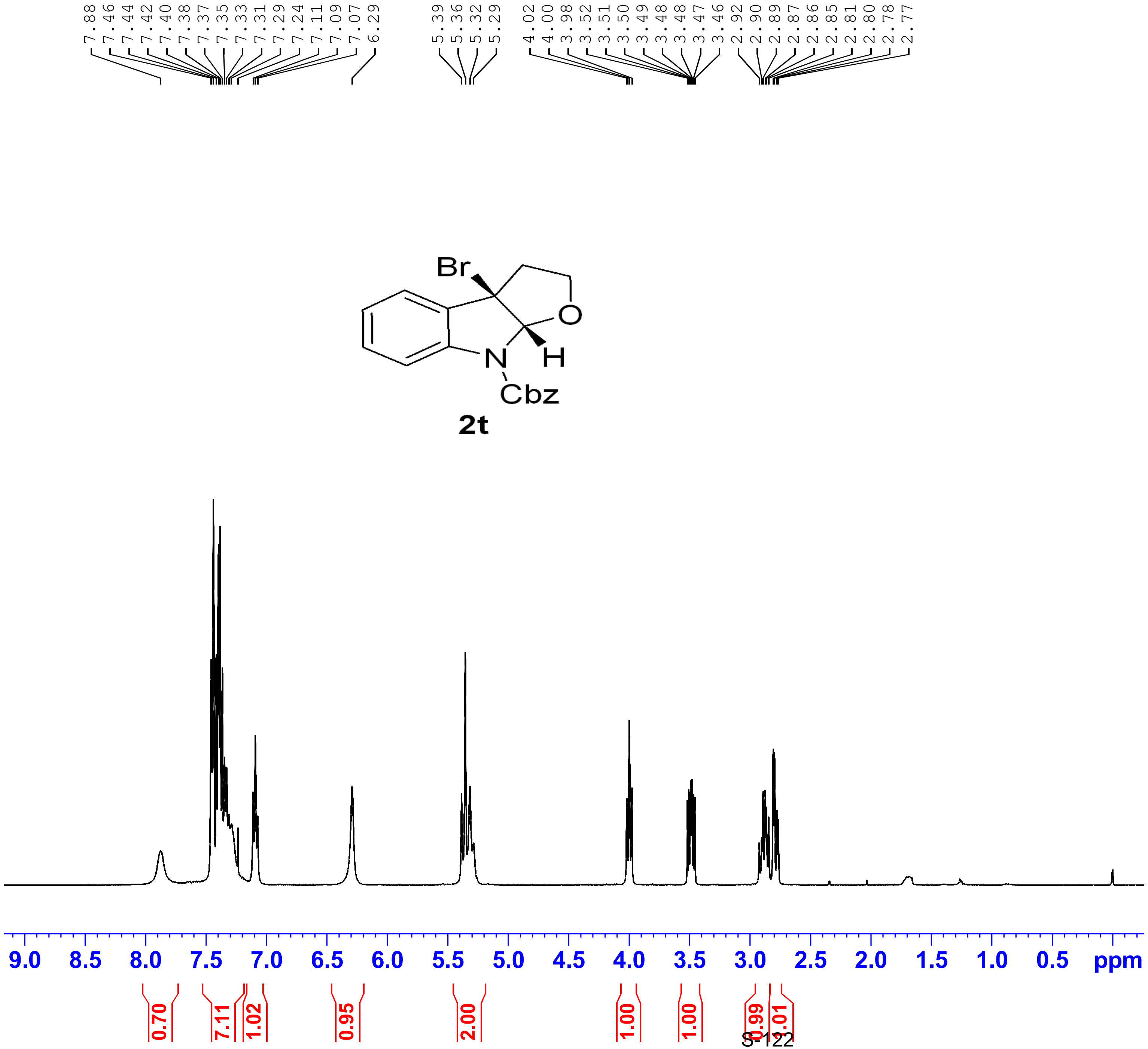


```
Current Data Parameters
NAME          txf-3-108nmr
EXPNO                    1
PROCNO                   1

F2 - Acquisition Parameters
Date_             20220104
Time                  9.20
INSTRUM              spect
PROBHD     5 mm PABBO BB/
PULPROG               zg30
TD                   65536
SOLVENT              CDCl3
NS                       4
DS                       0
SWH             8012.820 Hz
FIDRES          0.122266 Hz
AQ             4.0894465 sec
RG                   45.67
DW                  62.400 usec
DE                    6.50 usec
TE                   295.9 K
D1            1.00000000 sec
TD0                      1

======== CHANNEL f1 ========
SFO1         400.1324710 MHz
NUC1                    1H
P1                   14.50 usec
PLW1           11.99499989 W

F2 - Processing parameters
SI                   65536
SF           400.1300194 MHz
WDW                     EM
SSB                      0
LB                    0.30 Hz
GB                       0
PC                    1.00
```


Supplementary Figure 67. ^{1}H NMR spectrum of **2t** (400 MHz, r.t., $CDCl_3$)

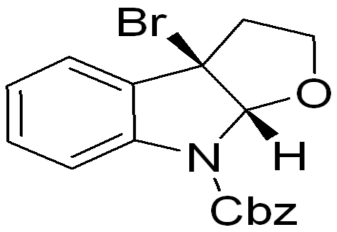

2t

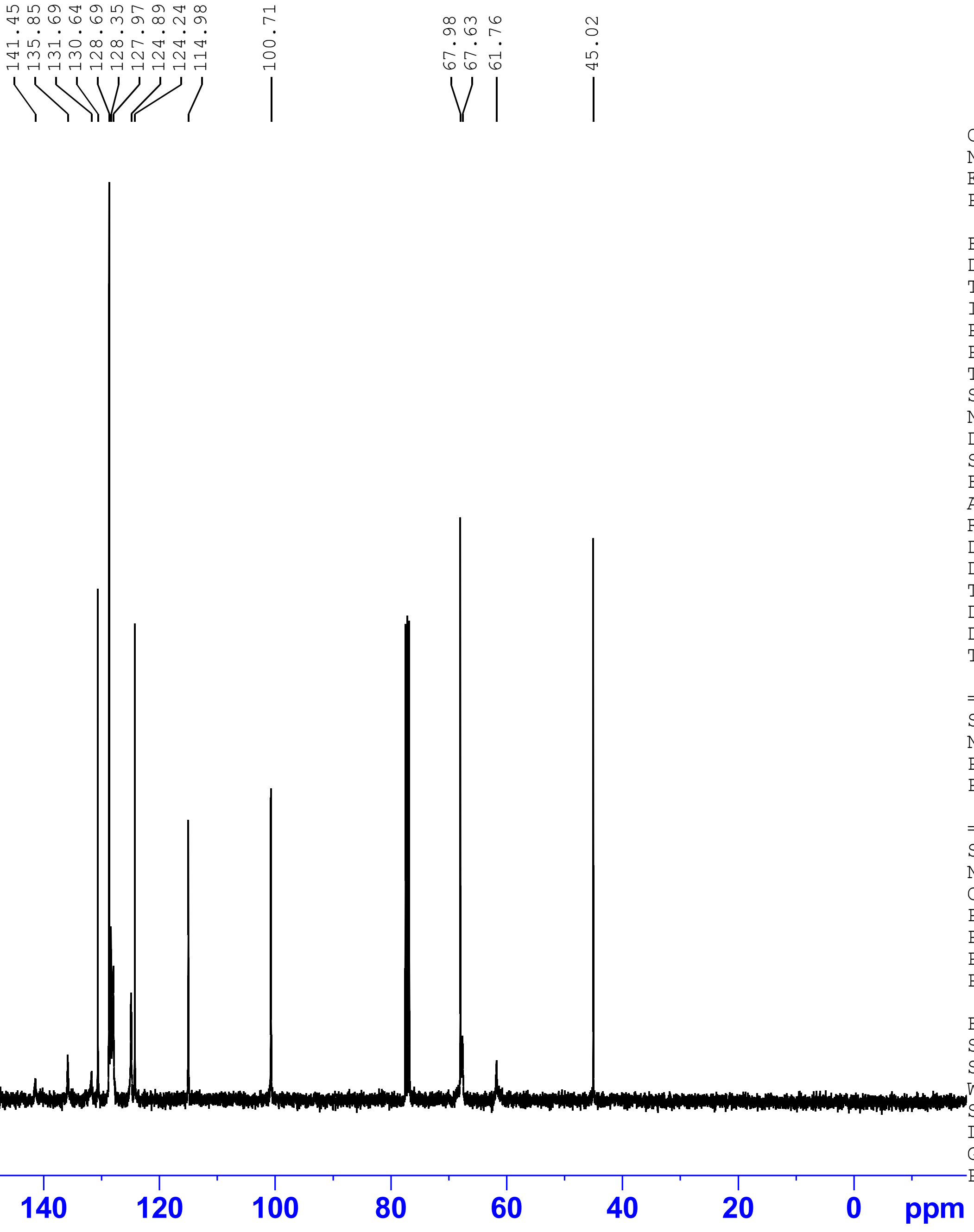

Current Data Parameters
NAME txf-3-108nmr
EXPNO 2
PROCNO 1

F2 - Acquisition Parameters
Date_ 20220104
Time 9.22
INSTRUM spect
PROBHD 5 mm PABBO BB/
PULPROG zgpg30
TD 65536
SOLVENT CDCl3
NS 196
DS 2
SWH 24038.461 Hz
FIDRES 0.366798 Hz
AQ 1.3631488 sec
RG 196.92
DW 20.800 usec
DE 6.50 usec
TE 296.6 K
D1 2.00000000 sec
D11 0.03000000 sec
TD0 1

======== CHANNEL f1 ========
SFO1 100.6228298 MHz
NUC1 13C
P1 9.70 usec
PLW1 46.98899841 W

======== CHANNEL f2 ========
SFO2 400.1316005 MHz
NUC2 1H
CPDPRG[2 waltz16
PCPD2 90.00 usec
PLW2 11.99499989 W
PLW12 0.34213999 W
PLW13 0.27713001 W

F2 - Processing parameters
SI 32768
SF 100.6127690 MHz
WDW EM
SSB 0
LB 1.00 Hz
GB 0
PC 1.40

Supplementary Figure 68. ^{13}C NMR spectrum of **2t** (100 MHz, r.t., $CDCl_3$)

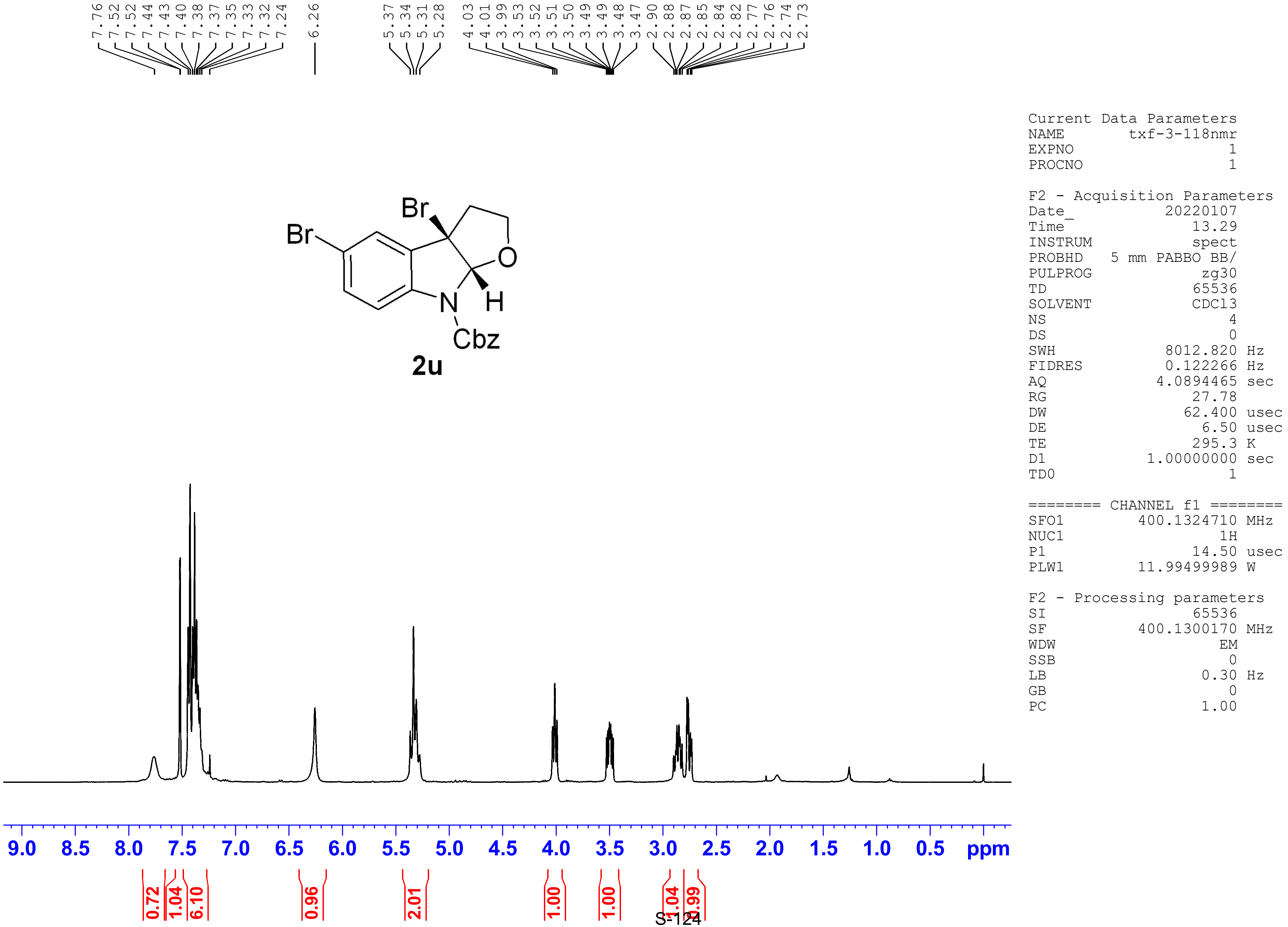

Supplementary Figure 69. ^{1}H NMR spectrum of **2u** (400 MHz, r.t., $CDCl_3$)

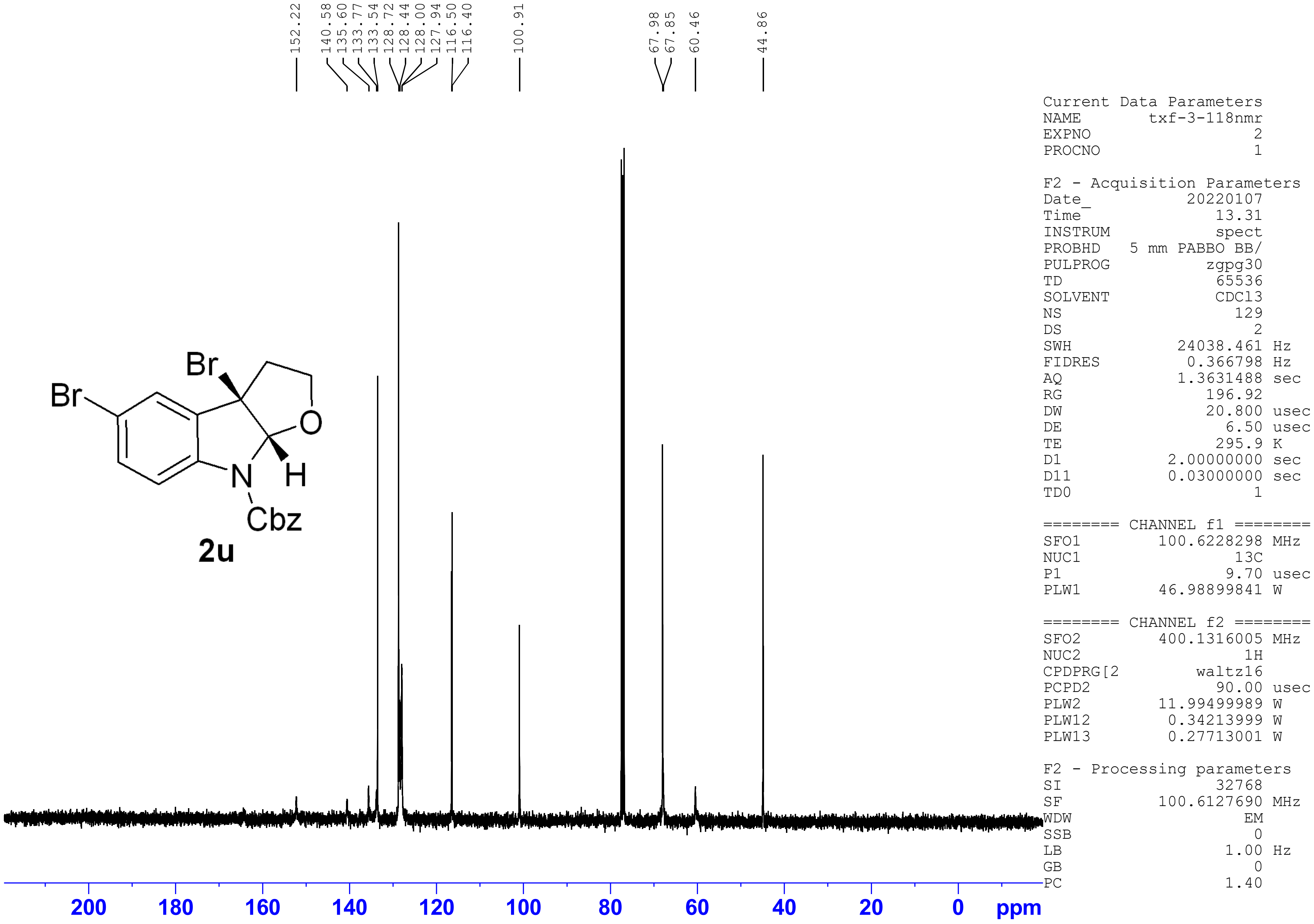

Supplementary Figure 70. ^{13}C NMR spectrum of **2u** (100 MHz, r.t., $CDCl_3$)

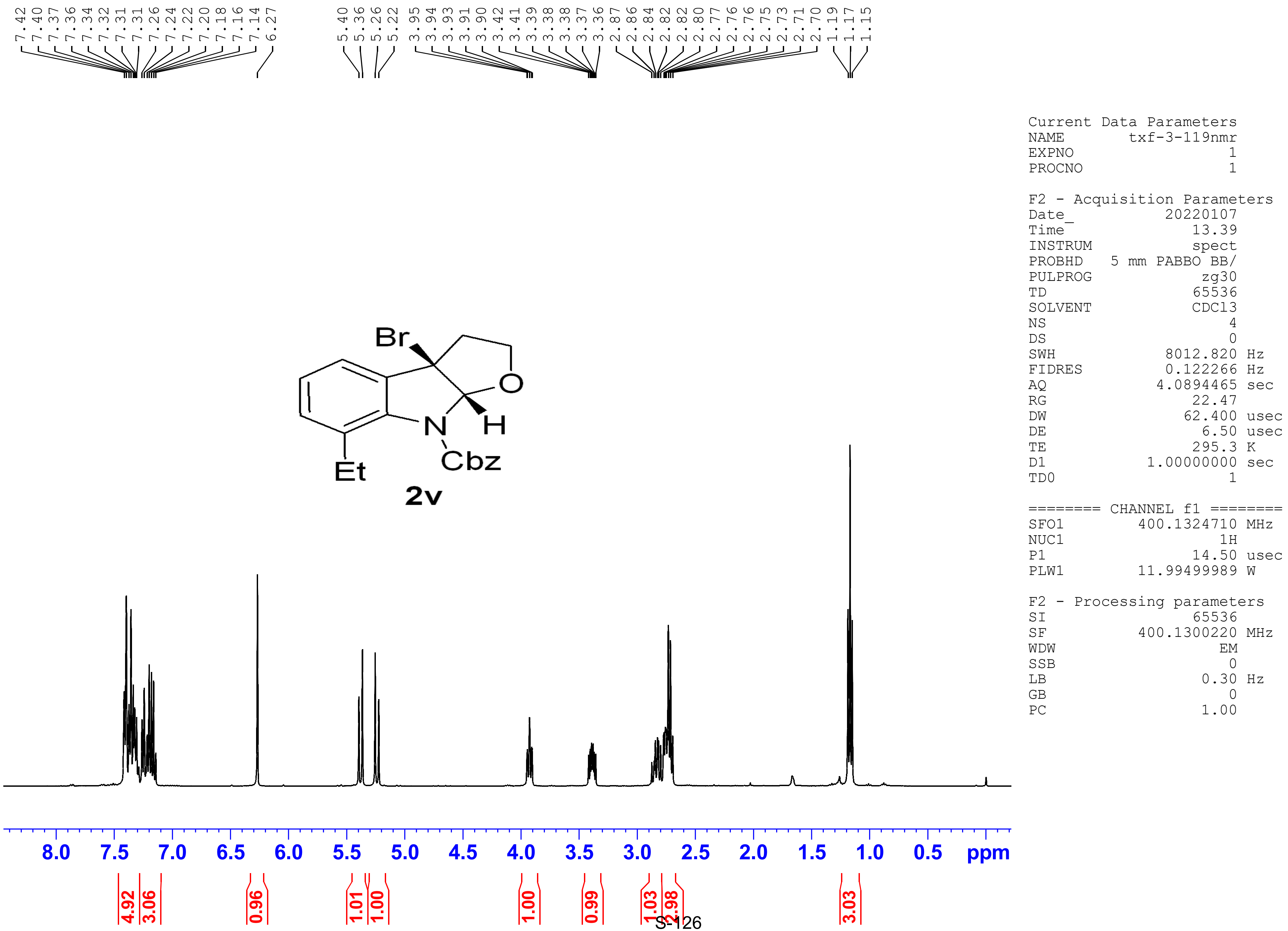

Supplementary Figure 71. ^{1}H NMR spectrum of **2v** (400 MHz, r.t., $CDCl_3$)

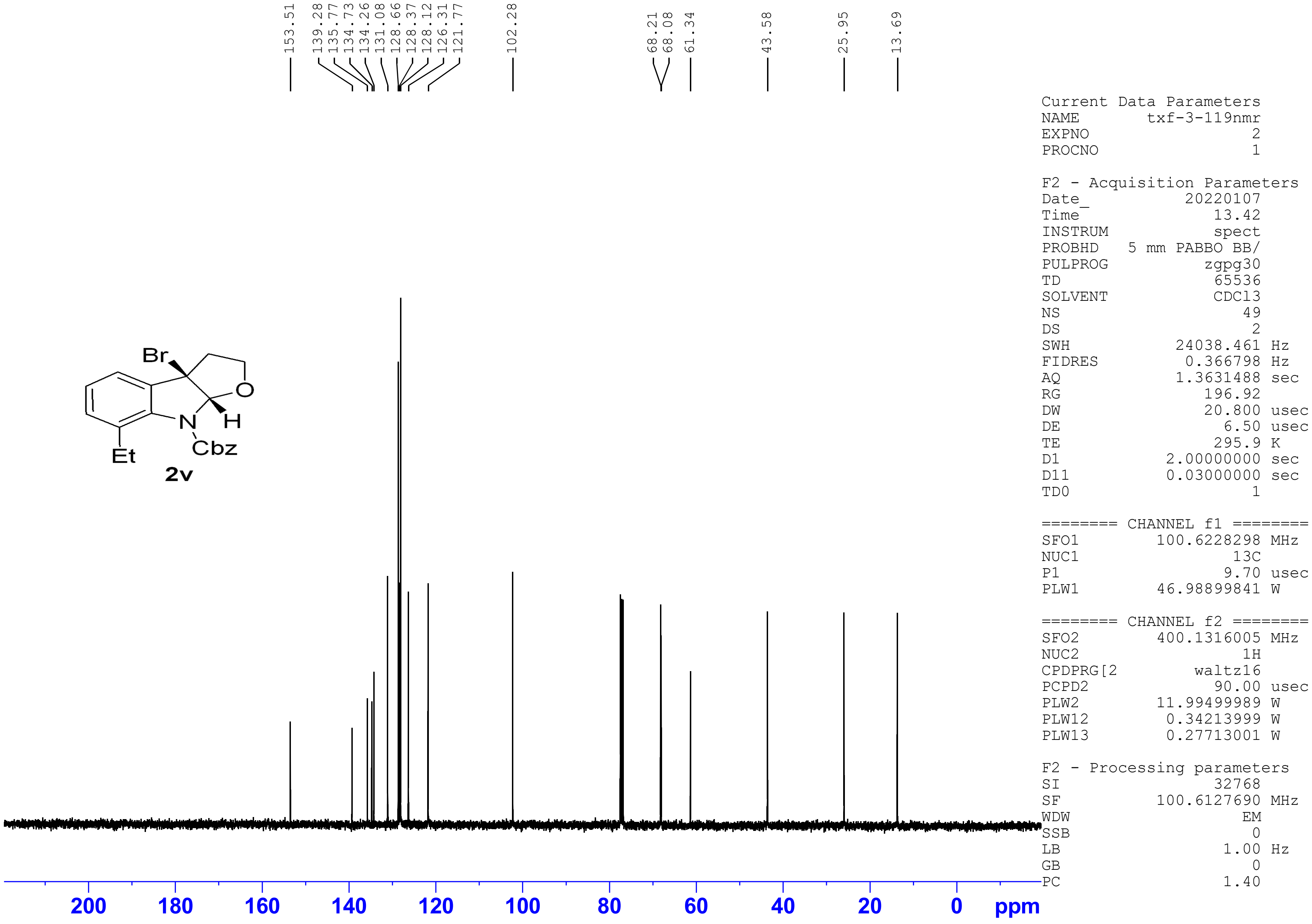

Supplementary Figure 72. ^{13}C NMR spectrum of **2v** (100 MHz, r.t., $CDCl_3$)

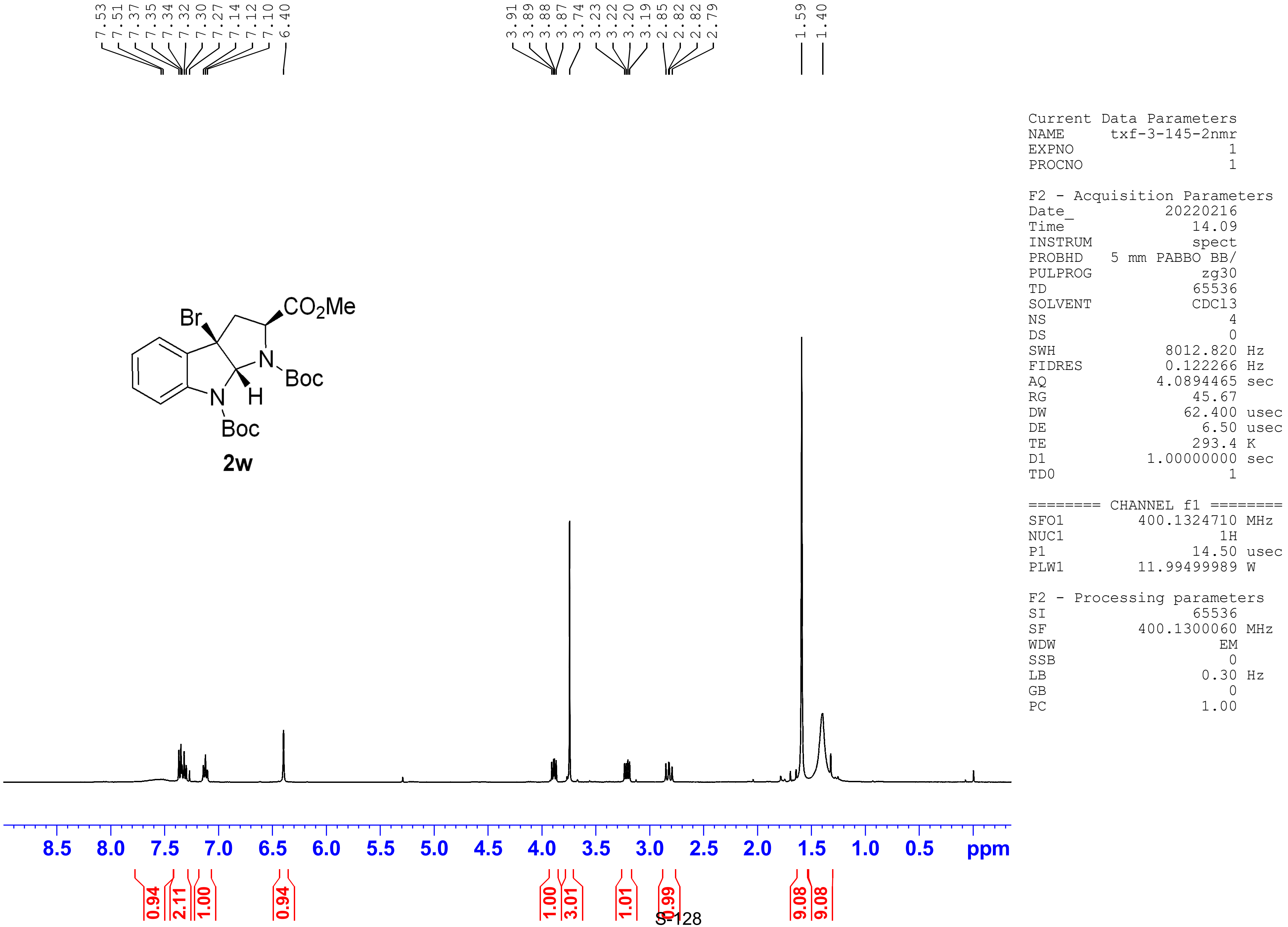

Supplementary Figure 73. ^{1}H NMR spectrum of **2w** (400 MHz, r.t., $CDCl_3$)

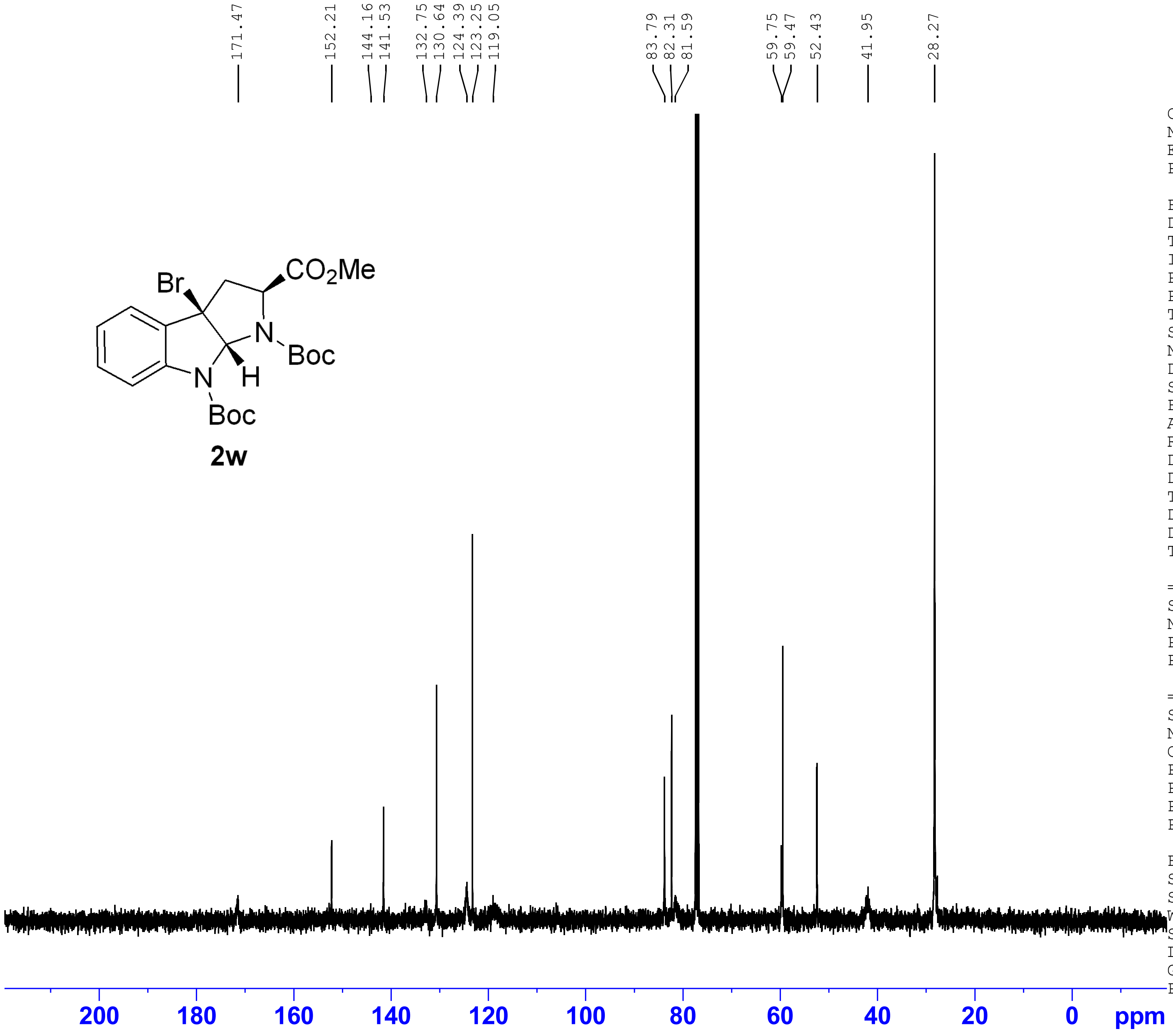


```
Current Data Parameters
NAME      txf-3-145-2nmr
EXPNO                  2
PROCNO                 1

F2 - Acquisition Parameters
Date_           20220216
Time               14.11
INSTRUM            spect
PROBHD    5 mm PABBO BB/
PULPROG           zgpg30
TD                 65536
SOLVENT            CDCl3
NS                   141
DS                     2
SWH            24038.461 Hz
FIDRES          0.366798 Hz
AQ             1.3631488 sec
RG                196.92
DW                20.800 usec
DE                  6.50 usec
TE                 293.8 K
D1            2.00000000 sec
D11           0.03000000 sec
TD0                    1

======== CHANNEL f1 ========
SFO1         100.6228298 MHz
NUC1                 13C
P1                  9.70 usec
PLW1         46.98899841 W

======== CHANNEL f2 ========
SFO2         400.1316005 MHz
NUC2                  1H
CPDPRG[2         waltz16
PCPD2              90.00 usec
PLW2         11.99499989 W
PLW12         0.34213999 W
PLW13         0.27713001 W

F2 - Processing parameters
SI                 32768
SF           100.6127690 MHz
WDW                   EM
SSB                    0
LB                  1.00 Hz
GB                     0
PC                  1.40
```


Supplementary Figure 74. ^{13}C NMR spectrum of **2w** (100 MHz, r.t., $CDCl_3$)

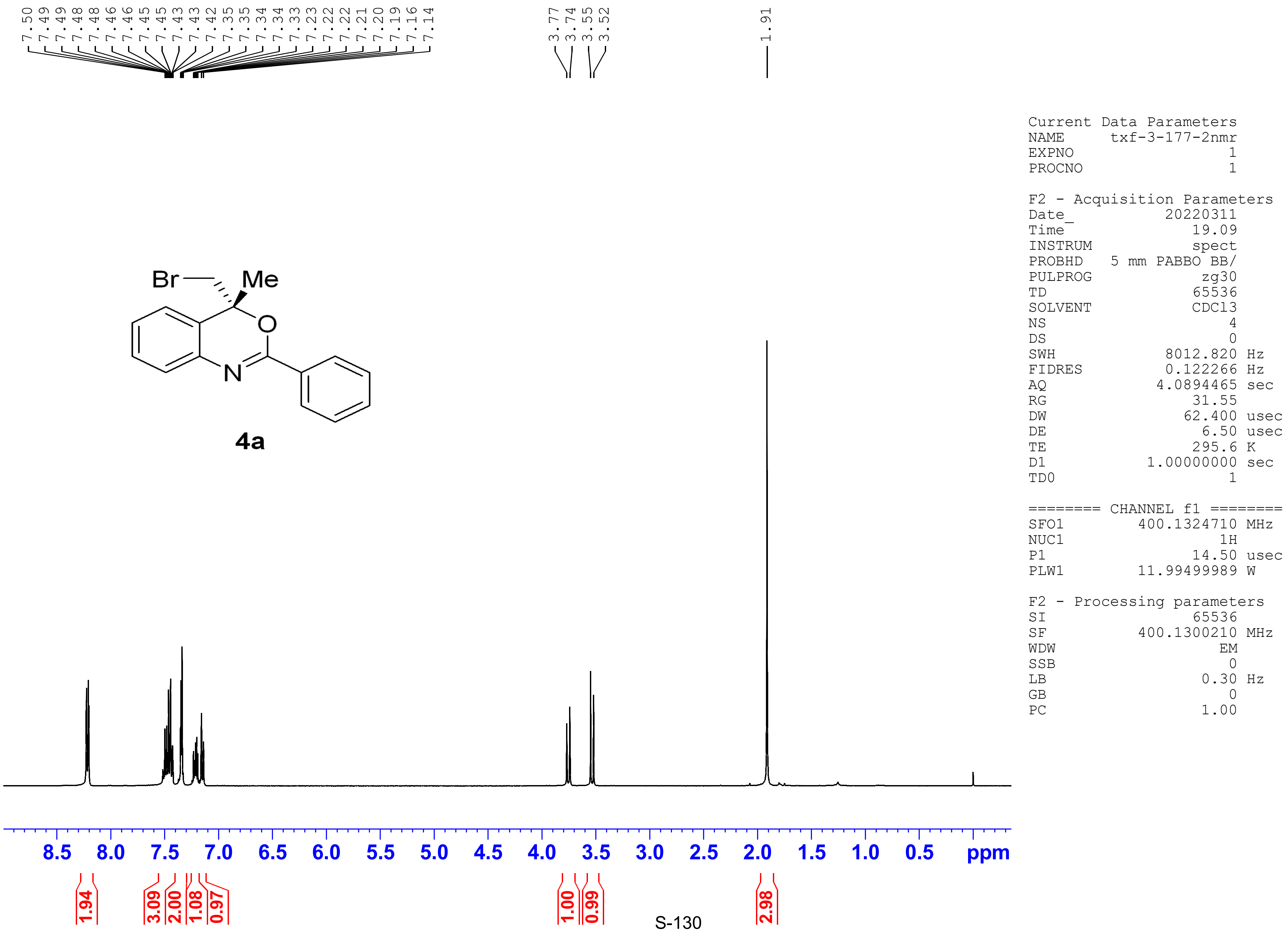

Supplementary Figure 75. ^{1}H NMR spectrum of **4a** (400 MHz, r.t., $CDCl_3$)

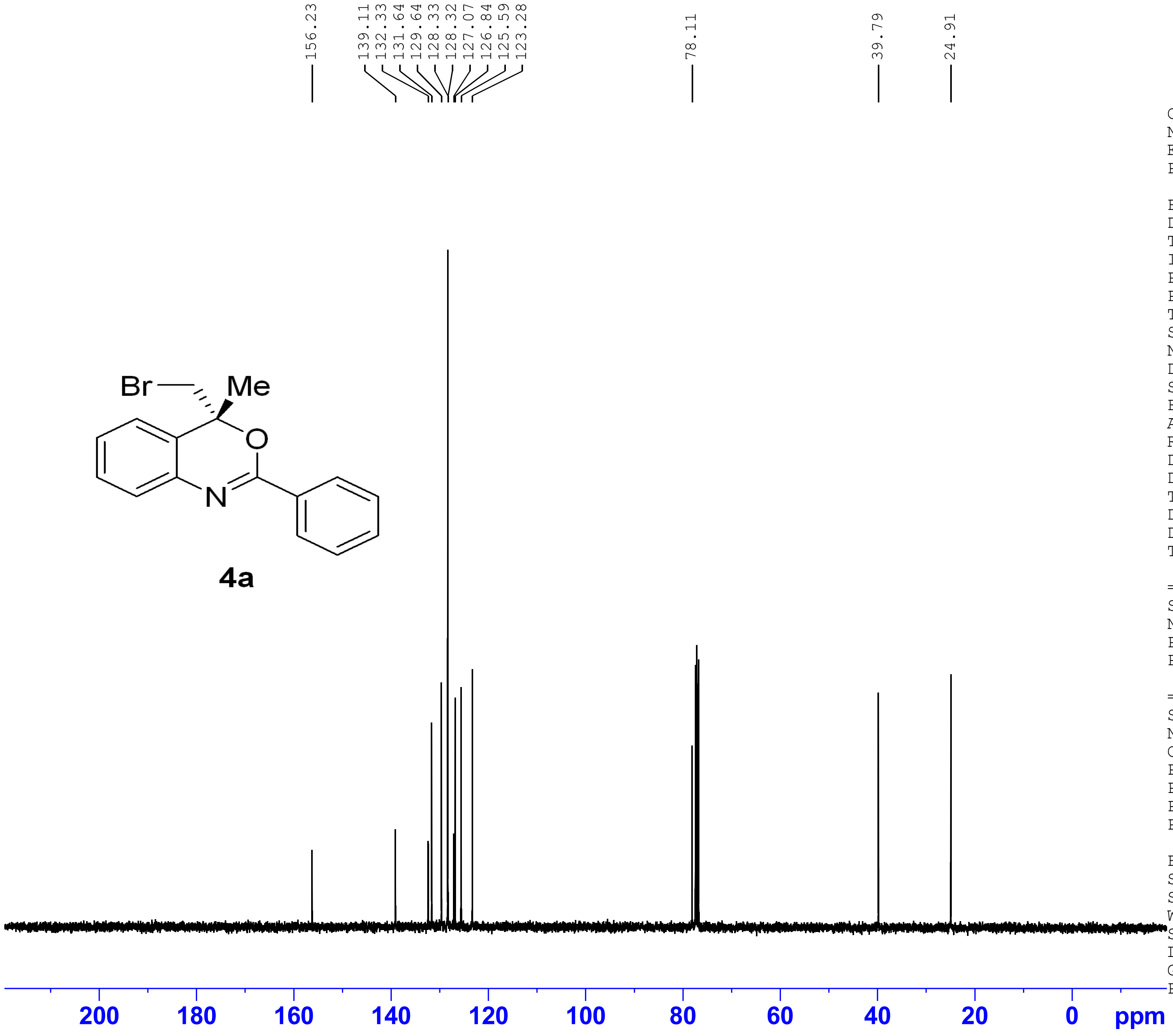

Current Data Parameters
NAME txf-3-177-2nmr
EXPNO 2
PROCNO 1

F2 - Acquisition Parameters
Date_ 20220311
Time 19.11
INSTRUM spect
PROBHD 5 mm PABBO BB/
PULPROG zgpg30
TD 65536
SOLVENT CDCl3
NS 55
DS 2
SWH 24038.461 Hz
FIDRES 0.366798 Hz
AQ 1.3631488 sec
RG 196.92
DW 20.800 usec
DE 6.50 usec
TE 296.2 K
D1 2.00000000 sec
D11 0.03000000 sec
TD0 1

======== CHANNEL f1 ========
SFO1 100.6228298 MHz
NUC1 13C
P1 9.70 usec
PLW1 46.98899841 W

======== CHANNEL f2 ========
SFO2 400.1316005 MHz
NUC2 1H
CPDPRG[2 waltz16
PCPD2 90.00 usec
PLW2 11.99499989 W
PLW12 0.34213999 W
PLW13 0.27713001 W

F2 - Processing parameters
SI 32768
SF 100.6127690 MHz
WDW EM
SSB 0
LB 1.00 Hz
GB 0
PC 1.40

Supplementary Figure 76. ^{13}C NMR spectrum of **4a** (100 MHz, r.t., $CDCl_3$)

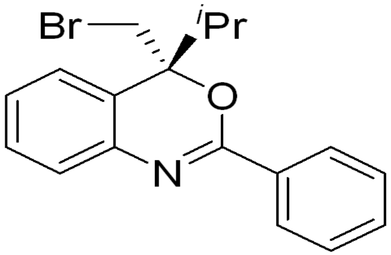

4b

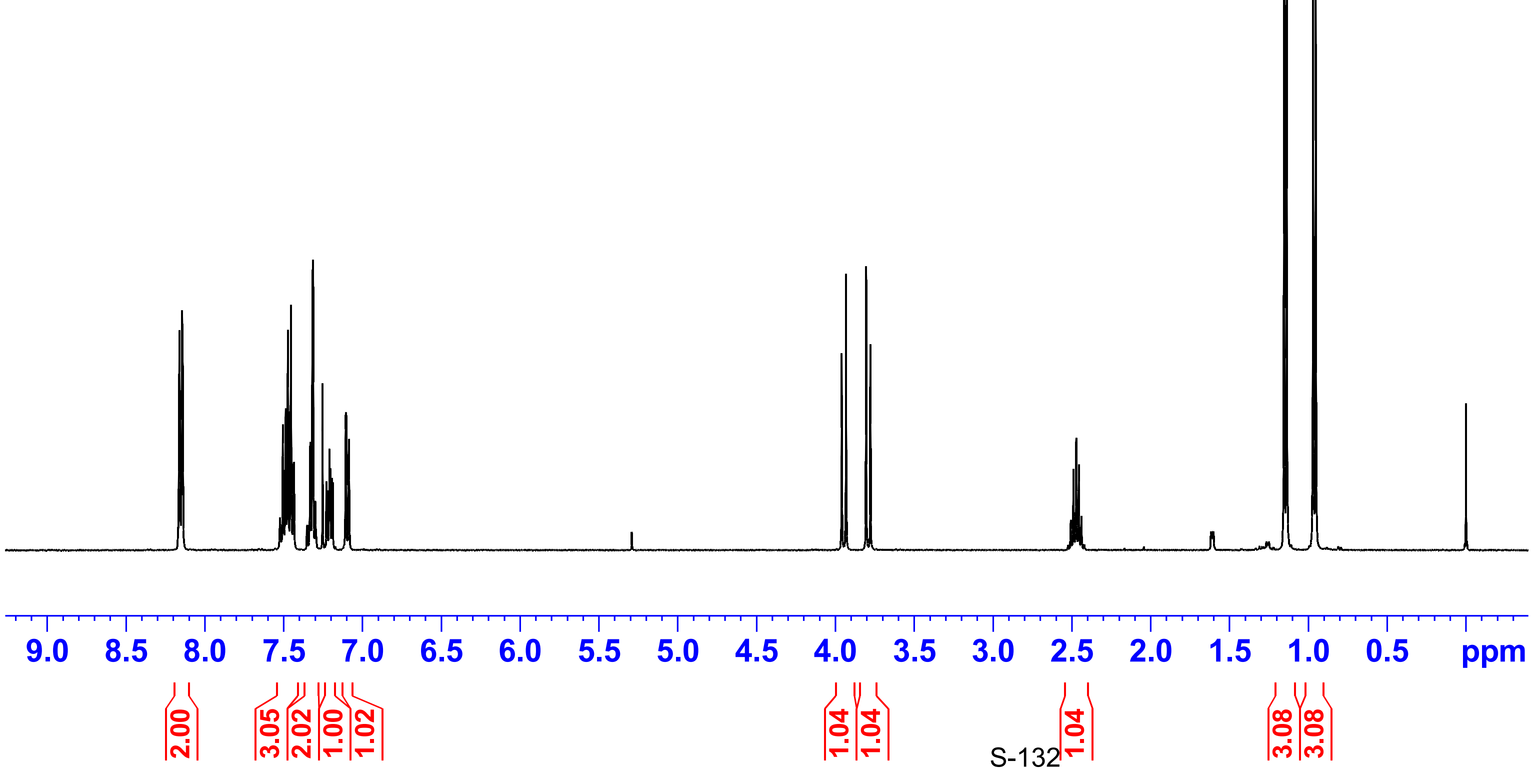

Current Data Parameters
NAME txf-3-176-8nmr
EXPNO 3
PROCNO 1

F2 - Acquisition Parameters
Date_ 20220312
Time 11.01
INSTRUM spect
PROBHD 5 mm PABBO BB/
PULPROG zg30
TD 65536
SOLVENT CDCl3
NS 4
DS 0
SWH 8012.820 Hz
FIDRES 0.122266 Hz
AQ 4.0894465 sec
RG 196.92
DW 62.400 usec
DE 6.50 usec
TE 295.6 K
D1 1.00000000 sec
TD0 1

======== CHANNEL f1 ========
SFO1 400.1324710 MHz
NUC1 1H
P1 14.50 usec
PLW1 11.99499989 W

F2 - Processing parameters
SI 65536
SF 400.1300123 MHz
WDW EM
SSB 0
LB 0.30 Hz
GB 0
PC 1.00

Supplementary Figure 77. ^{1}H NMR spectrum of **4b** (400 MHz, r.t., $CDCl_3$)

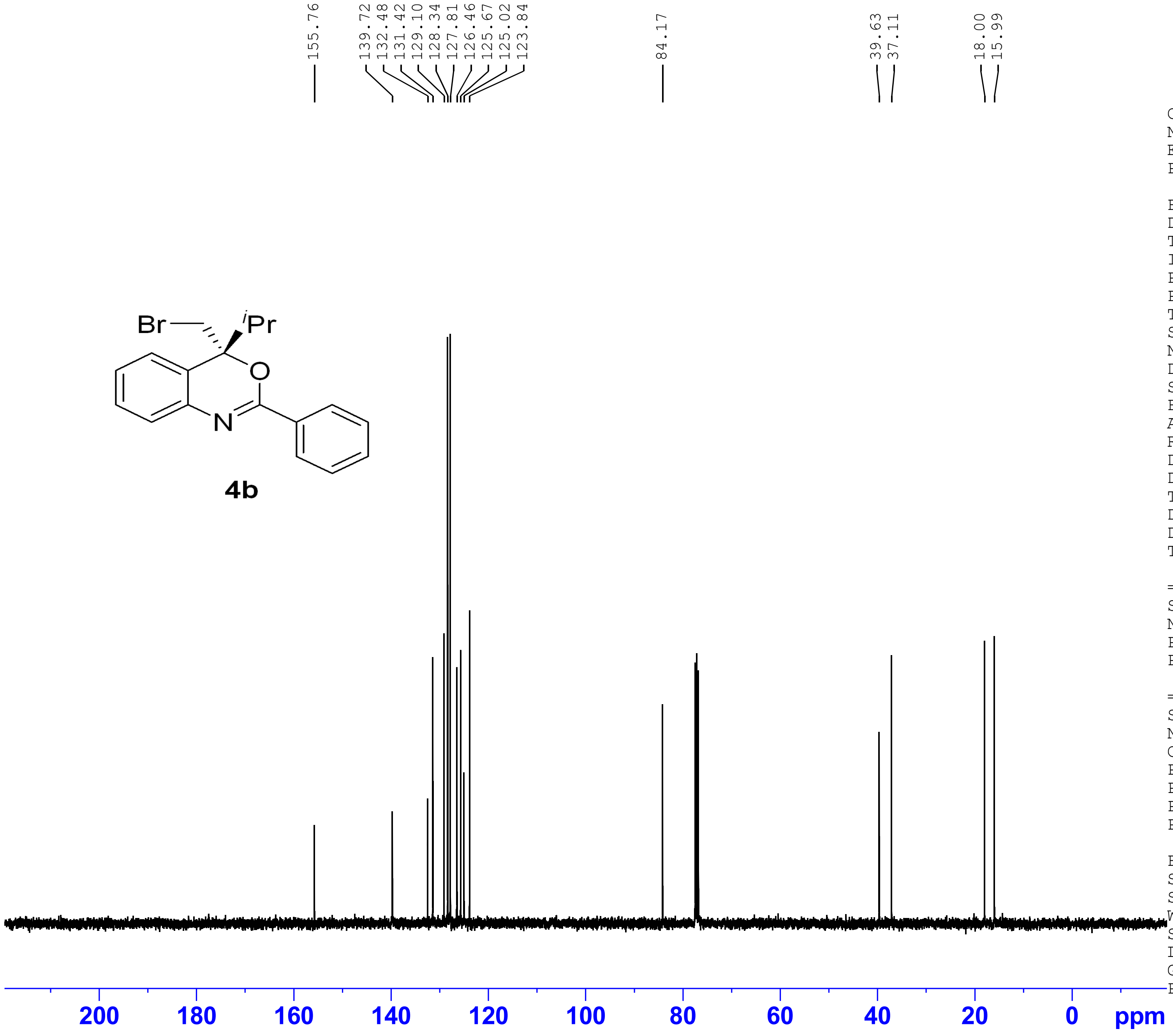

Current Data Parameters
NAME txf-3-176-8nmr
EXPNO 2
PROCNO 1

F2 - Acquisition Parameters
Date_ 20220311
Time 19.05
INSTRUM spect
PROBHD 5 mm PABBO BB/
PULPROG zgpg30
TD 65536
SOLVENT CDCl3
NS 48
DS 2
SWH 24038.461 Hz
FIDRES 0.366798 Hz
AQ 1.3631488 sec
RG 196.92
DW 20.800 usec
DE 6.50 usec
TE 295.9 K
D1 2.00000000 sec
D11 0.03000000 sec
TD0 1

======== CHANNEL f1 ========
SFO1 100.6228298 MHz
NUC1 13C
P1 9.70 usec
PLW1 46.98899841 W

======== CHANNEL f2 ========
SFO2 400.1316005 MHz
NUC2 1H
CPDPRG[2 waltz16
PCPD2 90.00 usec
PLW2 11.99499989 W
PLW12 0.34213999 W
PLW13 0.27713001 W

F2 - Processing parameters
SI 32768
SF 100.6127690 MHz
WDW EM
SSB 0
LB 1.00 Hz
GB 0
PC 1.40

Supplementary Figure 78. ^{13}C NMR spectrum of **4b** (100 MHz, r.t., $CDCl_3$)

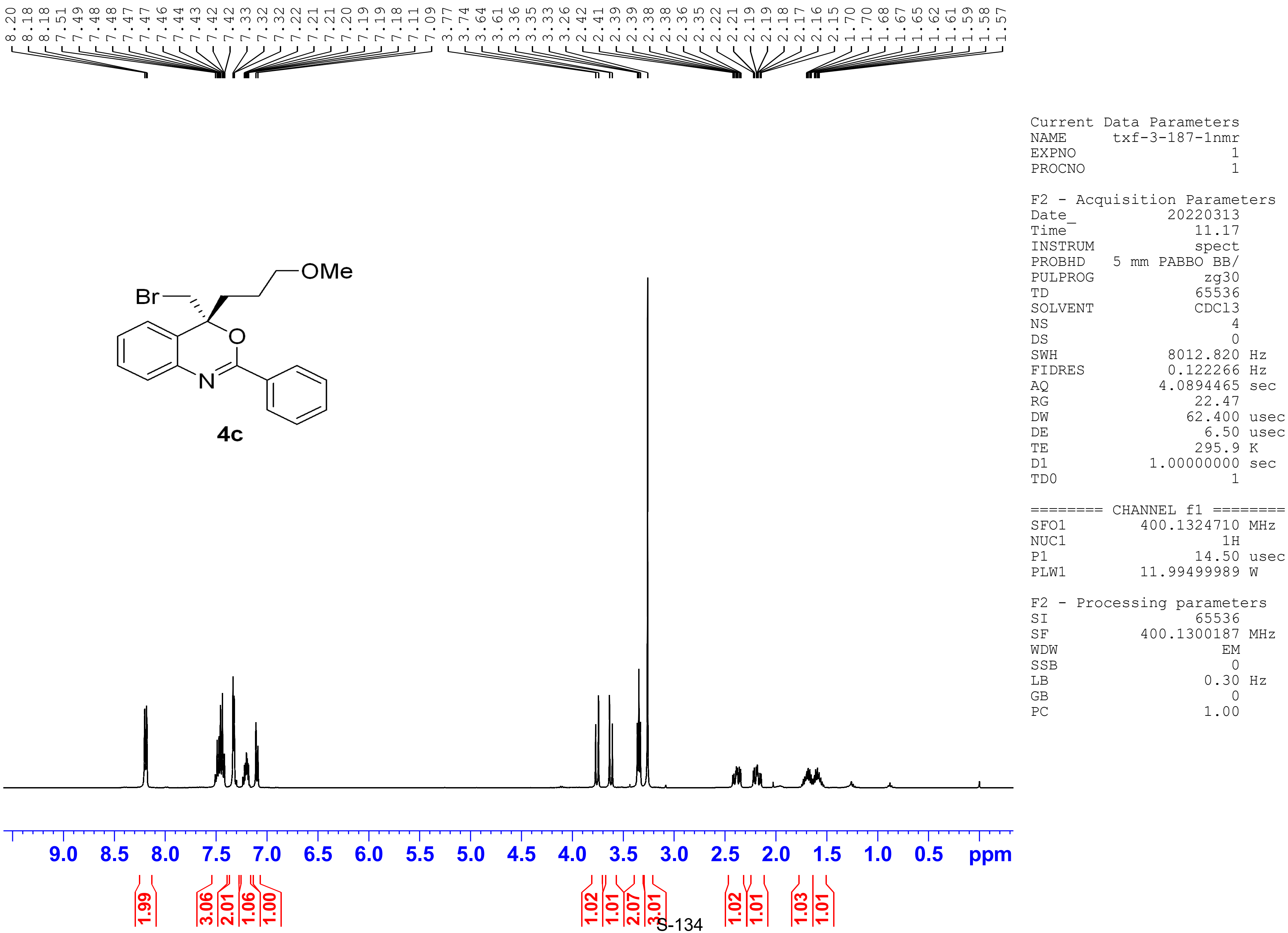

Supplementary Figure 79. ^{1}H NMR spectrum of **4c** (400 MHz, r.t., $CDCl_3$)

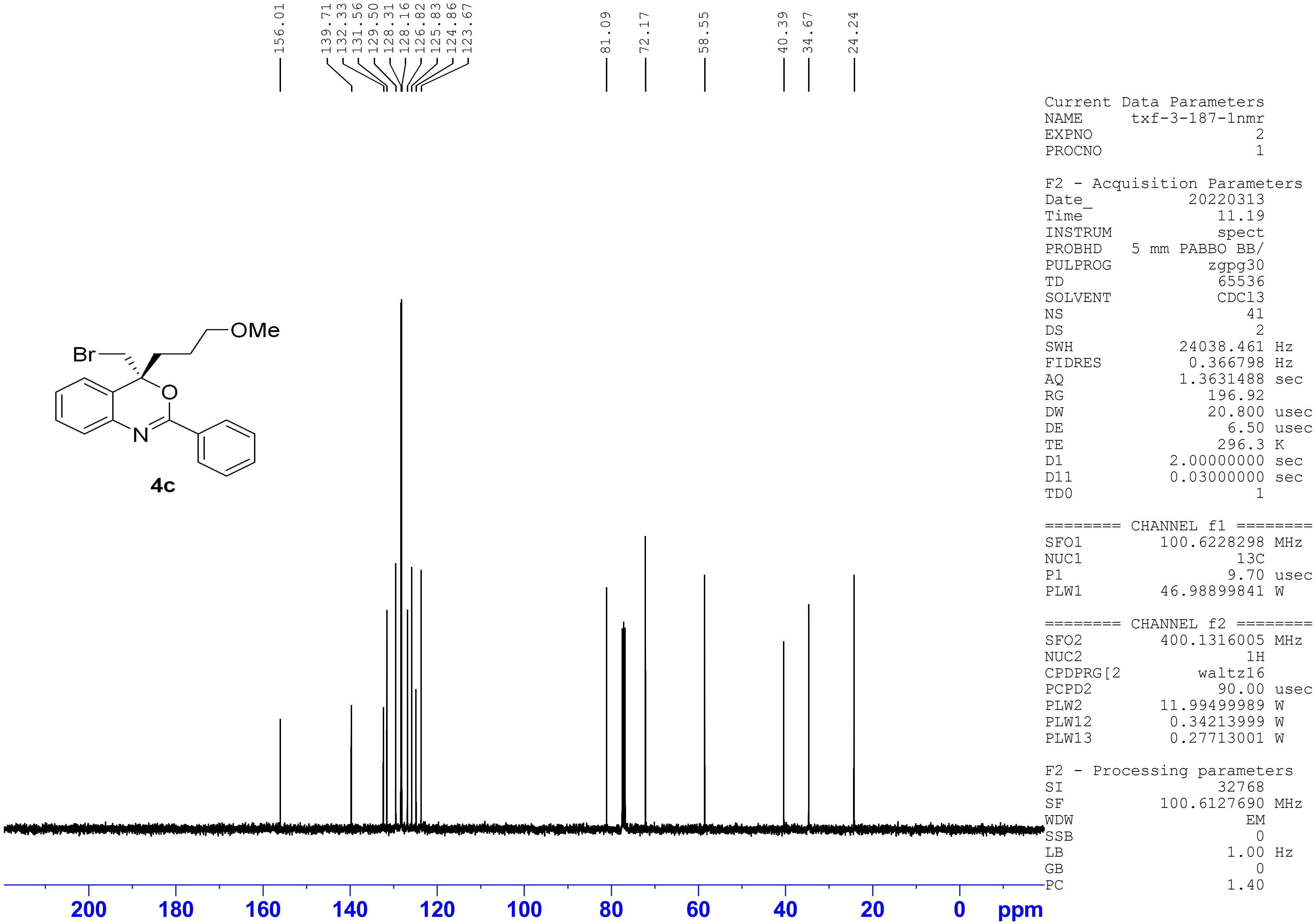

Supplementary Figure 80. ^{13}C NMR spectrum of **4c** (100 MHz, r.t., $CDCl_3$)

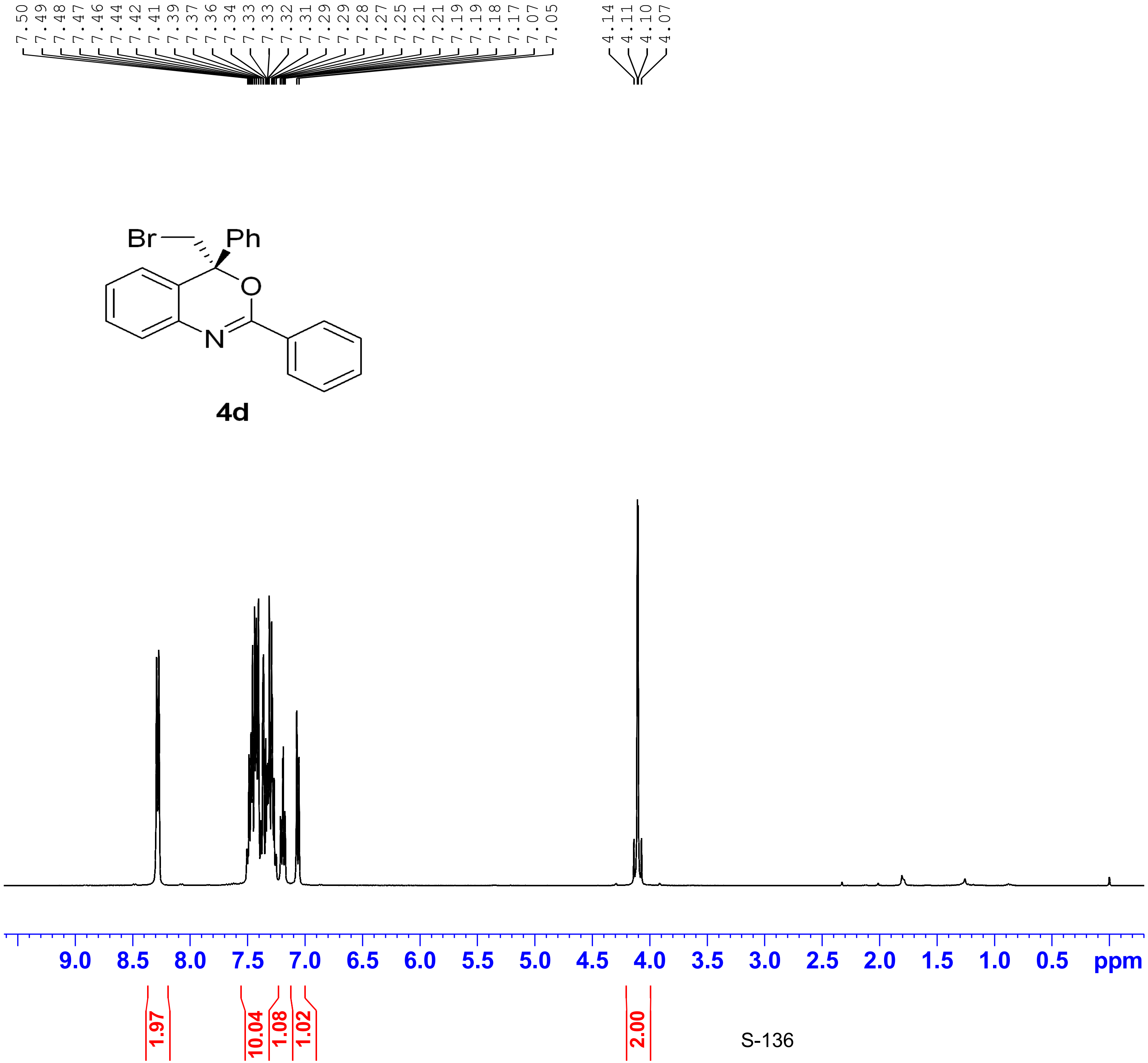


```
Current Data Parameters
NAME          txf-4-10nmr
EXPNO                   1
PROCNO                  1

F2 - Acquisition Parameters
Date_            20220318
Time                22.00
INSTRUM             spect
PROBHD   5 mm PABBO BB/
PULPROG              zg30
TD                  65536
SOLVENT             CDCl3
NS                      4
DS                      0
SWH              8012.820 Hz
FIDRES           0.122266 Hz
AQ              4.0894465 sec
RG                  34.77
DW                 62.400 usec
DE                   6.50 usec
TE                  296.1 K
D1             1.00000000 sec
TD0                     1

======== CHANNEL f1 ========
SFO1          400.1324710 MHz
NUC1                   1H
P1                  14.50 usec
PLW1          11.99499989 W

F2 - Processing parameters
SI                  65536
SF            400.1300378 MHz
WDW                    EM
SSB                     0
LB                   0.30 Hz
GB                      0
PC                   1.00
```


Supplementary Figure 81. ^{1}H NMR spectrum of **4d** (400 MHz, r.t., $CDCl_3$)

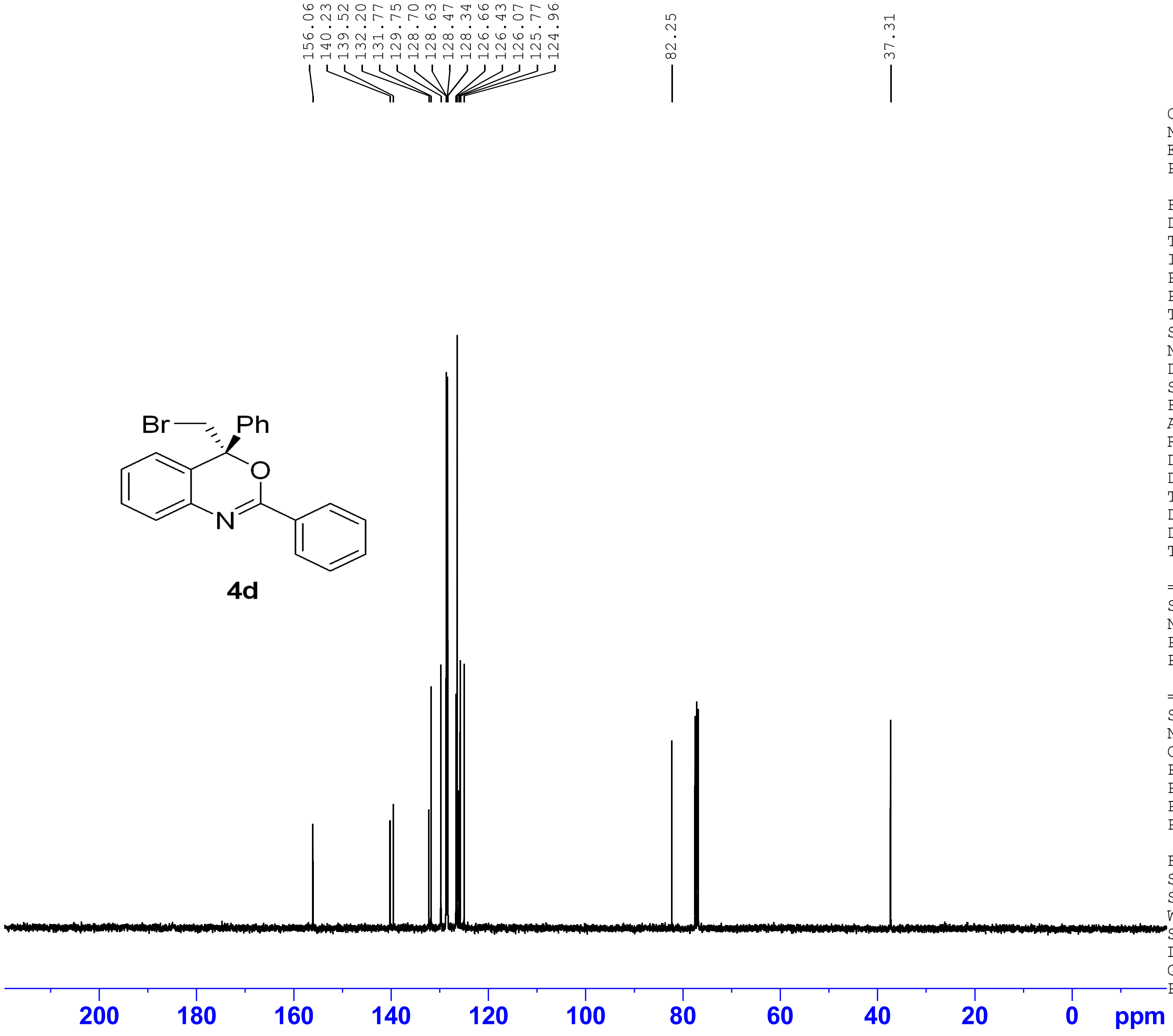


```
Current Data Parameters
NAME          txf-4-10nmr
EXPNO                   2
PROCNO                  1

F2 - Acquisition Parameters
Date_            20220318
Time                22.02
INSTRUM             spect
PROBHD    5 mm PABBO BB/
PULPROG            zgpg30
TD                  65536
SOLVENT             CDCl3
NS                     54
DS                      2
SWH             24038.461 Hz
FIDRES           0.366798 Hz
AQ              1.3631488 sec
RG                 196.92
DW                 20.800 usec
DE                   6.50 usec
TE                  296.5 K
D1             2.00000000 sec
D11            0.03000000 sec
TD0                     1

======== CHANNEL f1 ========
SFO1         100.6228298 MHz
NUC1                 13C
P1                   9.70 usec
PLW1          46.98899841 W

======== CHANNEL f2 ========
SFO2         400.1316005 MHz
NUC2                  1H
CPDPRG[2          waltz16
PCPD2               90.00 usec
PLW2          11.99499989 W
PLW12          0.34213999 W
PLW13          0.27713001 W

F2 - Processing parameters
SI                  32768
SF            100.6127690 MHz
WDW                    EM
SSB                     0
LB                   1.00 Hz
GB                      0
PC                   1.40
```


Supplementary Figure 82. ^{13}C NMR spectrum of **4d** (100 MHz, r.t., $CDCl_3$)

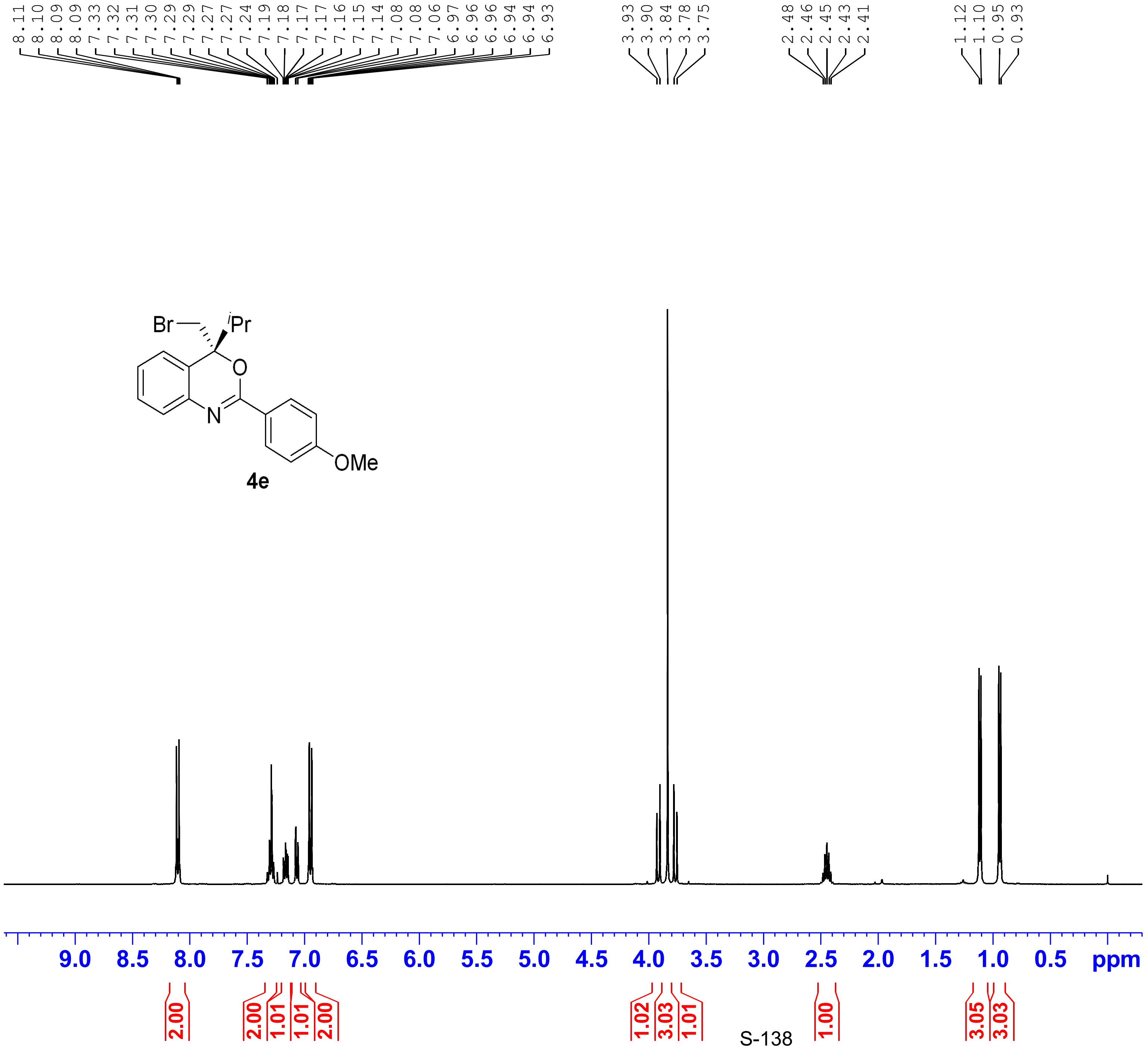


```
Current Data Parameters
NAME      txf-3-184-1nmr
EXPNO                  1
PROCNO                 1

F2 - Acquisition Parameters
Date_           20220312
Time               11.03
INSTRUM            spect
PROBHD   5 mm PABBO BB/
PULPROG             zg30
TD                 65536
SOLVENT            CDCl3
NS                     4
DS                     0
SWH             8012.820 Hz
FIDRES          0.122266 Hz
AQ             4.0894465 sec
RG                 62.93
DW                62.400 usec
DE                  6.50 usec
TE                 295.6 K
D1            1.00000000 sec
TD0                    1

======== CHANNEL f1 ========
SFO1         400.1324710 MHz
NUC1                  1H
P1                 14.50 usec
PLW1         11.99499989 W

F2 - Processing parameters
SI                 65536
SF           400.1300192 MHz
WDW                   EM
SSB                    0
LB                  0.30 Hz
GB                     0
PC                  1.00
```


Supplementary Figure 83. ^{1}H NMR spectrum of **4e** (400 MHz, r.t., $CDCl_3$)

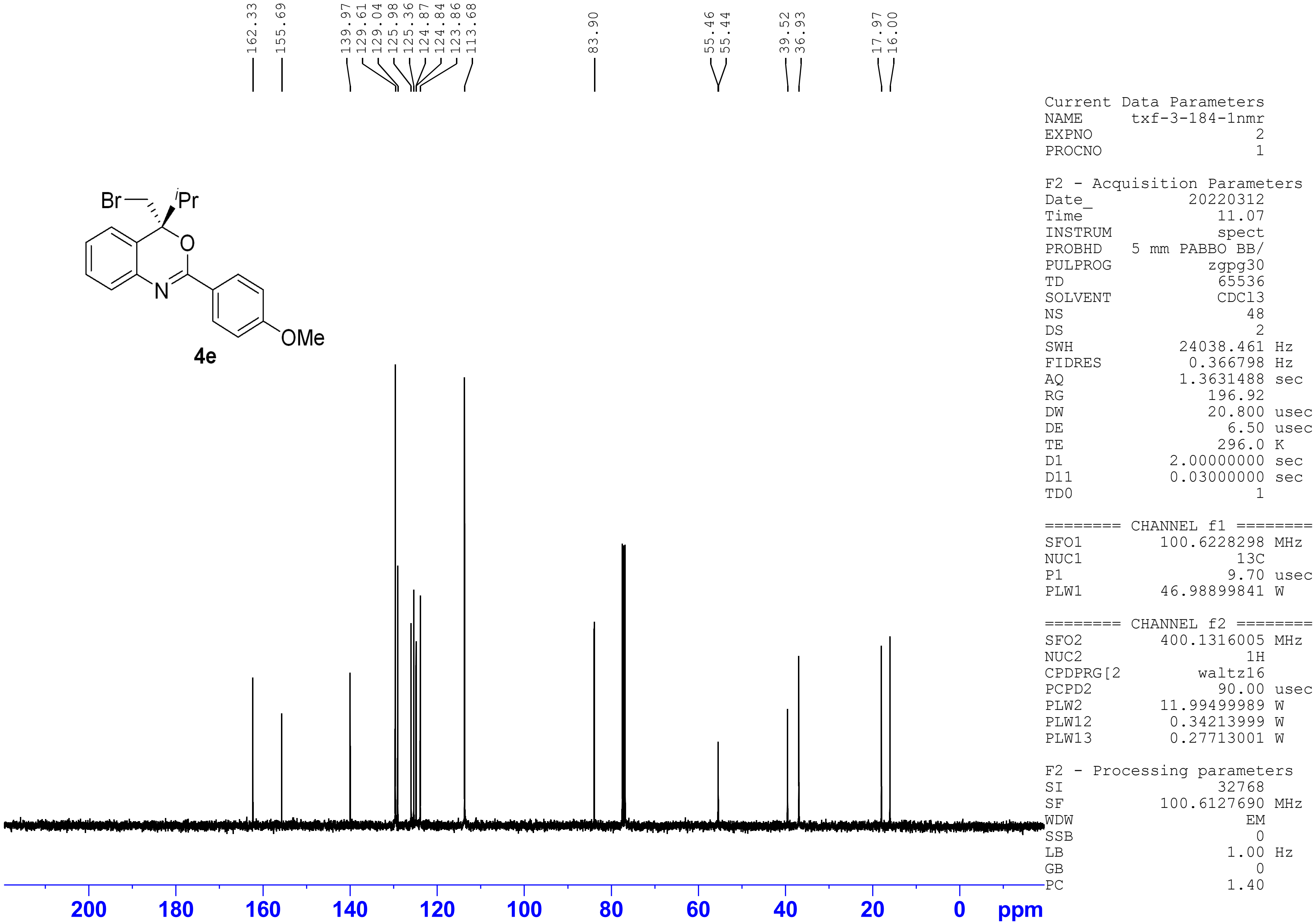

Supplementary Figure 84. ^{13}C NMR spectrum of **4e** (100 MHz, r.t., $CDCl_3$)

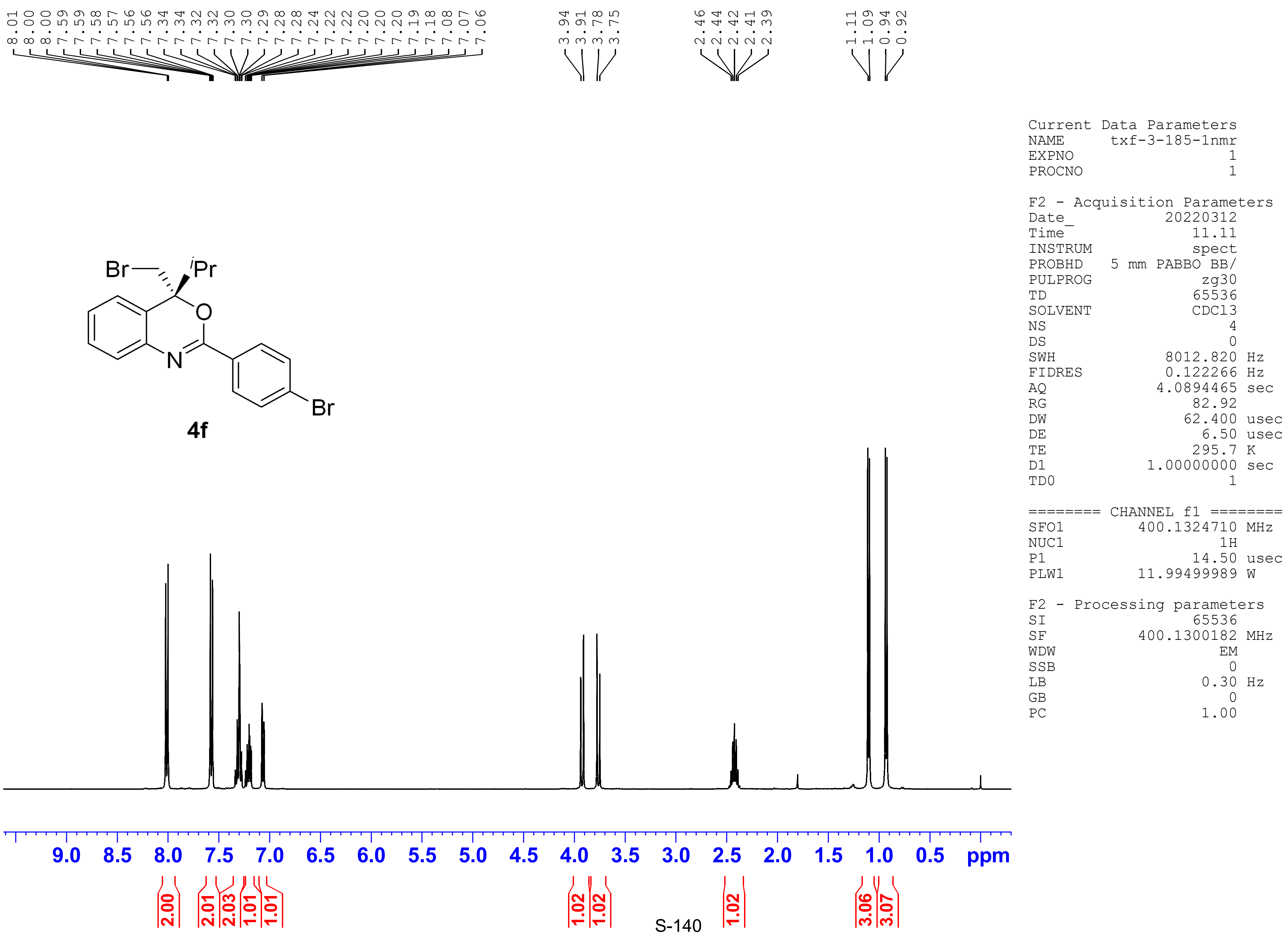

Supplementary Figure 85. ^{1}H NMR spectrum of **4f** (400 MHz, r.t., $CDCl_3$)

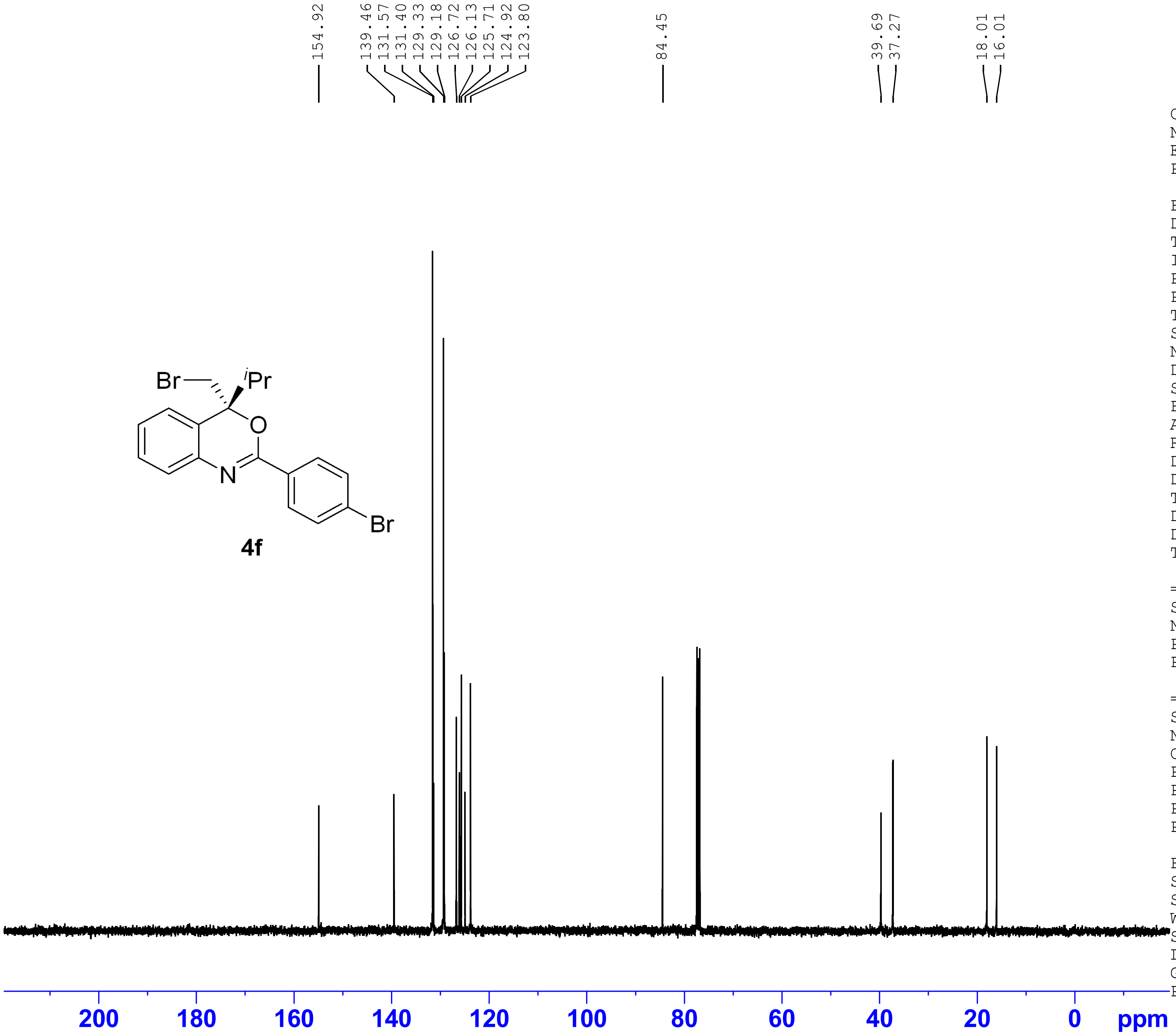

4f

```
Current Data Parameters
NAME       txf-3-185-1nmr
EXPNO                   2
PROCNO                  1

F2 - Acquisition Parameters
Date_            20220312
Time                11.12
INSTRUM             spect
PROBHD   5 mm PABBO BB/
PULPROG           zgpg30
TD                 65536
SOLVENT            CDCl3
NS                    56
DS                     2
SWH            24038.461 Hz
FIDRES          0.366798 Hz
AQ             1.3631488 sec
RG                196.92
DW                20.800 usec
DE                  6.50 usec
TE                 296.0 K
D1            2.00000000 sec
D11           0.03000000 sec
TD0                    1

======== CHANNEL f1 ========
SFO1         100.6228298 MHz
NUC1                 13C
P1                  9.70 usec
PLW1         46.98899841 W

======== CHANNEL f2 ========
SFO2         400.1316005 MHz
NUC2                  1H
CPDPRG[2         waltz16
PCPD2              90.00 usec
PLW2         11.99499989 W
PLW12         0.34213999 W
PLW13         0.27713001 W

F2 - Processing parameters
SI                 32768
SF           100.6127690 MHz
WDW                   EM
SSB                    0
LB                  1.00 Hz
GB                     0
PC                  1.40
```


Supplementary Figure 86. ^{13}C NMR spectrum of **4f** (100 MHz, r.t., $CDCl_3$)

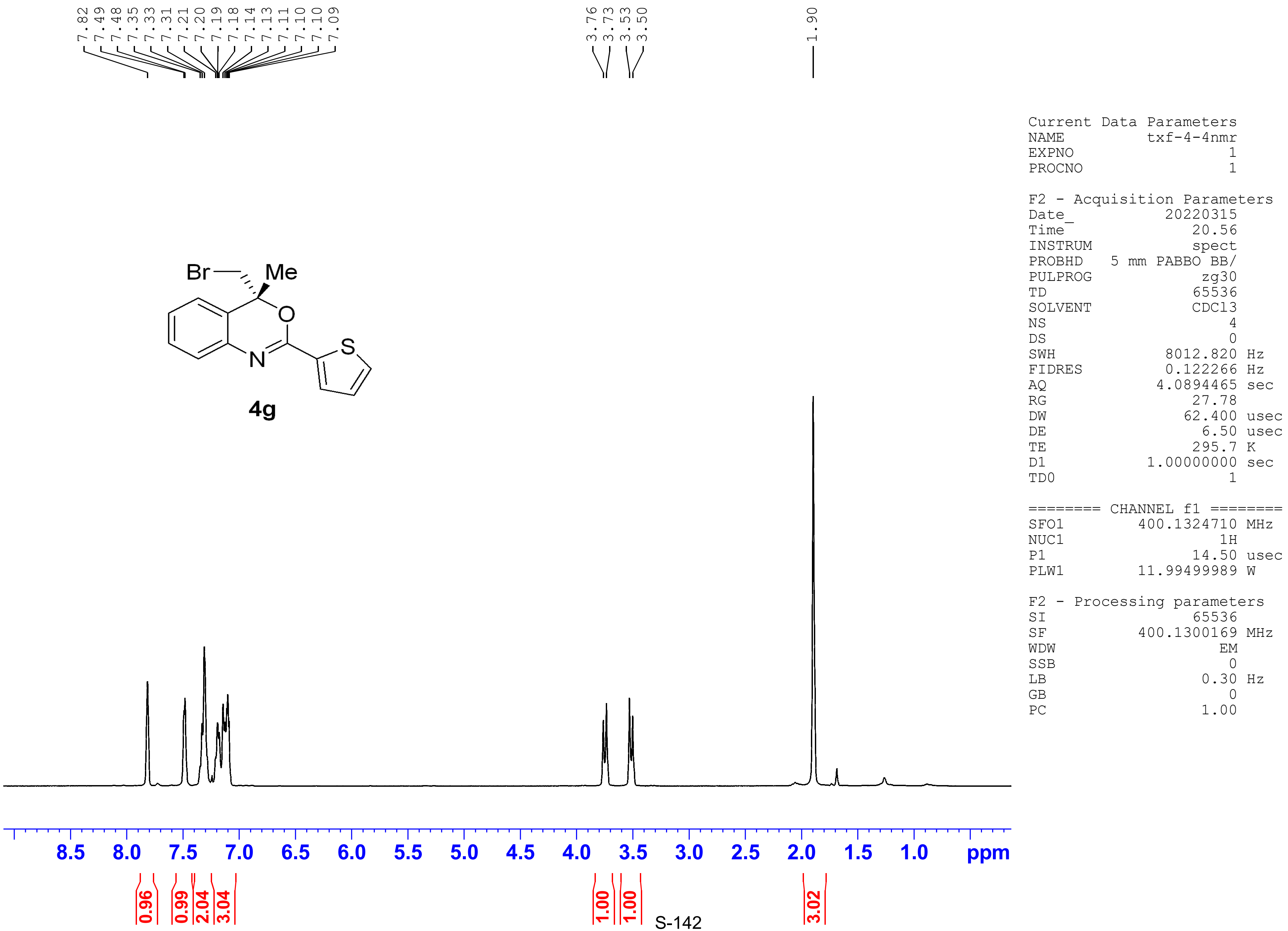

Supplementary Figure 87. ^{1}H NMR spectrum of **4g** (400 MHz, r.t., $CDCl_3$)

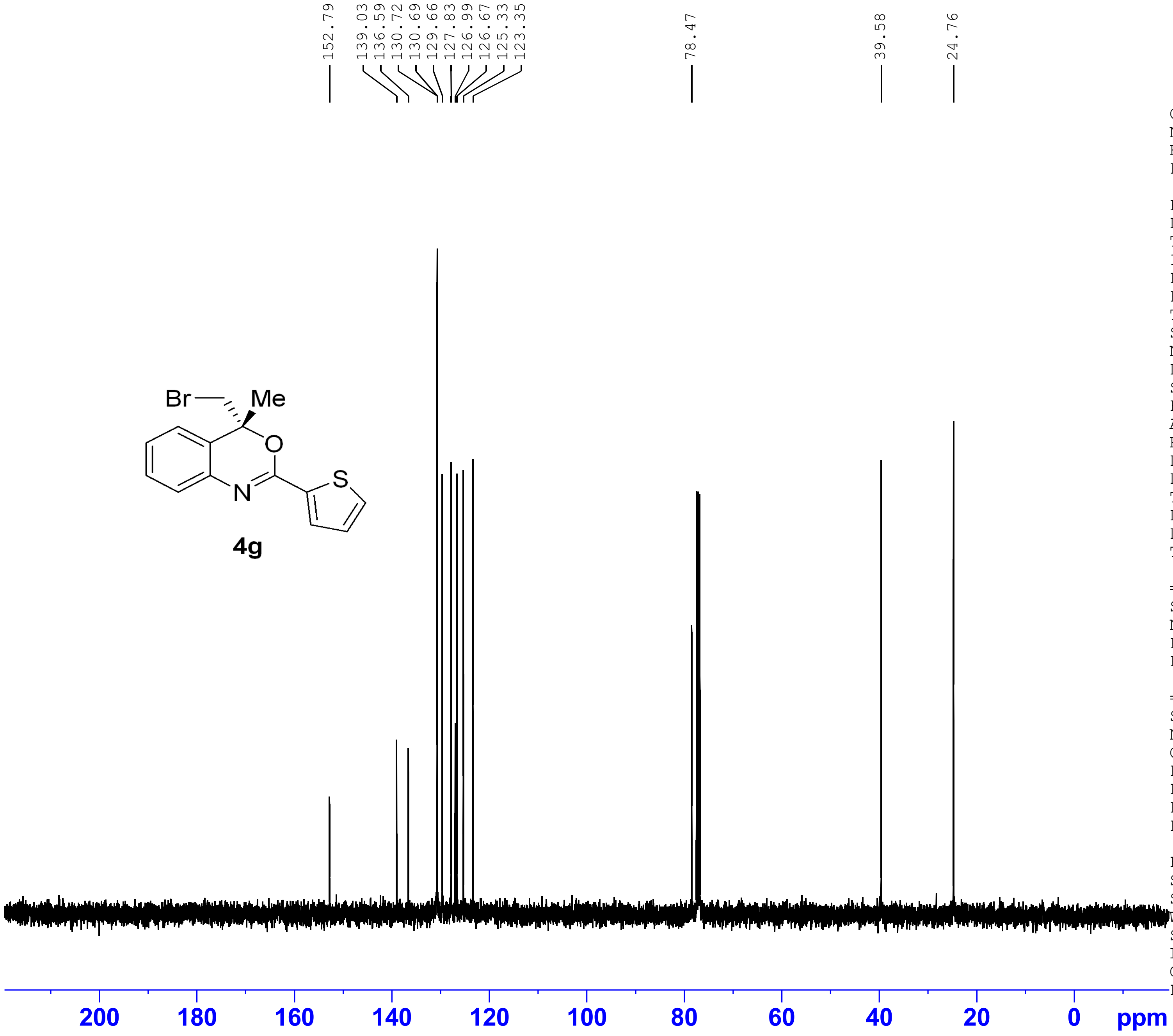


```
Current Data Parameters
NAME          txf-4-4nmr
EXPNO                  3
PROCNO                 1

F2 - Acquisition Parameters
Date_           20220315
Time               21.05
INSTRUM            spect
PROBHD   5 mm PABBO BB/
PULPROG          zgpg30
TD                 65536
SOLVENT            CDCl3
NS                    86
DS                     2
SWH            24038.461 Hz
FIDRES          0.366798 Hz
AQ             1.3631488 sec
RG                196.92
DW                20.800 usec
DE                  6.50 usec
TE                 296.6 K
D1            2.00000000 sec
D11           0.03000000 sec
TD0                    1

======== CHANNEL f1 ========
SFO1         100.6228298 MHz
NUC1                 13C
P1                  9.70 usec
PLW1         46.98899841 W

======== CHANNEL f2 ========
SFO2         400.1316005 MHz
NUC2                  1H
CPDPRG[2         waltz16
PCPD2              90.00 usec
PLW2         11.99499989 W
PLW12         0.34213999 W
PLW13         0.27713001 W

F2 - Processing parameters
SI                 32768
SF           100.6127690 MHz
WDW                   EM
SSB                    0
LB                  1.00 Hz
GB                     0
PC                  1.40
```


Supplementary Figure 88. ^{13}C NMR spectrum of **4g** (100 MHz, r.t., $CDCl_3$)

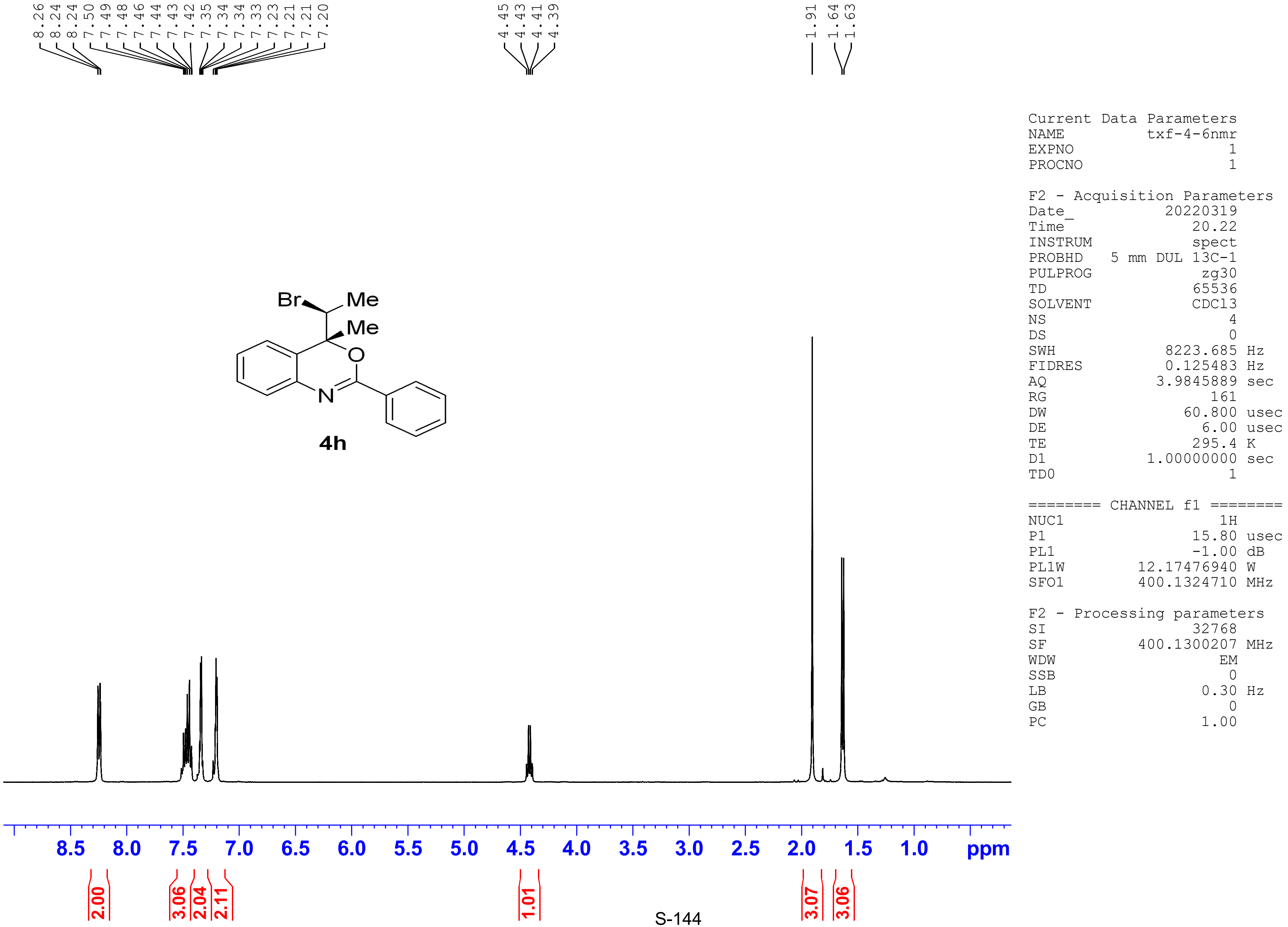

Supplementary Figure 89. ^{1}H NMR spectrum of **4h** (400 MHz, r.t., $CDCl_3$)

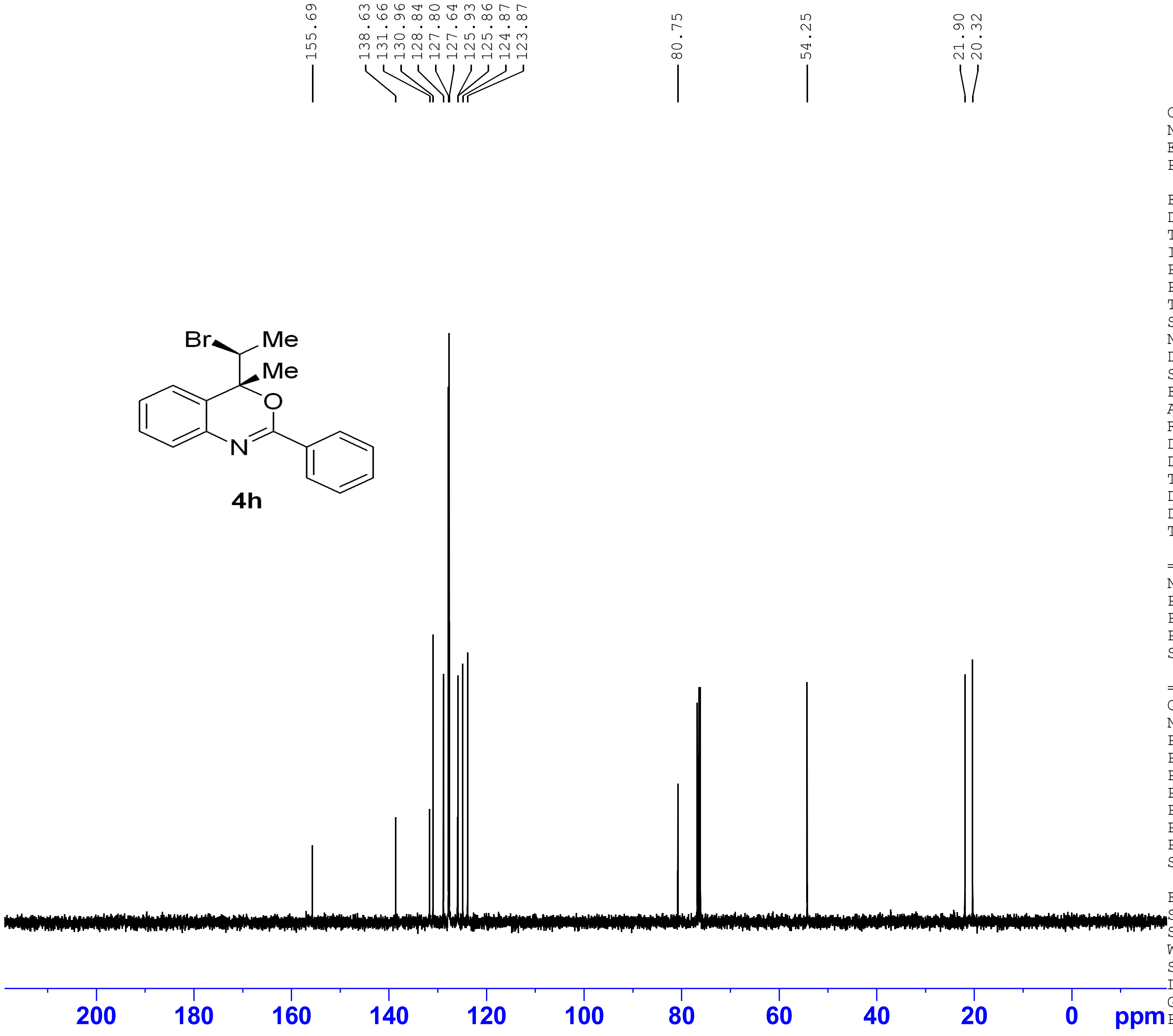


```
Current Data Parameters
NAME          txf-4-6nmr
EXPNO                  2
PROCNO                 1

F2 - Acquisition Parameters
Date_           20220319
Time               20.25
INSTRUM            spect
PROBHD    5 mm DUL 13C-1
PULPROG          zgpg30
TD                 65536
SOLVENT            CDCl3
NS                   106
DS                     1
SWH            24038.461 Hz
FIDRES          0.366798 Hz
AQ             1.3631488 sec
RG                  2050
DW                20.800 usec
DE                  6.00 usec
TE                 295.6 K
D1            2.00000000 sec
D11           0.03000000 sec
TD0                    1

======== CHANNEL f1 ========
NUC1                 13C
P1                 40.00 usec
PL1                -3.00 dB
PL1W         60.64365387 W
SFO1         100.6228298 MHz

======== CHANNEL f2 ========
CPDPRG[2         waltz16
NUC2                  1H
PCPD2              80.00 usec
PL2                -1.00 dB
PL12               14.39 dB
PL13               18.00 dB
PL2W         12.17476940 W
PL12W         0.35193357 W
PL13W         0.15327126 W
SFO2         400.1316005 MHz

F2 - Processing parameters
SI                 32768
SF           100.6128330 MHz
WDW                   EM
SSB                    0
LB                  1.00 Hz
GB                     0
PC                  1.40
```


Supplementary Figure 90. ^{13}C NMR spectrum of **4h** (100 MHz, r.t., $CDCl_3$)

(*R*)-**S2**

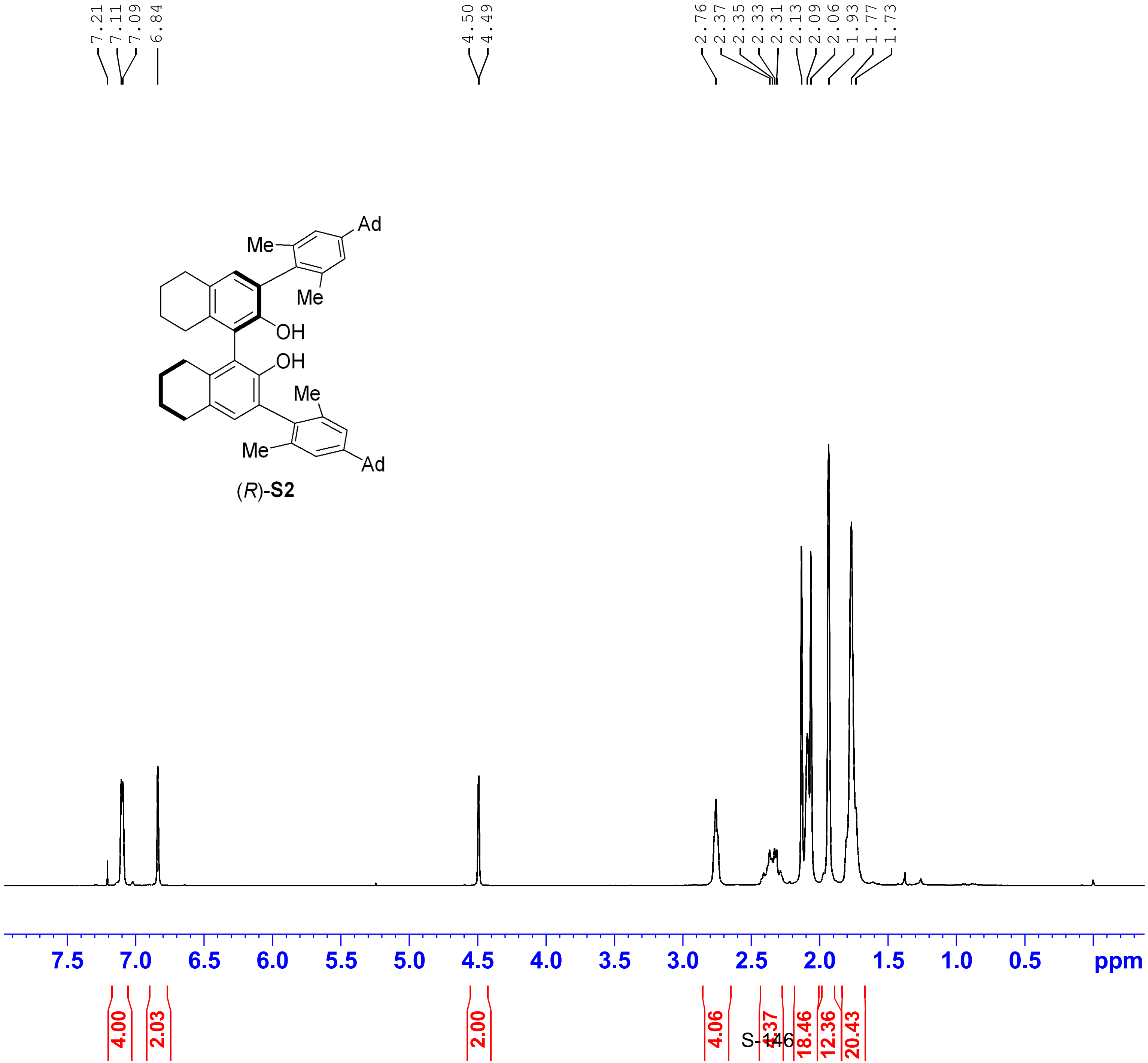


```
Current Data Parameters
NAME       txf-3-49-1nmr
EXPNO                  3
PROCNO                 1

F2 - Acquisition Parameters
Date_           20220322
Time_              18.44
INSTRUM            spect
PROBHD    5 mm PABBO BB/
PULPROG             zg30
TD                 65536
SOLVENT            CDCl3
NS                     4
DS                     0
SWH             8012.820 Hz
FIDRES          0.122266 Hz
AQ             4.0894465 sec
RG                 17.38
DW                62.400 usec
DE                  6.50 usec
TE                 295.5 K
D1            1.00000000 sec
TD0                    1

======== CHANNEL f1 ========
SFO1         400.1324710 MHz
NUC1                  1H
P1                 14.50 usec
PLW1         11.99499989 W

F2 - Processing parameters
SI                 65536
SF           400.1300311 MHz
WDW                   EM
SSB                    0
LB                  0.30 Hz
GB                     0
PC                  1.00
```


Supplementary Figure 91. ^{1}H NMR spectrum of (*R*)-**S2** (400 MHz, r.t., $CDCl_3$)

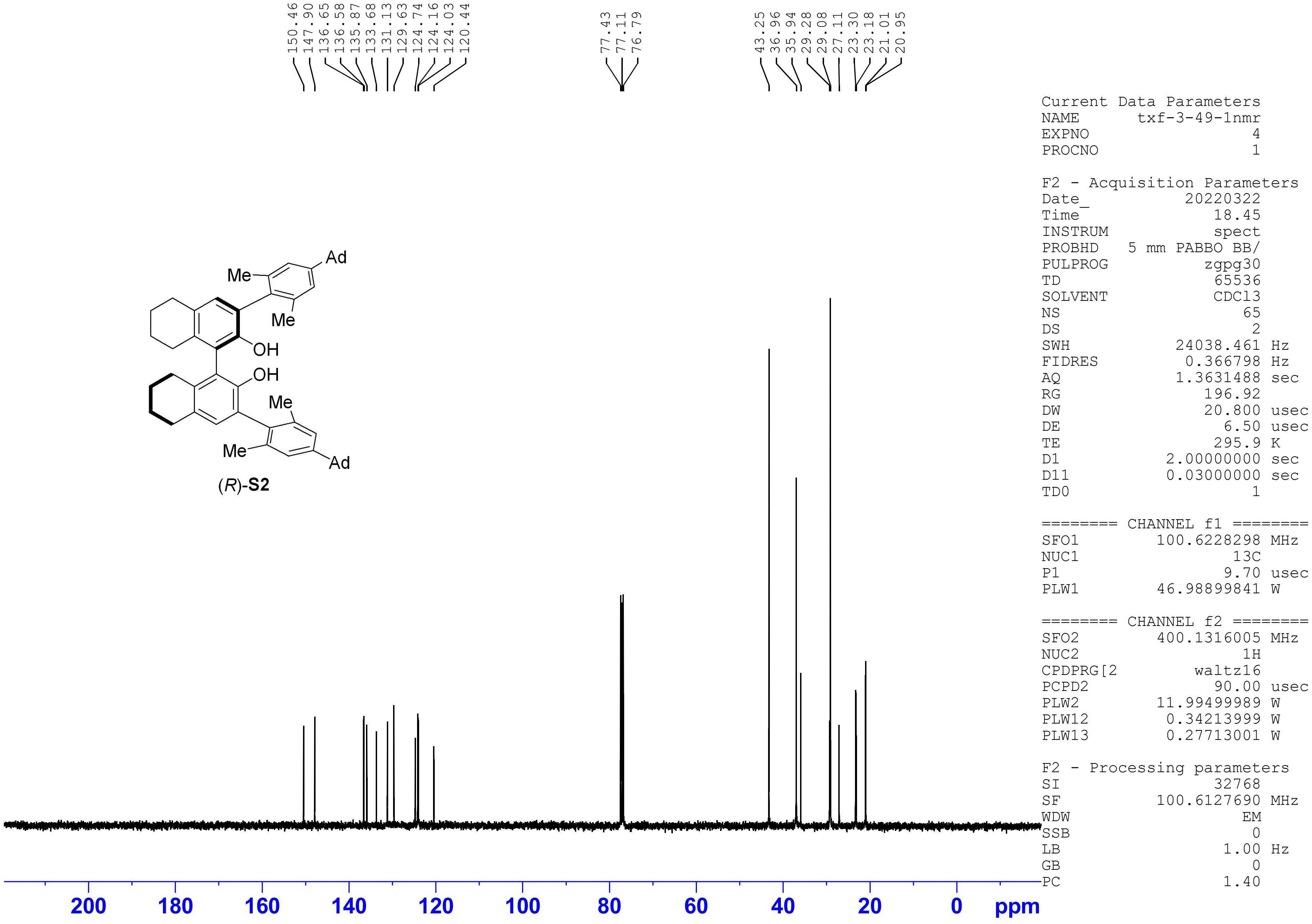

Supplementary Figure 92. ^{13}C NMR spectrum of (*R*)-**S2** (100 MHz, r.t., $CDCl_3$)

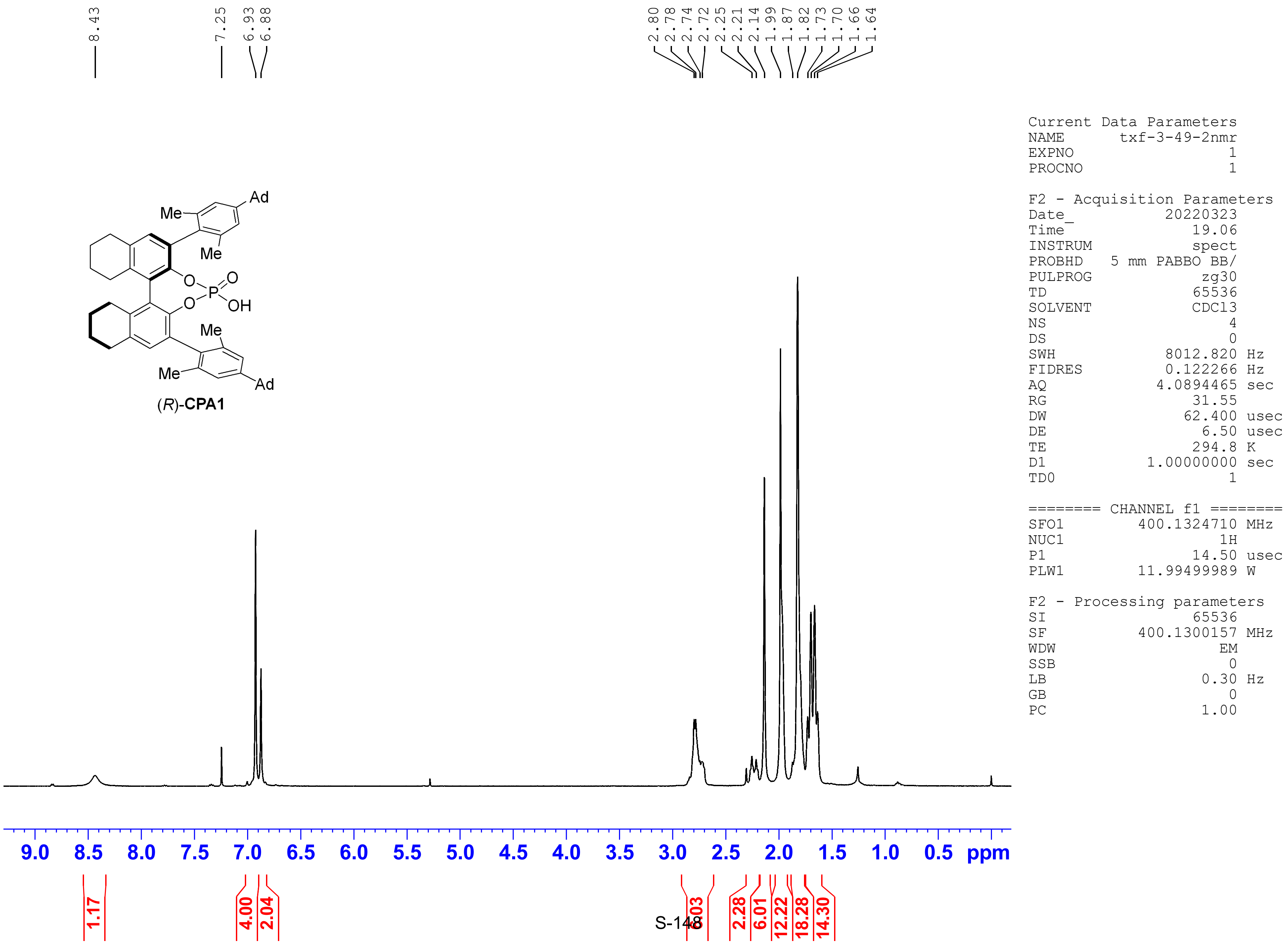

Supplementary Figure 93. ^{1}H NMR spectrum of (*R*)-**CPA1** (400 MHz, r.t., $CDCl_3$)

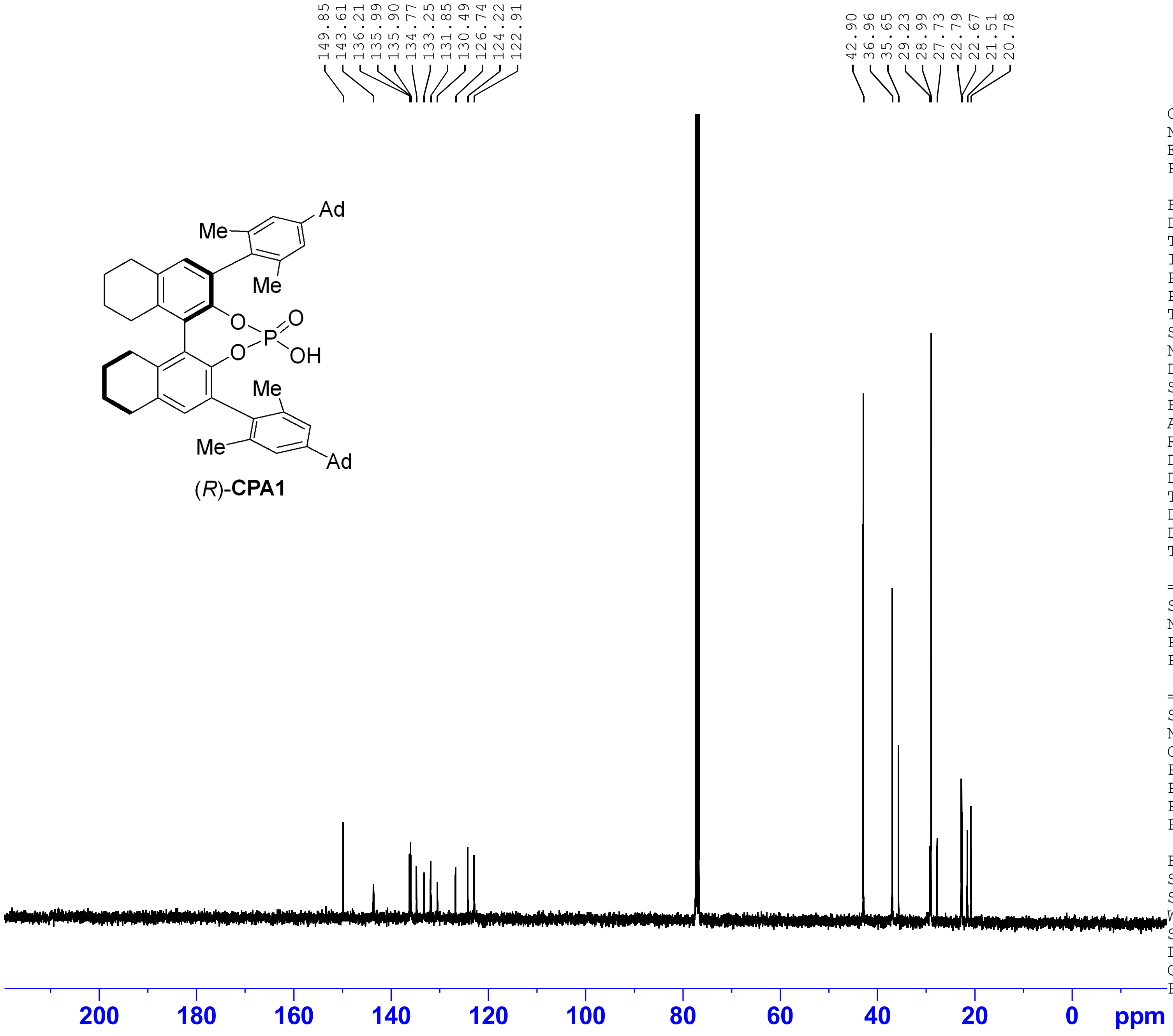

Current Data Parameters
NAME txf-3-49-2nmr
EXPNO 4
PROCNO 1

F2 - Acquisition Parameters
Date_ 20220323
Time 19.27
INSTRUM spect
PROBHD 5 mm PABBO BB/
PULPROG zgpg30
TD 65536
SOLVENT CDCl3
NS 253
DS 0
SWH 24038.461 Hz
FIDRES 0.366798 Hz
AQ 1.3631488 sec
RG 196.92
DW 20.800 usec
DE 6.50 usec
TE 295.5 K
D1 2.00000000 sec
D11 0.03000000 sec
TD0 1

======== CHANNEL f1 ========
SFO1 100.6228298 MHz
NUC1 13C
P1 9.70 usec
PLW1 46.98899841 W

======== CHANNEL f2 ========
SFO2 400.1316005 MHz
NUC2 1H
CPDPRG[2 waltz16
PCPD2 90.00 usec
PLW2 11.99499989 W
PLW12 0.34213999 W
PLW13 0.27713001 W

F2 - Processing parameters
SI 32768
SF 100.6127690 MHz
WDW EM
SSB 0
LB 1.00 Hz
GB 0
PC 1.40

Supplementary Figure 94. ^{13}C NMR spectrum of (*R*)-**CPA1** (100 MHz, r.t., $CDCl_3$)

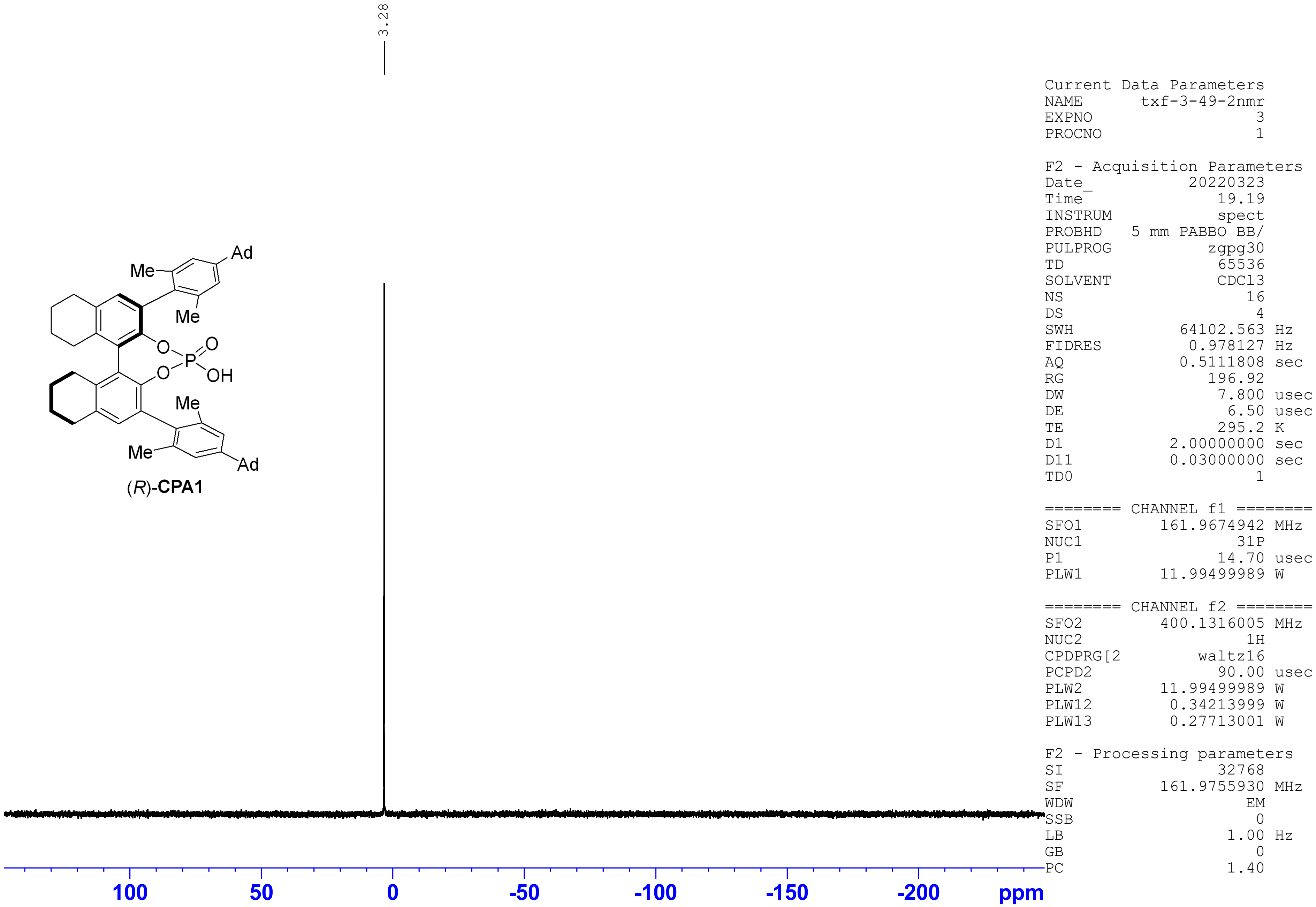

Supplementary Figure 95. ^{31}P NMR spectrum of (*R*)-**CPA1** (162 MHz, r.t., $CDCl_3$)

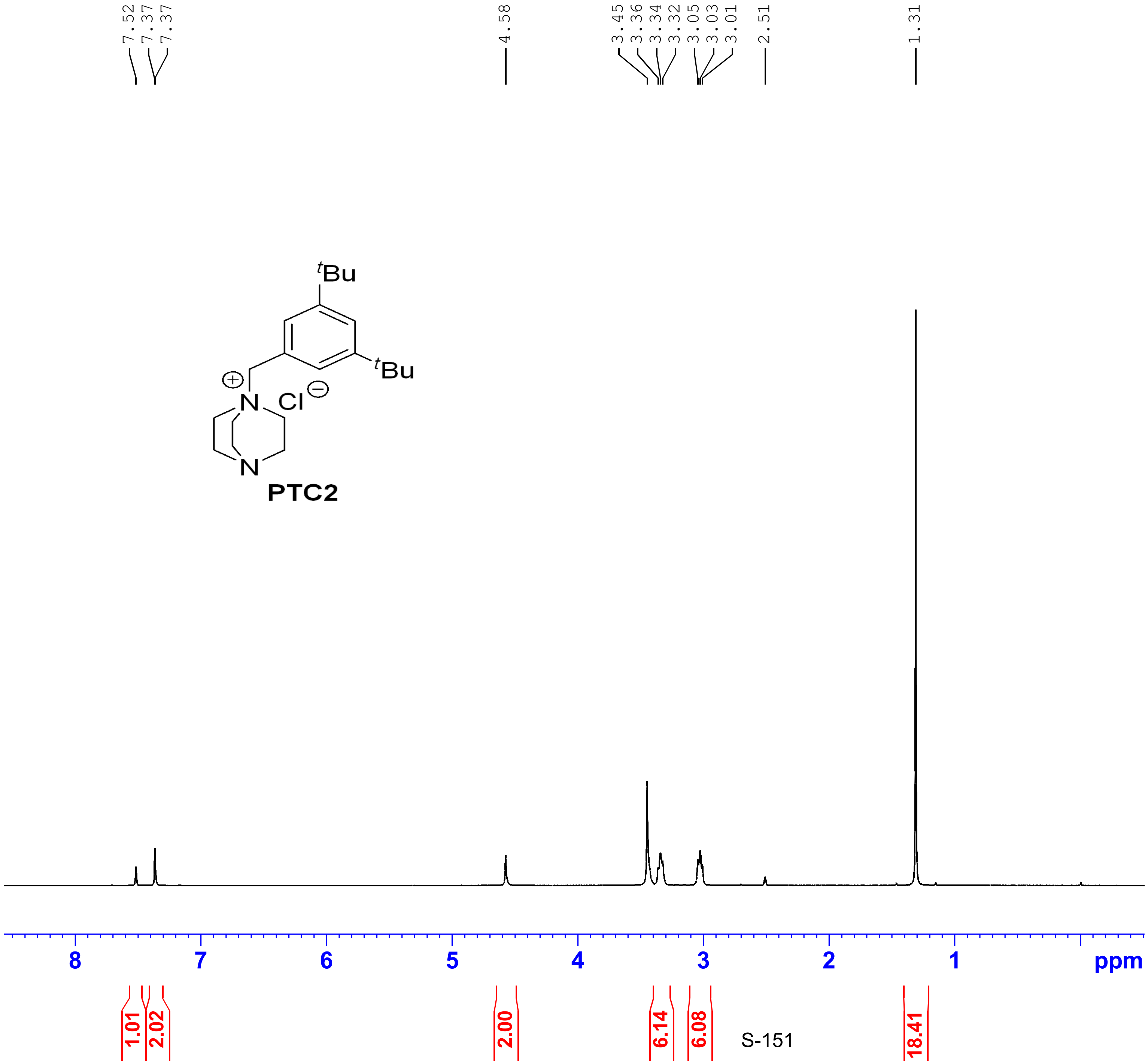

PTC2

```
Current Data Parameters
NAME      txf-3-151-amine-nmr
EXPNO                   1
PROCNO                  1

F2 - Acquisition Parameters
Date_             20220402
Time                 13.10
INSTRUM              spect
PROBHD    5 mm PABBO BB/
PULPROG               zg30
TD                   65536
SOLVENT               DMSO
NS                       4
DS                       0
SWH               8012.820 Hz
FIDRES            0.122266 Hz
AQ               4.0894465 sec
RG                   39.46
DW                  62.400 usec
DE                    6.50 usec
TE                   293.3 K
D1              1.00000000 sec
TD0                      1

======== CHANNEL f1 ========
SFO1           400.1324710 MHz
NUC1                    1H
P1                   14.50 usec
PLW1           11.99499989 W

F2 - Processing parameters
SI                   65536
SF             400.1300000 MHz
WDW                     EM
SSB                      0
LB                    0.30 Hz
GB                       0
PC                    1.00
```


Supplementary Figure 96. ^{1}H NMR spectrum of **PTC2** (400 MHz, r.t., d6-DMSO)

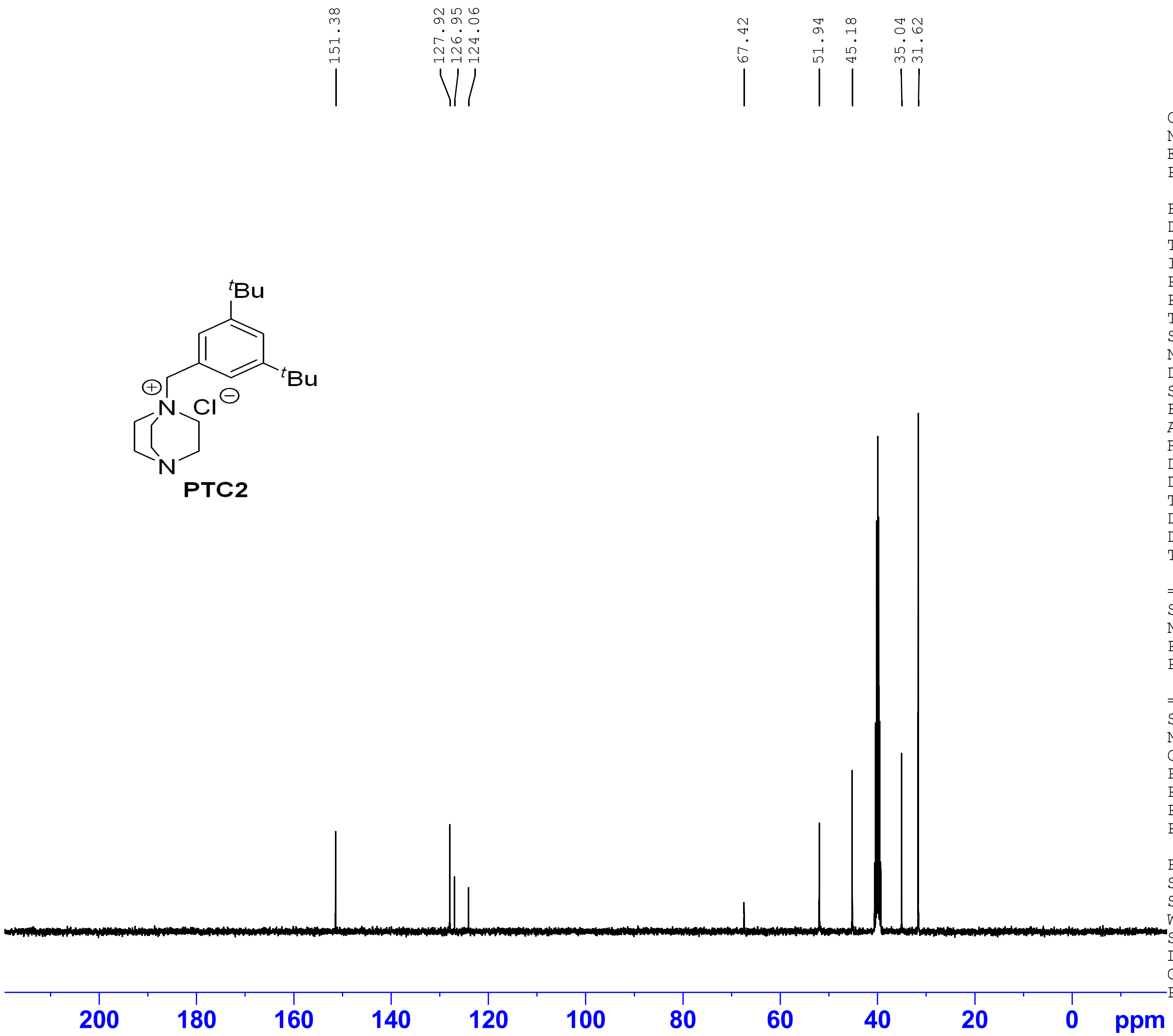

Current Data Parameters
NAME txf-3-151-amine-nmr
EXPNO 2
PROCNO 1

F2 - Acquisition Parameters
Date_ 20220402
Time 13.13
INSTRUM spect
PROBHD 5 mm PABBO BB/
PULPROG zgpg30
TD 65536
SOLVENT DMSO
NS 85
DS 2
SWH 24038.461 Hz
FIDRES 0.366798 Hz
AQ 1.3631488 sec
RG 196.92
DW 20.800 usec
DE 6.50 usec
TE 294.0 K
D1 2.00000000 sec
D11 0.03000000 sec
TD0 1

======== CHANNEL f1 ========
SFO1 100.6228298 MHz
NUC1 13C
P1 9.70 usec
PLW1 46.98899841 W

======== CHANNEL f2 ========
SFO2 400.1316005 MHz
NUC2 1H
CPDPRG[2 waltz16
PCPD2 90.00 usec
PLW2 11.99499989 W
PLW12 0.34213999 W
PLW13 0.27713001 W

F2 - Processing parameters
SI 32768
SF 100.6127690 MHz
WDW EM
SSB 0
LB 1.00 Hz
GB 0
PC 1.40

Supplementary Figure 97. ^{13}C NMR spectrum of **PTC2** (400 MHz, r.t., d6-DMSO)

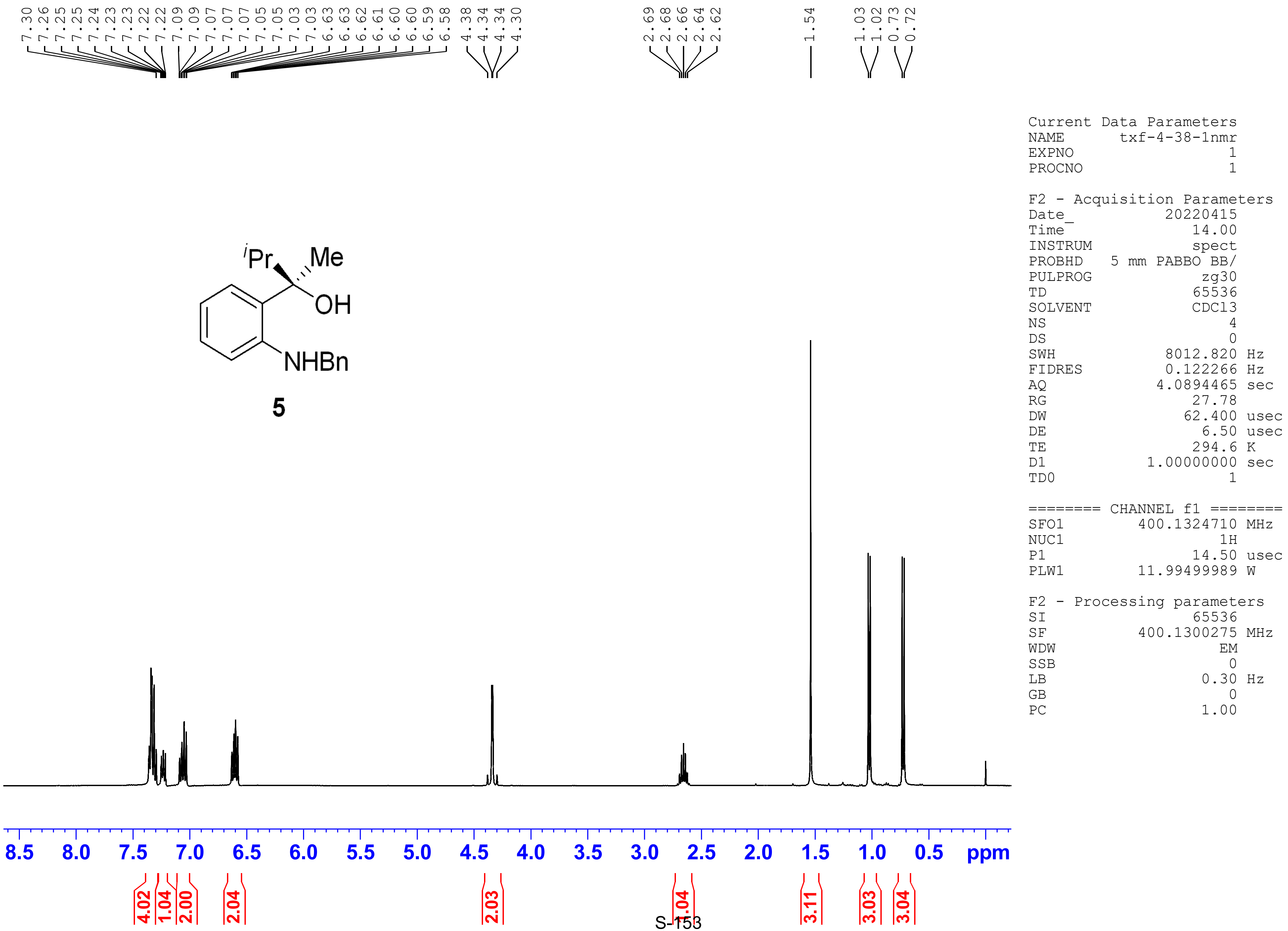

Supplementary Figure 98. ^{1}H NMR spectrum of **5** (400 MHz, r.t., $CDCl_3$)

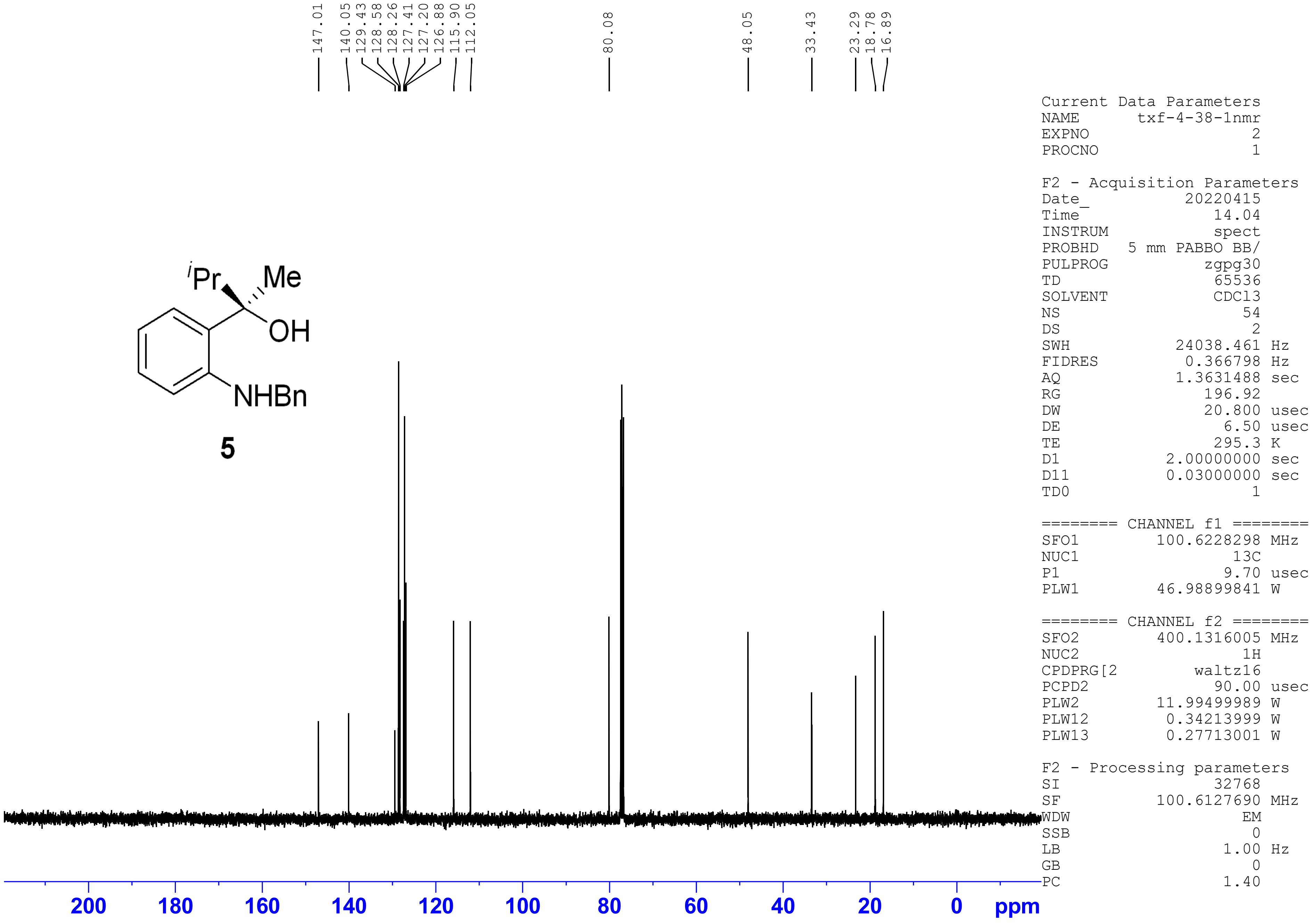

Supplementary Figure 99. ^{13}C NMR spectrum of **5** (400 MHz, r.t., $CDCl_3$)

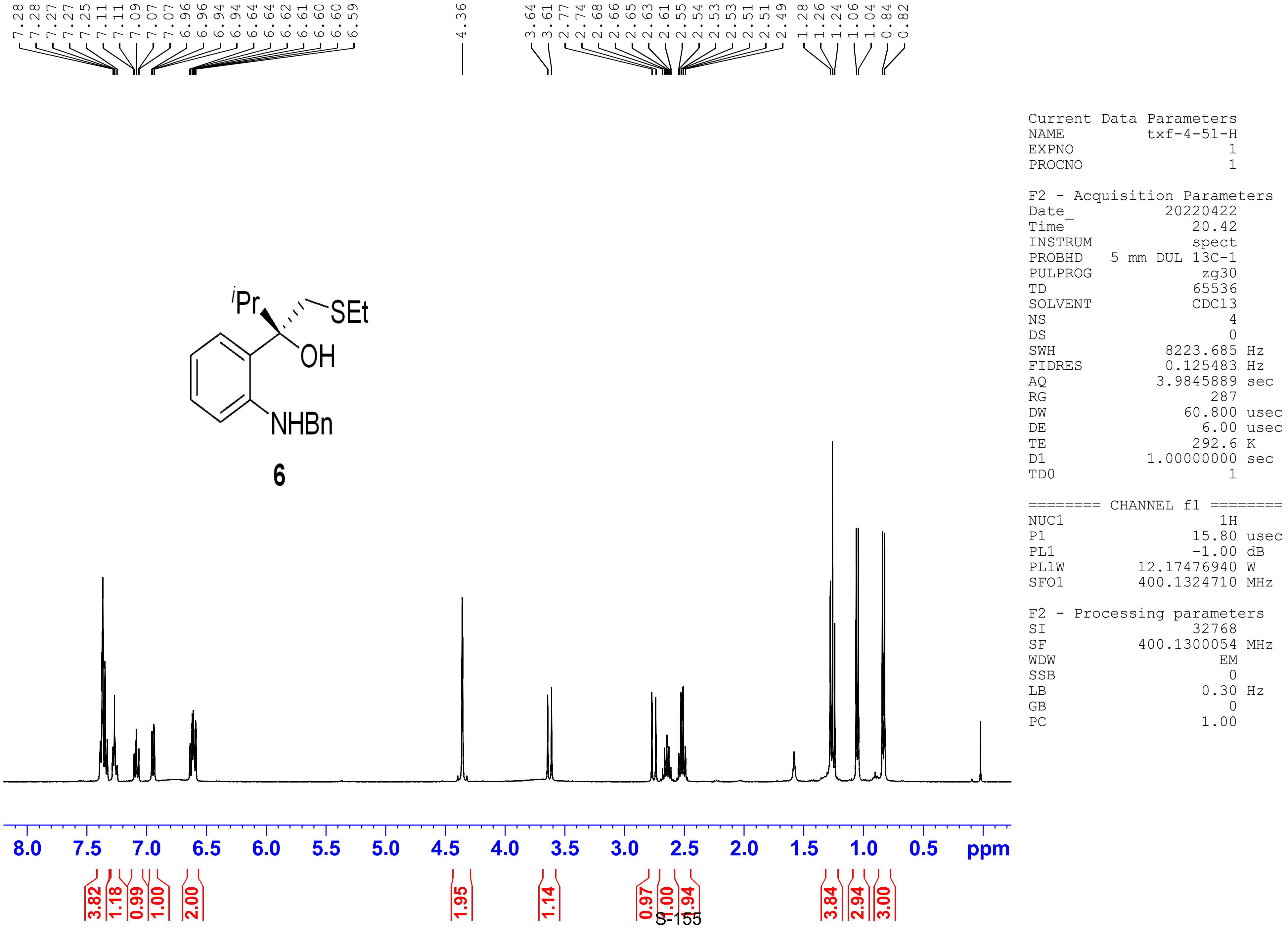

Supplementary Figure 100. ^{1}H NMR spectrum of **6** (400 MHz, r.t., CDCl3)

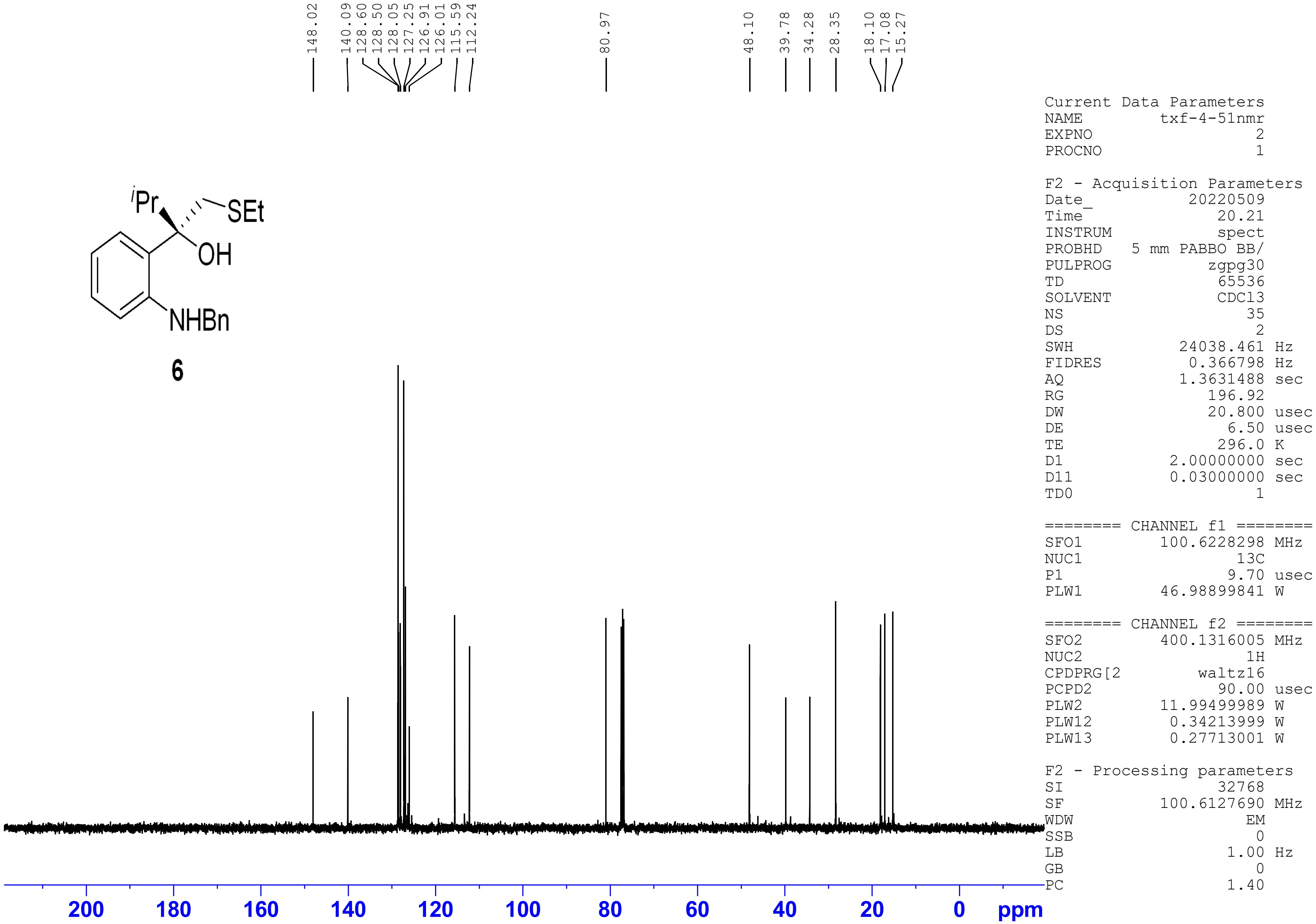

Supplementary Figure 101. ^{13}C NMR spectrum of **6** (400 MHz, r.t., $CDCl_3$)

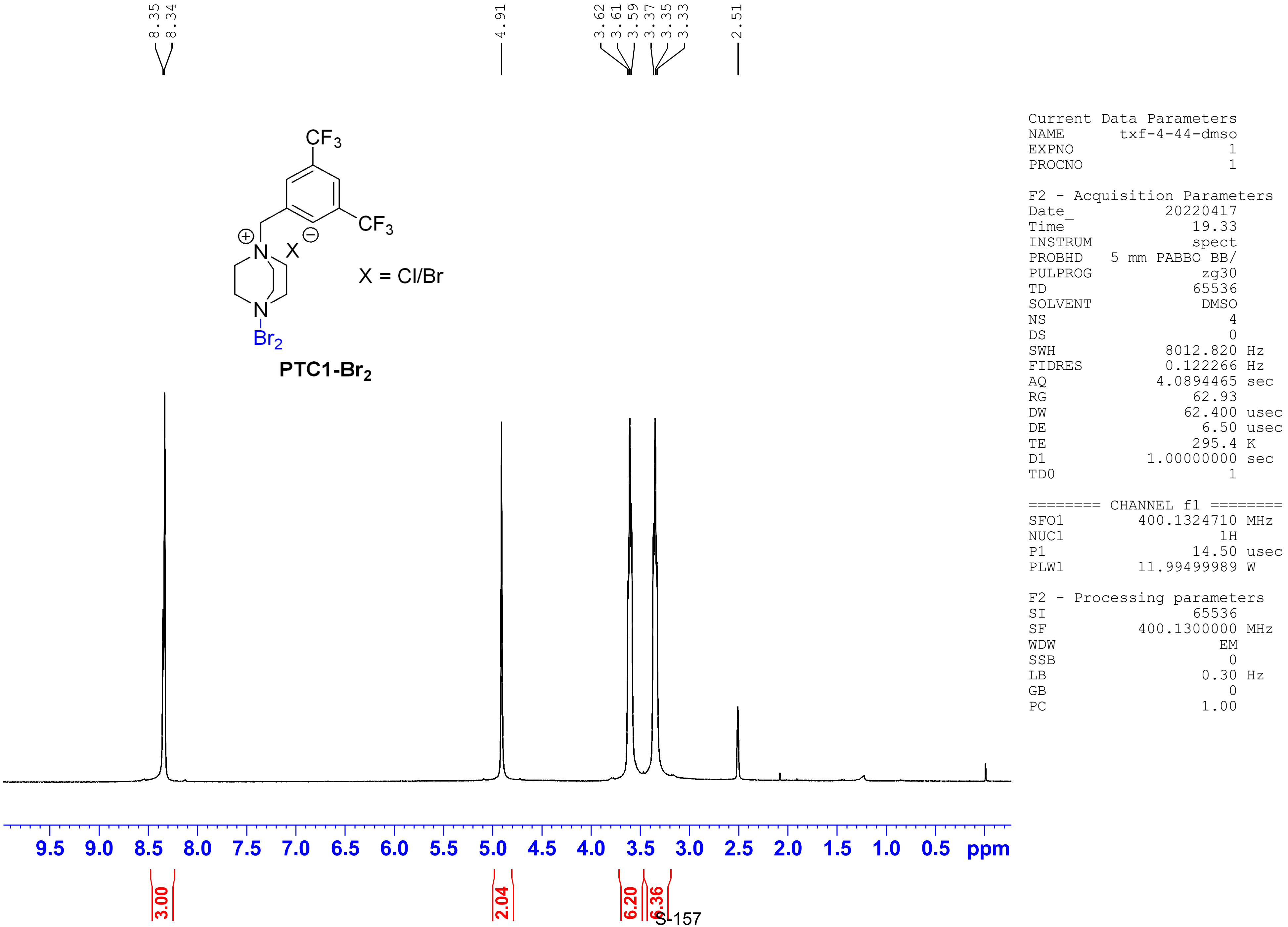

Supplementary Figure 102. ^{1}H NMR spectrum of **PTC1-Br₂** (400 MHz, r.t., DMSO-d_6)

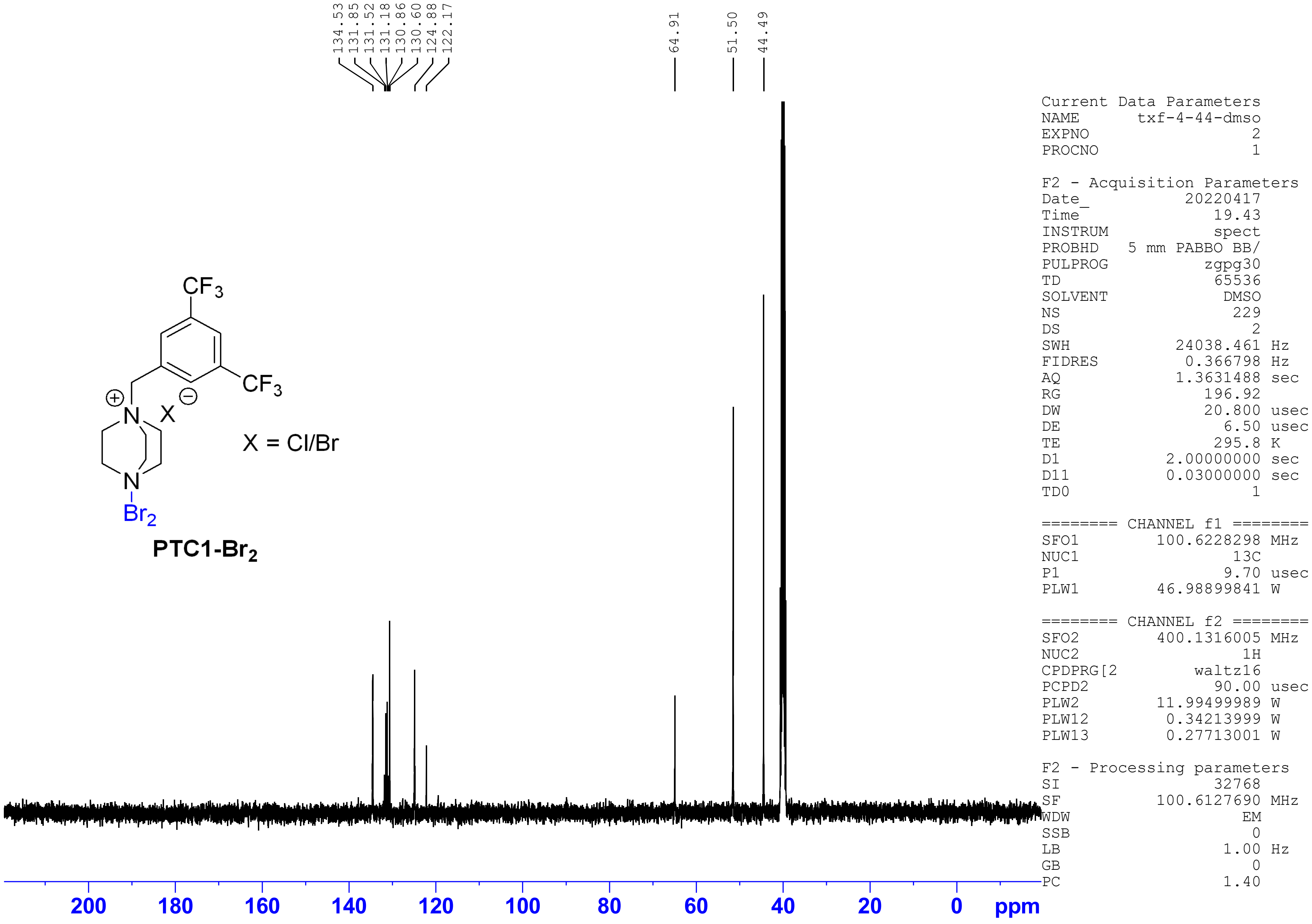

Supplementary Figure 103. ^{13}C NMR spectrum of **PTC1-Br$_2$** (400 MHz, r.t., DMSO-d_6)

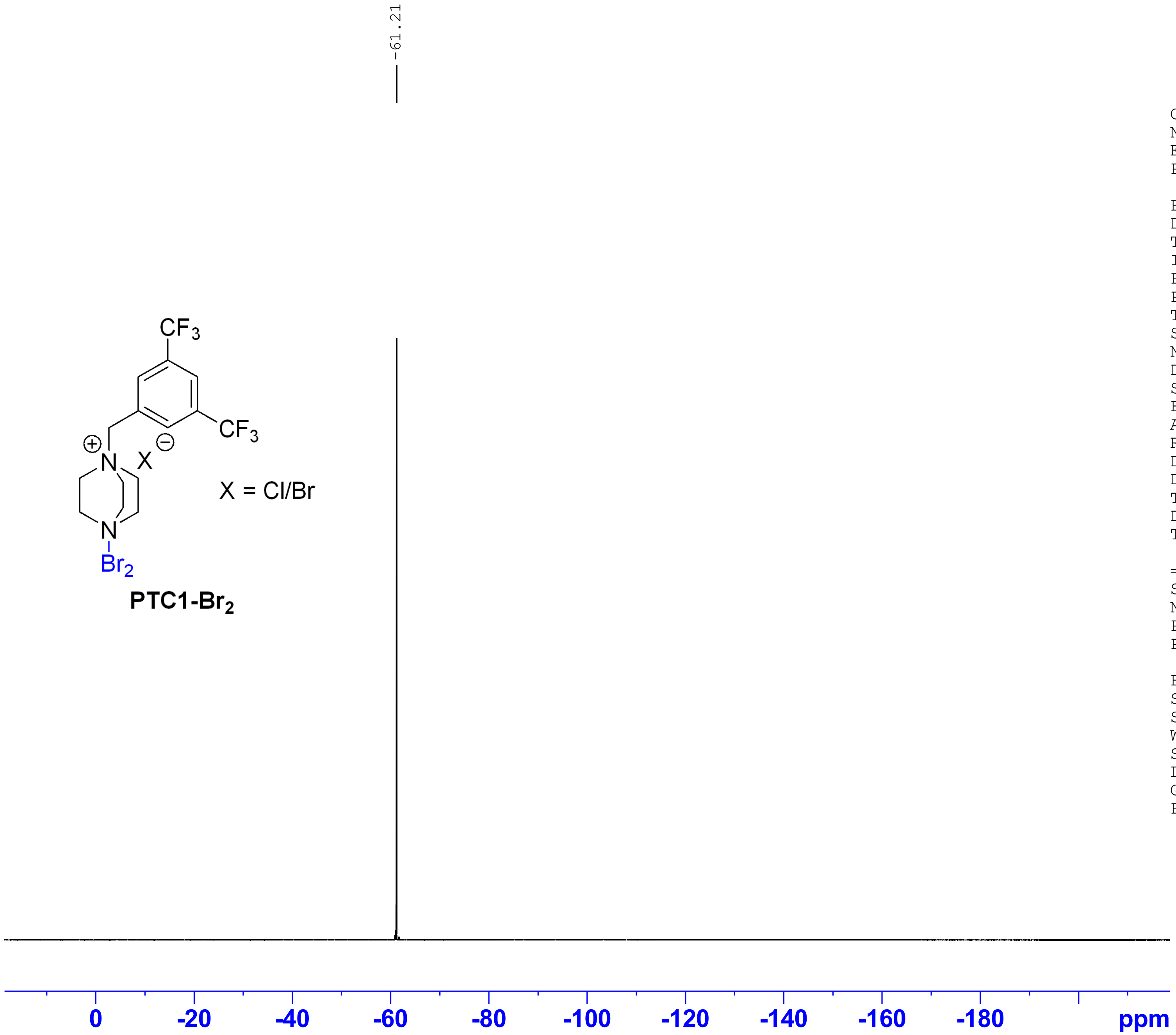


```
Current Data Parameters
NAME      txf-4-44-dmso
EXPNO                 3
PROCNO                1

F2 - Acquisition Parameters
Date_          20220417
Time              19.56
INSTRUM           spect
PROBHD   5 mm PABBO BB/
PULPROG          zgflqn
TD               131072
SOLVENT            DMSO
NS                    8
DS                    4
SWH          89285.711 Hz
FIDRES        0.681196 Hz
AQ           0.7340032 sec
RG               196.92
DW                5.600 usec
DE                 6.50 usec
TE                295.9 K
D1          1.00000000 sec
TD0                   1

======== CHANNEL f1 ========
SFO1       376.4607164 MHz
NUC1                19F
P1                14.70 usec
PLW1       15.99600029 W

F2 - Processing parameters
SI                65536
SF         376.4983660 MHz
WDW                  EM
SSB                   0
LB                 0.30 Hz
GB                    0
PC                 1.00
```


Supplementary Figure 104. ^{19}F NMR spectrum of **PTC1-Br₂** (400 MHz, r.t., DMSO-d_6)

Data File C:\CHEM32\1\DATA\SUN_12 2021-10-14 08-35-36\TXF-3-7-2-IC.D
Sample Name:

===

```
Acq. Operator   :                               Seq. Line :  10
Acq. Instrument : Instrument 1                   Location : Vial 36
Injection Date  : 10/14/2021 10:48:21 AM              Inj :   1
                                              Inj Volume : 5.000 µl
Different Inj Volume from Sequence !     Actual Inj Volume : 2.000 µl
Acq. Method     : C:\CHEM32\1\DATA\SUN_12 2021-10-14 08-35-36\IC-01-20.M
Last changed    : 10/14/2021 11:09:37 AM
                  (modified after loading)
Analysis Method : C:\CHEM32\1\METHODS\OD-03-60-0.6.M
Last changed    : 3/12/2022 7:26:06 PM
                  (modified after loading)
Additional Info : Peak(s) manually integrated
```

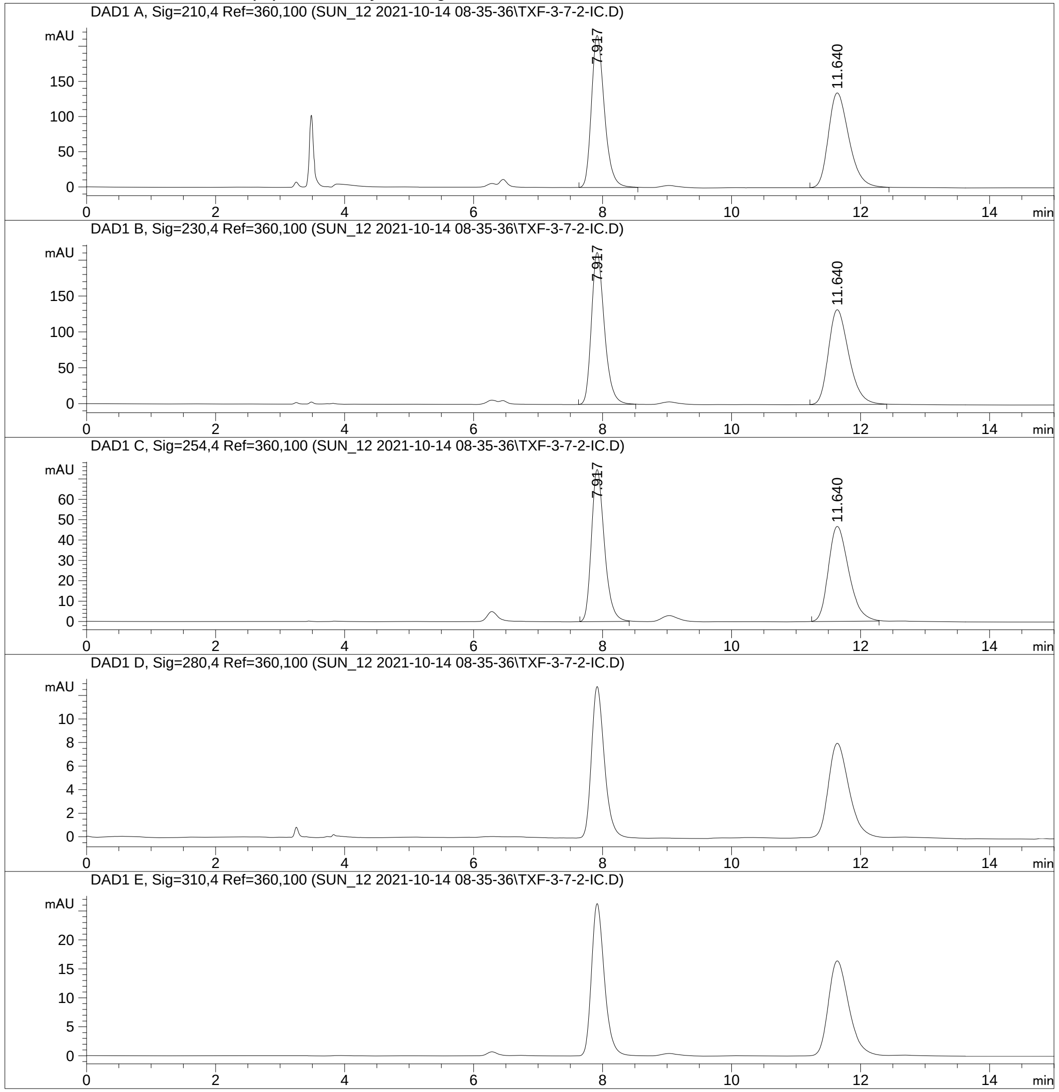

Data File C:\CHEM32\1\DATA\SUN_12 2021-10-14 08-35-36\TXF-3-7-2-IC.D
Sample Name:

===
Area Percent Report
===

```
Sorted By             :      Signal
Multiplier            :      1.0000
Dilution              :      1.0000
Use Multiplier & Dilution Factor with ISTDs
```

Signal 1: DAD1 A, Sig=210,4 Ref=360,100

Peak #	RetTime [min]	Type	Width [min]	Area [mAU*s]	Height [mAU]	Area %
1	7.917	BB	0.2145	3009.96875	216.04666	50.2654
2	11.640	BB	0.3403	2978.18701	134.52495	49.7346

Totals : 5988.15576 350.57161

Signal 2: DAD1 B, Sig=230,4 Ref=360,100

Peak #	RetTime [min]	Type	Width [min]	Area [mAU*s]	Height [mAU]	Area %
1	7.917	BB	0.2143	2946.27563	211.84302	50.2577
2	11.640	BB	0.3394	2916.05664	132.15445	49.7423

Totals : 5862.33228 343.99747

Signal 3: DAD1 C, Sig=254,4 Ref=360,100

Peak #	RetTime [min]	Type	Width [min]	Area [mAU*s]	Height [mAU]	Area %
1	7.917	BB	0.2140	1037.77515	74.72217	50.2760
2	11.640	BB	0.3387	1026.38220	46.64313	49.7240

Totals : 2064.15735 121.36530

Signal 4: DAD1 D, Sig=280,4 Ref=360,100

Signal 5: DAD1 E, Sig=310,4 Ref=360,100

===
*** End of Report ***

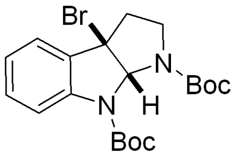

2a
(racemic)

Supplementary Figure 105. HPLC Spectrum of **2a** (racemic)

Data File C:\CHEM32\1\DATA\SUN_12 2021-12-08 09-20-54\TXF-3-66-1.D
Sample Name:

```
=====================================================================
Acq. Operator   :                               Seq. Line :   2
Acq. Instrument : Instrument 1                   Location : Vial 5
Injection Date  : 12/8/2021 9:28:40 AM                 Inj :   1
                                              Inj Volume : 5.000 µl
Different Inj Volume from Sequence !     Actual Inj Volume : 2.000 µl
Acq. Method     : C:\CHEM32\1\DATA\SUN_12 2021-12-08 09-20-54\IC-01-15.M
Last changed    : 10/21/2021 2:45:46 PM
Analysis Method : C:\CHEM32\1\METHODS\OD-03-60-0.6.M
Last changed    : 3/12/2022 7:26:06 PM
                  (modified after loading)
Additional Info : Peak(s) manually integrated
```

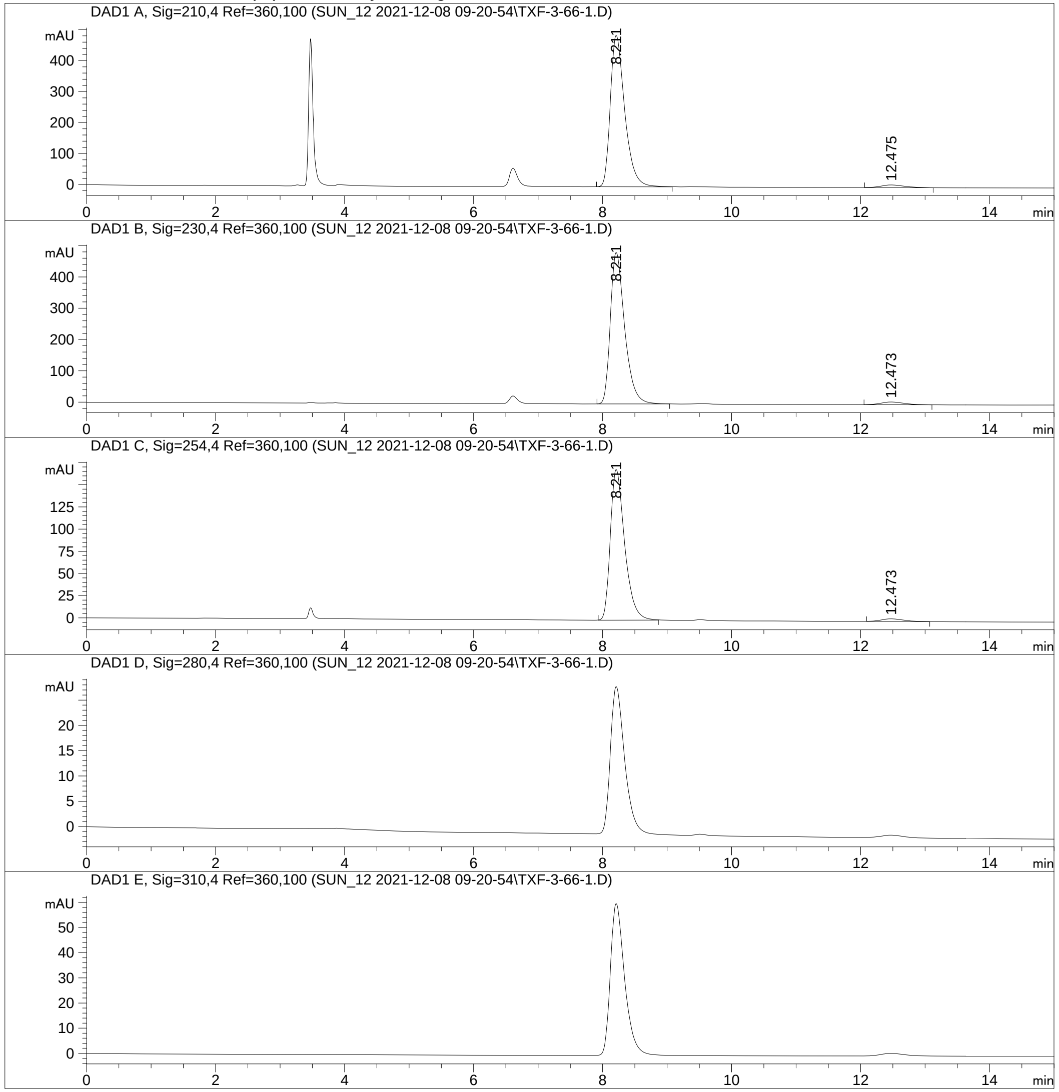

Data File C:\CHEM32\1\DATA\SUN_12 2021-12-08 09-20-54\TXF-3-66-1.D
Sample Name:

```
=====================================================================
                         Area Percent Report
=====================================================================

Sorted By             :      Signal
Multiplier            :      1.0000
Dilution              :      1.0000
Use Multiplier & Dilution Factor with ISTDs
```

Signal 1: DAD1 A, Sig=210,4 Ref=360,100

Peak #	RetTime [min]	Type	Width [min]	Area [mAU*s]	Height [mAU]	Area %
1	8.211	BB	0.2350	7582.03467	488.62357	97.1440
2	12.475	BB	0.3741	222.90654	8.78150	2.8560

Totals : 7804.94121 497.40507

Signal 2: DAD1 B, Sig=230,4 Ref=360,100

Peak #	RetTime [min]	Type	Width [min]	Area [mAU*s]	Height [mAU]	Area %
1	8.211	BB	0.2341	7466.31104	483.74875	97.1932
2	12.473	BB	0.3822	215.61504	8.72887	2.8068

Totals : 7681.92607 492.47762

Signal 3: DAD1 C, Sig=254,4 Ref=360,100

Peak #	RetTime [min]	Type	Width [min]	Area [mAU*s]	Height [mAU]	Area %
1	8.211	BB	0.2340	2618.59448	169.71388	97.2813
2	12.473	BB	0.3725	73.18083	3.02238	2.7187

Totals : 2691.77531 172.73626

Signal 4: DAD1 D, Sig=280,4 Ref=360,100

Signal 5: DAD1 E, Sig=310,4 Ref=360,100

===
*** End of Report ***

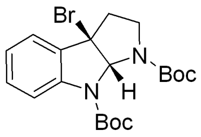

2a
(enantioenriched)

Supplementary Figure 106. HPLC Spectrum of **2a** (enantioenriched)

Data File C:\CHEM32\1\DATA\SUN_12 2021-12-10 20-57-47\TXF-3-70-2RAC.D
Sample Name:

```
=====================================================================
Acq. Operator   :                              Seq. Line :   7
Acq. Instrument : Instrument 1                  Location : Vial 1
Injection Date  : 12/10/2021 10:43:38 PM             Inj :   1
                                              Inj Volume : 5.000 µl
Different Inj Volume from Sequence !     Actual Inj Volume : 2.000 µl
Acq. Method     : C:\CHEM32\1\DATA\SUN_12 2021-12-10 20-57-47\IC-20-40.M
Last changed    : 12/10/2021 10:42:45 PM
                  (modified after loading)
Analysis Method : C:\CHEM32\1\METHODS\OD-03-60-0.6.M
Last changed    : 3/12/2022 7:32:45 PM
                  (modified after loading)
Additional Info : Peak(s) manually integrated
```

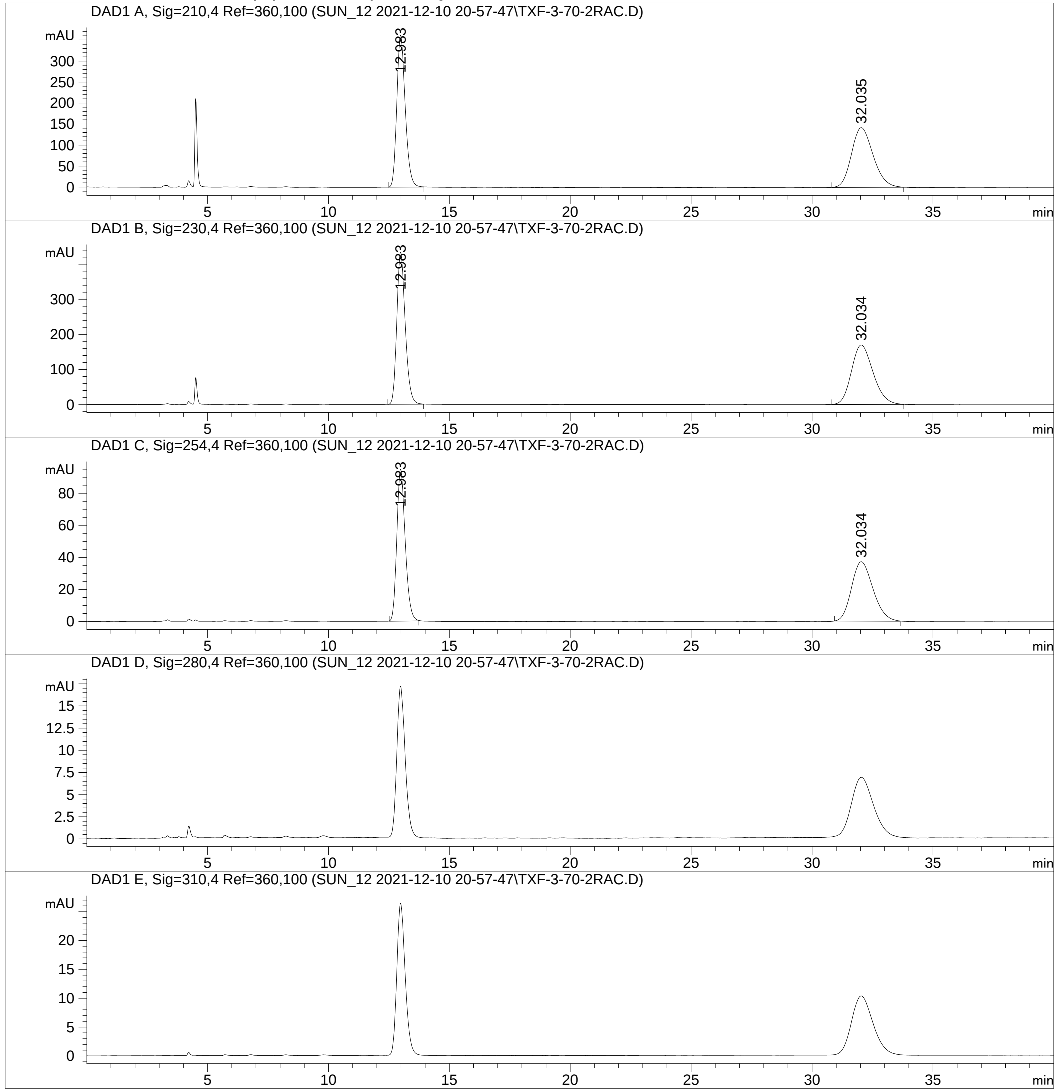

Data File C:\CHEM32\1\DATA\SUN_12 2021-12-10 20-57-47\TXF-3-70-2RAC.D
Sample Name:

Area Percent Report

```
Sorted By             :      Signal
Multiplier            :      1.0000
Dilution              :      1.0000
Use Multiplier & Dilution Factor with ISTDs
```

Signal 1: DAD1 A, Sig=210,4 Ref=360,100

Peak #	RetTime [min]	Type	Width [min]	Area [mAU*s]	Height [mAU]	Area %
1	12.983	BB	0.3739	8800.21680	361.69934	49.7727
2	32.035	BB	0.9445	8880.59961	142.21005	50.2273

Totals : 1.76808e4 503.90939

Signal 2: DAD1 B, Sig=230,4 Ref=360,100

Peak #	RetTime [min]	Type	Width [min]	Area [mAU*s]	Height [mAU]	Area %
1	12.983	BB	0.3732	1.05136e4	433.16977	49.8510
2	32.034	BB	0.9662	1.05765e4	169.45087	50.1490

Totals : 2.10901e4 602.62064

Signal 3: DAD1 C, Sig=254,4 Ref=360,100

Peak #	RetTime [min]	Type	Width [min]	Area [mAU*s]	Height [mAU]	Area %
1	12.983	BB	0.3726	2295.22559	94.73809	49.9650
2	32.034	BB	0.9511	2298.44141	37.08369	50.0350

Totals : 4593.66699 131.82178

Signal 4: DAD1 D, Sig=280,4 Ref=360,100

Signal 5: DAD1 E, Sig=310,4 Ref=360,100

*** End of Report ***

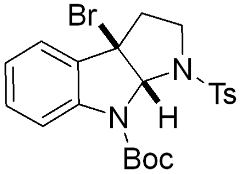

2b
(racemic)

Supplementary Figure 107. HPLC Spectrum of **2b** (racemic)

```
Data File C:\CHEM32\1\DATA\SUN_12 2021-12-10 20-57-47\TXF-3-70-3.D
Sample Name:

    =====================================================================
    Acq. Operator   :                                Seq. Line :   8
    Acq. Instrument : Instrument 1                    Location : Vial 7
    Injection Date  : 12/10/2021 11:24:36 PM               Inj :   1
                                                    Inj Volume : 5.000 µl
    Different Inj Volume from Sequence !     Actual Inj Volume : 2.000 µl
    Acq. Method     : C:\CHEM32\1\DATA\SUN_12 2021-12-10 20-57-47\IC-20-40.M
    Last changed    : 12/10/2021 10:42:45 PM
                      (modified after loading)
    Analysis Method : C:\CHEM32\1\METHODS\OD-03-60-0.6.M
    Last changed    : 3/12/2022 7:32:45 PM
                      (modified after loading)
    Additional Info : Peak(s) manually integrated
```

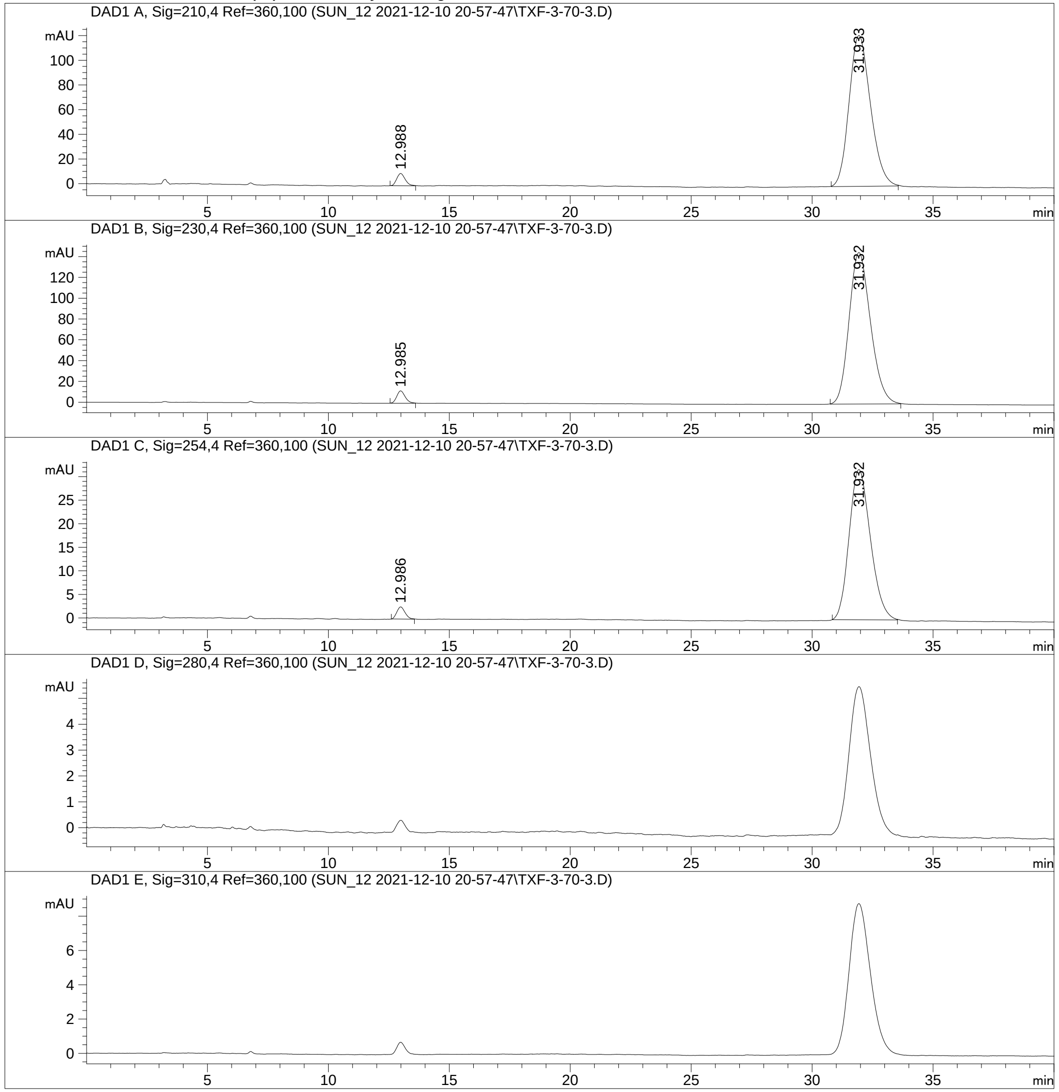

Data File C:\CHEM32\1\DATA\SUN_12 2021-12-10 20-57-47\TXF-3-70-3.D
Sample Name:

```
=====================================================================
                          Area Percent Report
=====================================================================

Sorted By             :      Signal
Multiplier            :      1.0000
Dilution              :      1.0000
Use Multiplier & Dilution Factor with ISTDs
```

Signal 1: DAD1 A, Sig=210,4 Ref=360,100

Peak #	RetTime [min]	Type	Width [min]	Area [mAU*s]	Height [mAU]	Area %
1	12.988	BB	0.3595	242.86293	9.99470	3.1224
2	31.933	BB	0.9231	7535.20947	122.24580	96.8776

Totals : 7778.07240 132.24050

Signal 2: DAD1 B, Sig=230,4 Ref=360,100

Peak #	RetTime [min]	Type	Width [min]	Area [mAU*s]	Height [mAU]	Area %
1	12.985	BB	0.3724	284.75186	11.84668	3.0682
2	31.932	BB	0.9500	8996.04492	145.76164	96.9318

Totals : 9280.79678 157.60832

Signal 3: DAD1 C, Sig=254,4 Ref=360,100

Peak #	RetTime [min]	Type	Width [min]	Area [mAU*s]	Height [mAU]	Area %
1	12.986	BB	0.3605	61.18916	2.58224	3.0338
2	31.932	BB	0.9412	1955.74792	31.90483	96.9662

Totals : 2016.93709 34.48707

Signal 4: DAD1 D, Sig=280,4 Ref=360,100

Signal 5: DAD1 E, Sig=310,4 Ref=360,100

===
*** End of Report ***

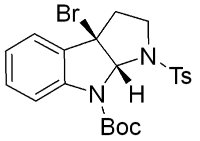

2b
(enantioenriched)

Supplementary Figure 108. HPLC Spectrum of **2b** (enantioenriched)

Data File C:\CHEM32\1\DATA\SUN_12 2021-12-11 20-22-58\TXF-3-74-2RAC.D
Sample Name:

```
=====================================================================
Acq. Operator   :                        Seq. Line :   2
Acq. Instrument : Instrument 1            Location : Vial 1
Injection Date  : 12/11/2021 8:35:49 PM        Inj :   1
                                        Inj Volume : 5.000 µl
Different Inj Volume from Sequence !     Actual Inj Volume : 2.000 µl
Acq. Method     : C:\CHEM32\1\DATA\SUN_12 2021-12-11 20-22-58\IC-10-15.M
Last changed    : 12/11/2021 8:34:57 PM
                  (modified after loading)
Analysis Method : C:\CHEM32\1\METHODS\OD-03-60-0.6.M
Last changed    : 3/12/2022 7:32:45 PM
                  (modified after loading)
Additional Info : Peak(s) manually integrated
```

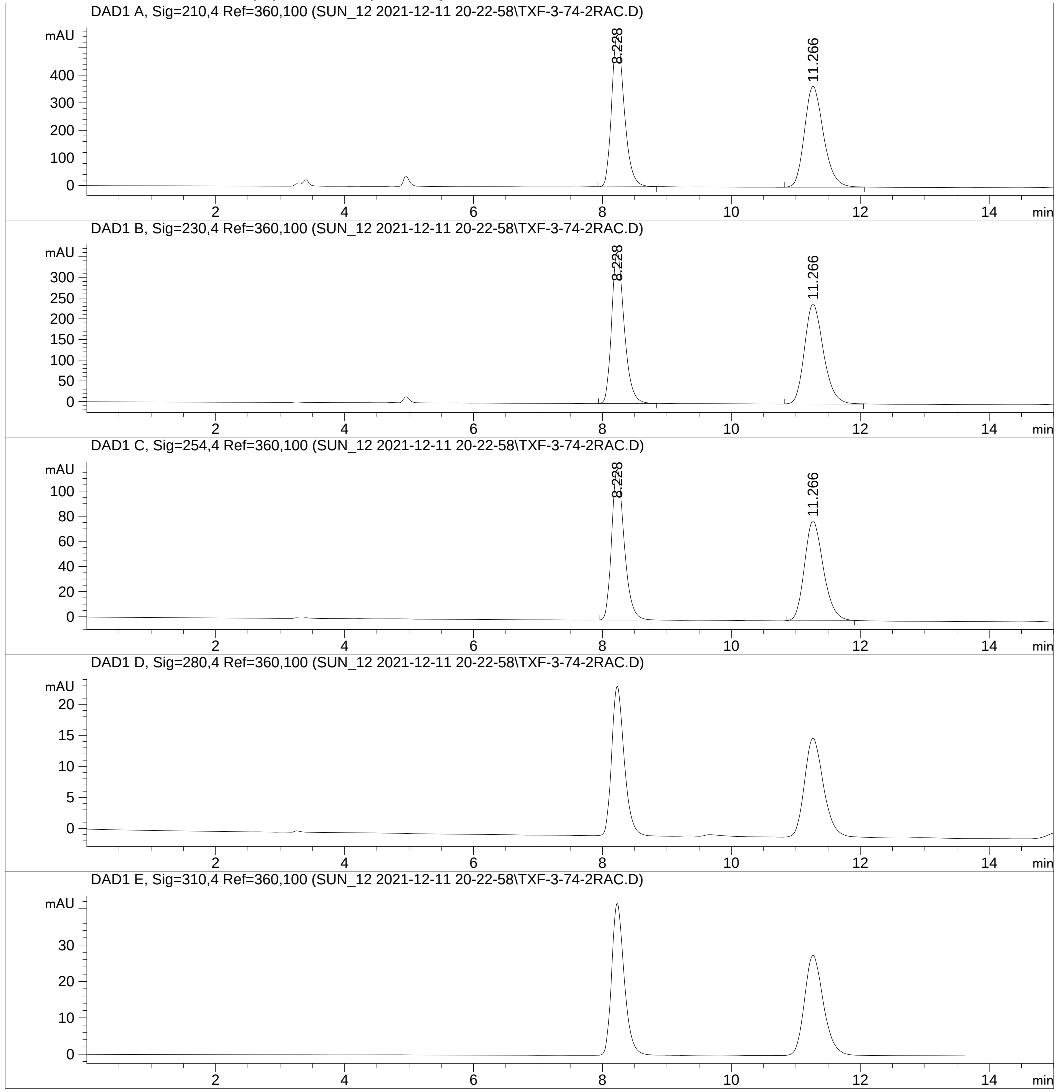

Data File C:\CHEM32\1\DATA\SUN_12 2021-12-11 20-22-58\TXF-3-74-2RAC.D
Sample Name:

Area Percent Report

Sorted By : Signal
Multiplier : 1.0000
Dilution : 1.0000
Use Multiplier & Dilution Factor with ISTDs

Signal 1: DAD1 A, Sig=210,4 Ref=360,100

Peak #	RetTime [min]	Type	Width [min]	Area [mAU*s]	Height [mAU]	Area %
1	8.228	VB	0.2084	7464.97461	549.87280	49.8095
2	11.266	BB	0.3159	7522.08936	365.82117	50.1905
Totals :				1.49871e4	915.69397	

Signal 2: DAD1 B, Sig=230,4 Ref=360,100

Peak #	RetTime [min]	Type	Width [min]	Area [mAU*s]	Height [mAU]	Area %
1	8.228	BB	0.2077	4935.38623	365.17322	49.9240
2	11.266	BB	0.3149	4950.40967	241.71548	50.0760
Totals :				9885.79590	606.88870	

Signal 3: DAD1 C, Sig=254,4 Ref=360,100

Peak #	RetTime [min]	Type	Width [min]	Area [mAU*s]	Height [mAU]	Area %
1	8.228	BB	0.2078	1622.82263	120.01346	49.9832
2	11.266	BB	0.3143	1623.91467	79.51582	50.0168
Totals :				3246.73730	199.52928	

Signal 4: DAD1 D, Sig=280,4 Ref=360,100

Signal 5: DAD1 E, Sig=310,4 Ref=360,100

*** End of Report ***

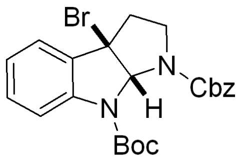

2c
(racemic)

Supplementary Figure 109. HPLC Spectrum of **2c** (racemic)

===

Acq. Operator : Seq. Line : 3
Acq. Instrument : Instrument 1 Location : Vial 2
Injection Date : 12/11/2021 8:51:45 PM Inj : 1
Inj Volume : 5.000 µl
Different Inj Volume from Sequence ! Actual Inj Volume : 2.000 µl
Acq. Method : C:\CHEM32\1\DATA\SUN_12 2021-12-11 20-22-58\IC-10-15.M
Last changed : 12/11/2021 8:34:57 PM
(modified after loading)
Analysis Method : C:\CHEM32\1\METHODS\OD-03-60-0.6.M
Last changed : 3/12/2022 7:32:45 PM
(modified after loading)
Additional Info : Peak(s) manually integrated

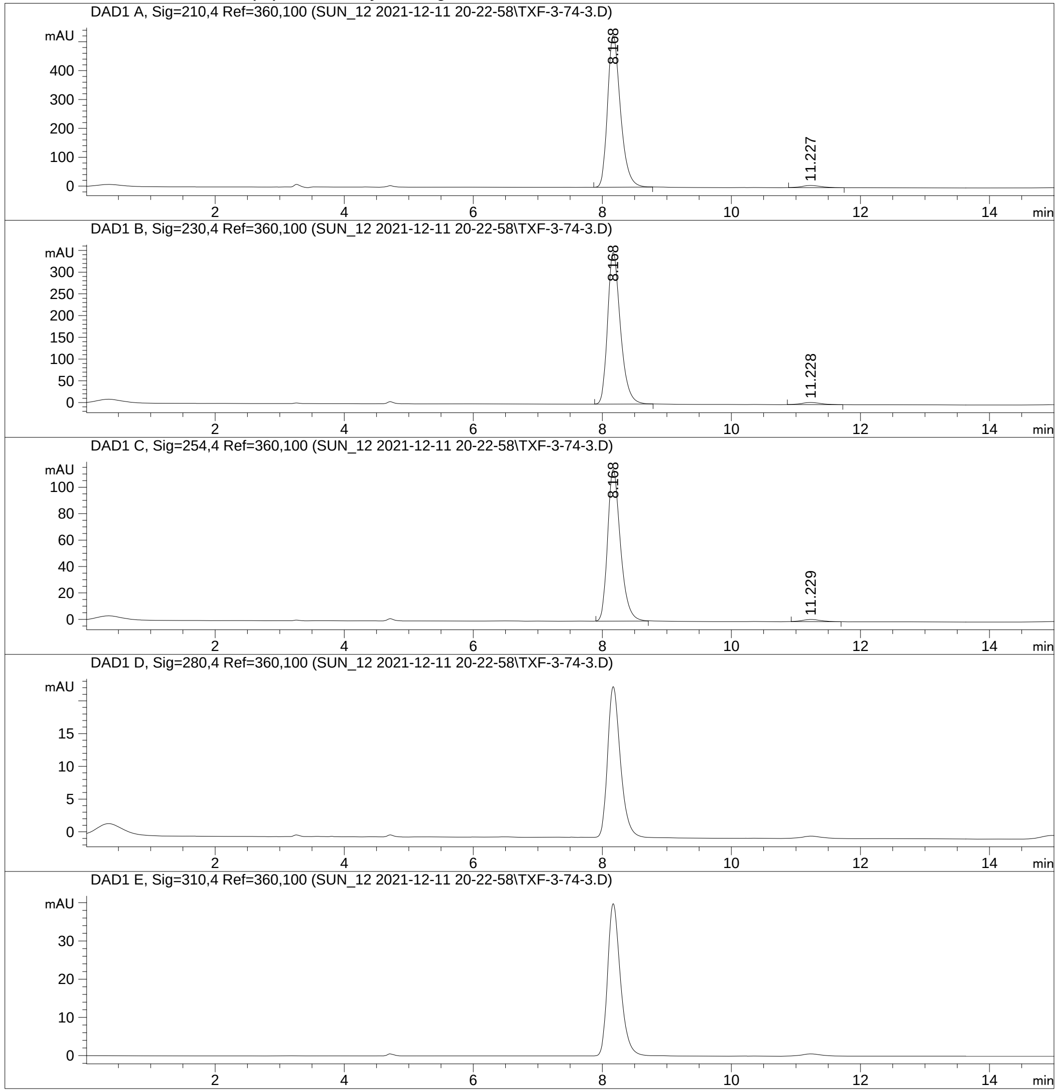

Data File C:\CHEM32\1\DATA\SUN_12 2021-12-11 20-22-58\TXF-3-74-3.D
Sample Name:

===

Area Percent Report

===

Sorted By : Signal
Multiplier : 1.0000
Dilution : 1.0000
Use Multiplier & Dilution Factor with ISTDs

Signal 1: DAD1 A, Sig=210,4 Ref=360,100

Peak #	RetTime [min]	Type	Width [min]	Area [mAU*s]	Height [mAU]	Area %
1	8.168	BB	0.2113	7267.67041	525.70020	97.8844
2	11.227	BB	0.3042	157.07608	7.82442	2.1156

Totals : 7424.74649 533.52461

Signal 2: DAD1 B, Sig=230,4 Ref=360,100

Peak #	RetTime [min]	Type	Width [min]	Area [mAU*s]	Height [mAU]	Area %
1	8.168	BB	0.2107	4803.59814	348.75833	97.9357
2	11.228	BB	0.3025	101.25163	5.12462	2.0643

Totals : 4904.84977 353.88295

Signal 3: DAD1 C, Sig=254,4 Ref=360,100

Peak #	RetTime [min]	Type	Width [min]	Area [mAU*s]	Height [mAU]	Area %
1	8.168	BB	0.2108	1580.17993	114.68114	97.9624
2	11.229	BB	0.2978	32.86703	1.68363	2.0376

Totals : 1613.04696 116.36477

Signal 4: DAD1 D, Sig=280,4 Ref=360,100

Signal 5: DAD1 E, Sig=310,4 Ref=360,100

===

*** End of Report ***

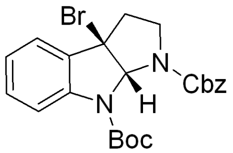

2c
(enantioenriched)

Supplementary Figure 110. HPLC Spectrum of **2c** (enantioenriched)

Data File C:\CHEM32\1\DATA\SUN_12 2021-12-11 20-22-58\TXF-3-75-2RAC.D
Sample Name:

```
=====================================================================
Acq. Operator   :                               Seq. Line :   4
Acq. Instrument : Instrument 1                   Location : Vial 3
Injection Date  : 12/11/2021 9:07:42 PM               Inj :   1
                                               Inj Volume : 5.000 µl
Different Inj Volume from Sequence !     Actual Inj Volume : 2.000 µl
Acq. Method     : C:\CHEM32\1\DATA\SUN_12 2021-12-11 20-22-58\IC-10-15.M
Last changed    : 12/11/2021 8:34:57 PM
                  (modified after loading)
Analysis Method : C:\CHEM32\1\METHODS\OD-03-60-0.6.M
Last changed    : 3/12/2022 7:32:45 PM
                  (modified after loading)
Additional Info : Peak(s) manually integrated
```

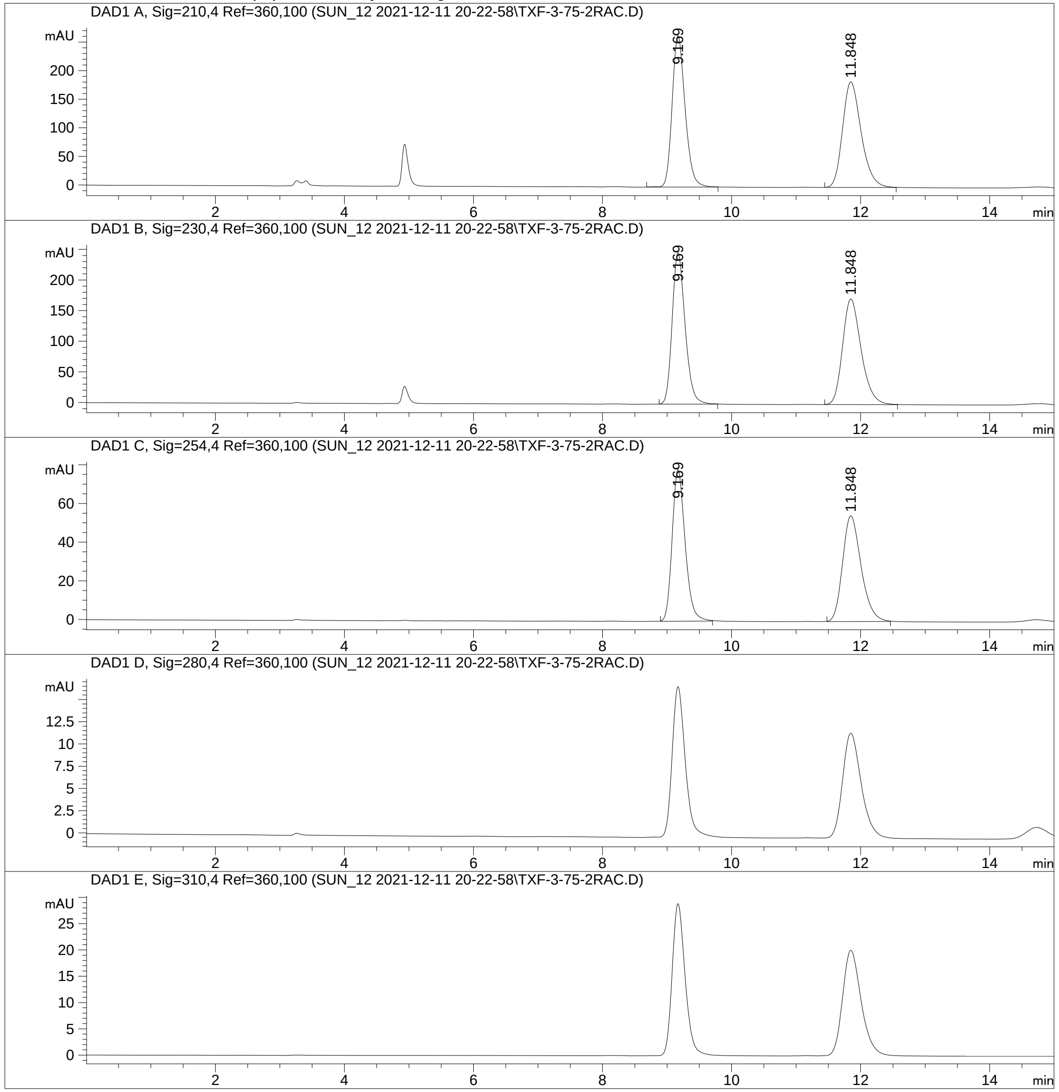

Data File C:\CHEM32\1\DATA\SUN_12 2021-12-11 20-22-58\TXF-3-75-2RAC.D
Sample Name:

Area Percent Report

Sorted By : Signal
Multiplier : 1.0000
Dilution : 1.0000
Use Multiplier & Dilution Factor with ISTDs

Signal 1: DAD1 A, Sig=210,4 Ref=360,100

Peak #	RetTime [min]	Type	Width [min]	Area [mAU*s]	Height [mAU]	Area %
1	9.169	BB	0.2147	3701.99658	265.42279	49.9865
2	11.848	BB	0.3058	3703.99512	184.82404	50.0135
Totals :				7405.99170	450.24683	

Signal 2: DAD1 B, Sig=230,4 Ref=360,100

Peak #	RetTime [min]	Type	Width [min]	Area [mAU*s]	Height [mAU]	Area %
1	9.169	BB	0.2143	3444.34521	247.56381	49.9793
2	11.848	BB	0.3054	3447.19971	172.34232	50.0207
Totals :				6891.54492	419.90613	

Signal 3: DAD1 C, Sig=254,4 Ref=360,100

Peak #	RetTime [min]	Type	Width [min]	Area [mAU*s]	Height [mAU]	Area %
1	9.169	BB	0.2148	1094.60425	78.44391	50.0672
2	11.848	BB	0.3049	1091.66431	54.68898	49.9328
Totals :				2186.26855	133.13288	

Signal 4: DAD1 D, Sig=280,4 Ref=360,100

Signal 5: DAD1 E, Sig=310,4 Ref=360,100

*** End of Report ***

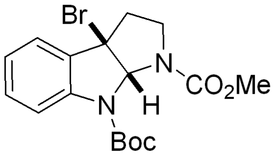

2d
(racemic)

Supplementary Figure 111. HPLC Spectrum of **2d** (racemic)

===

Acq. Operator   :   Seq. Line :   5
Acq. Instrument : Instrument 1   Location : Vial 4
Injection Date : 12/11/2021 9:23:39 PM   Inj :   1
Inj Volume : 5.000 µl
Different Inj Volume from Sequence !   Actual Inj Volume : 2.000 µl
Acq. Method   : C:\CHEM32\1\DATA\SUN_12 2021-12-11 20-22-58\IC-10-15.M
Last changed   : 12/11/2021 8:34:57 PM
(modified after loading)
Analysis Method : C:\CHEM32\1\METHODS\OD-03-60-0.6.M
Last changed   : 3/12/2022 7:32:45 PM
(modified after loading)
Additional Info : Peak(s) manually integrated

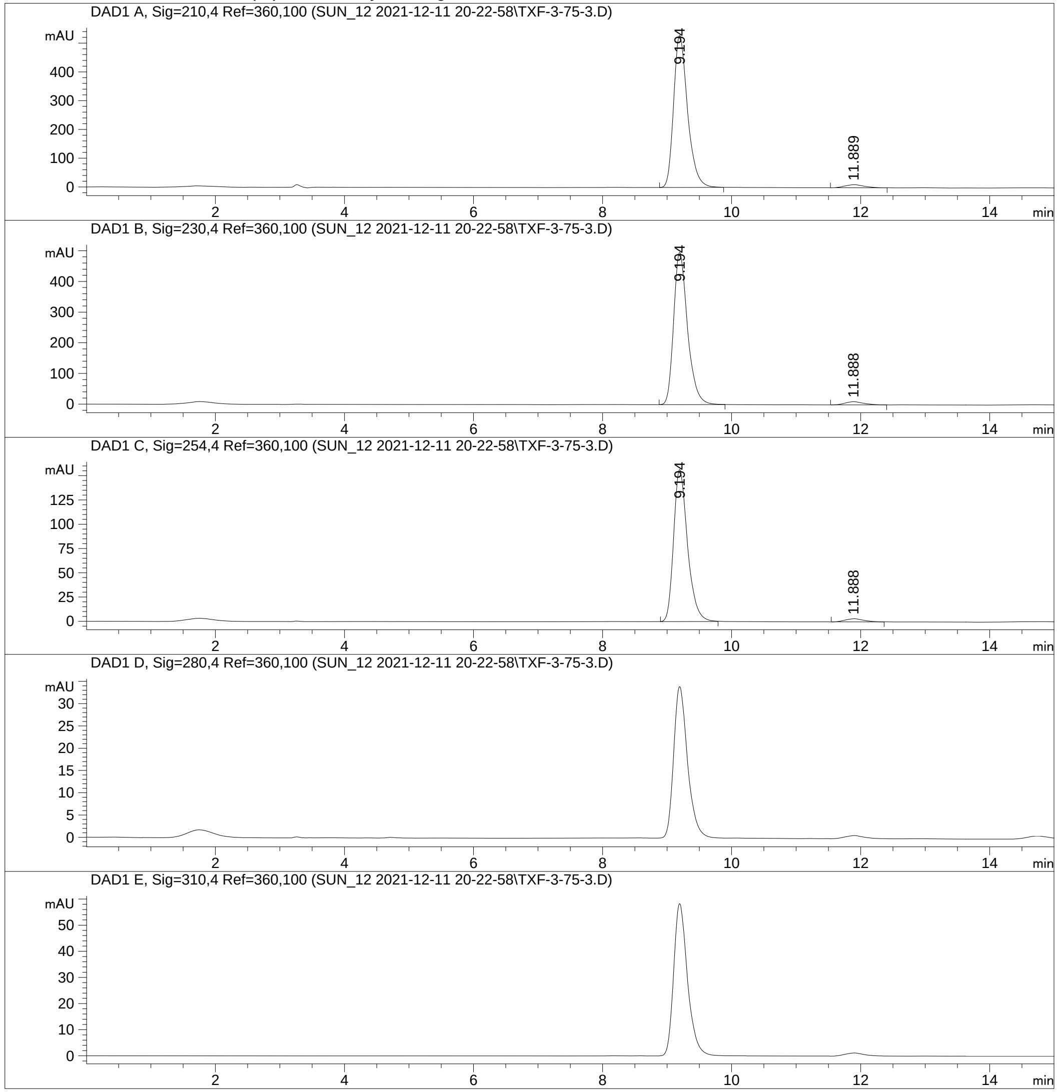

Data File C:\CHEM32\1\DATA\SUN_12 2021-12-11 20-22-58\TXF-3-75-3.D
Sample Name:

===

Area Percent Report

===

Sorted By   :   Signal
Multiplier   :   1.0000
Dilution   :   1.0000
Use Multiplier & Dilution Factor with ISTDs

Signal 1: DAD1 A, Sig=210,4 Ref=360,100

Peak #	RetTime [min]	Type	Width [min]	Area [mAU*s]	Height [mAU]	Area %
1	9.194	BB	0.2290	7927.85889	528.61560	97.2715
2	11.889	BB	0.3046	222.38321	11.05733	2.7285

Totals :   8150.24210 539.67293

Signal 2: DAD1 B, Sig=230,4 Ref=360,100

Peak #	RetTime [min]	Type	Width [min]	Area [mAU*s]	Height [mAU]	Area %
1	9.194	BB	0.2264	7416.90771	496.12534	97.3416
2	11.888	BB	0.3022	202.55492	10.26545	2.6584

Totals :   7619.46263 506.39078

Signal 3: DAD1 C, Sig=254,4 Ref=360,100

Peak #	RetTime [min]	Type	Width [min]	Area [mAU*s]	Height [mAU]	Area %
1	9.194	BB	0.2266	2344.88794	156.68584	97.3624
2	11.888	BB	0.2983	63.52337	3.24668	2.6376

Totals :   2408.41131 159.93251

Signal 4: DAD1 D, Sig=280,4 Ref=360,100

Signal 5: DAD1 E, Sig=310,4 Ref=360,100

===

*** End of Report ***

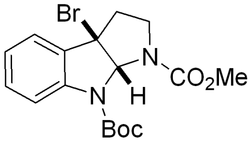

2d
(enantioenriched)

Supplementary Figure 112. HPLC Spectrum of **2d** (enantioenriched)

```
=====================================================================
Acq. Operator   :                               Seq. Line :  11
Acq. Instrument : Instrument 1                   Location : Vial 1
Injection Date  : 12/18/2021 10:22:40 PM              Inj :   1
                                               Inj Volume : 5.000 µl
Different Inj Volume from Sequence !     Actual Inj Volume : 2.000 µl
Acq. Method     : C:\CHEM32\1\DATA\SUN_12 2021-12-18 19-00-37\IC-04-20.M
Last changed    : 3/28/2021 9:30:05 AM
Analysis Method : C:\CHEM32\1\METHODS\OD-03-60-0.6.M
Last changed    : 3/12/2022 7:32:45 PM
                  (modified after loading)
Additional Info : Peak(s) manually integrated
```

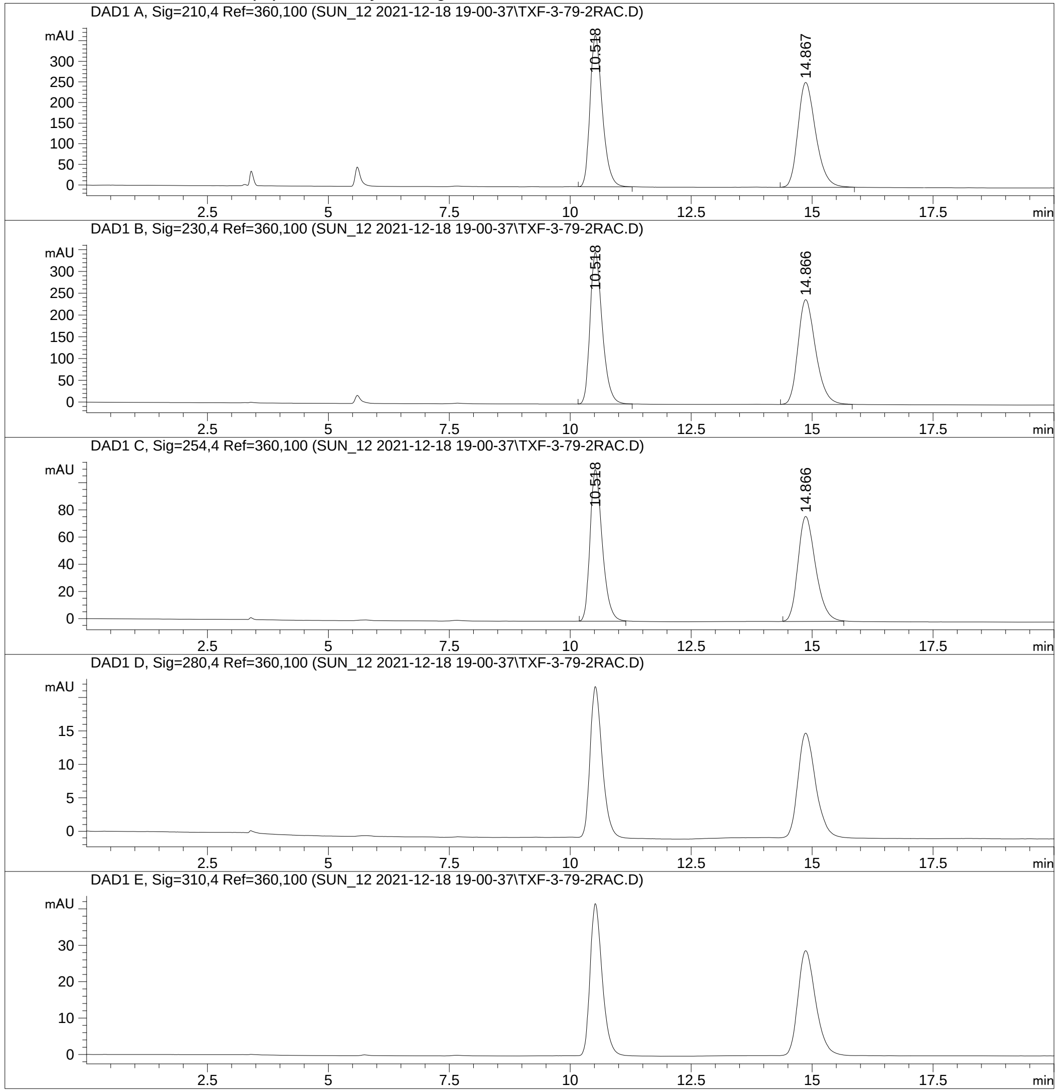

Data File C:\CHEM32\1\DATA\SUN_12 2021-12-18 19-00-37\TXF-3-79-2RAC.D
Sample Name:

Area Percent Report

Sorted By : Signal
Multiplier : 1.0000
Dilution : 1.0000
Use Multiplier & Dilution Factor with ISTDs

Signal 1: DAD1 A, Sig=210,4 Ref=360,100

Peak #	RetTime [min]	Type	Width [min]	Area [mAU*s]	Height [mAU]	Area %
1	10.518	BB	0.2687	6444.05371	367.29462	49.7537
2	14.867	BB	0.3923	6507.85352	254.49123	50.2463

Totals : 1.29519e4 621.78584

Signal 2: DAD1 B, Sig=230,4 Ref=360,100

Peak #	RetTime [min]	Type	Width [min]	Area [mAU*s]	Height [mAU]	Area %
1	10.518	BB	0.2686	6101.67529	348.00635	49.8678
2	14.866	BB	0.3915	6134.03760	240.55365	50.1322

Totals : 1.22357e4 588.56000

Signal 3: DAD1 C, Sig=254,4 Ref=360,100

Peak #	RetTime [min]	Type	Width [min]	Area [mAU*s]	Height [mAU]	Area %
1	10.518	BB	0.2686	1957.80261	111.62119	49.9092
2	14.866	BB	0.3907	1964.93018	77.25814	50.0908

Totals : 3922.73279 188.87933

Signal 4: DAD1 D, Sig=280,4 Ref=360,100

Signal 5: DAD1 E, Sig=310,4 Ref=360,100

*** End of Report ***

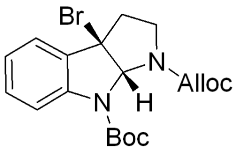

2e
(racemic)

Supplementary Figure 113. HPLC Spectrum of **2e** (racemic)

Data File C:\CHEM32\1\DATA\SUN_12 2021-12-18 19-00-37\TXF-3-79-3.D
Sample Name:

===

Acq. Operator : Seq. Line : 12
Acq. Instrument : Instrument 1 Location : Vial 2
Injection Date : 12/18/2021 10:43:36 PM Inj : 1
Inj Volume : 5.000 µl
Different Inj Volume from Sequence ! Actual Inj Volume : 2.000 µl
Acq. Method : C:\CHEM32\1\DATA\SUN_12 2021-12-18 19-00-37\IC-04-20.M
Last changed : 3/28/2021 9:30:05 AM
Analysis Method : C:\CHEM32\1\METHODS\OD-03-60-0.6.M
Last changed : 3/12/2022 7:32:45 PM
(modified after loading)
Additional Info : Peak(s) manually integrated

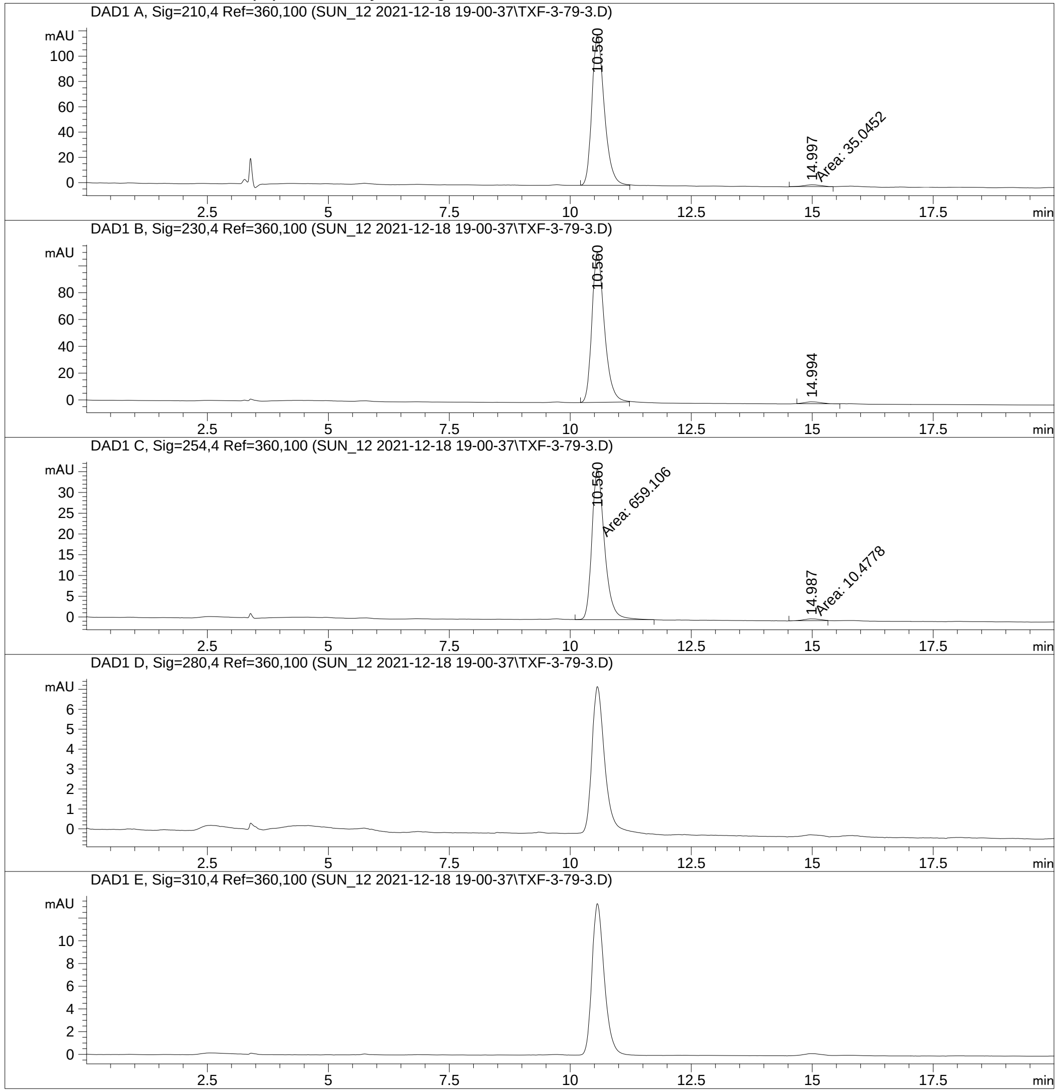

Data File C:\CHEM32\1\DATA\SUN_12 2021-12-18 19-00-37\TXF-3-79-3.D
Sample Name:

===

Area Percent Report

===

Sorted By : Signal
Multiplier : 1.0000
Dilution : 1.0000
Use Multiplier & Dilution Factor with ISTDs

Signal 1: DAD1 A, Sig=210,4 Ref=360,100

Peak #	RetTime [min]	Type	Width [min]	Area [mAU*s]	Height [mAU]	Area %
1	10.560	BB	0.2720	2112.49268	118.49528	98.3681
2	14.997	MM	0.4005	35.04523	1.45843	1.6319

Totals : 2147.53791 119.95370

Signal 2: DAD1 B, Sig=230,4 Ref=360,100

Peak #	RetTime [min]	Type	Width [min]	Area [mAU*s]	Height [mAU]	Area %
1	10.560	BB	0.2726	1998.59302	111.78140	98.4374
2	14.994	BB	0.2887	31.72669	1.37779	1.5626

Totals : 2030.31971 113.15920

Signal 3: DAD1 C, Sig=254,4 Ref=360,100

Peak #	RetTime [min]	Type	Width [min]	Area [mAU*s]	Height [mAU]	Area %
1	10.560	MM	0.3036	659.10614	36.17924	98.4352
2	14.987	MM	0.3848	10.47779	4.53875e-1	1.5648

Totals : 669.58393 36.63312

Signal 4: DAD1 D, Sig=280,4 Ref=360,100

Signal 5: DAD1 E, Sig=310,4 Ref=360,100

===

*** End of Report ***

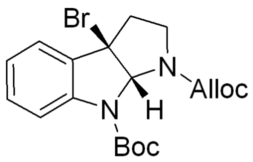

2e
(enantioenriched)

Supplementary Figure 114. HPLC Spectrum of **2e** (enantioenriched)

Data File C:\CHEM32\1\DATA\SUN_12 2021-12-23 22-38-16\TXF-3-89RAC.D
Sample Name:

===

Acq. Operator   :   Seq. Line :   9
Acq. Instrument : Instrument 1   Location : Vial 3
Injection Date : 12/24/2021 1:10:31 AM   Inj :   1
                                         Inj Volume : 5.000 µl
Different Inj Volume from Sequence !   Actual Inj Volume : 2.000 µl
Acq. Method   : C:\CHEM32\1\DATA\SUN_12 2021-12-23 22-38-16\IC-20-20.M
Last changed   : 12/24/2021 1:09:41 AM
                  (modified after loading)
Analysis Method : C:\CHEM32\1\METHODS\OD-03-60-0.6.M
Last changed   : 3/3/2022 9:54:51 PM
Additional Info : Peak(s) manually integrated

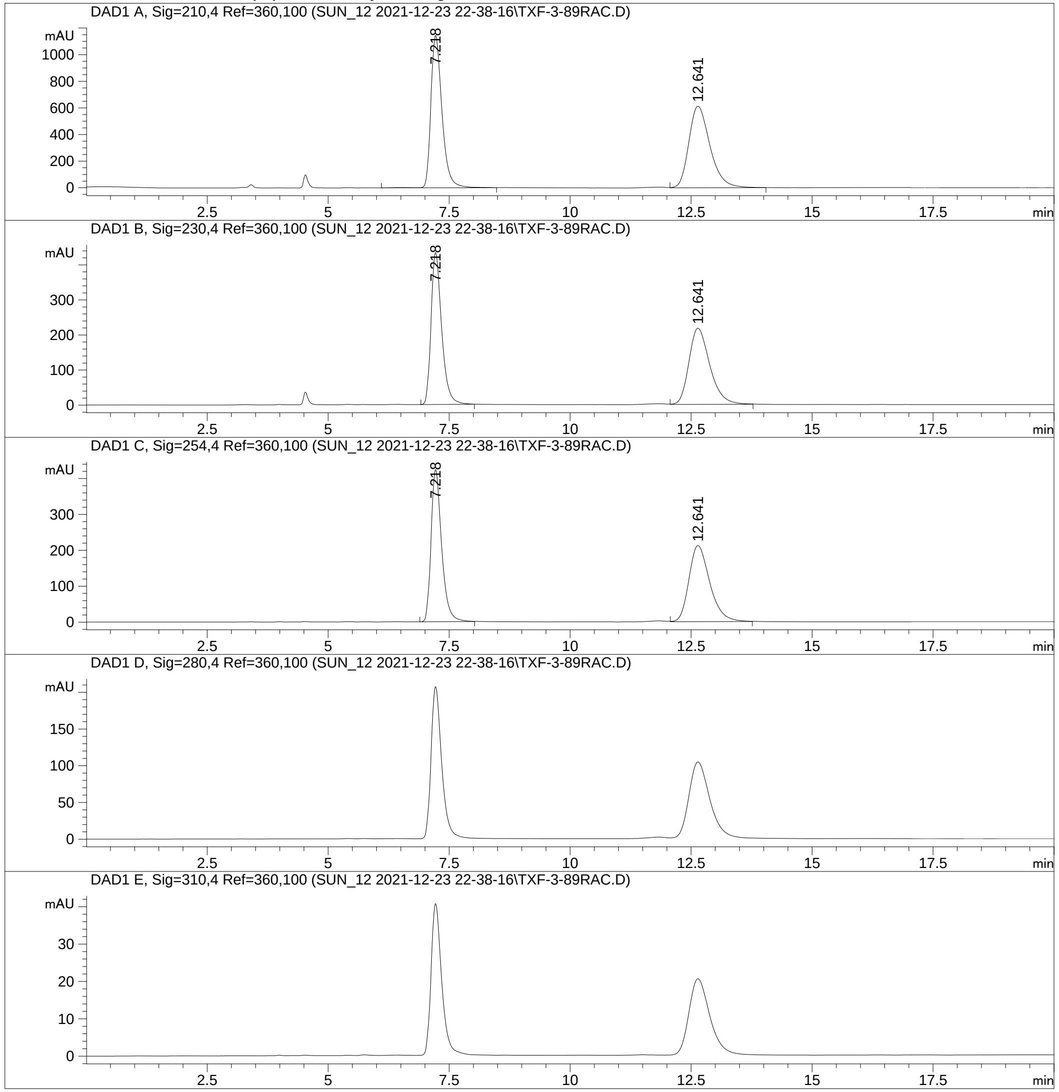

Data File C:\CHEM32\1\DATA\SUN_12 2021-12-23 22-38-16\TXF-3-89RAC.D
Sample Name:

===

Area Percent Report

===

Sorted By : Signal
Multiplier : 1.0000
Dilution : 1.0000
Use Multiplier & Dilution Factor with ISTDs

Signal 1: DAD1 A, Sig=210,4 Ref=360,100

Peak #	RetTime [min]	Type	Width [min]	Area [mAU*s]	Height [mAU]	Area %
1	7.218	BB	0.2451	1.83638e4	1144.98108	49.6111
2	12.641	VB	0.4649	1.86517e4	613.17487	50.3889

Totals : 3.70155e4 1758.15594

Signal 2: DAD1 B, Sig=230,4 Ref=360,100

Peak #	RetTime [min]	Type	Width [min]	Area [mAU*s]	Height [mAU]	Area %
1	7.218	BB	0.2315	6596.12012	433.50140	50.2698
2	12.641	VB	0.4602	6525.32568	217.36183	49.7302

Totals : 1.31214e4 650.86324

Signal 3: DAD1 C, Sig=254,4 Ref=360,100

Peak #	RetTime [min]	Type	Width [min]	Area [mAU*s]	Height [mAU]	Area %
1	7.218	BB	0.2288	6425.81836	424.10419	50.3053
2	12.641	VB	0.4573	6347.82715	212.01532	49.6947

Totals : 1.27736e4 636.11951

Signal 4: DAD1 D, Sig=280,4 Ref=360,100

Signal 5: DAD1 E, Sig=310,4 Ref=360,100

===

*** End of Report ***

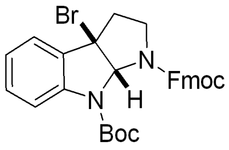

2f
(racemic)

Supplementary Figure 115. HPLC Spectrum of **2f** (racemic)

```
=====================================================================
Acq. Operator   :                                 Seq. Line :  10
Acq. Instrument : Instrument 1                     Location : Vial 4
Injection Date  : 12/24/2021 1:31:30 AM                 Inj :   1
                                                 Inj Volume : 5.000 µl
Different Inj Volume from Sequence !     Actual Inj Volume : 2.000 µl
Acq. Method     : C:\CHEM32\1\DATA\SUN_12 2021-12-23 22-38-16\IC-20-20.M
Last changed    : 12/24/2021 1:09:41 AM
                  (modified after loading)
Analysis Method : C:\CHEM32\1\METHODS\OD-03-60-0.6.M
Last changed    : 3/3/2022 9:54:51 PM
Additional Info : Peak(s) manually integrated
```

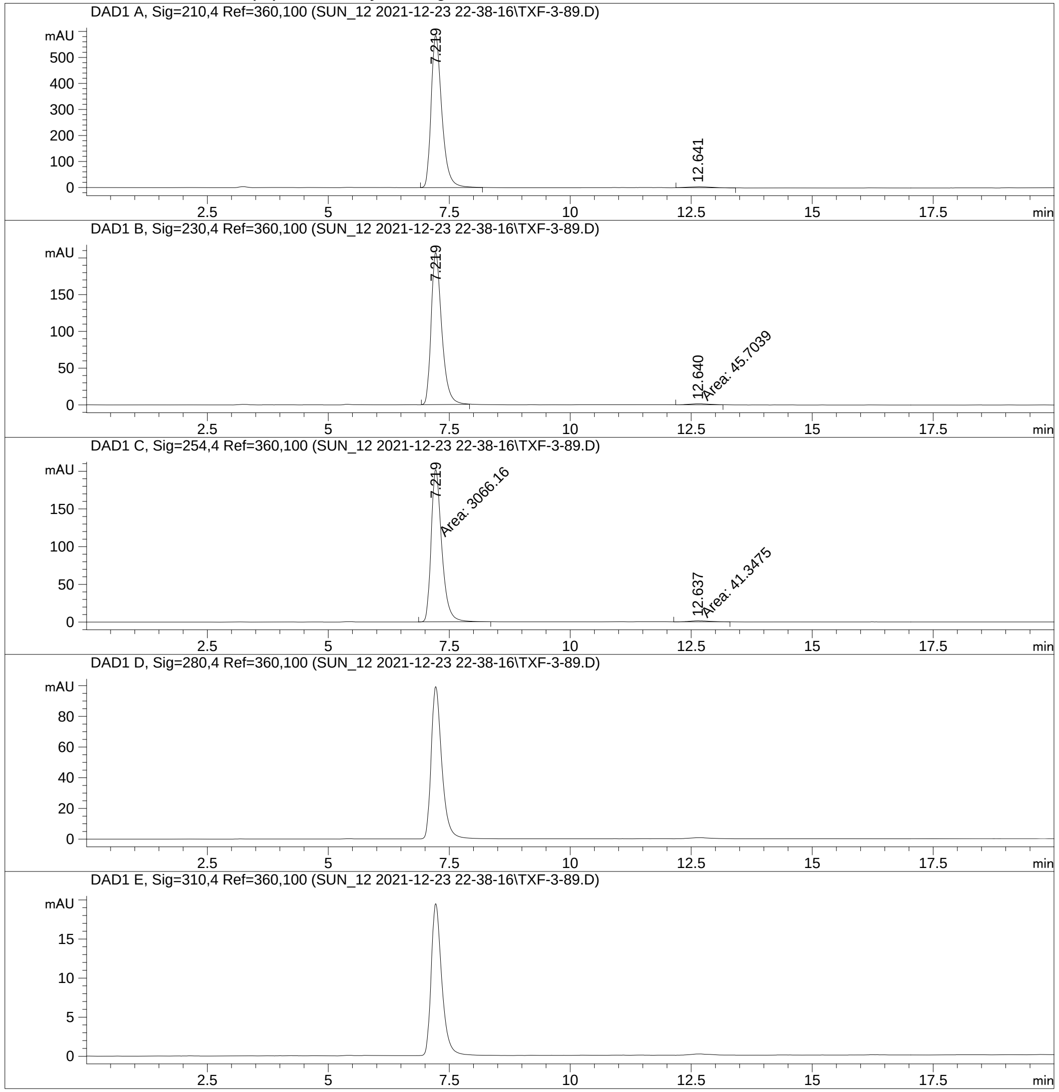

Data File C:\CHEM32\1\DATA\SUN_12 2021-12-23 22-38-16\TXF-3-89.D
Sample Name:

```
=====================================================================
                          Area Percent Report
=====================================================================

Sorted By             :      Signal
Multiplier            :      1.0000
Dilution              :      1.0000
Use Multiplier & Dilution Factor with ISTDs


Signal 1: DAD1 A, Sig=210,4 Ref=360,100

Peak RetTime Type  Width     Area      Height     Area
  #   [min]        [min]   [mAU*s]     [mAU]        %
----|-------|----|-------|----------|----------|--------|
   1   7.219 BB    0.2334 8992.04492  584.84991  98.5984
   2  12.641 BB    0.3853  127.82084    4.31907   1.4016

Totals :                  9119.86576  589.16898


Signal 2: DAD1 B, Sig=230,4 Ref=360,100

Peak RetTime Type  Width     Area      Height     Area
  #   [min]        [min]   [mAU*s]     [mAU]        %
----|-------|----|-------|----------|----------|--------|
   1   7.219 BB    0.2307 3134.69751  206.94528  98.5630
   2  12.640 MM    0.4934   45.70386    1.54374   1.4370

Totals :                  3180.40137  208.48902


Signal 3: DAD1 C, Sig=254,4 Ref=360,100

Peak RetTime Type  Width     Area      Height     Area
  #   [min]        [min]   [mAU*s]     [mAU]        %
----|-------|----|-------|----------|----------|--------|
   1   7.219 MM    0.2531 3066.15820  201.87172  98.6694
   2  12.637 MM    0.4855   41.34752    1.41946   1.3306

Totals :                  3107.50572  203.29118


Signal 4: DAD1 D, Sig=280,4 Ref=360,100


Signal 5: DAD1 E, Sig=310,4 Ref=360,100

=====================================================================
                          *** End of Report ***
```

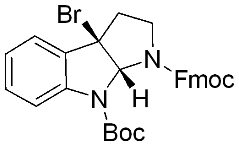

2f
(enantioenriched)

Supplementary Figure 116. HPLC Spectrum of **2f** (enantioenriched)

Data File C:\CHEM32\1\DATA\SUN_12 2022-01-21 15-02-39\TXF-3-133-2RAC.D
Sample Name:

===

Acq. Operator : Seq. Line : 5
Acq. Instrument : Instrument 1 Location : Vial 6
Injection Date : 1/21/2022 5:08:33 PM Inj : 1
Inj Volume : 5.000 µl
Different Inj Volume from Sequence ! Actual Inj Volume : 2.000 µl
Acq. Method : C:\CHEM32\1\DATA\SUN_12 2022-01-21 15-02-39\IC-01-20.M
Last changed : 1/21/2022 5:07:42 PM
(modified after loading)
Analysis Method : C:\CHEM32\1\METHODS\OD-03-60-0.6.M
Last changed : 3/3/2022 9:54:51 PM
Additional Info : Peak(s) manually integrated

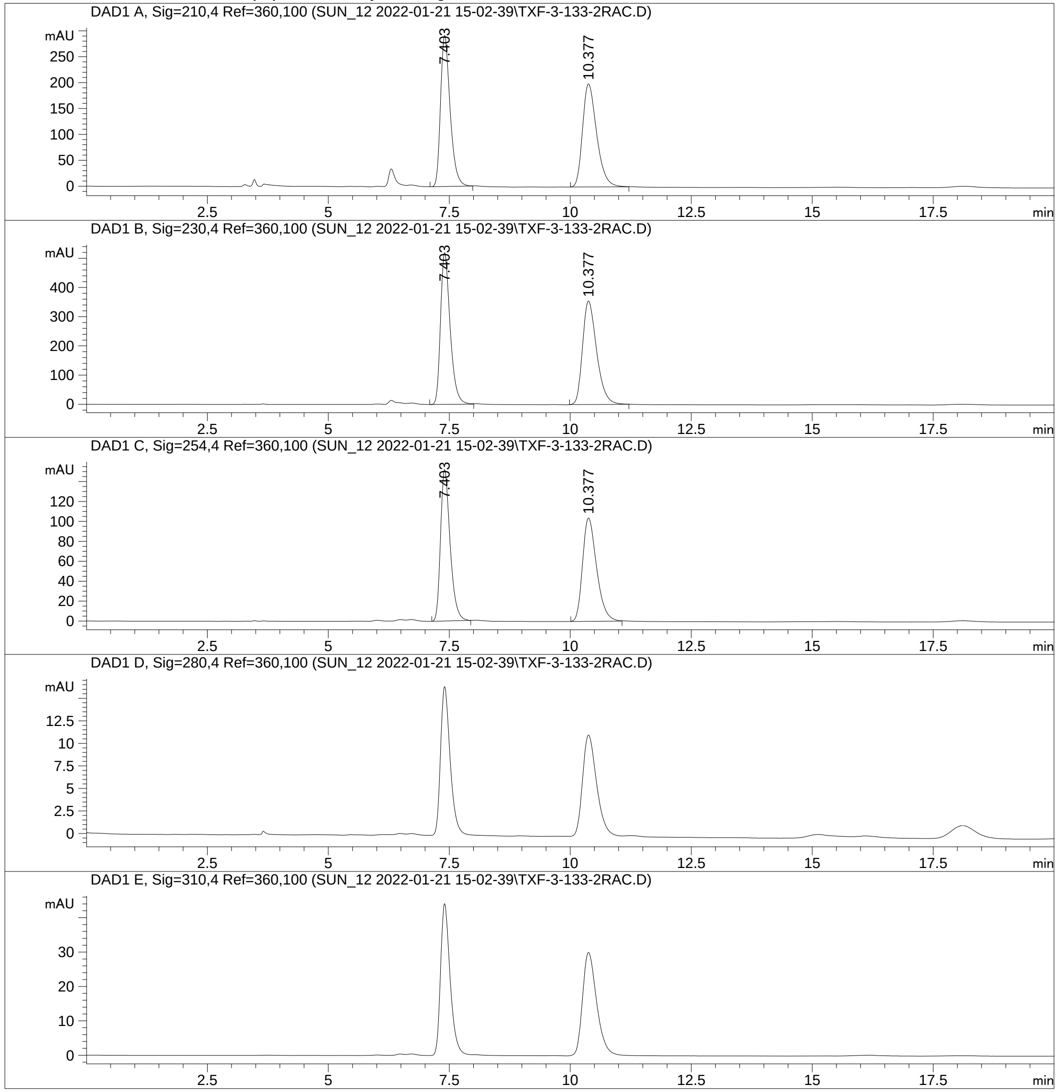

Data File C:\CHEM32\1\DATA\SUN_12 2022-01-21 15-02-39\TXF-3-133-2RAC.D
Sample Name:

===

Area Percent Report

===

Sorted By : Signal
Multiplier : 1.0000
Dilution : 1.0000
Use Multiplier & Dilution Factor with ISTDs

Signal 1: DAD1 A, Sig=210,4 Ref=360,100

Peak #	RetTime [min]	Type	Width [min]	Area [mAU*s]	Height [mAU]	Area %
1	7.403	VB	0.2106	4017.60376	291.83264	49.8582
2	10.377	BB	0.3106	4040.46045	199.26074	50.1418
Totals :				8058.06421	491.09338	

Signal 2: DAD1 B, Sig=230,4 Ref=360,100

Peak #	RetTime [min]	Type	Width [min]	Area [mAU*s]	Height [mAU]	Area %
1	7.403	VB	0.2089	7175.13232	520.17639	49.9571
2	10.377	BB	0.3103	7187.44336	354.91550	50.0429
Totals :				1.43626e4	875.09189	

Signal 3: DAD1 C, Sig=254,4 Ref=360,100

Peak #	RetTime [min]	Type	Width [min]	Area [mAU*s]	Height [mAU]	Area %
1	7.403	BB	0.2079	2083.80859	152.00900	49.9043
2	10.377	BB	0.3092	2091.79834	103.75228	50.0957
Totals :				4175.60693	255.76128	

Signal 4: DAD1 D, Sig=280,4 Ref=360,100

Signal 5: DAD1 E, Sig=310,4 Ref=360,100

===

*** End of Report ***

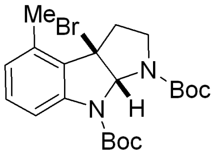

2g
(racemic)

Supplementary Figure 117. HPLC Spectrum of **2g** (racemic)

===

Acq. Operator : Seq. Line : 6
Acq. Instrument : Instrument 1 Location : Vial 7
Injection Date : 1/21/2022 5:29:32 PM Inj : 1
Inj Volume : 5.000 µl
Different Inj Volume from Sequence ! Actual Inj Volume : 2.000 µl
Acq. Method : C:\CHEM32\1\DATA\SUN_12 2022-01-21 15-02-39\IC-01-20.M
Last changed : 1/21/2022 5:07:42 PM
(modified after loading)
Analysis Method : C:\CHEM32\1\METHODS\OD-03-60-0.6.M
Last changed : 3/3/2022 9:54:51 PM
Additional Info : Peak(s) manually integrated

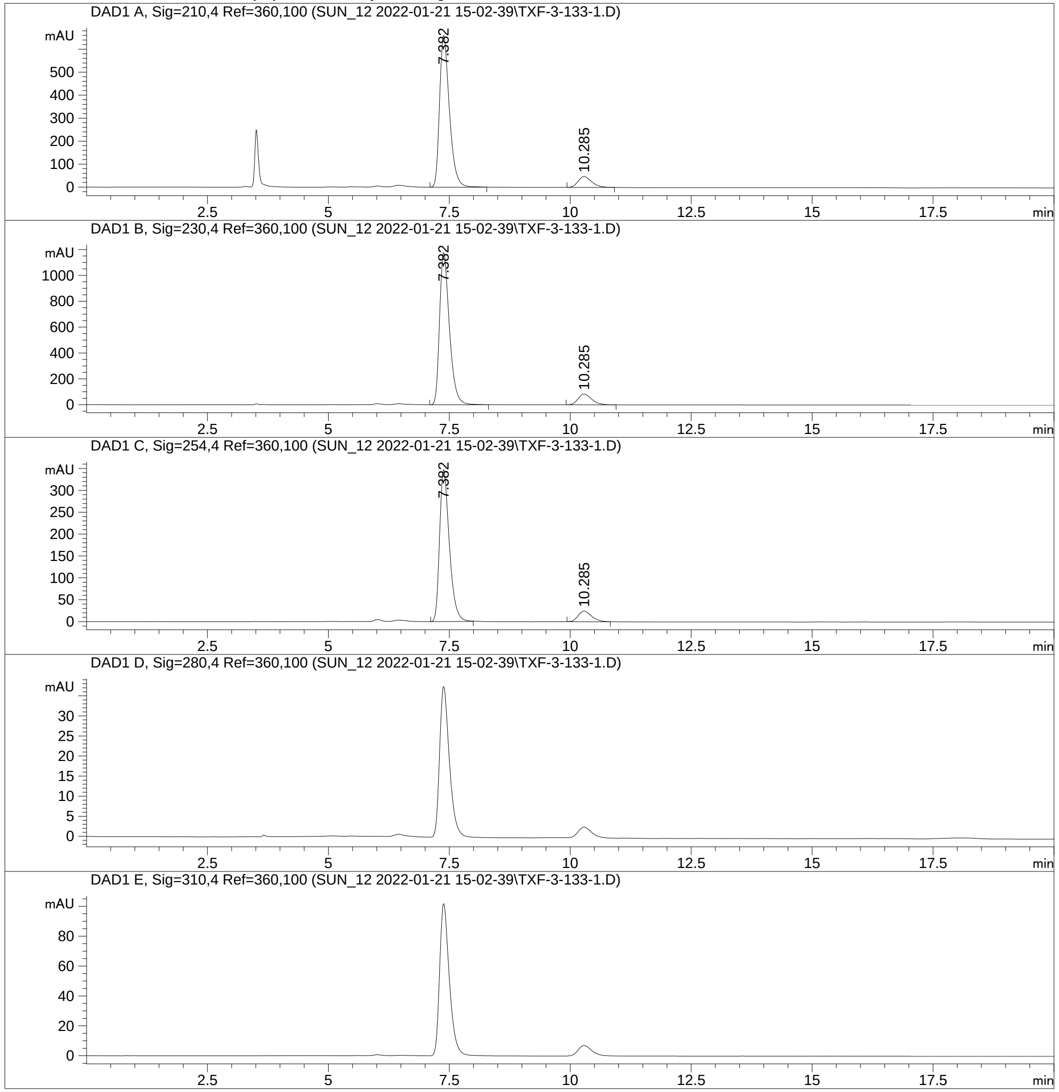

Data File C:\CHEM32\1\DATA\SUN_12 2022-01-21 15-02-39\TXF-3-133-1.D
Sample Name:

===

Area Percent Report

===

Sorted By : Signal
Multiplier : 1.0000
Dilution : 1.0000
Use Multiplier & Dilution Factor with ISTDs

Signal 1: DAD1 A, Sig=210,4 Ref=360,100

Peak #	RetTime [min]	Type	Width [min]	Area [mAU*s]	Height [mAU]	Area %
1	7.382	BB	0.2147	9324.76270	660.37994	90.9122
2	10.285	BB	0.3014	932.12067	47.39770	9.0878

Totals : 1.02569e4 707.77765

Signal 2: DAD1 B, Sig=230,4 Ref=360,100

Peak #	RetTime [min]	Type	Width [min]	Area [mAU*s]	Height [mAU]	Area %
1	7.382	BB	0.2146	1.66084e4	1177.36926	91.0250
2	10.285	BB	0.3007	1637.56885	83.53437	8.9750

Totals : 1.82459e4 1260.90363

Signal 3: DAD1 C, Sig=254,4 Ref=360,100

Peak #	RetTime [min]	Type	Width [min]	Area [mAU*s]	Height [mAU]	Area %
1	7.382	BB	0.2130	4852.35840	347.37186	91.0569
2	10.285	BB	0.2996	476.56979	24.43163	8.9431

Totals : 5328.92819 371.80348

Signal 4: DAD1 D, Sig=280,4 Ref=360,100

Signal 5: DAD1 E, Sig=310,4 Ref=360,100

===

*** End of Report ***

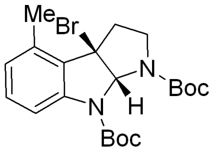

2g
(enantioenriched)

Supplementary Figure 118. HPLC Spectrum of **2g** (enantioenriched)

===

Acq. Operator : Seq. Line : 15
Acq. Instrument : Instrument 1 Location : Vial 5
Injection Date : 12/19/2021 12:06:34 AM Inj : 1
Inj Volume : 5.000 µl
Different Inj Volume from Sequence ! Actual Inj Volume : 2.000 µl
Acq. Method : C:\CHEM32\1\DATA\SUN_12 2021-12-18 19-00-37\IC-04-20.M
Last changed : 3/28/2021 9:30:05 AM
Analysis Method : C:\CHEM32\1\METHODS\OD-03-60-0.6.M
Last changed : 3/12/2022 7:32:45 PM
(modified after loading)
Additional Info : Peak(s) manually integrated

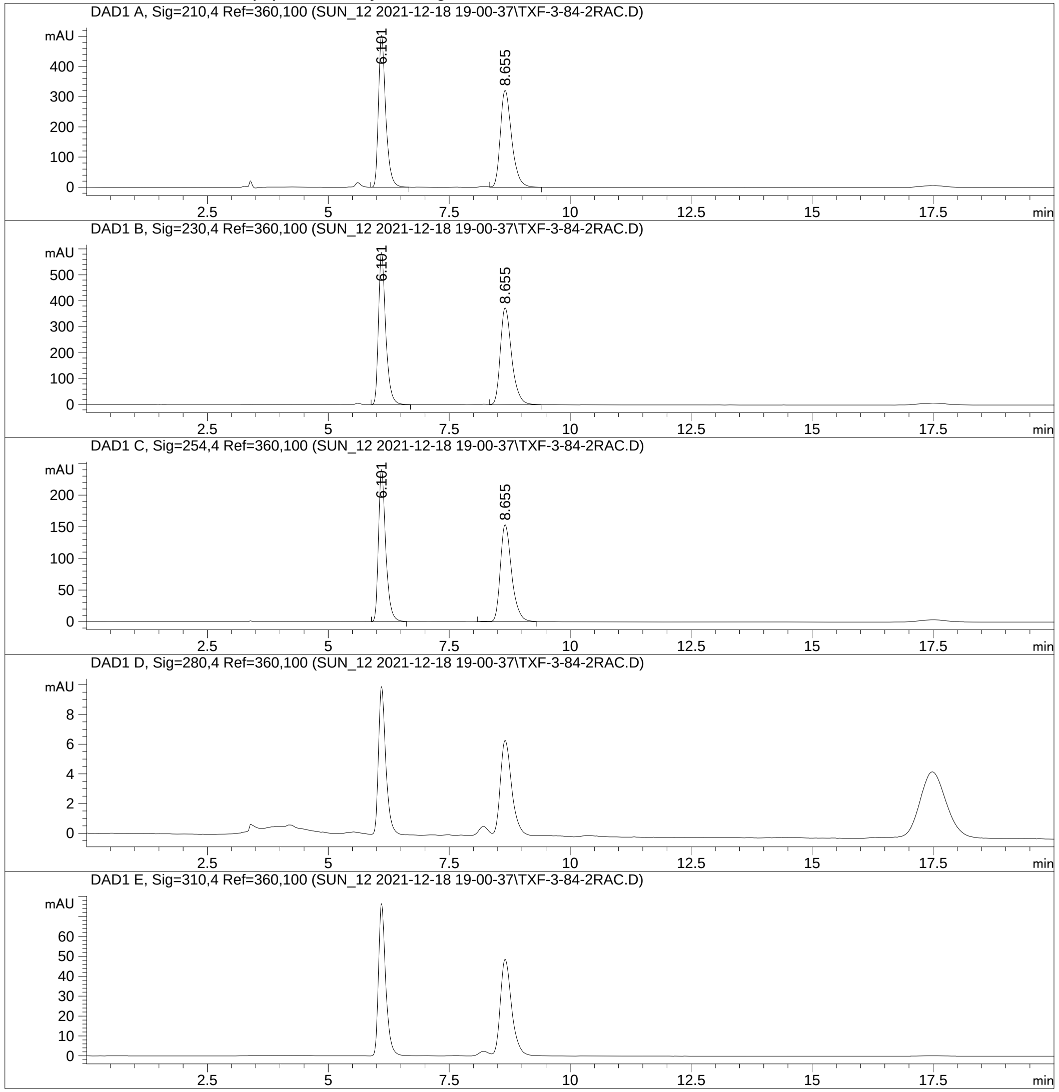

Data File C:\CHEM32\1\DATA\SUN_12 2021-12-18 19-00-37\TXF-3-84-2RAC.D
Sample Name:

===

Area Percent Report

===

Sorted By : Signal
Multiplier : 1.0000
Dilution : 1.0000
Use Multiplier & Dilution Factor with ISTDs

Signal 1: DAD1 A, Sig=210,4 Ref=360,100

Peak #	RetTime [min]	Type	Width [min]	Area [mAU*s]	Height [mAU]	Area %
1	6.101	VB	0.1574	5244.64160	503.86960	49.9467
2	8.655	VB	0.2504	5255.83203	322.04965	50.0533

Totals : 1.05005e4 825.91925

Signal 2: DAD1 B, Sig=230,4 Ref=360,100

Peak #	RetTime [min]	Type	Width [min]	Area [mAU*s]	Height [mAU]	Area %
1	6.101	BB	0.1571	6089.20313	586.49963	50.0669
2	8.655	VB	0.2499	6072.92920	373.09009	49.9331

Totals : 1.21621e4 959.58972

Signal 3: DAD1 C, Sig=254,4 Ref=360,100

Peak #	RetTime [min]	Type	Width [min]	Area [mAU*s]	Height [mAU]	Area %
1	6.101	BB	0.1571	2497.16431	240.55075	49.9877
2	8.655	BB	0.2483	2498.39478	153.15919	50.0123

Totals : 4995.55908 393.70995

Signal 4: DAD1 D, Sig=280,4 Ref=360,100

Signal 5: DAD1 E, Sig=310,4 Ref=360,100

===

*** End of Report ***

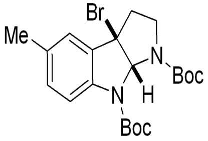

2h
(racemic)

Supplementary Figure 119. HPLC Spectrum of **2h** (racemic)

Data File C:\CHEM32\1\DATA\SUN_12 2021-12-18 19-00-37\TXF-3-84-3.D
Sample Name:

===

Acq. Operator   :   Seq. Line : 16
Acq. Instrument : Instrument 1   Location : Vial 6
Injection Date : 12/19/2021 12:27:31 AM   Inj :   1
                                              Inj Volume : 5.000 µl
Different Inj Volume from Sequence !   Actual Inj Volume : 2.000 µl
Acq. Method   : C:\CHEM32\1\DATA\SUN_12 2021-12-18 19-00-37\IC-04-20.M
Last changed   : 3/28/2021 9:30:05 AM
Analysis Method : C:\CHEM32\1\METHODS\OD-03-60-0.6.M
Last changed   : 3/12/2022 7:32:45 PM
                  (modified after loading)
Additional Info : Peak(s) manually integrated

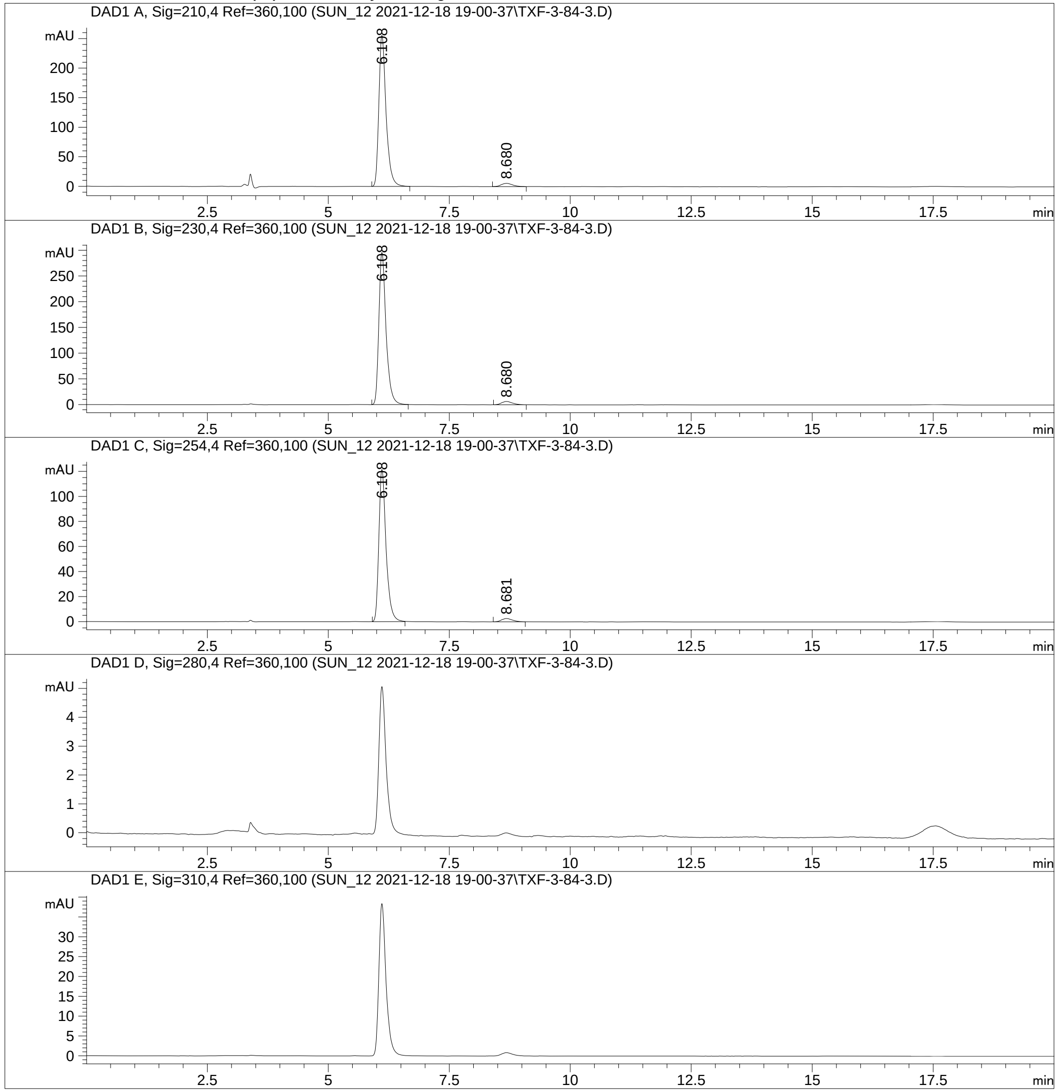

Data File C:\CHEM32\1\DATA\SUN_12 2021-12-18 19-00-37\TXF-3-84-3.D
Sample Name:

===

Area Percent Report

===

Sorted By   :   Signal
Multiplier   :   1.0000
Dilution   :   1.0000
Use Multiplier & Dilution Factor with ISTDs

Signal 1: DAD1 A, Sig=210,4 Ref=360,100

Peak #	RetTime [min]	Type	Width [min]	Area [mAU*s]	Height [mAU]	Area %
1	6.108	BB	0.1571	2654.14111	255.75586	96.7463
2	8.680	BB	0.2406	89.26092	5.64184	3.2537

Totals :   2743.40203   261.39769

Signal 2: DAD1 B, Sig=230,4 Ref=360,100

Peak #	RetTime [min]	Type	Width [min]	Area [mAU*s]	Height [mAU]	Area %
1	6.108	BB	0.1567	3062.84937	296.03235	96.7211
2	8.680	BB	0.2442	103.83143	6.50719	3.2789

Totals :   3166.68079   302.53954

Signal 3: DAD1 C, Sig=254,4 Ref=360,100

Peak #	RetTime [min]	Type	Width [min]	Area [mAU*s]	Height [mAU]	Area %
1	6.108	BB	0.1567	1258.00879	121.61863	96.7438
2	8.681	BB	0.2408	42.34211	2.67250	3.2562

Totals :   1300.35090   124.29113

Signal 4: DAD1 D, Sig=280,4 Ref=360,100

Signal 5: DAD1 E, Sig=310,4 Ref=360,100

===

*** End of Report ***

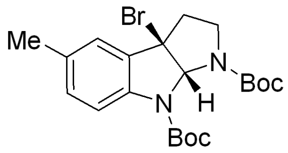

2h
(enantioenriched)

Supplementary Figure 120. HPLC Spectrum of **2h** (enantioenriched)

Data File C:\CHEM32\1\DATA\SUN_12 2021-12-18 19-00-37\TXF-3-80-2RAC.D
Sample Name:

===

Acq. Operator : Seq. Line : 13
Acq. Instrument : Instrument 1 Location : Vial 3
Injection Date : 12/18/2021 11:04:34 PM Inj : 1
Inj Volume : 5.000 µl
Different Inj Volume from Sequence ! Actual Inj Volume : 2.000 µl
Acq. Method : C:\CHEM32\1\DATA\SUN_12 2021-12-18 19-00-37\IC-04-30.M
Last changed : 12/18/2021 11:03:45 PM
(modified after loading)
Analysis Method : C:\CHEM32\1\METHODS\OD-03-60-0.6.M
Last changed : 3/12/2022 7:32:45 PM
(modified after loading)
Additional Info : Peak(s) manually integrated

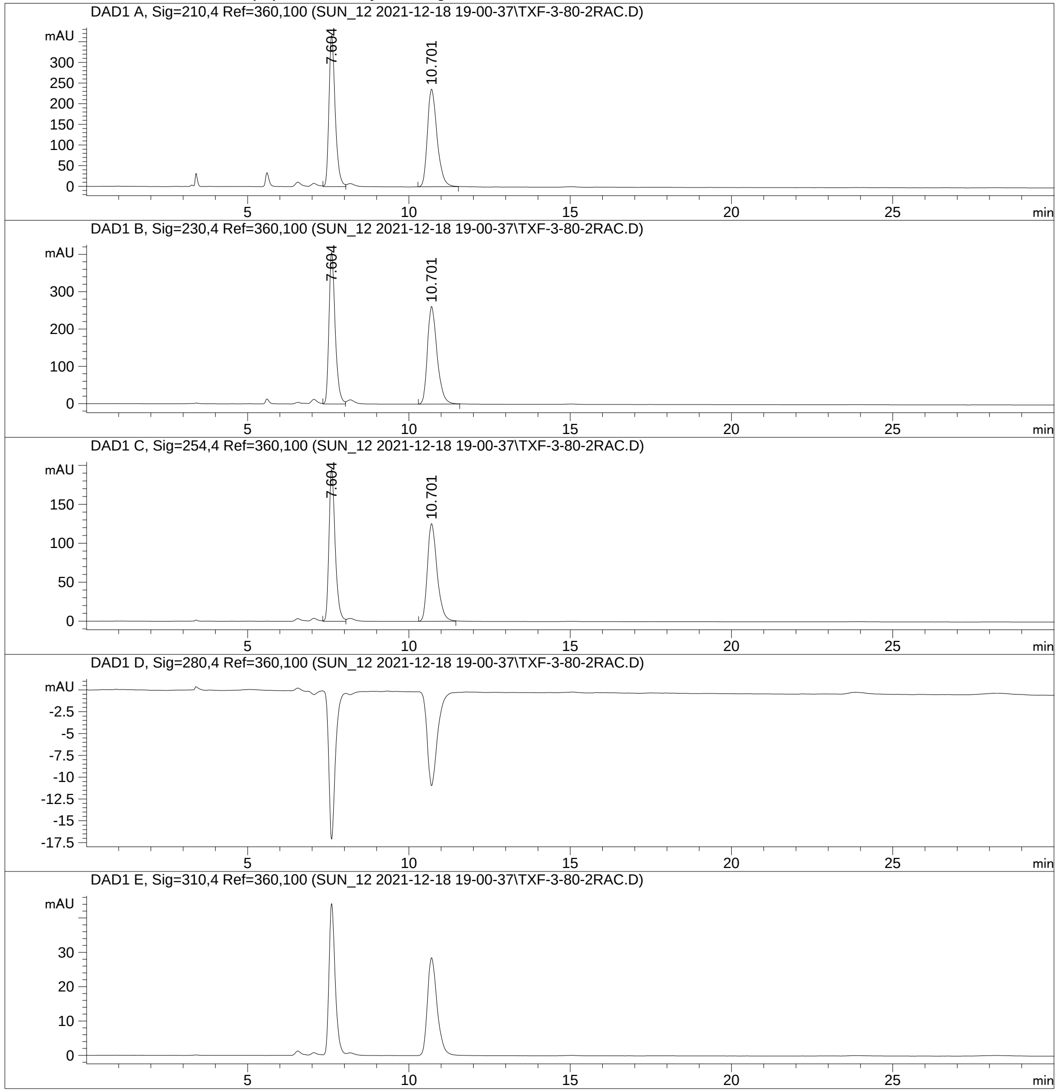

Data File C:\CHEM32\1\DATA\SUN_12 2021-12-18 19-00-37\TXF-3-80-2RAC.D
Sample Name:

===

Area Percent Report

===

Sorted By : Signal
Multiplier : 1.0000
Dilution : 1.0000
Use Multiplier & Dilution Factor with ISTDs

Signal 1: DAD1 A, Sig=210,4 Ref=360,100

Peak #	RetTime [min]	Type	Width [min]	Area [mAU*s]	Height [mAU]	Area %
1	7.604	VV	0.2043	4906.27441	366.32013	49.7945
2	10.701	BB	0.3199	4946.77588	236.59332	50.2055

Totals : 9853.05029 602.91345

Signal 2: DAD1 B, Sig=230,4 Ref=360,100

Peak #	RetTime [min]	Type	Width [min]	Area [mAU*s]	Height [mAU]	Area %
1	7.604	VV	0.2036	5416.02197	406.12543	49.8085
2	10.701	BB	0.3194	5457.66748	261.60910	50.1915

Totals : 1.08737e4 667.73453

Signal 3: DAD1 C, Sig=254,4 Ref=360,100

Peak #	RetTime [min]	Type	Width [min]	Area [mAU*s]	Height [mAU]	Area %
1	7.604	VV	0.2037	2600.86060	194.90192	49.9339
2	10.701	BB	0.3167	2607.74463	125.37426	50.0661

Totals : 5208.60522 320.27618

Signal 4: DAD1 D, Sig=280,4 Ref=360,100

Signal 5: DAD1 E, Sig=310,4 Ref=360,100

===

*** End of Report ***

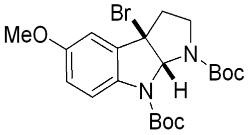

2i
(racemic)

Supplementary Figure 121. HPLC Spectrum of **2i** (racemic)

```
=====================================================================
Acq. Operator   :                               Seq. Line :  14
Acq. Instrument : Instrument 1                   Location : Vial 4
Injection Date  : 12/18/2021 11:35:33 PM              Inj :   1
                                               Inj Volume : 5.000 µl
Different Inj Volume from Sequence !     Actual Inj Volume : 2.000 µl
Acq. Method     : C:\CHEM32\1\DATA\SUN_12 2021-12-18 19-00-37\IC-04-30.M
Last changed    : 12/18/2021 11:03:45 PM
                  (modified after loading)
Analysis Method : C:\CHEM32\1\METHODS\OD-03-60-0.6.M
Last changed    : 3/12/2022 7:32:45 PM
                  (modified after loading)
Additional Info : Peak(s) manually integrated
```

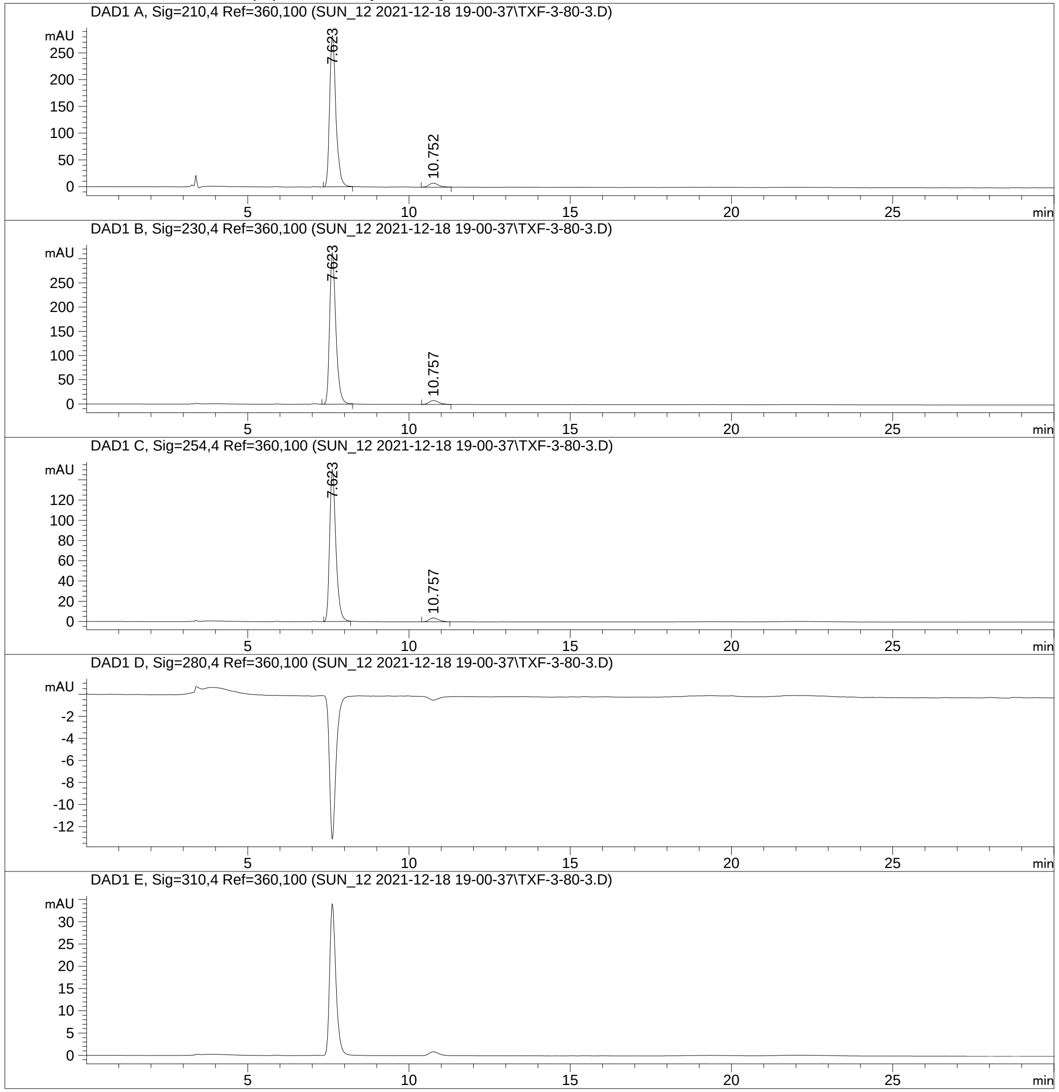

Area Percent Report

```
Sorted By            :      Signal
Multiplier           :      1.0000
Dilution             :      1.0000
Use Multiplier & Dilution Factor with ISTDs
```

Signal 1: DAD1 A, Sig=210,4 Ref=360,100

Peak #	RetTime [min]	Type	Width [min]	Area [mAU*s]	Height [mAU]	Area %
1	7.623	BB	0.2033	3818.73120	283.23096	96.1330
2	10.752	BB	0.3067	153.60873	7.50886	3.8670

Totals : 3972.33994 290.73982

Signal 2: DAD1 B, Sig=230,4 Ref=360,100

Peak #	RetTime [min]	Type	Width [min]	Area [mAU*s]	Height [mAU]	Area %
1	7.623	VB	0.2029	4211.64453	313.18240	96.1896
2	10.757	BB	0.3169	166.83781	8.08065	3.8104

Totals : 4378.48235 321.26305

Signal 3: DAD1 C, Sig=254,4 Ref=360,100

Peak #	RetTime [min]	Type	Width [min]	Area [mAU*s]	Height [mAU]	Area %
1	7.623	BB	0.2026	2015.97559	150.23587	96.2042
2	10.757	BB	0.3158	79.54237	3.86970	3.7958

Totals : 2095.51796 154.10557

Signal 4: DAD1 D, Sig=280,4 Ref=360,100

Signal 5: DAD1 E, Sig=310,4 Ref=360,100

*** End of Report ***

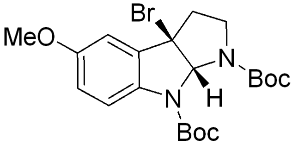

2i
(enantioenriched)

Supplementary Figure 122. HPLC Spectrum of **2i** (enantioenriched)

```
=====================================================================
Acq. Operator   :                             Seq. Line :   2
Acq. Instrument : Instrument 1                 Location : Vial 8
Injection Date  : 1/7/2022 11:15:53 AM              Inj :   1
                                             Inj Volume : 5.000 µl
Different Inj Volume from Sequence !     Actual Inj Volume : 2.000 µl
Acq. Method     : C:\CHEM32\1\DATA\SUN_12 2022-01-07 11-03-06\IC-04-20.M
Last changed    : 3/28/2021 9:30:05 AM
Analysis Method : C:\CHEM32\1\METHODS\OD-03-60-0.6.M
Last changed    : 3/3/2022 9:54:51 PM
Additional Info : Peak(s) manually integrated
```

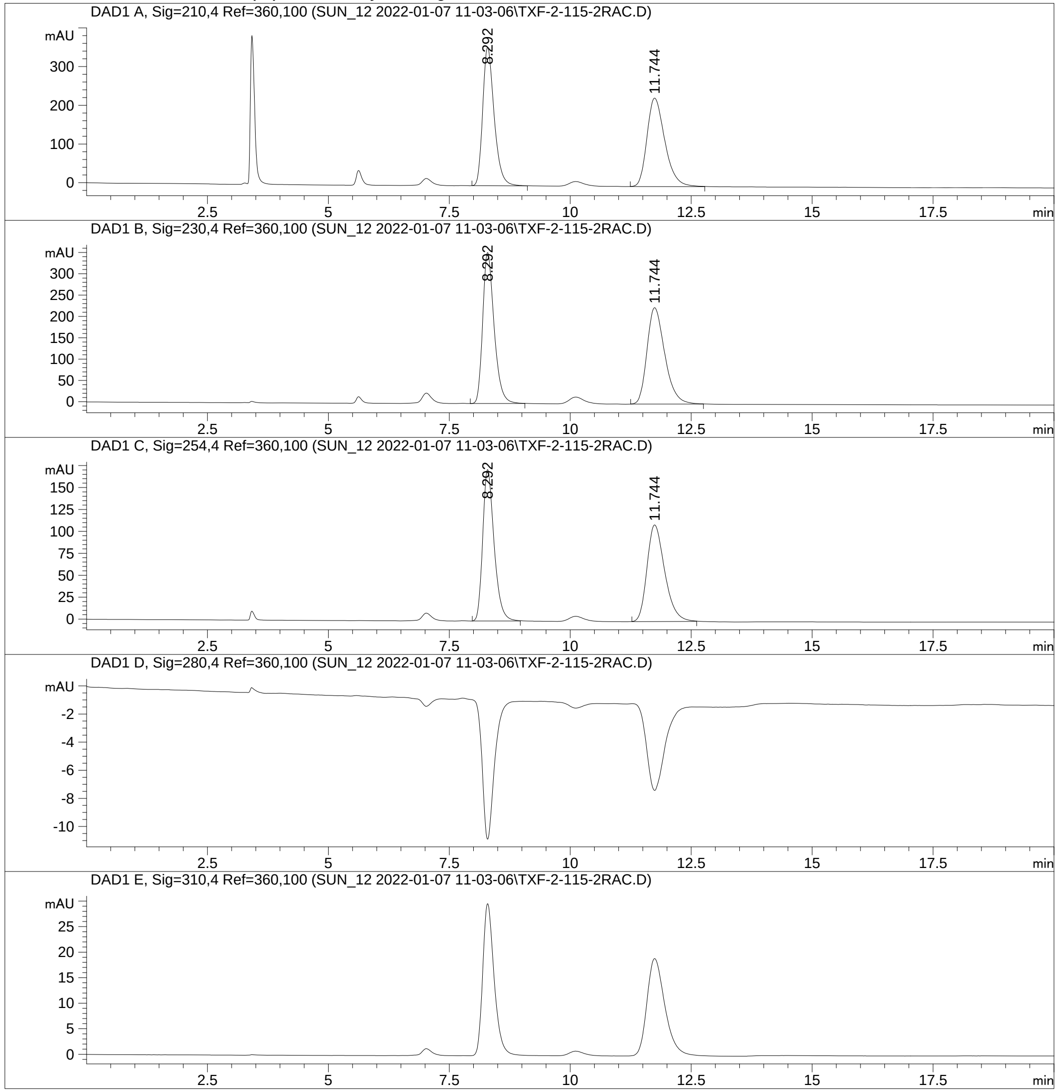

Data File C:\CHEM32\1\DATA\SUN_12 2022-01-07 11-03-06\TXF-2-115-2RAC.D
Sample Name:

```
=====================================================================
                         Area Percent Report
=====================================================================

Sorted By             :      Signal
Multiplier            :      1.0000
Dilution              :      1.0000
Use Multiplier & Dilution Factor with ISTDs
```

Signal 1: DAD1 A, Sig=210,4 Ref=360,100

Peak #	RetTime [min]	Type	Width [min]	Area [mAU*s]	Height [mAU]	Area %
1	8.292	BB	0.2519	5872.99658	357.14413	50.0131
2	11.744	BB	0.3936	5869.91650	228.60013	49.9869

Totals : 1.17429e4 585.74426

Signal 2: DAD1 B, Sig=230,4 Ref=360,100

Peak #	RetTime [min]	Type	Width [min]	Area [mAU*s]	Height [mAU]	Area %
1	8.292	VB	0.2511	5797.79541	354.00287	50.0533
2	11.744	BB	0.3927	5785.45215	225.96706	49.9467

Totals : 1.15832e4 579.96992

Signal 3: DAD1 C, Sig=254,4 Ref=360,100

Peak #	RetTime [min]	Type	Width [min]	Area [mAU*s]	Height [mAU]	Area %
1	8.292	BB	0.2506	2818.72461	172.57149	50.0700
2	11.744	BB	0.3915	2810.84229	110.21164	49.9300

Totals : 5629.56689 282.78313

Signal 4: DAD1 D, Sig=280,4 Ref=360,100

Signal 5: DAD1 E, Sig=310,4 Ref=360,100

```
=====================================================================
                        *** End of Report ***
```

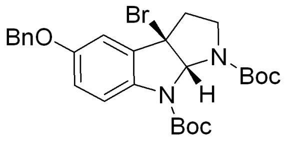

2j
(racemic)

Supplementary Figure 123. HPLC Spectrum of **2j** (racemic)

Data File C:\CHEM32\1\DATA\SUN_12 2022-01-07 11-03-06\TXF-2-115-1.D
Sample Name:

```
=====================================================================
Acq. Operator   :                        Seq. Line :   3
Acq. Instrument : Instrument 1            Location : Vial 9
Injection Date  : 1/7/2022 11:36:53 AM         Inj :   1
                                        Inj Volume : 5.000 µl
Different Inj Volume from Sequence !     Actual Inj Volume : 2.000 µl
Acq. Method     : C:\CHEM32\1\DATA\SUN_12 2022-01-07 11-03-06\IC-04-20.M
Last changed    : 3/28/2021 9:30:05 AM
Analysis Method : C:\CHEM32\1\METHODS\OD-03-60-0.6.M
Last changed    : 3/3/2022 9:54:51 PM
Additional Info : Peak(s) manually integrated
```

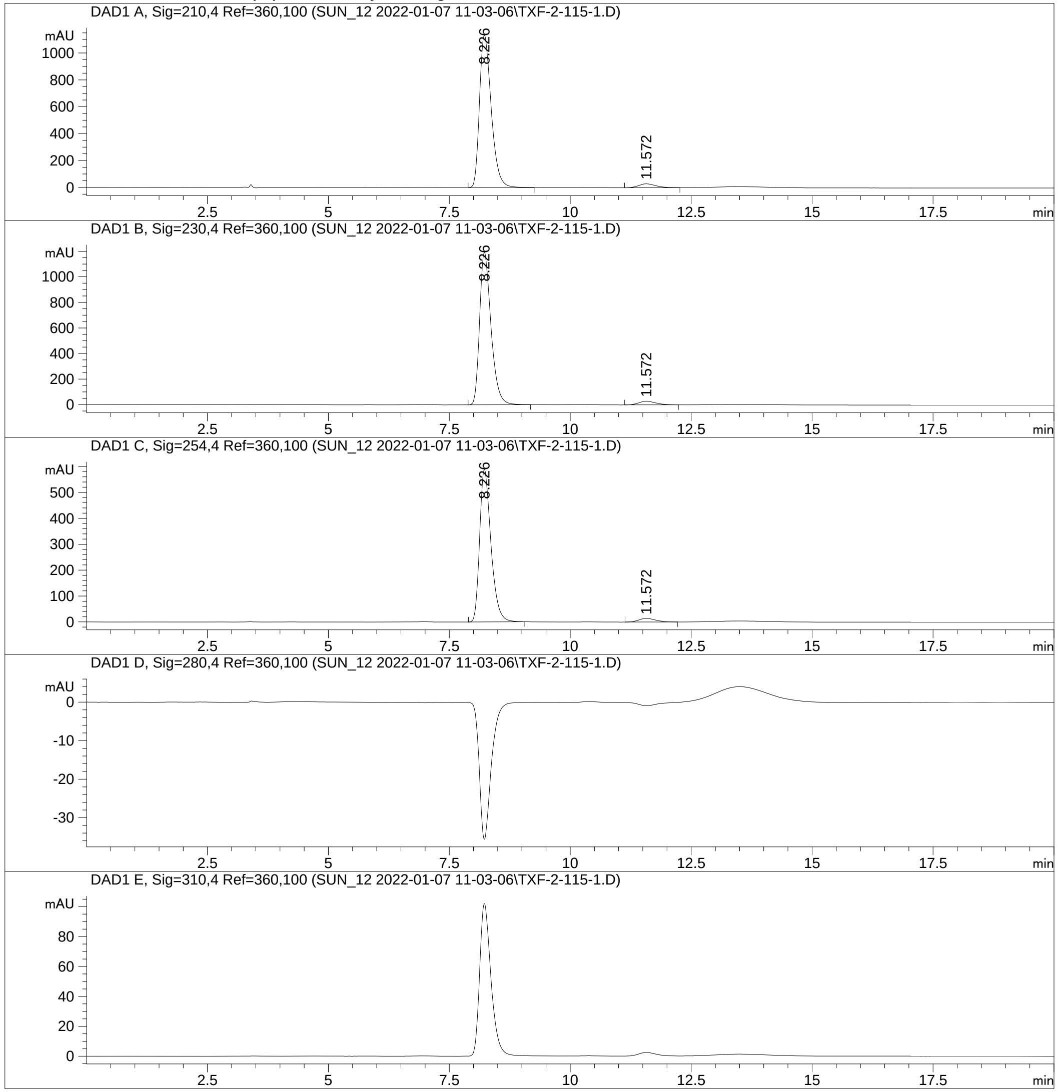

Data File C:\CHEM32\1\DATA\SUN_12 2022-01-07 11-03-06\TXF-2-115-1.D
Sample Name:

Area Percent Report

Sorted By : Signal
Multiplier : 1.0000
Dilution : 1.0000
Use Multiplier & Dilution Factor with ISTDs

Signal 1: DAD1 A, Sig=210,4 Ref=360,100

Peak #	RetTime [min]	Type	Width [min]	Area [mAU*s]	Height [mAU]	Area %
1	8.226	BB	0.2590	1.91176e4	1132.07471	96.4261
2	11.572	BB	0.3778	708.57434	29.12740	3.5739

Totals : 1.98262e4 1161.20211

Signal 2: DAD1 B, Sig=230,4 Ref=360,100

Peak #	RetTime [min]	Type	Width [min]	Area [mAU*s]	Height [mAU]	Area %
1	8.226	BB	0.2509	1.94965e4	1191.21472	96.5096
2	11.572	BB	0.3775	705.11499	28.81731	3.4904

Totals : 2.02016e4 1220.03203

Signal 3: DAD1 C, Sig=254,4 Ref=360,100

Peak #	RetTime [min]	Type	Width [min]	Area [mAU*s]	Height [mAU]	Area %
1	8.226	BB	0.2475	9556.65723	588.30548	96.5577
2	11.572	BB	0.3758	340.69388	14.00360	3.4423

Totals : 9897.35110 602.30908

Signal 4: DAD1 D, Sig=280,4 Ref=360,100

Signal 5: DAD1 E, Sig=310,4 Ref=360,100

*** End of Report ***

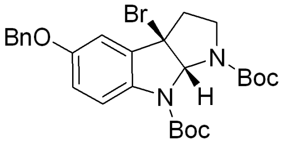

2j
(enantioenriched)

Supplementary Figure 124. HPLC Spectrum of **2j** (enantioenriched)

===

Acq. Operator : Seq. Line : 2
Acq. Instrument : Instrument 1 Location : Vial 1
Injection Date : 1/13/2022 10:50:48 PM Inj : 1
Inj Volume : 5.000 µl
Different Inj Volume from Sequence ! Actual Inj Volume : 2.000 µl
Acq. Method : C:\CHEM32\1\DATA\SUN_12 2022-01-13 22-38-00\IC-01-20.M
Last changed : 1/13/2022 10:49:55 PM
(modified after loading)
Analysis Method : C:\CHEM32\1\METHODS\OD-03-60-0.6.M
Last changed : 3/3/2022 9:54:51 PM
Additional Info : Peak(s) manually integrated

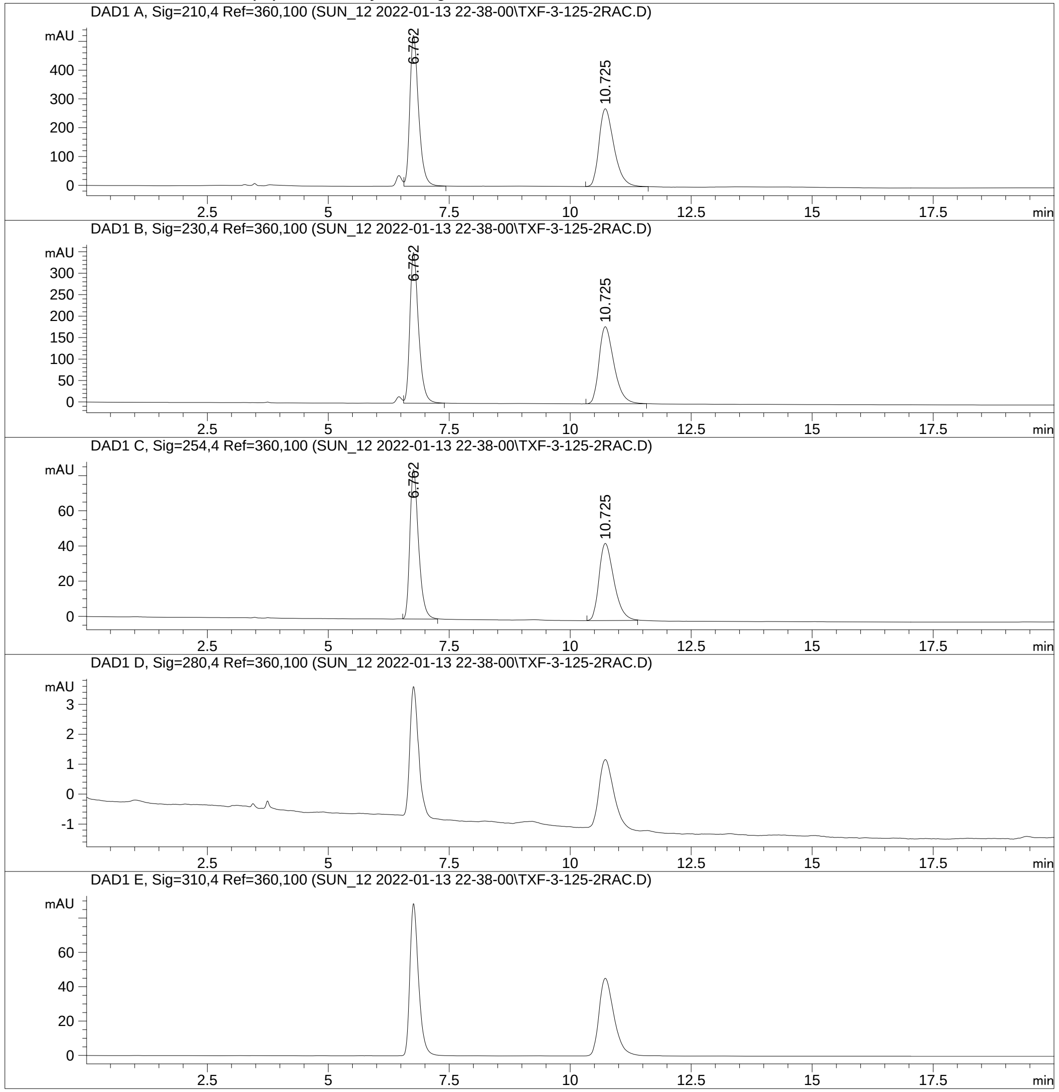

Data File C:\CHEM32\1\DATA\SUN_12 2022-01-13 22-38-00\TXF-3-125-2RAC.D
Sample Name:

===

Area Percent Report

===

Sorted By : Signal
Multiplier : 1.0000
Dilution : 1.0000
Use Multiplier & Dilution Factor with ISTDs

Signal 1: DAD1 A, Sig=210,4 Ref=360,100

Peak #	RetTime [min]	Type	Width [min]	Area [mAU*s]	Height [mAU]	Area %
1	6.762	VB	0.1908	6591.38037	524.00769	53.0567
2	10.725	BB	0.3294	5831.90234	270.61572	46.9433
Totals :				1.24233e4	794.62341	

Signal 2: DAD1 B, Sig=230,4 Ref=360,100

Peak #	RetTime [min]	Type	Width [min]	Area [mAU*s]	Height [mAU]	Area %
1	6.762	VB	0.1896	4384.31641	351.28815	53.1479
2	10.725	BB	0.3288	3864.95728	179.75935	46.8521
Totals :				8249.27368	531.04750	

Signal 3: DAD1 C, Sig=254,4 Ref=360,100

Peak #	RetTime [min]	Type	Width [min]	Area [mAU*s]	Height [mAU]	Area %
1	6.762	BB	0.1894	1059.91321	85.04692	53.0353
2	10.725	BB	0.3295	938.59363	43.89342	46.9647
Totals :				1998.50684	128.94035	

Signal 4: DAD1 D, Sig=280,4 Ref=360,100

Signal 5: DAD1 E, Sig=310,4 Ref=360,100

===

*** End of Report ***

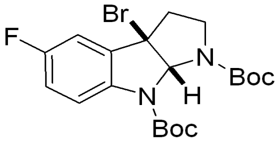

2k
(racemic)

Supplementary Figure 125. HPLC Spectrum of **2k** (racemic)

```
=====================================================================
Acq. Operator   :                                 Seq. Line :    3
Acq. Instrument : Instrument 1                     Location : Vial 2
Injection Date  : 1/13/2022 11:11:43 PM                 Inj :    1
                                                 Inj Volume : 5.000 µl
Different Inj Volume from Sequence !     Actual Inj Volume : 2.000 µl
Acq. Method     : C:\CHEM32\1\DATA\SUN_12 2022-01-13 22-38-00\IC-01-20.M
Last changed    : 1/13/2022 10:49:55 PM
                  (modified after loading)
Analysis Method : C:\CHEM32\1\METHODS\OD-03-60-0.6.M
Last changed    : 3/3/2022 9:54:51 PM
Additional Info : Peak(s) manually integrated
```

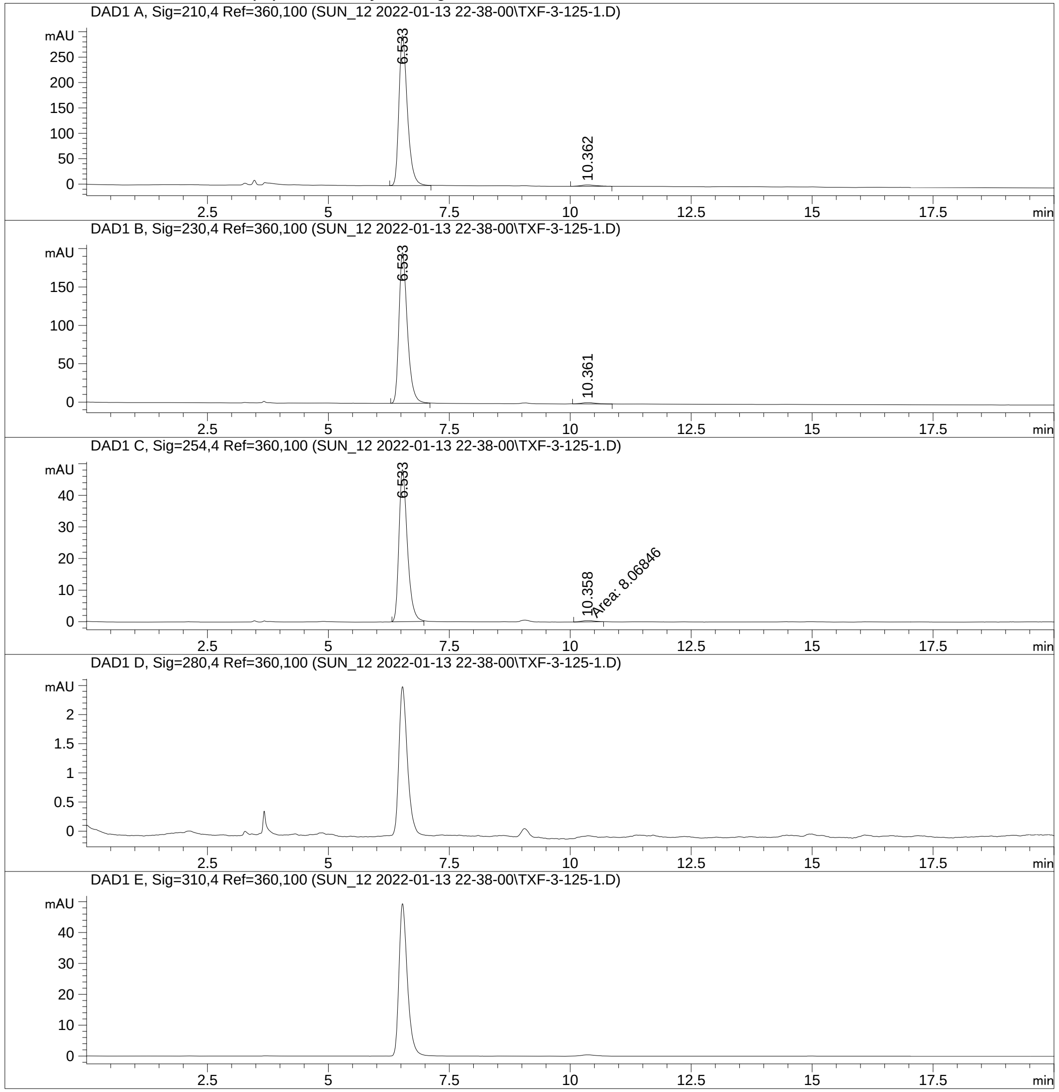

Data File C:\CHEM32\1\DATA\SUN_12 2022-01-13 22-38-00\TXF-3-125-1.D
Sample Name:

Area Percent Report

```
Sorted By             :      Signal
Multiplier            :      1.0000
Dilution              :      1.0000
Use Multiplier & Dilution Factor with ISTDs
```

Signal 1: DAD1 A, Sig=210,4 Ref=360,100

Peak #	RetTime [min]	Type	Width [min]	Area [mAU*s]	Height [mAU]	Area %
1	6.533	BB	0.1855	3586.88574	295.80911	98.3649
2	10.362	BB	0.2992	59.62311	2.72593	1.6351

Totals : 3646.50885 298.53504

Signal 2: DAD1 B, Sig=230,4 Ref=360,100

Peak #	RetTime [min]	Type	Width [min]	Area [mAU*s]	Height [mAU]	Area %
1	6.533	BB	0.1833	2380.57007	196.50677	98.5381
2	10.361	BB	0.3194	35.31897	1.69303	1.4619

Totals : 2415.88904 198.19981

Signal 3: DAD1 C, Sig=254,4 Ref=360,100

Peak #	RetTime [min]	Type	Width [min]	Area [mAU*s]	Height [mAU]	Area %
1	6.533	BB	0.1853	583.33203	48.16604	98.6357
2	10.358	MM	0.3183	8.06846	4.22464e-1	1.3643

Totals : 591.40049 48.58851

Signal 4: DAD1 D, Sig=280,4 Ref=360,100

Signal 5: DAD1 E, Sig=310,4 Ref=360,100

*** End of Report ***

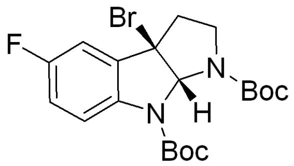

2k
(enantioenriched)

Supplementary Figure 126. HPLC Spectrum of **2k** (enantioenriched)

```
=====================================================================
Acq. Operator   :                              Seq. Line :  17
Acq. Instrument : Instrument 1                  Location : Vial 7
Injection Date  : 12/19/2021 12:48:28 AM             Inj :   1
                                              Inj Volume : 5.000 µl
Different Inj Volume from Sequence !     Actual Inj Volume : 2.000 µl
Acq. Method     : C:\CHEM32\1\DATA\SUN_12 2021-12-18 19-00-37\IC-04-20.M
Last changed    : 3/28/2021 9:30:05 AM
Analysis Method : C:\CHEM32\1\METHODS\OD-03-60-0.6.M
Last changed    : 3/12/2022 7:32:45 PM
                  (modified after loading)
Additional Info : Peak(s) manually integrated
```

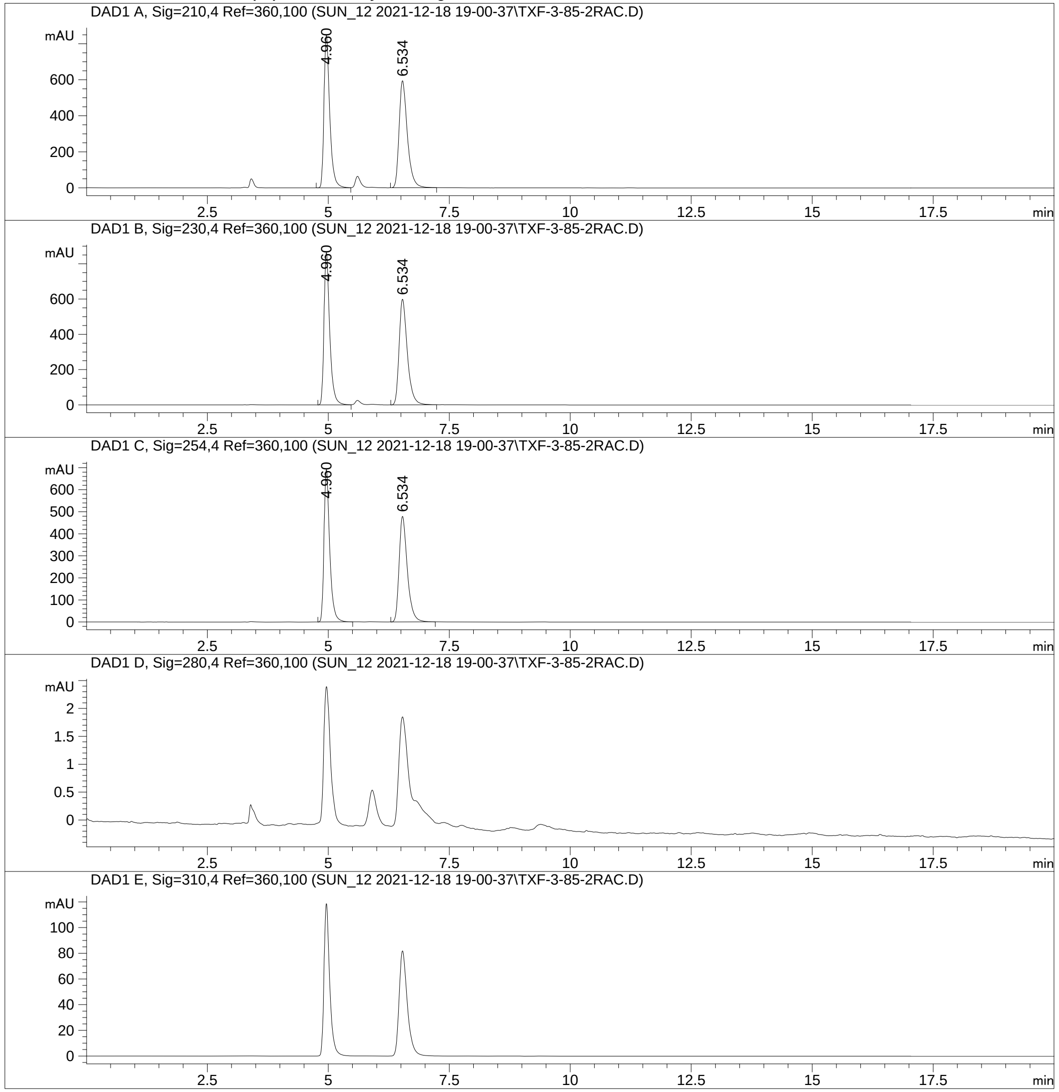

Data File C:\CHEM32\1\DATA\SUN_12 2021-12-18 19-00-37\TXF-3-85-2RAC.D
Sample Name:

```
=====================================================================
                         Area Percent Report
=====================================================================

Sorted By             :      Signal
Multiplier            :      1.0000
Dilution              :      1.0000
Use Multiplier & Dilution Factor with ISTDs
```

Signal 1: DAD1 A, Sig=210,4 Ref=360,100

Peak #	RetTime [min]	Type	Width [min]	Area [mAU*s]	Height [mAU]	Area %
1	4.960	BV	0.1244	7000.92676	847.56354	49.7473
2	6.534	BB	0.1788	7072.05713	594.53143	50.2527

Totals : 1.40730e4 1442.09497

Signal 2: DAD1 B, Sig=230,4 Ref=360,100

Peak #	RetTime [min]	Type	Width [min]	Area [mAU*s]	Height [mAU]	Area %
1	4.960	BV	0.1214	7073.22217	865.28894	49.9586
2	6.534	BB	0.1779	7084.95068	599.26898	50.0414

Totals : 1.41582e4 1464.55792

Signal 3: DAD1 C, Sig=254,4 Ref=360,100

Peak #	RetTime [min]	Type	Width [min]	Area [mAU*s]	Height [mAU]	Area %
1	4.960	BB	0.1211	5645.26904	692.44287	49.9208
2	6.534	BB	0.1779	5663.18457	479.18460	50.0792

Totals : 1.13085e4 1171.62747

Signal 4: DAD1 D, Sig=280,4 Ref=360,100

Signal 5: DAD1 E, Sig=310,4 Ref=360,100

```
=====================================================================
                         *** End of Report ***
```

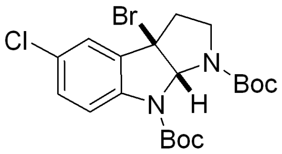

2l
(racemic)

Supplementary Figure 127. HPLC Spectrum of **2l** (racemic)

Data File C:\CHEM32\1\DATA\SUN_12 2021-12-18 19-00-37\TXF-3-85-3.D
Sample Name:

===

Acq. Operator : Seq. Line : 18
Acq. Instrument : Instrument 1 Location : Vial 8
Injection Date : 12/19/2021 1:09:25 AM Inj : 1
Inj Volume : 5.000 µl
Different Inj Volume from Sequence ! Actual Inj Volume : 2.000 µl
Acq. Method : C:\CHEM32\1\DATA\SUN_12 2021-12-18 19-00-37\IC-04-20.M
Last changed : 3/28/2021 9:30:05 AM
Analysis Method : C:\CHEM32\1\METHODS\OD-03-60-0.6.M
Last changed : 3/12/2022 7:32:45 PM
(modified after loading)
Additional Info : Peak(s) manually integrated

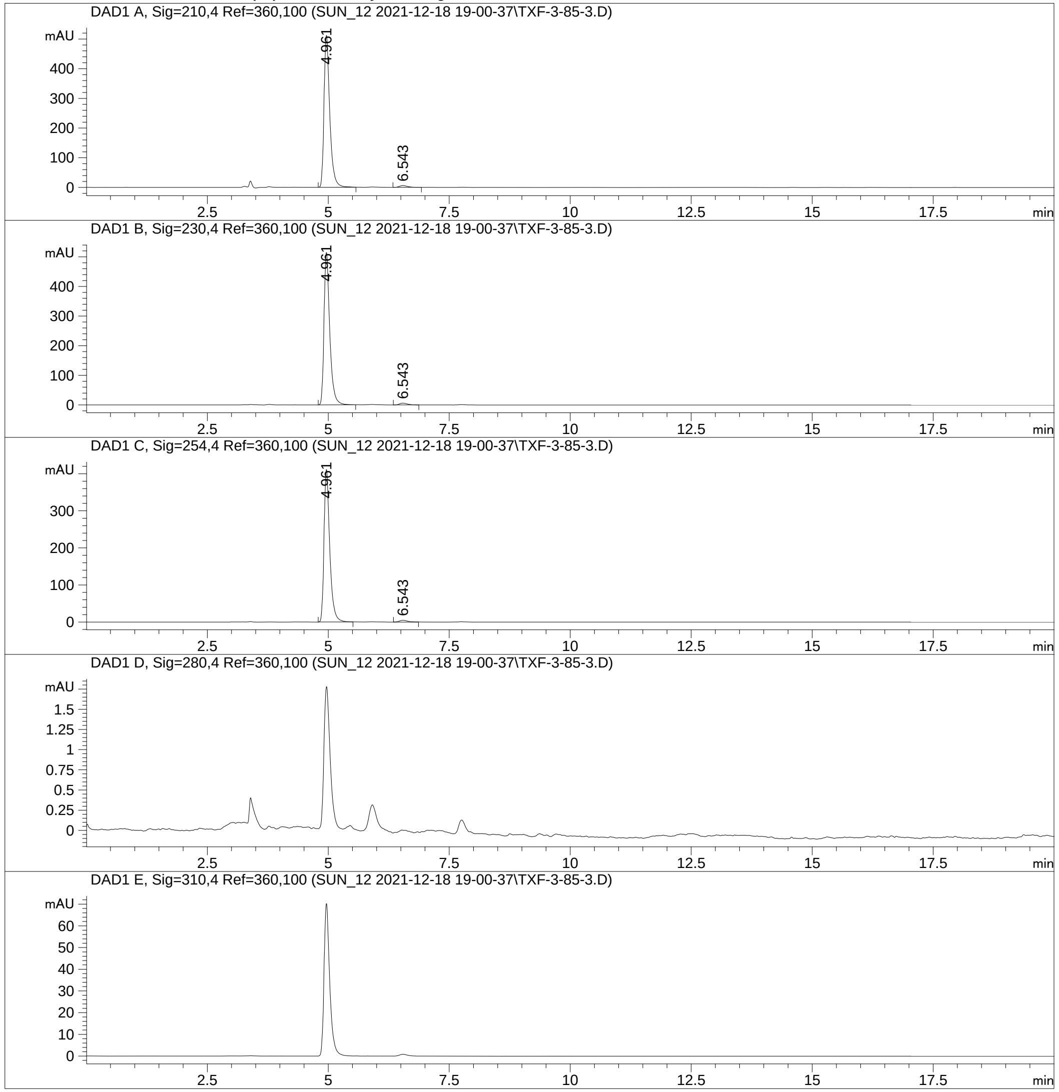

Data File C:\CHEM32\1\DATA\SUN_12 2021-12-18 19-00-37\TXF-3-85-3.D
Sample Name:

===

Area Percent Report

===

Sorted By : Signal
Multiplier : 1.0000
Dilution : 1.0000
Use Multiplier & Dilution Factor with ISTDs

Signal 1: DAD1 A, Sig=210,4 Ref=360,100

Peak #	RetTime [min]	Type	Width [min]	Area [mAU*s]	Height [mAU]	Area %
1	4.961	BB	0.1237	4207.01709	512.73444	98.3380
2	6.543	BB	0.1803	71.10410	6.08441	1.6620

Totals : 4278.12119 518.81885

Signal 2: DAD1 B, Sig=230,4 Ref=360,100

Peak #	RetTime [min]	Type	Width [min]	Area [mAU*s]	Height [mAU]	Area %
1	4.961	BB	0.1233	4213.24023	515.68512	98.3681
2	6.543	BB	0.1746	69.89822	6.05995	1.6319

Totals : 4283.13846 521.74507

Signal 3: DAD1 C, Sig=254,4 Ref=360,100

Peak #	RetTime [min]	Type	Width [min]	Area [mAU*s]	Height [mAU]	Area %
1	4.961	BB	0.1232	3363.69922	412.04279	98.3691
2	6.543	BB	0.1746	55.76674	4.83312	1.6309

Totals : 3419.46596 416.87591

Signal 4: DAD1 D, Sig=280,4 Ref=360,100

Signal 5: DAD1 E, Sig=310,4 Ref=360,100

===

*** End of Report ***

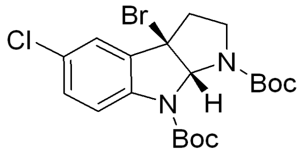

2l
(enantioenriched)

Supplementary Figure 128. HPLC Spectrum of **2l** (enantioenriched)

```
=====================================================================
Acq. Operator   :                                 Seq. Line :   2
Acq. Instrument : Instrument 1                     Location : Vial 1
Injection Date  : 12/23/2021 10:53:42 PM                Inj :   1
                                                 Inj Volume : 5.000 µl
Different Inj Volume from Sequence !     Actual Inj Volume : 2.000 µl
Acq. Method     : C:\CHEM32\1\DATA\SUN_12 2021-12-23 22-38-16\IC-02-20.M
Last changed    : 12/23/2021 10:52:50 PM
                  (modified after loading)
Analysis Method : C:\CHEM32\1\METHODS\OD-03-60-0.6.M
Last changed    : 3/3/2022 9:54:51 PM
Additional Info : Peak(s) manually integrated
```

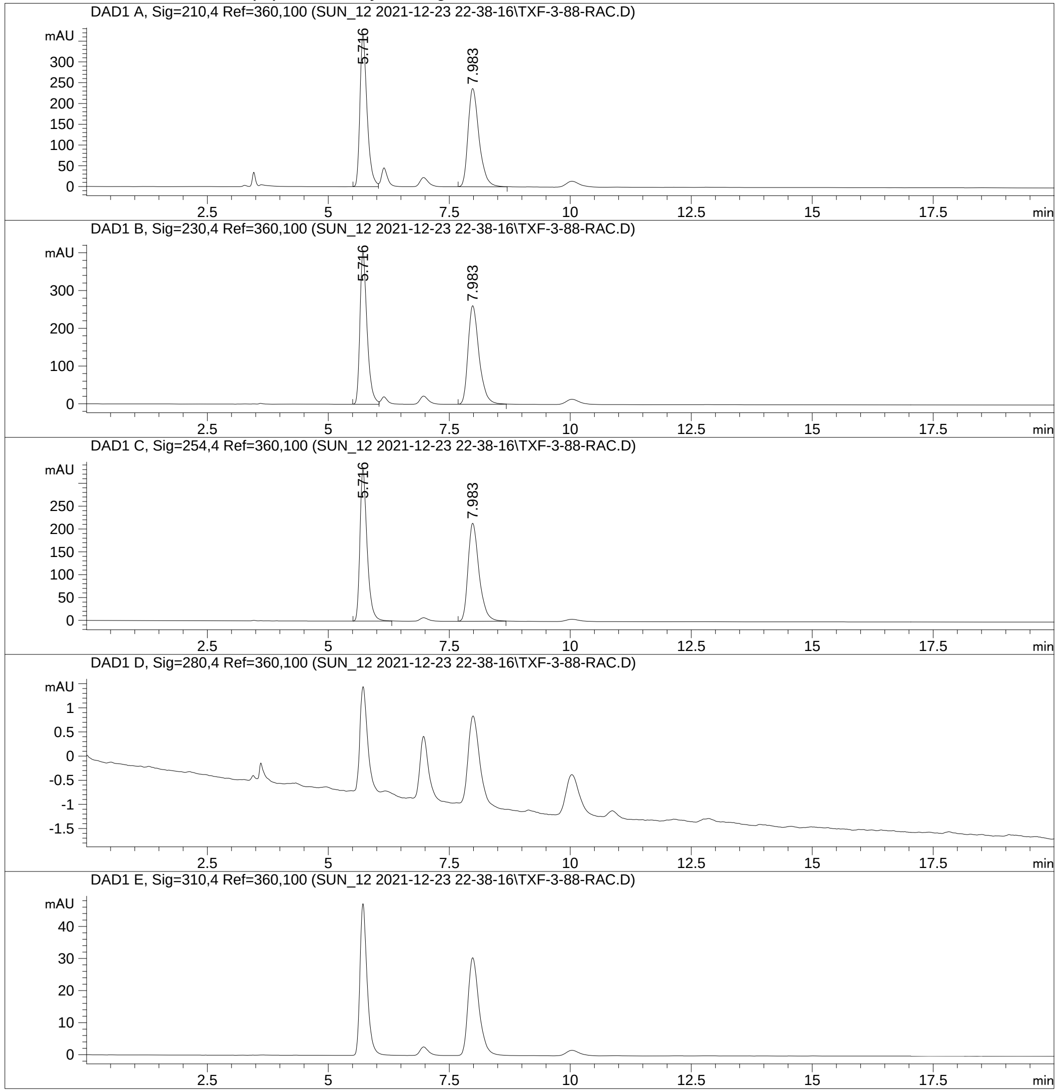

Data File C:\CHEM32\1\DATA\SUN_12 2021-12-23 22-38-16\TXF-3-88-RAC.D
Sample Name:

Area Percent Report

```
Sorted By             :      Signal
Multiplier            :      1.0000
Dilution              :      1.0000
Use Multiplier & Dilution Factor with ISTDs
```

Signal 1: DAD1 A, Sig=210,4 Ref=360,100

Peak #	RetTime [min]	Type	Width [min]	Area [mAU*s]	Height [mAU]	Area %
1	5.716	BV	0.1554	3725.48926	364.17041	49.8854
2	7.983	BB	0.2425	3742.60034	236.60272	50.1146

Totals : 7468.08960 600.77313

Signal 2: DAD1 B, Sig=230,4 Ref=360,100

Peak #	RetTime [min]	Type	Width [min]	Area [mAU*s]	Height [mAU]	Area %
1	5.716	BV	0.1552	4108.42627	401.97891	49.9358
2	7.983	BB	0.2401	4118.98682	261.00769	50.0642

Totals : 8227.41309 662.98660

Signal 3: DAD1 C, Sig=254,4 Ref=360,100

Peak #	RetTime [min]	Type	Width [min]	Area [mAU*s]	Height [mAU]	Area %
1	5.716	BB	0.1558	3408.00806	331.79187	50.1777
2	7.983	BB	0.2401	3383.87061	214.49803	49.8223

Totals : 6791.87866 546.28990

Signal 4: DAD1 D, Sig=280,4 Ref=360,100

Signal 5: DAD1 E, Sig=310,4 Ref=360,100

*** End of Report ***

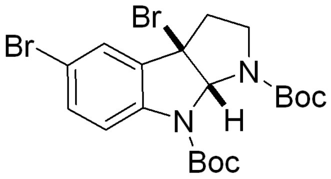

2m
(racemic)

Supplementary Figure 129. HPLC Spectrum of **2m** (racemic)

Data File C:\CHEM32\1\DATA\SUN_12 2021-12-23 22-38-16\TXF-3-88.D
Sample Name:

===

Acq. Operator : Seq. Line : 3
Acq. Instrument : Instrument 1 Location : Vial 2
Injection Date : 12/23/2021 11:14:37 PM Inj : 1
Inj Volume : 5.000 µl
Different Inj Volume from Sequence ! Actual Inj Volume : 2.000 µl
Acq. Method : C:\CHEM32\1\DATA\SUN_12 2021-12-23 22-38-16\IC-02-20.M
Last changed : 12/23/2021 10:52:50 PM
(modified after loading)
Analysis Method : C:\CHEM32\1\METHODS\OD-03-60-0.6.M
Last changed : 3/3/2022 9:54:51 PM
Additional Info : Peak(s) manually integrated

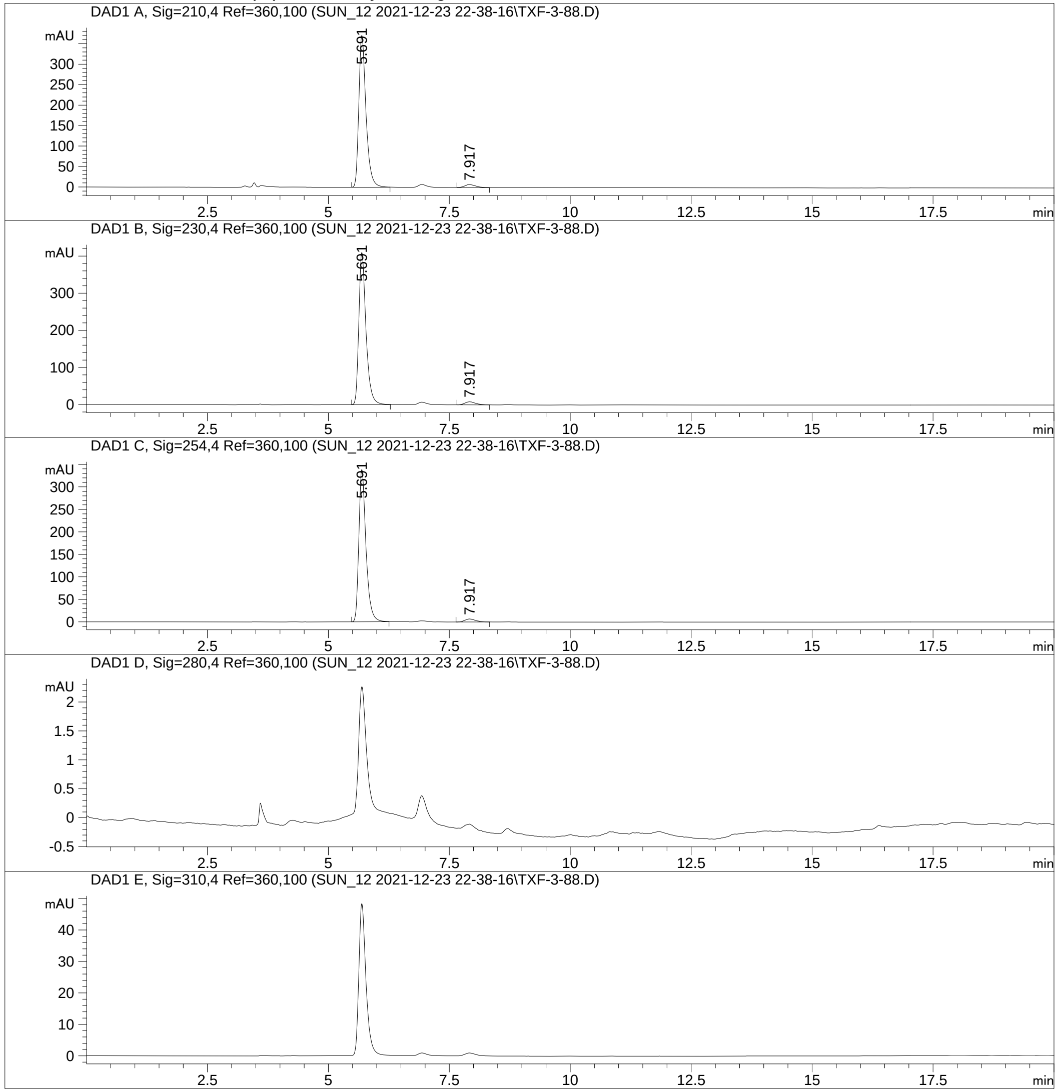

Data File C:\CHEM32\1\DATA\SUN_12 2021-12-23 22-38-16\TXF-3-88.D
Sample Name:

===

Area Percent Report

===

Sorted By : Signal
Multiplier : 1.0000
Dilution : 1.0000
Use Multiplier & Dilution Factor with ISTDs

Signal 1: DAD1 A, Sig=210,4 Ref=360,100

Peak #	RetTime [min]	Type	Width [min]	Area [mAU*s]	Height [mAU]	Area %
1	5.691	BB	0.1562	3823.81616	371.22287	97.0366
2	7.917	BB	0.2425	116.77749	7.46707	2.9634

Totals : 3940.59365 378.68994

Signal 2: DAD1 B, Sig=230,4 Ref=360,100

Peak #	RetTime [min]	Type	Width [min]	Area [mAU*s]	Height [mAU]	Area %
1	5.691	BB	0.1558	4211.39258	410.13028	97.0436
2	7.917	BB	0.2377	128.30009	8.23868	2.9564

Totals : 4339.69267 418.36895

Signal 3: DAD1 C, Sig=254,4 Ref=360,100

Peak #	RetTime [min]	Type	Width [min]	Area [mAU*s]	Height [mAU]	Area %
1	5.691	BB	0.1556	3472.20239	338.71628	97.0960
2	7.917	BB	0.2397	103.84917	6.66765	2.9040

Totals : 3576.05157 345.38393

Signal 4: DAD1 D, Sig=280,4 Ref=360,100

Signal 5: DAD1 E, Sig=310,4 Ref=360,100

===

*** End of Report ***

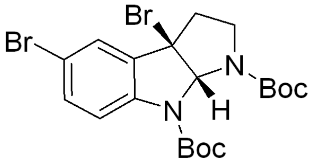

2m
(enantioenriched)

Supplementary Figure 130. HPLC Spectrum of **2m** (enantioenriched)

Data File C:\CHEM32\1\DATA\SUN_12 2022-01-21 15-02-39\TXF-3-134-2RAC.D
Sample Name:

===

```
Acq. Operator   :                              Seq. Line :   7
Acq. Instrument : Instrument 1                  Location : Vial 8
Injection Date  : 1/21/2022 5:50:29 PM               Inj :   1
                                              Inj Volume : 5.000 µl
Different Inj Volume from Sequence !     Actual Inj Volume : 2.000 µl
Acq. Method     : C:\CHEM32\1\DATA\SUN_12 2022-01-21 15-02-39\IC-01-20.M
Last changed    : 1/21/2022 5:07:42 PM
                  (modified after loading)
Analysis Method : C:\CHEM32\1\METHODS\OD-03-60-0.6.M
Last changed    : 3/3/2022 9:54:51 PM
Additional Info : Peak(s) manually integrated
```

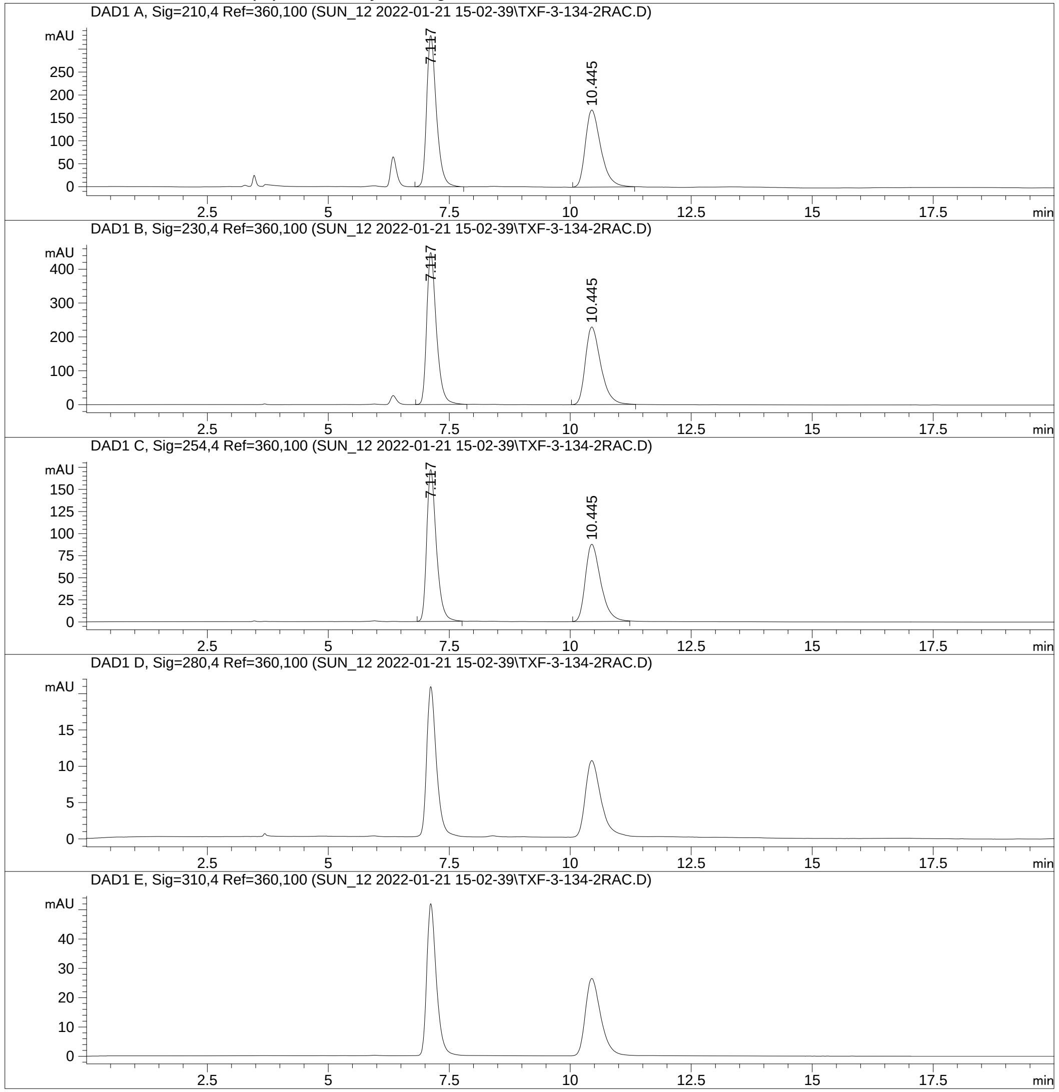

Data File C:\CHEM32\1\DATA\SUN_12 2022-01-21 15-02-39\TXF-3-134-2RAC.D
Sample Name:

===

Area Percent Report

===

```
Sorted By            :      Signal
Multiplier           :      1.0000
Dilution             :      1.0000
Use Multiplier & Dilution Factor with ISTDs
```

Signal 1: DAD1 A, Sig=210,4 Ref=360,100

Peak #	RetTime [min]	Type	Width [min]	Area [mAU*s]	Height [mAU]	Area %
1	7.117	BB	0.2121	4580.96582	329.68082	55.0731
2	10.445	BB	0.3396	3737.01440	167.92422	44.9269

Totals : 8317.98022 497.60504

Signal 2: DAD1 B, Sig=230,4 Ref=360,100

Peak #	RetTime [min]	Type	Width [min]	Area [mAU*s]	Height [mAU]	Area %
1	7.117	BB	0.2118	6217.94336	448.44330	54.9486
2	10.445	BB	0.3396	5097.98193	229.11920	45.0514

Totals : 1.13159e4 677.56250

Signal 3: DAD1 C, Sig=254,4 Ref=360,100

Peak #	RetTime [min]	Type	Width [min]	Area [mAU*s]	Height [mAU]	Area %
1	7.117	BB	0.2094	2376.95093	171.86595	55.0910
2	10.445	BB	0.3385	1937.64136	87.43691	44.9090

Totals : 4314.59229 259.30286

Signal 4: DAD1 D, Sig=280,4 Ref=360,100

Signal 5: DAD1 E, Sig=310,4 Ref=360,100

===

*** End of Report ***

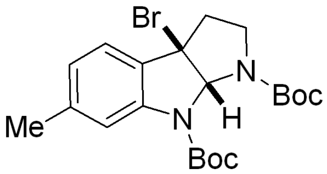

2n
(racemic)

Supplementary Figure 131. HPLC Spectrum of **2n** (racemic)

```
=====================================================================
Acq. Operator   :                                 Seq. Line :   8
Acq. Instrument : Instrument 1                     Location : Vial 9
Injection Date  : 1/21/2022 6:11:28 PM                   Inj :   1
                                                Inj Volume : 5.000 µl
Different Inj Volume from Sequence !     Actual Inj Volume : 2.000 µl
Acq. Method     : C:\CHEM32\1\DATA\SUN_12 2022-01-21 15-02-39\IC-01-20.M
Last changed    : 1/21/2022 5:07:42 PM
                  (modified after loading)
Analysis Method : C:\CHEM32\1\METHODS\OD-03-60-0.6.M
Last changed    : 3/3/2022 9:54:51 PM
Additional Info : Peak(s) manually integrated
```

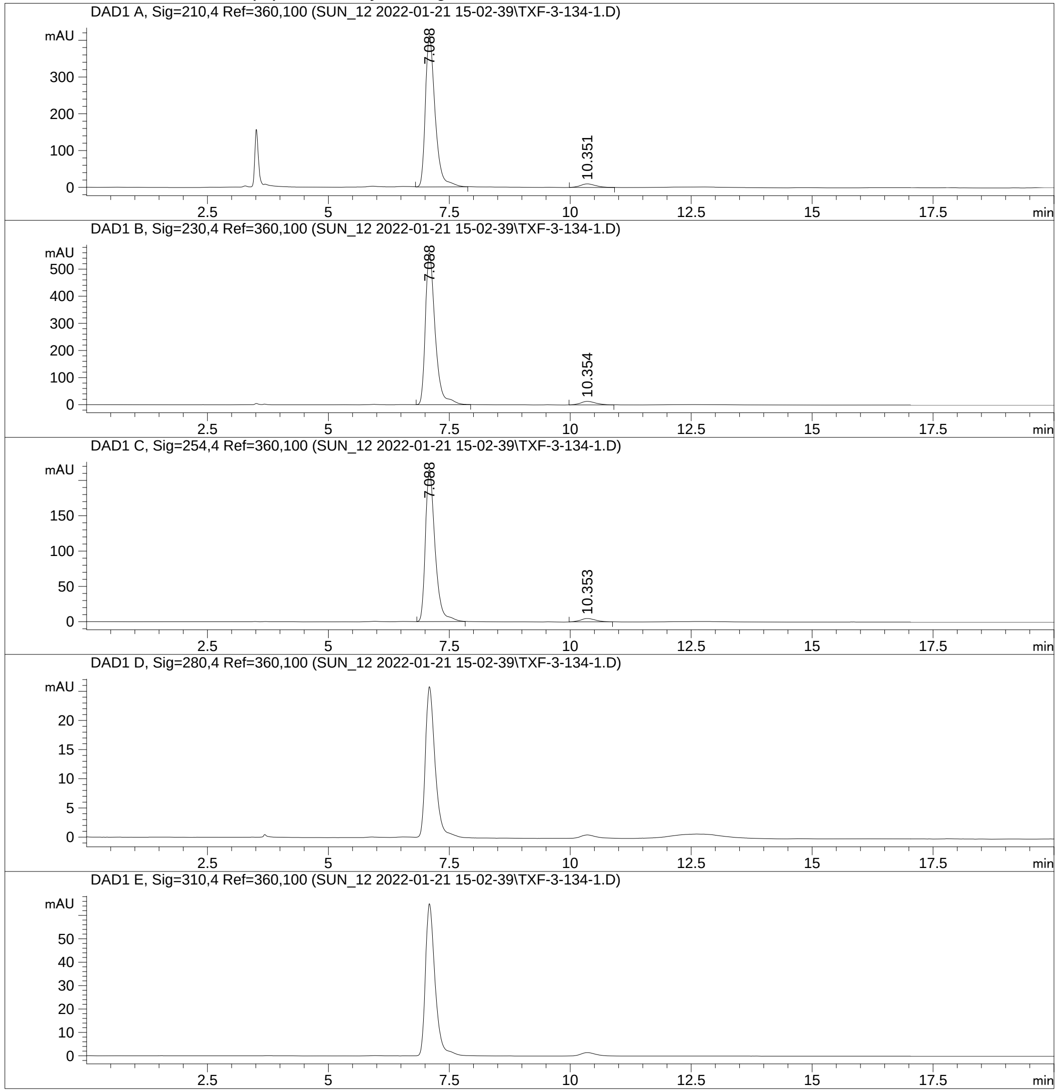

Data File C:\CHEM32\1\DATA\SUN_12 2022-01-21 15-02-39\TXF-3-134-1.D
Sample Name:

===
Area Percent Report
===

```
Sorted By             :      Signal
Multiplier            :      1.0000
Dilution              :      1.0000
Use Multiplier & Dilution Factor with ISTDs
```

Signal 1: DAD1 A, Sig=210,4 Ref=360,100

Peak #	RetTime [min]	Type	Width [min]	Area [mAU*s]	Height [mAU]	Area %
1	7.088	VB	0.2127	5742.99365	411.73129	96.4627
2	10.351	BB	0.3262	210.59422	9.74345	3.5373
Totals :				5953.58788	421.47475	

Signal 2: DAD1 B, Sig=230,4 Ref=360,100

Peak #	RetTime [min]	Type	Width [min]	Area [mAU*s]	Height [mAU]	Area %
1	7.088	BB	0.2131	7843.49072	561.04999	96.6238
2	10.354	BB	0.3242	274.06503	12.88356	3.3762
Totals :				8117.55576	573.93355	

Signal 3: DAD1 C, Sig=254,4 Ref=360,100

Peak #	RetTime [min]	Type	Width [min]	Area [mAU*s]	Height [mAU]	Area %
1	7.088	BB	0.2113	2976.07202	215.20959	96.7019
2	10.353	BB	0.3194	101.50182	4.82487	3.2981
Totals :				3077.57384	220.03446	

Signal 4: DAD1 D, Sig=280,4 Ref=360,100

Signal 5: DAD1 E, Sig=310,4 Ref=360,100

===
*** End of Report ***

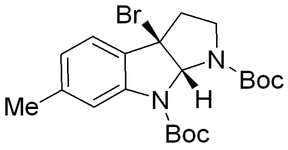

2n
(enantioenriched)

Supplementary Figure 132. HPLC Spectrum of **2n** (enantioenriched)

Data File C:\CHEM32\1\DATA\SUN_12 2021-12-24 14-13-09\TXF-3-94-RAC2.D
Sample Name:

```
=====================================================================
Acq. Operator   :                        Seq. Line :   6
Acq. Instrument : Instrument 1            Location : Vial 1
Injection Date  : 12/24/2021 3:22:03 PM        Inj :   1
                                        Inj Volume : 5.000 µl
Different Inj Volume from Sequence !   Actual Inj Volume : 2.000 µl
Acq. Method     : C:\CHEM32\1\DATA\SUN_12 2021-12-24 14-13-09\IC-01-20.M
Last changed    : 12/24/2021 3:36:05 PM
                  (modified after loading)
Analysis Method : C:\CHEM32\1\METHODS\OD-03-60-0.6.M
Last changed    : 3/3/2022 9:54:51 PM
Additional Info : Peak(s) manually integrated
```

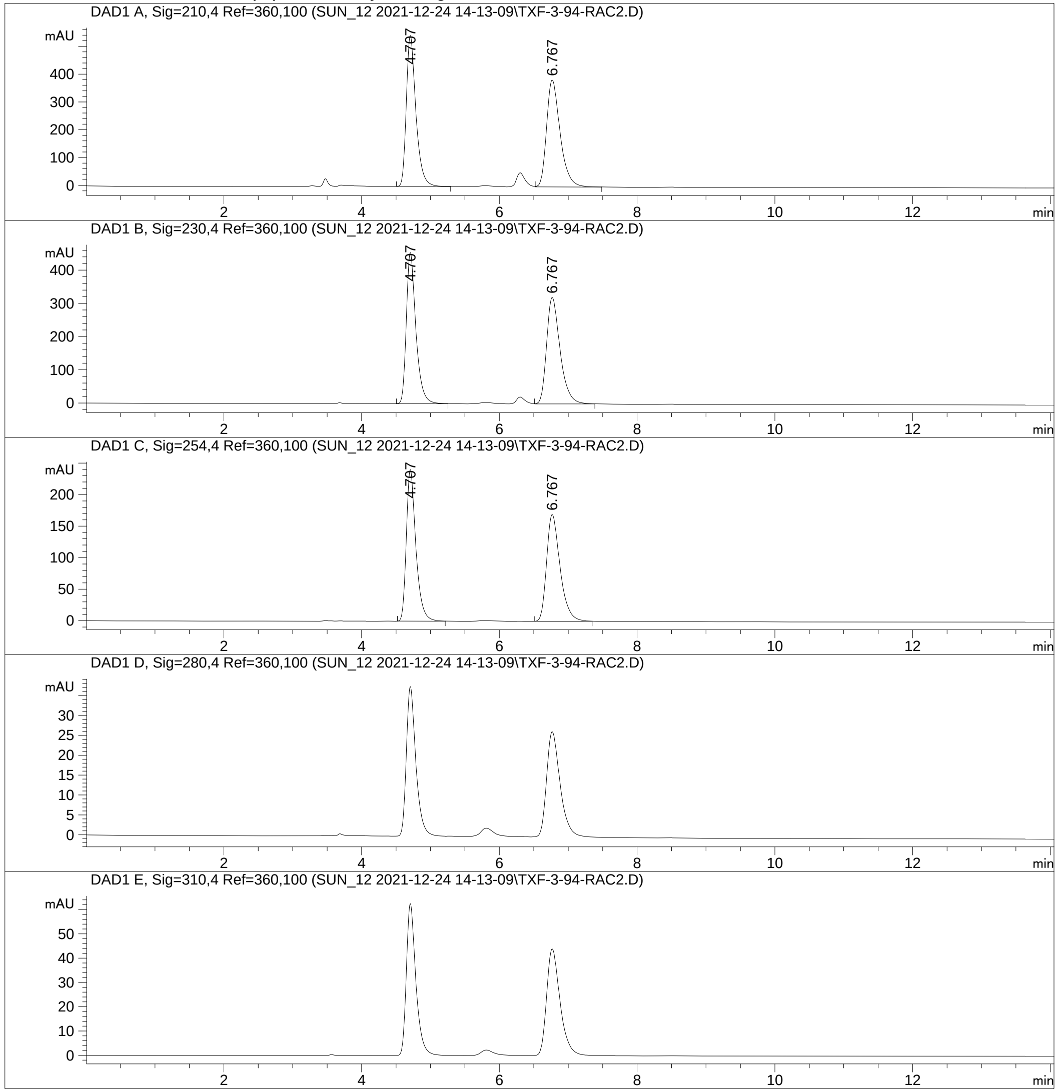

Data File C:\CHEM32\1\DATA\SUN_12 2021-12-24 14-13-09\TXF-3-94-RAC2.D
Sample Name:

Area Percent Report

Sorted By : Signal
Multiplier : 1.0000
Dilution : 1.0000
Use Multiplier & Dilution Factor with ISTDs

Signal 1: DAD1 A, Sig=210,4 Ref=360,100

Peak #	RetTime [min]	Type	Width [min]	Area [mAU*s]	Height [mAU]	Area %
1	4.707	BB	0.1466	5243.32910	543.16559	49.9266
2	6.767	VB	0.2075	5258.74072	384.54654	50.0734

Totals : 1.05021e4 927.71213

Signal 2: DAD1 B, Sig=230,4 Ref=360,100

Peak #	RetTime [min]	Type	Width [min]	Area [mAU*s]	Height [mAU]	Area %
1	4.707	BB	0.1461	4373.26904	455.14401	50.0504
2	6.767	VB	0.2068	4364.45752	320.67175	49.9496

Totals : 8737.72656 775.81577

Signal 3: DAD1 C, Sig=254,4 Ref=360,100

Peak #	RetTime [min]	Type	Width [min]	Area [mAU*s]	Height [mAU]	Area %
1	4.707	BB	0.1460	2307.94971	240.33946	50.0778
2	6.767	BB	0.2065	2300.77930	169.33157	49.9222

Totals : 4608.72900 409.67104

Signal 4: DAD1 D, Sig=280,4 Ref=360,100

Signal 5: DAD1 E, Sig=310,4 Ref=360,100

*** End of Report ***

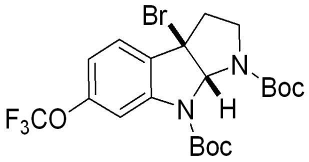

2o
(racemic)

Supplementary Figure 133. HPLC Spectrum of **2o** (racemic)

===

Acq. Operator   :   Seq. Line :   7
Acq. Instrument : Instrument 1   Location : Vial 2
Injection Date : 12/24/2021 3:37:03 PM   Inj :   1
                                   Inj Volume : 5.000 µl
Different Inj Volume from Sequence !   Actual Inj Volume : 2.000 µl
Acq. Method   : C:\CHEM32\1\DATA\SUN_12 2021-12-24 14-13-09\IC-01-20.M
Last changed   : 12/24/2021 3:36:05 PM
                  (modified after loading)
Analysis Method : C:\CHEM32\1\METHODS\OD-03-60-0.6.M
Last changed   : 3/3/2022 9:54:51 PM
Additional Info : Peak(s) manually integrated

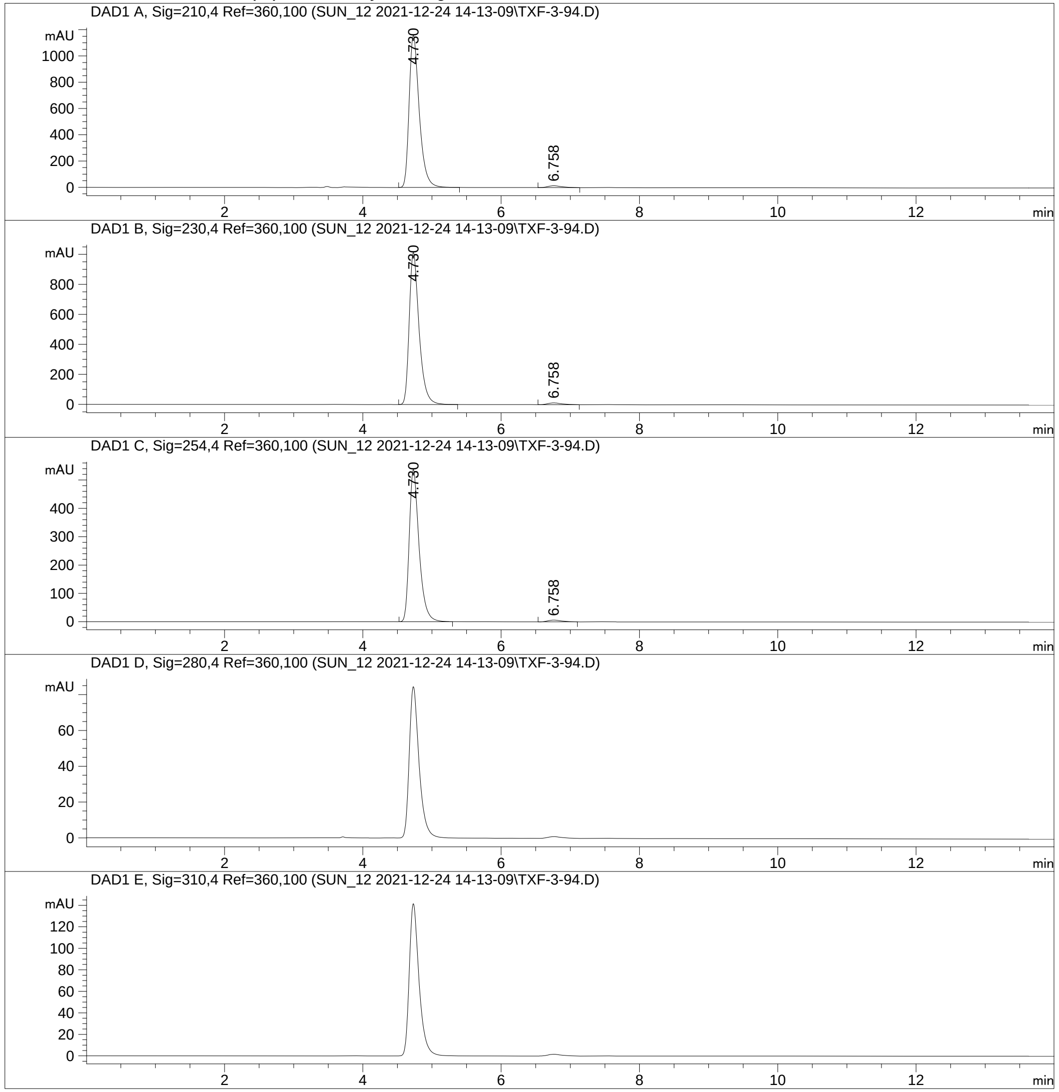

Data File C:\CHEM32\1\DATA\SUN_12 2021-12-24 14-13-09\TXF-3-94.D
Sample Name:

===

Area Percent Report

===

Sorted By   :   Signal
Multiplier   :   1.0000
Dilution   :   1.0000
Use Multiplier & Dilution Factor with ISTDs

Signal 1: DAD1 A, Sig=210,4 Ref=360,100

Peak #	RetTime [min]	Type	Width [min]	Area [mAU*s]	Height [mAU]	Area %
1	4.730	BB	0.1547	1.17754e4	1157.48254	98.4288
2	6.758	BB	0.1986	187.97305	14.37082	1.5712

Totals :   1.19633e4 1171.85336

Signal 2: DAD1 B, Sig=230,4 Ref=360,100

Peak #	RetTime [min]	Type	Width [min]	Area [mAU*s]	Height [mAU]	Area %
1	4.730	BB	0.1497	1.00491e4	1013.71191	98.4468
2	6.758	BB	0.2001	158.54391	12.00241	1.5532

Totals :   1.02077e4 1025.71432

Signal 3: DAD1 C, Sig=254,4 Ref=360,100

Peak #	RetTime [min]	Type	Width [min]	Area [mAU*s]	Height [mAU]	Area %
1	4.730	BB	0.1494	5312.52637	536.86505	98.4563
2	6.758	BB	0.1995	83.29405	6.33232	1.5437

Totals :   5395.82042 543.19737

Signal 4: DAD1 D, Sig=280,4 Ref=360,100

Signal 5: DAD1 E, Sig=310,4 Ref=360,100

===

*** End of Report ***

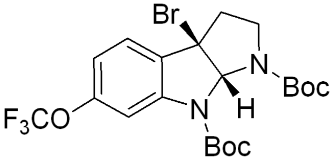

2o
(enantioenriched)

Supplementary Figure 134. HPLC Spectrum of **2o** (enantioenriched)

===

Acq. Operator   :   Seq. Line :   6
Acq. Instrument : Instrument 1   Location : Vial 7
Injection Date : 12/24/2021 12:17:31 AM   Inj :   1
                                                  Inj Volume : 5.000 µl
Different Inj Volume from Sequence !   Actual Inj Volume : 2.000 µl
Acq. Method   : C:\CHEM32\1\DATA\SUN_12 2021-12-23 22-38-16\IC-02-20.M
Last changed   : 12/23/2021 10:52:50 PM
                  (modified after loading)
Analysis Method : C:\CHEM32\1\METHODS\OD-03-60-0.6.M
Last changed   : 3/3/2022 9:54:51 PM
Additional Info : Peak(s) manually integrated

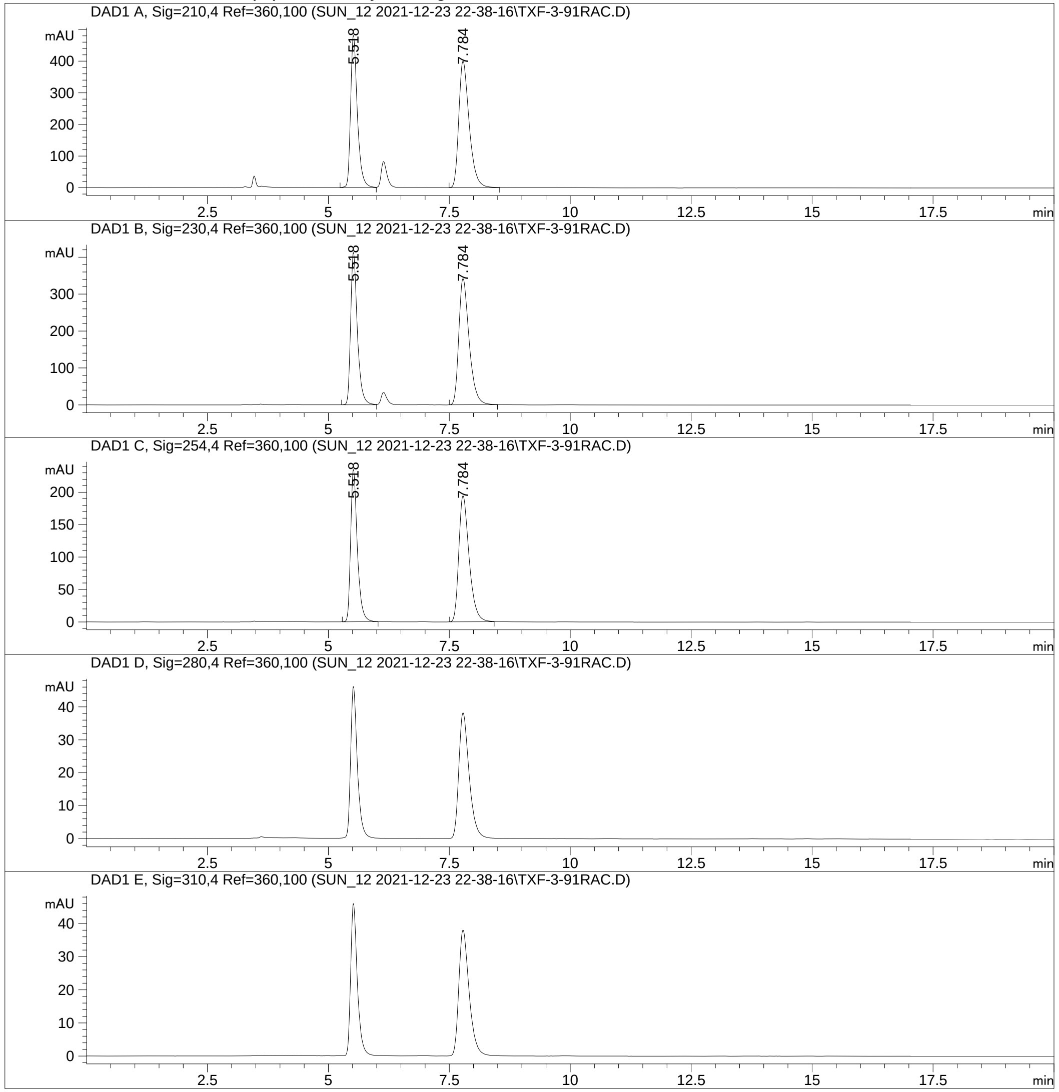

===

Area Percent Report

===

Sorted By   :   Signal
Multiplier   :   1.0000
Dilution   :   1.0000
Use Multiplier & Dilution Factor with ISTDs

Signal 1: DAD1 A, Sig=210,4 Ref=360,100

Peak #	RetTime [min]	Type	Width [min]	Area [mAU*s]	Height [mAU]	Area %
1	5.518	BV	0.1451	4670.24219	481.61826	43.8789
2	7.784	BB	0.2264	5973.24414	399.56943	56.1211

Totals :   1.06435e4   881.18768

Signal 2: DAD1 B, Sig=230,4 Ref=360,100

Peak #	RetTime [min]	Type	Width [min]	Area [mAU*s]	Height [mAU]	Area %
1	5.518	BV	0.1444	3980.50317	413.04346	43.8872
2	7.784	BB	0.2257	5089.35352	341.85913	56.1128

Totals :   9069.85669   754.90259

Signal 3: DAD1 C, Sig=254,4 Ref=360,100

Peak #	RetTime [min]	Type	Width [min]	Area [mAU*s]	Height [mAU]	Area %
1	5.518	BB	0.1439	2254.57837	235.09560	43.8555
2	7.784	BB	0.2252	2886.34595	194.37715	56.1445

Totals :   5140.92432   429.47275

Signal 4: DAD1 D, Sig=280,4 Ref=360,100

Signal 5: DAD1 E, Sig=310,4 Ref=360,100

===

*** End of Report ***

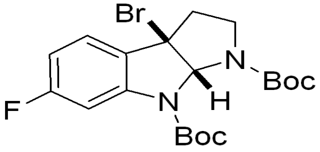

2p
(racemic)

Supplementary Figure 135. HPLC Spectrum of **2p** (racemic)

Data File C:\CHEM32\1\DATA\SUN_12 2021-12-23 22-38-16\TXF-3-91.D
Sample Name:

===

Acq. Operator : Seq. Line : 7
Acq. Instrument : Instrument 1 Location : Vial 8
Injection Date : 12/24/2021 12:38:29 AM Inj : 1
Inj Volume : 5.000 µl
Different Inj Volume from Sequence ! Actual Inj Volume : 2.000 µl
Acq. Method : C:\CHEM32\1\DATA\SUN_12 2021-12-23 22-38-16\IC-02-20.M
Last changed : 12/23/2021 10:52:50 PM
(modified after loading)
Analysis Method : C:\CHEM32\1\METHODS\OD-03-60-0.6.M
Last changed : 3/3/2022 9:54:51 PM
Additional Info : Peak(s) manually integrated

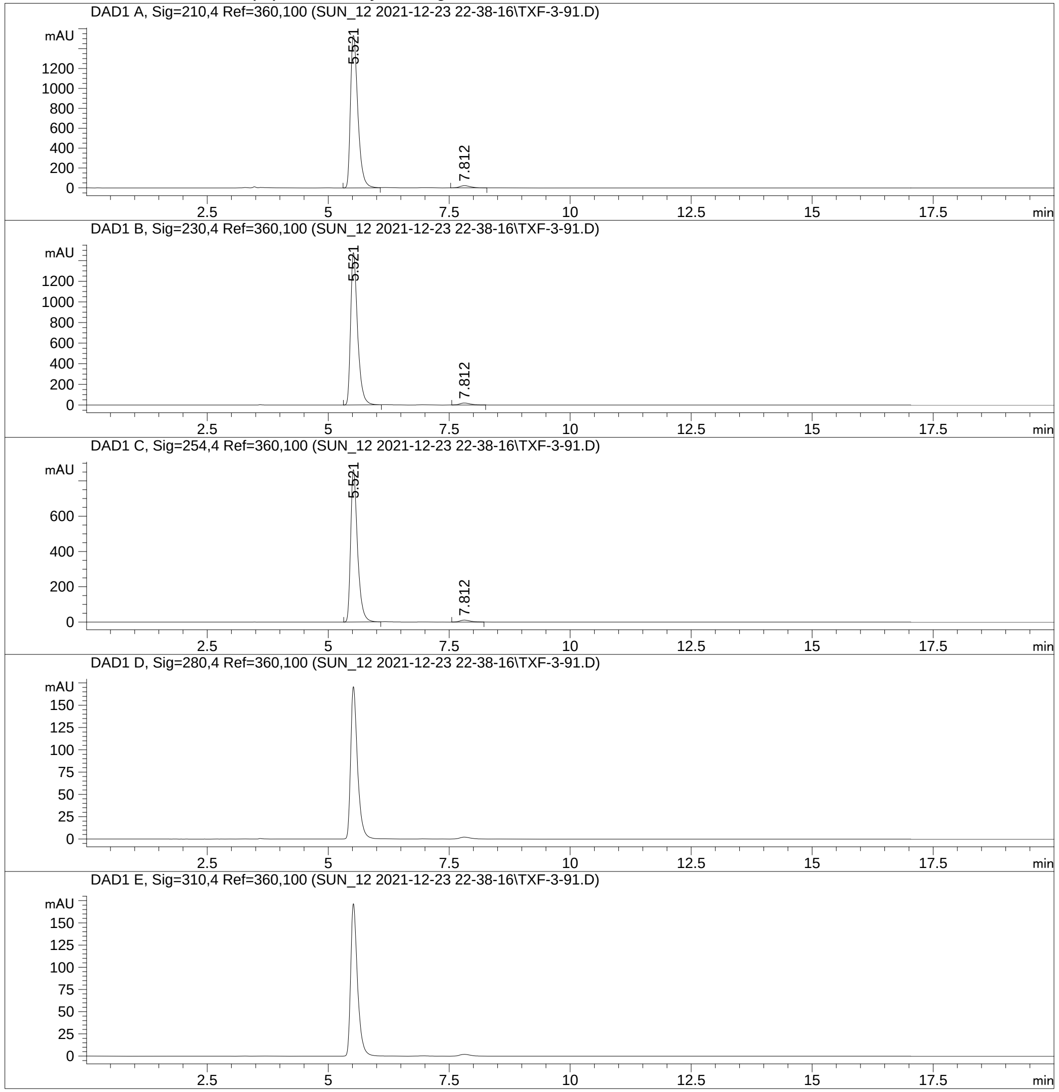

===
Area Percent Report
===

Sorted By : Signal
Multiplier : 1.0000
Dilution : 1.0000
Use Multiplier & Dilution Factor with ISTDs

Signal 1: DAD1 A, Sig=210,4 Ref=360,100

Peak #	RetTime [min]	Type	Width [min]	Area [mAU*s]	Height [mAU]	Area %
1	5.521	BV	0.1634	1.62131e4	1532.92395	97.8548
2	7.812	BB	0.2340	355.42789	23.03656	2.1452

Totals : 1.65685e4 1555.96051

Signal 2: DAD1 B, Sig=230,4 Ref=360,100

Peak #	RetTime [min]	Type	Width [min]	Area [mAU*s]	Height [mAU]	Area %
1	5.521	BB	0.1496	1.46552e4	1478.45435	98.0162
2	7.812	BB	0.2317	296.61581	19.47819	1.9838

Totals : 1.49518e4 1497.93253

Signal 3: DAD1 C, Sig=254,4 Ref=360,100

Peak #	RetTime [min]	Type	Width [min]	Area [mAU*s]	Height [mAU]	Area %
1	5.521	BB	0.1482	8457.19824	864.08807	98.0769
2	7.812	BB	0.2303	165.83253	10.97355	1.9231

Totals : 8623.03078 875.06162

Signal 4: DAD1 D, Sig=280,4 Ref=360,100

Signal 5: DAD1 E, Sig=310,4 Ref=360,100

===
*** End of Report ***

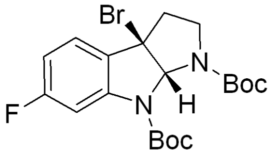

2p
(enantioenriched)

Supplementary Figure 136. HPLC Spectrum of **2p** (enantioenriched)

```
=====================================================================
Acq. Operator   :                               Seq. Line :   7
Acq. Instrument : Instrument 1                   Location : Vial 3
Injection Date  : 12/29/2021 11:14:06 PM              Inj :   1
                                               Inj Volume : 5.000 µl
Different Inj Volume from Sequence !     Actual Inj Volume : 2.000 µl
Acq. Method     : C:\CHEM32\1\DATA\SUN_12 2021-12-29 21-33-13\IC-02-20.M
Last changed    : 12/29/2021 11:13:14 PM
                  (modified after loading)
Analysis Method : C:\CHEM32\1\METHODS\OD-03-60-0.6.M
Last changed    : 3/3/2022 9:54:51 PM
Additional Info : Peak(s) manually integrated
```

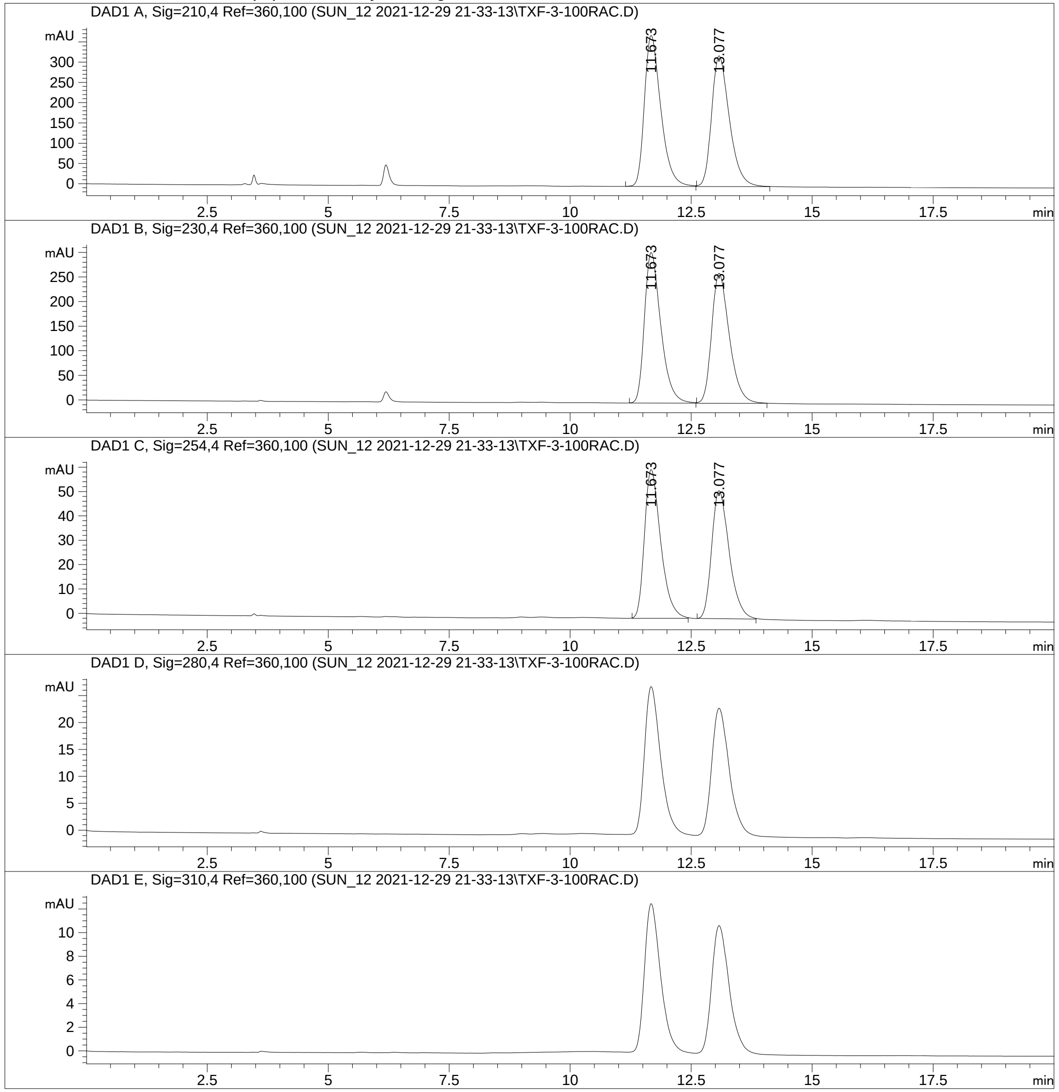

Data File C:\CHEM32\1\DATA\SUN_12 2021-12-29 21-33-13\TXF-3-100RAC.D
Sample Name:

```
=====================================================================
                         Area Percent Report
=====================================================================

Sorted By             :      Signal
Multiplier            :      1.0000
Dilution              :      1.0000
Use Multiplier & Dilution Factor with ISTDs
```

Signal 1: DAD1 A, Sig=210,4 Ref=360,100

Peak #	RetTime [min]	Type	Width [min]	Area [mAU*s]	Height [mAU]	Area %
1	11.673	BB	0.3602	8820.18555	372.55942	51.5794
2	13.077	BB	0.3935	8280.01172	322.51837	48.4206
Totals :				1.71002e4	695.07779	

Signal 2: DAD1 B, Sig=230,4 Ref=360,100

Peak #	RetTime [min]	Type	Width [min]	Area [mAU*s]	Height [mAU]	Area %
1	11.673	BB	0.3588	7212.27686	306.20602	51.6814
2	13.077	BB	0.3915	6742.97705	264.40631	48.3186
Totals :				1.39553e4	570.61234	

Signal 3: DAD1 C, Sig=254,4 Ref=360,100

Peak #	RetTime [min]	Type	Width [min]	Area [mAU*s]	Height [mAU]	Area %
1	11.673	BB	0.3564	1426.16736	61.07103	51.9036
2	13.077	BB	0.3874	1321.55688	52.55351	48.0964
Totals :				2747.72424	113.62454	

Signal 4: DAD1 D, Sig=280,4 Ref=360,100

Signal 5: DAD1 E, Sig=310,4 Ref=360,100

```
=====================================================================
                        *** End of Report ***
```

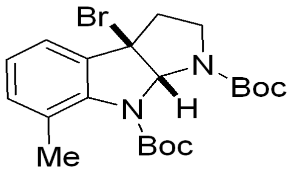

2q
(racemic)

Supplementary Figure 137. HPLC Spectrum of **2q** (racemic)

```
=====================================================================
Acq. Operator   :                               Seq. Line :   8
Acq. Instrument : Instrument 1                   Location : Vial 4
Injection Date  : 12/29/2021 11:35:04 PM              Inj :   1
                                               Inj Volume : 5.000 µl
Different Inj Volume from Sequence !     Actual Inj Volume : 2.000 µl
Acq. Method     : C:\CHEM32\1\DATA\SUN_12 2021-12-29 21-33-13\IC-02-20.M
Last changed    : 12/29/2021 11:13:14 PM
                  (modified after loading)
Analysis Method : C:\CHEM32\1\METHODS\OD-03-60-0.6.M
Last changed    : 3/3/2022 9:54:51 PM
Additional Info : Peak(s) manually integrated
```

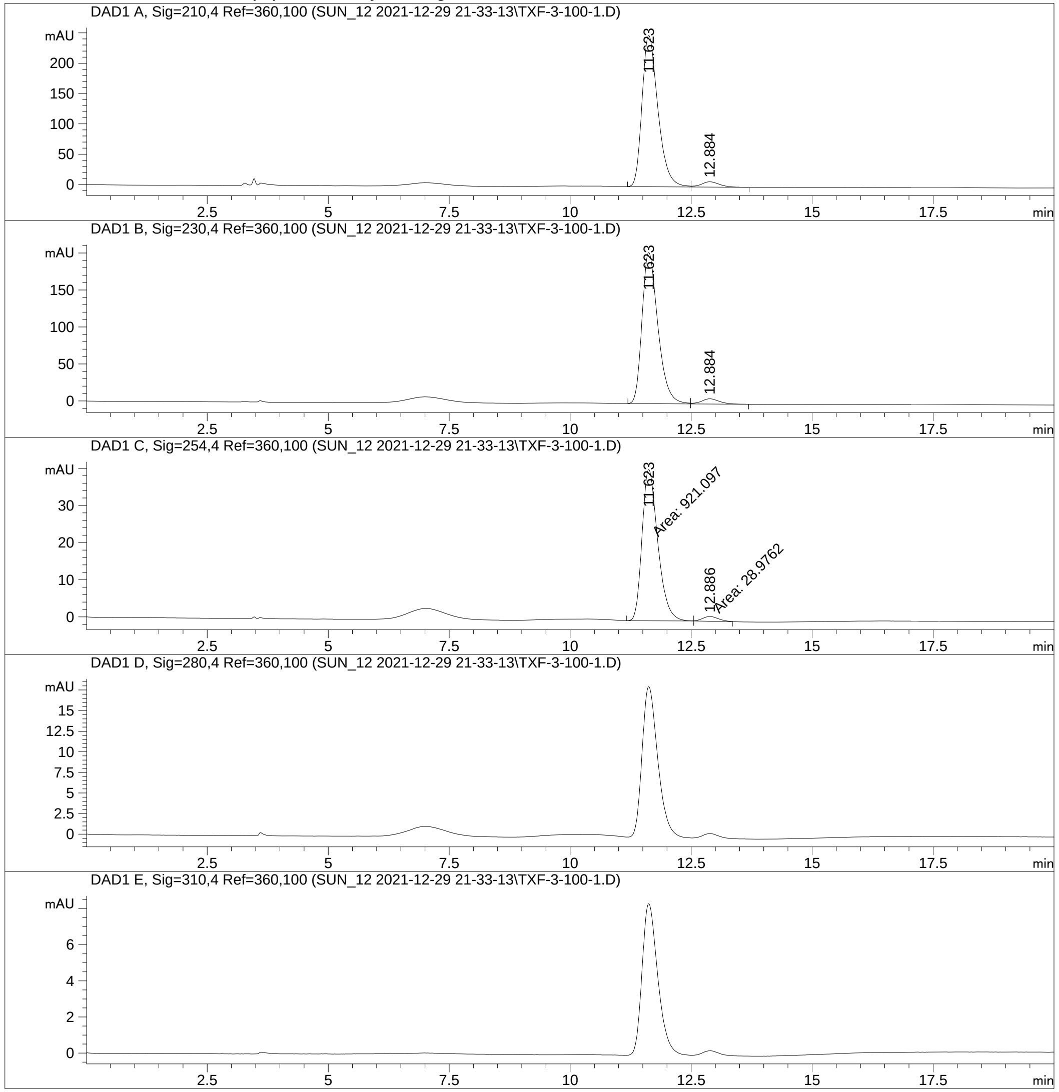

Data File C:\CHEM32\1\DATA\SUN_12 2021-12-29 21-33-13\TXF-3-100-1.D
Sample Name:

```
=====================================================================
                         Area Percent Report
=====================================================================

Sorted By             :      Signal
Multiplier            :      1.0000
Dilution              :      1.0000
Use Multiplier & Dilution Factor with ISTDs


Signal 1: DAD1 A, Sig=210,4 Ref=360,100

Peak RetTime Type  Width     Area      Height     Area
  #   [min]        [min]   [mAU*s]     [mAU]        %
----|-------|----|-------|----------|----------|--------|
   1  11.623 BB    0.3487 5700.69531  249.41661  96.1688
   2  12.884 BB    0.3892  227.10699    8.79596   3.8312

Totals :                  5927.80231  258.21257


Signal 2: DAD1 B, Sig=230,4 Ref=360,100

Peak RetTime Type  Width     Area      Height     Area
  #   [min]        [min]   [mAU*s]     [mAU]        %
----|-------|----|-------|----------|----------|--------|
   1  11.623 BB    0.3481 4663.06982  204.43134  96.1935
   2  12.884 BB    0.3864  184.52580    7.11795   3.8065

Totals :                  4847.59563  211.54929


Signal 3: DAD1 C, Sig=254,4 Ref=360,100

Peak RetTime Type  Width     Area      Height     Area
  #   [min]        [min]   [mAU*s]     [mAU]        %
----|-------|----|-------|----------|----------|--------|
   1  11.623 MM    0.3770  921.09668   40.71619  96.9501
   2  12.886 MM    0.3743   28.97620    1.29024   3.0499

Totals :                   950.07288   42.00643


Signal 4: DAD1 D, Sig=280,4 Ref=360,100


Signal 5: DAD1 E, Sig=310,4 Ref=360,100

=====================================================================
                          *** End of Report ***
```

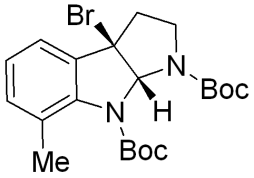

2q
(enantioenriched)

Supplementary Figure 138. HPLC Spectrum of **2q** (enantioenriched)

```
Data File C:\CHEM32\1\DATA\SUN_12 2021-12-29 21-33-13\TXF-3-101RAC.D
Sample Name:

    =====================================================================
    Acq. Operator   :                                 Seq. Line :   9
    Acq. Instrument : Instrument 1                     Location : Vial 5
    Injection Date  : 12/29/2021 11:56:03 PM                Inj :   1
                                                     Inj Volume : 5.000 µl
    Different Inj Volume from Sequence !     Actual Inj Volume : 2.000 µl
    Acq. Method     : C:\CHEM32\1\DATA\SUN_12 2021-12-29 21-33-13\IC-02-20.M
    Last changed    : 12/29/2021 11:13:14 PM
                      (modified after loading)
    Analysis Method : C:\CHEM32\1\METHODS\OD-03-60-0.6.M
    Last changed    : 3/3/2022 9:54:51 PM
    Additional Info : Peak(s) manually integrated
```

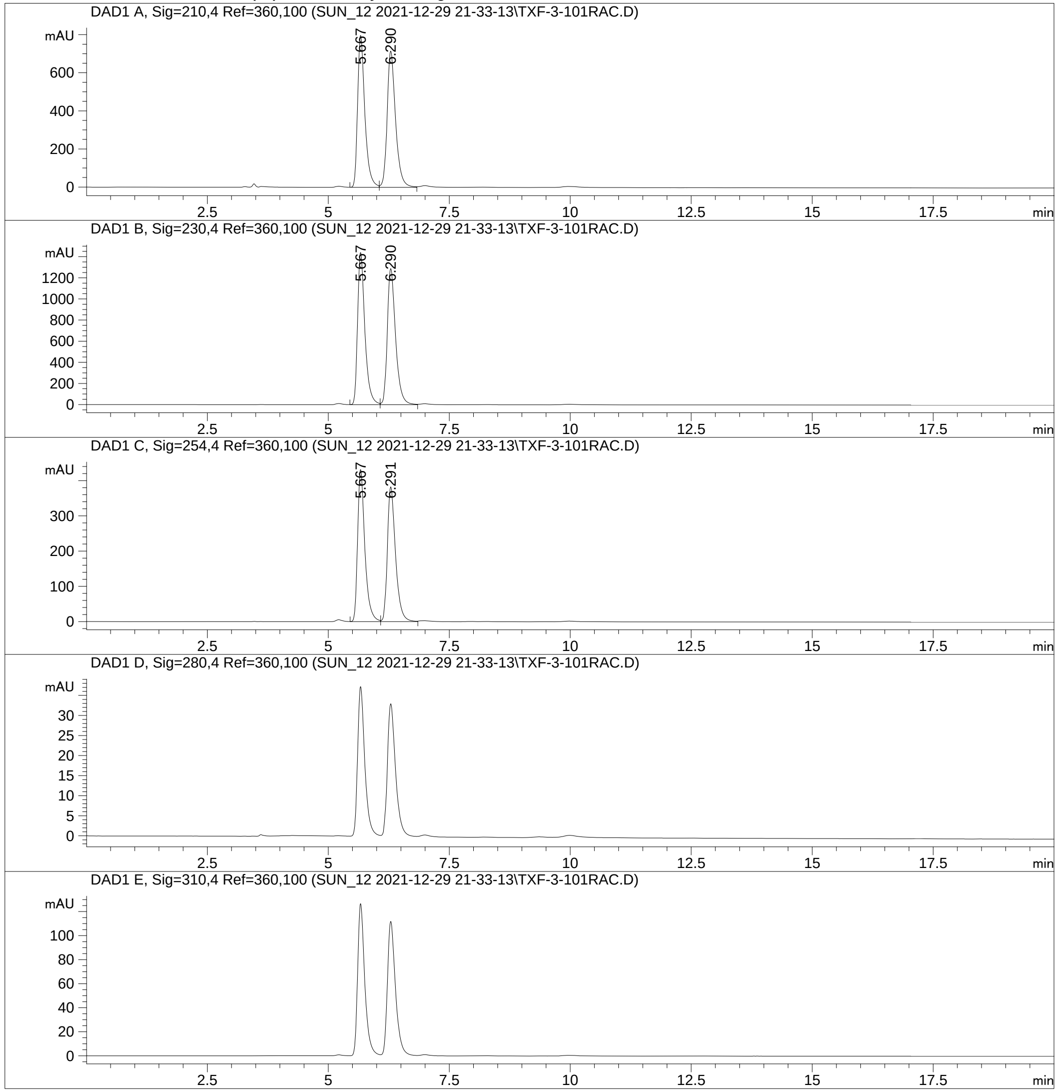


```
Data File C:\CHEM32\1\DATA\SUN_12 2021-12-29 21-33-13\TXF-3-101RAC.D
Sample Name:

    =====================================================================
                             Area Percent Report
    =====================================================================

    Sorted By             :      Signal
    Multiplier            :      1.0000
    Dilution              :      1.0000
    Use Multiplier & Dilution Factor with ISTDs


    Signal 1: DAD1 A, Sig=210,4 Ref=360,100

    Peak RetTime Type  Width     Area      Height     Area
      #   [min]        [min]   [mAU*s]     [mAU]        %
    ----|-------|----|-------|----------|----------|--------|
       1   5.667 VV    0.1572 8289.16016  797.79279  49.0304
       2   6.290 VV    0.1826 8616.98828  715.01038  50.9696

    Totals :                  1.69061e4  1512.80316


    Signal 2: DAD1 B, Sig=230,4 Ref=360,100

    Peak RetTime Type  Width     Area      Height     Area
      #   [min]        [min]   [mAU*s]     [mAU]        %
    ----|-------|----|-------|----------|----------|--------|
       1   5.667 VV    0.1579 1.50667e4  1441.80676  49.6481
       2   6.290 VV    0.1803 1.52803e4  1289.01746  50.3519

    Totals :                  3.03469e4  2730.82422


    Signal 3: DAD1 C, Sig=254,4 Ref=360,100

    Peak RetTime Type  Width     Area      Height     Area
      #   [min]        [min]   [mAU*s]     [mAU]        %
    ----|-------|----|-------|----------|----------|--------|
       1   5.667 VV    0.1572 4488.55908  432.07144  50.0555
       2   6.291 VB    0.1786 4478.61230  382.51825  49.9445

    Totals :                  8967.17139  814.58969


    Signal 4: DAD1 D, Sig=280,4 Ref=360,100


    Signal 5: DAD1 E, Sig=310,4 Ref=360,100

    =====================================================================
                             *** End of Report ***
```

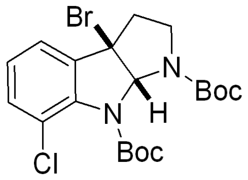

2r
(racemic)

Supplementary Figure 139. HPLC Spectrum of **2r** (racemic)

Data File C:\CHEM32\1\DATA\SUN_12 2021-12-29 21-33-13\TXF-3-101-1.D
Sample Name:

===

Acq. Operator : Seq. Line : 10
Acq. Instrument : Instrument 1 Location : Vial 6
Injection Date : 12/30/2021 12:17:02 AM Inj : 1
Inj Volume : 5.000 µl
Different Inj Volume from Sequence ! Actual Inj Volume : 2.000 µl
Acq. Method : C:\CHEM32\1\DATA\SUN_12 2021-12-29 21-33-13\IC-02-20.M
Last changed : 12/29/2021 11:13:14 PM
(modified after loading)
Analysis Method : C:\CHEM32\1\METHODS\OD-03-60-0.6.M
Last changed : 3/3/2022 9:54:51 PM
Additional Info : Peak(s) manually integrated

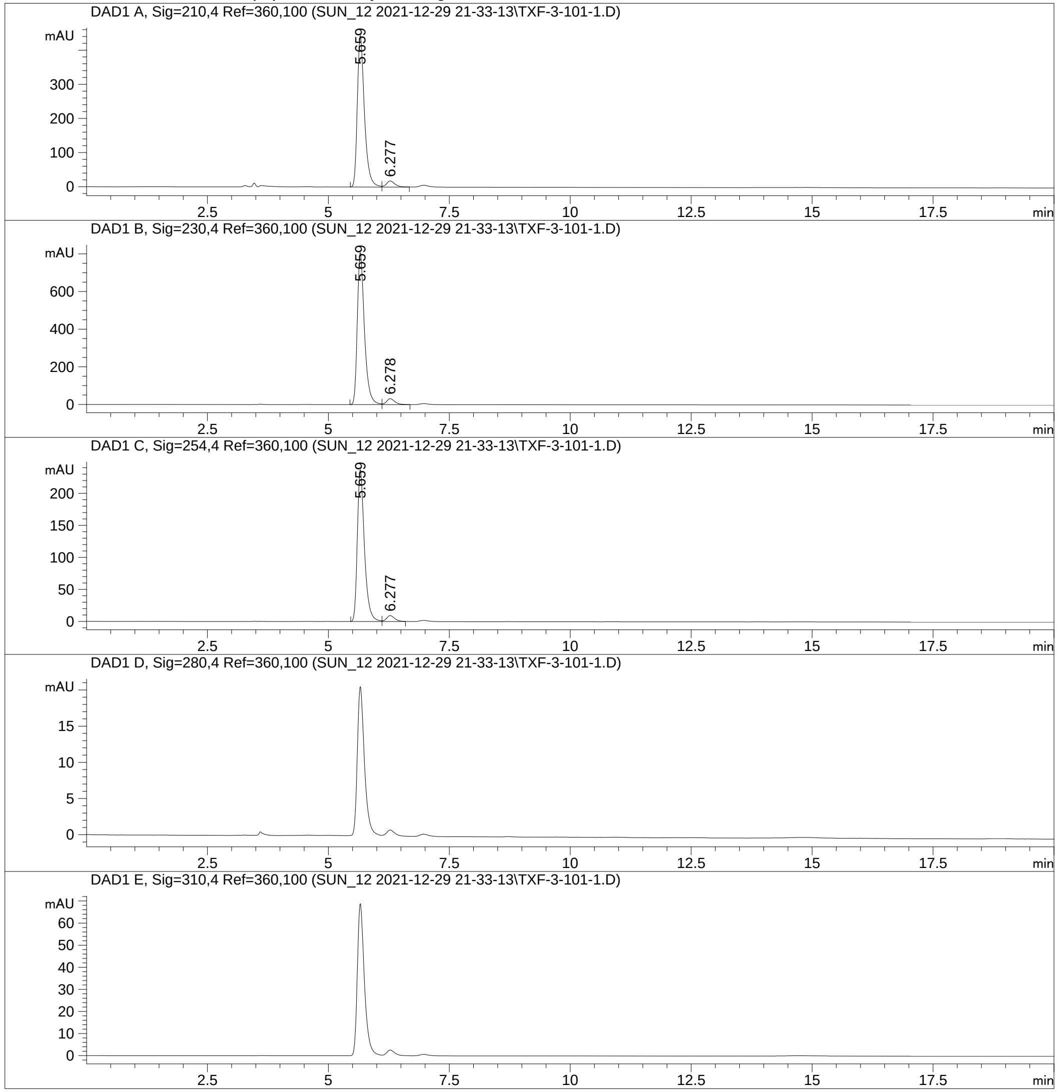

Data File C:\CHEM32\1\DATA\SUN_12 2021-12-29 21-33-13\TXF-3-101-1.D
Sample Name:

===

Area Percent Report

===

Sorted By : Signal
Multiplier : 1.0000
Dilution : 1.0000
Use Multiplier & Dilution Factor with ISTDs

Signal 1: DAD1 A, Sig=210,4 Ref=360,100

Peak #	RetTime [min]	Type	Width [min]	Area [mAU*s]	Height [mAU]	Area %
1	5.659	BV	0.1549	4527.64111	444.07684	95.3176
2	6.277	VB	0.1831	222.41728	18.12750	4.6824

Totals : 4750.05840 462.20435

Signal 2: DAD1 B, Sig=230,4 Ref=360,100

Peak #	RetTime [min]	Type	Width [min]	Area [mAU*s]	Height [mAU]	Area %
1	5.659	BV	0.1547	8228.82715	808.63873	95.5783
2	6.278	VB	0.1793	380.68976	31.88699	4.4217

Totals : 8609.51691 840.52572

Signal 3: DAD1 C, Sig=254,4 Ref=360,100

Peak #	RetTime [min]	Type	Width [min]	Area [mAU*s]	Height [mAU]	Area %
1	5.659	BV	0.1545	2413.56421	237.56018	95.6404
2	6.277	VB	0.1767	110.01810	9.38742	4.3596

Totals : 2523.58231 246.94760

Signal 4: DAD1 D, Sig=280,4 Ref=360,100

Signal 5: DAD1 E, Sig=310,4 Ref=360,100

===

*** End of Report ***

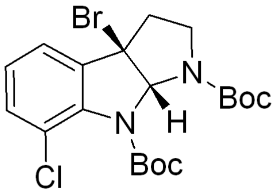

2r
(enantioenriched)

Supplementary Figure 140. HPLC Spectrum of **2r** (enantioenriched)

Data File C:\CHEM32\1\DATA\SUN_12 2022-02-16 10-19-46\TXF-3-113-RAC.D
Sample Name:

```
=====================================================================
Acq. Operator   :                                 Seq. Line :   2
Acq. Instrument : Instrument 1                     Location : Vial 8
Injection Date  : 2/16/2022 10:32:39 AM                 Inj :   1
                                                 Inj Volume : 5.000 µl
Different Inj Volume from Sequence !     Actual Inj Volume : 2.000 µl
Acq. Method     : C:\CHEM32\1\DATA\SUN_12 2022-02-16 10-19-46\IC-05-20.M
Last changed    : 2/16/2022 10:44:07 AM
                  (modified after loading)
Analysis Method : C:\CHEM32\1\METHODS\OD-03-60-0.6.M
Last changed    : 3/3/2022 9:54:51 PM
Additional Info : Peak(s) manually integrated
```

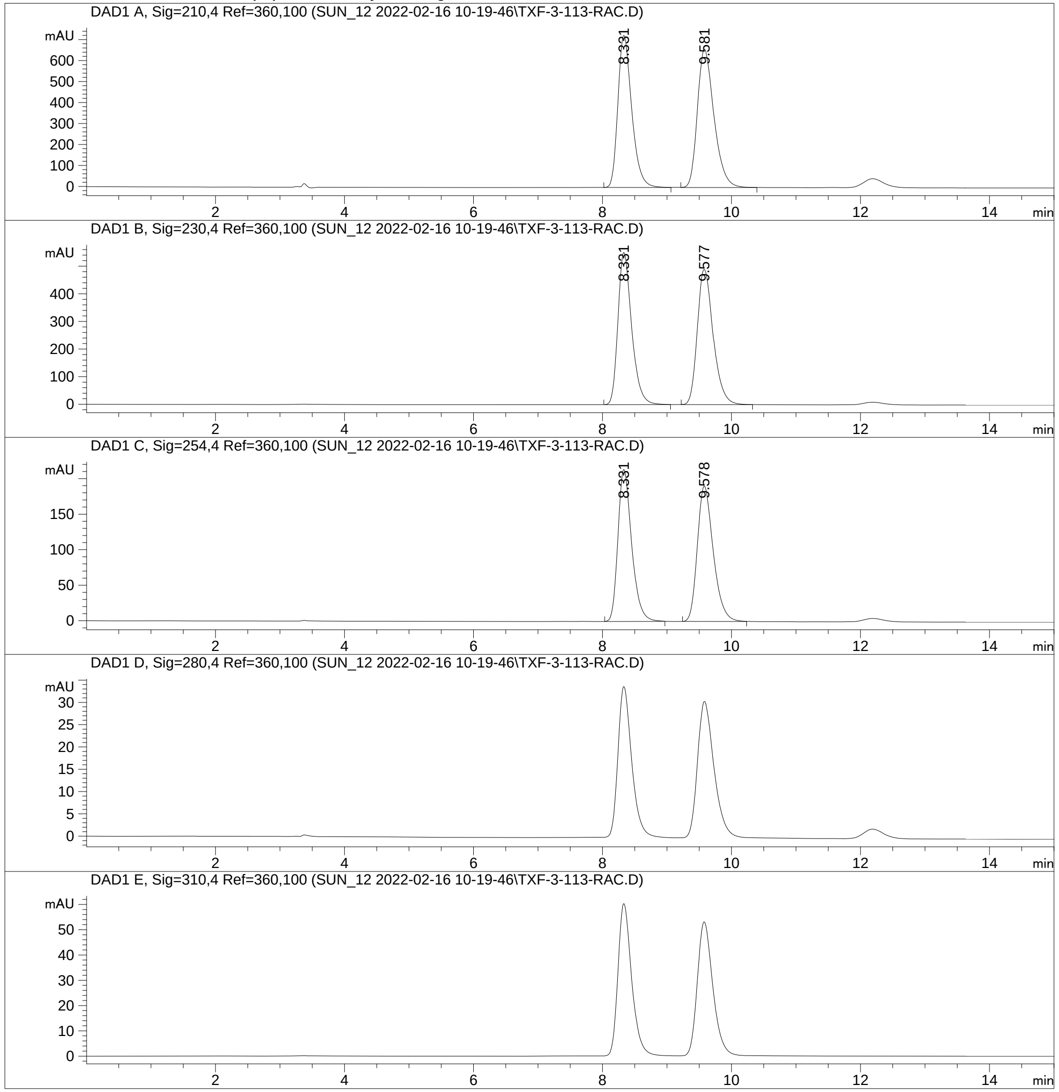

Data File C:\CHEM32\1\DATA\SUN_12 2022-02-16 10-19-46\TXF-3-113-RAC.D
Sample Name:

Area Percent Report

```
Sorted By             :      Signal
Multiplier            :      1.0000
Dilution              :      1.0000
Use Multiplier & Dilution Factor with ISTDs
```

Signal 1: DAD1 A, Sig=210,4 Ref=360,100

Peak #	RetTime [min]	Type	Width [min]	Area [mAU*s]	Height [mAU]	Area %
1	8.331	BB	0.2300	1.08135e4	724.96954	47.8129
2	9.581	BB	0.2747	1.18027e4	659.74951	52.1871

Totals : 2.26162e4 1384.71906

Signal 2: DAD1 B, Sig=230,4 Ref=360,100

Peak #	RetTime [min]	Type	Width [min]	Area [mAU*s]	Height [mAU]	Area %
1	8.331	BB	0.2244	8144.30811	551.29608	49.3374
2	9.577	BB	0.2610	8363.07813	490.22809	50.6626

Totals : 1.65074e4 1041.52417

Signal 3: DAD1 C, Sig=254,4 Ref=360,100

Peak #	RetTime [min]	Type	Width [min]	Area [mAU*s]	Height [mAU]	Area %
1	8.331	BB	0.2246	3163.37573	213.82677	49.2095
2	9.578	BB	0.2620	3265.01270	190.43779	50.7905

Totals : 6428.38843 404.26456

Signal 4: DAD1 D, Sig=280,4 Ref=360,100

Signal 5: DAD1 E, Sig=310,4 Ref=360,100

*** End of Report ***

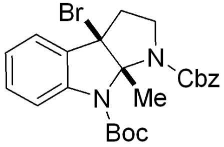

2s
(racemic)

Supplementary Figure 141. HPLC Spectrum of **2s** (racemic)

Data File C:\CHEM32\1\DATA\SUN_12 2022-02-16 10-19-46\TXF-3-113-5.D
Sample Name:

===

```
Acq. Operator   :                                Seq. Line :    3
Acq. Instrument : Instrument 1                    Location : Vial 9
Injection Date  : 2/16/2022 10:48:39 AM                Inj :    1
                                                Inj Volume : 5.000 µl
Different Inj Volume from Sequence !     Actual Inj Volume : 2.000 µl
Acq. Method     : C:\CHEM32\1\DATA\SUN_12 2022-02-16 10-19-46\IC-05-20.M
Last changed    : 2/16/2022 10:44:07 AM
                  (modified after loading)
Analysis Method : C:\CHEM32\1\METHODS\OD-03-60-0.6.M
Last changed    : 3/3/2022 9:54:51 PM
Additional Info : Peak(s) manually integrated
```

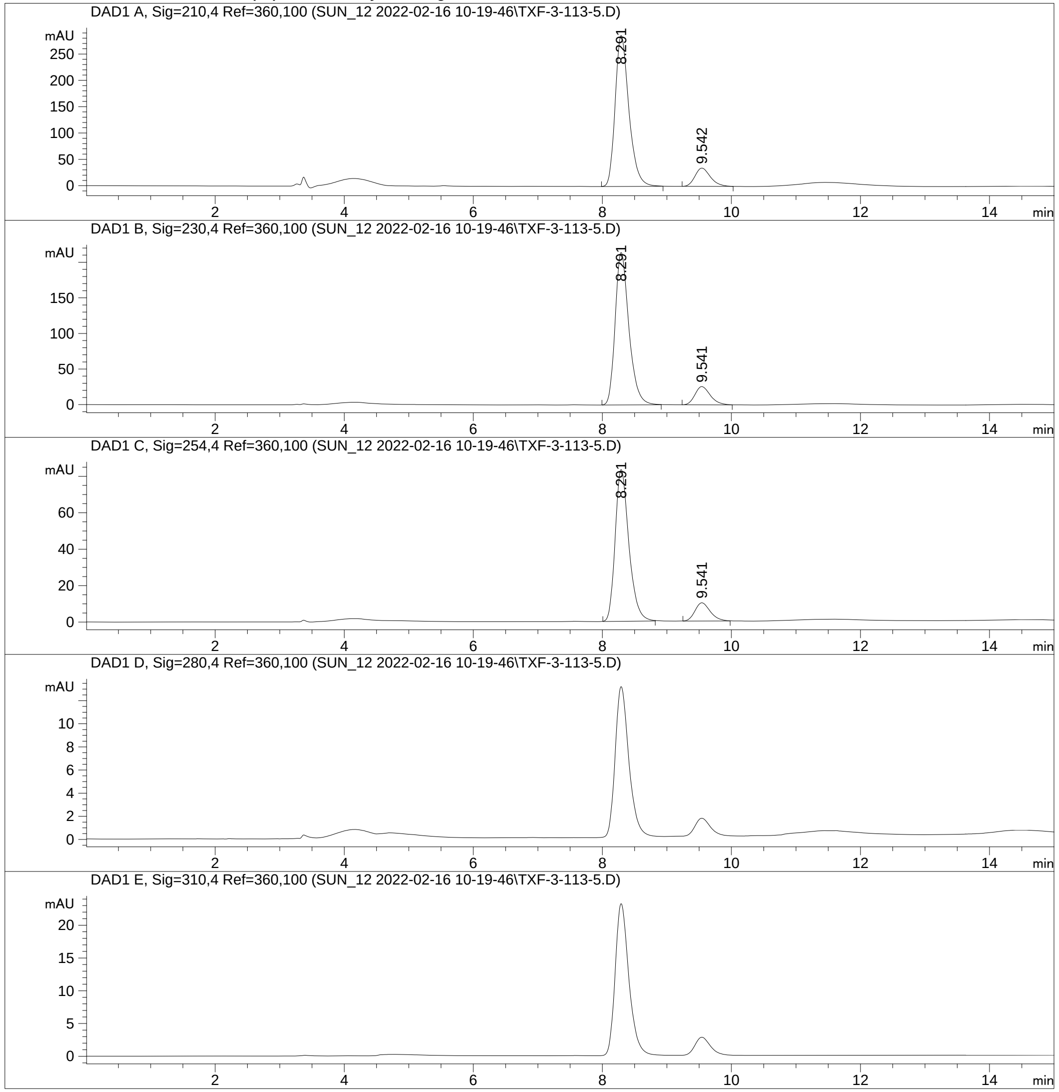

Data File C:\CHEM32\1\DATA\SUN_12 2022-02-16 10-19-46\TXF-3-113-5.D
Sample Name:

===

Area Percent Report

===

```
Sorted By             :      Signal
Multiplier            :      1.0000
Dilution              :      1.0000
Use Multiplier & Dilution Factor with ISTDs
```

Signal 1: DAD1 A, Sig=210,4 Ref=360,100

Peak #	RetTime [min]	Type	Width [min]	Area [mAU*s]	Height [mAU]	Area %
1	8.291	BB	0.2247	4243.31445	286.66757	87.9912
2	9.542	BB	0.2584	579.11719	34.75763	12.0088

Totals : 4822.43164 321.42521

Signal 2: DAD1 B, Sig=230,4 Ref=360,100

Peak #	RetTime [min]	Type	Width [min]	Area [mAU*s]	Height [mAU]	Area %
1	8.291	BB	0.2244	3164.15918	214.15660	88.1259
2	9.541	BB	0.2555	426.33990	25.70360	11.8741

Totals : 3590.49908 239.86020

Signal 3: DAD1 C, Sig=254,4 Ref=360,100

Peak #	RetTime [min]	Type	Width [min]	Area [mAU*s]	Height [mAU]	Area %
1	8.291	BB	0.2241	1228.64771	83.28817	88.1370
2	9.541	BB	0.2549	165.37294	10.00154	11.8630

Totals : 1394.02065 93.28971

Signal 4: DAD1 D, Sig=280,4 Ref=360,100

Signal 5: DAD1 E, Sig=310,4 Ref=360,100

===

*** End of Report ***

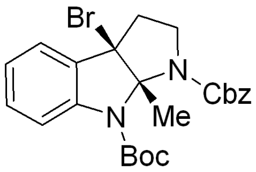

2s
(enantioenriched)

Supplementary Figure 142. HPLC Spectrum of **2s** (enantioenriched)

```
=================================================================
Acq. Operator   :                        Seq. Line :   3
Acq. Instrument : Instrument 1            Location : Vial 2
Injection Date  : 1/3/2022 8:46:27 PM          Inj :   1
                                        Inj Volume : 5.000 µl
Different Inj Volume from Sequence !   Actual Inj Volume : 2.000 µl
Acq. Method     : C:\CHEM32\1\DATA\SUN_12 2022-01-03 20-12-43\IC-03-20.M
Last changed    : 1/3/2022 8:24:39 PM
                  (modified after loading)
Analysis Method : C:\CHEM32\1\METHODS\OD-03-60-0.6.M
Last changed    : 3/3/2022 9:54:51 PM
Additional Info : Peak(s) manually integrated
```

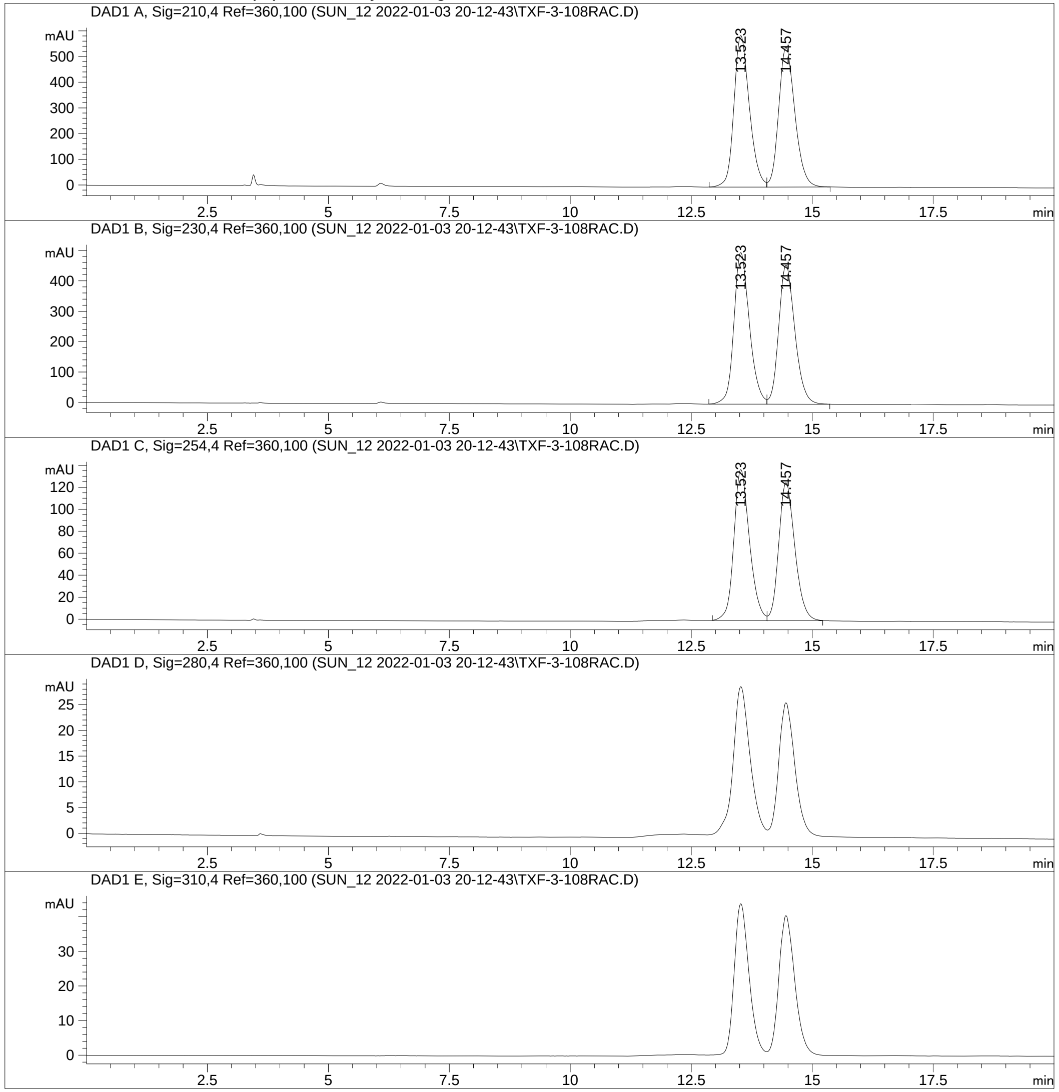

Data File C:\CHEM32\1\DATA\SUN_12 2022-01-03 20-12-43\TXF-3-108RAC.D
Sample Name:

```
=================================================================
                     Area Percent Report
=================================================================

Sorted By            :      Signal
Multiplier           :      1.0000
Dilution             :      1.0000
Use Multiplier & Dilution Factor with ISTDs


Signal 1: DAD1 A, Sig=210,4 Ref=360,100

Peak RetTime Type  Width     Area      Height     Area
  #   [min]        [min]   [mAU*s]     [mAU]        %
----|-------|----|-------|----------|----------|--------|
   1  13.523 BV    0.3397 1.31335e4   590.01941  50.8216
   2  14.457 VB    0.3589 1.27089e4   543.25238  49.1784

Totals :                  2.58423e4  1133.27179


Signal 2: DAD1 B, Sig=230,4 Ref=360,100

Peak RetTime Type  Width     Area      Height     Area
  #   [min]        [min]   [mAU*s]     [mAU]        %
----|-------|----|-------|----------|----------|--------|
   1  13.523 BV    0.3389 1.11598e4   499.10568  51.1856
   2  14.457 VB    0.3575 1.06428e4   457.39017  48.8144

Totals :                  2.18025e4   956.49585


Signal 3: DAD1 C, Sig=254,4 Ref=360,100

Peak RetTime Type  Width     Area      Height     Area
  #   [min]        [min]   [mAU*s]     [mAU]        %
----|-------|----|-------|----------|----------|--------|
   1  13.523 BV    0.3440 3108.21606  137.38234  51.5560
   2  14.457 VB    0.3575 2920.60278  125.49256  48.4440

Totals :                  6028.81885  262.87490


Signal 4: DAD1 D, Sig=280,4 Ref=360,100


Signal 5: DAD1 E, Sig=310,4 Ref=360,100

=================================================================
                    *** End of Report ***
```

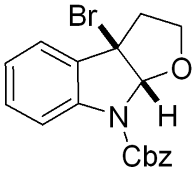

2t
(racemic)

Supplementary Figure 143. HPLC Spectrum of **2t** (racemic)

```
=====================================================================
Acq. Operator   :                                 Seq. Line :   2
Acq. Instrument : Instrument 1                     Location : Vial 1
Injection Date  : 1/3/2022 8:25:31 PM                   Inj :   1
                                                 Inj Volume : 5.000 µl
Different Inj Volume from Sequence !     Actual Inj Volume : 2.000 µl
Acq. Method     : C:\CHEM32\1\DATA\SUN_12 2022-01-03 20-12-43\IC-03-20.M
Last changed    : 1/3/2022 8:24:39 PM
                  (modified after loading)
Analysis Method : C:\CHEM32\1\METHODS\OD-03-60-0.6.M
Last changed    : 3/3/2022 9:54:51 PM
Additional Info : Peak(s) manually integrated
```

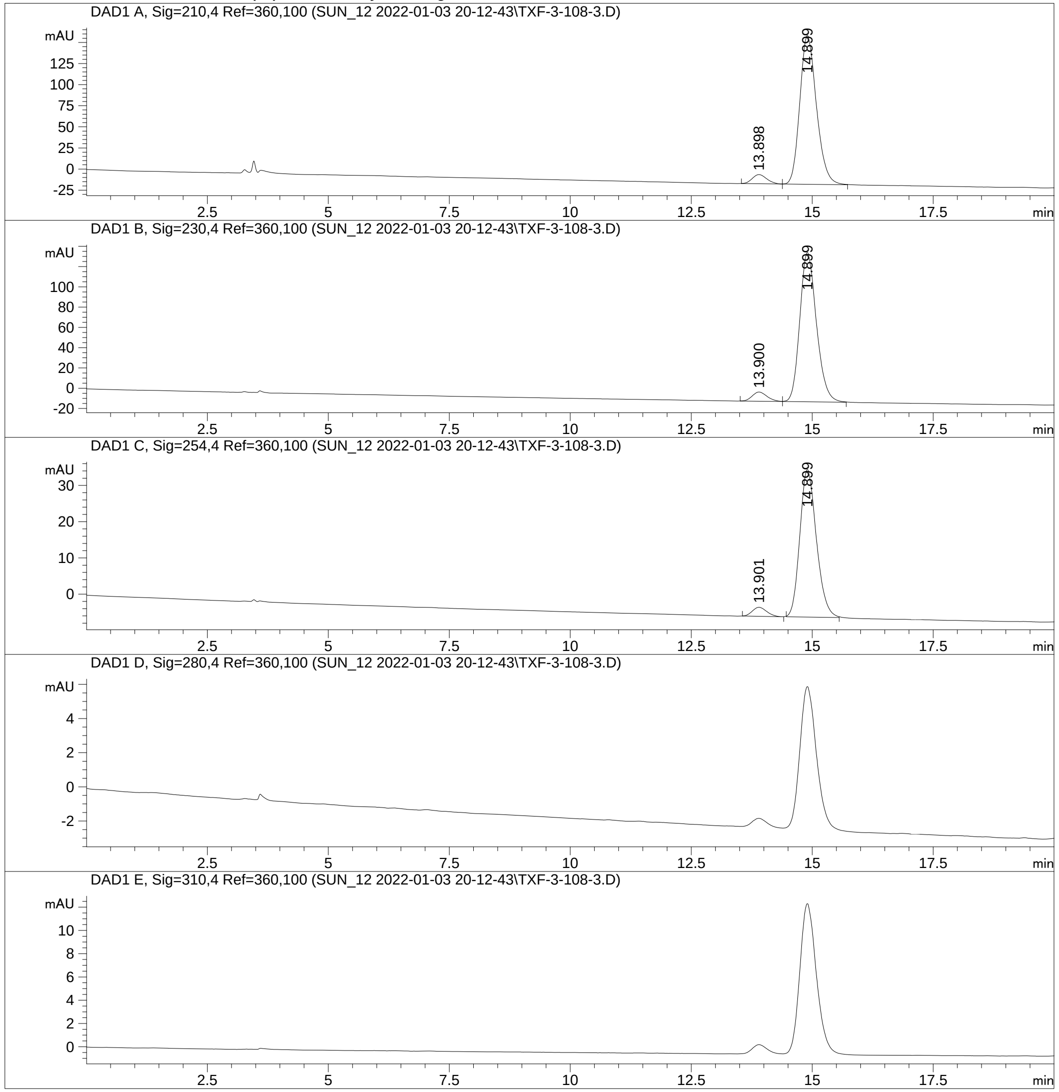

Area Percent Report

Sorted By : Signal
Multiplier : 1.0000
Dilution : 1.0000
Use Multiplier & Dilution Factor with ISTDs

Signal 1: DAD1 A, Sig=210,4 Ref=360,100

Peak #	RetTime [min]	Type	Width [min]	Area [mAU*s]	Height [mAU]	Area %
1	13.898	BV	0.3177	226.96394	10.86349	5.1086
2	14.899	VB	0.3689	4215.80908	176.33023	94.8914

Totals : 4442.77303 187.19372

Signal 2: DAD1 B, Sig=230,4 Ref=360,100

Peak #	RetTime [min]	Type	Width [min]	Area [mAU*s]	Height [mAU]	Area %
1	13.900	BV	0.3307	193.97423	9.09783	5.2224
2	14.899	VB	0.3668	3520.30029	147.29567	94.7776

Totals : 3714.27452 156.39350

Signal 3: DAD1 C, Sig=254,4 Ref=360,100

Peak #	RetTime [min]	Type	Width [min]	Area [mAU*s]	Height [mAU]	Area %
1	13.901	BB	0.3155	51.05390	2.48662	5.0082
2	14.899	BB	0.3655	968.35199	40.70721	94.9918

Totals : 1019.40589 43.19382

Signal 4: DAD1 D, Sig=280,4 Ref=360,100

Signal 5: DAD1 E, Sig=310,4 Ref=360,100

*** End of Report ***

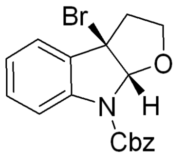

2t
(enantioenriched)

Supplementary Figure 144. HPLC Spectrum of **2t** (enantioenriched)

Data File C:\CHEM32\1\DATA\SUN_12 2022-01-07 14-13-00\TXF-2-118-RAC-RE.D
Sample Name:

```
=====================================================================
Acq. Operator   :                              Seq. Line :   3
Acq. Instrument : Instrument 1                  Location : Vial 1
Injection Date  : 1/7/2022 2:42:46 PM                Inj :   1
                                              Inj Volume : 5.000 µl
Different Inj Volume from Sequence !     Actual Inj Volume : 2.000 µl
Acq. Method     : C:\CHEM32\1\DATA\SUN_12 2022-01-07 14-13-00\OD-05-20.M
Last changed    : 1/7/2022 2:52:52 PM
                  (modified after loading)
Analysis Method : C:\CHEM32\1\METHODS\OD-03-60-0.6.M
Last changed    : 3/3/2022 9:54:51 PM
Additional Info : Peak(s) manually integrated
```

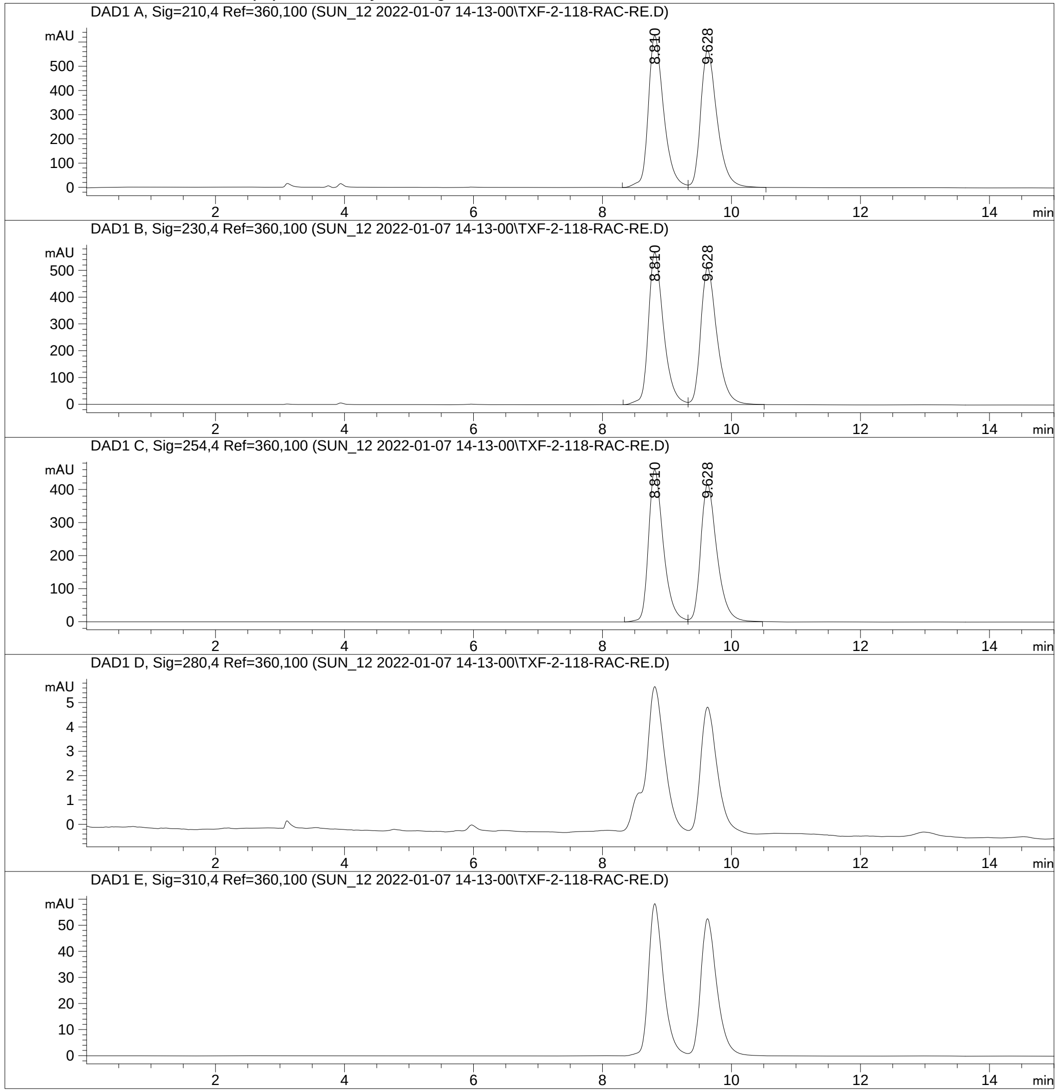

Data File C:\CHEM32\1\DATA\SUN_12 2022-01-07 14-13-00\TXF-2-118-RAC-RE.D
Sample Name:

```
=====================================================================
                         Area Percent Report
=====================================================================

Sorted By             :      Signal
Multiplier            :      1.0000
Dilution              :      1.0000
Use Multiplier & Dilution Factor with ISTDs
```

Signal 1: DAD1 A, Sig=210,4 Ref=360,100

Peak #	RetTime [min]	Type	Width [min]	Area [mAU*s]	Height [mAU]	Area %
1	8.810	BV	0.2490	1.03860e4	627.75610	50.9001
2	9.628	VB	0.2685	1.00187e4	566.15735	49.0999
Totals :				2.04047e4	1193.91345	

Signal 2: DAD1 B, Sig=230,4 Ref=360,100

Peak #	RetTime [min]	Type	Width [min]	Area [mAU*s]	Height [mAU]	Area %
1	8.810	BV	0.2465	9276.51660	568.25732	50.8288
2	9.628	VB	0.2668	8974.00000	511.26633	49.1712
Totals :				1.82505e4	1079.52365	

Signal 3: DAD1 C, Sig=254,4 Ref=360,100

Peak #	RetTime [min]	Type	Width [min]	Area [mAU*s]	Height [mAU]	Area %
1	8.810	BV	0.2427	7378.35400	460.96875	50.2517
2	9.628	VB	0.2668	7304.43652	416.16684	49.7483
Totals :				1.46828e4	877.13559	

Signal 4: DAD1 D, Sig=280,4 Ref=360,100

Signal 5: DAD1 E, Sig=310,4 Ref=360,100

```
=====================================================================
                         *** End of Report ***
```

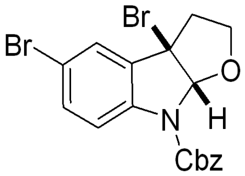

2u
(racemic)

Supplementary Figure 145. HPLC Spectrum of **2u** (racemic)

Data File C:\CHEM32\1\DATA\SUN_12 2022-01-07 14-13-00\TXF-2-118-3.D
Sample Name:

===

Acq. Operator   :   Seq. Line :   4
Acq. Instrument : Instrument 1   Location : Vial 2
Injection Date : 1/7/2022 2:58:45 PM   Inj :   1
                                        Inj Volume : 5.000 µl
Different Inj Volume from Sequence !   Actual Inj Volume : 2.000 µl
Acq. Method   : C:\CHEM32\1\DATA\SUN_12 2022-01-07 14-13-00\OD-05-20.M
Last changed   : 1/7/2022 2:52:52 PM
                  (modified after loading)
Analysis Method : C:\CHEM32\1\METHODS\OD-03-60-0.6.M
Last changed   : 3/3/2022 9:54:51 PM
Additional Info : Peak(s) manually integrated

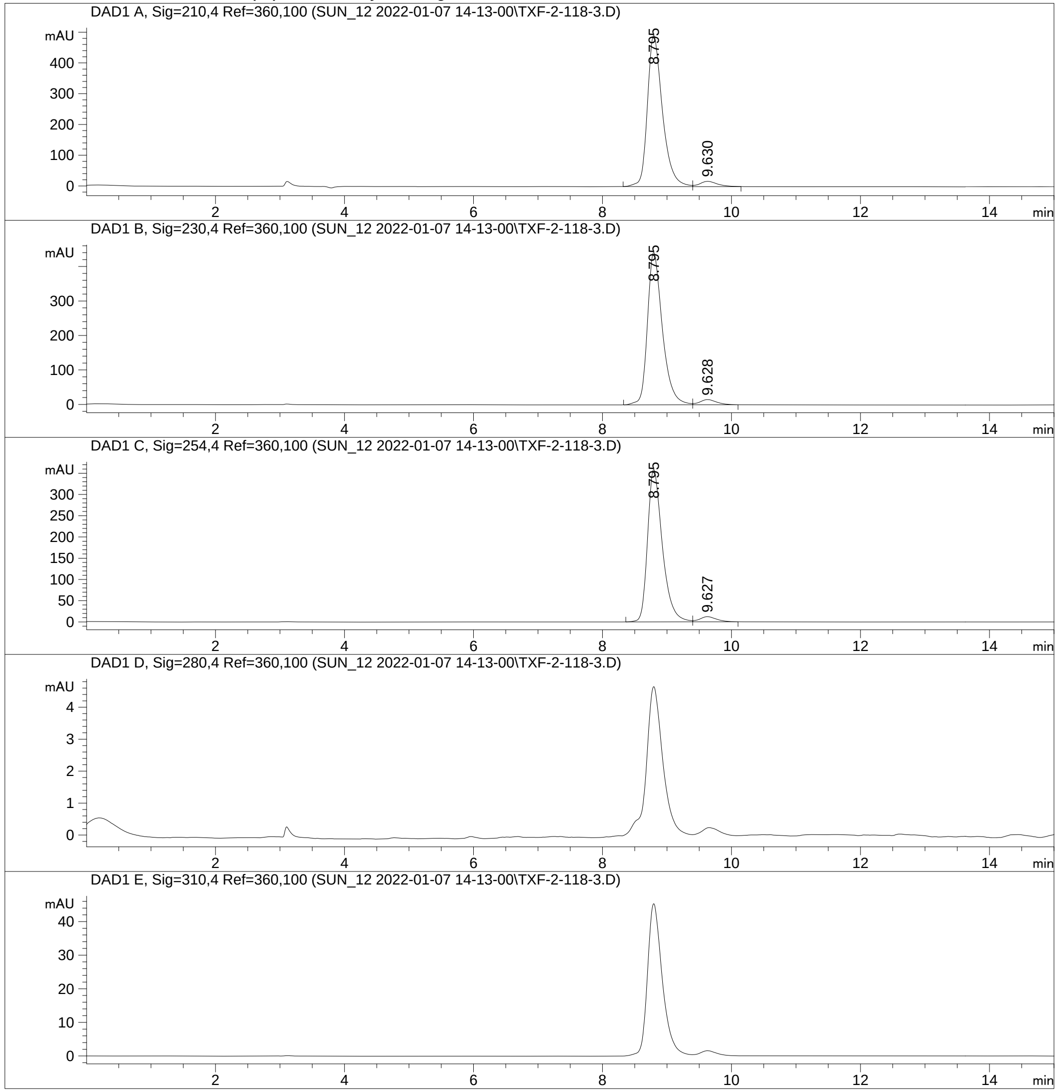

Data File C:\CHEM32\1\DATA\SUN_12 2022-01-07 14-13-00\TXF-2-118-3.D
Sample Name:

===

Area Percent Report

===

Sorted By   :   Signal
Multiplier   :   1.0000
Dilution   :   1.0000
Use Multiplier & Dilution Factor with ISTDs

Signal 1: DAD1 A, Sig=210,4 Ref=360,100

Peak #	RetTime [min]	Type	Width [min]	Area [mAU*s]	Height [mAU]	Area %
1	8.795	BV	0.2433	7973.85156	491.48416	96.0526
2	9.630	VB	0.2839	327.69510	17.38530	3.9474

Totals : 8301.54666 508.86946

Signal 2: DAD1 B, Sig=230,4 Ref=360,100

Peak #	RetTime [min]	Type	Width [min]	Area [mAU*s]	Height [mAU]	Area %
1	8.795	BV	0.2421	7121.69531	441.76529	96.2062
2	9.628	VB	0.2743	280.83887	15.29137	3.7938

Totals : 7402.53418 457.05666

Signal 3: DAD1 C, Sig=254,4 Ref=360,100

Peak #	RetTime [min]	Type	Width [min]	Area [mAU*s]	Height [mAU]	Area %
1	8.795	BV	0.2402	5723.03564	358.67038	96.2391
2	9.627	VB	0.2730	223.65027	12.24858	3.7609

Totals : 5946.68591 370.91896

Signal 4: DAD1 D, Sig=280,4 Ref=360,100

Signal 5: DAD1 E, Sig=310,4 Ref=360,100

===

*** End of Report ***

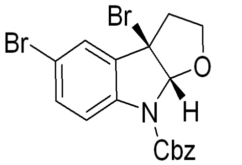

2u
(enantioenriched)

Supplementary Figure 146. HPLC Spectrum of **2u** (enantioenriched)

Data File C:\CHEM32\1\DATA\SUN_12 2022-01-07 14-13-00\TXF-2-119-RAC.D
Sample Name:

```
=====================================================================
Acq. Operator   :                               Seq. Line :   7
Acq. Instrument : Instrument 1                   Location : Vial 3
Injection Date  : 1/7/2022 3:30:57 PM                 Inj :   1
                                               Inj Volume : 5.000 µl
Different Inj Volume from Sequence !     Actual Inj Volume : 2.000 µl
Acq. Method     : C:\CHEM32\1\DATA\SUN_12 2022-01-07 14-13-00\AD-15-22.M
Last changed    : 1/7/2022 3:46:03 PM
                  (modified after loading)
Analysis Method : C:\CHEM32\1\METHODS\OD-03-60-0.6.M
Last changed    : 3/3/2022 9:54:51 PM
Additional Info : Peak(s) manually integrated
```

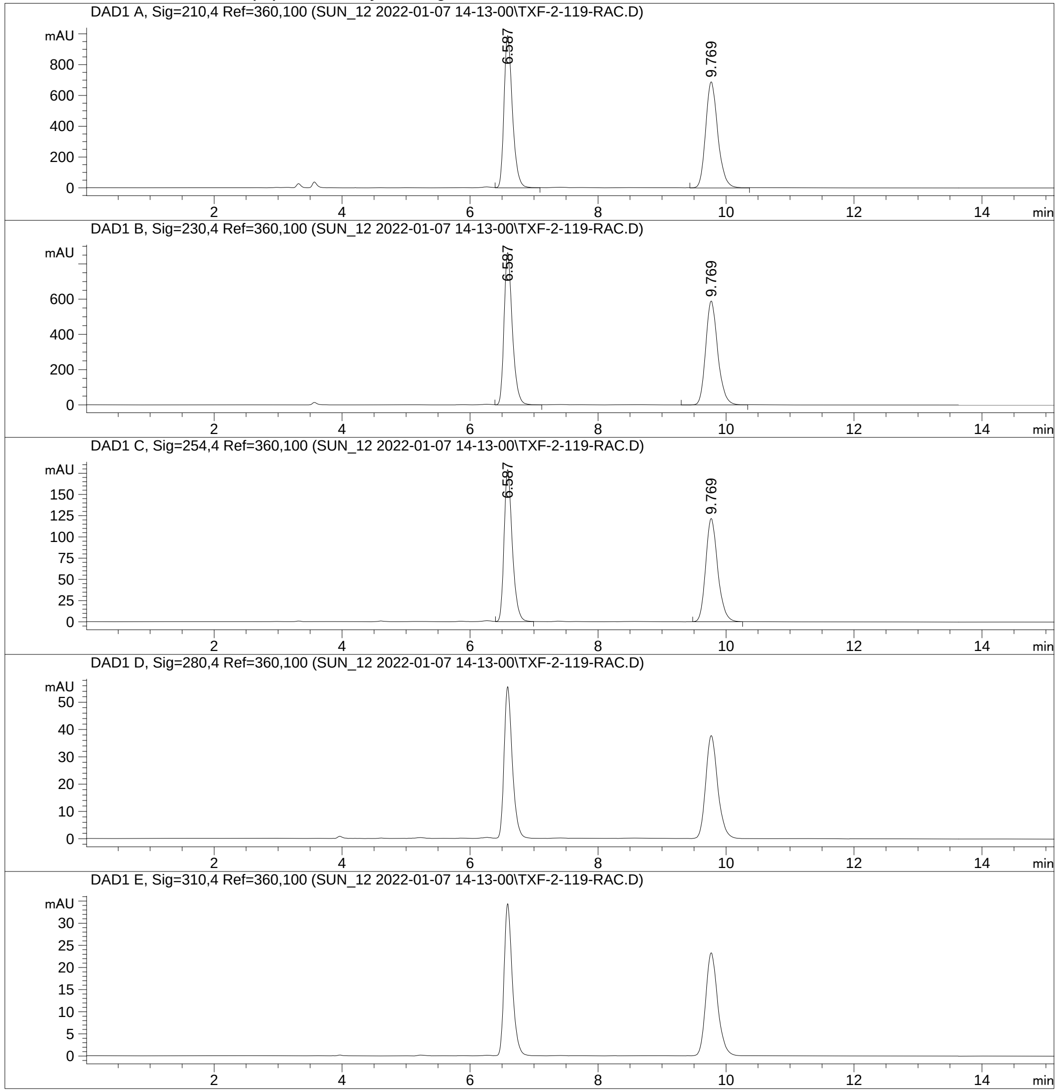

Data File C:\CHEM32\1\DATA\SUN_12 2022-01-07 14-13-00\TXF-2-119-RAC.D
Sample Name:


```
=====================================================================
                         Area Percent Report
=====================================================================

Sorted By             :      Signal
Multiplier            :      1.0000
Dilution              :      1.0000
Use Multiplier & Dilution Factor with ISTDs
```

Signal 1: DAD1 A, Sig=210,4 Ref=360,100

Peak #	RetTime [min]	Type	Width [min]	Area [mAU*s]	Height [mAU]	Area %
1	6.587	VV	0.1404	9028.96191	990.30408	49.7192
2	9.769	VB	0.2045	9130.93848	689.37830	50.2808

Totals : 1.81599e4 1679.68237

Signal 2: DAD1 B, Sig=230,4 Ref=360,100

Peak #	RetTime [min]	Type	Width [min]	Area [mAU*s]	Height [mAU]	Area %
1	6.587	VB	0.1345	7745.49414	864.87689	50.0617
2	9.769	BB	0.1989	7726.39648	589.61157	49.9383

Totals : 1.54719e4 1454.48846

Signal 3: DAD1 C, Sig=254,4 Ref=360,100

Peak #	RetTime [min]	Type	Width [min]	Area [mAU*s]	Height [mAU]	Area %
1	6.587	VB	0.1339	1595.01746	179.10780	50.0936
2	9.769	BB	0.1985	1589.05493	121.60085	49.9064

Totals : 3184.07239 300.70866

Signal 4: DAD1 D, Sig=280,4 Ref=360,100

Signal 5: DAD1 E, Sig=310,4 Ref=360,100

```
=====================================================================
                          *** End of Report ***
```

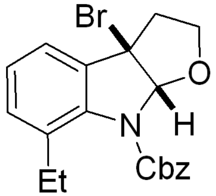

2v
(racemic)

Supplementary Figure 147. HPLC Spectrum of **2v** (racemic)

Data File C:\CHEM32\1\DATA\SUN_12 2022-01-07 14-13-00\TXF-2-119-3RE.D
Sample Name:

```
=====================================================================
Acq. Operator   :                             Seq. Line :  16
Acq. Instrument : Instrument 1                 Location : Vial 4
Injection Date  : 1/7/2022 5:55:25 PM               Inj :   1
                                             Inj Volume : 5.000 µl
Different Inj Volume from Sequence !     Actual Inj Volume : 2.000 µl
Acq. Method     : C:\CHEM32\1\DATA\SUN_12 2022-01-07 14-13-00\AD-15-22.M
Last changed    : 9/15/2015 8:34:07 PM
Analysis Method : C:\CHEM32\1\METHODS\OD-03-60-0.6.M
Last changed    : 3/3/2022 9:54:51 PM
Additional Info : Peak(s) manually integrated
```

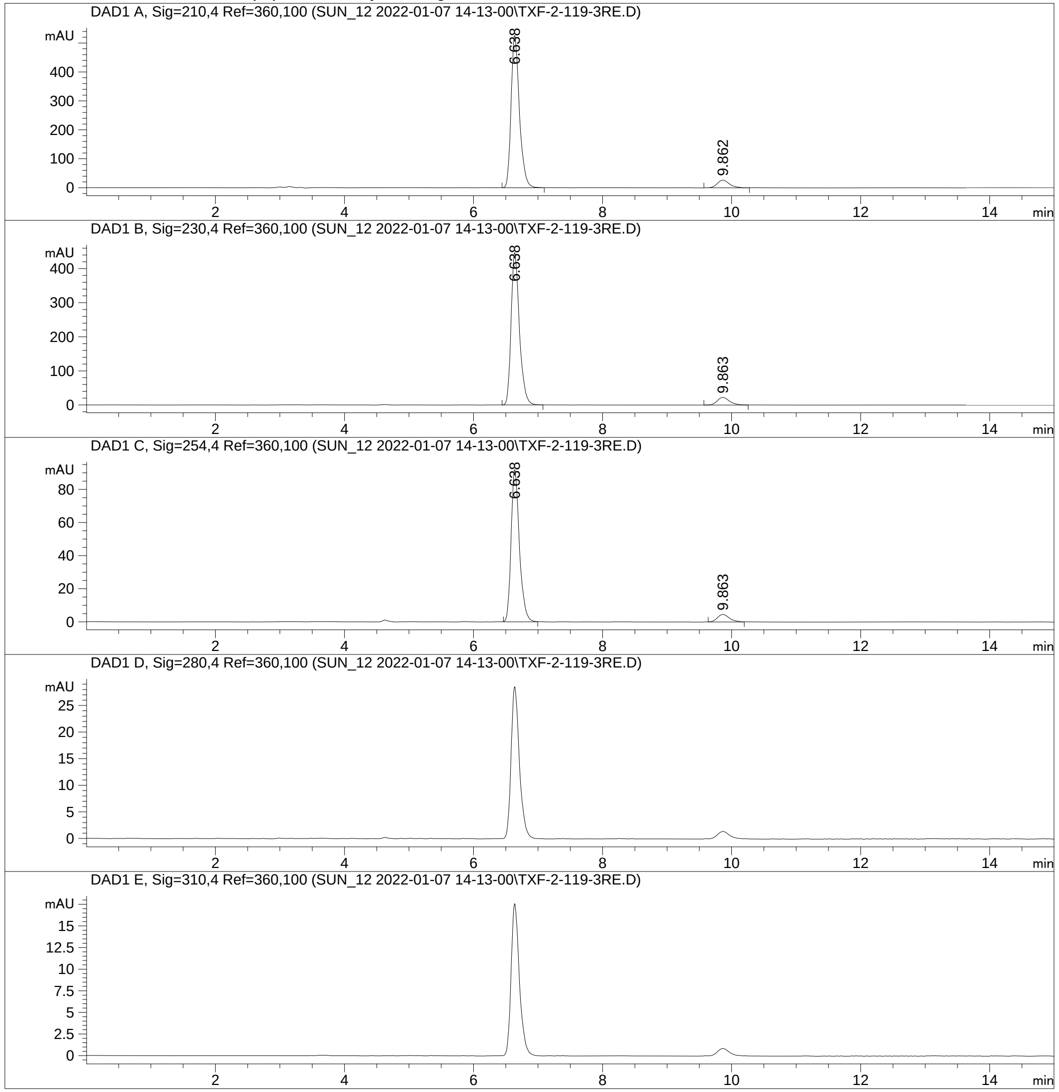

Data File C:\CHEM32\1\DATA\SUN_12 2022-01-07 14-13-00\TXF-2-119-3RE.D
Sample Name:

Area Percent Report

Sorted By : Signal
Multiplier : 1.0000
Dilution : 1.0000
Use Multiplier & Dilution Factor with ISTDs

Signal 1: DAD1 A, Sig=210,4 Ref=360,100

Peak #	RetTime [min]	Type	Width [min]	Area [mAU*s]	Height [mAU]	Area %
1	6.638	BB	0.1367	4728.69873	526.66498	93.0810
2	9.862	BB	0.1980	351.49985	26.62877	6.9190

Totals : 5080.19858 553.29375

Signal 2: DAD1 B, Sig=230,4 Ref=360,100

Peak #	RetTime [min]	Type	Width [min]	Area [mAU*s]	Height [mAU]	Area %
1	6.638	BB	0.1342	3996.17676	447.47458	93.0358
2	9.863	BB	0.2010	299.13245	22.51715	6.9642

Totals : 4295.30920 469.99173

Signal 3: DAD1 C, Sig=254,4 Ref=360,100

Peak #	RetTime [min]	Type	Width [min]	Area [mAU*s]	Height [mAU]	Area %
1	6.638	BB	0.1340	820.78845	92.05262	93.1704
2	9.863	BB	0.1952	60.16528	4.58103	6.8296

Totals : 880.95373 96.63365

Signal 4: DAD1 D, Sig=280,4 Ref=360,100

Signal 5: DAD1 E, Sig=310,4 Ref=360,100

*** End of Report ***

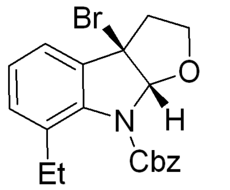

2v
(enantioenriched)

Supplementary Figure 148. HPLC Spectrum of **2v** (enantioenriched)

Data File C:\CHEM32\1\DATA\SUN_12 2022-03-11 14-01-43\TXF-3-177RAC.D
Sample Name:

```
=====================================================================
Acq. Operator   :                              Seq. Line :   4
Acq. Instrument : Instrument 1                   Location : Vial 3
Injection Date  : 3/11/2022 2:42:49 PM                Inj :   1
                                               Inj Volume : 5.000 µl
Different Inj Volume from Sequence !     Actual Inj Volume : 3.000 µl
Acq. Method     : C:\CHEM32\1\DATA\SUN_12 2022-03-11 14-01-43\OD-01-15.M
Last changed    : 3/11/2022 2:27:51 PM
                  (modified after loading)
Analysis Method : C:\CHEM32\1\METHODS\OD-03-60-0.6.M
Last changed    : 3/3/2022 9:54:51 PM
Additional Info : Peak(s) manually integrated
```

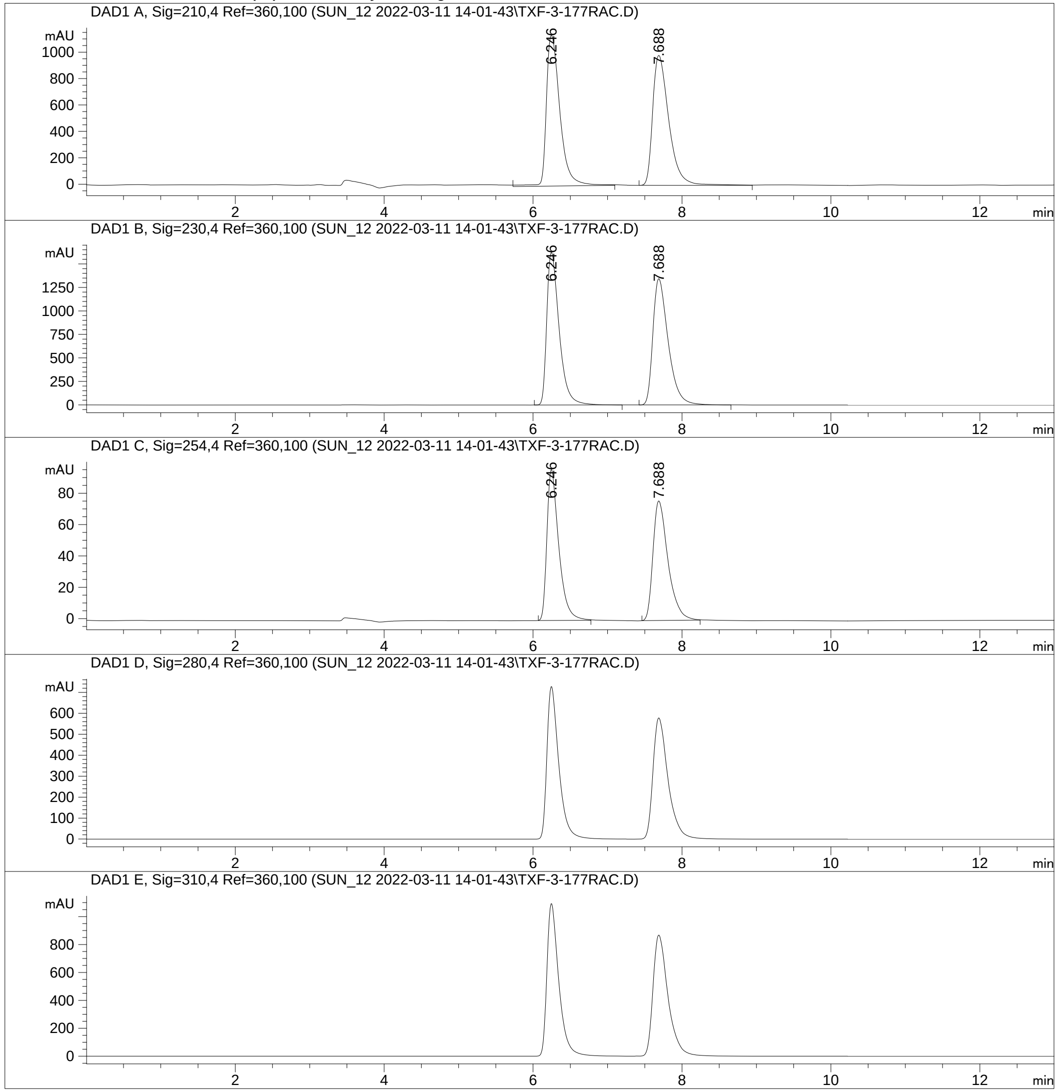

Data File C:\CHEM32\1\DATA\SUN_12 2022-03-11 14-01-43\TXF-3-177RAC.D
Sample Name:

Area Percent Report

Sorted By	:	Signal
Multiplier	:	1.0000
Dilution	:	1.0000

Use Multiplier & Dilution Factor with ISTDs

Signal 1: DAD1 A, Sig=210,4 Ref=360,100

Peak #	RetTime [min]	Type	Width [min]	Area [mAU*s]	Height [mAU]	Area %
1	6.246	VB	0.1944	1.45204e4	1141.30945	49.4058
2	7.688	BB	0.2323	1.48696e4	984.08594	50.5942
Totals :				2.93900e4	2125.39539	

Signal 2: DAD1 B, Sig=230,4 Ref=360,100

Peak #	RetTime [min]	Type	Width [min]	Area [mAU*s]	Height [mAU]	Area %
1	6.246	BB	0.1769	1.87836e4	1624.28967	49.5289
2	7.688	BB	0.2162	1.91409e4	1343.32422	50.4711
Totals :				3.79245e4	2967.61389	

Signal 3: DAD1 C, Sig=254,4 Ref=360,100

Peak #	RetTime [min]	Type	Width [min]	Area [mAU*s]	Height [mAU]	Area %
1	6.246	BB	0.1651	1066.52075	96.39680	49.9862
2	7.688	BB	0.2112	1067.11145	76.30145	50.0138
Totals :				2133.63220	172.69826	

Signal 4: DAD1 D, Sig=280,4 Ref=360,100

Signal 5: DAD1 E, Sig=310,4 Ref=360,100

*** End of Report ***

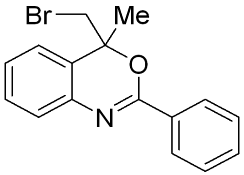

4a
(racemic)

Supplementary Figure 149. HPLC Spectrum of **4a** (racemic)

Data File C:\CHEM32\1\DATA\SUN_12 2022-03-11 14-01-43\TXF-3-177-2.D
Sample Name:

===

```
Acq. Operator   :                                  Seq. Line :   5
Acq. Instrument : Instrument 1                      Location : Vial 4
Injection Date  : 3/11/2022 2:56:46 PM                   Inj :   1
                                                  Inj Volume : 5.000 µl
Different Inj Volume from Sequence !     Actual Inj Volume : 2.000 µl
Acq. Method     : C:\CHEM32\1\DATA\SUN_12 2022-03-11 14-01-43\OD-01-15.M
Last changed    : 3/11/2022 2:27:51 PM
                  (modified after loading)
Analysis Method : C:\CHEM32\1\METHODS\OD-03-60-0.6.M
Last changed    : 3/3/2022 9:54:51 PM
Additional Info : Peak(s) manually integrated
```

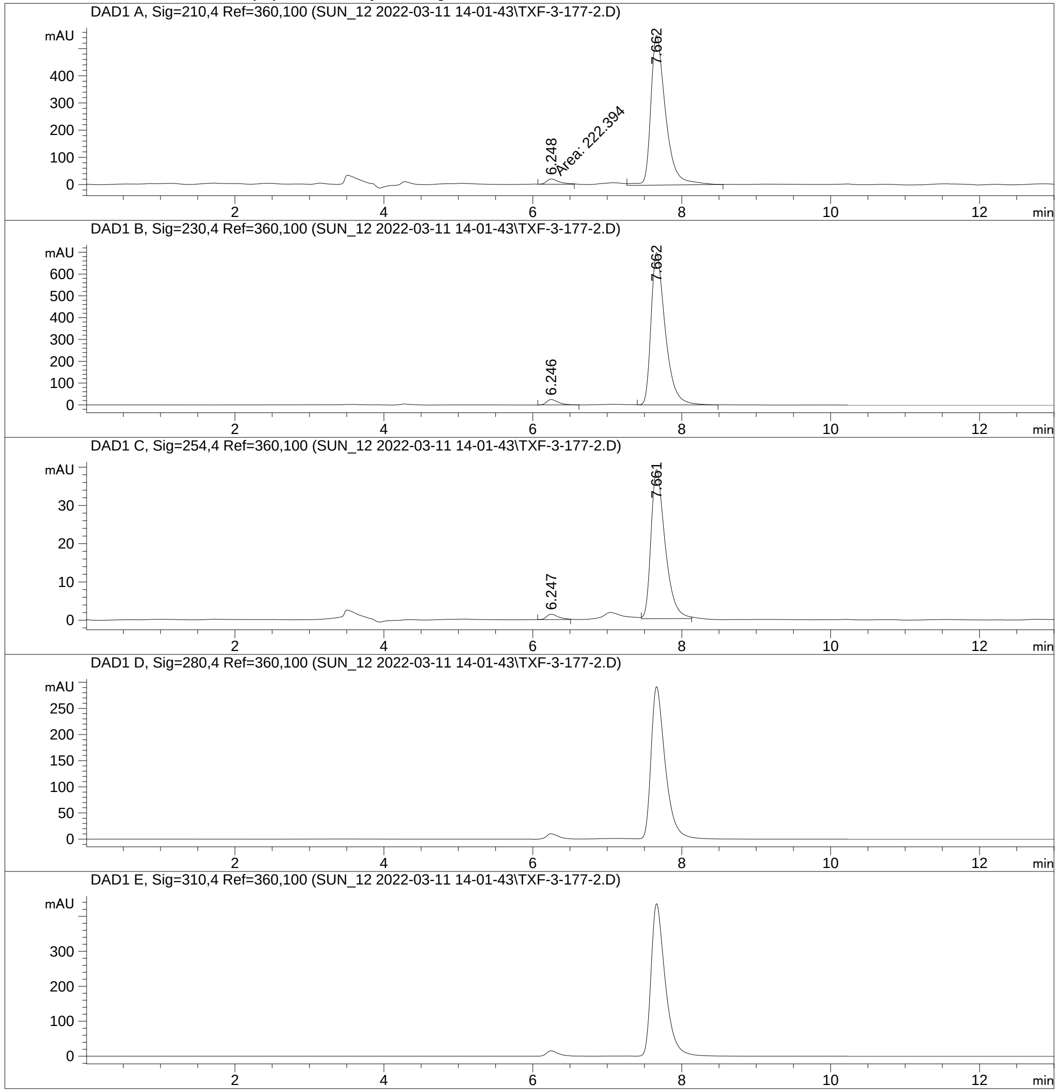

Data File C:\CHEM32\1\DATA\SUN_12 2022-03-11 14-01-43\TXF-3-177-2.D
Sample Name:

===

Area Percent Report

===

```
Sorted By             :      Signal
Multiplier            :      1.0000
Dilution              :      1.0000
Use Multiplier & Dilution Factor with ISTDs
```

Signal 1: DAD1 A, Sig=210,4 Ref=360,100

Peak #	RetTime [min]	Type	Width [min]	Area [mAU*s]	Height [mAU]	Area %
1	6.248	MM	0.1906	222.39377	19.45015	2.8393
2	7.662	VB	0.2073	7610.32959	550.61444	97.1607

Totals : 7832.72336 570.06459

Signal 2: DAD1 B, Sig=230,4 Ref=360,100

Peak #	RetTime [min]	Type	Width [min]	Area [mAU*s]	Height [mAU]	Area %
1	6.246	BB	0.1606	267.53104	25.05035	2.8151
2	7.662	VB	0.1982	9235.87500	698.76257	97.1849

Totals : 9503.40604 723.81292

Signal 3: DAD1 C, Sig=254,4 Ref=360,100

Peak #	RetTime [min]	Type	Width [min]	Area [mAU*s]	Height [mAU]	Area %
1	6.247	BB	0.1621	15.29777	1.41496	2.9003
2	7.661	BB	0.1973	512.15436	38.98171	97.0997

Totals : 527.45213 40.39667

Signal 4: DAD1 D, Sig=280,4 Ref=360,100

Signal 5: DAD1 E, Sig=310,4 Ref=360,100

===

*** End of Report ***

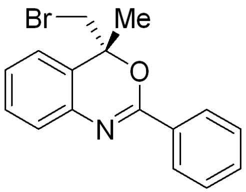

4a
(enantioenriched)

Supplementary Figure 150. HPLC Spectrum of **4a** (enantioenriched)

```
=====================================================================
Acq. Operator   :                        Seq. Line :   2
Acq. Instrument : Instrument 1            Location : Vial 1
Injection Date  : 3/12/2022 2:31:44 PM          Inj :   1
                                        Inj Volume : 5.000 µl
Different Inj Volume from Sequence !     Actual Inj Volume : 3.000 µl
Acq. Method     : C:\CHEM32\1\DATA\SUN_12 2022-03-12 14-13-57\OD-01-15.M
Last changed    : 3/1/2022 2:47:17 PM
Analysis Method : C:\CHEM32\1\METHODS\OD-03-60-0.6.M
Last changed    : 3/3/2022 9:54:51 PM
Additional Info : Peak(s) manually integrated
```

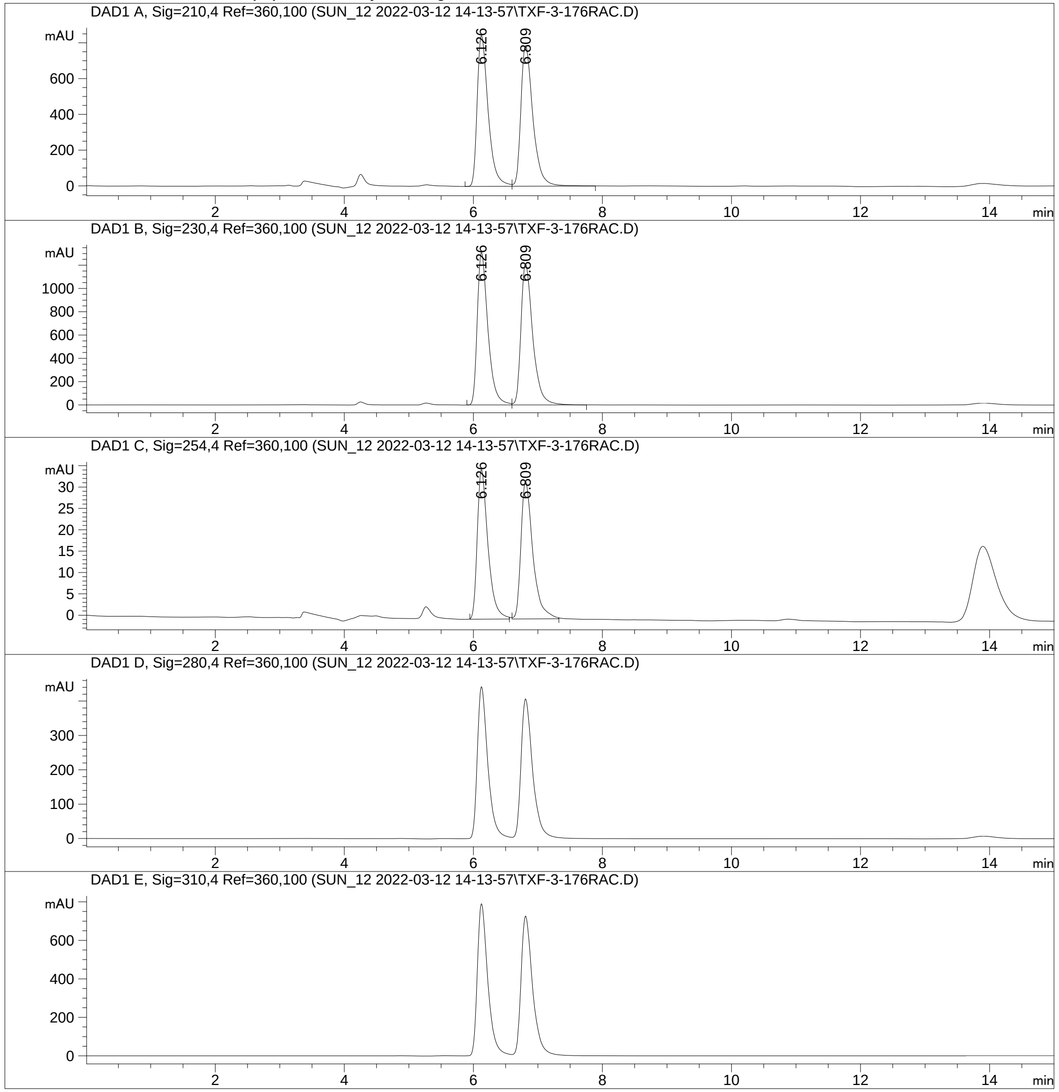

Data File C:\CHEM32\1\DATA\SUN_12 2022-03-12 14-13-57\TXF-3-176RAC.D
Sample Name:

```
=====================================================================
                         Area Percent Report
=====================================================================

Sorted By             :      Signal
Multiplier            :      1.0000
Dilution              :      1.0000
Use Multiplier & Dilution Factor with ISTDs
```

Signal 1: DAD1 A, Sig=210,4 Ref=360,100

Peak #	RetTime [min]	Type	Width [min]	Area [mAU*s]	Height [mAU]	Area %
1	6.126	BV	0.1801	9984.78711	843.62970	49.5189
2	6.809	VB	0.1970	1.01788e4	786.31116	50.4811

Totals : 2.01636e4 1629.94086

Signal 2: DAD1 B, Sig=230,4 Ref=360,100

Peak #	RetTime [min]	Type	Width [min]	Area [mAU*s]	Height [mAU]	Area %
1	6.126	BV	0.1743	1.50661e4	1309.08813	49.4639
2	6.809	VB	0.1920	1.53926e4	1213.13220	50.5361

Totals : 3.04587e4 2522.22034

Signal 3: DAD1 C, Sig=254,4 Ref=360,100

Peak #	RetTime [min]	Type	Width [min]	Area [mAU*s]	Height [mAU]	Area %
1	6.126	BB	0.1726	403.97940	35.01407	49.4476
2	6.809	BB	0.1935	413.00513	32.21949	50.5524

Totals : 816.98453 67.23356

Signal 4: DAD1 D, Sig=280,4 Ref=360,100

Signal 5: DAD1 E, Sig=310,4 Ref=360,100

```
=====================================================================
                         *** End of Report ***
```

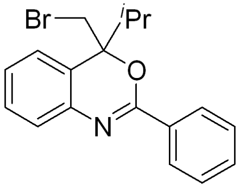

4b
(racemic)

Supplementary Figure 151. HPLC Spectrum of **4b** (racemic)

Data File C:\CHEM32\1\DATA\SUN_12 2022-03-12 14-13-57\TXF-3-176-8.D
Sample Name:

```
=====================================================================
Acq. Operator   :                              Seq. Line :   3
Acq. Instrument : Instrument 1                  Location : Vial 2
Injection Date  : 3/12/2022 2:47:42 PM               Inj :   1
                                              Inj Volume : 5.000 µl
Different Inj Volume from Sequence !     Actual Inj Volume : 2.000 µl
Acq. Method     : C:\CHEM32\1\DATA\SUN_12 2022-03-12 14-13-57\OD-01-15.M
Last changed    : 3/1/2022 2:47:17 PM
Analysis Method : C:\CHEM32\1\METHODS\OD-03-60-0.6.M
Last changed    : 3/3/2022 9:54:51 PM
Additional Info : Peak(s) manually integrated
```

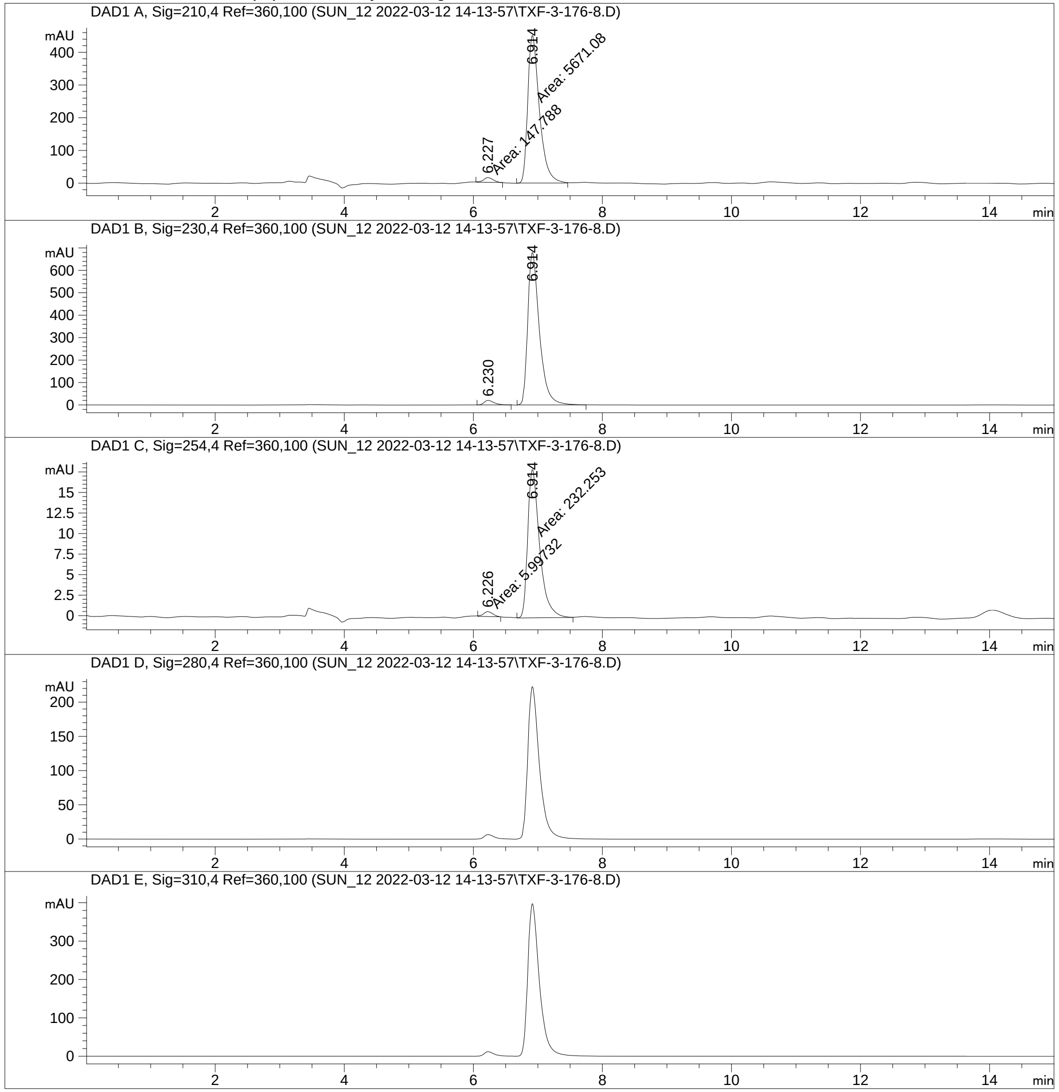

Data File C:\CHEM32\1\DATA\SUN_12 2022-03-12 14-13-57\TXF-3-176-8.D
Sample Name:

Area Percent Report

```
Sorted By             :      Signal
Multiplier            :      1.0000
Dilution              :      1.0000
Use Multiplier & Dilution Factor with ISTDs
```

Signal 1: DAD1 A, Sig=210,4 Ref=360,100

Peak #	RetTime [min]	Type	Width [min]	Area [mAU*s]	Height [mAU]	Area %
1	6.227	MM	0.1704	147.78778	14.45545	2.5398
2	6.914	MM	0.2096	5671.08057	450.93967	97.4602

Totals : 5818.86835 465.39511

Signal 2: DAD1 B, Sig=230,4 Ref=360,100

Peak #	RetTime [min]	Type	Width [min]	Area [mAU*s]	Height [mAU]	Area %
1	6.230	BB	0.1623	220.86606	20.40550	2.5190
2	6.914	BB	0.1887	8546.97852	679.64880	97.4810

Totals : 8767.84457 700.05431

Signal 3: DAD1 C, Sig=254,4 Ref=360,100

Peak #	RetTime [min]	Type	Width [min]	Area [mAU*s]	Height [mAU]	Area %
1	6.226	MM	0.1670	5.99732	5.98549e-1	2.5172
2	6.914	MM	0.2143	232.25301	18.06551	97.4828

Totals : 238.25033 18.66406

Signal 4: DAD1 D, Sig=280,4 Ref=360,100

Signal 5: DAD1 E, Sig=310,4 Ref=360,100

*** End of Report ***

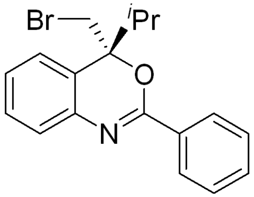

4b
(enantioenriched)

Supplementary Figure 152. HPLC Spectrum of **4b** (enantioenriched)

Data File C:\CHEM32\1\DATA\SUN_12 2022-03-13 18-54-13\TXF-3-187-RAC3.D
Sample Name:

===

```
Acq. Operator   :                               Seq. Line :   7
Acq. Instrument : Instrument 1                   Location : Vial 3
Injection Date  : 3/13/2022 8:15:51 PM                Inj :   1
                                               Inj Volume : 5.000 µl
Different Inj Volume from Sequence !     Actual Inj Volume : 2.000 µl
Acq. Method     : C:\CHEM32\1\DATA\SUN_12 2022-03-13 18-54-13\OD-01-30-0.5.M
Last changed    : 3/13/2022 8:32:36 PM
                  (modified after loading)
Analysis Method : C:\CHEM32\1\METHODS\AD-10-10.M
Last changed    : 6/17/2016 2:27:06 PM
Additional Info : Peak(s) manually integrated
```

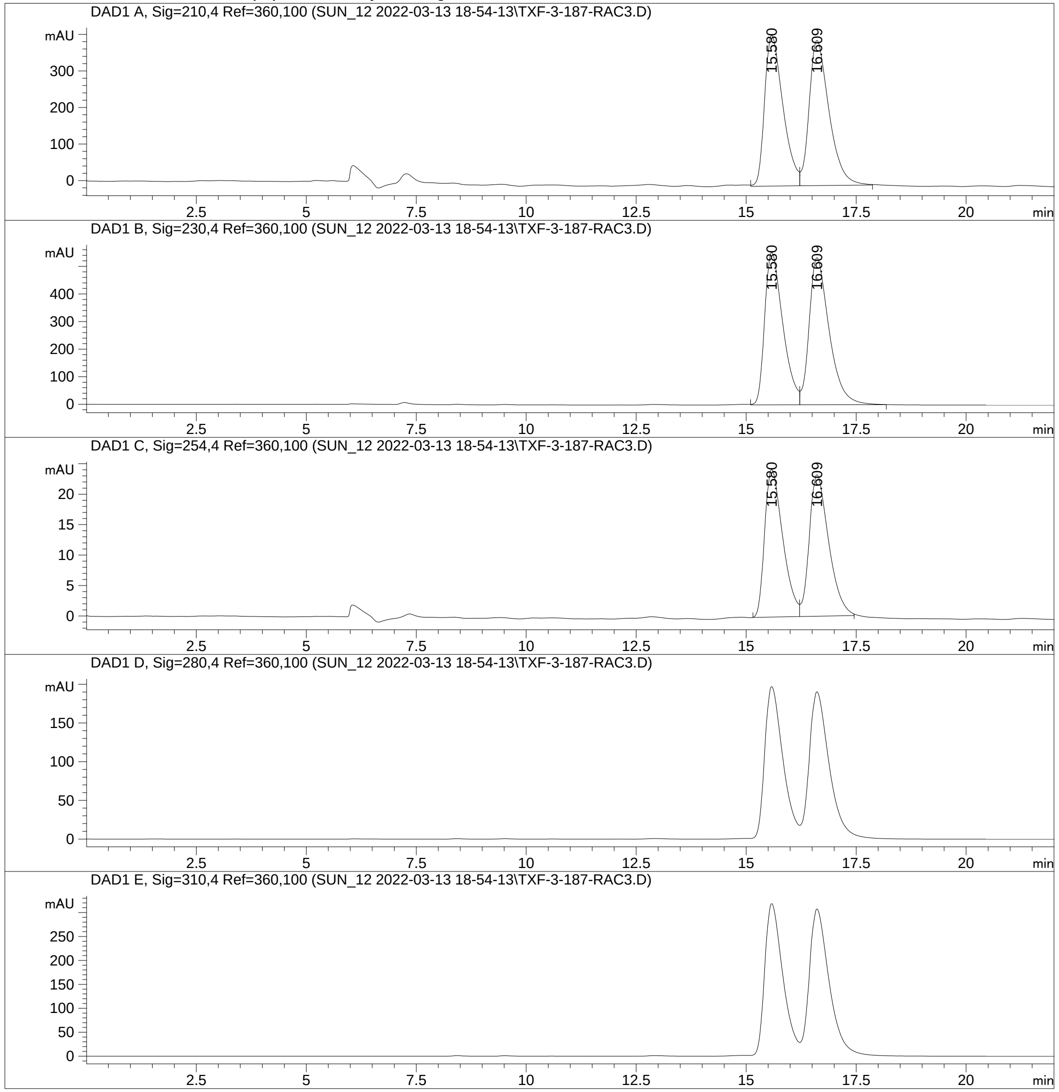

Data File C:\CHEM32\1\DATA\SUN_12 2022-03-13 18-54-13\TXF-3-187-RAC3.D
Sample Name:

===
Area Percent Report
===

```
Sorted By             :      Signal
Multiplier            :      1.0000
Dilution              :      1.0000
Use Multiplier & Dilution Factor with ISTDs
```

Signal 1: DAD1 A, Sig=210,4 Ref=360,100

Peak #	RetTime [min]	Type	Width [min]	Area [mAU*s]	Height [mAU]	Area %
1	15.580	VV	0.4456	1.19250e4	412.06711	48.8483
2	16.609	VB	0.4768	1.24873e4	397.21948	51.1517

Totals : 2.44124e4 809.28659

Signal 2: DAD1 B, Sig=230,4 Ref=360,100

Peak #	RetTime [min]	Type	Width [min]	Area [mAU*s]	Height [mAU]	Area %
1	15.580	BV	0.4383	1.58335e4	552.50690	48.4656
2	16.609	VB	0.4784	1.68361e4	533.11957	51.5344

Totals : 3.26696e4 1085.62646

Signal 3: DAD1 C, Sig=254,4 Ref=360,100

Peak #	RetTime [min]	Type	Width [min]	Area [mAU*s]	Height [mAU]	Area %
1	15.580	BV	0.4402	693.87476	24.22605	49.3095
2	16.609	VB	0.4684	713.30719	23.21907	50.6905

Totals : 1407.18195 47.44512

Signal 4: DAD1 D, Sig=280,4 Ref=360,100

Signal 5: DAD1 E, Sig=310,4 Ref=360,100

===
*** End of Report ***

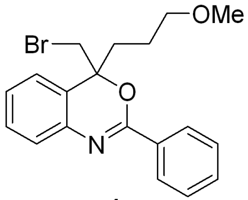

4c
(racemic)

Supplementary Figure 153. HPLC Spectrum of **4c** (racemic)

===

Acq. Operator :   Seq. Line : 8
Acq. Instrument : Instrument 1   Location : Vial 4
Injection Date : 3/13/2022 8:38:51 PM   Inj : 1
                                                  Inj Volume : 5.000 µl
Different Inj Volume from Sequence !   Actual Inj Volume : 2.000 µl
Acq. Method : C:\CHEM32\1\DATA\SUN_12 2022-03-13 18-54-13\OD-01-30-0.5.M
Last changed : 3/13/2022 8:32:36 PM
(modified after loading)
Analysis Method : C:\CHEM32\1\METHODS\AD-10-10.M
Last changed : 6/17/2016 2:27:06 PM
Additional Info : Peak(s) manually integrated

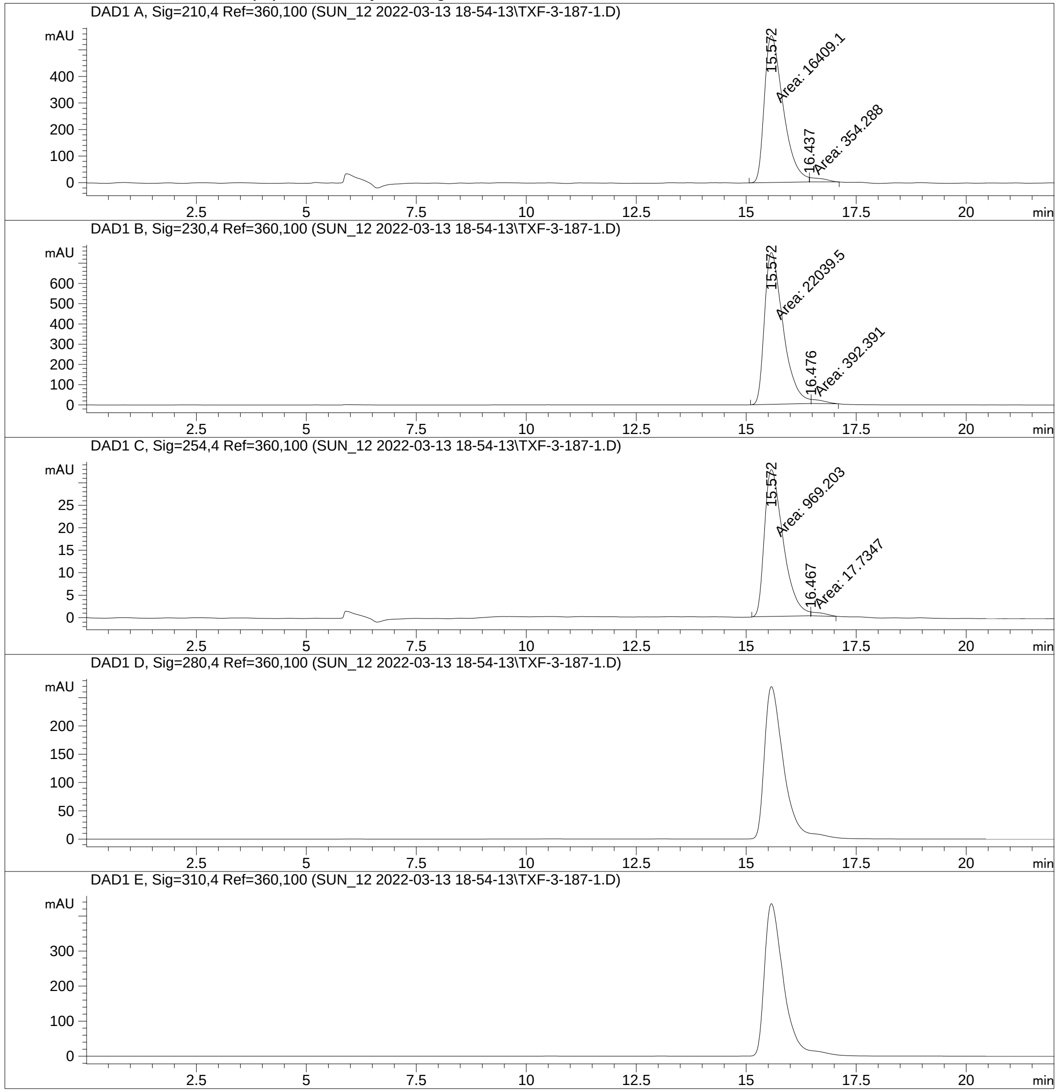

Data File C:\CHEM32\1\DATA\SUN_12 2022-03-13 18-54-13\TXF-3-187-1.D
Sample Name:

===
Area Percent Report
===

Sorted By : Signal
Multiplier : 1.0000
Dilution : 1.0000
Use Multiplier & Dilution Factor with ISTDs

Signal 1: DAD1 A, Sig=210,4 Ref=360,100

Peak #	RetTime [min]	Type	Width [min]	Area [mAU*s]	Height [mAU]	Area %
1	15.572	MM	0.4939	1.64091e4	553.67719	97.8865
2	16.437	MM	0.2649	354.28809	16.59890	2.1135

Totals : 1.67634e4 570.27609

Signal 2: DAD1 B, Sig=230,4 Ref=360,100

Peak #	RetTime [min]	Type	Width [min]	Area [mAU*s]	Height [mAU]	Area %
1	15.572	MM	0.4898	2.20395e4	749.93494	98.2507
2	16.476	MM	0.2417	392.39066	20.27401	1.7493

Totals : 2.24319e4 770.20894

Signal 3: DAD1 C, Sig=254,4 Ref=360,100

Peak #	RetTime [min]	Type	Width [min]	Area [mAU*s]	Height [mAU]	Area %
1	15.572	MM	0.4941	969.20258	32.68940	98.2031
2	16.467	MM	0.2489	17.73473	8.45761e-1	1.7969

Totals : 986.93730 33.53516

Signal 4: DAD1 D, Sig=280,4 Ref=360,100

Signal 5: DAD1 E, Sig=310,4 Ref=360,100

===
*** End of Report ***

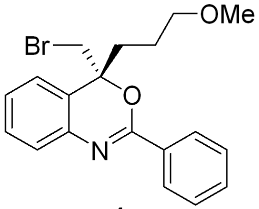

4c
(enantioenriched)

Supplementary Figure 154. HPLC Spectrum of **4c** (enantioenriched)

Data File C:\CHEM32\1\DATA\SUN_12 2022-03-19 08-46-22\TXF-4-10RAC.D
Sample Name:

```
=====================================================================
Acq. Operator   :                             Seq. Line :   4
Acq. Instrument : Instrument 1                 Location : Vial 8
Injection Date  : 3/19/2022 9:45:08 AM              Inj :   1
                                             Inj Volume : 5.000 µl
Different Inj Volume from Sequence !     Actual Inj Volume : 2.000 µl
Acq. Method     : C:\CHEM32\1\DATA\SUN_12 2022-03-19 08-46-22\AD-01-20.M
Last changed    : 3/19/2022 9:26:03 AM
                  (modified after loading)
Analysis Method : C:\CHEM32\1\METHODS\AD-10-10.M
Last changed    : 6/17/2016 2:27:06 PM
Additional Info : Peak(s) manually integrated
```

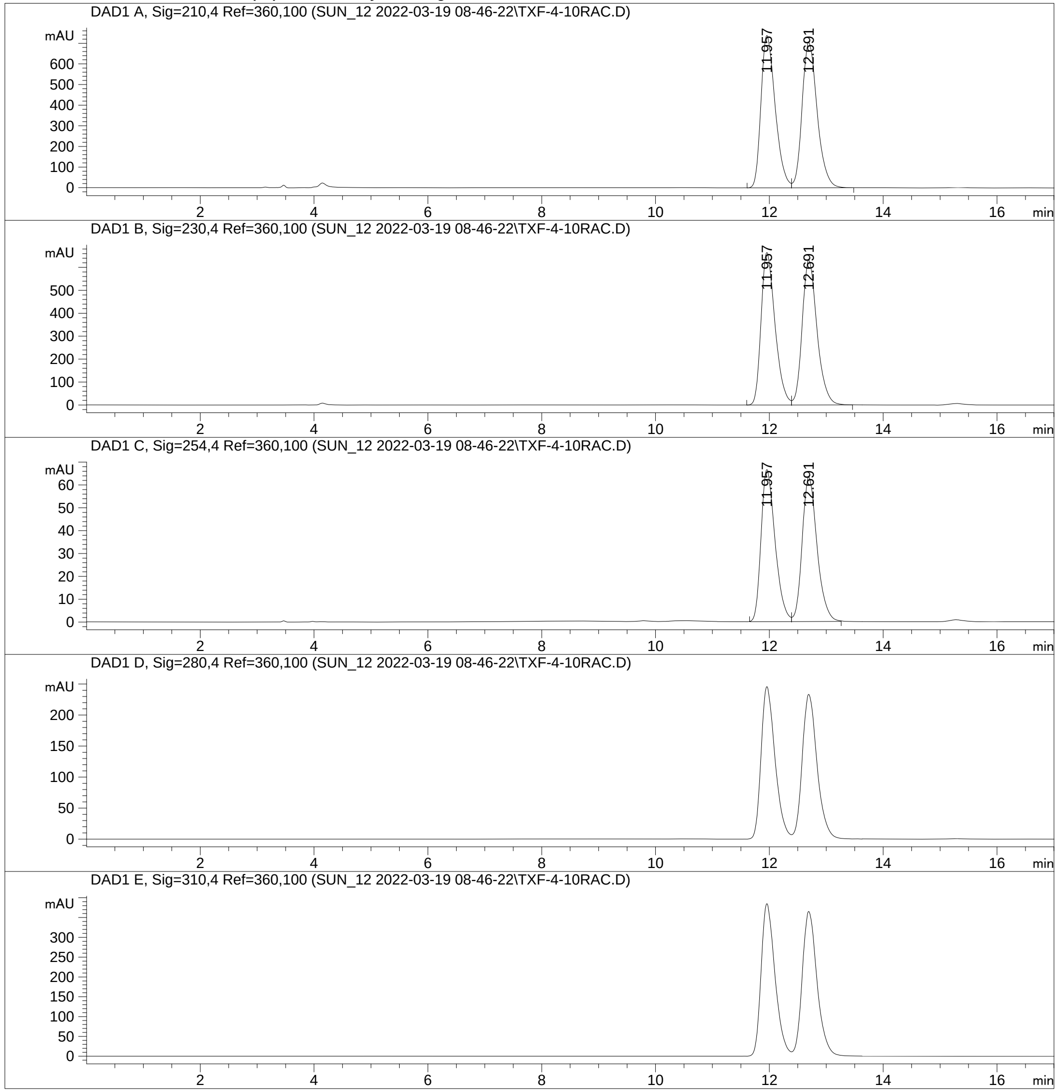

Data File C:\CHEM32\1\DATA\SUN_12 2022-03-19 08-46-22\TXF-4-10RAC.D
Sample Name:

```
=====================================================================
                         Area Percent Report
=====================================================================

Sorted By             :      Signal
Multiplier            :      1.0000
Dilution              :      1.0000
Use Multiplier & Dilution Factor with ISTDs
```

Signal 1: DAD1 A, Sig=210,4 Ref=360,100

Peak #	RetTime [min]	Type	Width [min]	Area [mAU*s]	Height [mAU]	Area %
1	11.957	BV	0.2686	1.28322e4	738.84497	49.6309
2	12.691	VB	0.2856	1.30230e4	704.76740	50.3691

Totals : 2.58552e4 1443.61237

Signal 2: DAD1 B, Sig=230,4 Ref=360,100

Peak #	RetTime [min]	Type	Width [min]	Area [mAU*s]	Height [mAU]	Area %
1	11.957	BV	0.2628	1.13340e4	665.02155	49.7120
2	12.691	VB	0.2799	1.14653e4	631.39008	50.2880

Totals : 2.27993e4 1296.41162

Signal 3: DAD1 C, Sig=254,4 Ref=360,100

Peak #	RetTime [min]	Type	Width [min]	Area [mAU*s]	Height [mAU]	Area %
1	11.957	BV	0.2637	1139.19189	66.56116	49.7768
2	12.691	VB	0.2802	1149.40747	63.18465	50.2232

Totals : 2288.59937 129.74581

Signal 4: DAD1 D, Sig=280,4 Ref=360,100

Signal 5: DAD1 E, Sig=310,4 Ref=360,100

```
=====================================================================
                        *** End of Report ***
```

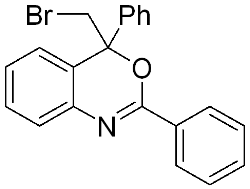

4d
(racemic)

Supplementary Figure 155. HPLC Spectrum of **4d** (racemic)

Data File C:\CHEM32\1\DATA\SUN_12 2022-03-19 08-46-22\TXF-4-10-1.D
Sample Name:

===

```
Acq. Operator   :                               Seq. Line :   5
Acq. Instrument : Instrument 1                   Location : Vial 10
Injection Date  : 3/19/2022 10:03:09 AM               Inj :   1
                                               Inj Volume : 5.000 µl
Different Inj Volume from Sequence !     Actual Inj Volume : 2.000 µl
Acq. Method     : C:\CHEM32\1\DATA\SUN_12 2022-03-19 08-46-22\AD-01-20.M
Last changed    : 3/19/2022 9:26:03 AM
                  (modified after loading)
Analysis Method : C:\CHEM32\1\METHODS\AD-10-10.M
Last changed    : 6/17/2016 2:27:06 PM
Additional Info : Peak(s) manually integrated
```

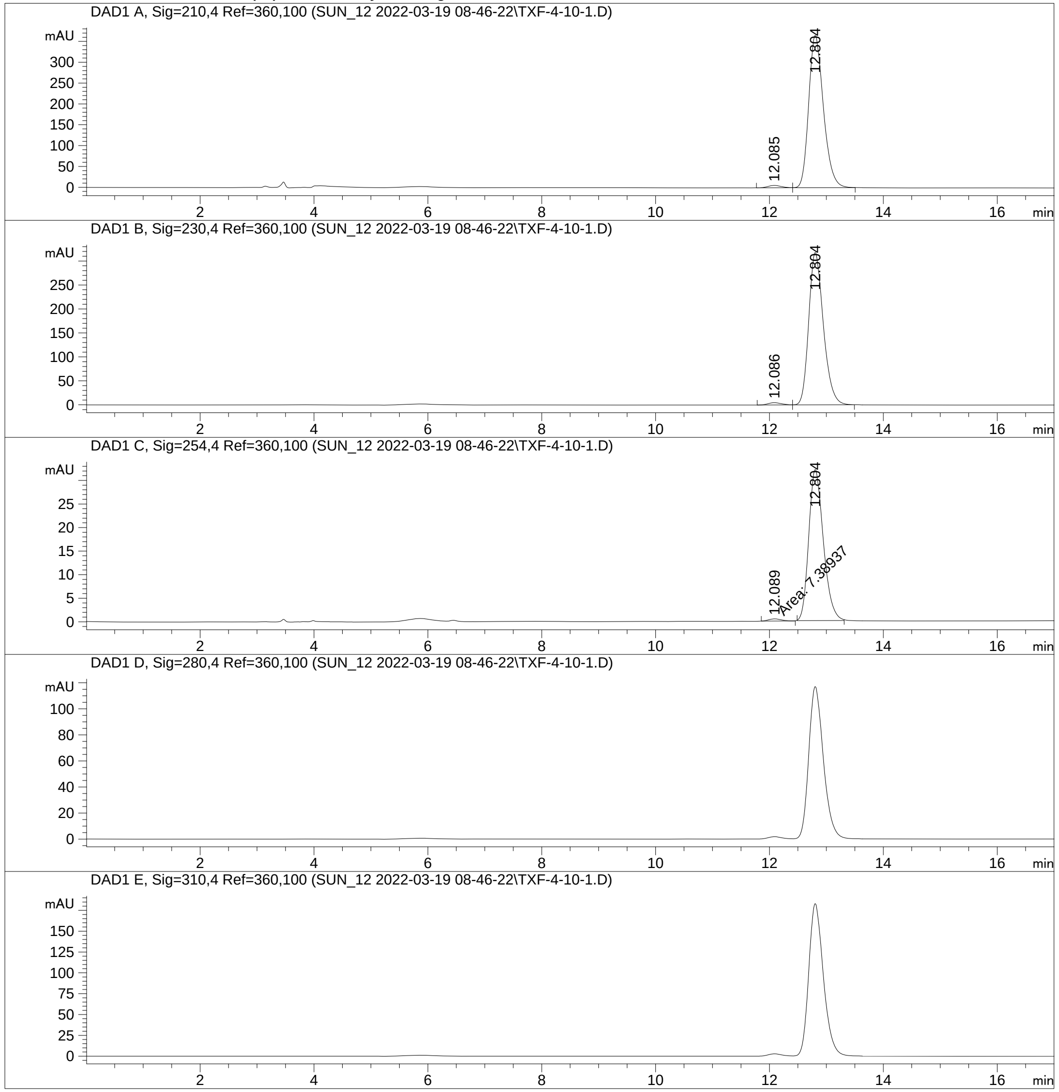

Data File C:\CHEM32\1\DATA\SUN_12 2022-03-19 08-46-22\TXF-4-10-1.D
Sample Name:

===

Area Percent Report

===

```
Sorted By             :      Signal
Multiplier            :      1.0000
Dilution              :      1.0000
Use Multiplier & Dilution Factor with ISTDs
```

Signal 1: DAD1 A, Sig=210,4 Ref=360,100

Peak #	RetTime [min]	Type	Width [min]	Area [mAU*s]	Height [mAU]	Area %
1	12.085	BV	0.2542	93.68593	5.68541	1.4047
2	12.804	VB	0.2759	6575.58008	365.46170	98.5953
Totals :				6669.26601	371.14711	

Signal 2: DAD1 B, Sig=230,4 Ref=360,100

Peak #	RetTime [min]	Type	Width [min]	Area [mAU*s]	Height [mAU]	Area %
1	12.086	BV	0.2544	79.85189	4.84250	1.3848
2	12.804	VB	0.2746	5686.31494	317.97787	98.6152
Totals :				5766.16683	322.82037	

Signal 3: DAD1 C, Sig=254,4 Ref=360,100

Peak #	RetTime [min]	Type	Width [min]	Area [mAU*s]	Height [mAU]	Area %
1	12.089	MM	0.2650	7.38937	4.64765e-1	1.2761
2	12.804	BB	0.2741	571.69220	32.05682	98.7239
Totals :				579.08157	32.52159	

Signal 4: DAD1 D, Sig=280,4 Ref=360,100

Signal 5: DAD1 E, Sig=310,4 Ref=360,100

===

*** End of Report ***

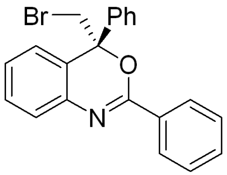

4d
(enantioenriched)

Supplementary Figure 156. HPLC Spectrum of **4d** (enantioenriched)

```
=====================================================================
Acq. Operator   :                               Seq. Line :   7
Acq. Instrument : Instrument 1                   Location : Vial 3
Injection Date  : 3/12/2022 3:41:46 PM                Inj :   1
                                               Inj Volume : 5.000 µl
Different Inj Volume from Sequence !     Actual Inj Volume : 3.000 µl
Acq. Method     : C:\CHEM32\1\DATA\SUN_12 2022-03-12 14-13-57\OD-03-30.M
Last changed    : 3/12/2022 3:52:02 PM
                  (modified after loading)
Analysis Method : C:\CHEM32\1\METHODS\OD-03-60-0.6.M
Last changed    : 3/3/2022 9:54:51 PM
Additional Info : Peak(s) manually integrated
```

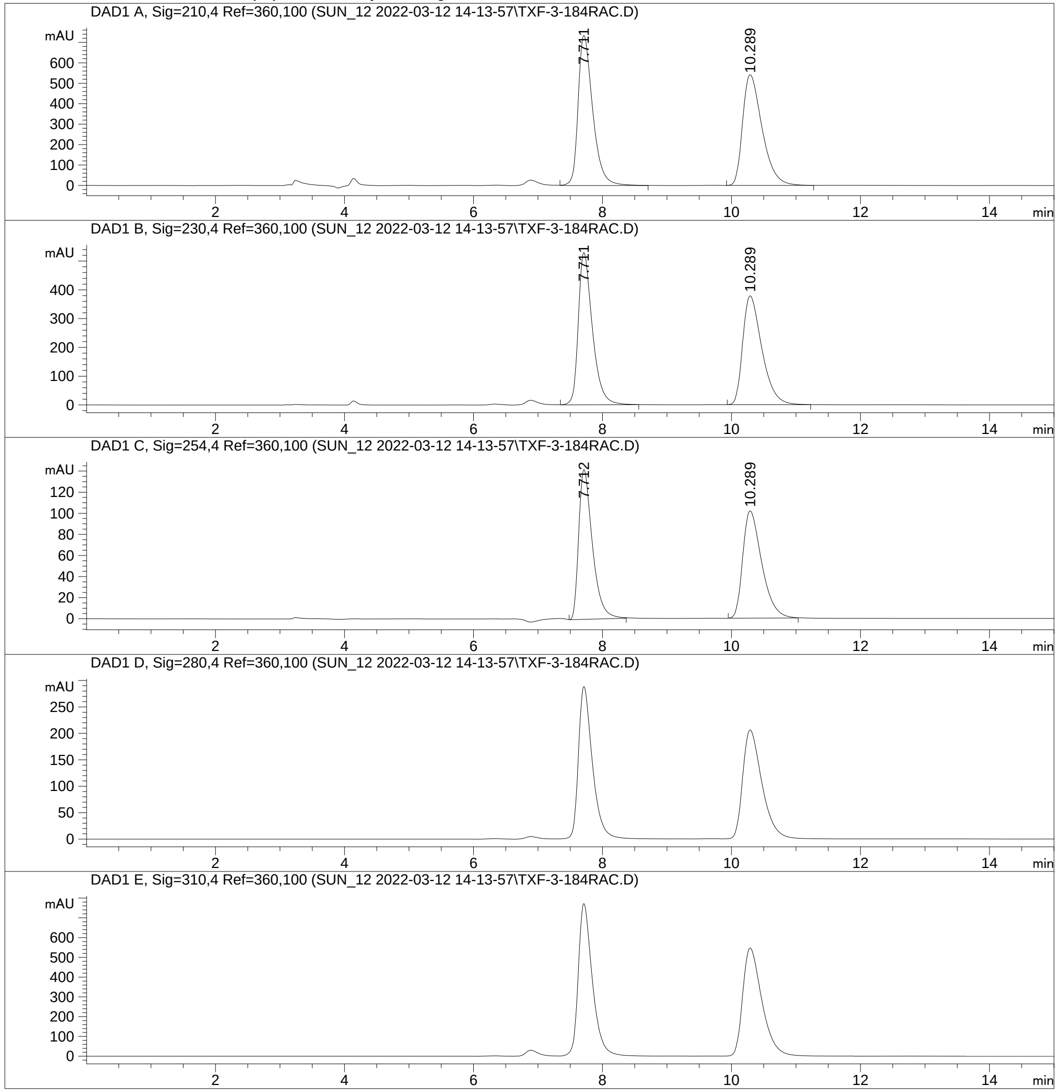

Data File C:\CHEM32\1\DATA\SUN_12 2022-03-12 14-13-57\TXF-3-184RAC.D
Sample Name:

Area Percent Report

```
Sorted By             :      Signal
Multiplier            :      1.0000
Dilution              :      1.0000
Use Multiplier & Dilution Factor with ISTDs
```

Signal 1: DAD1 A, Sig=210,4 Ref=360,100

Peak #	RetTime [min]	Type	Width [min]	Area [mAU*s]	Height [mAU]	Area %
1	7.711	BB	0.2371	1.15255e4	734.51007	50.3829
2	10.289	BB	0.3225	1.13503e4	541.50800	49.6171
Totals :				2.28758e4	1276.01807	

Signal 2: DAD1 B, Sig=230,4 Ref=360,100

Peak #	RetTime [min]	Type	Width [min]	Area [mAU*s]	Height [mAU]	Area %
1	7.711	BB	0.2290	8037.55225	529.67798	50.7302
2	10.289	BB	0.3149	7806.18164	378.08099	49.2698
Totals :				1.58437e4	907.75897	

Signal 3: DAD1 C, Sig=254,4 Ref=360,100

Peak #	RetTime [min]	Type	Width [min]	Area [mAU*s]	Height [mAU]	Area %
1	7.712	VB	0.2217	2089.47241	141.97711	50.1484
2	10.289	BB	0.3123	2077.10327	101.67886	49.8516
Totals :				4166.57568	243.65597	

Signal 4: DAD1 D, Sig=280,4 Ref=360,100

Signal 5: DAD1 E, Sig=310,4 Ref=360,100

*** End of Report ***

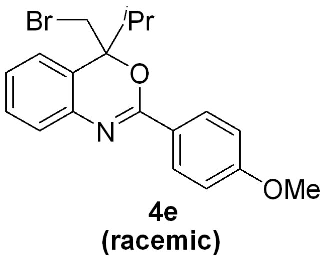

4e
(racemic)

Supplementary Figure 157. HPLC Spectrum of **4e** (racemic)

Data File C:\CHEM32\1\DATA\SUN_12 2022-03-12 14-13-57\TXF-3-184-1.D
Sample Name:

===

Acq. Operator : Seq. Line : 8
Acq. Instrument : Instrument 1 Location : Vial 4
Injection Date : 3/12/2022 3:57:44 PM Inj : 1
Inj Volume : 5.000 µl
Different Inj Volume from Sequence ! Actual Inj Volume : 2.000 µl
Acq. Method : C:\CHEM32\1\DATA\SUN_12 2022-03-12 14-13-57\OD-03-30.M
Last changed : 3/12/2022 3:52:02 PM
(modified after loading)
Analysis Method : C:\CHEM32\1\METHODS\OD-03-60-0.6.M
Last changed : 3/3/2022 9:54:51 PM
Additional Info : Peak(s) manually integrated

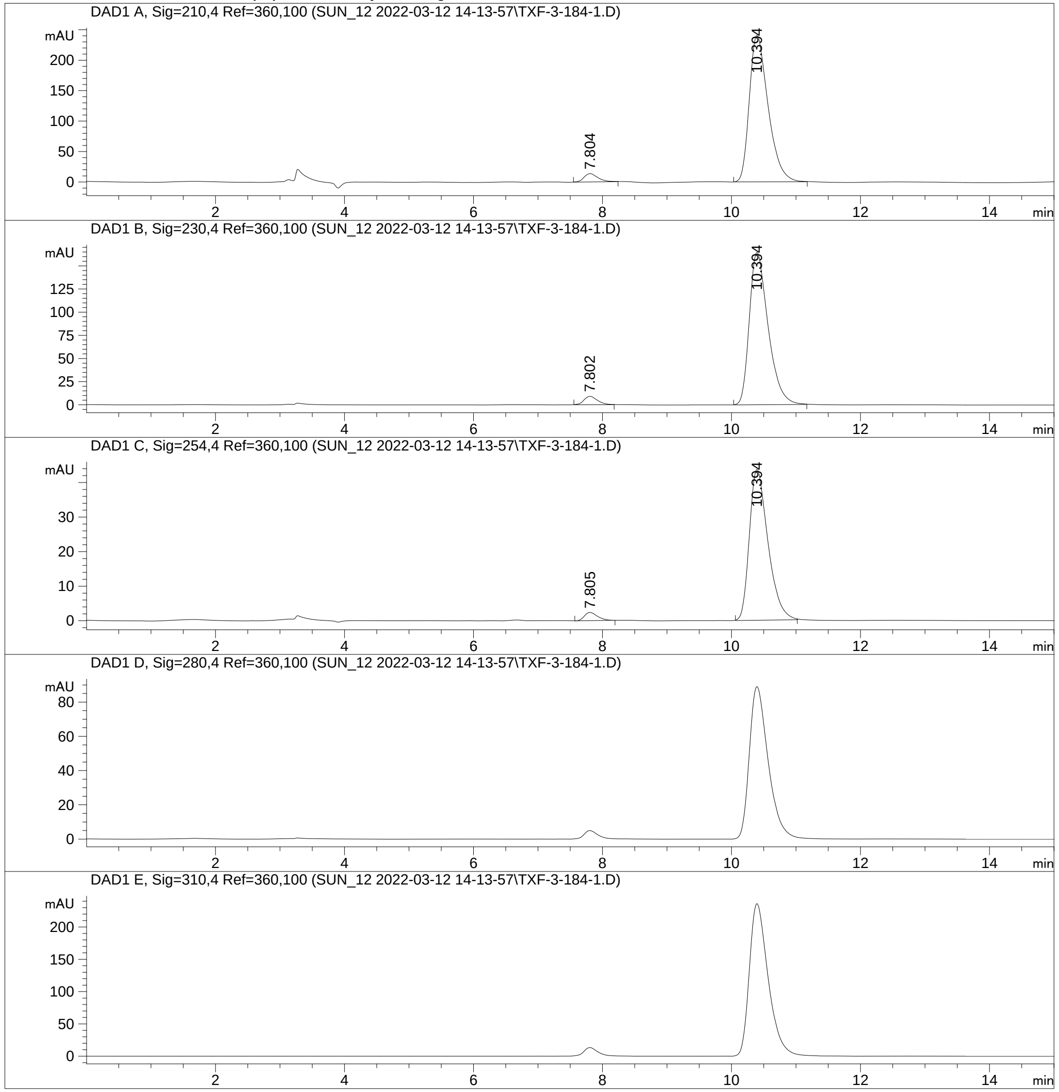

Data File C:\CHEM32\1\DATA\SUN_12 2022-03-12 14-13-57\TXF-3-184-1.D
Sample Name:

===

Area Percent Report

===

Sorted By : Signal
Multiplier : 1.0000
Dilution : 1.0000
Use Multiplier & Dilution Factor with ISTDs

Signal 1: DAD1 A, Sig=210,4 Ref=360,100

Peak #	RetTime [min]	Type	Width [min]	Area [mAU*s]	Height [mAU]	Area %
1	7.804	BB	0.2244	202.44952	13.69961	3.9943
2	10.394	BB	0.3081	4865.97607	240.41522	96.0057

Totals : 5068.42560 254.11483

Signal 2: DAD1 B, Sig=230,4 Ref=360,100

Peak #	RetTime [min]	Type	Width [min]	Area [mAU*s]	Height [mAU]	Area %
1	7.802	BB	0.2194	130.85599	9.11891	3.7876
2	10.394	BB	0.3080	3324.02246	164.34389	96.2124

Totals : 3454.87845 173.46280

Signal 3: DAD1 C, Sig=254,4 Ref=360,100

Peak #	RetTime [min]	Type	Width [min]	Area [mAU*s]	Height [mAU]	Area %
1	7.805	BB	0.2129	33.36340	2.38901	3.6784
2	10.394	BB	0.3058	873.63947	43.59868	96.3216

Totals : 907.00286 45.98769

Signal 4: DAD1 D, Sig=280,4 Ref=360,100

Signal 5: DAD1 E, Sig=310,4 Ref=360,100

===

*** End of Report ***

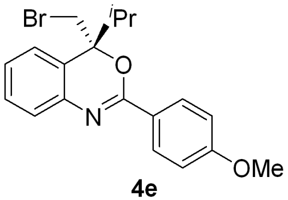

4e
(enantioenriched)

Supplementary Figure 158. HPLC Spectrum of **4e** (enantioenriched)

Data File C:\CHEM32\1\DATA\SUN_12 2022-03-12 14-13-57\TXF-3-185RAC.D
Sample Name:

Acq. Operator : Seq. Line : 4
Acq. Instrument : Instrument 1 Location : Vial 5
Injection Date : 3/12/2022 3:03:42 PM Inj : 1
Inj Volume : 5.000 µl
Different Inj Volume from Sequence ! Actual Inj Volume : 3.000 µl
Acq. Method : C:\CHEM32\1\DATA\SUN_12 2022-03-12 14-13-57\OD-01-15.M
Last changed : 3/1/2022 2:47:17 PM
Analysis Method : C:\CHEM32\1\METHODS\OD-03-60-0.6.M
Last changed : 3/3/2022 9:54:51 PM
Additional Info : Peak(s) manually integrated

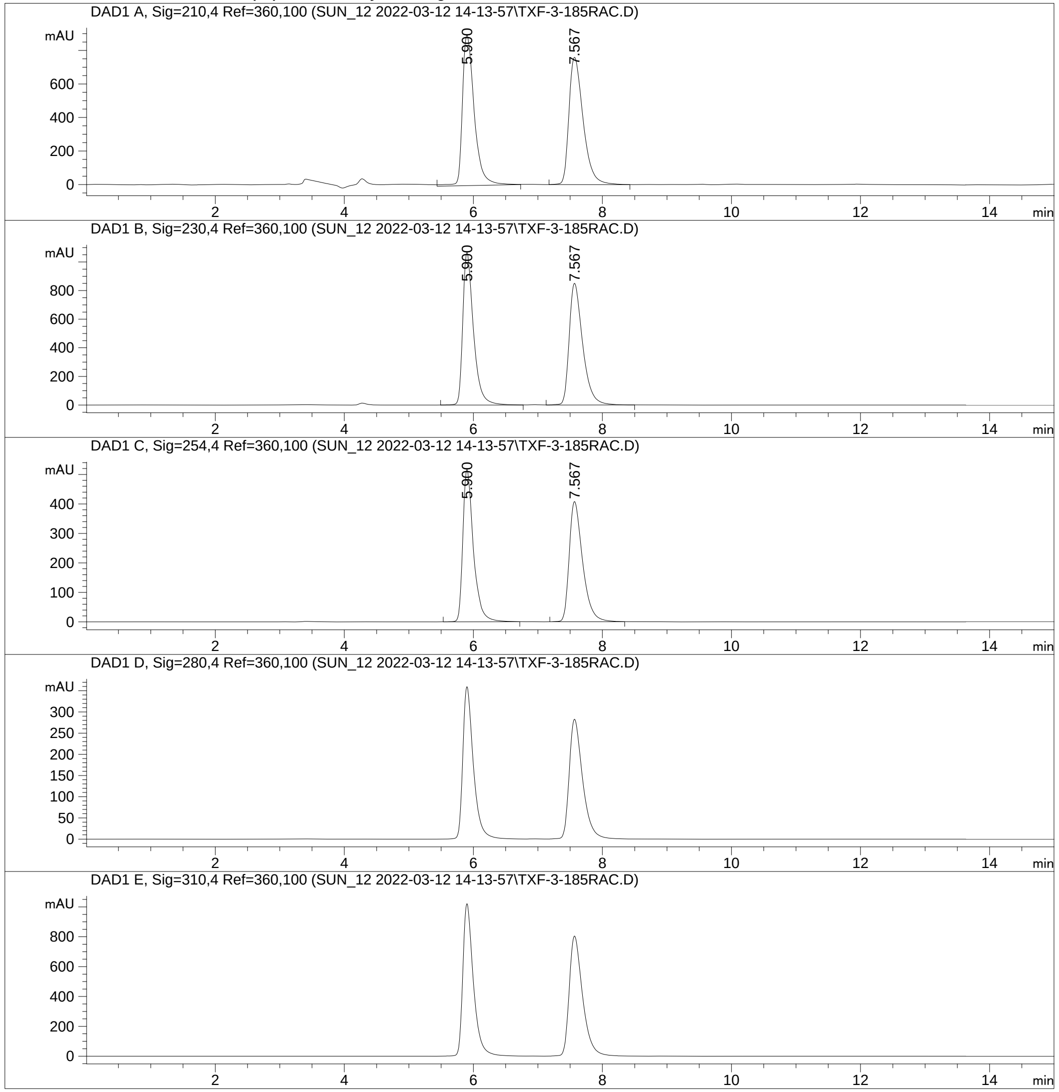

Data File C:\CHEM32\1\DATA\SUN_12 2022-03-12 14-13-57\TXF-3-185RAC.D
Sample Name:

Area Percent Report

Sorted By : Signal
Multiplier : 1.0000
Dilution : 1.0000
Use Multiplier & Dilution Factor with ISTDs

Signal 1: DAD1 A, Sig=210,4 Ref=360,100

Peak #	RetTime [min]	Type	Width [min]	Area [mAU*s]	Height [mAU]	Area %
1	5.900	VB	0.1956	1.14782e4	895.43744	49.6914
2	7.567	BB	0.2364	1.16208e4	759.74573	50.3086

Totals : 2.30990e4 1655.18317

Signal 2: DAD1 B, Sig=230,4 Ref=360,100

Peak #	RetTime [min]	Type	Width [min]	Area [mAU*s]	Height [mAU]	Area %
1	5.900	BB	0.1746	1.22989e4	1066.08740	49.8427
2	7.567	VB	0.2216	1.23765e4	851.25989	50.1573

Totals : 2.46753e4 1917.34729

Signal 3: DAD1 C, Sig=254,4 Ref=360,100

Peak #	RetTime [min]	Type	Width [min]	Area [mAU*s]	Height [mAU]	Area %
1	5.900	BB	0.1709	5884.04492	516.44159	49.9847
2	7.567	BB	0.2183	5887.63672	408.05331	50.0153

Totals : 1.17717e4 924.49490

Signal 4: DAD1 D, Sig=280,4 Ref=360,100

Signal 5: DAD1 E, Sig=310,4 Ref=360,100

*** End of Report ***

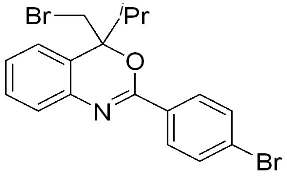

4f
(racemic)

Supplementary Figure 159. HPLC Spectrum of **4f** (racemic)

Data File C:\CHEM32\1\DATA\SUN_12 2022-03-12 14-13-57\TXF-3-185-1.D
Sample Name:

```
Acq. Operator   :                              Seq. Line :   5
Acq. Instrument : Instrument 1                  Location : Vial 6
Injection Date  : 3/12/2022 3:19:40 PM               Inj :   1
                                              Inj Volume : 5.000 µl
Different Inj Volume from Sequence !     Actual Inj Volume : 2.000 µl
Acq. Method     : C:\CHEM32\1\DATA\SUN_12 2022-03-12 14-13-57\OD-01-15.M
Last changed    : 3/1/2022 2:47:17 PM
Analysis Method : C:\CHEM32\1\METHODS\OD-03-60-0.6.M
Last changed    : 3/3/2022 9:54:51 PM
Additional Info : Peak(s) manually integrated
```

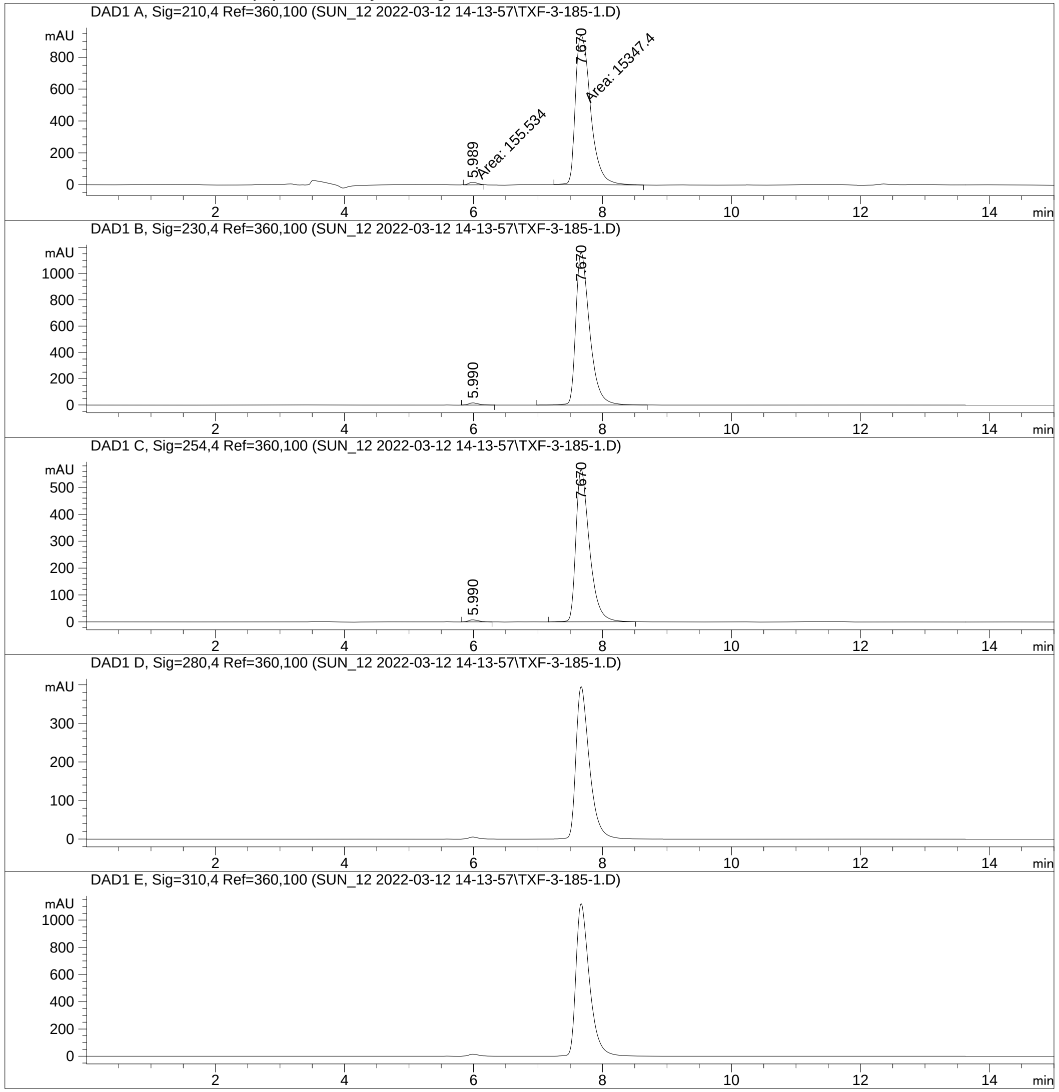

Data File C:\CHEM32\1\DATA\SUN_12 2022-03-12 14-13-57\TXF-3-185-1.D
Sample Name:

Area Percent Report

```
Sorted By            :      Signal
Multiplier           :      1.0000
Dilution             :      1.0000
Use Multiplier & Dilution Factor with ISTDs
```

Signal 1: DAD1 A, Sig=210,4 Ref=360,100

Peak #	RetTime [min]	Type	Width [min]	Area [mAU*s]	Height [mAU]	Area %
1	5.989	MM	0.1601	155.53447	16.19185	1.0033
2	7.670	MM	0.2732	1.53474e4	936.18329	98.9967

Totals : 1.55029e4 952.37514

Signal 2: DAD1 B, Sig=230,4 Ref=360,100

Peak #	RetTime [min]	Type	Width [min]	Area [mAU*s]	Height [mAU]	Area %
1	5.990	BB	0.1624	176.41058	16.28792	1.0122
2	7.670	BB	0.2255	1.72523e4	1160.15479	98.9878

Totals : 1.74287e4 1176.44270

Signal 3: DAD1 C, Sig=254,4 Ref=360,100

Peak #	RetTime [min]	Type	Width [min]	Area [mAU*s]	Height [mAU]	Area %
1	5.990	BB	0.1622	84.53172	7.81489	1.0112
2	7.670	BB	0.2225	8275.04297	566.31616	98.9888

Totals : 8359.57469 574.13105

Signal 4: DAD1 D, Sig=280,4 Ref=360,100

Signal 5: DAD1 E, Sig=310,4 Ref=360,100

*** End of Report ***

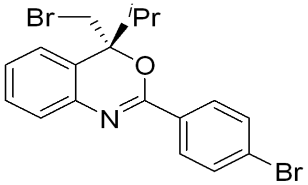

4f
(enantioenriched)

Supplementary Figure 160. HPLC Spectrum of **4f** (enantioenriched)

Data File C:\CHEM32\1\DATA\SUN_12 2022-03-15 20-30-20\TXF-4-4-2RAC.D
Sample Name:

==

Acq. Operator : Seq. Line : 2
Acq. Instrument : Instrument 1 Location : Vial 1
Injection Date : 3/15/2022 8:43:08 PM Inj : 1
Inj Volume : 5.000 µl
Different Inj Volume from Sequence ! Actual Inj Volume : 2.000 µl
Acq. Method : C:\CHEM32\1\DATA\SUN_12 2022-03-15 20-30-20\OD-01-15.M
Last changed : 3/1/2022 2:47:17 PM
Analysis Method : C:\CHEM32\1\METHODS\AD-10-10.M
Last changed : 6/17/2016 2:27:06 PM
Additional Info : Peak(s) manually integrated

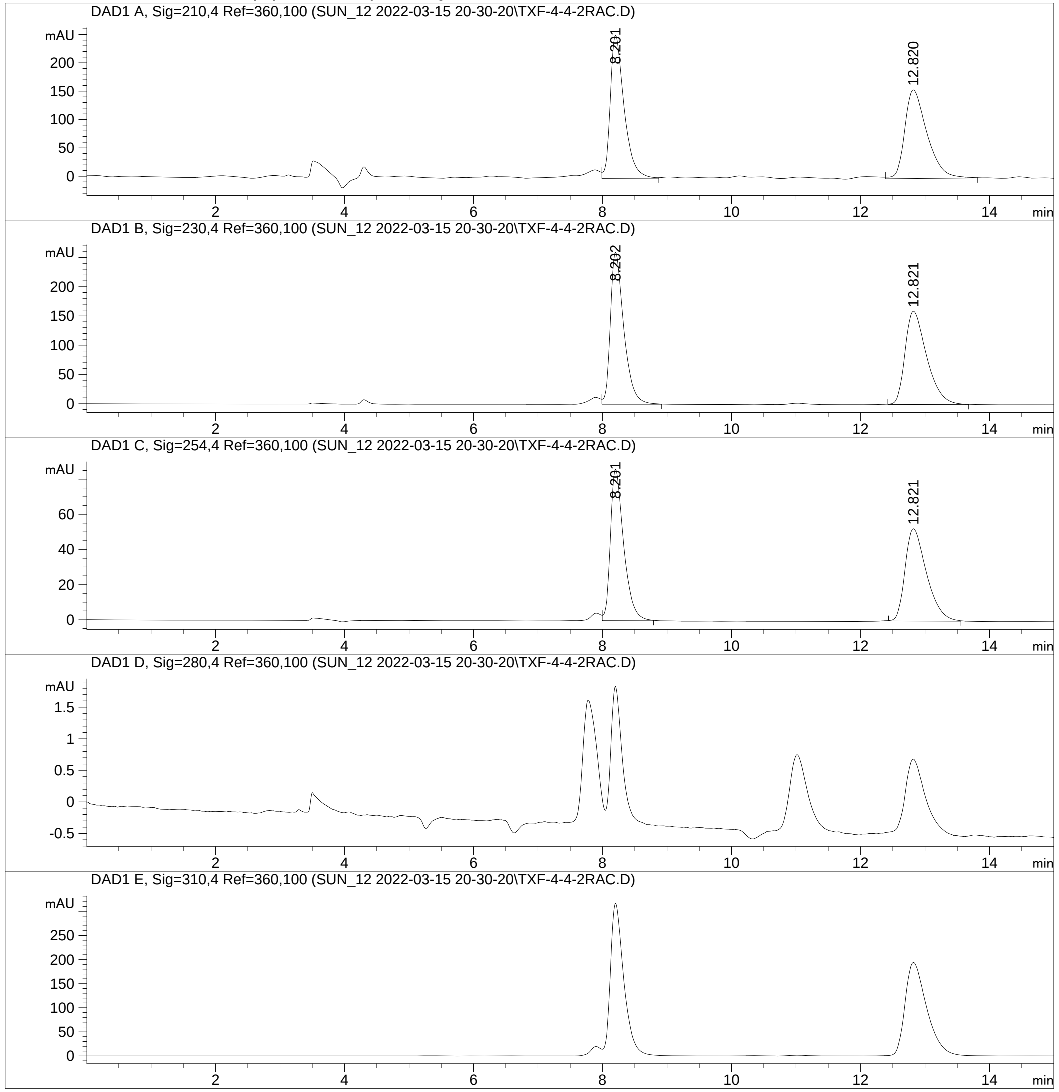

Data File C:\CHEM32\1\DATA\SUN_12 2022-03-15 20-30-20\TXF-4-4-2RAC.D
Sample Name:

==

Area Percent Report

==

Sorted By : Signal
Multiplier : 1.0000
Dilution : 1.0000
Use Multiplier & Dilution Factor with ISTDs

Signal 1: DAD1 A, Sig=210,4 Ref=360,100

Peak #	RetTime [min]	Type	Width [min]	Area [mAU*s]	Height [mAU]	Area %
1	8.201	VB	0.2254	3802.20215	252.95953	50.3164
2	12.820	VB	0.3602	3754.37939	156.30843	49.6836

Totals : 7556.58154 409.26796

Signal 2: DAD1 B, Sig=230,4 Ref=360,100

Peak #	RetTime [min]	Type	Width [min]	Area [mAU*s]	Height [mAU]	Area %
1	8.202	VB	0.2168	3763.59839	260.04791	50.3537
2	12.821	BB	0.3534	3710.71924	159.53140	49.6463

Totals : 7474.31763 419.57932

Signal 3: DAD1 C, Sig=254,4 Ref=360,100

Peak #	RetTime [min]	Type	Width [min]	Area [mAU*s]	Height [mAU]	Area %
1	8.201	VB	0.2152	1235.49988	86.21720	50.4495
2	12.821	BB	0.3514	1213.48450	52.55660	49.5505

Totals : 2448.98438 138.77380

Signal 4: DAD1 D, Sig=280,4 Ref=360,100

Signal 5: DAD1 E, Sig=310,4 Ref=360,100

==

*** End of Report ***

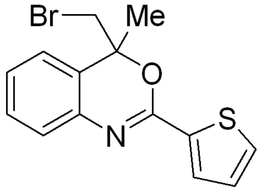

4g
(racemic)

Supplementary Figure 161. HPLC Spectrum of **4g** (racemic)

Data File C:\CHEM32\1\DATA\SUN_12 2022-03-15 20-30-20\TXF-4-4-1.D
Sample Name:

===

Acq. Operator : Seq. Line : 3
Acq. Instrument : Instrument 1 Location : Vial 2
Injection Date : 3/15/2022 8:59:04 PM Inj : 1
Inj Volume : 5.000 µl
Different Inj Volume from Sequence ! Actual Inj Volume : 2.000 µl
Acq. Method : C:\CHEM32\1\DATA\SUN_12 2022-03-15 20-30-20\OD-01-15.M
Last changed : 3/1/2022 2:47:17 PM
Analysis Method : C:\CHEM32\1\METHODS\AD-10-10.M
Last changed : 6/17/2016 2:27:06 PM
Additional Info : Peak(s) manually integrated

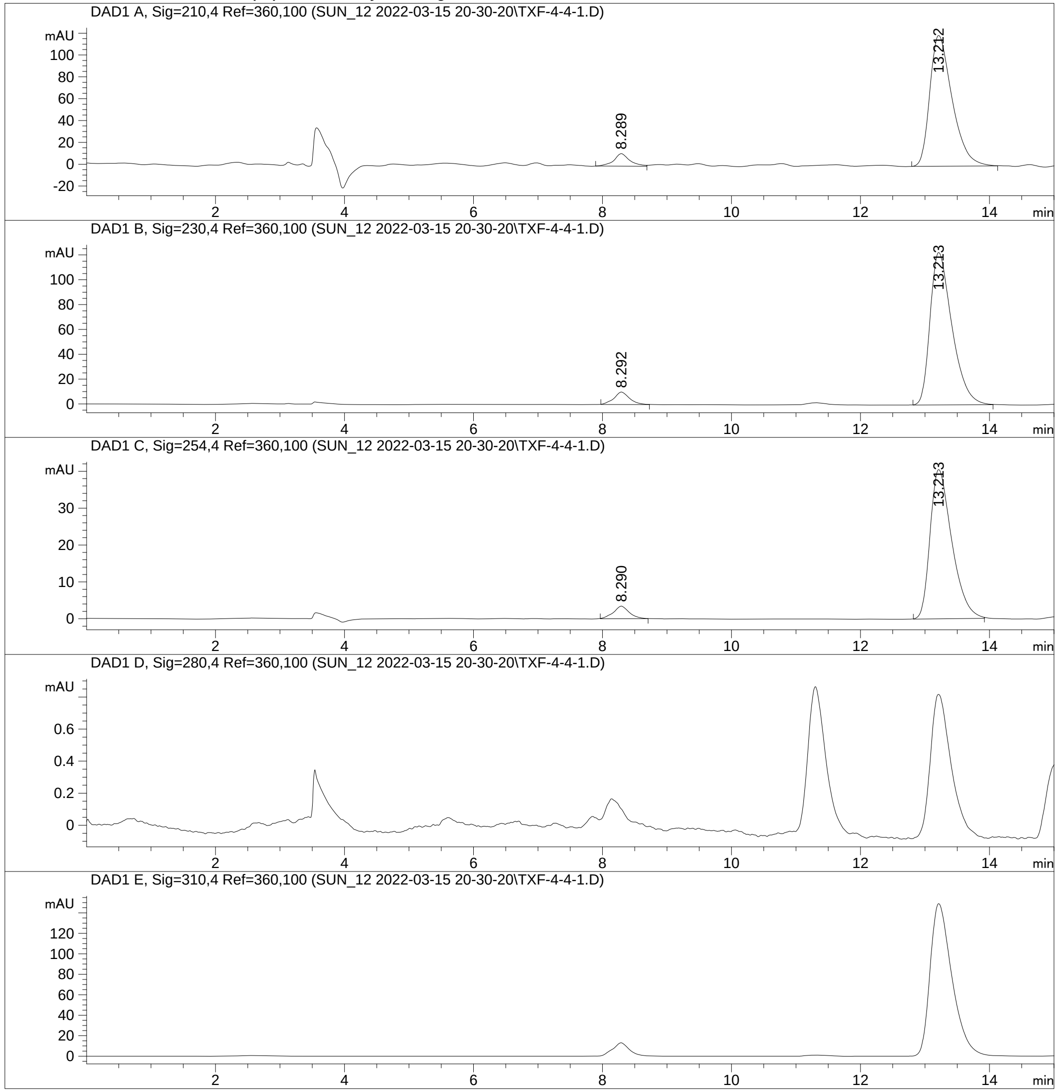

Data File C:\CHEM32\1\DATA\SUN_12 2022-03-15 20-30-20\TXF-4-4-1.D
Sample Name:

===

Area Percent Report

===

Sorted By : Signal
Multiplier : 1.0000
Dilution : 1.0000
Use Multiplier & Dilution Factor with ISTDs

Signal 1: DAD1 A, Sig=210,4 Ref=360,100

Peak #	RetTime [min]	Type	Width [min]	Area [mAU*s]	Height [mAU]	Area %
1	8.289	BV	0.2459	196.17249	11.44881	6.3729
2	13.212	BB	0.3648	2882.04688	119.75896	93.6271
Totals :				3078.21936	131.20777	

Signal 2: DAD1 B, Sig=230,4 Ref=360,100

Peak #	RetTime [min]	Type	Width [min]	Area [mAU*s]	Height [mAU]	Area %
1	8.292	BB	0.2437	168.98502	10.07485	5.5209
2	13.213	BB	0.3589	2891.85010	122.71573	94.4791
Totals :				3060.83511	132.79058	

Signal 3: DAD1 C, Sig=254,4 Ref=360,100

Peak #	RetTime [min]	Type	Width [min]	Area [mAU*s]	Height [mAU]	Area %
1	8.290	BB	0.2473	58.68481	3.43544	5.8246
2	13.213	BB	0.3575	948.84094	40.47426	94.1754
Totals :				1007.52575	43.90969	

Signal 4: DAD1 D, Sig=280,4 Ref=360,100

Signal 5: DAD1 E, Sig=310,4 Ref=360,100

===

*** End of Report ***

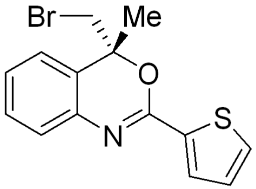

4g
(enantioenriched)

Supplementary Figure 162. HPLC Spectrum of **4g** (enantioenriched)

Data File C:\CHEM32\1\DATA\SUN_12 2022-03-19 20-08-03\TXF-4-6-RAC.D
Sample Name:

```
=====================================================================
Acq. Operator   :                               Seq. Line :   3
Acq. Instrument : Instrument 1                   Location : Vial 2
Injection Date  : 3/19/2022 8:41:49 PM                Inj :   1
                                               Inj Volume : 5.000 µl
Different Inj Volume from Sequence !     Actual Inj Volume : 3.000 µl
Acq. Method     : C:\CHEM32\1\DATA\SUN_12 2022-03-19 20-08-03\OD-01-15.M
Last changed    : 3/1/2022 2:47:17 PM
Analysis Method : C:\CHEM32\1\METHODS\AD-10-10.M
Last changed    : 6/17/2016 2:27:06 PM
Additional Info : Peak(s) manually integrated
```

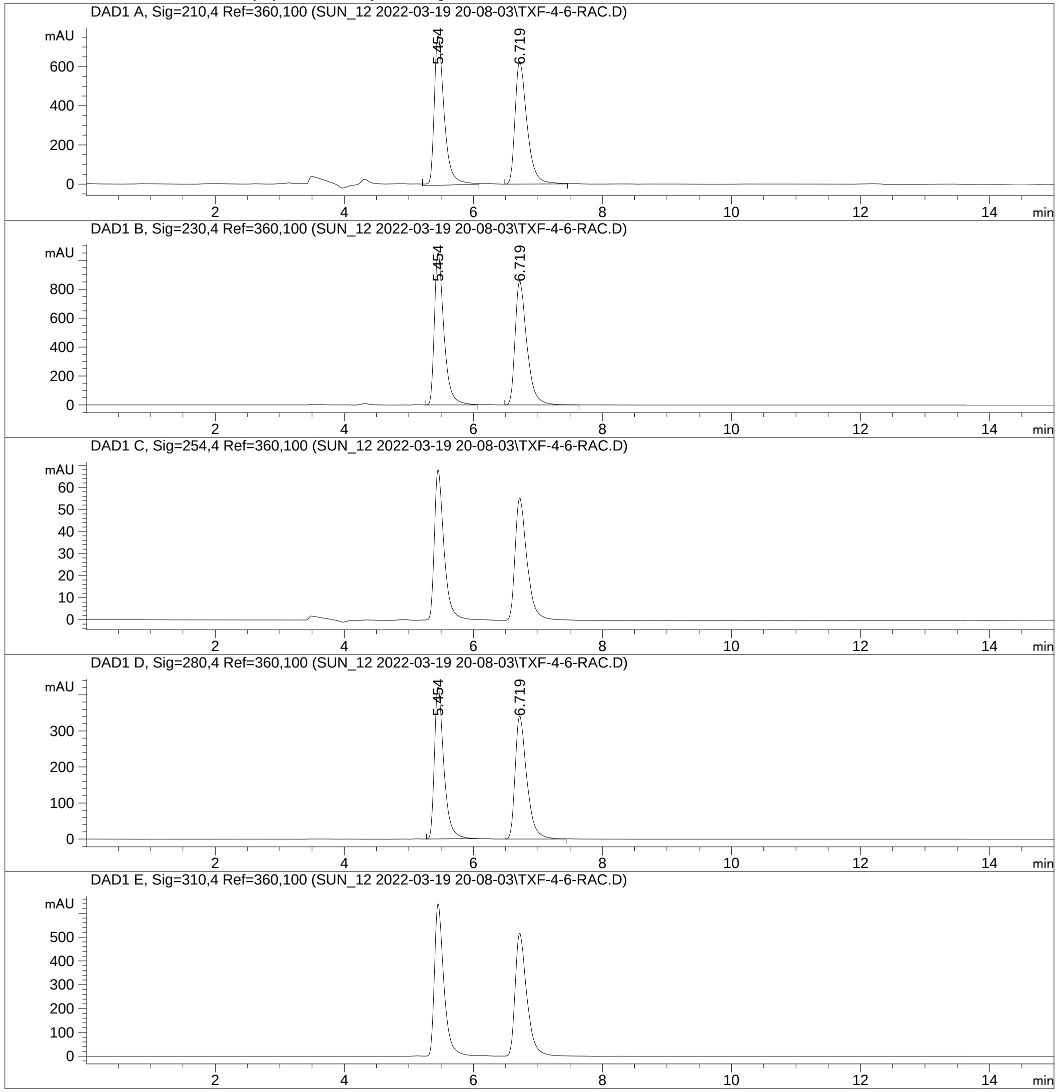

Data File C:\CHEM32\1\DATA\SUN_12 2022-03-19 20-08-03\TXF-4-6-RAC.D
Sample Name:

Area Percent Report

Sorted By : Signal
Multiplier : 1.0000
Dilution : 1.0000
Use Multiplier & Dilution Factor with ISTDs

Signal 1: DAD1 A, Sig=210,4 Ref=360,100

Peak #	RetTime [min]	Type	Width [min]	Area [mAU*s]	Height [mAU]	Area %
1	5.454	VB	0.1610	8196.45410	765.04529	50.3880
2	6.719	BB	0.1963	8070.23926	626.44275	49.6120

Totals : 1.62667e4 1391.48804

Signal 2: DAD1 B, Sig=230,4 Ref=360,100

Peak #	RetTime [min]	Type	Width [min]	Area [mAU*s]	Height [mAU]	Area %
1	5.454	VV	0.1535	1.08003e4	1054.08386	49.9350
2	6.719	BB	0.1895	1.08284e4	856.34821	50.0650

Totals : 2.16287e4 1910.43207

Signal 3: DAD1 C, Sig=254,4 Ref=360,100

Signal 4: DAD1 D, Sig=280,4 Ref=360,100

Peak #	RetTime [min]	Type	Width [min]	Area [mAU*s]	Height [mAU]	Area %
1	5.454	BB	0.1517	4259.95752	422.38513	49.9084
2	6.719	BB	0.1883	4275.59619	340.97195	50.0916

Totals : 8535.55371 763.35709

Signal 5: DAD1 E, Sig=310,4 Ref=360,100

*** End of Report ***

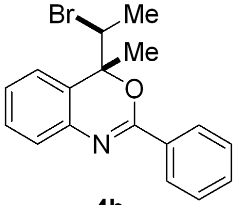

4h
(racemic)

Supplementary Figure 163. HPLC Spectrum of **4h** (racemic)

Data File C:\CHEM32\1\DATA\SUN_12 2022-03-19 20-08-03\TXF-4-6-3.D
Sample Name:

```
==================================================================
Acq. Operator   :                              Seq. Line :   2
Acq. Instrument : Instrument 1                  Location : Vial 1
Injection Date  : 3/19/2022 8:25:51 PM               Inj :   1
                                              Inj Volume : 5.000 µl
Different Inj Volume from Sequence !     Actual Inj Volume : 2.000 µl
Acq. Method     : C:\CHEM32\1\DATA\SUN_12 2022-03-19 20-08-03\OD-01-15.M
Last changed    : 3/1/2022 2:47:17 PM
Analysis Method : C:\CHEM32\1\METHODS\AD-10-10.M
Last changed    : 6/17/2016 2:27:06 PM
Additional Info : Peak(s) manually integrated
```

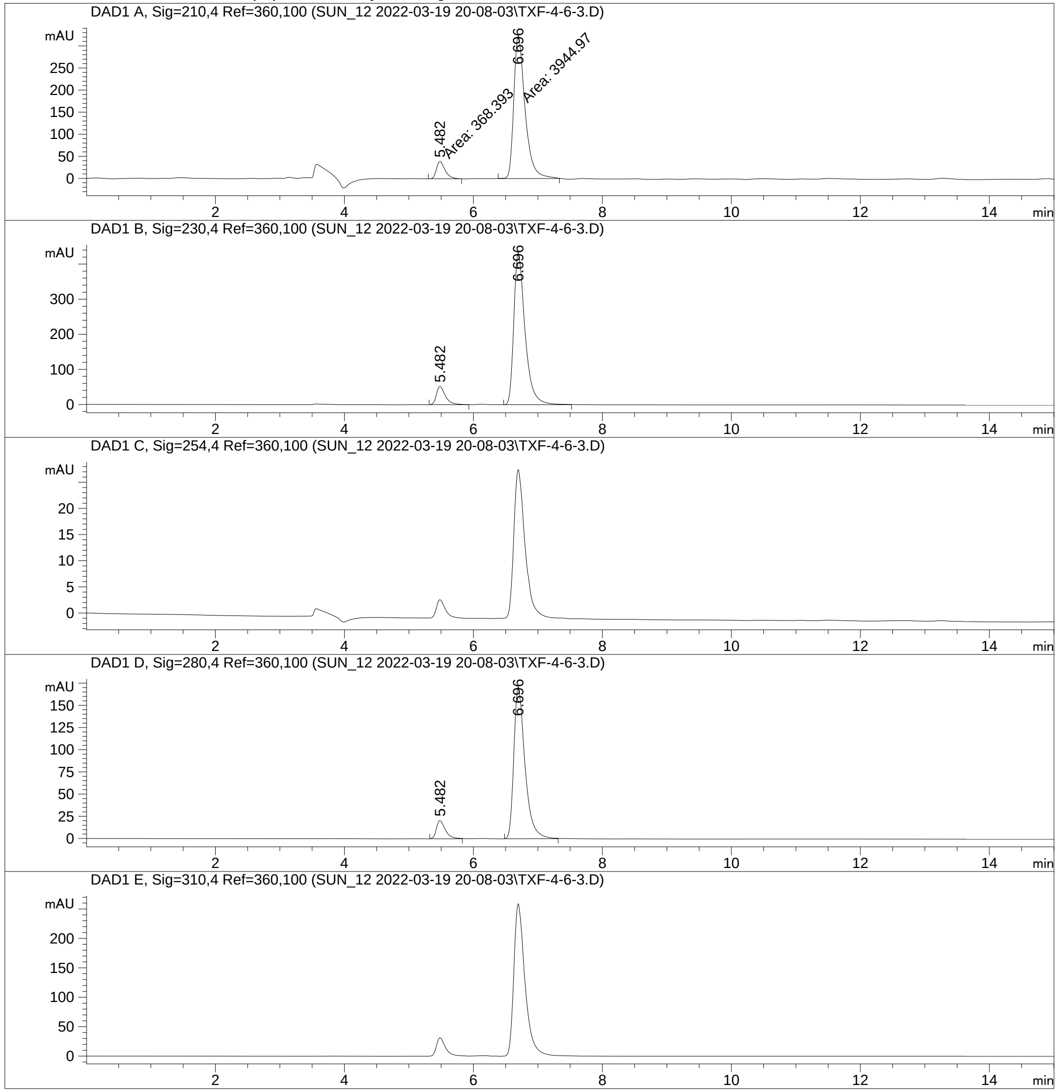

Instrument 1 4/1/2022 10:07:51 PM Page 1 of 2

Data File C:\CHEM32\1\DATA\SUN_12 2022-03-19 20-08-03\TXF-4-6-3.D
Sample Name:

```
==================================================================
                     Area Percent Report
==================================================================

Sorted By             :      Signal
Multiplier            :      1.0000
Dilution              :      1.0000
Use Multiplier & Dilution Factor with ISTDs


Signal 1: DAD1 A, Sig=210,4 Ref=360,100

Peak RetTime Type  Width     Area      Height     Area
  #   [min]        [min]   [mAU*s]     [mAU]        %
----|-------|----|-------|----------|----------|--------|
   1   5.482 MM    0.1562  368.39282   39.29974   8.5407
   2   6.696 MM    0.2028 3944.96973  324.22867  91.4593

Totals :                  4313.36255  363.52840


Signal 2: DAD1 B, Sig=230,4 Ref=360,100

Peak RetTime Type  Width     Area      Height     Area
  #   [min]        [min]   [mAU*s]     [mAU]        %
----|-------|----|-------|----------|----------|--------|
   1   5.482 BB    0.1463  513.64130   52.44886   8.9332
   2   6.696 BB    0.1828 5236.18652  433.73135  91.0668

Totals :                  5749.82782  486.18021


Signal 3: DAD1 C, Sig=254,4 Ref=360,100


Signal 4: DAD1 D, Sig=280,4 Ref=360,100

Peak RetTime Type  Width     Area      Height     Area
  #   [min]        [min]   [mAU*s]     [mAU]        %
----|-------|----|-------|----------|----------|--------|
   1   5.482 BB    0.1445  198.66734   20.60723   8.8128
   2   6.696 BB    0.1820 2055.63525  171.34473  91.1872

Totals :                  2254.30260  191.95196


Signal 5: DAD1 E, Sig=310,4 Ref=360,100

==================================================================
                     *** End of Report ***
```

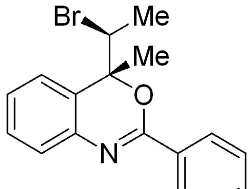

4h
(enantioenriched)

Supplementary Figure 164. HPLC Spectrum of **4h** (enantioenriched)

Instrument 1 4/1/2022 10:07:51 PM Page 2 of 2

```
=====================================================================
Acq. Operator   :                               Seq. Line :   2
Acq. Instrument : Instrument 1                   Location : Vial 1
Injection Date  : 3/13/2022 7:07:01 PM                Inj :   1
                                               Inj Volume : 5.000 µl
Different Inj Volume from Sequence !     Actual Inj Volume : 2.000 µl
Acq. Method     : C:\CHEM32\1\DATA\SUN_12 2022-03-13 18-54-13\OD-01-15.M
Last changed    : 3/1/2022 2:47:17 PM
Analysis Method : C:\CHEM32\1\METHODS\AD-10-10.M
Last changed    : 6/17/2016 2:27:06 PM
Additional Info : Peak(s) manually integrated
```

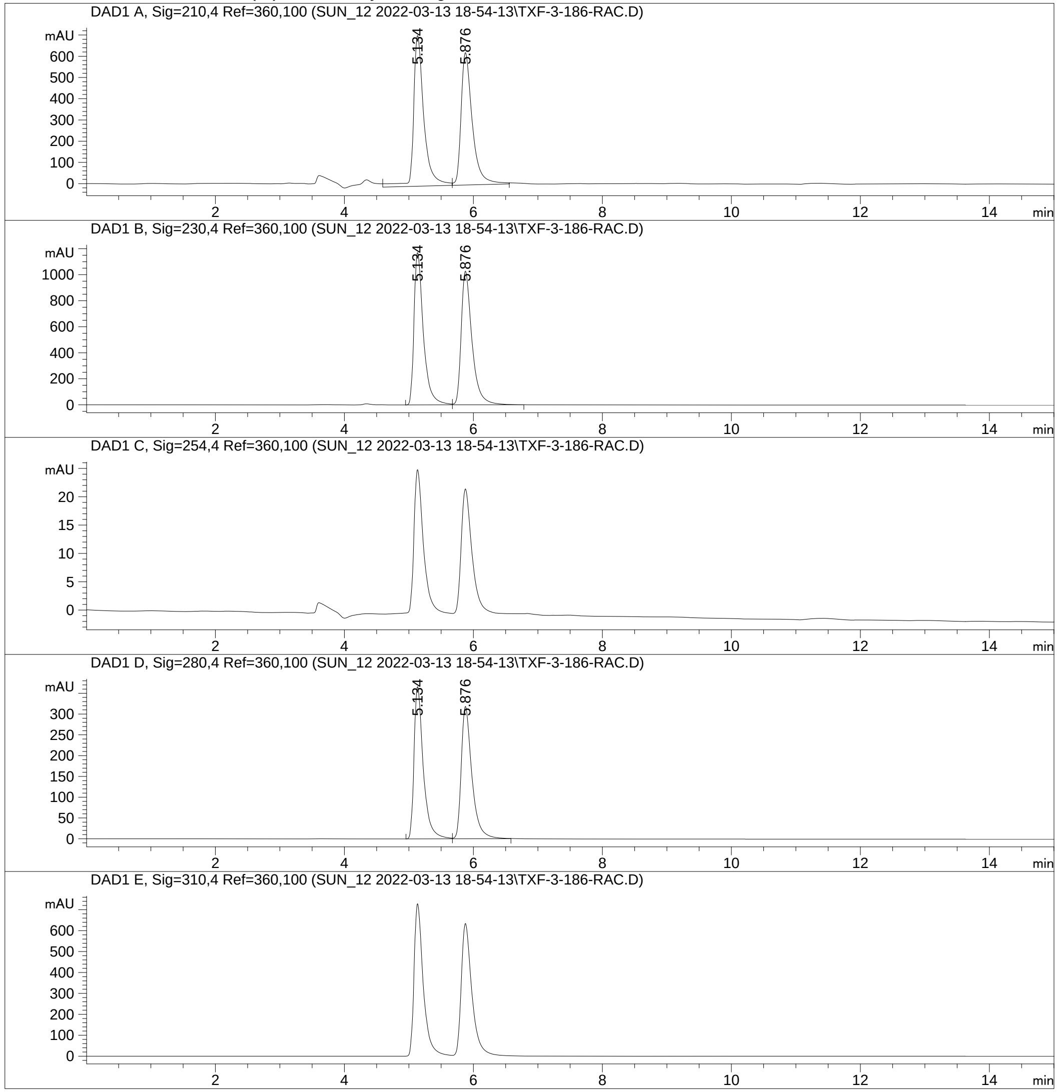

Data File C:\CHEM32\1\DATA\SUN_12 2022-03-13 18-54-13\TXF-3-186-RAC.D
Sample Name:

```
=====================================================================
                         Area Percent Report
=====================================================================

Sorted By             :      Signal
Multiplier            :      1.0000
Dilution              :      1.0000
Use Multiplier & Dilution Factor with ISTDs
```

Signal 1: DAD1 A, Sig=210,4 Ref=360,100

Peak #	RetTime [min]	Type	Width [min]	Area [mAU*s]	Height [mAU]	Area %
1	5.134	VV	0.1645	7944.72607	710.16632	51.2420
2	5.876	VB	0.1814	7559.60986	623.87708	48.7580

Totals : 1.55043e4 1334.04340

Signal 2: DAD1 B, Sig=230,4 Ref=360,100

Peak #	RetTime [min]	Type	Width [min]	Area [mAU*s]	Height [mAU]	Area %
1	5.134	BV	0.1516	1.18067e4	1171.25696	49.6839
2	5.876	VB	0.1737	1.19570e4	1028.36084	50.3161

Totals : 2.37637e4 2199.61780

Signal 3: DAD1 C, Sig=254,4 Ref=360,100

Signal 4: DAD1 D, Sig=280,4 Ref=360,100

Peak #	RetTime [min]	Type	Width [min]	Area [mAU*s]	Height [mAU]	Area %
1	5.134	BV	0.1477	3633.88550	366.29724	49.8803
2	5.876	VB	0.1717	3651.31934	318.61270	50.1197

Totals : 7285.20483 684.90994

Signal 5: DAD1 E, Sig=310,4 Ref=360,100

===
*** End of Report ***

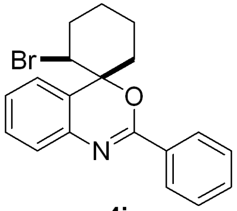

4i
(racemic)

Supplementary Figure 165. HPLC Spectrum of **4i** (racemic)

Data File C:\CHEM32\1\DATA\SUN_12 2022-03-13 18-54-13\TXF-3-186-1.D
Sample Name:

```
=====================================================================
Acq. Operator   :                               Seq. Line :   3
Acq. Instrument : Instrument 1                   Location : Vial 2
Injection Date  : 3/13/2022 7:22:57 PM                Inj :   1
                                               Inj Volume : 5.000 µl
Different Inj Volume from Sequence !     Actual Inj Volume : 2.000 µl
Acq. Method     : C:\CHEM32\1\DATA\SUN_12 2022-03-13 18-54-13\OD-01-15.M
Last changed    : 3/1/2022 2:47:17 PM
Analysis Method : C:\CHEM32\1\METHODS\AD-10-10.M
Last changed    : 6/17/2016 2:27:06 PM
Additional Info : Peak(s) manually integrated
```

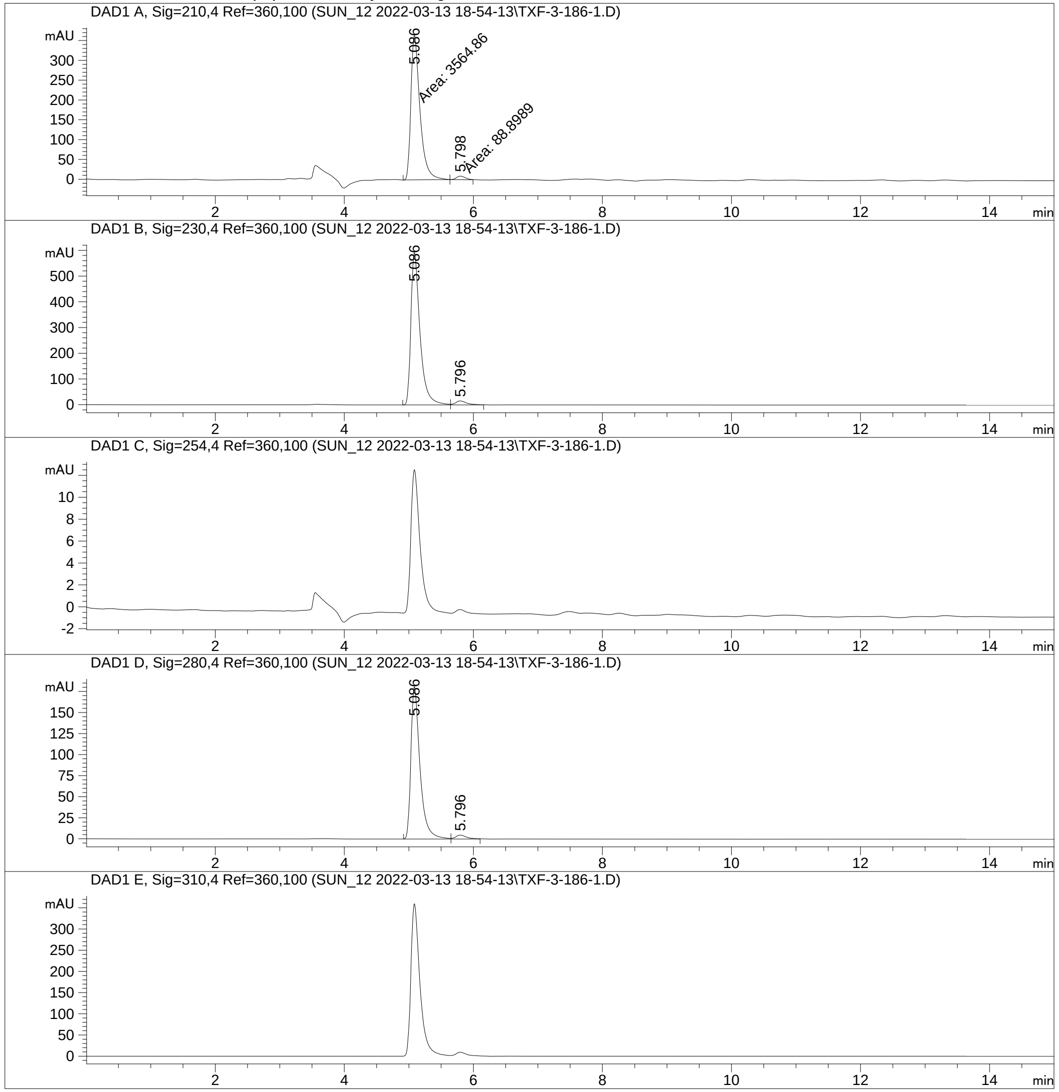

Data File C:\CHEM32\1\DATA\SUN_12 2022-03-13 18-54-13\TXF-3-186-1.D
Sample Name:

Area Percent Report

Sorted By : Signal
Multiplier : 1.0000
Dilution : 1.0000
Use Multiplier & Dilution Factor with ISTDs

Signal 1: DAD1 A, Sig=210,4 Ref=360,100

Peak #	RetTime [min]	Type	Width [min]	Area [mAU*s]	Height [mAU]	Area %
1	5.086	MM	0.1630	3564.85718	364.53656	97.5669
2	5.798	MM	0.1607	88.89892	9.22106	2.4331
Totals :				3653.75610	373.75762	

Signal 2: DAD1 B, Sig=230,4 Ref=360,100

Peak #	RetTime [min]	Type	Width [min]	Area [mAU*s]	Height [mAU]	Area %
1	5.086	BV	0.1443	5801.79590	592.26190	96.9536
2	5.796	VB	0.1717	182.30125	15.67550	3.0464
Totals :				5984.09715	607.93740	

Signal 3: DAD1 C, Sig=254,4 Ref=360,100

Signal 4: DAD1 D, Sig=280,4 Ref=360,100

Peak #	RetTime [min]	Type	Width [min]	Area [mAU*s]	Height [mAU]	Area %
1	5.086	BB	0.1439	1766.49915	180.90895	97.0579
2	5.796	BB	0.1687	53.54794	4.70909	2.9421
Totals :				1820.04708	185.61804	

Signal 5: DAD1 E, Sig=310,4 Ref=360,100

*** End of Report ***

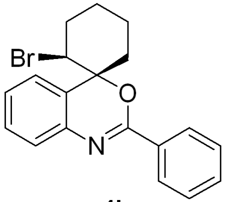

4i
(enantioenriched)

Supplementary Figure 166. HPLC Spectrum of **4i** (enantioenriched)

==

Acq. Operator : Seq. Line : 2
Acq. Instrument : Instrument 1 Location : Vial 8
Injection Date : 4/15/2022 9:39:49 PM Inj : 1
Inj Volume : 5.000 µl
Different Inj Volume from Sequence ! Actual Inj Volume : 2.000 µl
Acq. Method : C:\CHEM32\1\DATA\SUN_12 2022-04-15 21-27-00\OD-07-15.M
Last changed : 4/22/2021 5:33:37 PM
Analysis Method : C:\CHEM32\1\METHODS\OJ-10-10.M
Last changed : 5/7/2018 4:44:08 PM
Additional Info : Peak(s) manually integrated

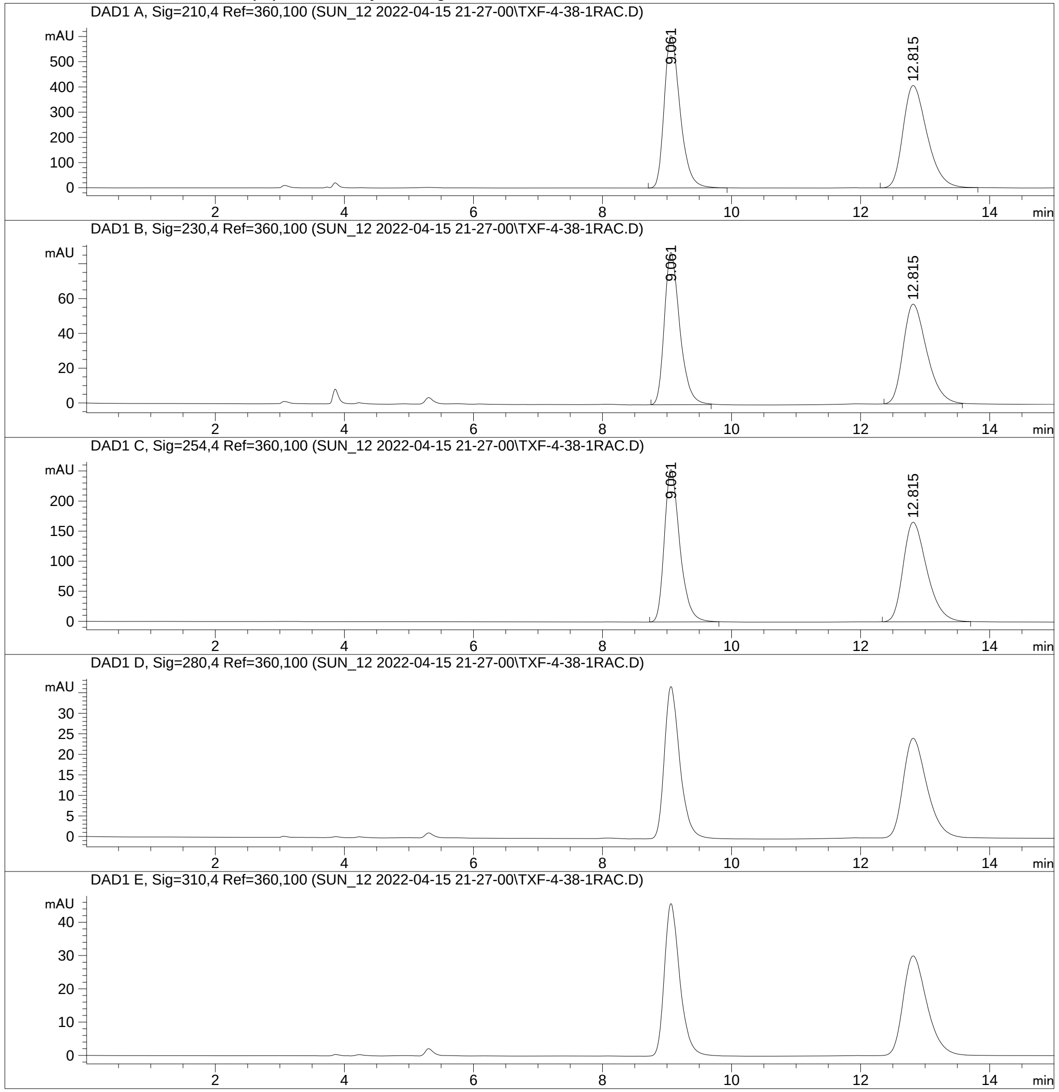

Data File C:\CHEM32\1\DATA\SUN_12 2022-04-15 21-27-00\TXF-4-38-1RAC.D
Sample Name:

==

Area Percent Report

==

Sorted By : Signal
Multiplier : 1.0000
Dilution : 1.0000
Use Multiplier & Dilution Factor with ISTDs

Signal 1: DAD1 A, Sig=210,4 Ref=360,100

Peak #	RetTime [min]	Type	Width [min]	Area [mAU*s]	Height [mAU]	Area %
1	9.061	BB	0.2590	1.03159e4	604.80383	49.5411
2	12.815	BB	0.3984	1.05069e4	405.39697	50.4589

Totals : 2.08228e4 1010.20081

Signal 2: DAD1 B, Sig=230,4 Ref=360,100

Peak #	RetTime [min]	Type	Width [min]	Area [mAU*s]	Height [mAU]	Area %
1	9.061	BB	0.2524	1458.34705	87.52996	49.8964
2	12.815	BB	0.3919	1464.40356	57.34204	50.1036

Totals : 2922.75061 144.87200

Signal 3: DAD1 C, Sig=254,4 Ref=360,100

Peak #	RetTime [min]	Type	Width [min]	Area [mAU*s]	Height [mAU]	Area %
1	9.061	BB	0.2524	4227.25439	253.64980	49.9155
2	12.815	BB	0.3925	4241.55957	165.75102	50.0845

Totals : 8468.81396 419.40082

Signal 4: DAD1 D, Sig=280,4 Ref=360,100

Signal 5: DAD1 E, Sig=310,4 Ref=360,100

==

*** End of Report ***

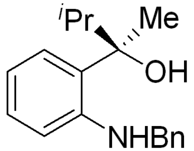

5
(racemic)

Supplementary Figure 167. HPLC Spectrum of **5** (racemic)

===

Acq. Operator : Seq. Line : 3
Acq. Instrument : Instrument 1 Location : Vial 9
Injection Date : 4/15/2022 9:55:49 PM Inj : 1
Inj Volume : 5.000 µl
Different Inj Volume from Sequence ! Actual Inj Volume : 2.000 µl
Acq. Method : C:\CHEM32\1\DATA\SUN_12 2022-04-15 21-27-00\OD-07-15.M
Last changed : 4/22/2021 5:33:37 PM
Analysis Method : C:\CHEM32\1\METHODS\OJ-10-10.M
Last changed : 5/7/2018 4:44:08 PM
Additional Info : Peak(s) manually integrated

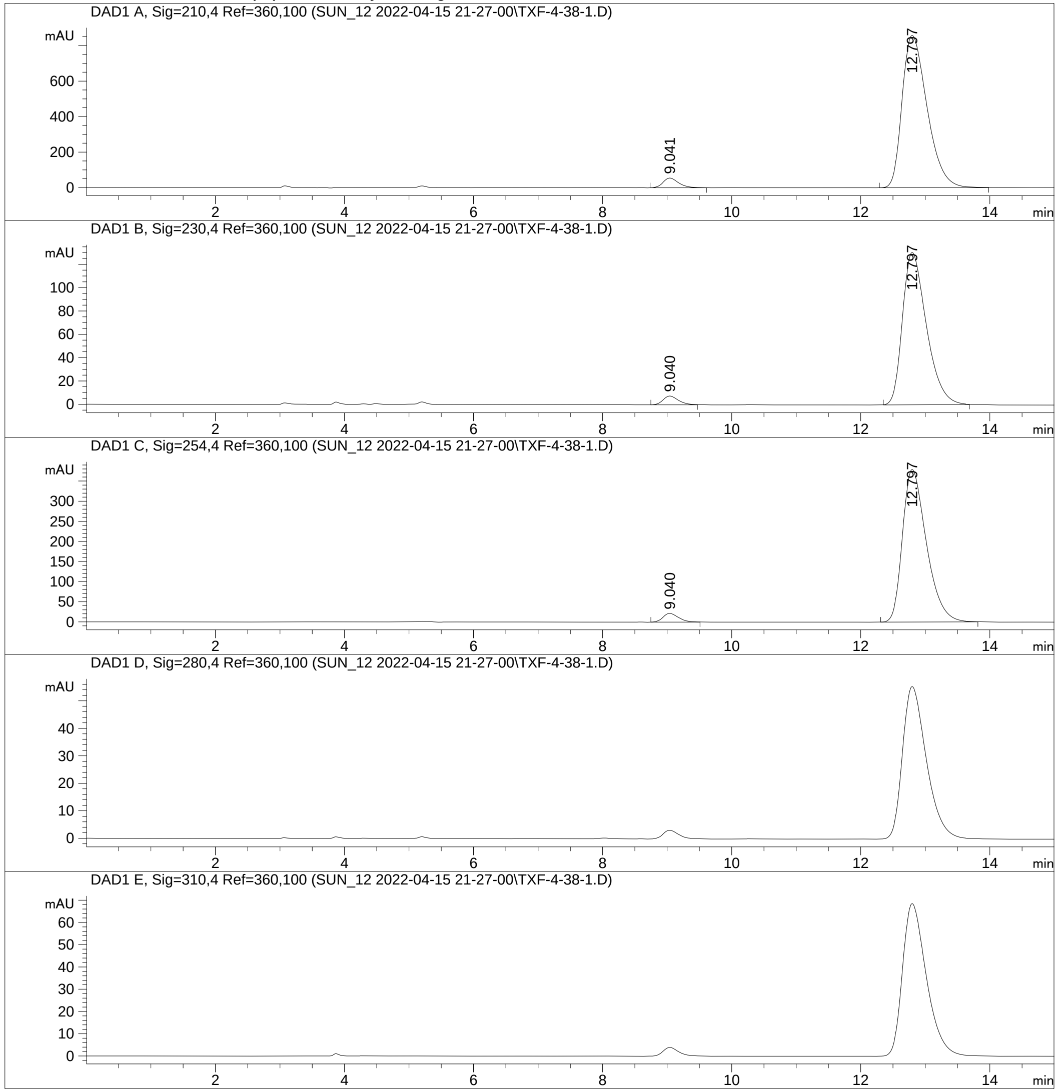

Data File C:\CHEM32\1\DATA\SUN_12 2022-04-15 21-27-00\TXF-4-38-1.D
Sample Name:

===

Area Percent Report

===

Sorted By : Signal
Multiplier : 1.0000
Dilution : 1.0000
Use Multiplier & Dilution Factor with ISTDs

Signal 1: DAD1 A, Sig=210,4 Ref=360,100

Peak #	RetTime [min]	Type	Width [min]	Area [mAU*s]	Height [mAU]	Area %
1	9.041	BB	0.2555	920.36237	54.92960	3.8341
2	12.797	BB	0.4200	2.30842e4	857.81238	96.1659

Totals : 2.40045e4 912.74197

Signal 2: DAD1 B, Sig=230,4 Ref=360,100

Peak #	RetTime [min]	Type	Width [min]	Area [mAU*s]	Height [mAU]	Area %
1	9.040	BB	0.2505	124.36388	7.53859	3.5702
2	12.797	BB	0.3942	3359.02832	130.54863	96.4298

Totals : 3483.39220 138.08722

Signal 3: DAD1 C, Sig=254,4 Ref=360,100

Peak #	RetTime [min]	Type	Width [min]	Area [mAU*s]	Height [mAU]	Area %
1	9.040	BB	0.2536	360.95044	21.74783	3.5682
2	12.797	BB	0.3943	9754.82129	378.92462	96.4318

Totals : 1.01158e4 400.67245

Signal 4: DAD1 D, Sig=280,4 Ref=360,100

Signal 5: DAD1 E, Sig=310,4 Ref=360,100

===

*** End of Report ***

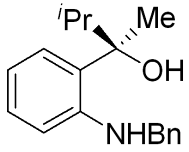

5
(enantioenriched)

Supplementary Figure 168. HPLC Spectrum of **5** (enantioenriched)

Data File C:\CHEM32\1\DATA\SUN_12 2022-04-23 16-06-16\TXF-4-51-RAC.D
Sample Name:

===

Acq. Operator : Seq. Line : 2
Acq. Instrument : Instrument 1 Location : Vial 10
Injection Date : 4/23/2022 4:19:05 PM Inj : 1
Inj Volume : 5.000 µl
Different Inj Volume from Sequence ! Actual Inj Volume : 2.000 µl
Acq. Method : C:\CHEM32\1\DATA\SUN_12 2022-04-23 16-06-16\OD-02-10.M
Last changed : 4/23/2022 4:06:23 PM
(modified after loading)
Analysis Method : C:\CHEM32\1\METHODS\OJ-10-10.M
Last changed : 5/7/2018 4:44:08 PM
Additional Info : Peak(s) manually integrated

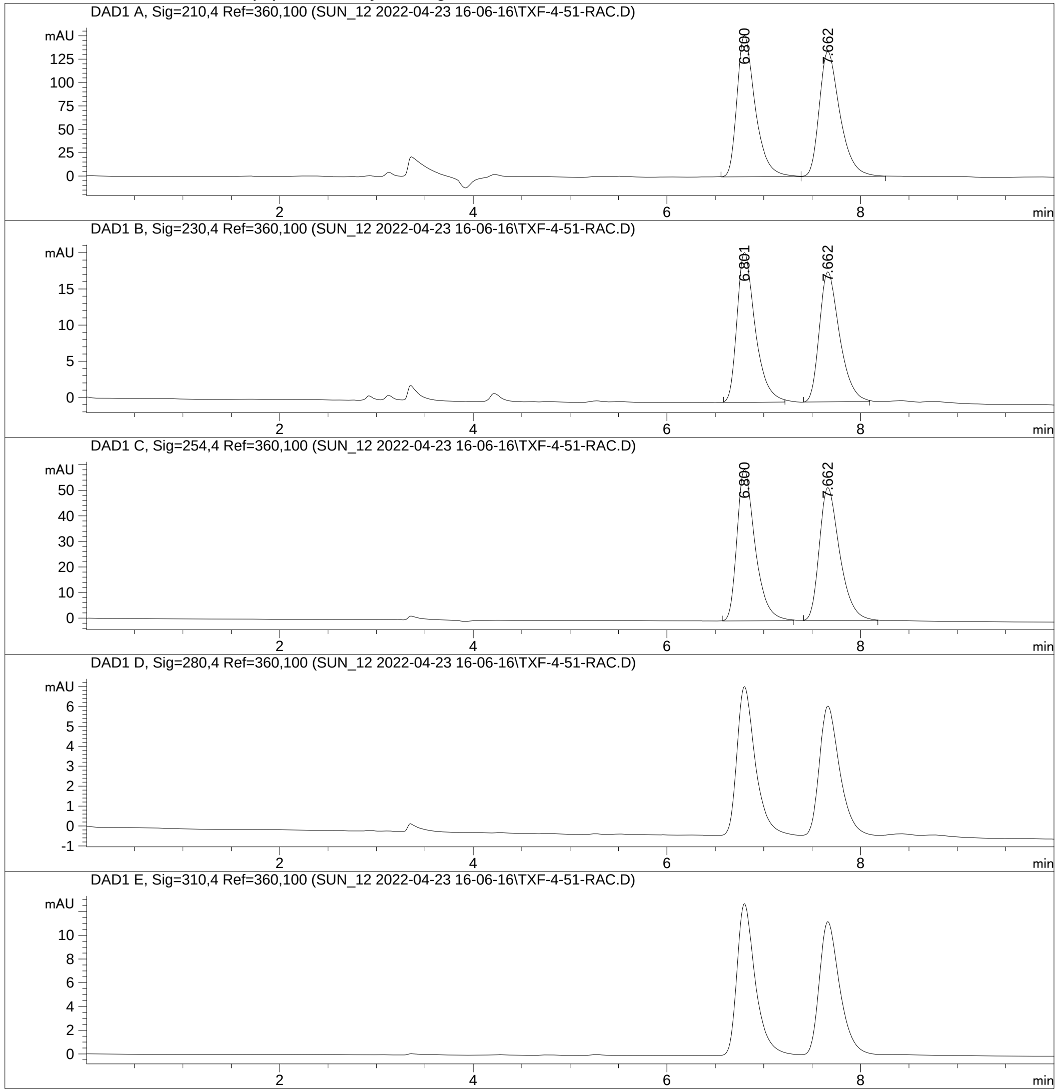

Data File C:\CHEM32\1\DATA\SUN_12 2022-04-23 16-06-16\TXF-4-51-RAC.D
Sample Name:

===

Area Percent Report

===

Sorted By : Signal
Multiplier : 1.0000
Dilution : 1.0000
Use Multiplier & Dilution Factor with ISTDs

Signal 1: DAD1 A, Sig=210,4 Ref=360,100

Peak #	RetTime [min]	Type	Width [min]	Area [mAU*s]	Height [mAU]	Area %
1	6.800	BV	0.1991	1983.24072	151.15863	50.0980
2	7.662	VB	0.2246	1975.48328	133.53168	49.9020

Totals : 3958.72400 284.69031

Signal 2: DAD1 B, Sig=230,4 Ref=360,100

Peak #	RetTime [min]	Type	Width [min]	Area [mAU*s]	Height [mAU]	Area %
1	6.801	BB	0.1967	271.36948	20.73533	51.0161
2	7.662	BB	0.2211	260.55963	17.98016	48.9839

Totals : 531.92911 38.71549

Signal 3: DAD1 C, Sig=254,4 Ref=360,100

Peak #	RetTime [min]	Type	Width [min]	Area [mAU*s]	Height [mAU]	Area %
1	6.800	BB	0.1960	771.70923	59.22691	50.2837
2	7.662	BB	0.2229	762.99988	52.07290	49.7163

Totals : 1534.70911 111.29981

Signal 4: DAD1 D, Sig=280,4 Ref=360,100

Signal 5: DAD1 E, Sig=310,4 Ref=360,100

===

*** End of Report ***

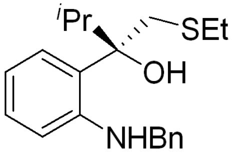

6
(racemic)

Supplementary Figure 169. HPLC Spectrum of **6** (racemic)

```
Data File C:\CHEM32\1\DATA\SUN_12 2022-04-23 16-06-16\TXF-4-51-3.D
Sample Name:

    =====================================================================
    Acq. Operator   :                                 Seq. Line :   3
    Acq. Instrument : Instrument 1                     Location : Vial 9
    Injection Date  : 4/23/2022 4:30:06 PM                  Inj :   1
                                                     Inj Volume : 5.000 µl
    Different Inj Volume from Sequence !     Actual Inj Volume : 2.000 µl
    Acq. Method     : C:\CHEM32\1\DATA\SUN_12 2022-04-23 16-06-16\OD-02-10.M
    Last changed    : 4/23/2022 4:06:23 PM
                      (modified after loading)
    Analysis Method : C:\CHEM32\1\METHODS\OJ-10-10.M
    Last changed    : 5/7/2018 4:44:08 PM
    Additional Info : Peak(s) manually integrated
```

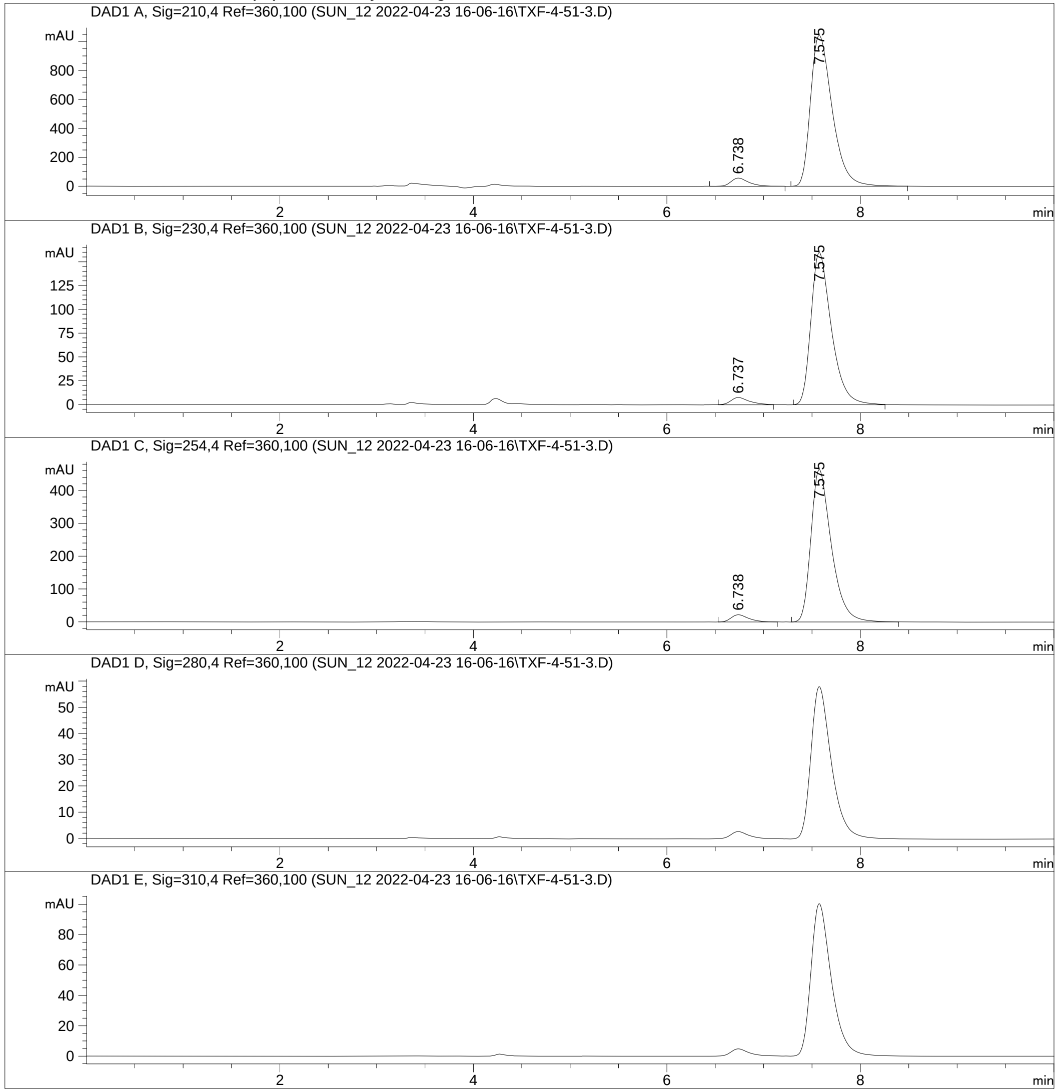

Data File C:\CHEM32\1\DATA\SUN_12 2022-04-23 16-06-16\TXF-4-51-3.D
Sample Name:

Area Percent Report

```
Sorted By             :      Signal
Multiplier            :      1.0000
Dilution              :      1.0000
Use Multiplier & Dilution Factor with ISTDs
```

Signal 1: DAD1 A, Sig=210,4 Ref=360,100

Peak #	RetTime [min]	Type	Width [min]	Area [mAU*s]	Height [mAU]	Area %
1	6.738	BB	0.1907	722.44348	56.69535	4.2562
2	7.575	BB	0.2400	1.62514e4	1041.93286	95.7438
Totals :				1.69739e4	1098.62821	

Signal 2: DAD1 B, Sig=230,4 Ref=360,100

Peak #	RetTime [min]	Type	Width [min]	Area [mAU*s]	Height [mAU]	Area %
1	6.737	BB	0.1980	102.76078	7.68377	4.1786
2	7.575	BB	0.2218	2356.43701	159.99257	95.8214
Totals :				2459.19779	167.67634	

Signal 3: DAD1 C, Sig=254,4 Ref=360,100

Peak #	RetTime [min]	Type	Width [min]	Area [mAU*s]	Height [mAU]	Area %
1	6.738	BB	0.1900	279.70596	22.05466	3.9389
2	7.575	BB	0.2216	6821.40723	463.86334	96.0611
Totals :				7101.11319	485.91800	

Signal 4: DAD1 D, Sig=280,4 Ref=360,100

Signal 5: DAD1 E, Sig=310,4 Ref=360,100

*** End of Report ***

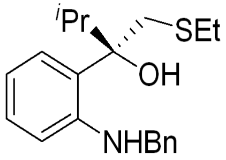

6
(enantioenriched)

Supplementary Figure 170. HPLC Spectrum of **6** (enantioenriched)
